# Unconventional *exo* selectivity in thermal normal-electron-demand Diels–Alder reactions

**DOI:** 10.1038/srep35147

**Published:** 2016-10-12

**Authors:** Guo-Ming Ho, Ci-Jhang Huang, Elise Yu-Tzu Li, Sheng-Kai Hsu, Ti Wu, Medel Manuel L. Zulueta, Kevin Binchia Wu, Shang-Cheng Hung

**Affiliations:** 1 Genomics Research Center, Academia Sinica, 128, Section 2, Academia Road, Taipei 115, Taiwan; 2 Department of Chemistry, National Taiwan Normal University, 88, Section 4, Ting-Chow Road, Taipei 116, Taiwan

## Abstract

The Diels–Alder reaction is a useful tool for generating functionalized chiral molecules through the concerted cycloaddition of dienes and dienophiles leading to six-membered rings. Traditionally, the selective predictions of the products rely heavily on consideration of the secondary orbital interactions that stabilize the *endo* pathway. However, there remain some basic examples defying this notion and produce the *exo*-isomer as major product. Here we systematically evaluated of the structural features driving *exo* selectivity in thermal normal-electron-demand Diels–Alder reactions. Substitution at the Cβ position and the size and electronegativity of the electron-withdrawing group of the dienophile are contributing factors. Experimental and computational studies both point toward the steric and electrostatic forces between the substituents in both the diene and the dienophile that increase the likelihood of the *exo* pathway. For these substrates, the dominance of the *endo* pathway is reduced by transition state distortions and poor structural alignments of the reacting partners. We also noted the tilt of the dienophile with respect to the diene causing steric strain on the functionalities at the more advanced bond forming carbon-carbon position of the *endo* transition state. Insights into such factors may benefit synthetic planning and asserting control over this important named reaction.

Since Otto Diels and Kurt Alder announced their discovery of the pericyclic reaction involving dienes and dienophiles in 1928^1^, the Diels–Alder reaction has been intensively developed and refined to become one of the most powerful carbon–carbon bond forming methods in organic chemistry^2^. This reaction enables the simultaneous regioselective formation of two σ bonds leading to six-membered rings, thereby establishing up to four stereogenic centers in a single step. Such elegant potential to control the regio- and stereochemical outcomes of inter- and intramolecular [4 + 2] cycloadditions subsequently proved a valuable resource for constructing many complex biologically active molecules and natural products^3^,^4^,^5^,^6^.

Normal-electron-demand Diels–Alder cycloaddition can be achieved by the interaction of the highest occupied molecular orbital of an electron-rich diene (e.g., **1**) and the lowest unoccupied molecular orbital of an electron-deficient dienophile (e.g., **2**) generating the *exo* and *endo* transition states (e.g., **3** and **4**, respectively) (Fig. 1). Since Woodward and Hoffmann’s proposal for secondary orbital interactions^7^, *endo* selectivity has been regarded as rather predictable and considered a familiar attribute of these reactions. Thus, while *exo* addition seemed preferable over the more sterically hindered *endo* approach, secondary orbital interactions in the *endo* transition state **4** is understood to promote the formation of the *endo* cycloadduct **6** as the major product^8^. Correspondingly, *exo*-selective Diels–Alder cycloadditions occur less frequently, be it under plain thermal^9^,^10^,^11^,^12^,^13^,^14^,^15^ or catalyzed^13^,^16^,^17^,^18^,^19^,^20^,^21^,^22^,^23^,^24^,^25^,^26^,^27^,^28^,^29^,^30^,^31^ conditions. In many of these cases, suitably positioned structural features from the added catalyst or from the substrates themselves apparently override the factors that stabilize the *endo* transition state.

While preparing several versatile cyclohexanones using thermal Diels–Alder reaction, we noted some interesting cases of *exo* selectivity. Cycloaddition of the silylated dienes **7**^32^ and **8**^33^ with cinnamonitrile (**9**) at 140 °C for 12 h surprisingly both gave the *exo*-adducts **exo-10** and **exo-11** as major coupling products, respectively (Fig. 2a). The structures of these *exo*-adducts were confirmed by X-ray crystallographic analysis (Fig. 2b,c). The stereochemical relationships of the contiguous carbons on the cyclohexene ring of the adducts were also characterized by NMR coupling constant analysis and nuclear Overhauser effect correlation experiments (see Supplementary Methods). Isomerization of the kinetic *endo*-adduct toward the thermodynamically more stable *exo*-adduct, however, remained a possibility^34^. If such *endo*-to-*exo* isomerization occurred, the *endo*-product should accumulate at the early stage of the reaction before eventually decreasing in proportion to the *exo* isomer. However, we noted by NMR monitoring that the apparent ratios of the *exo*- and *endo*-adducts remained almost constant throughout the process (measured at 0.5, 1, 2, 4, 8, and 12 h) (Supplementary Fig. 1). Exposure of the *endo*-adduct to the same reaction conditions for 4 h also did not produce the *exo* counterpart (Supplementary Fig. 2). In light of these results, we concluded that no *endo*-to-*exo* transformation occurred in our reactions.

Houk and co-workers also found that some Diels–Alder reactions of α,β-unsaturated *N*-acyloxazolidinones under Lewis acid-catalyzed conditions were *exo*-selective^25^. They suggested that methyl substitution at both C1 of the silylated diene and Cβ of the dienophile (for the carbon designations used in this paper, refer to Fig. 1) are necessary features that support *exo* selectivity. The lack of substituents at either of those carbons, which hold the shorter of the two σ bonds being formed in the transition states in such concerted asynchronous reactions, largely resulted in *endo* preference. As they described in these cases, the catalyst orientation likely exerted steric influence at the transition state and was partly responsible for the ultimate isomer preference.

The apparent similarity and deviation (i.e., *exo* preference for the cycloaddition of **8** and **9**) of our initial results from Houk’s work stimulated us to study the structural factors that influence *exo* and *endo* stereoselectivity in thermal noncatalyzed Diels–Alder reactions involving dienes **7** and **8**. To flesh out the fundamental source of the stereoselectivity, we used simple dienophiles with different electron-withdrawing functionalities such as nitrile, aldehyde, ketone, ester and nitro groups. Lewis acid catalysts were not employed to avoid any intervening chelation or steric effects that did not originate from the diene and the dienophile themselves. We also became interested in the effects of the substitution pattern of the reactants on stereoselectivity and focused on these structural features instead of optimizing yields or determining the best conditions necessary for cycloaddition. The results of the synthetic evaluations are described herein as well as the computational studies that further shed light on the intricate details of the cycloaddition process.

## Results and Discussion

### Synthesis experiments

The Diels–Alder reactions of dienes **7** and **8** were carried out with a series of acyclic dienophiles bearing electron-withdrawing groups and with different substitution patterns at the Cα and Cβ positions (Table 1). We opted for variations that involved either methyl or phenyl group at the Cβ position in *trans* orientation with respect to the electron-withdrawing group or a methyl group at the Cα position to examine the influence of the reactant substituents on the *exo* and *endo* selectivity. The thermal [4 + 2] cycloadditions were performed in a sealed tube at 120–180 °C under neat conditions or in the presence of xylene (at 140 °C) or toluene (at 120 °C, in some nitroolefin cases) as solvent (see the Supplementary Methods for the detailed conditions). The *tert*-butyldimethylsilyl moiety remained intact throughout the process allowing the enol ether state to be preserved and characterized, albeit with double bond migration in some cases as mentioned below. The *exo* and *endo* assignments of the cycloadducts were based on the results of extensive NMR experiments (see Supplementary Methods) and X-ray crystallographic analysis in the cases of **exo-31**, **endo-62**, **exo-64** and **exo-67** (Supplementary Fig. 3). We similarly checked the possibility of *endo*-to-*exo* isomerization, this time with **endo-52** as representative compound, but no such transformation was again observed (Supplementary Fig. 2). Aside from the usual adducts, cycloaddition with diene **7** also gave, in some instances, products resulting from the migration of the double bond toward the adjacent, more substituted position. A similar phenomenon was also observed by Nakashima and Yamamoto in Brønsted acid-catalyzed Diels–Alder addition of 1,4-dimethyl silyloxydiene with ethyl vinyl ketone^35^. Because we are interested in observing the ultimate *exo* and *endo* selectivities, the quantities of these migration products were combined with the values of their parent *exo* or *endo* adducts.

The presence of functionalities at the C1 and C4 positions of the diene and the variations in the dienophiles provided some interesting general trends. For cycloadditions with diene **7**, excellent *exo* selectivities were observed with Cβ-methylated dienophiles (i.e., compounds **18**, **27**, **39**, **51** and **63**) in all the compound classes tested. Replacement of the Cβ-methyl with phenyl group still furnished the *exo*-cycloadducts as major products albeit in moderate-to-good stereoselectivity. The attributes brought about by these functional groups, thus, were able to considerably prevail over the factors, mainly secondary orbital interaction, often invoked to favour the *endo* pathway^8^. Absence of a substituent at Cβ resulted in either stereorandomness or a plunge into the “normal” *endo* territory. The same observation remained true even when a methyl group is positioned at Cα. Comparing the cycloaddition outcomes involving the related Cβ-unsubstituted carbonyl-containing dienophiles, the α,β-unsaturated aldehydes displayed the best tendency to form the *endo*-adduct, followed by the corresponding methyl ketones, and with the methyl ester losing much of the *endo* selectivity. A methyl group at the Cα position appeared to further increase the *endo* selectivity in the aldehyde (with dienophile **24**) and ketone (with dienophile **36**) cases. The stereorandom outcomes seen for the Cβ-unsubstituted nitrile and ester classes as opposed to that of aldehydes and ketones are suggestive of the roles of the electron-rich oxygen (from the methoxy group) and nitrogen atoms in the transition state preference of the reactants. For cycloadditions with nitroolefins, an early study by Node and co-workers^10^ noted that *exo* selectivity was a feature of Diels–Alder cycloadditions of 1-methoxy-3-trimethylsilyloxy-1,3-butadiene (Danishefsky’s diene) with Cβ-substituted nitroolefins. This *exo* preference was linked to the destabilization of the *endo* transition state as a result of the electrostatic repulsion between the silyloxy group of the diene and the nitro group of the dienophile. In our case, cycloadditions with nitroolefins behaved similarly as with other dienophile classes, and moderate inclination toward *endo* was observed when Cβ is unsubstituted.

Substitution, particularly with a methyl group, at C1 of the diene was found necessary for *exo* selectivity in Lewis acid-catalyzed cycloadditions with Cβ-methylated dienophiles^25^. However, despite the lack of substitution at the C1 position of diene **8**, our experiments showed that the *exo*-isomers remained the dominant products in Diels–Alder cycloadditions with Cβ-substituted olefins. It was noted in such cases that, compared to cycloadditions with diene **7**, the magnitude of the differences in the *exo* preference between dienophiles with methyl and phenyl substitution at the Cβ position became less evident. To illustrate, note the differences in the ratio of the ester *exo*/*endo*-adducts between **52** (>20/1) and **55** (2.6/1) both of which were derived from **7** as opposed to **53** (2.7/1) and **56** (1.6/1) both of which were derived from **8**. The absence of an interfering functionality at C1 of diene **8**, which may provide more steric repulsion to the three-dimensional methyl than to the flat phenyl group at Cβ, was the probable reason for the reduced selectivity difference. Interestingly, diene **8** provided a modest increase in *exo* preference in the cycloaddition with the Cβ-unsubstituted dienophiles as compared to diene **7**. This result is likely due to the subtle steric influence of the wider C4-phenyl group on the electron-withdrawing group in the *endo* pathway. In these cases, only methacrolein (**24**) supplied meaningful *endo* selectivity. Moreover, the best *exo*-selective outcomes were noted from dienophiles holding nitrile and nitro groups—the most electronegative electron-withdrawing groups in this series.

Further replacement of the C4-phenyl in **8** with methyl group and cycloaddition with the same set of α,β-unsaturated ketones (Supplementary Table 1) provided a blend of selectivities characteristic of both dienes **7** and **8** (i.e., less pronounced difference in *exo* selectivity between the Cβ-substituted dienophiles **39** and **42** and substantial *endo* selectivity with Cα-methylated dienophile **36**). These comparisons suggest steric strain between the C4-methyl group of the diene and the Cα-methyl group of the dienophile, destabilizing the *exo* transition state—a destabilization that was not as extensive as when a phenyl group was present at C4.

### Transition State Computations

Density functional theory calculations were performed to examine the *exo* and *endo* reaction pathways using B3LYP^36^,^37^ and M06-2X^38^ hybrid functionals with 6-311++G(d,p)^39^,^40^ basis sets. The electronic activation barriers (Δ*E*_a_^‡^) and the free energies of activation (Δ*G*_a_^‡^) for the *endo* and *exo* pathways involving diene **7** are presented in Supplementary Table 2 and Supplementary Table 3, respectively, and those for diene **8** are presented in Table 2 and Supplementary Table 4, respectively. The correlations between the experimental observations and the respective theoretically calculated Δ*E*_a_^‡^ and Δ*G*_a_^‡^ are shown in Supplementary Fig. 4 and Supplementary Fig. 5. Our M06-2X calculations generally produced lower activation energies than B3LYP, but in many of the studied cases, the B3LYP and M06-2X functionals were consistent and gave similar trends. For both functionals, about 70% to 80% of the calculated trends (*exo* vs *endo* selectivity) agreed qualitatively with the experiments and fall within the shaded region in Supplementary Fig. 4 and Supplementary Fig. 5. In particular, both functionals reproduced, with only a few exceptions, the experimental observations that cycloaddition reactions yield high *exo* selectivity if the Cβ position is substituted, especially with a methyl group (i.e., using dienophiles **18**, **27**, **39** and **51**). Notable outliers were the calculation results for nitroolefins, which often failed to mimic the selectivity trend in the experiments. On the other hand, calculations corresponding to dienophiles with no substituent at the Cβ positions produced the *endo*-isomer as major products, except in the nitrile and ester cases wherein the *exo* pathways were largely favoured. Overall, our computations suggest, as with the experimental observations, that better *exo* selectivity can be achieved with α,β-unsaturated nitriles and esters and less so with the corresponding aldehydes and ketones.

Previous studies^41^,^42^,^43^ suggested that the M06-2X functional gives better free energy values for concerted cycloadditions due to sensible treatment of medium-range correlation effects, such as van der Waals interactions^38^. For some Cβ-phenyl substituted dienophiles (e.g., **30** and **42**), the B3LYP functional predicted *endo* selectivities upon cycloaddition with diene **8**, but M06-2X calculations showed *exo* selectivities, matching those observed experimentally for such cases (Table 2). Nevertheless, we also noted that, compared with experimental observations, the M06-2X functional overstabilized the *exo* with respect to the *endo* pathway when the Cβ position is substituted with a phenyl group. With lower mean absolute error for both Δ*E*_a_^‡^ and Δ*G*_a_^‡^, the B3LYP results appeared to correlate better with experiments over M06-2X for this set of reactions (Supplementary Fig. 4 and Supplementary Fig. 5).

To rationalize the origin of the unusually high *exo* selectivity found in the C1-unsubstituted diene **8** and to further examine the difference between the activation energies of the *endo* and *exo* pathways, we decomposed the Δ*E*_a_^‡^ listed in Table 2 (and also Supplementary Table 2) into their component distortion energies (Δ*E*_d_^‡^) and interaction energies (Δ*E*_i_^‡^) following previous literature^25^,^44^,^45^. Δ*E*_d_^‡^ is the difference between the energies of the reactants in the optimized geometries and the same molecules in the transition state conformations but without interactions in between them. Δ*E*_i_^‡^, on the other hand, is the difference between the energies of the reactants in the transition state conformations summed separately and the energy of the entire transition state complex. Δ*E*_a_^‡^ is then the sum of Δ*E*_d_^‡^ and Δ*E*_i_^‡^.

Table 3 shows the activation energy decompositions for the [4 + 2] cycloadditions of dienes **7** and **8** with carbonyl-containing dienophiles functionalized with a methyl group at the Cβ position. In general, the M06-2X optimizations led to structures in closer proximity, hence a much larger interaction between the diene and the dienophile fragments in the transition state and a lower total Δ*E*_a_^‡^ than those calculated by B3LYP. The *endo* pathways involving diene **7** have larger stabilizing Δ*E*_i_^‡^ over *exo* as predicted by both B3LYP and M06-2X functionals. Nevertheless, the generally smaller Δ*E*_d_^‡^ in the *exo* pathway (1–3 kcal/mol less than that in the *endo* pathway) dominated and determined the final product selectivity. Conversely, we observed a slightly larger stabilizing Δ*E*_i_^‡^ in the *exo* than in the *endo* pathway in the B3LYP calculations for diene **8**. The overall distortion was also diminished when compared to that of diene **7**, which may be chiefly attributed to the lack of substitution at the C1 position of **8**. Moreover, the stabilization from Δ*E*_i_^‡^ for cycloadditions with diene **8** were about 4–5 kcal/mol lower than the values with diene **7** as predicted by both functionals, regardless of pathway. The B3LYP computations suggested a largely diminished Δ*E*_i_^‡^ stabilizing the *endo* pathway when the 1,4-dimethyl-substituted **7** was replaced by the C4-phenyl-substituted **8**. For reactions involving diene **8**, decompositions with M06-2X produced a much larger interaction between the diene and the dienophile fragments in the transition state, giving rise to lower total Δ*E*_a_^‡^. The total Δ*E*_d_^‡^, however, remained roughly the same regardless of the choice of functionals. The M06-2X results for **8** predicted stronger stabilizing Δ*E*_i_^‡^ in the *endo* pathway (by around 1–1.6 kcal/mol), consistent with the traditional picture for secondary orbital interaction^46^. The overall *exo* selectivity, nevertheless, came from the smaller Δ*E*_d_^‡^ in the *exo* pathway. Hence, the two functionals predicted slightly different underlying mechanisms for the *exo* selectivity of diene **8**—interaction energy-driven versus distortion energy-driven. We imagined the observed *exo* selectivity as a possible combination of both factors, that is, the usual governing *endo* pathway lost its dominance due to stronger transition state distortion and/or weaker stabilization occurred in the *endo* transition state due to poor structural alignment.

We then analyzed the critical geometric parameters of the transition states involving diene **8** and the α,β-unsaturated nitriles and esters, the two most *exo*-selective dienophile classes calculated by B3LYP (Table 4, also see Supplementary Table 5). The corresponding parameters derived from cycloadditions with diene **7** are listed in Supplementary Table 6. Between the two incipient carbon–carbon bonds, Table 4 shows a much shorter *d*_1−β_ (bond distance between C1 of diene and Cβ of dienophile, about 1.9 Å) than *d*_4−α_ (bond distance between C4 of diene and Cα of dienophile, about 2.9 Å), indicating the typical asynchronous behavior in most asymmetric Diels–Alder reactions^47^. In particular, we observed that *d*_1−β_ was usually slightly longer in *endo* than in *exo* transition states, implying larger steric hindrance within the proximity of C1 and Cβ in the *endo* pathway, despite the lack of substitution at C1. Curiously, while *d*_1−β_ remained essentially the same across the range of dienophiles examined, *d*_4−α_ was notably shorter for Cβ-substituted (2.75–2.86 Å) than Cβ-unsubstituted dienophiles (2.92–2.98 Å). Turning to cycloadditions with diene **7**, the *d*_4−α_ values were even shorter and more so for cycloadditions with Cβ-substituted dienophiles (2.38–2.62 Å). These observations suggested that the steric repulsion experienced by the Cβ (or C1) functionality prompted the shortening of *d*_4−α_ (a seesaw-like effect). We suspect that the short *d*_4−α_ in the pathways with diene **7** consequently translated into the high Δ*E*_i_^‡^ indicated in Table 3. Table 4 also lists the deviations of the dienophiles from planarity in the transition state geometry. The *endo* pathways showed much larger deviations from planarity than the *exo* counterparts by about 6° or more, further offering support for the larger distortion in the *endo* transition state.

**Figure 3:**
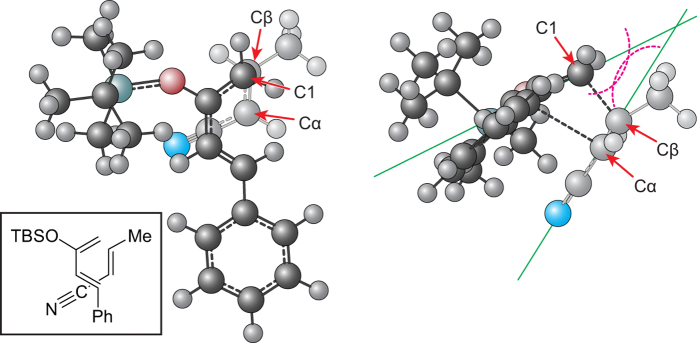
*Endo* transition state structures for cycloaddition of 8 and 18 optimized by B3LYP showing the diene–dienophile overlay (left) and the tilt of the dienophile with respect to the plane of the diene (right, approximated by green lines). The inset structure is included for clarity. Diene **8** is shown in a darker shade than the dienophiles **18**.

In accordance with the twist asynchronous model^48^, we also analyzed the twisting given by the C4-C1–Cβ-Cα dihedral angle as a means of balance between stress alleviation and interaction of the reacting partners (Table 4). The twisting angles in the *exo* pathway showed tendencies to push the electron-withdrawing group outward, away from the C4-phenyl group of diene **8**. The *endo* pathway, on the other hand, held the electron-withdrawing group under the repulsive influence of the silyloxy and C4-phenyl groups, minimizing the twisting. The repulsion of the Cα-methyl by the C4-phenyl group in the pathways involving the dienophiles **15** and **48** also strongly countered the phenyl repulsion of the nitrile and ester groups. This is evidenced, for example, by the smaller outward twist for the *exo* (−5°) than the *endo* pathway (−11°) with the Cα-methylated **15**. In contrast, the *exo* transition state involving the unmethylated dienophile **12** supplied a twist angle of −15°. The dienophile was also tilted at an angle in reference to the plane of the diene with the nearest point at the C1–Cβ junction (Fig. 3, also see Supplementary Fig. 6). This, in turn, brought the Cβ-substituent under the greater influence of the attached moieties at C1. Such pressure on the Cβ-substituent was not evident in the *exo* pathway and even though some degree of outward twisting would bring the Cβ-substituent somewhat in closer proximity to the silyloxy group, the experiments and calculations showed that the preference for the *exo* pathway was usually maintained.

**Figure 4:**
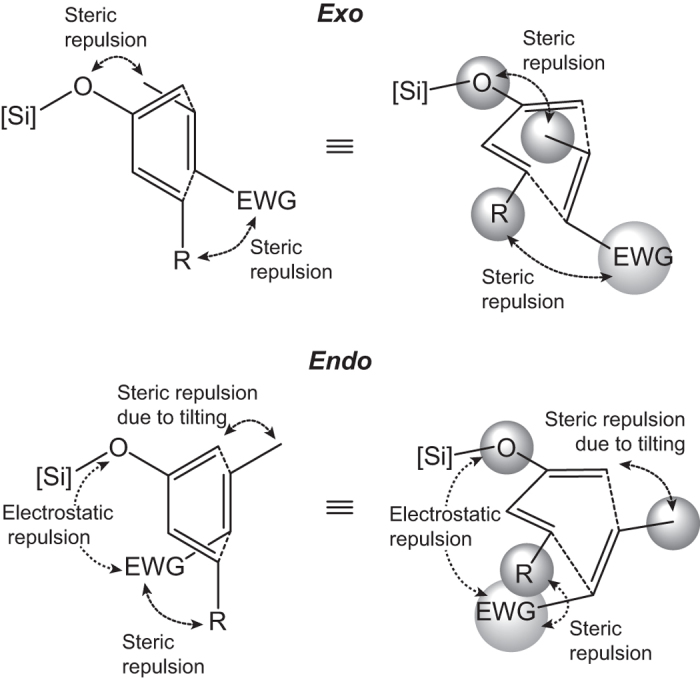
Proposed major rivalling interaction forces in the two pathways of the examined normal-electron demand Diels–Alder reactions.

Based on the acquired data, an underlying mechanism may be proposed for the unconventional *exo* selectivity in this series of Diels–Alder reactions. The stereoselectivity is derived from the interplay between different interaction forces in two pathways. Specifically, these include (i) the steric repulsion between the Cβ-substituent of the dienophile and the silyloxy group and C1-substituent (if any) of the diene in the *exo* pathway, (ii) the steric repulsion of groups at C1 and the Cβ-substituent as a consequence of dienophile tilting in the *endo* pathway and (iii) the steric repulsion between the electron-withdrawing group and Cα-substituent (if any) of the dienophile and the C4-substituent of the diene (Fig. 4). Twisting along the shorter forming bond in the transition state relieves such repulsive forces, likely more readily for the *exo* pathway. The effect of the strong steric repulsion found in the *endo* transition states may be two-fold. On one hand, it raises the Δ*E*_d_^‡^ of the *endo* pathway to the extent that possible stabilization from secondary orbital interactions might be overwhelmed. On the other hand, the secondary orbital interaction itself may be reduced when close alignment is not geometrically favored. In addition, the *endo* transition state may also involve extra electrostatic repulsion between the electron-withdrawing group of the dienophiles and the silyloxy group of the dienes^10^,^25^. This repulsion is amplified by the presence of appropriately positioned electron-rich atoms in the electron-withdrawing group, such as in the nitrile, ester, and even nitro functionalities. Given the experimentally and computationally observed *exo* selectivity trend relating to the electron-withdrawing group, the degree of destabilization of the *endo* transition states can be proportionately explained by the combination of the size and electronegativity of such electron-withdrawing group.

## Conclusions

We have noted and systematically investigated a number of *exo*-selective thermal normal-electron-demand Diels–Alder reactions involving 2-silyloxydienes substituted at the terminal positions and simple dienophiles with a range of substitution patterns. These variations in the reacting partners fleshed out the salient factors that permitted such unconventional *exo* selectivity. Structural features such as substitution at Cβ, but not at the Cα position of the dienophile and the presence of nitrile and ester electron-withdrawing groups favour the *exo* adduct. Such observations are the likely result of the stress on the Cβ-substituent and the electrostatic and steric repulsions experienced by the corresponding electron-withdrawing group in the *endo* transition state. The larger distortion experienced in the *endo* pathway as a consequence of repulsive forces is consistent with the diminished preference for the *endo* product, even overriding the stabilizing interaction forces in many of the studied cases. Overall, the present work brings important insights into the intricate *exo*/*endo* selectivity mechanics of the Diels–Alder cycloadditions. The findings in this report may help guide chemists in their pursuit of wielding this celebrated and very useful reaction.

## Methods

### Chemical synthesis

The complete experimental details and compound characterization can be found in the Supplementary Methods. For the NMR spectra of the compounds in this article, see Supplementary Figs 7–270.

### Computation

Density functional theory calculations were carried out with *Gaussian09*^49^ using the B3LYP and M06-2X hybrid functionals with the 6-311++G(d,p) basis set. Transition state optimizations were performed using the Synchronous Transit-Guided Quasi-Newton (STQN) method^50^,^51^. Harmonic vibrational frequencies were computed for all optimized structures to ensure that they are either potential energy surface minima (all real frequencies) or transition states (one imaginary frequency). Zero-point energies were included in all thermodynamic quantities at 298 K. Product distributions at room temperature were calculated using the Arrhenius rate expression derived from the standard transition state theory^52^. Solvation corrections for the solvent used in most experiments (xylene, *o*-, *m*-, *p*-mixture, ε = 2.3879) were calculated using the polarizable continuum model (PCM) method^53^ with default universal force field (UFF) radii.

### Data availability

The X-ray crystallographic coordinates for compounds **exo-10**, **exo-11**, **exo-31**, **endo-62**, **exo-64** and **exo-67** in this study have been deposited at the Cambridge Crystallographic Data Centre (CCDC), under deposition numbers CCDC 1450387, CCDC 1450388, CCDC 1450389, CCDC 1450392, CCDC 1450390 and CCDC 1450391, respectively. This data can be obtained free of charge from the CCDC via www.ccdc.cam.ac.uk/data_request/cif.

## Additional Information

**How to cite this article**: Ho, G.-M. *et al*. Unconventional *exo* selectivity in thermal normal-electron-demand Diels–Alder reactions. *Sci. Rep.* **6**, 35147; doi: 10.1038/srep35147 (2016).

## Supplementary Material

Supplementary Information

## Figures and Tables

**Figure 1:**
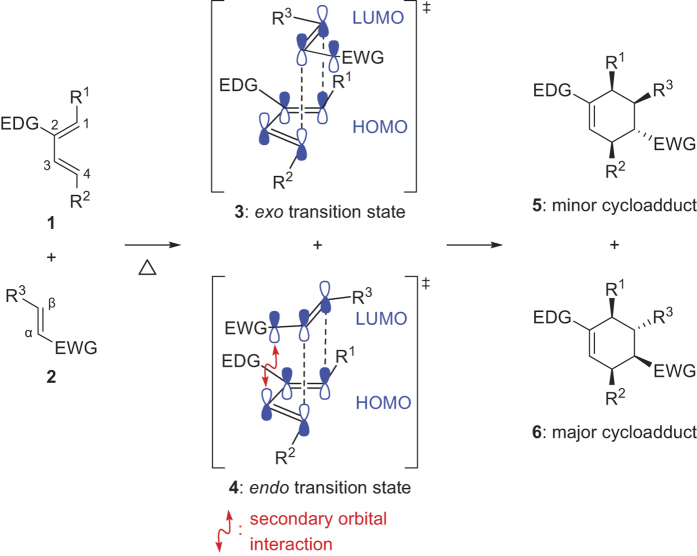
General stereoselective prediction for normal-electron-demand Diels–Alder reactions. EWG = electron-withdrawing group, EDG = electron-donating group, HOMO = highest occupied molecular orbital, LUMO = lowest unoccupied molecular orbital.

**Figure 2:**
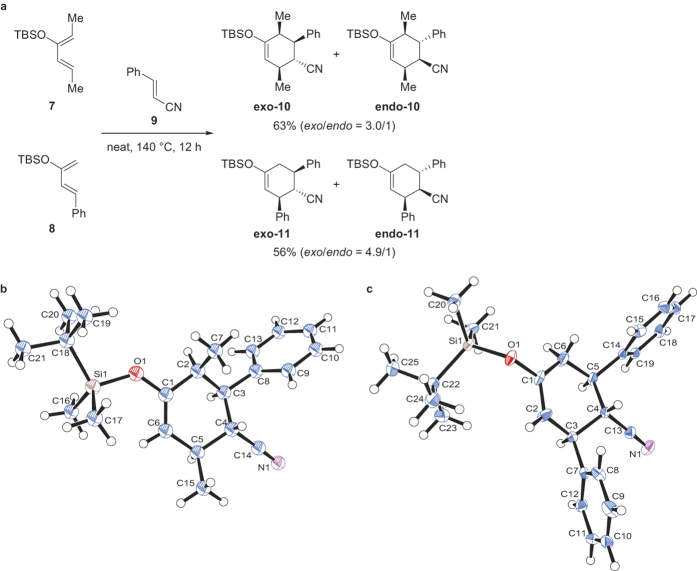
Diels–Alder reactions of dienes 7 and 8 with cinnamonitrile (9). (**a**) The reaction conditions and outcomes. (**b**) X-ray crystal structure of **exo-10**. (**c**) X-ray crystal structure of **exo-11**. TBS = *tert*-butyldimethylsilyl.

**Table 1:** Thermal Diels–Alder reactions of dienes 7 and 8 with various dienophiles.
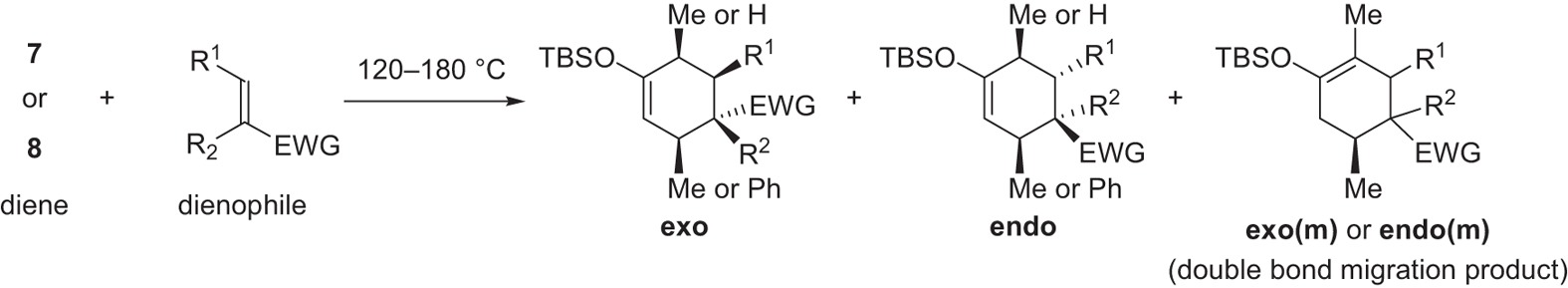

Dienophile	Cycloaddition with **7**			Cycloaddition with **8**		
	Product	Yield^a^	*exo*/*endo*^b^	Product	Yield^a^	*exo*/*endo*^b^
**12**: R^1^ = R^2^ = H, EWG = CN	**13**	60%	1/1	**14**	54%	3.3/1
**15**: R^1^ = H, R^2^ = Me, EWG = CN	**16**	65%	1.2/1	**17**	55%	1.3/1
**18**: R^1^ = Me, R^2^ = H, EWG = CN	**19**	57%	>20/1	**20**	55%	4.0/1
**21**: R^1^ = R^2^ = H, EWG = C(O)H	**22**	74%	1/2.1	**23**	63%	1/1
**24**: R^1^ = H, R^2^ = Me, EWG = C(O)H	**25**	96%	1/4.6	**26**	73%	1/2.7
**27**: R^1^ = Me, R^2^ = H, EWG = C(O)H	**28**	79%	>20/1^c^	**29**	42%	1.8/1
**30**: R^1^ = Ph, R^2^ = H, EWG = C(O)H	**31**	70%	3.0/1	**32**	45%	1.9/1
**33**: R^1^ = R^2^ = H, EWG = C(O)Me	**34**	64%	1/1.3^d^	**35**	99%	1/1.1
**36**: R^1^ = H, R^2^ = Me, EWG = C(O)Me	**37**	83%	1/2.8^e^	**38**	93%	1/1
**39**: R^1^ = Me, R^2^ = H, EWG = C(O)Me	**40**	89%	19/1^f^	**41**	71%	2.3/1
**42**: R^1^ = Ph, R^2^ = H, EWG = C(O)Me	**43**	92%	5.3/1	**44**	65%	1.9/1
**45**: R^1^ = R^2^ = H, EWG = CO_2_Me	**46**	73%	1.1/1	**47**	29%	1.3/1
**48**: R^1^ = H, R^2^ = Me, EWG = CO_2_Me	**49**	79%	1.3/1	**50**	44%	1.2/1
**51**: R^1^ = Me, R^2^ = H, EWG = CO_2_Me	**52**	79%	>20/1^g^	**53**	32%	2.7/1
**54**: R^1^ = Ph, R^2^ = H, EWG = CO_2_Me	**55**	89%	2.6/1^h^	**56**	63%	1.6/1
**57**: R^1^ = R^2^ = H, EWG = NO_2_	**58**	66%	1/2.8	**59**	62%	2.3/1
**60**: R^1^ = H, R^2^ = Me, EWG = NO_2_	**61**	67%	1/1.9	**62**	75%	1.8/1
**63**: R^1^ = Me, R^2^ = H, EWG = NO_2_	**64**	64%	19/1	**65**	60%	4.3/1
**66**: R^1^ = Ph, R^2^ = H, EWG = NO_2_	**67**	76%	3.8/1	**68**	80%	5.7/1

^a^Based on the total isolated amounts of the isomers.

^b^Determined from the ^1^H NMR spectra of the crude product mixture with the amount for the double bond migration isomer included in the corresponding *exo* or *endo* values.

^c^**exo(m)-28** is 17% of *exo* selectivity.

^d^**endo(m)-34** is 68% of *endo* selectivity.

^e^**endo(m)-37** is 4% of *endo* selectivity.

^f^**exo(m)-40** is 4% of *exo* selectivity.

^g^**exo(m)-52** is 33% of *exo* selectivity.

^h^**endo(m)-55** is 21% of *endo* selectivity.

**Table 2:** Calculated activation energy barriers (Δ*E*_a_^‡^) and product ratios of the Diels–Alder reaction pathways involving diene 8 by B3LYP and M06-2X functionals^a^.

Dienophile: substituent	ΔE_a_^‡^ (kcal/mol)		k_*exo*_/k_*endo*_^b^
	*Exo*	*Endo*	
**12**: EWG = CN	16.9 (13.4)	17.8 (12.9)	2.8/1 (1/1.9)
**15**: α-Me, EWG = CN	20.3 (14.2)	20.8 (13.7)	1.8/1 (1/1.9)
**18**: β-Me, EWG = CN	21.8 (15.5)	23.0 (16.2)	4.2/1 (2.3/1)
**11**: β-Ph, EWG = CN	23.9 (12.0)	24.5 (14.9)	1.9/1 (33/1)
**21**: EWG = C(O)H	16.9 (13.8)	16.9 (12.6)	1/1 (1/4.5)
**24**: α-Me, EWG = C(O)H	19.4 (13.8)	18.5 (12.7)	1/3.3 (1/4.2)
**27**: β-Me, EWG = C(O)H	20.8 (15.3)	21.2 (15.4)	1.6/1 (1.2/1)
**30**: β-Ph, EWG = C(O)H)	24.7 (14.1)	24.6 (16.2)	1/1.2 (13/1)
**33**: EWG = C(O)Me	19.1 (14.5)	19.1 (12.8)	1/1 (1/7.6)
**36**: α-Me, EWG = C(O)Me	21.4 (13.7)	20.4 (12.8)	1/3.5 (1/3.3)
**39**: β-Me, EWG = C(O)Me	24.1 (16.5)	24.3 (16.2)	1.4/1 (1/1.4)
**42**: β-Ph, EWG = C(O)Me	26.5 (14.4)	26.2 (15.7)	1/1.3 (5.0/1)
**45**: EWG = CO_2_Me	18.7 (14.0)	19.2 (12.6)	2.0/1 (1/5.2)
**48**: α-Me, EWG = CO_2_Me	20.7 (13.7)	20.9 (13.4)	1.4/1 (1/1.5)
**51**: β-Me, EWG = CO_2_Me	21.4 (13.8)	22.8 (15.0)	5.6/1 (4.2/1)
**54**: β-Ph, EWG = CO_2_Me	25.2 (12.6)	26.0 (15.6)	2.9/1 (36/1)
**57**: EWG = NO_2_	10.6 (6.6)	9.9 (5.0)	1/2.5 (1/7.4)
**60**: α-Me, EWG = NO_2_	12.8 (7.5)	12.1 (5.4)	1/2.3 (1/13)
**63**: β-Me, EWG = NO_2_	14.8 (6.8)	14.7 (8.7)	1/1.5 (1/10)
**66**: β-Ph, EWG = NO_2_	16.7 (6.0)	16.0 (6.0)	1/2.2 (1/1)

^a^The results of the M06-2X calculations are given in parenthesis.

^b^Relative reaction rate constants estimated by the Arrhenius equation^52^.

**Table 3:** Decomposition of Δ*E*_a_^‡^ into distortion (Δ*E*_d_^‡^) and interaction energies (Δ*E*_i_^‡^) for the reactions between dienes 7 and 8 and the Cβ-methylated carbonyl-containing dienophiles calculated by B3LYP and M06-2X functionals^a^.

Diene	Dienophile	Pathway	Δ*E*_d_^‡^ (kcal/mol)			Δ*E*_i_^‡^ (kcal/mol)
			Diene	Dienophile	Total	
**7**	aldehyde **27**	*Exo*	15.8 (17.6)	14.5 (11.7)	30.3 (29.3)	−7.9 (−15.3)
		*Endo*	16.3 (17.8)	16.4 (13.1)	32.7 (30.9)	−8.9 (−15.3)
	ketone **39**	*Exo*	16.6 (17.8)	15.7 (12.7)	32.3 (30.5)	−7.0 (−14.3)
		*Endo*	17.1 (18.3)	17.7 (13.2)	34.8 (31.5)	−7.8 (−14.9)
	ester **51**	*Exo*	16.2 (16.5)	13.8 (10.5)	30.0 (27.0)	−6.9 (−14.3)
		*Endo*	17.1 (19.2)	15.7 (10.7)	32.8 (29.9)	−7.2 (−14.9)
**8**	aldehyde **27**	*Exo*	11.8 (15.2)	13.5 (10.3)	25.3 (25.5)	−4.5 (−10.3)
		*Endo*	11.4 (15.7)	13.9 (10.9)	25.3 (26.6)	−4.1 (−11.2)
	ketone **39**	*Exo*	12.6 (14.9)	15.0 (11.9)	27.6 (26.8)	−3.6 (−10.4)
		*Endo*	12.3 (16.4)	15.6 (11.8)	27.9 (28.2)	−3.6 (−12.0)
	ester **51**	*Exo*	12.4 (15.1)	13.2 (9.5)	25.6 (24.6)	−4.3 (−10.7)
		*Endo*	12.5 (16.2)	13.4 (10.6)	25.9 (26.8)	−3.1 (−11.7)

^a^The relevant Δ*E*_a_^‡^ are listed in Table 2 and Supplementary Table 2. The results of the M06-2X calculations are given in parenthesis.

**Table 4:** Critical geometrical parameters of the *exo* and *endo* transition states of Diels–Alder reactions involving diene 8 calculated at B3LYP/6-311++G(d,p) level.

Dienophile: substituent	Pathway	Forming bond length (Å)^a^		Deviation from planarity^b^	Twist angle^c^
		*d*_1−β_	*d*_4−α_		
**12** (EWG = CN)	*Exo*	1.96	2.95	19°	−15°
	*Endo*	1.98	2.92	25°	−5°
**15** (α-Me, EWG = CN)	*Exo*	1.96	2.95	22°	−5°
	*Endo*	1.98	2.93	28°	−11°
**18** (β-Me, EWG = CN)	*Exo*	1.95	2.86	22°	−19°
	*Endo*	1.97	2.86	28°	3°
**11** (β-Ph, EWG = CN)	*Exo*	1.94	2.80	23°	−20°
	*Endo*	1.96	2.75	31°	2°
**45** (EWG = CO_2_Me)	*Exo*	1.96	2.98	20°	−16°
	*Endo*	1.97	2.94	28°	−11°
**48** (α-Me, EWG = CO_2_Me)	*Exo*	1.97	2.96	22°	−5°
	*Endo*	1.97	2.98	29°	−16°
**51** (β-Me, EWG = CO_2_Me)	*Exo*	1.95	2.86	23°	−20°
	*Endo*	1.97	2.86	33°	−4°
**54** (β-Ph, EWG = CO_2_Me)	*Exo*	1.93	2.81	23°	−21°
	*Endo*	1.96	2.75	36°	−3°

^a^The subscripts represent the carbons involved in bond formation.

^b^Deviation from planarity of the dienophile spanning from the EWG to the *trans*-β-function.

^c^Deviation of the diene and dienophile from being parallel given by the C4-C1–Cβ-Cα dihedral angle with pivot point at the forming C1-Cβ bond; positive angles represent inward twist by the dienophile, negative angle is outward twist.
