# Supplementary material for: Unconventional exo selectivity in thermal normal-electron-demand Diels–Alder reactions
Source: Sci Rep. 2016 Oct 12;6:35147. doi: 10.1038/srep35147 (PMC5059706; doi:10.1038/srep35147)
Supplement: Supplementary Information [file srep35147-s1.pdf]

Supplementary Information

Unconventional *exo* selectivity in thermal normal-electron-demand Diels–Alder reactions

Guo-Ming Ho[1], Ci-Jhang Huang[2], Elise Yu-Tzu Li[2,*], Sheng-Kai Hsu[1], Ti Wu[1], Medel Manuel L. Zulueta[1], Kevin Binchia Wu[1] & Shang-Cheng Hung[1,*]

[1]Genomics Research Center, Academia Sinica, 128, Section 2, Academia Road, Taipei 115, Taiwan

[2]Department of Chemistry, National Taiwan Normal University, 88, Section 4, Ting-Chow Road, Taipei 116, Taiwan

*E-mail: schung@gate.sinica.edu.tw and eliseytli@ntnu.edu.tw

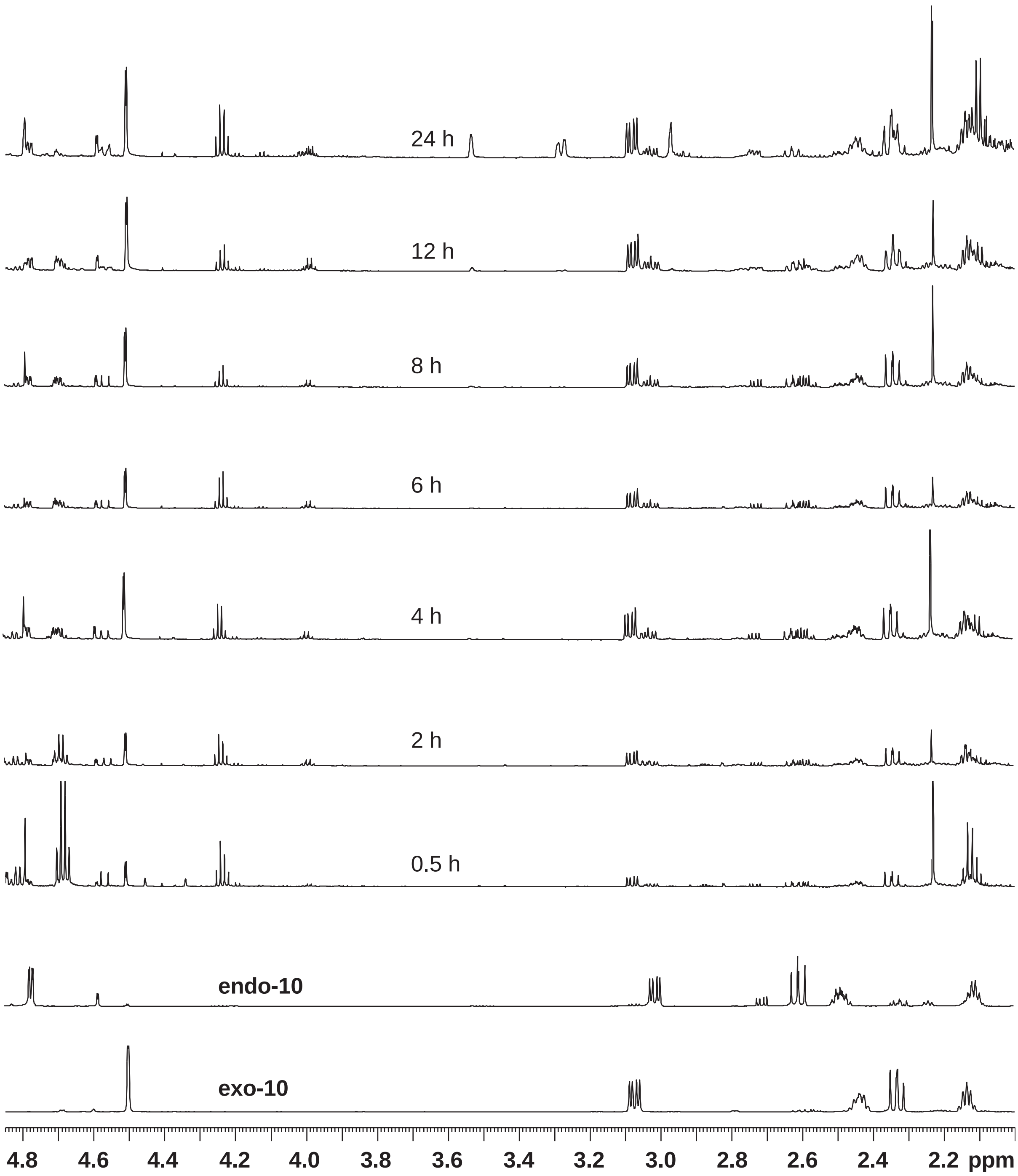

Supplementary Figure 1. ^{1}**H NMR monitoring of the thermal cycloaddition of diene 7 and dienophile 9 toward the *exo*/*endo*-adduct 10.**

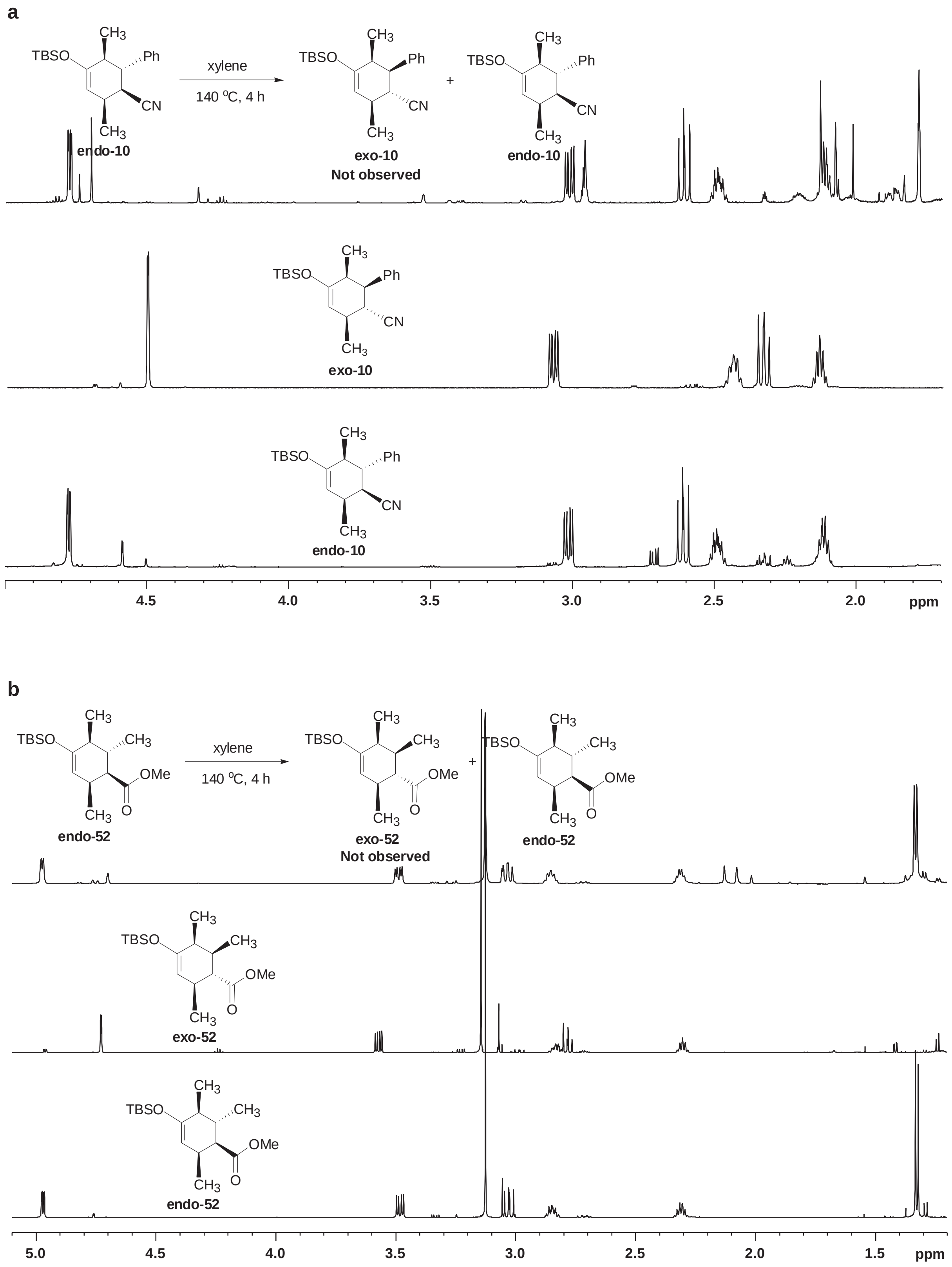

Supplementary Figure 2. ^{1}**H NMR spectra showing the lack of endo-to-exo isomerization after heating the *endo* isomer for 4 h with the spectra for the exo and endo isomers provided as references. a**, Evaluation of **endo-10**. **b**, Evaluation of **endo-52**.

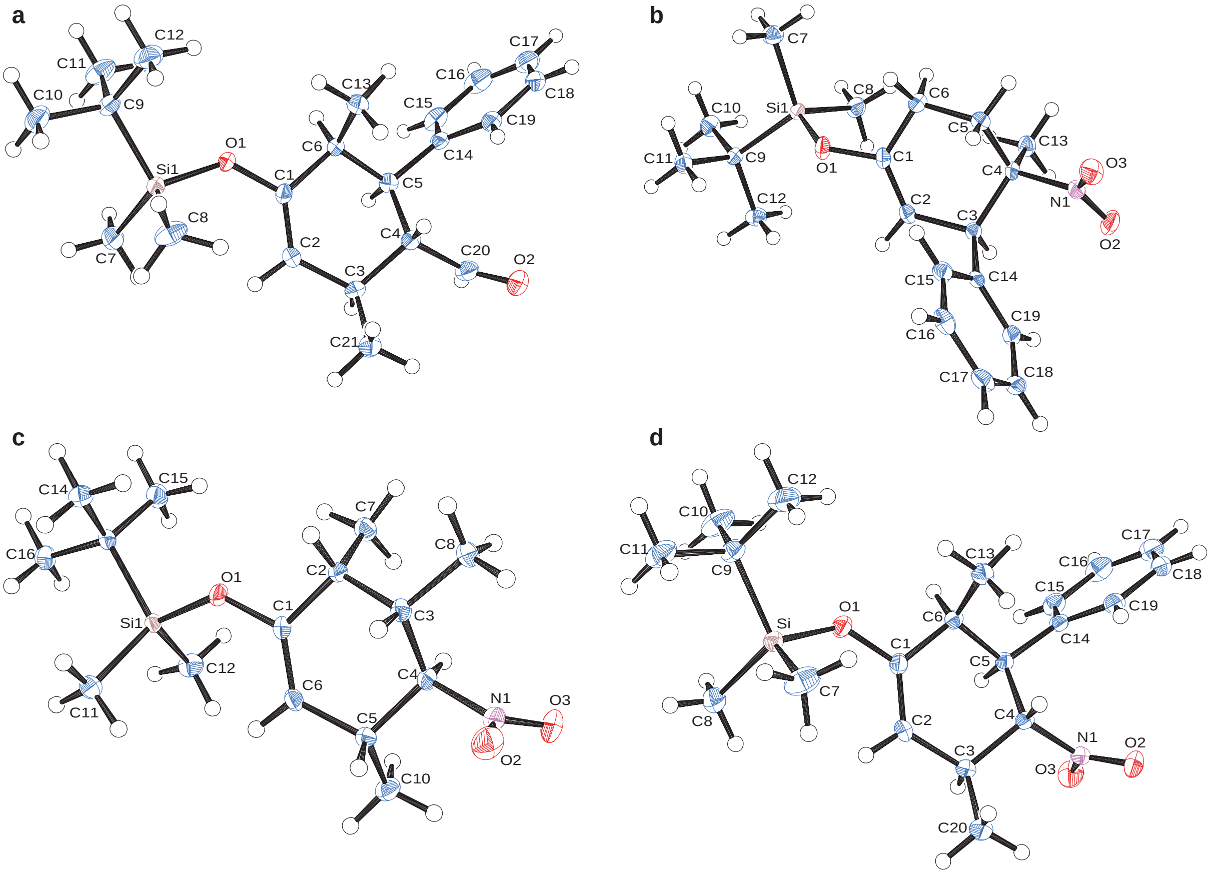

Supplementary Figure 3. X-ray crystal structures of some Diels–Alder cycloadducts. a, compound **exo-31**; **b**, compound **endo-62**; **c**, compound **exo-64**; **d**, compound **exo-67**.

a

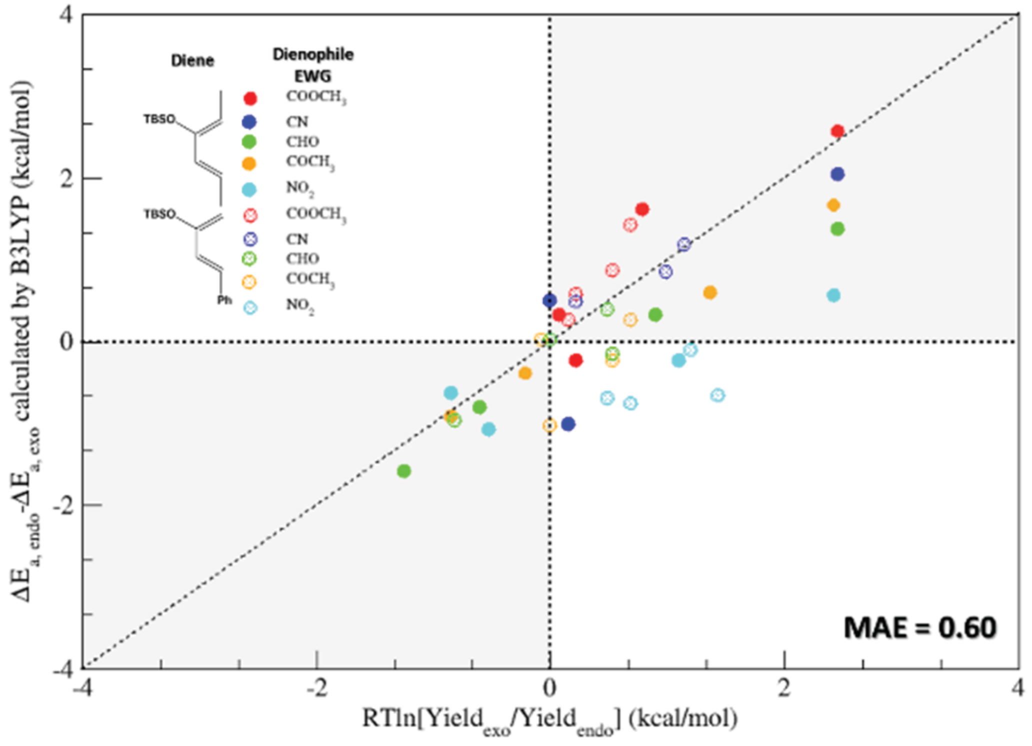

b

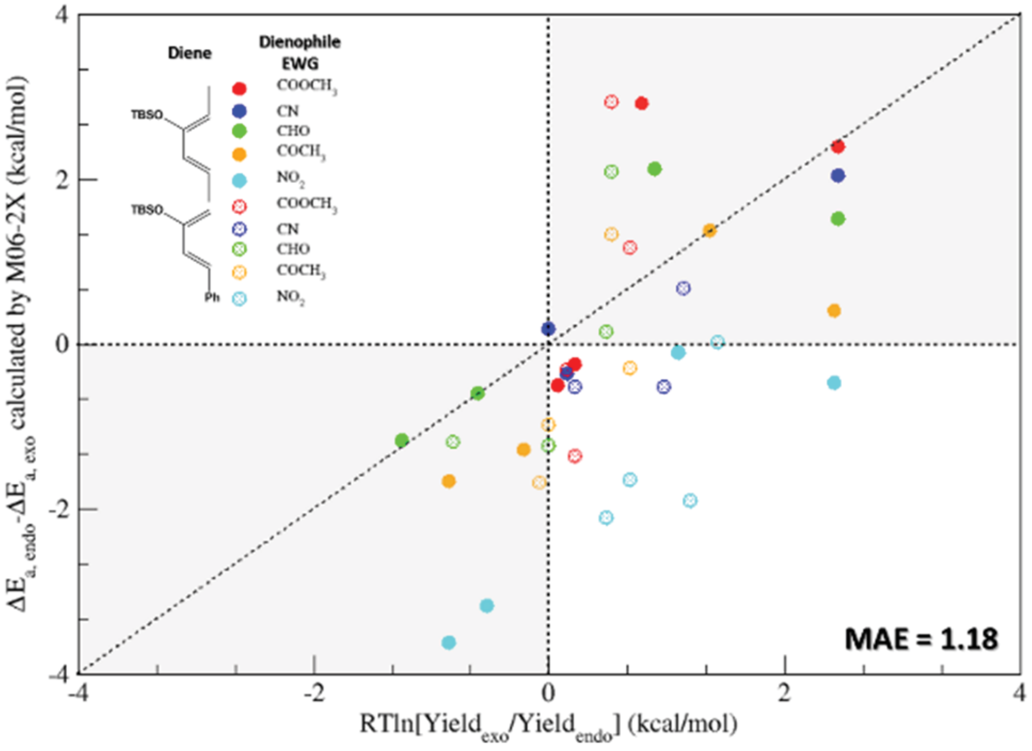

Supplementary Figure 4. Correlations of the difference in activation energy barrier ($\Delta E_a^{\ddagger}$) between the *endo* and *exo* reaction pathways calculated by (a) B3LYP and (b) M06-2X and the experimentally observed product ratio based on 38 different reactant combinations. MAE = mean absolute error.

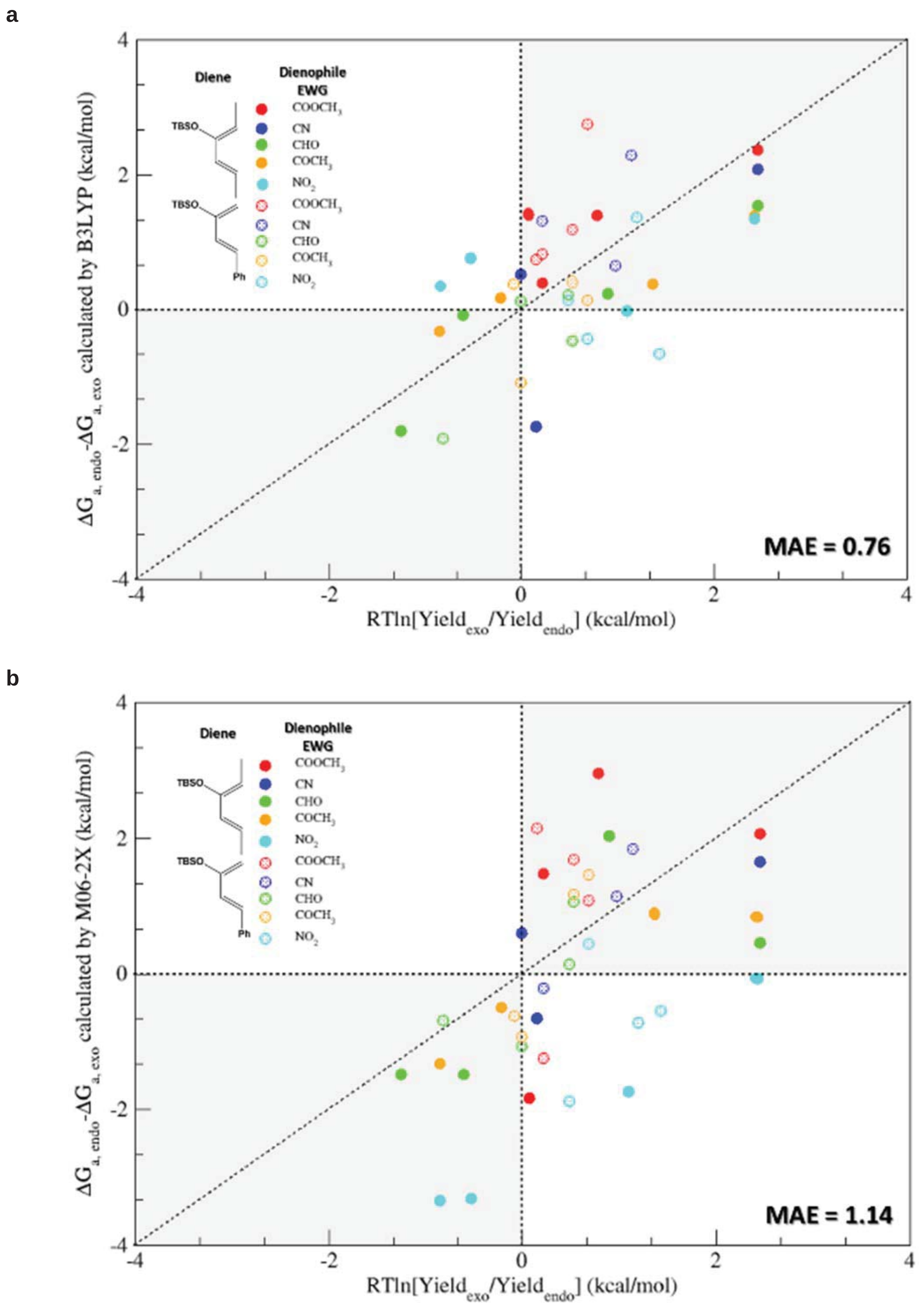

Supplementary Figure 5. Correlations of the difference in Gibbs free energy of activation ($\Delta G_a^{\ddagger}$) between the *endo* and *exo* reaction pathways calculated by (a) B3LYP and (b) M06-2X and the experimentally observed product ratio based on 38 different reactant combinations.

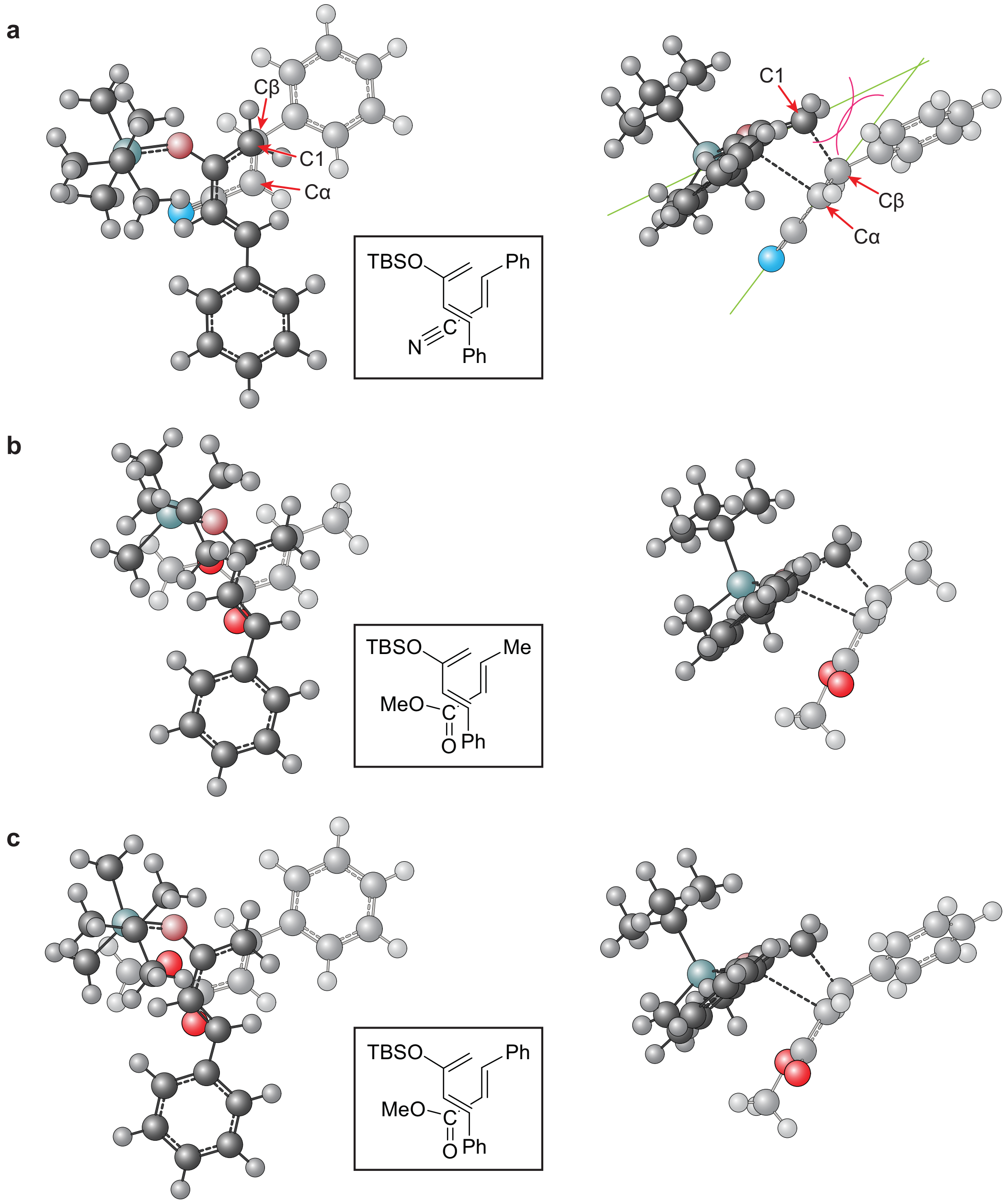

Supplementary Figure 6. Additional *endo*-transition state structures optimized by B3LYP showing the diene–dienophile overlay (left) and the tilt of the dienophile with respect to the plane of the diene (right, approximated by green lines in panel a). a, cycloaddition of **8** and **11**; **b**, cycloaddition of **8** and **51**; **c**, cycloaddition of **8** and **54**. The inset structures are included for clarity. The dienes are shown in a darker shade than the dienophiles.

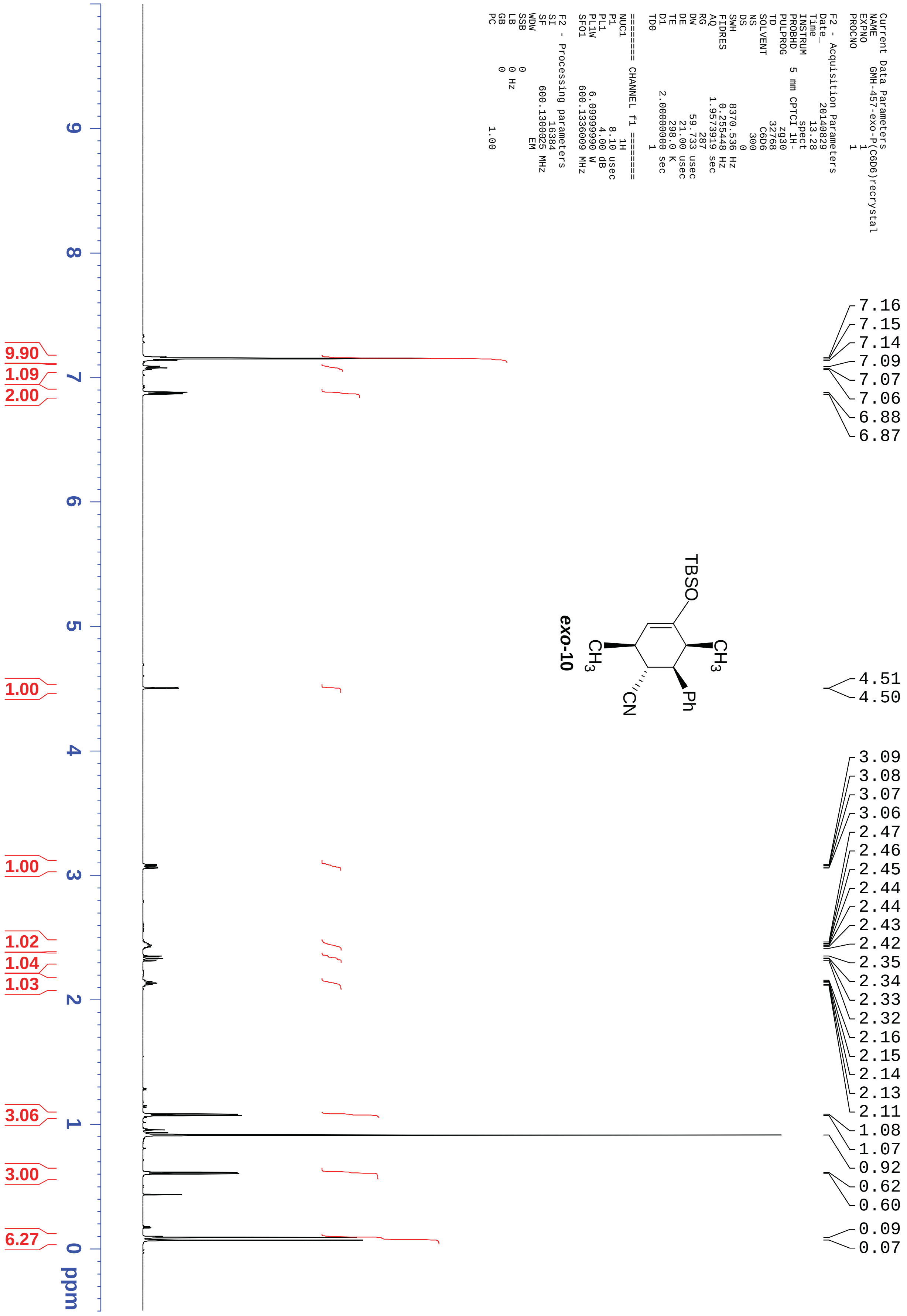

Supplementary Figure 7. ^{1}**H NMR spectrum of compound exo-10.**

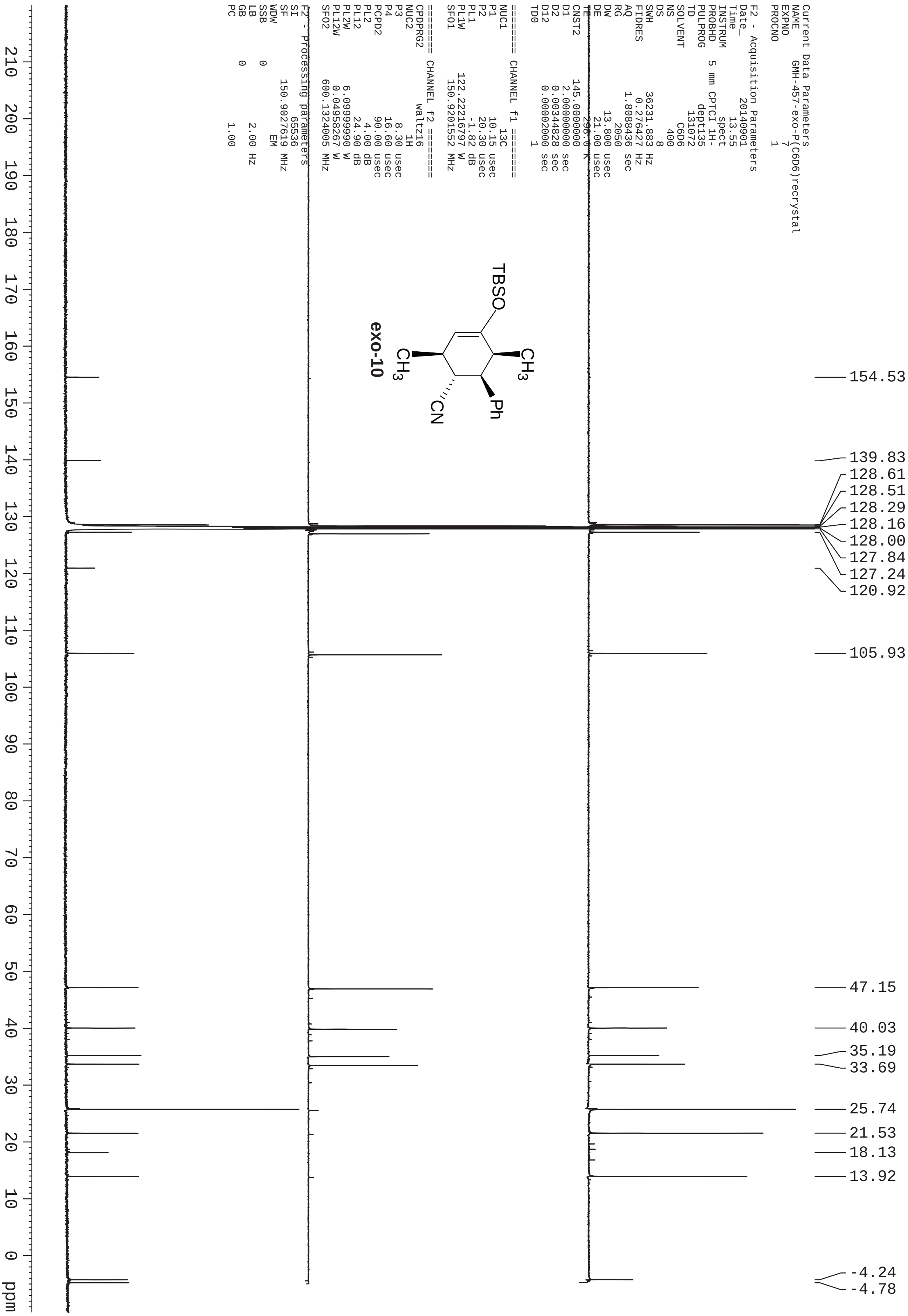

Supplementary Figure 8. ^{13}C and DEPT NMR spectra of compound exo-10.

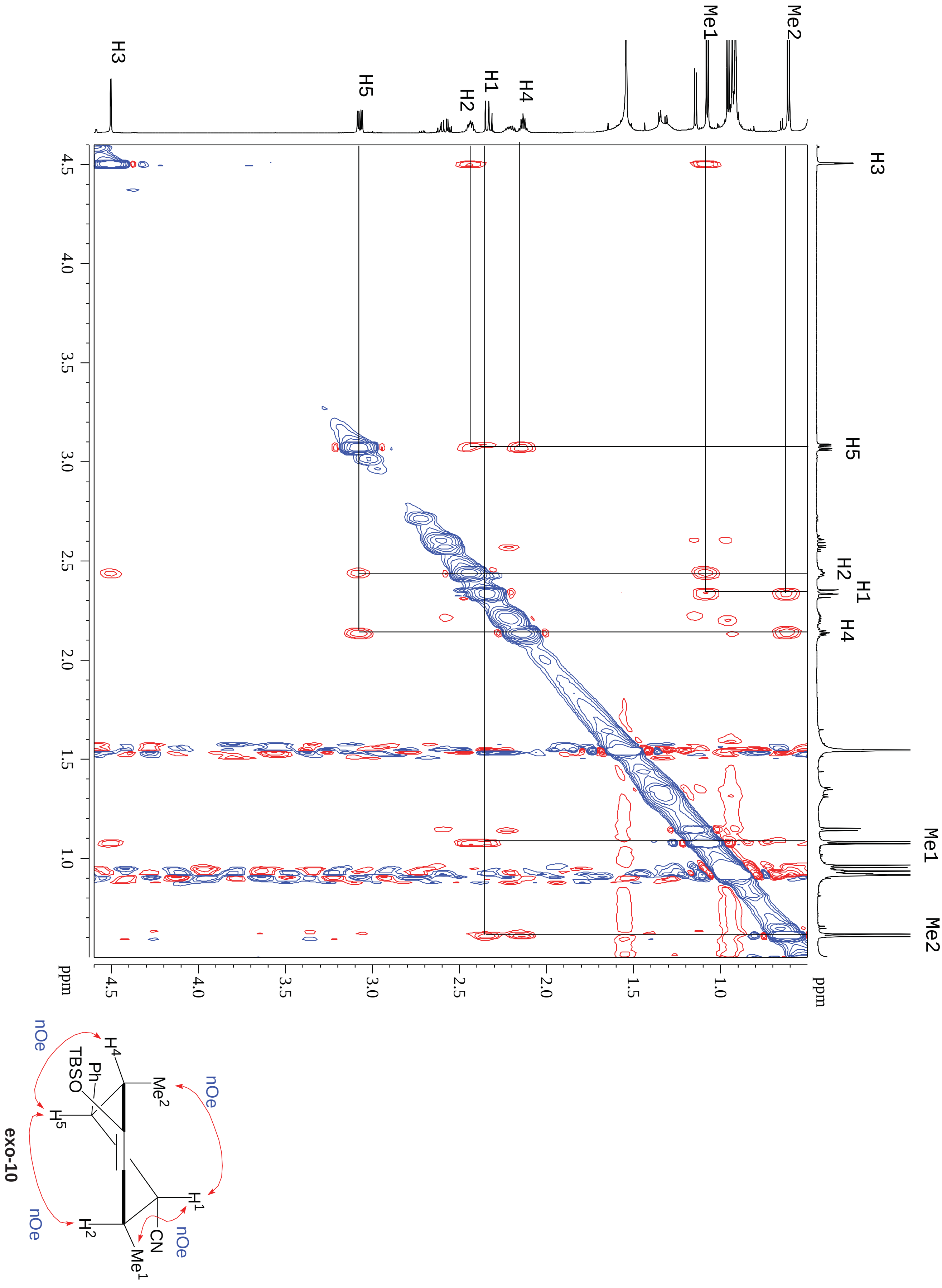

Supplementary Figure 9. NOESY NMR spectrum of compound exo-10.

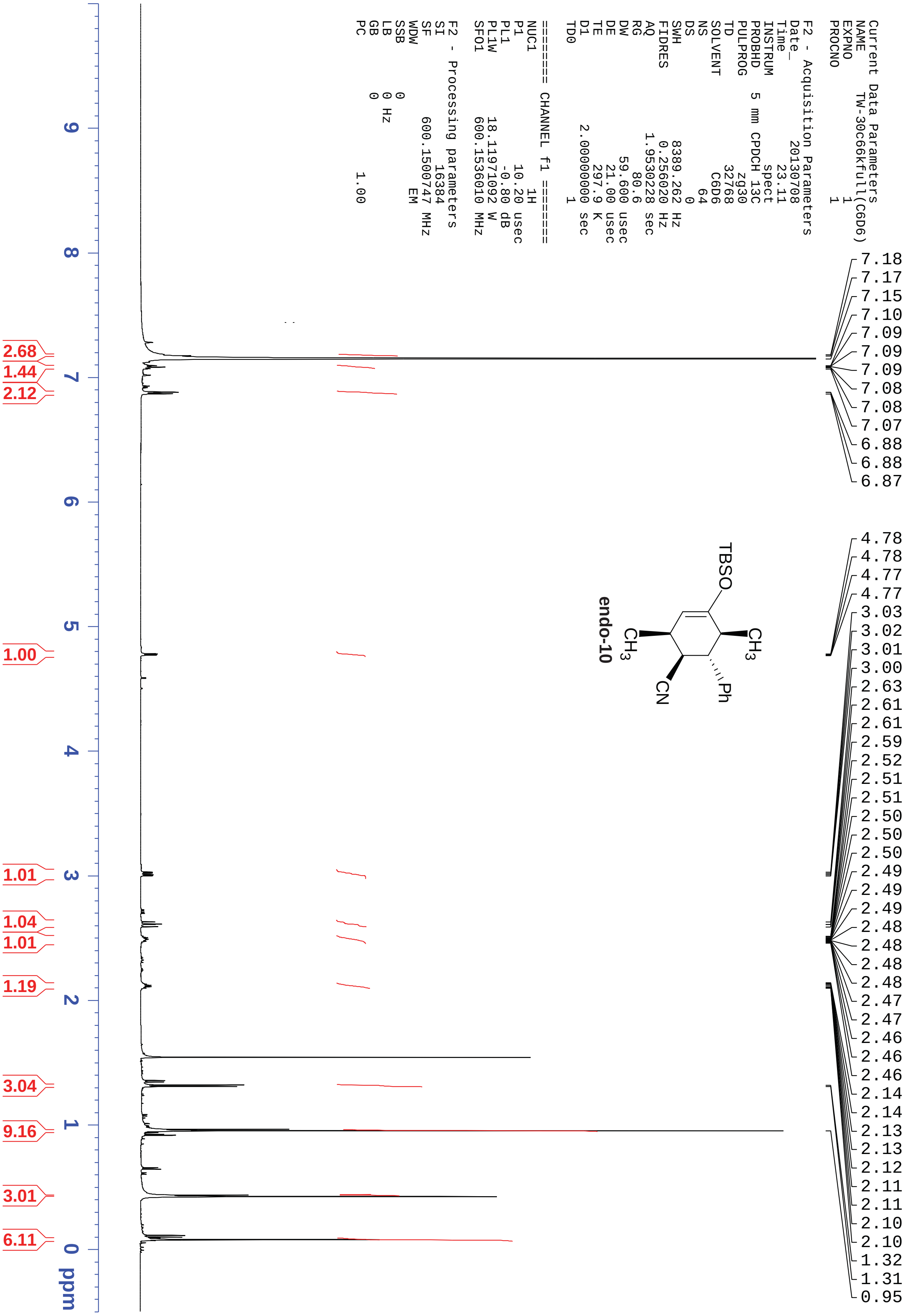

Supplementary Figure 10. **^{1}H NMR spectrum of compound endo-10.**

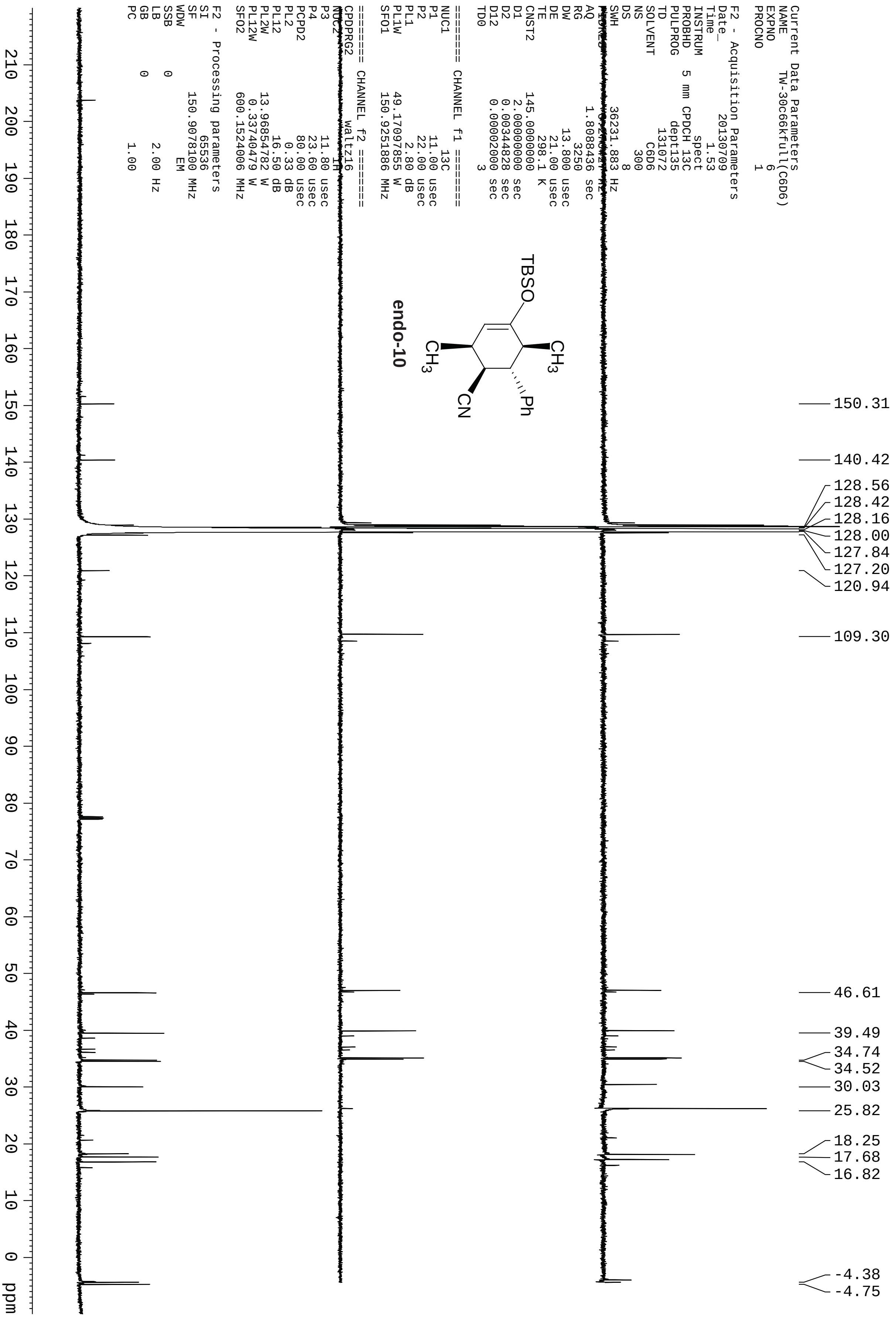

Supplementary Figure 11. ^{13}C and DEPT NMR spectra of compound endo-10.

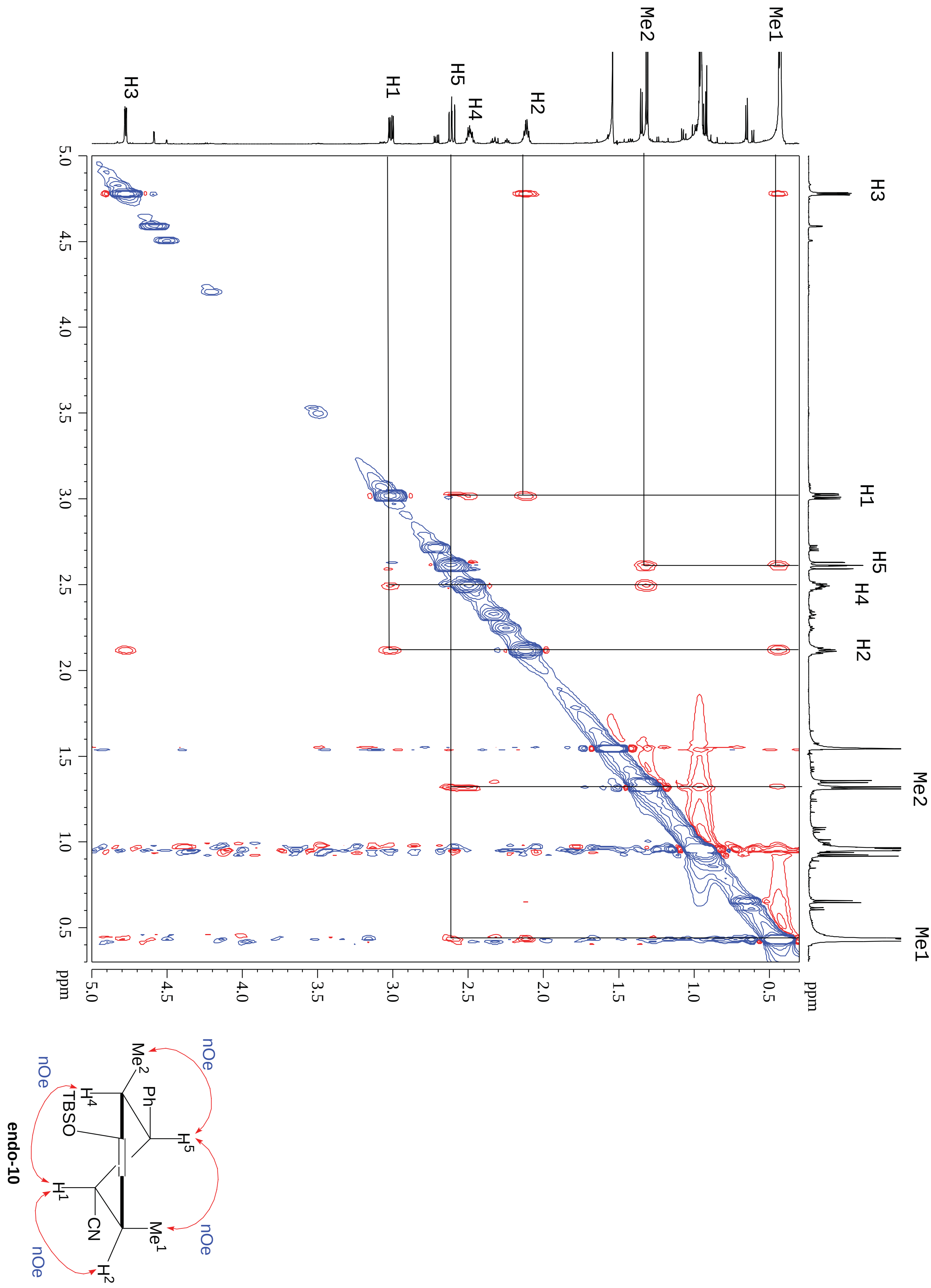

Supplementary Figure 12. NOESY NMR spectrum of compound endo-10.

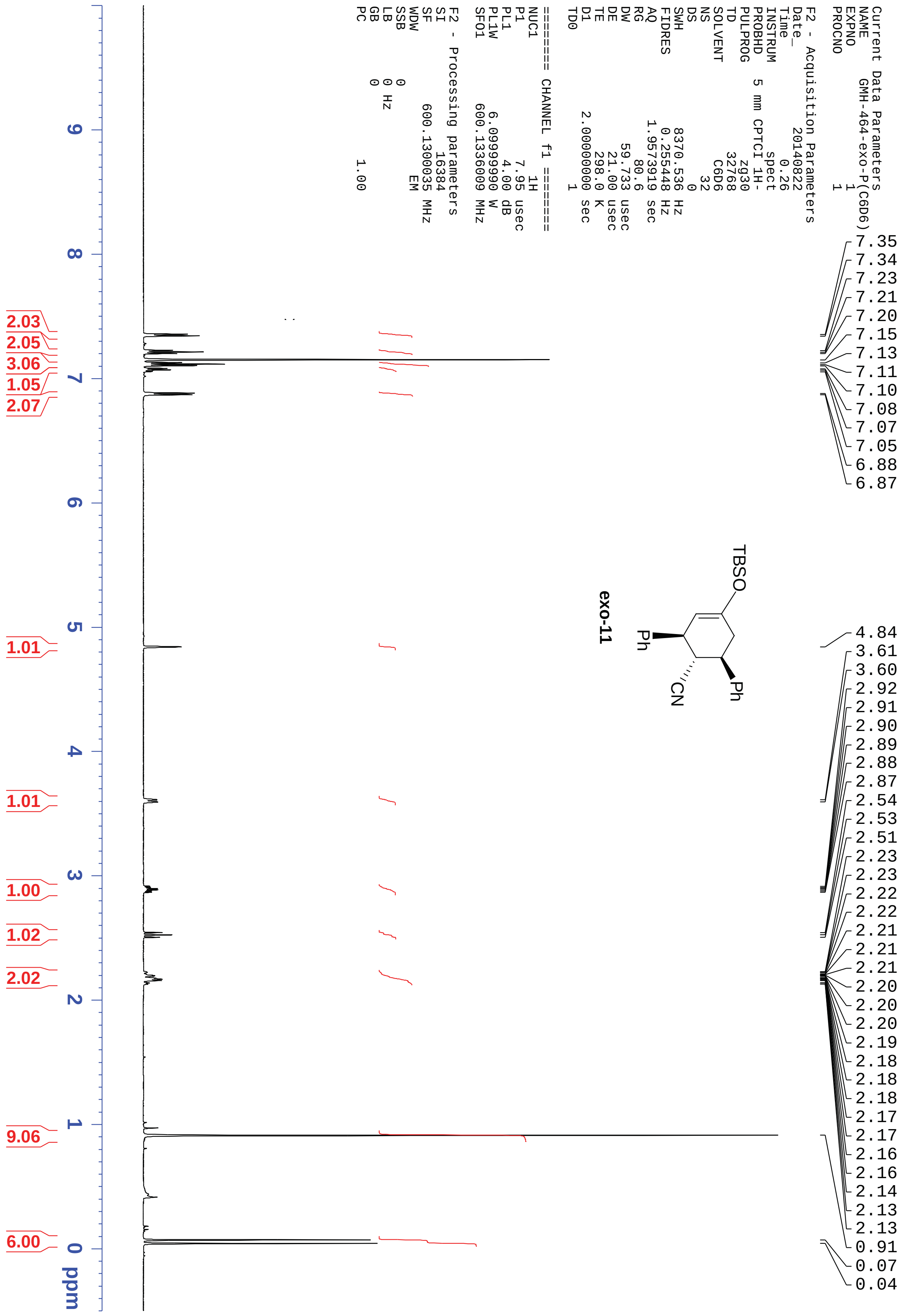

Supplementary Figure 13. **^{1}H NMR spectrum of compound exo-11.**

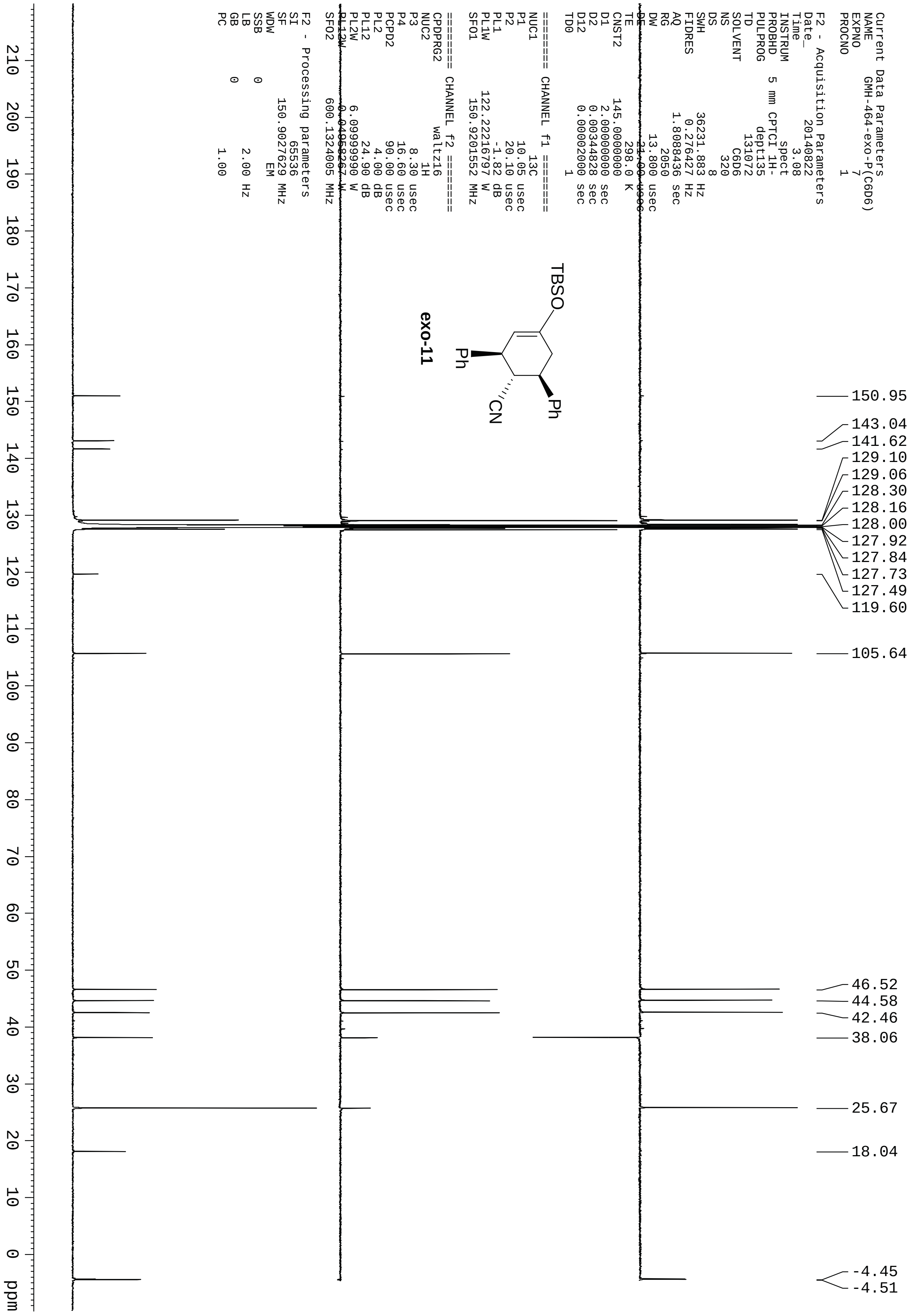

Supplementary Figure 14. ^{13}C NMR and DEPT spectra of compound exo-11.

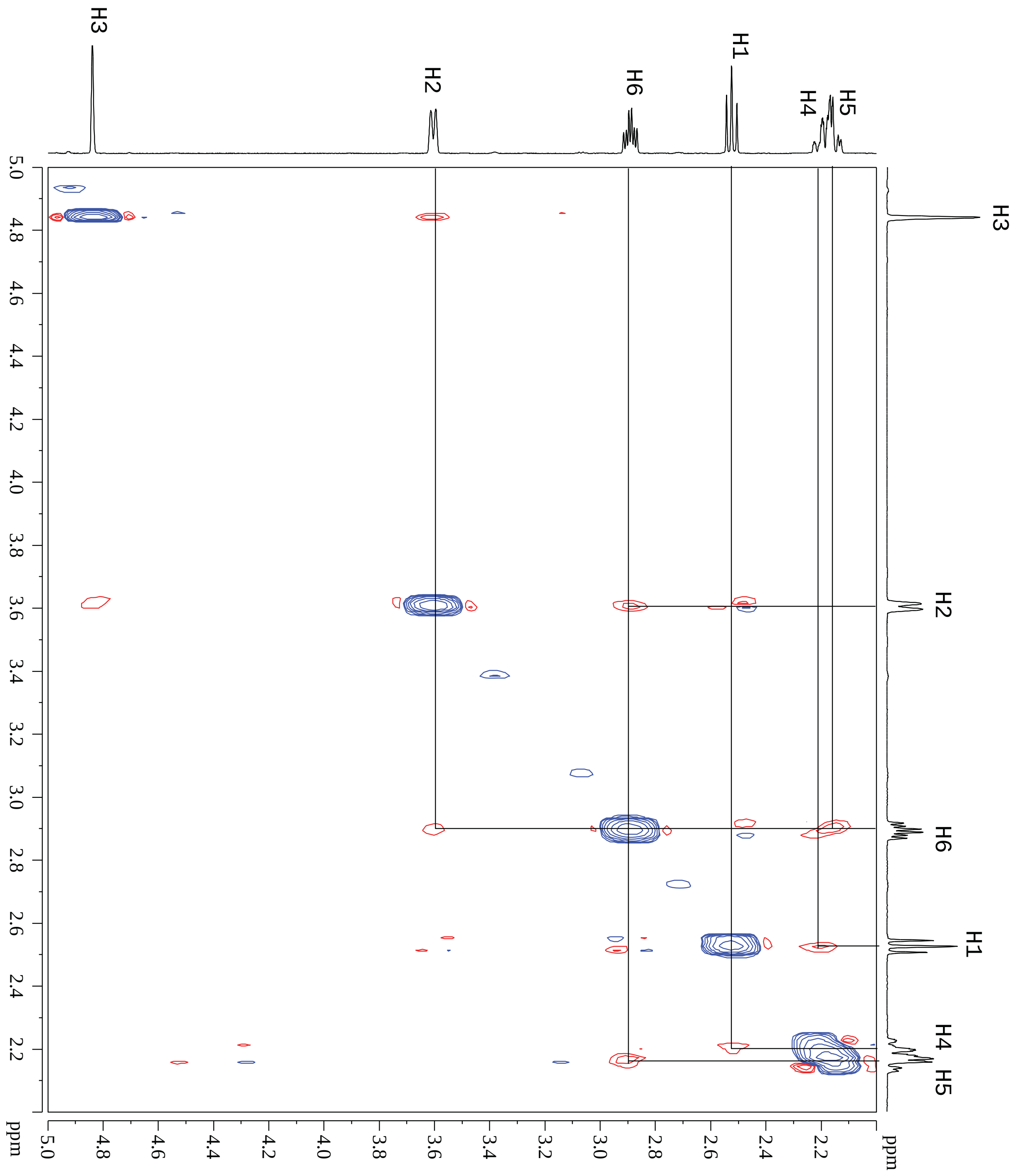

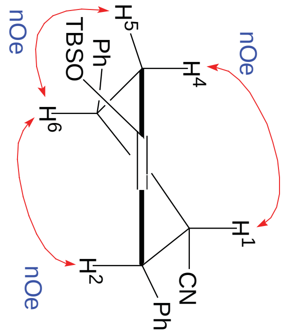

exo-11

Supplementary Figure 15. NOESY NMR spectrum of compound exo-11.

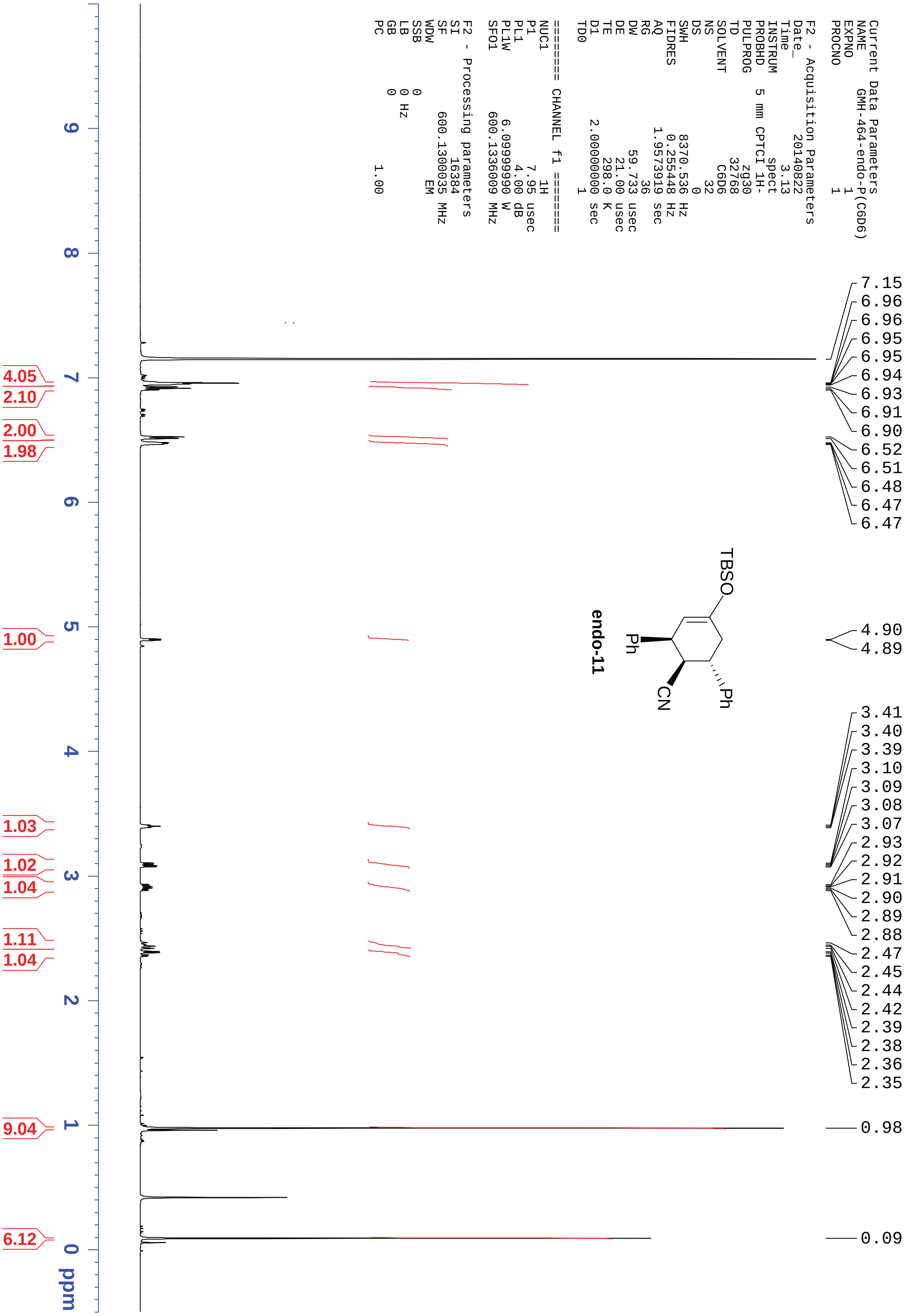

Supplementary Figure 16. ^{1}**H NMR spectrum of compound endo-11.**

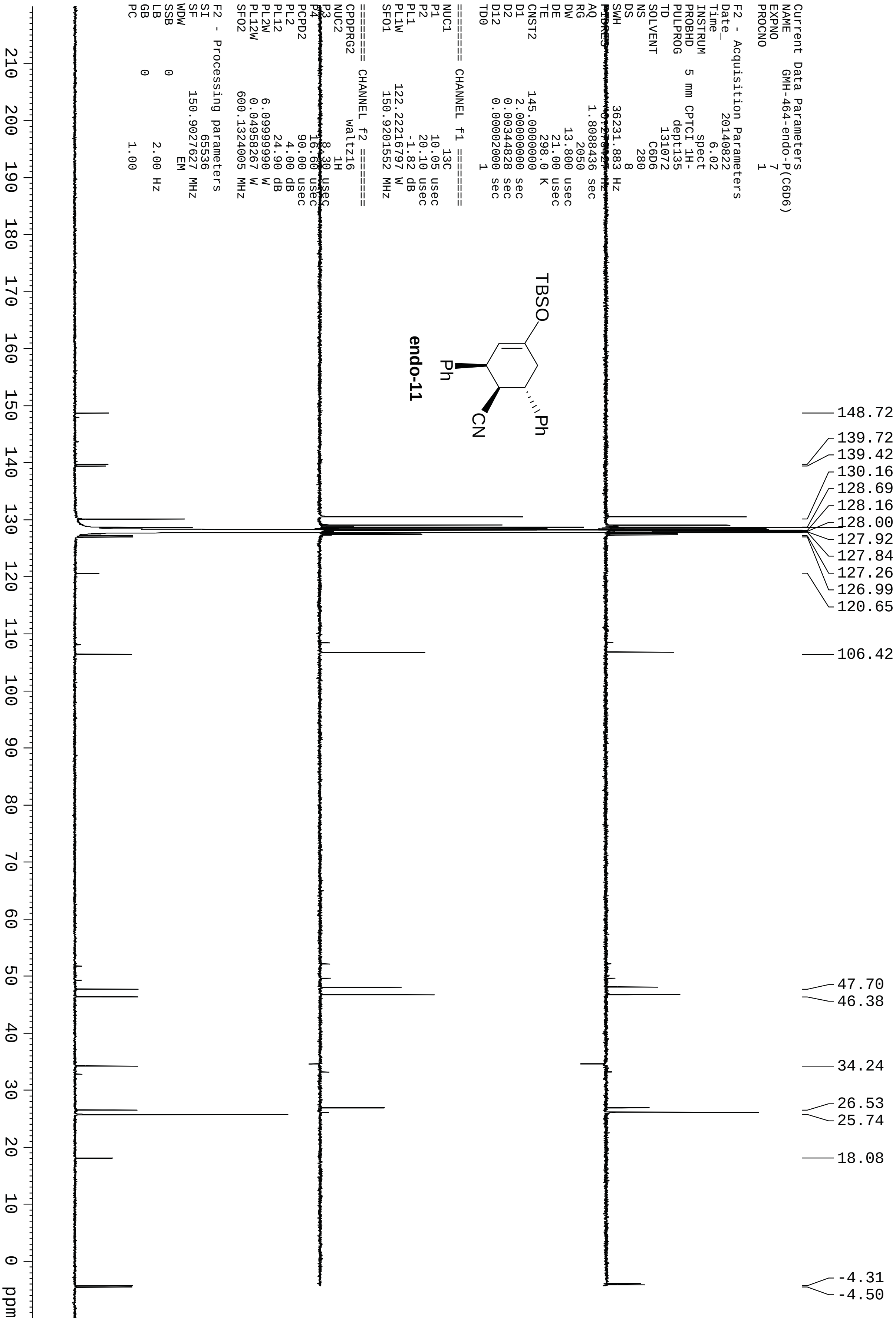

Supplementary Figure 17. ^{13}C and DEPT NMR spectra of compound endo-11.

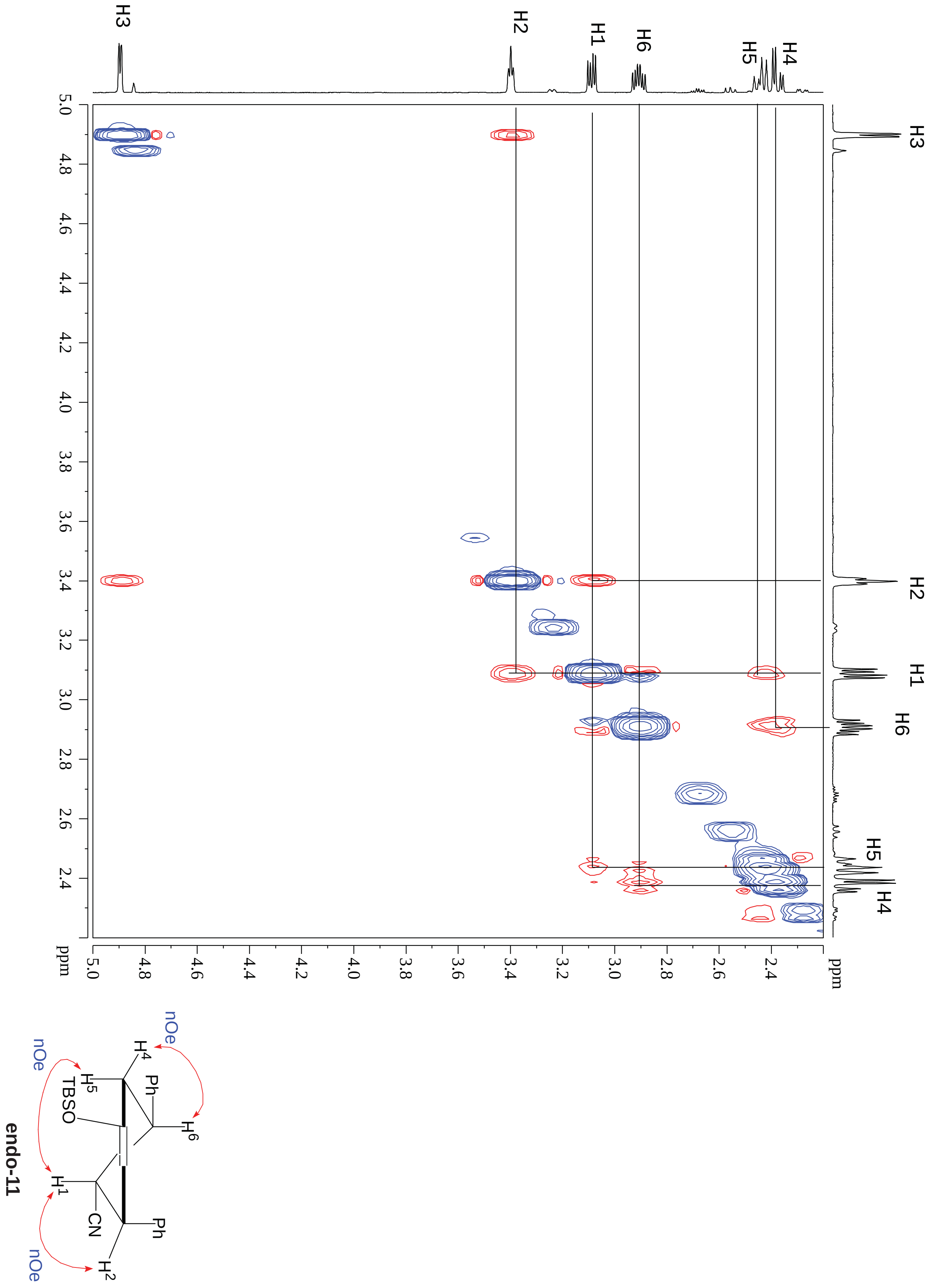

Supplementary Figure 18. NOESY NMR spectrum of compound endo-11.

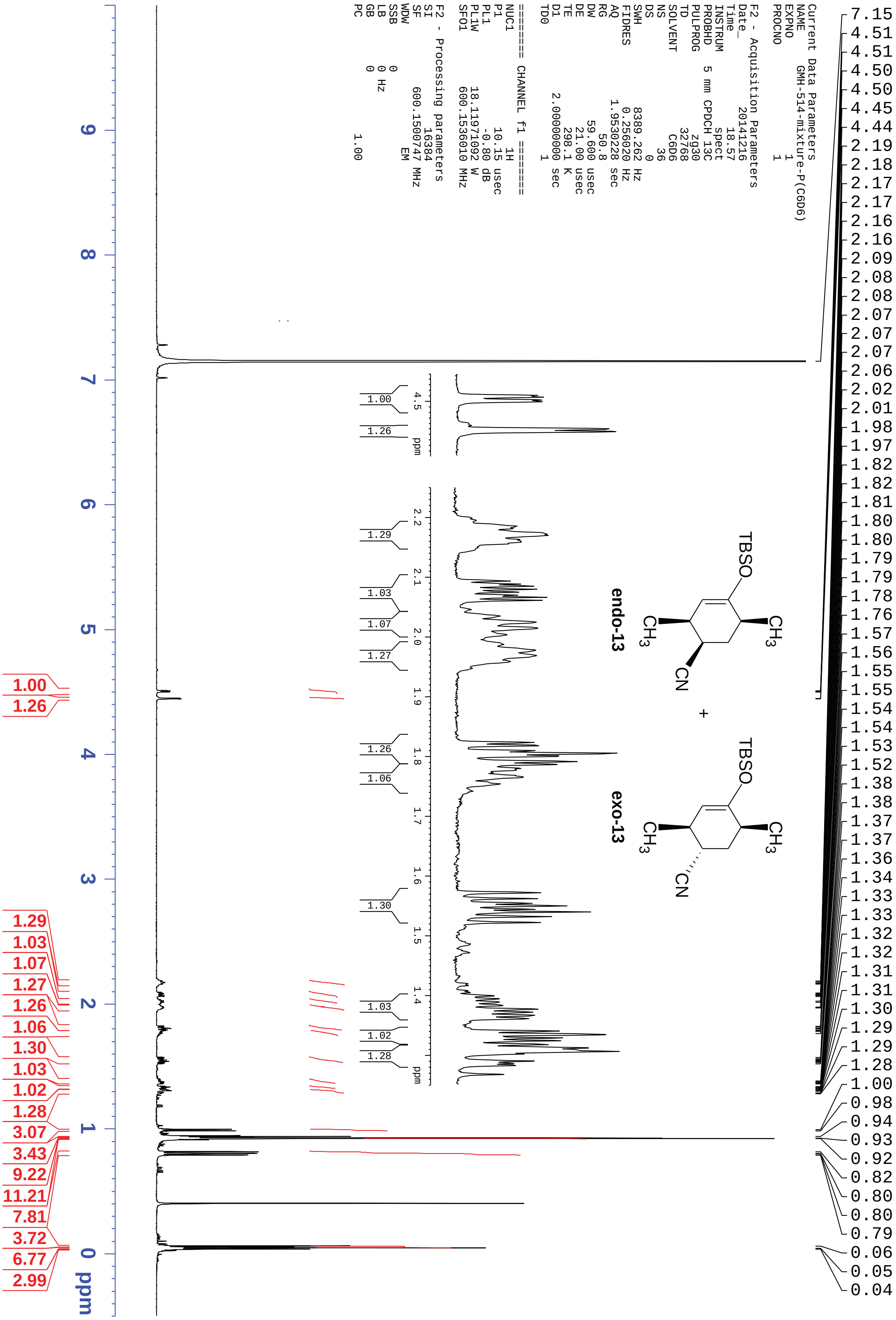

Supplementary Figure 19. ^{1}H NMR spectrum of mixed compounds endo/exo-13.

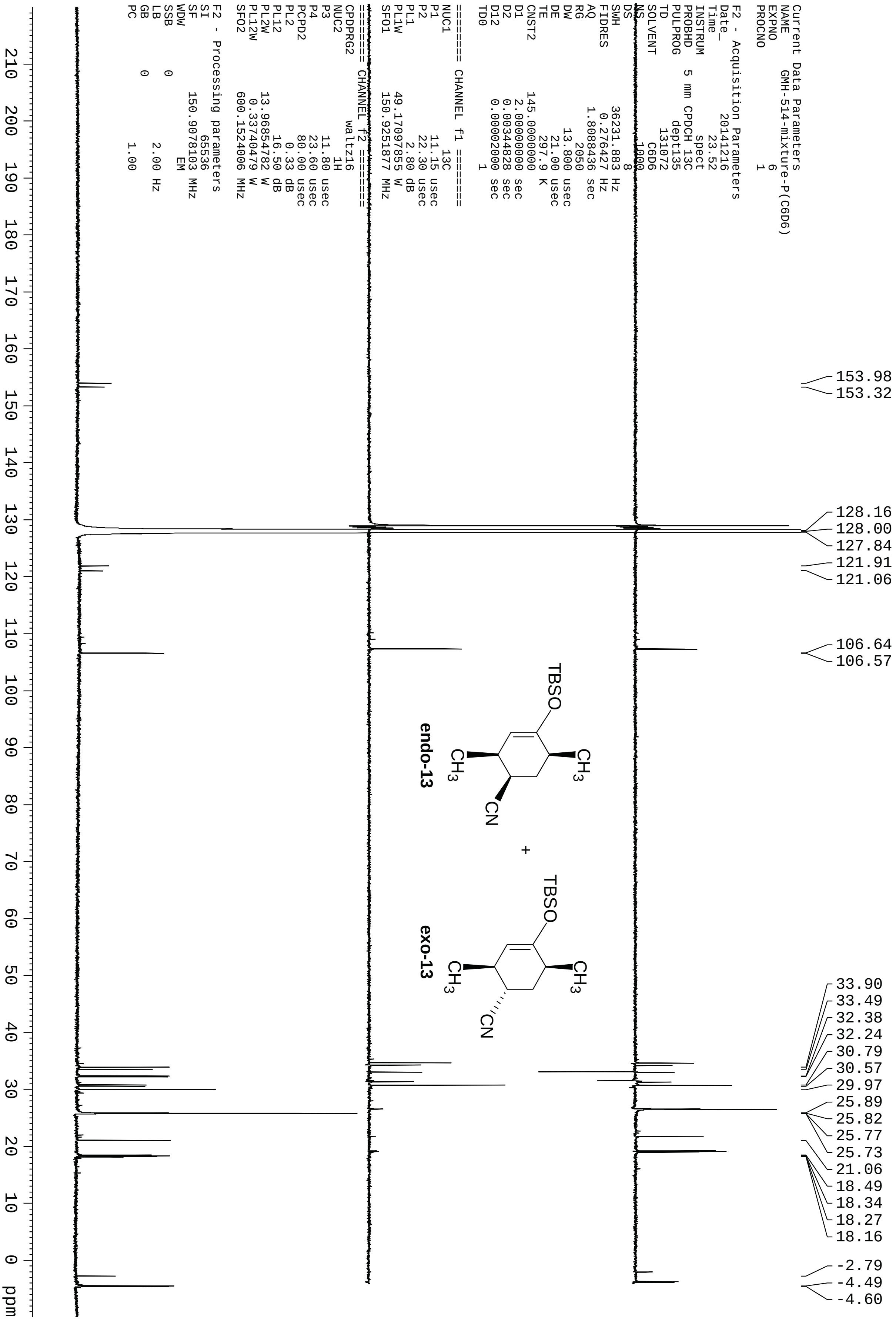

Supplementary Figure 20. ^{13}C and DEPT NMR spectra of mixed compound endo/exo-13.

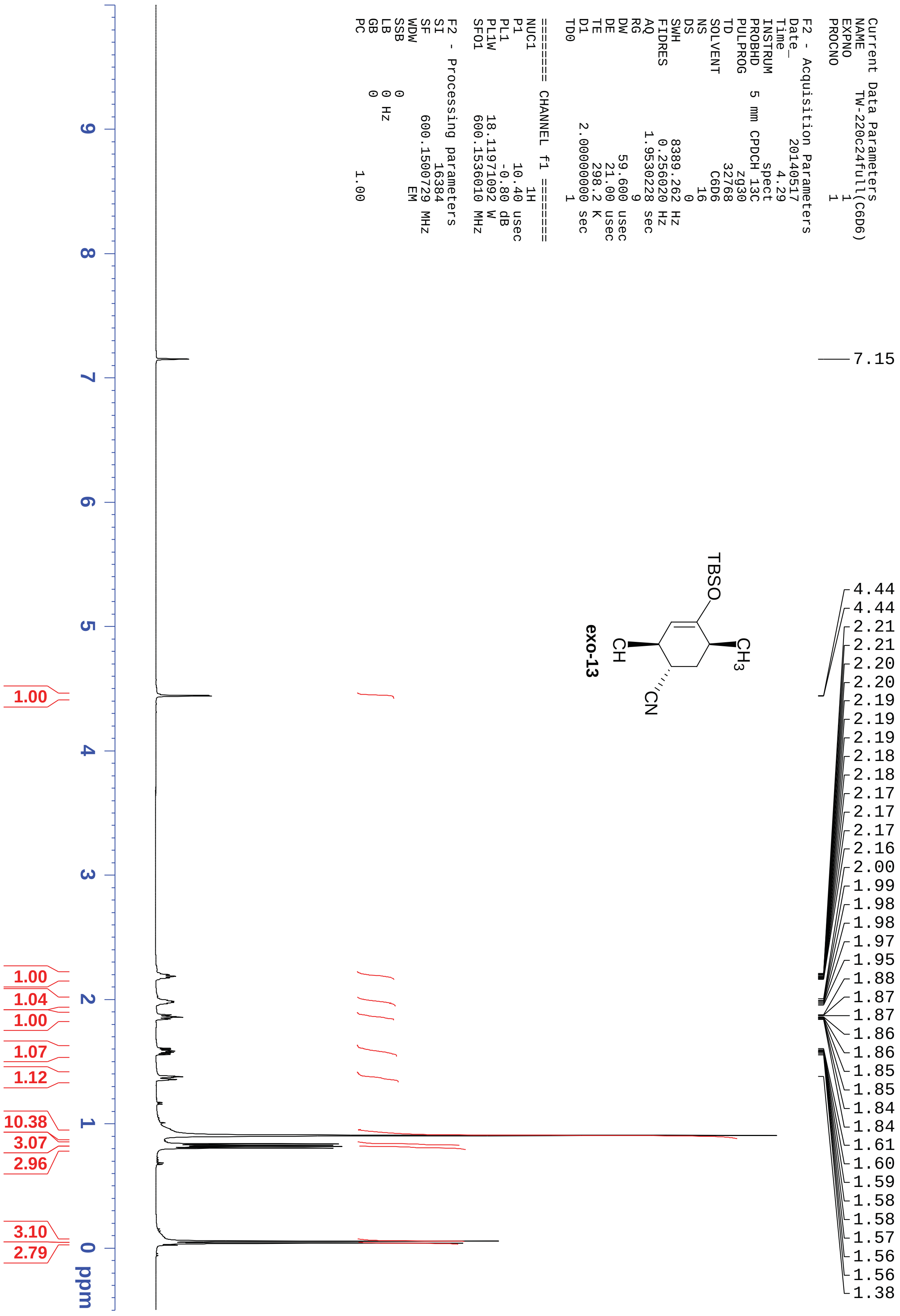

Supplementary Figure 21. **^{1}H NMR spectrum of compound exo-13.**

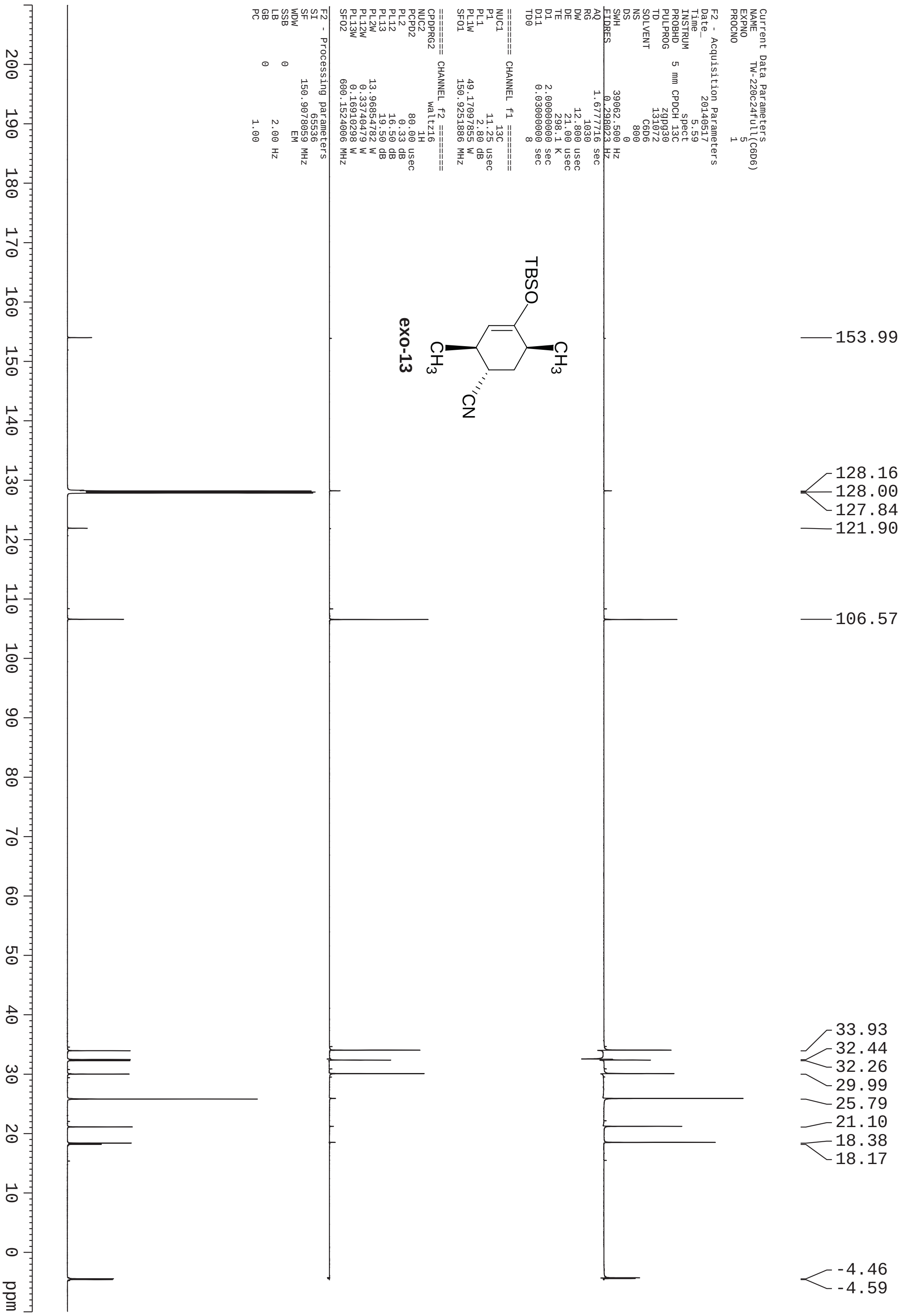

Supplementary Figure 22. ^{13}C DEPT NMR spectrum of compound exo-13.

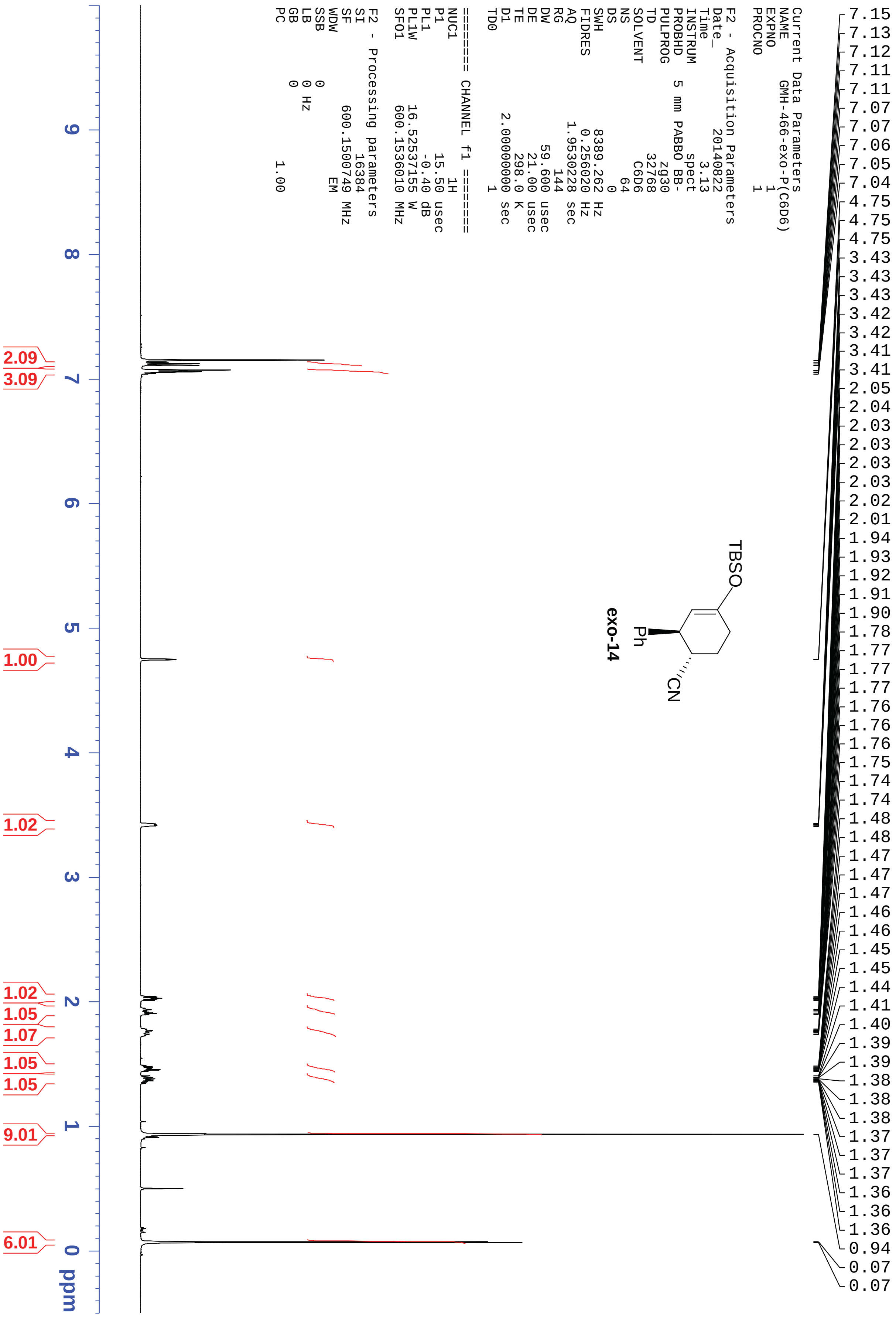

Supplementary Figure 23. ^{1}**H NMR spectrum of compound exo-14.**

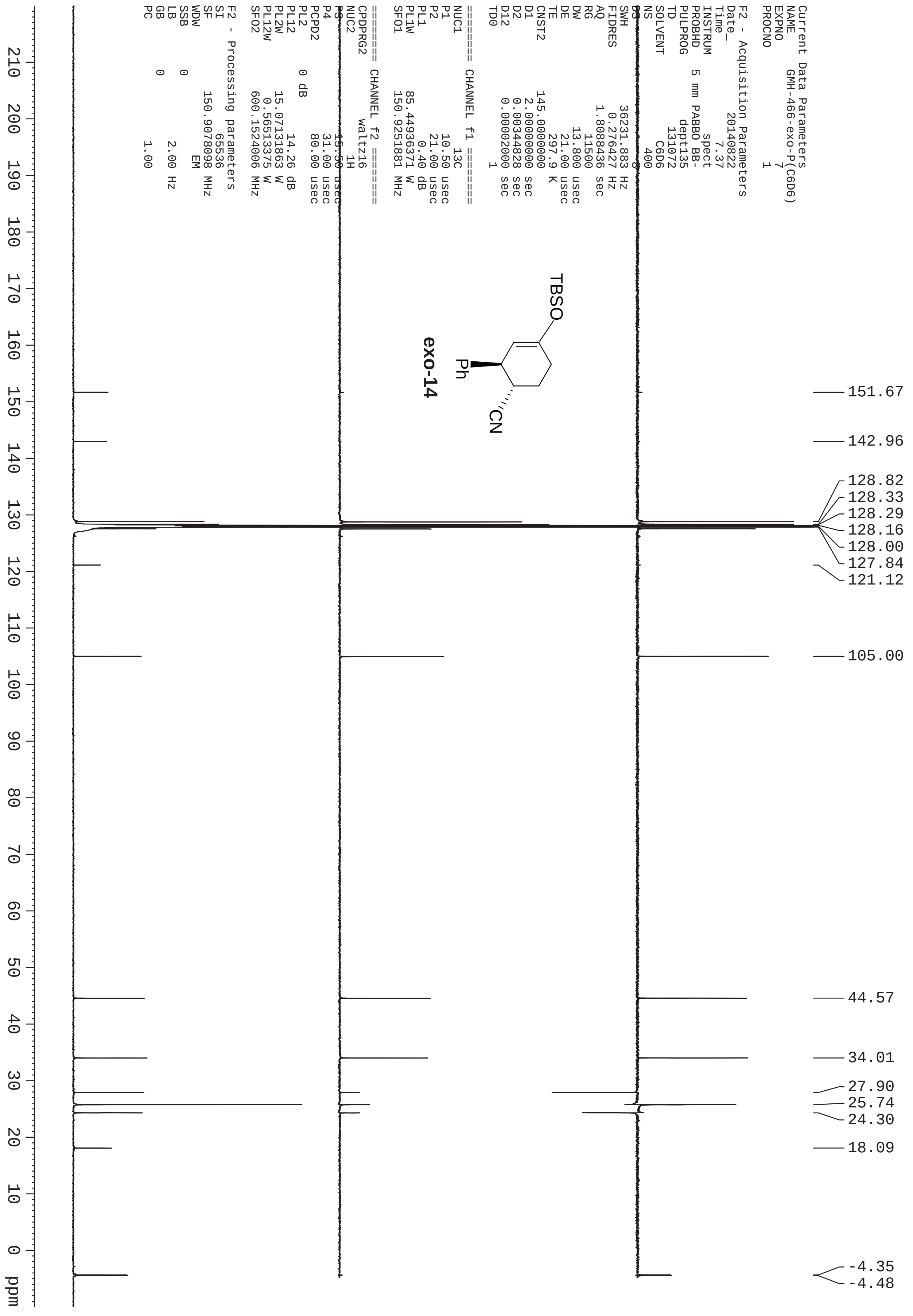

Supplementary Figure 24. ^{13}C DEPT NMR spectrum of compound exo-14.

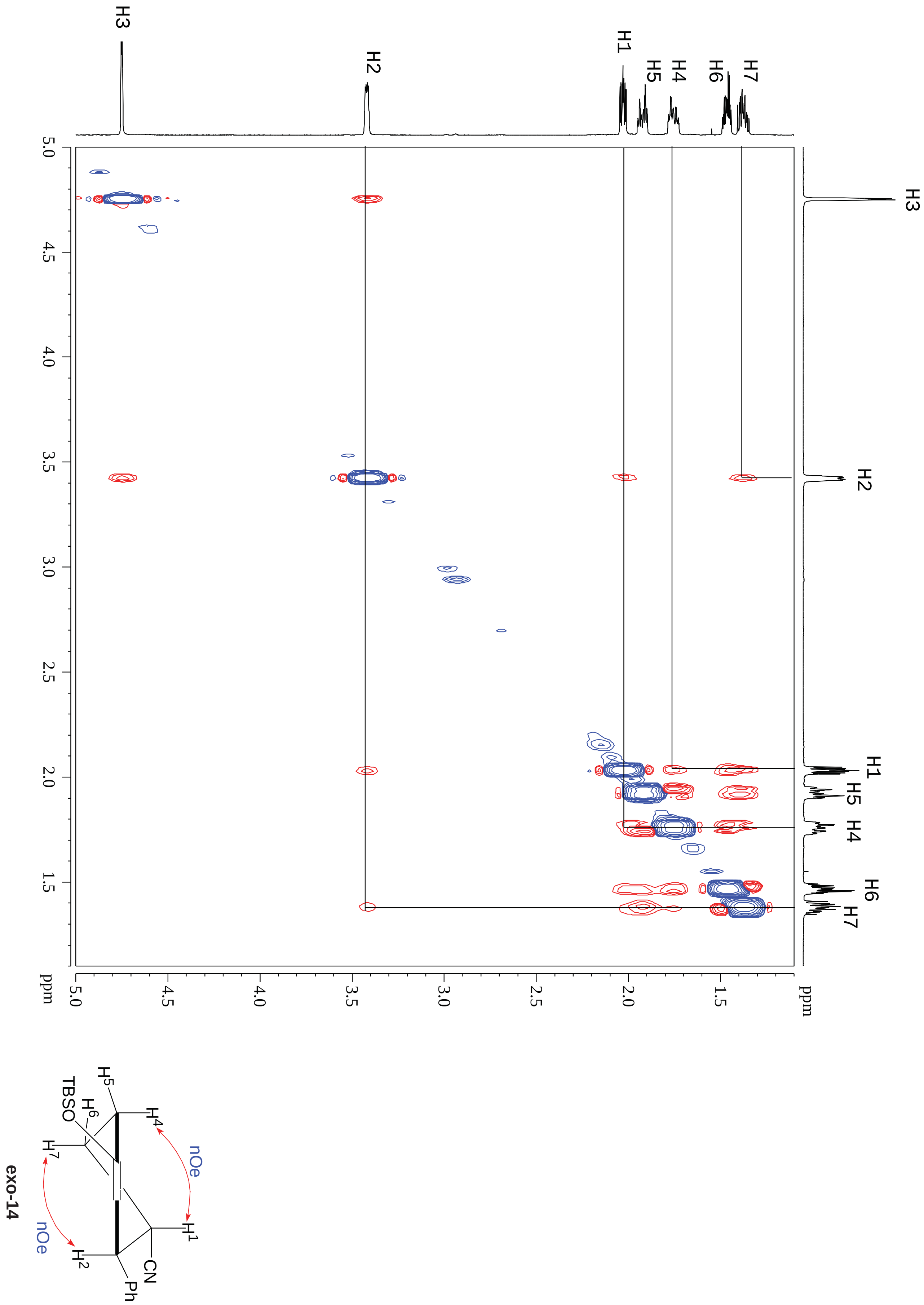

Supplementary Figure 25. NOESY NMR spectrum of compound exo-14.

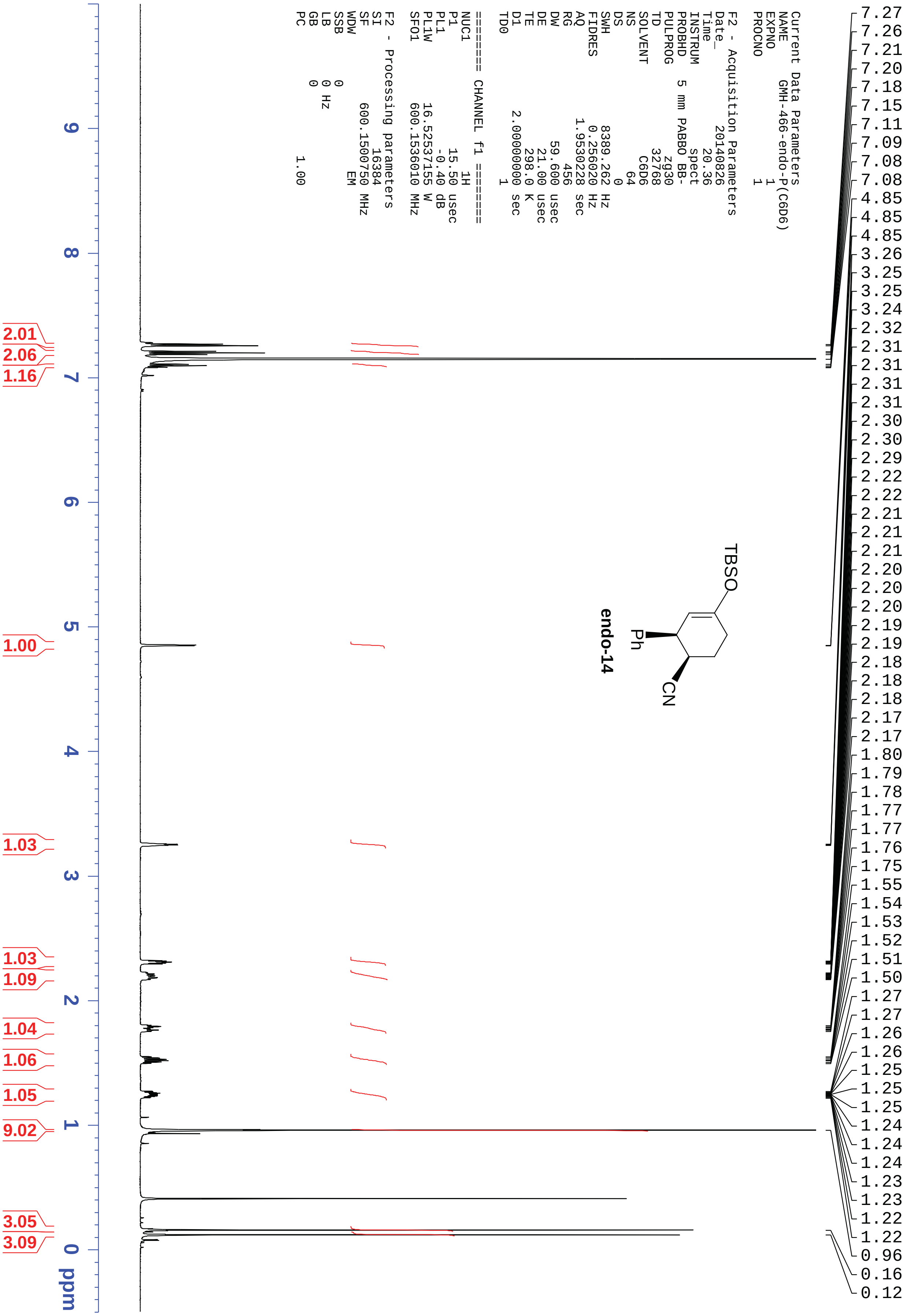

Supplementary Figure 26. ^{1}H NMR spectrum of compound endo-14.

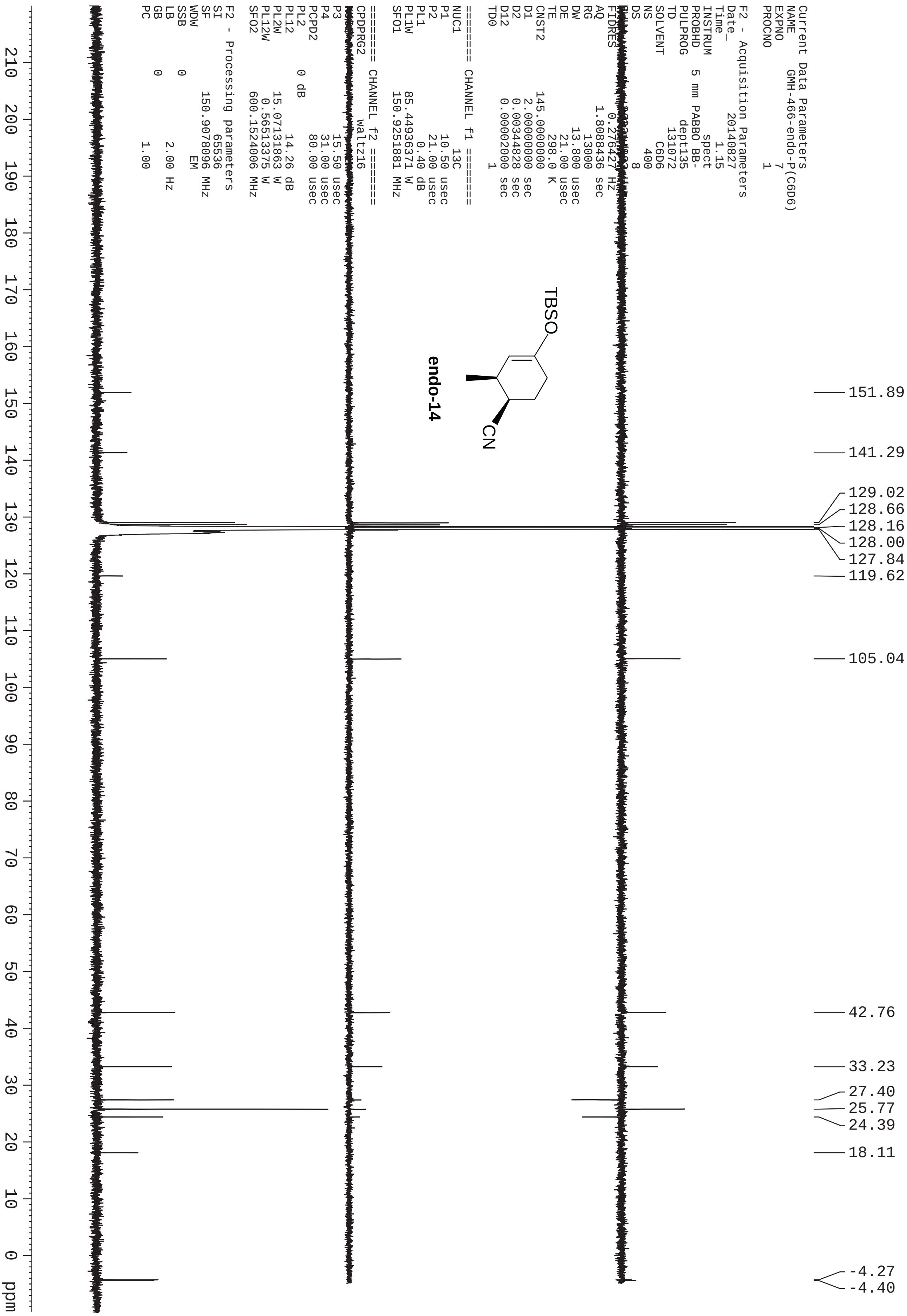

Supplementary Figure 27. ^{13}C and DEPT NMR spectra of compound endo-14.

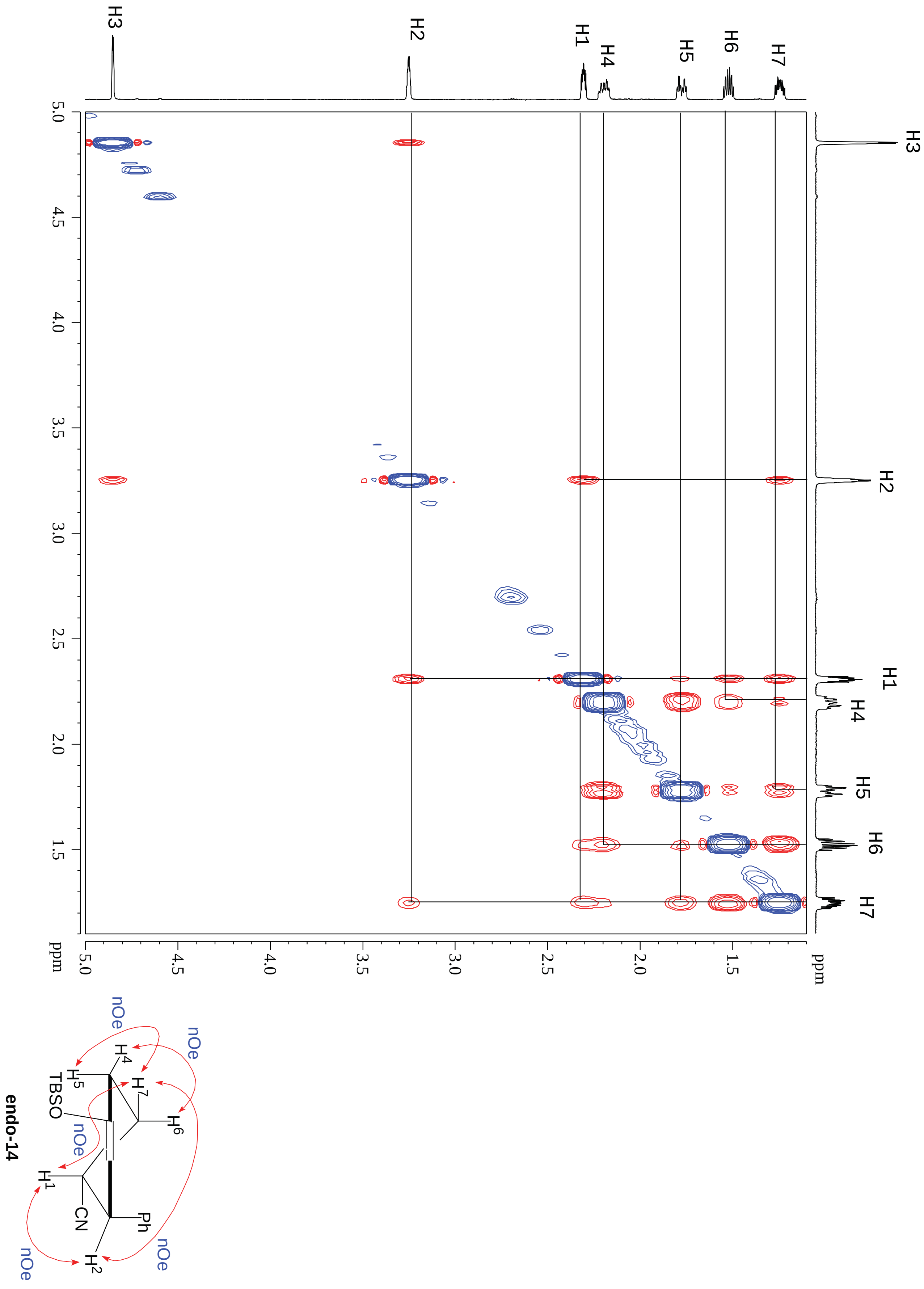

Supplementary Figure 28. NOESY NMR spectrum of compound endo-14.

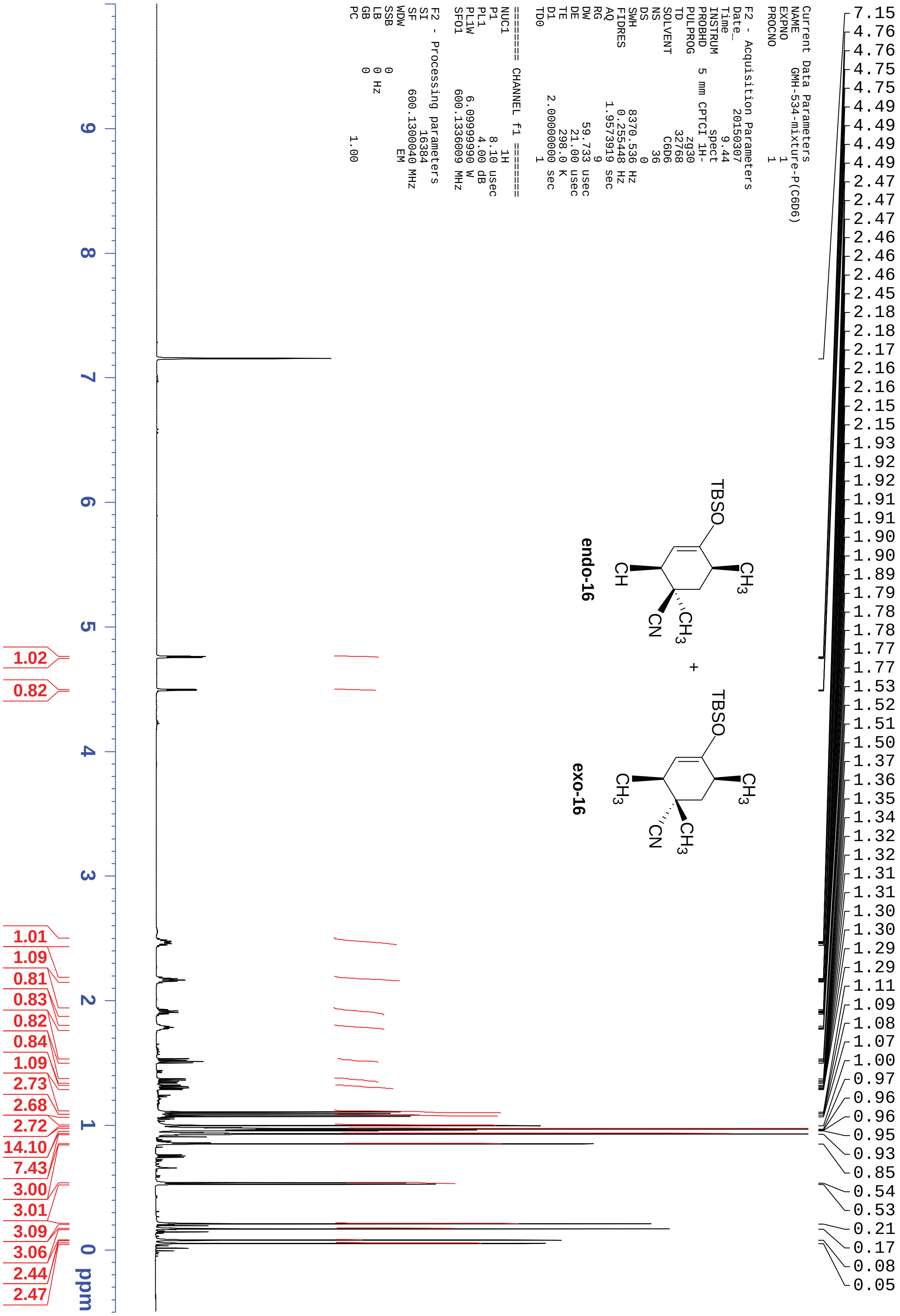

Supplementary Figure 29. ^{1}H NMR spectrum of mixed compound endo/exo-16.

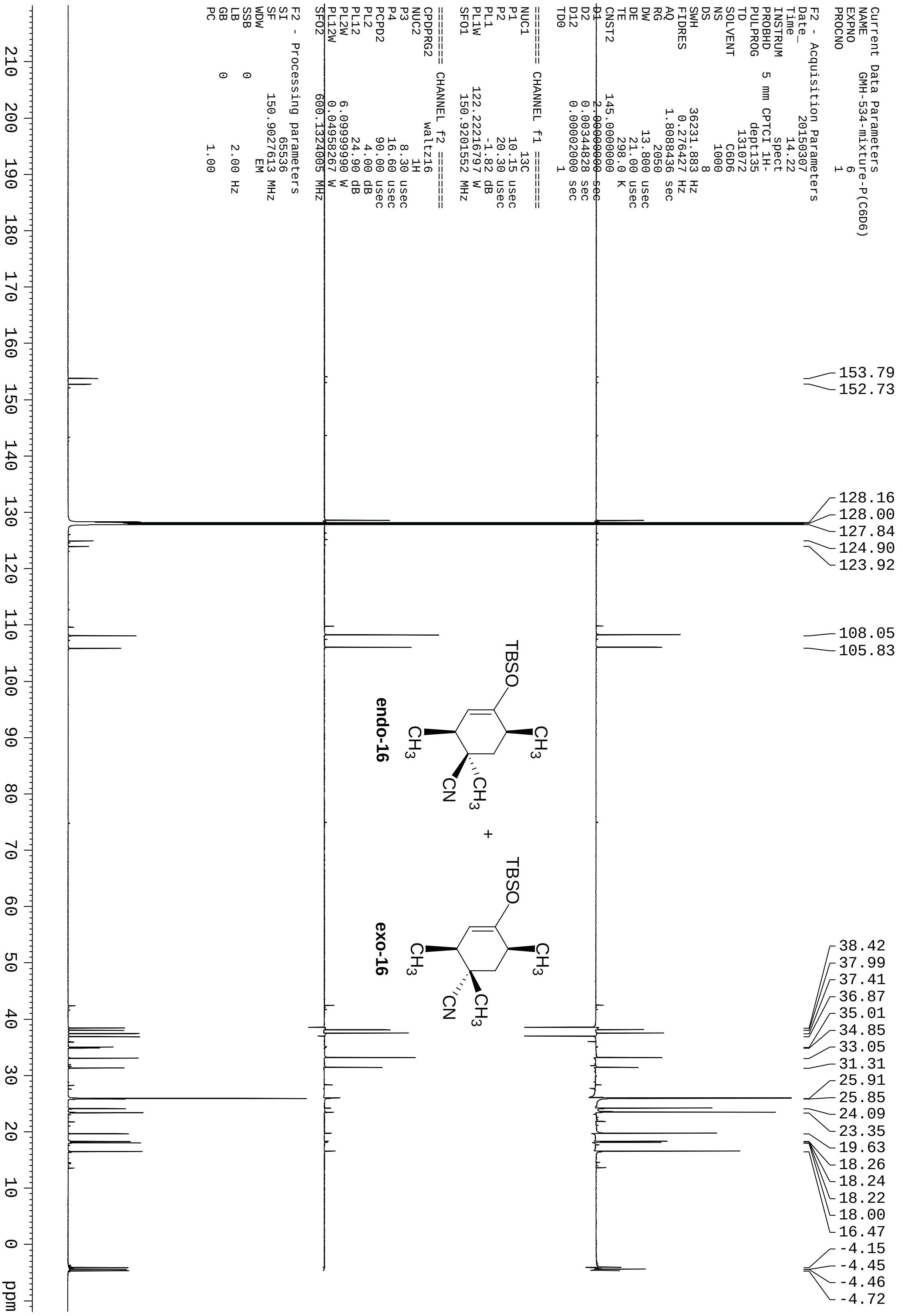

Supplementary Figure 30. ^{13}C and DEPT NMR spectra of mixed compound endo/exo-16.

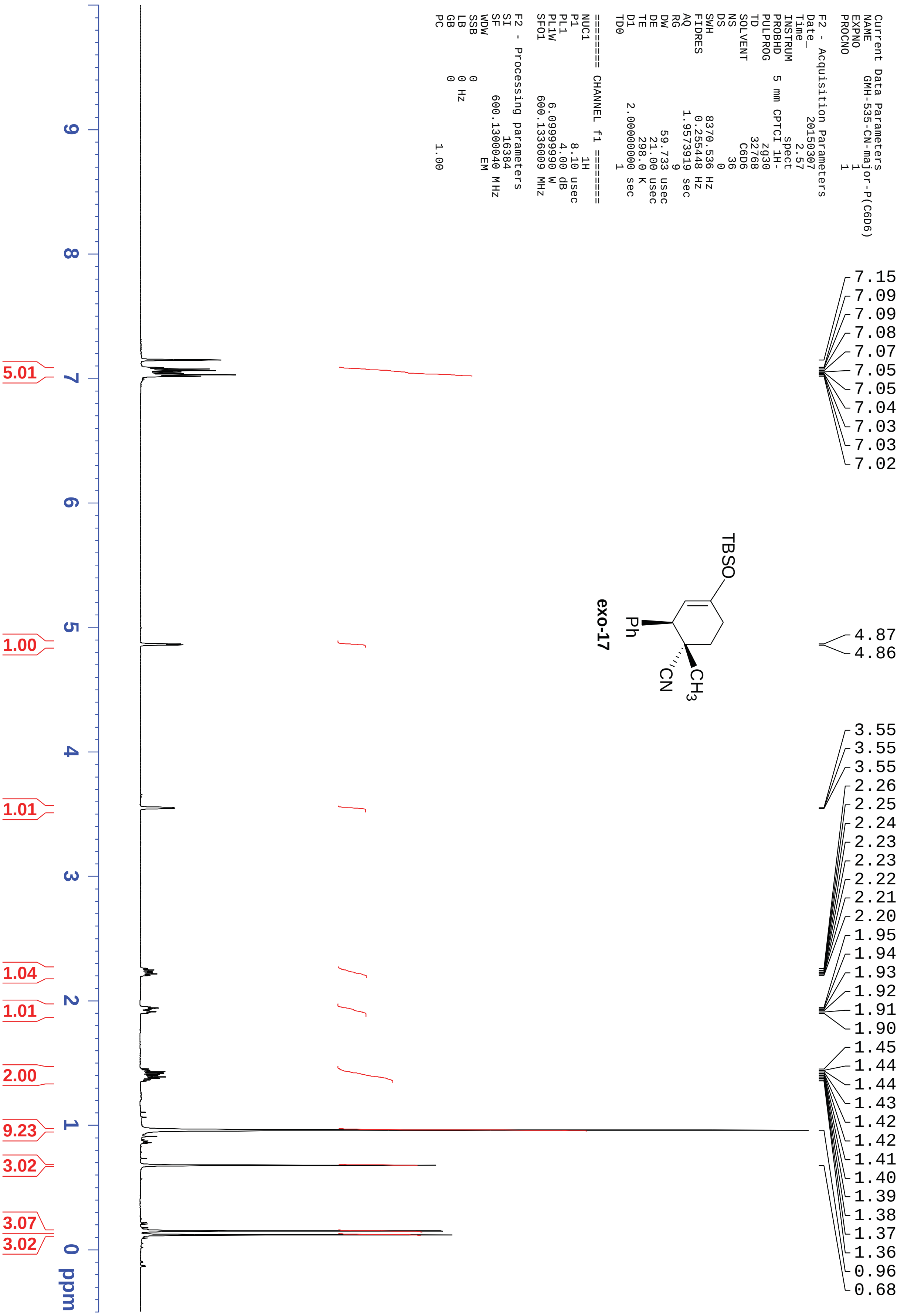

Supplementary Figure 31. **^{1}H NMR spectrum of compound exo-17.**

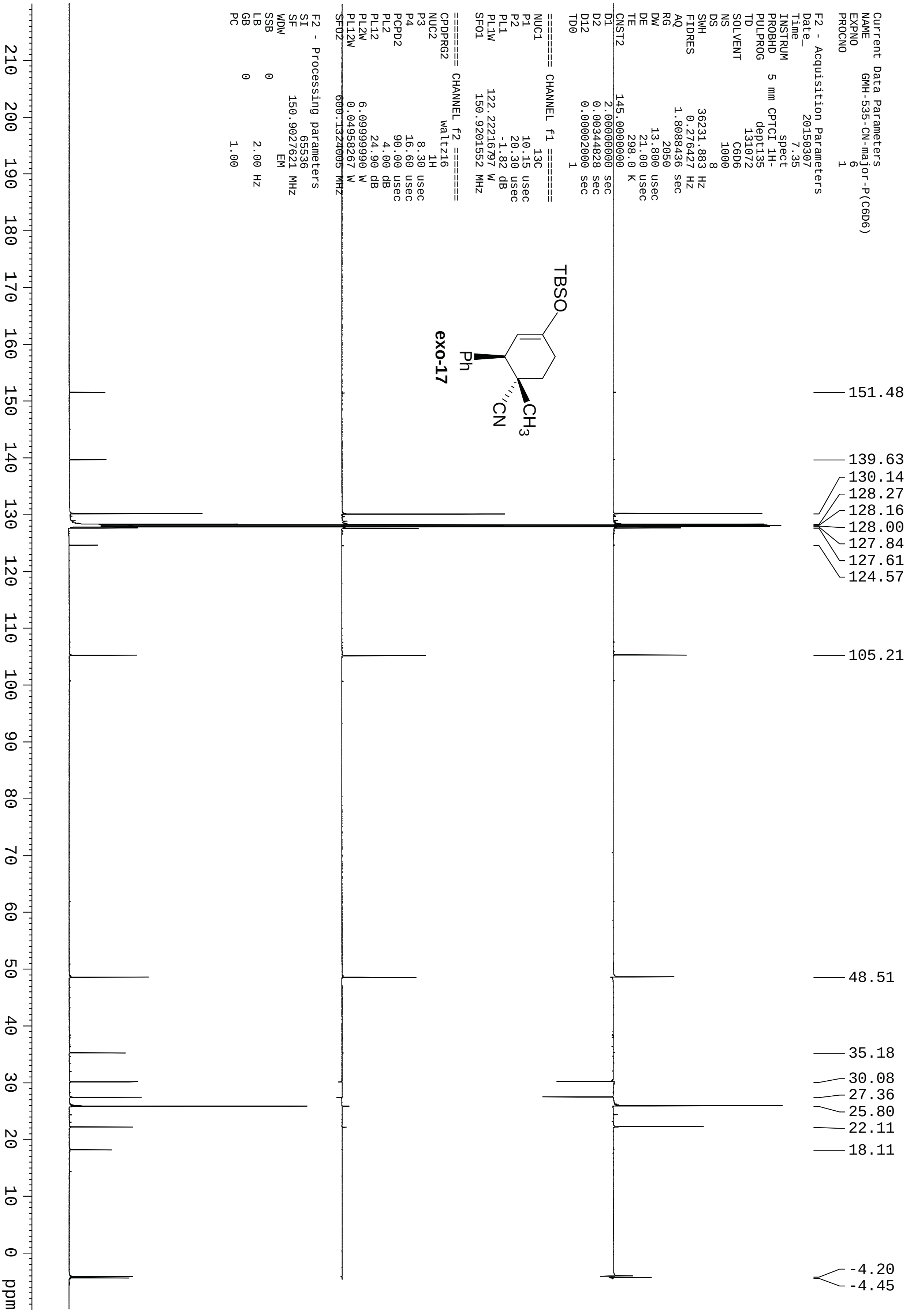

Supplementary Figure 32. 13C and DEPT NMR spectra of compound exo-17.

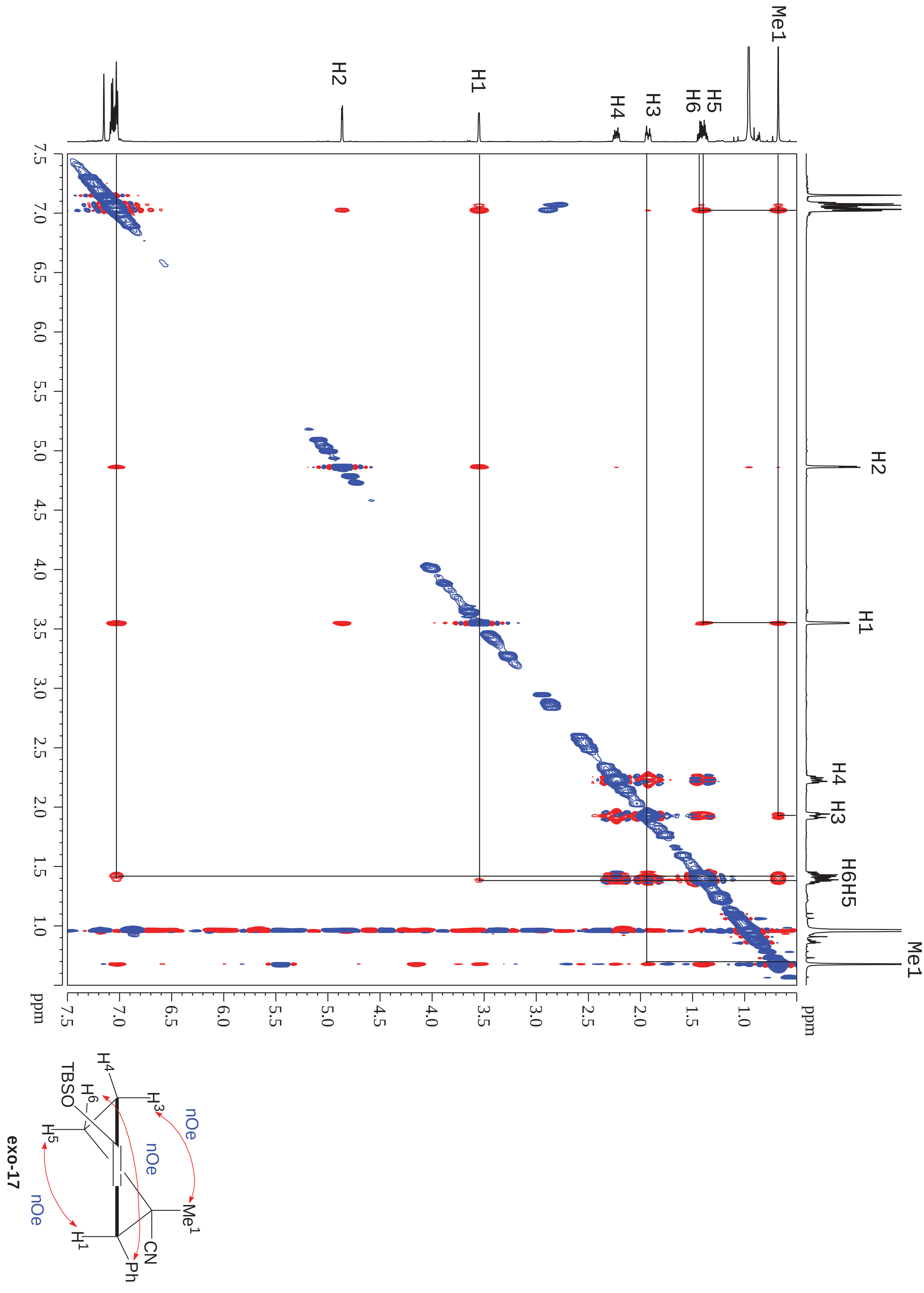

Supplementary Figure 33. NOESY NMR spectrum of compound exo-17.

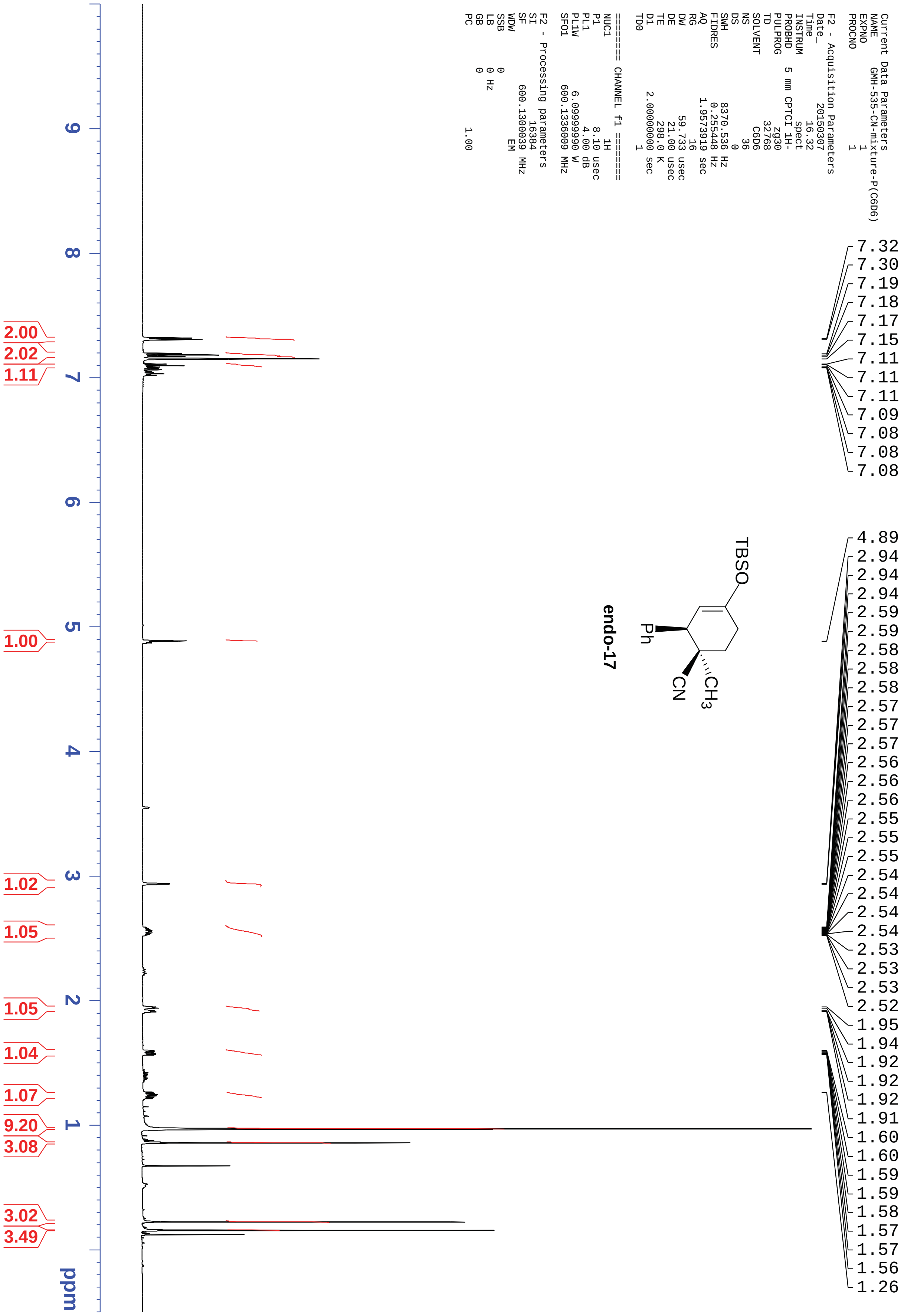

Supplementary Figure 34. ¹H NMR spectrum of compound endo-17.

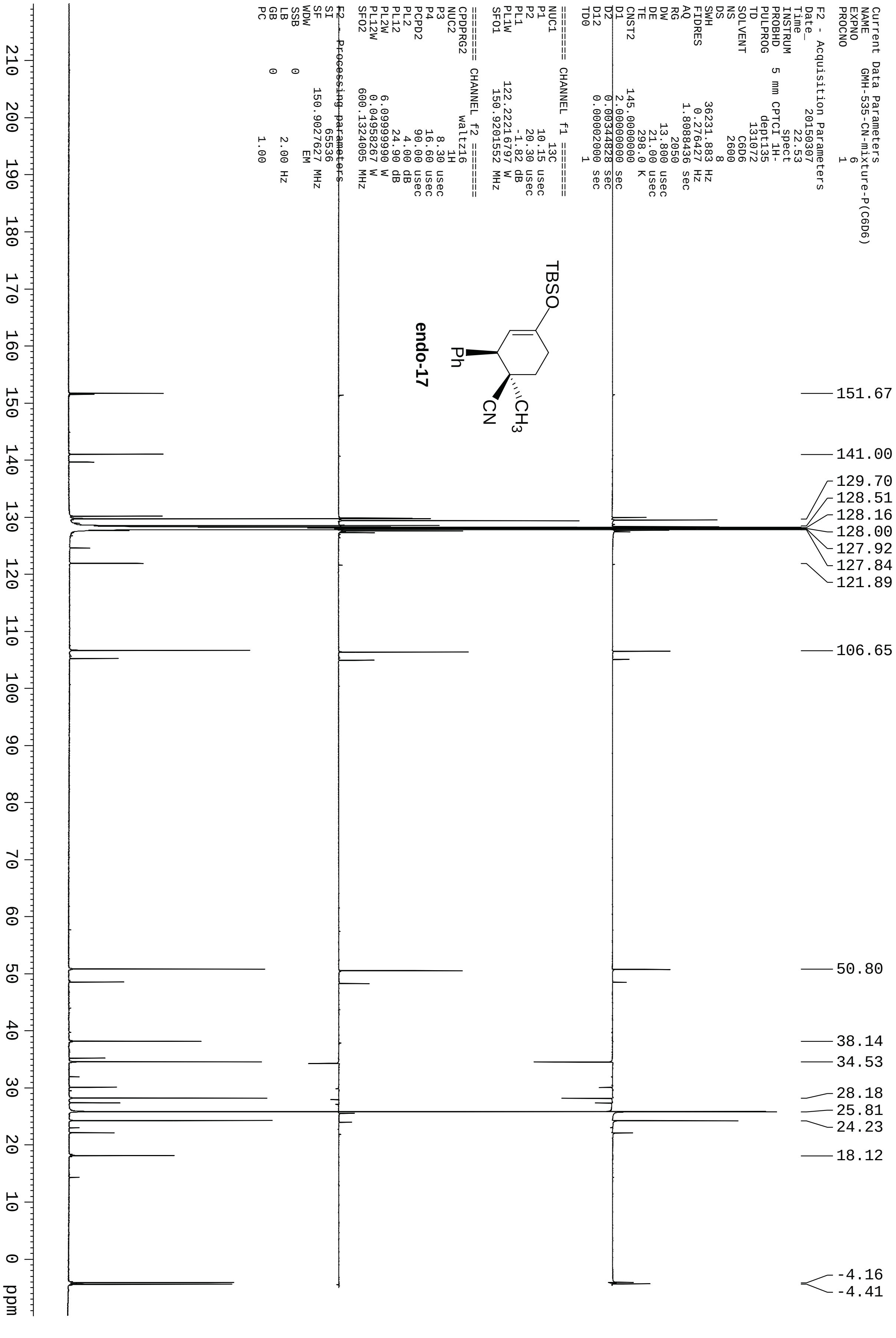

Supplementary Figure 35. ^{13}C and DEPT NMR spectra of compound endo-17.

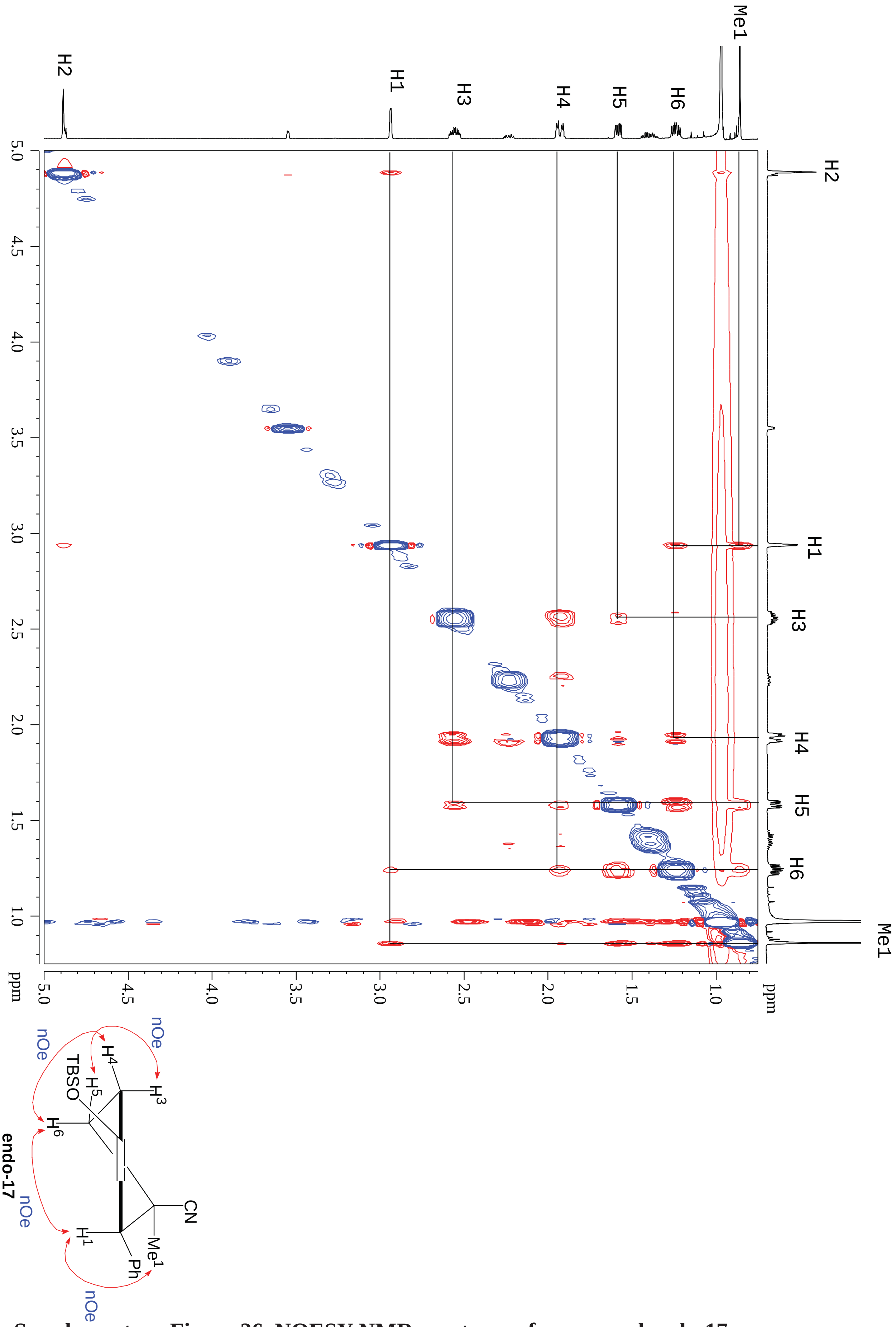

Supplementary Figure 36. NOESY NMR spectrum of compound endo-17.

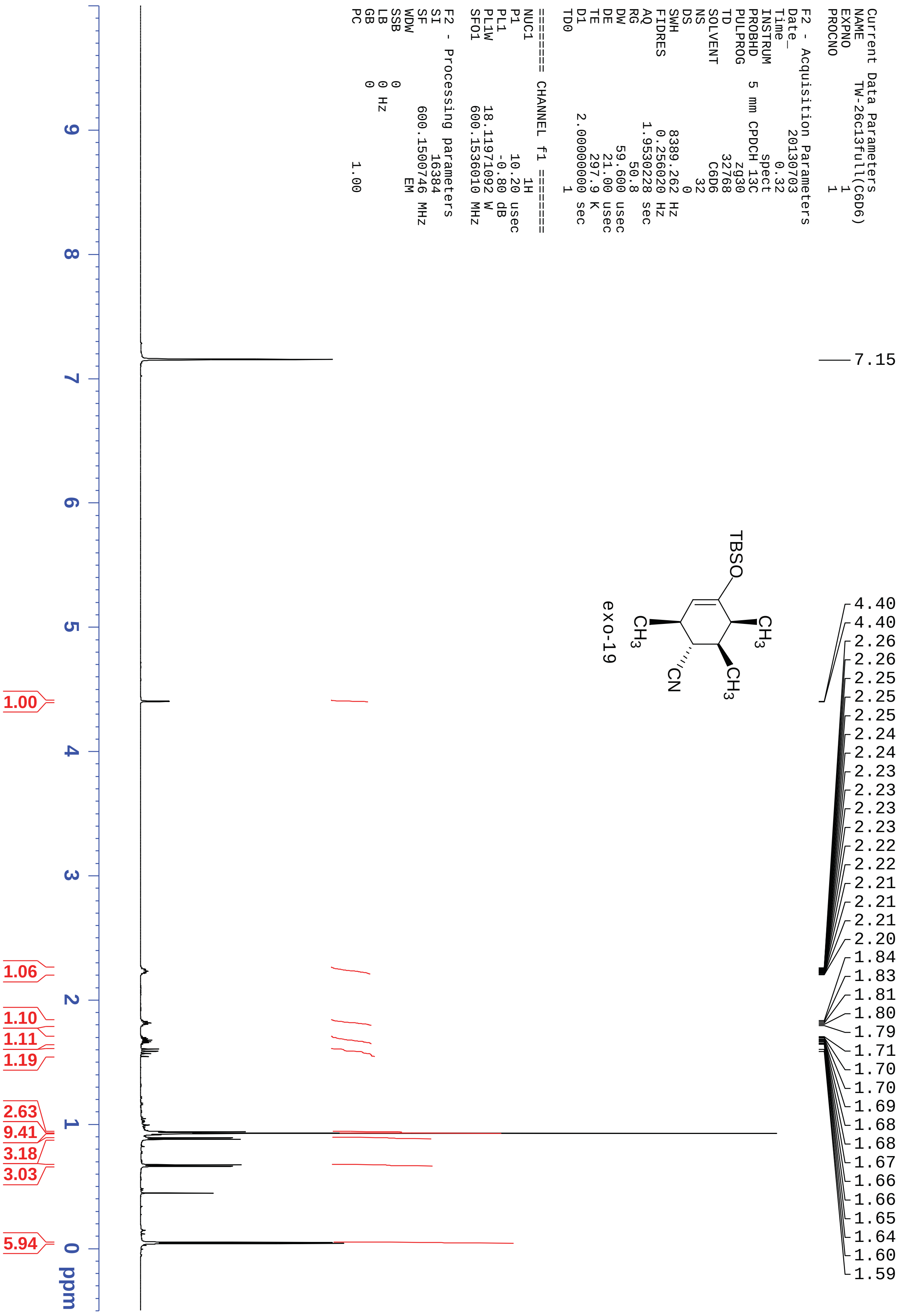

Supplementary Figure 37. **^{1}H NMR spectrum of compound exo-19.**

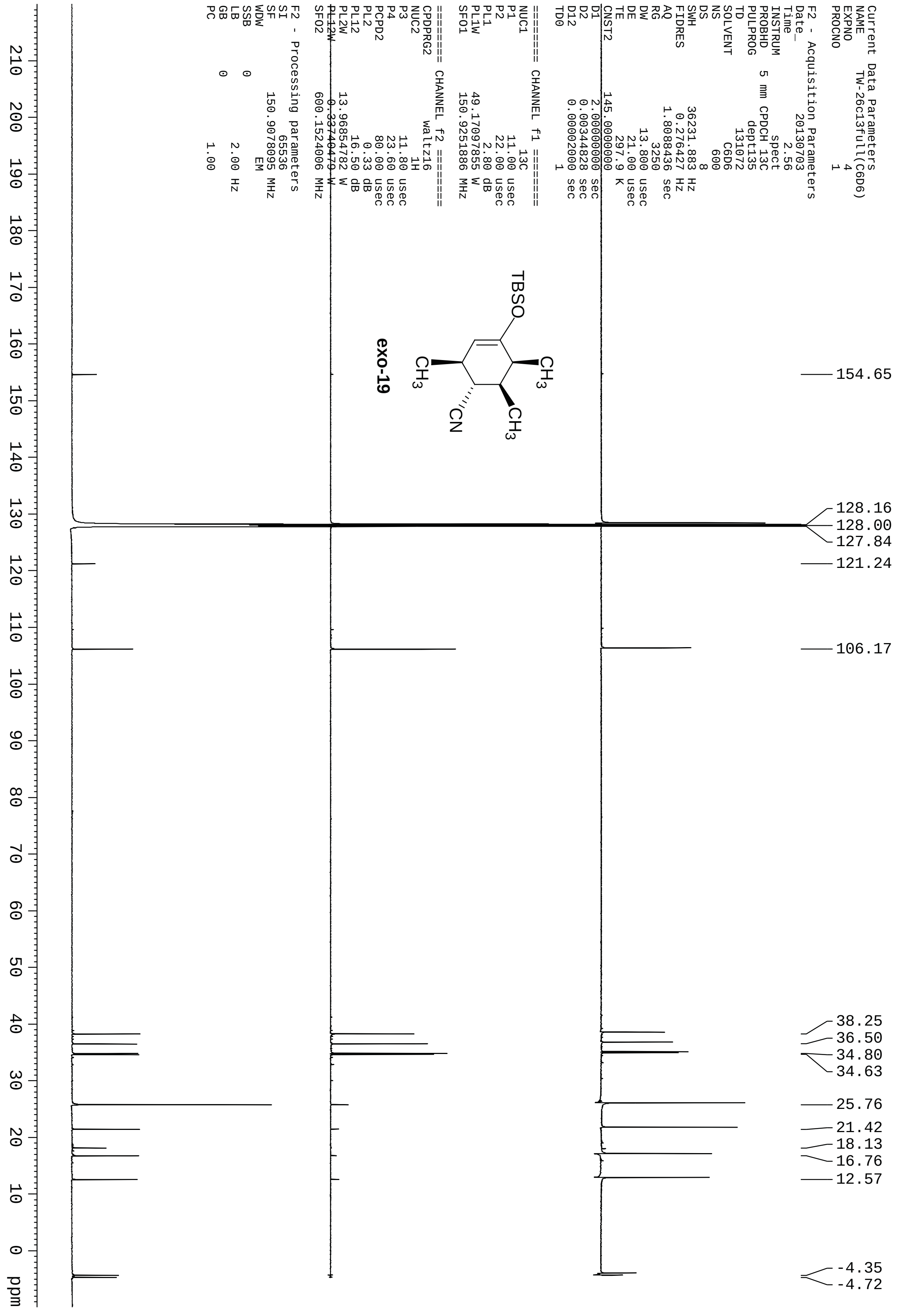

Supplementary Figure 38. ^{13}C and DEPT NMR spectra of compound exo-19.

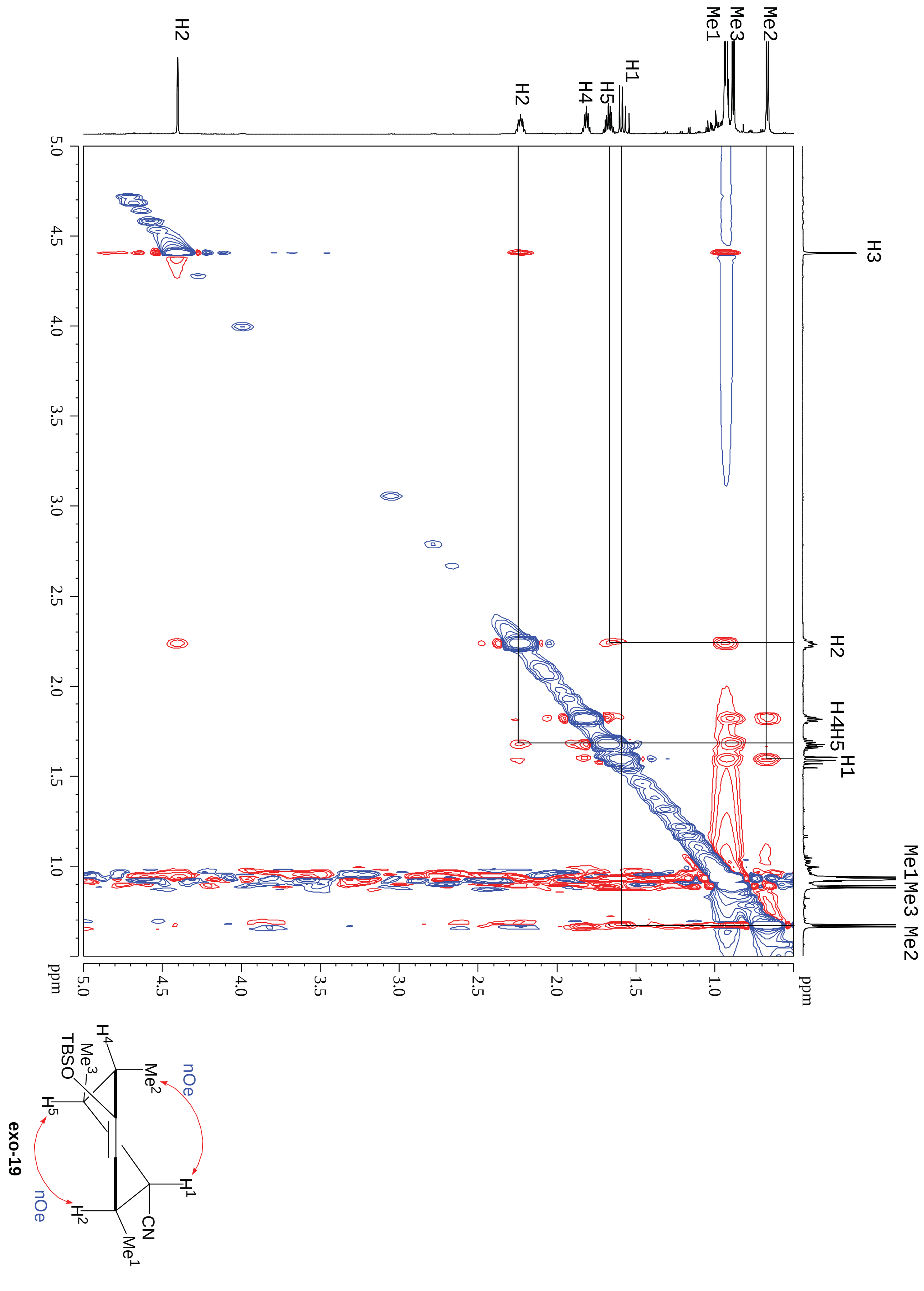

Supplementary Figure 39. NOESY NMR spectrum of compound exo-19.

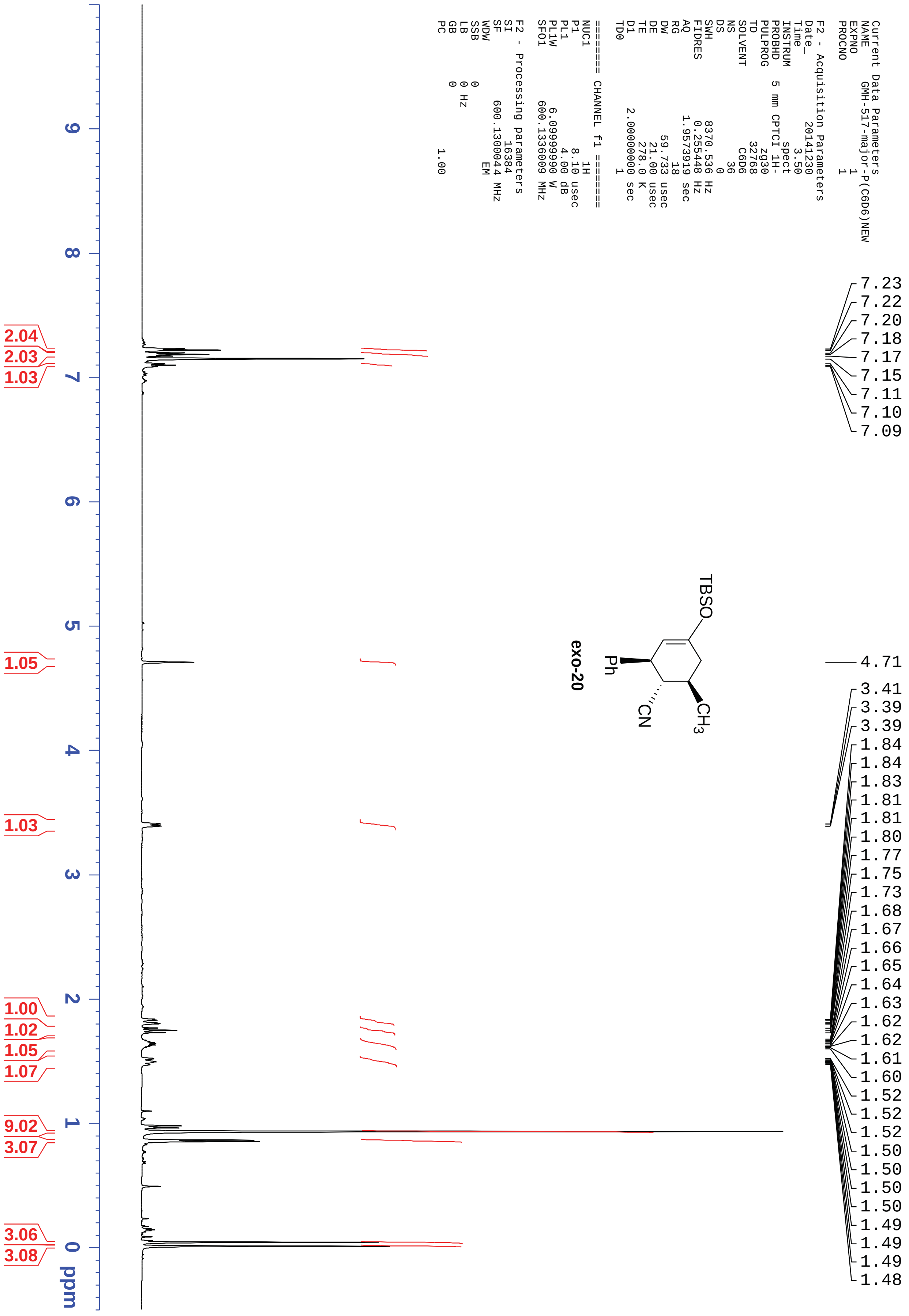

Supplementary Figure 40. ^{1}**H NMR spectrum of compound exo-20.**

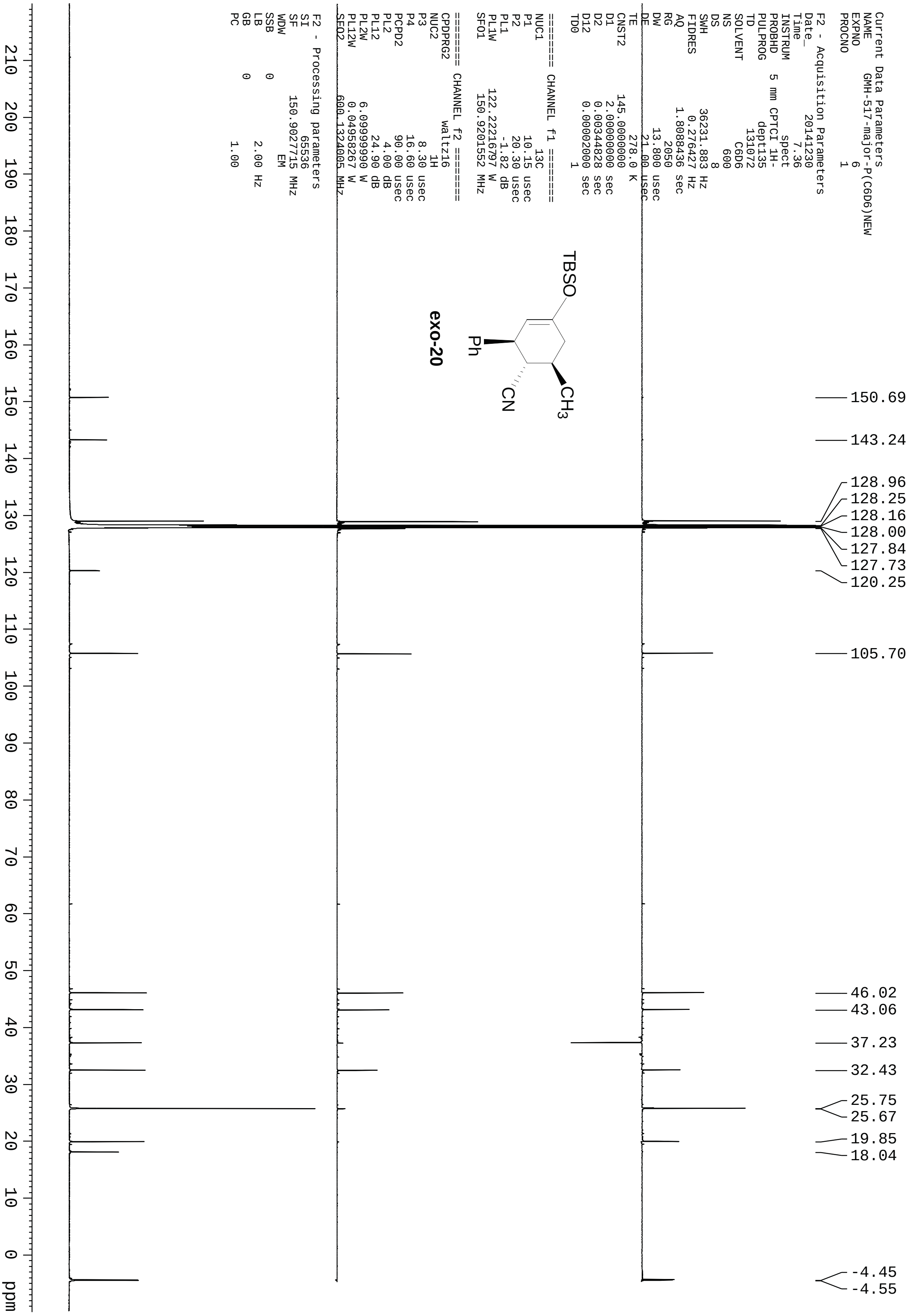

Supplementary Figure 41. ^{13}C and DEPT NMR spectra of compound exo-20.

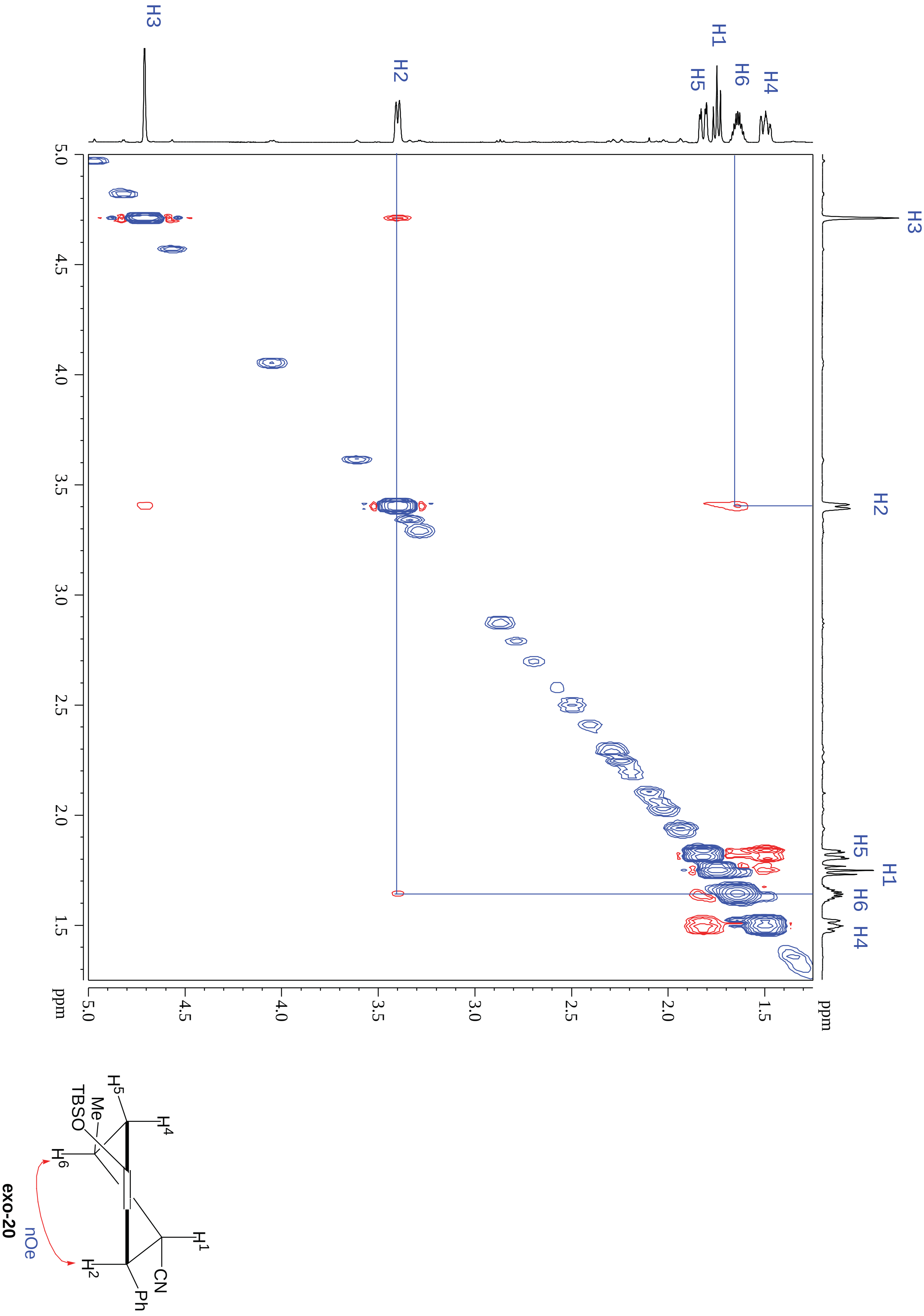

Supplementary Figure 42. NOESY NMR spectrum of compound exo-20.

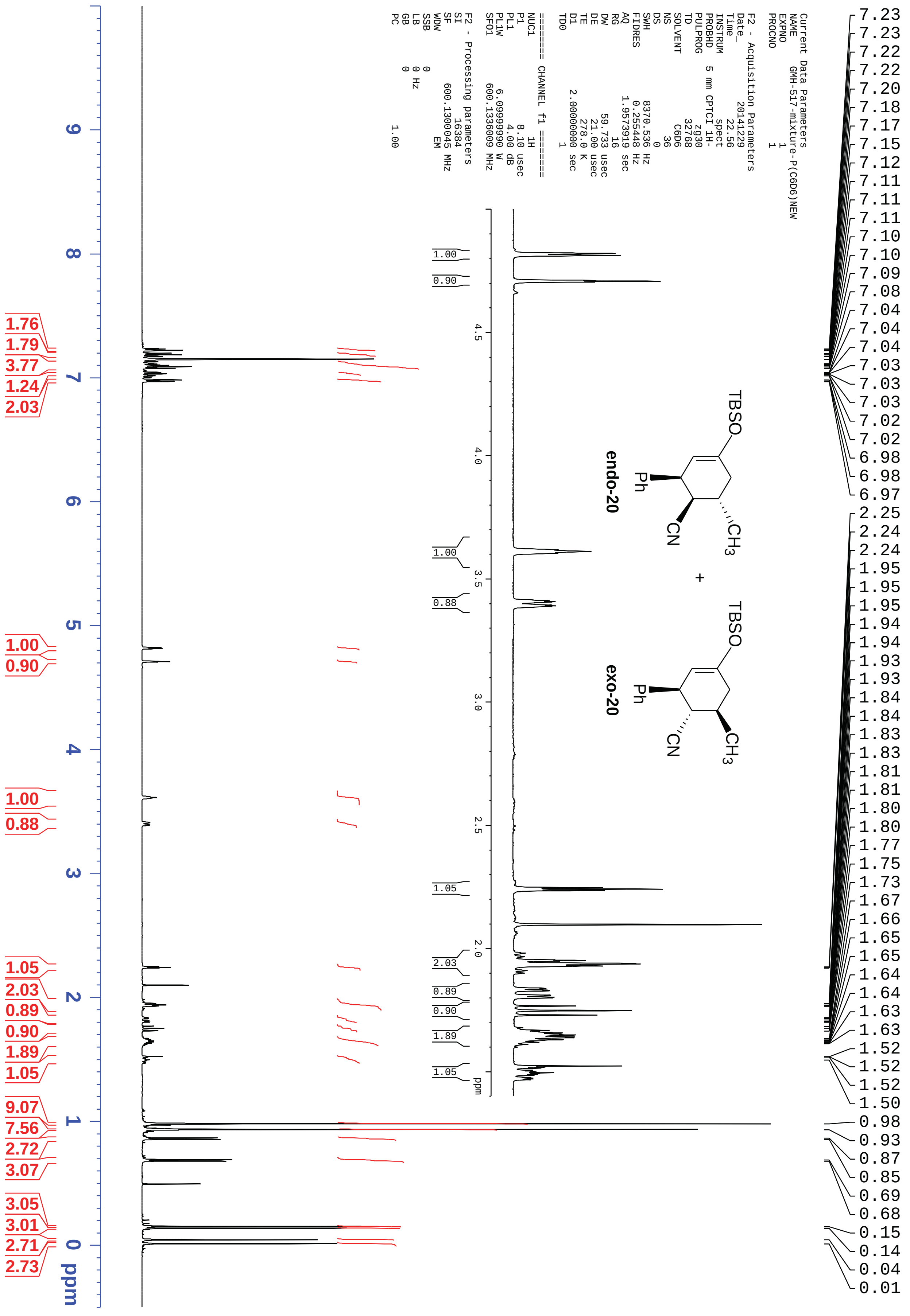

Supplementary Figure 43. ^{1}H NMR spectrum of mixed compounds exo/endo-20.

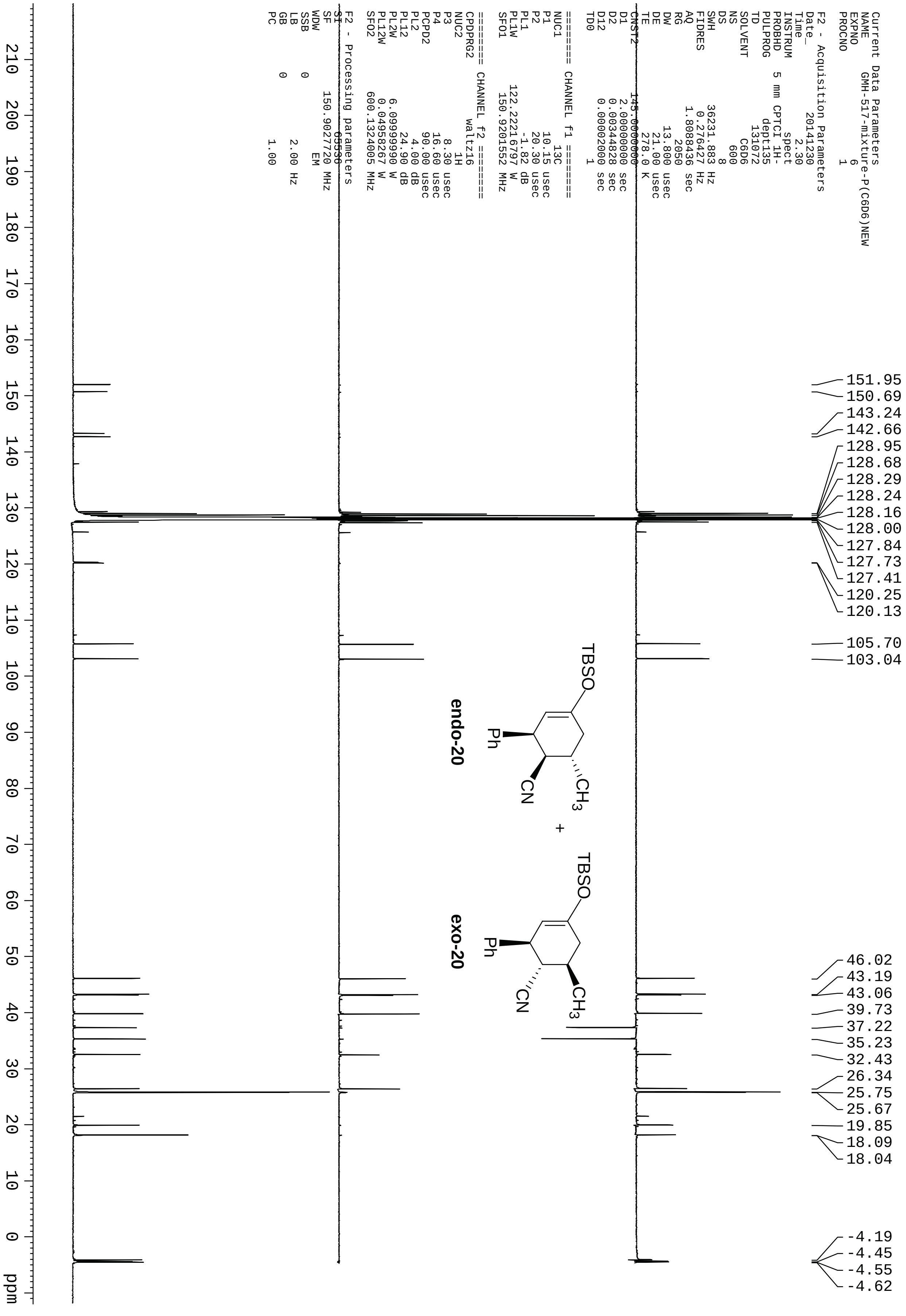

Supplementary Figure 44. ^{13}**C and DEPT NMR spectra of mixed compounds exo/endo-20.**

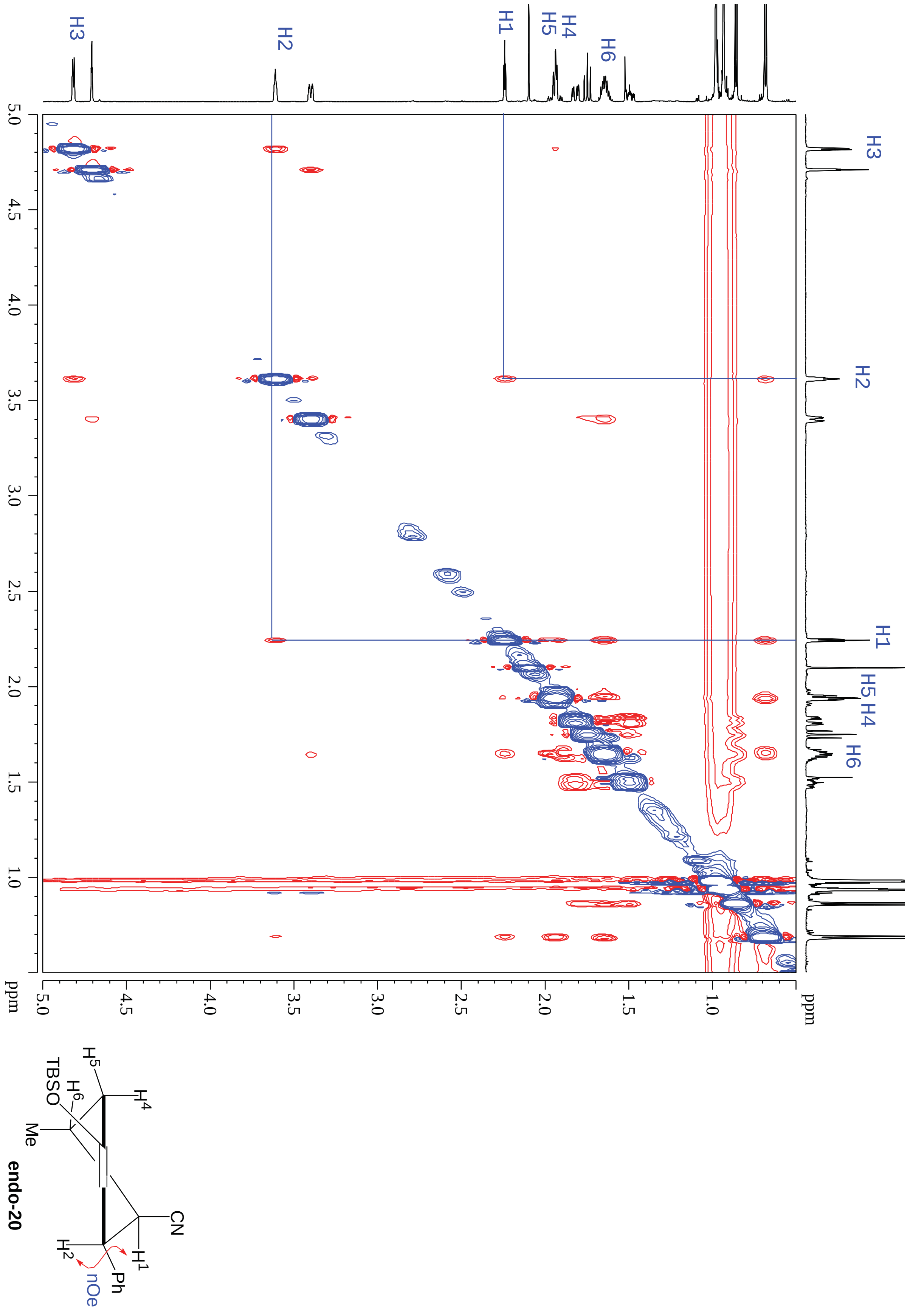

Supplementary Figure 45. NOESY NMR spectrum of mixed compounds endo/exo-20 highlighting the correlation for endo-20.

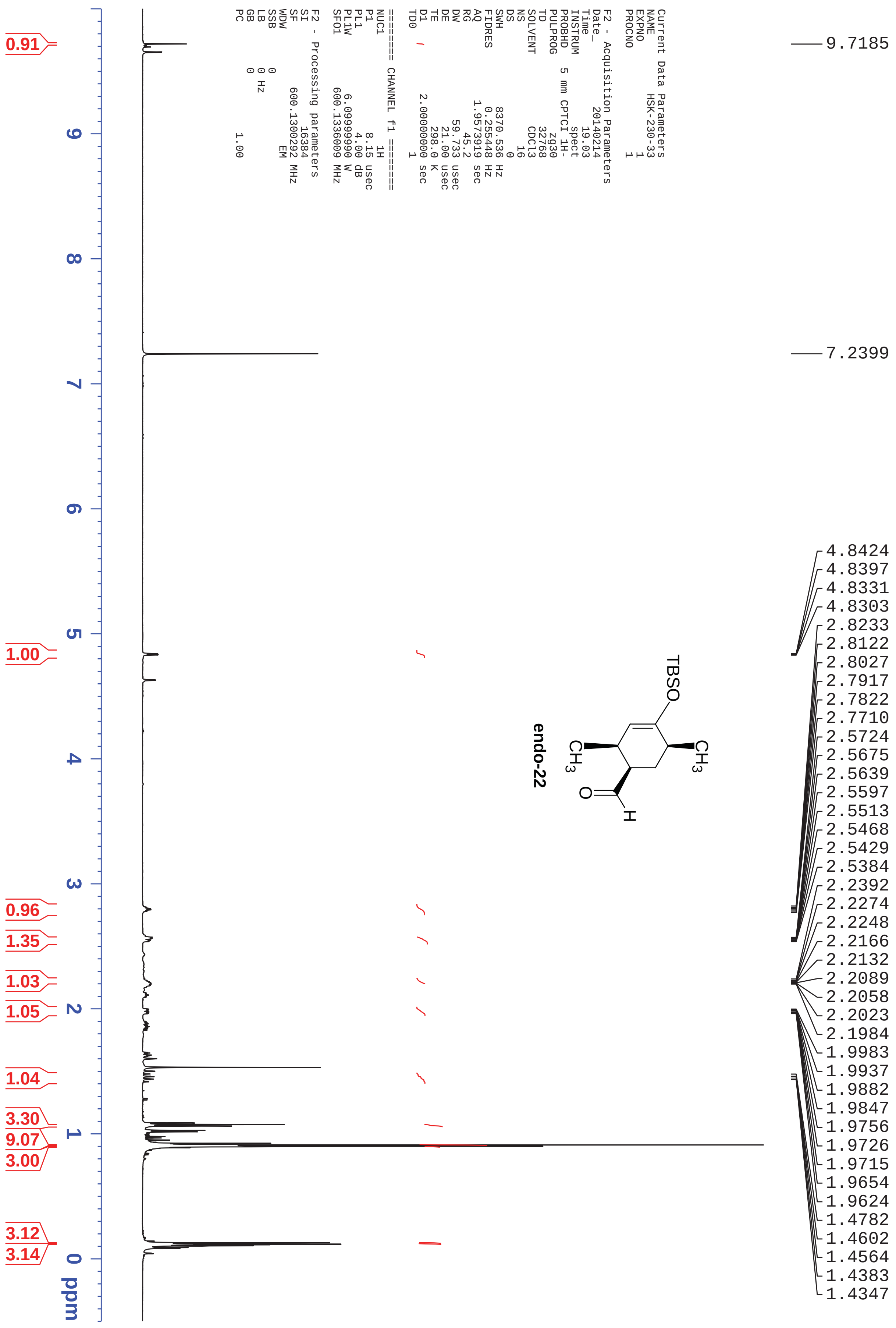

Supplementary Figure 46. ^{1}**H NMR spectrum of compound endo-22.**

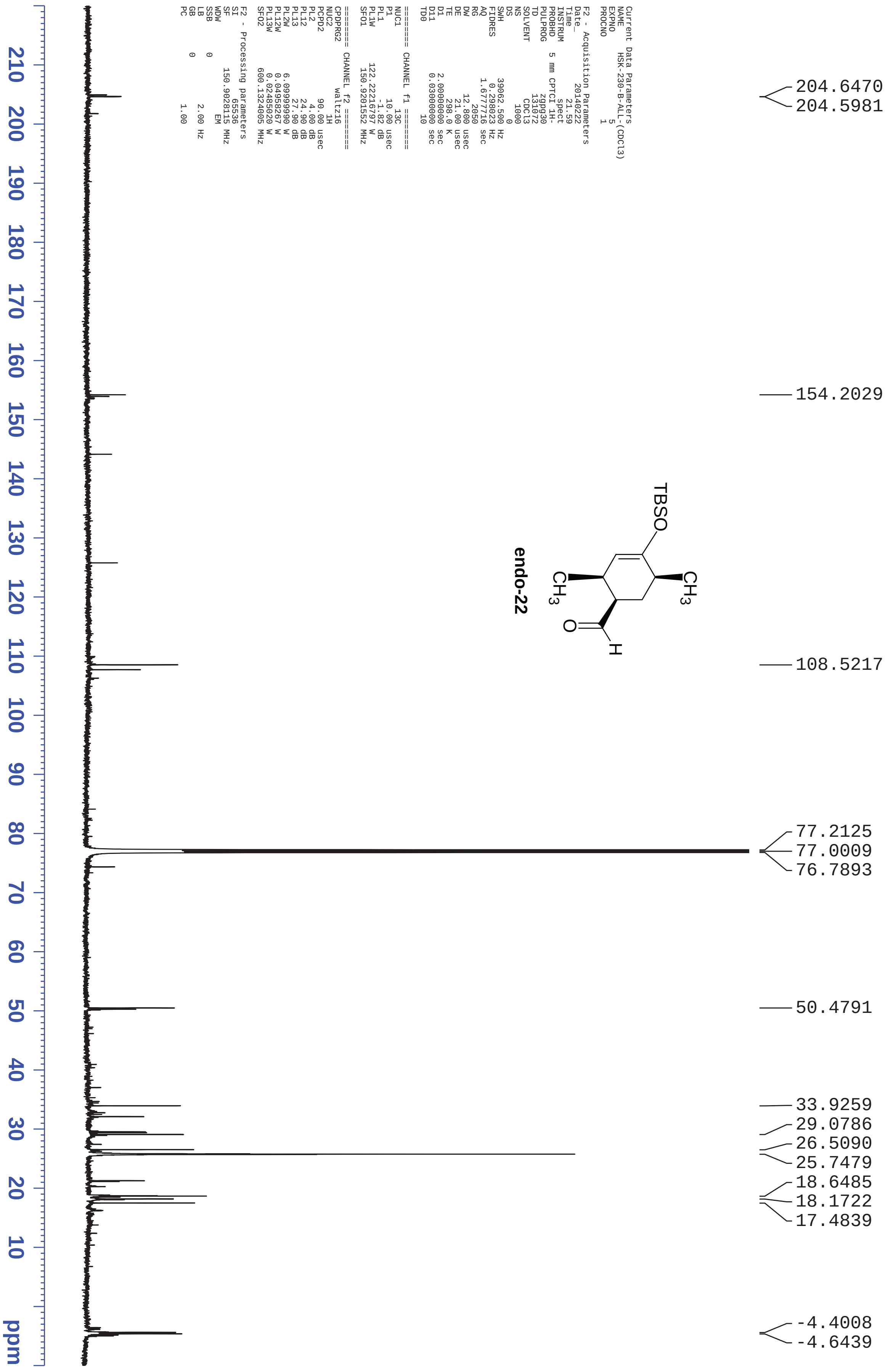

Supplementary Figure 47. **^{13}C NMR spectrum of compound endo-22.**

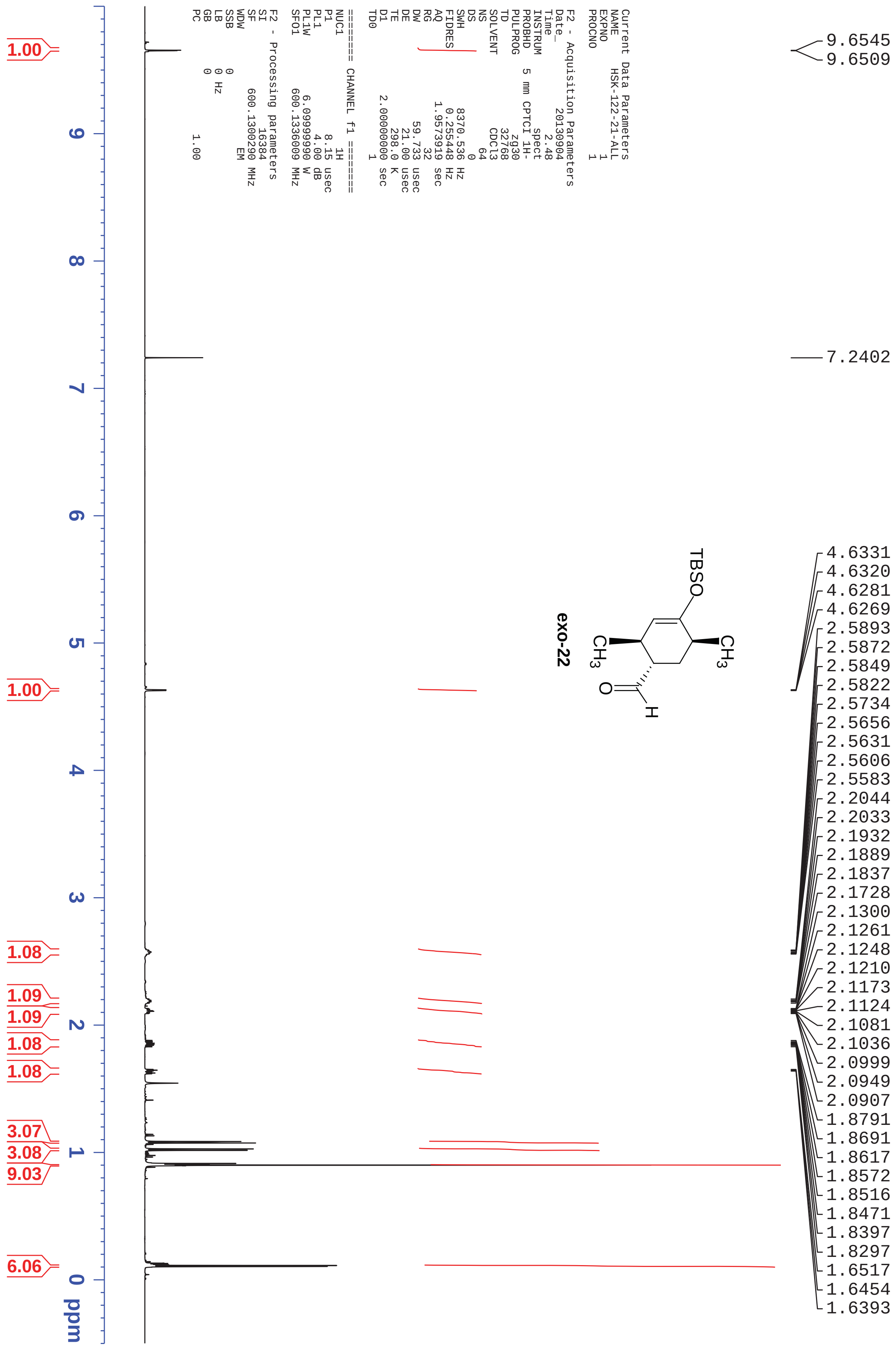

Supplementary Figure 48. **^{1}H NMR spectrum of compound exo-22.**

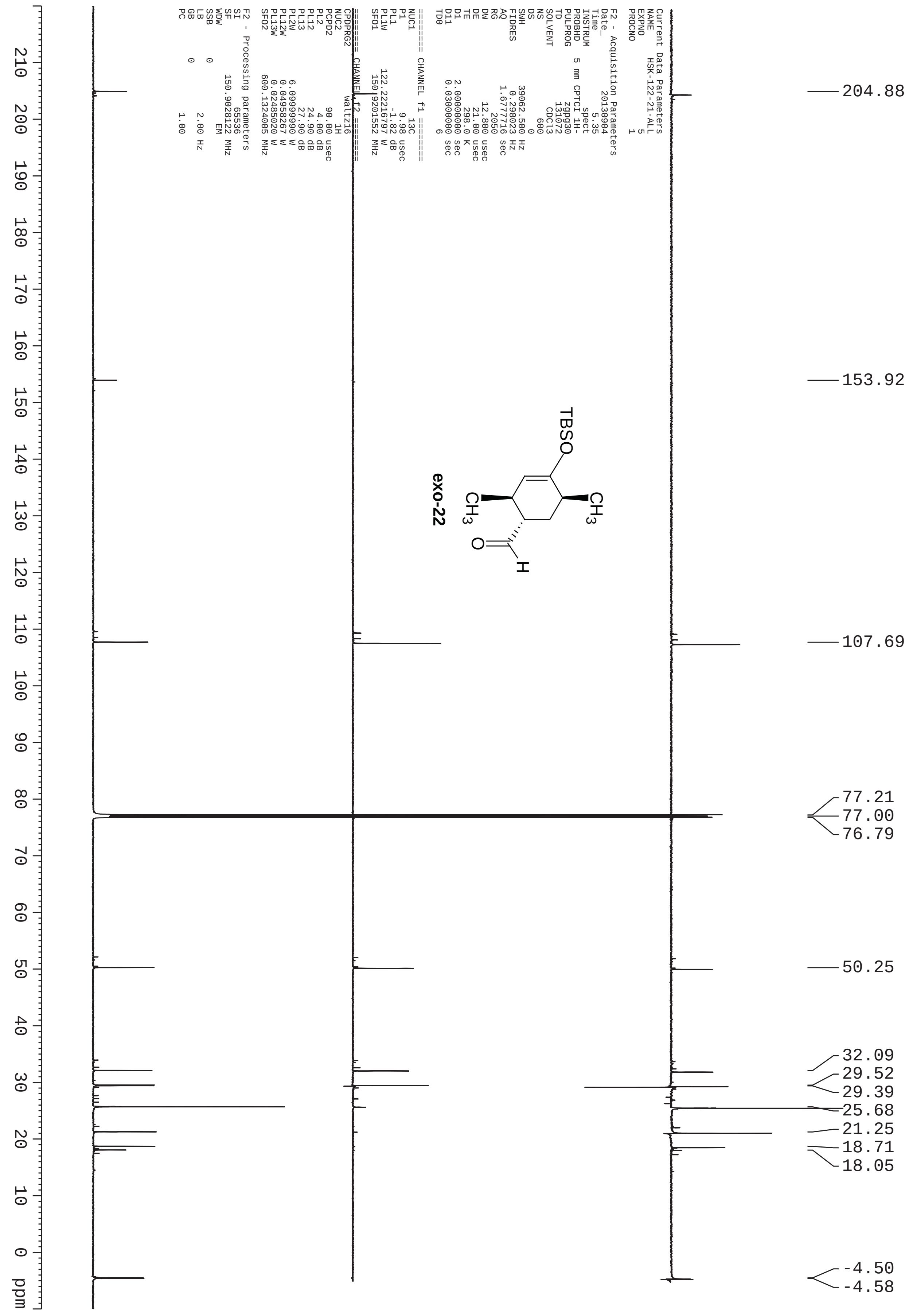

Supplementary Figure 49. ^{13}C and DEPT NMR spectra of compound exo-22.

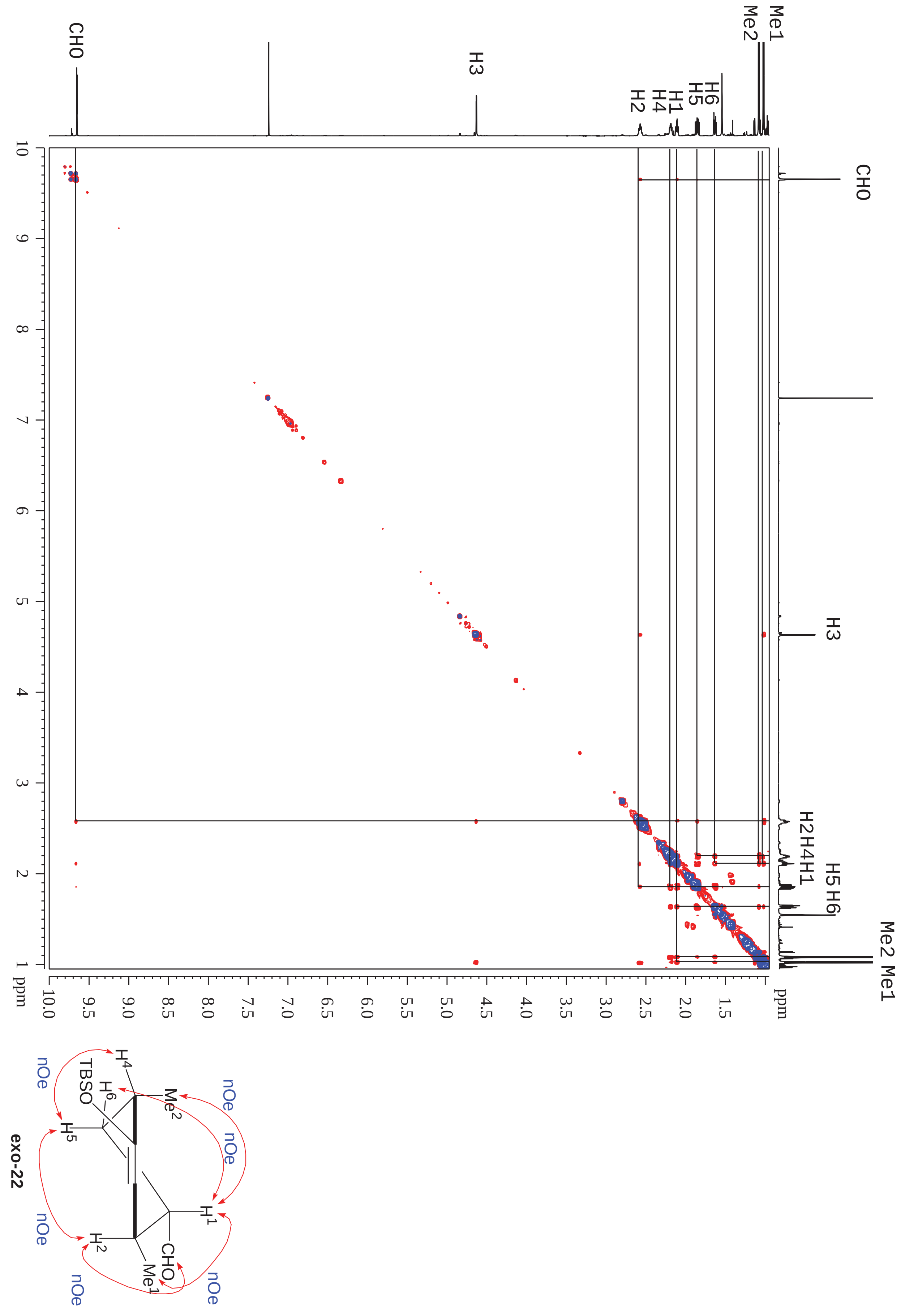

Supplementary Figure 50. NOESY NMR spectrum of compound exo-22.

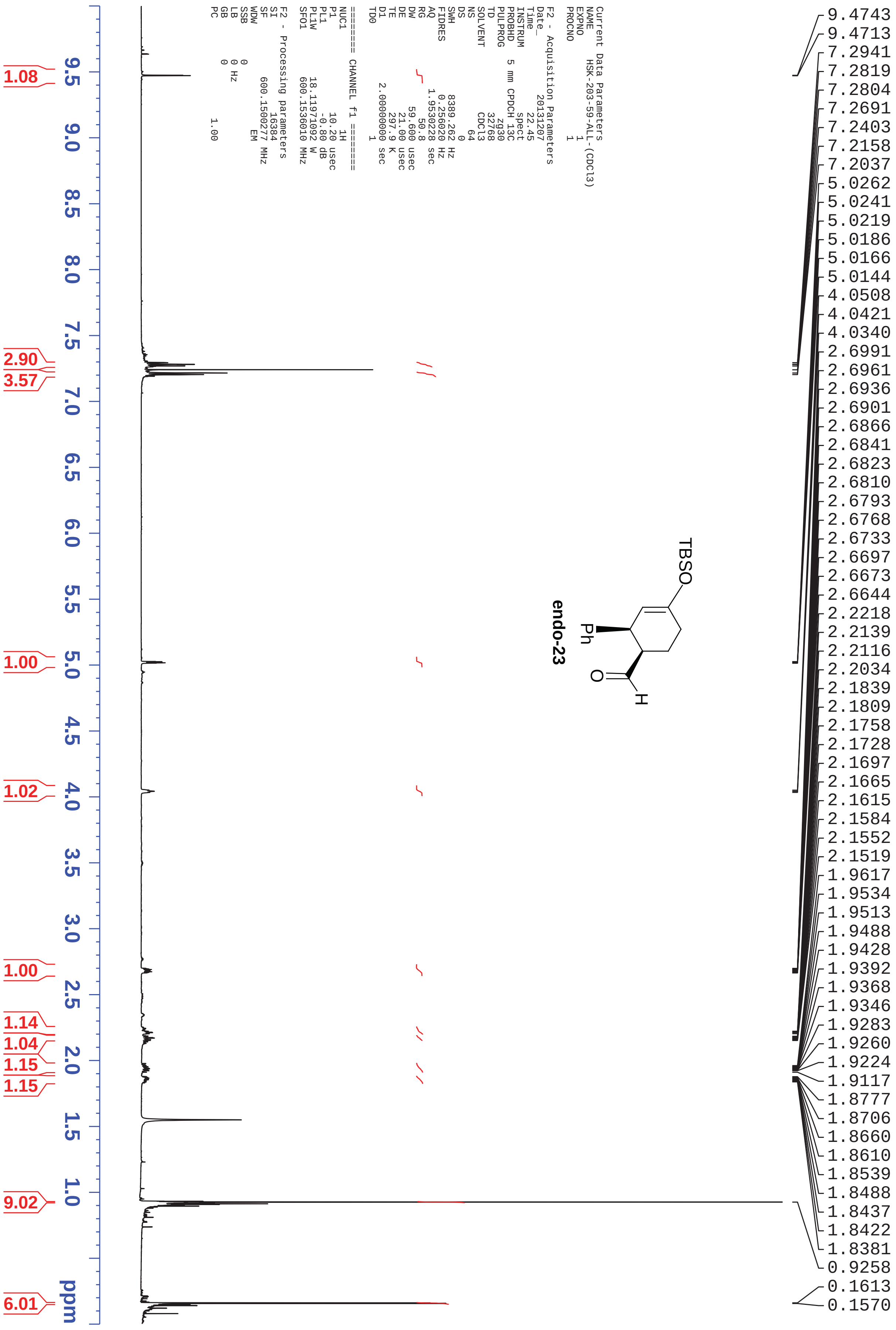

Supplementary Figure 51. **¹H NMR spectrum of compound endo-23.**

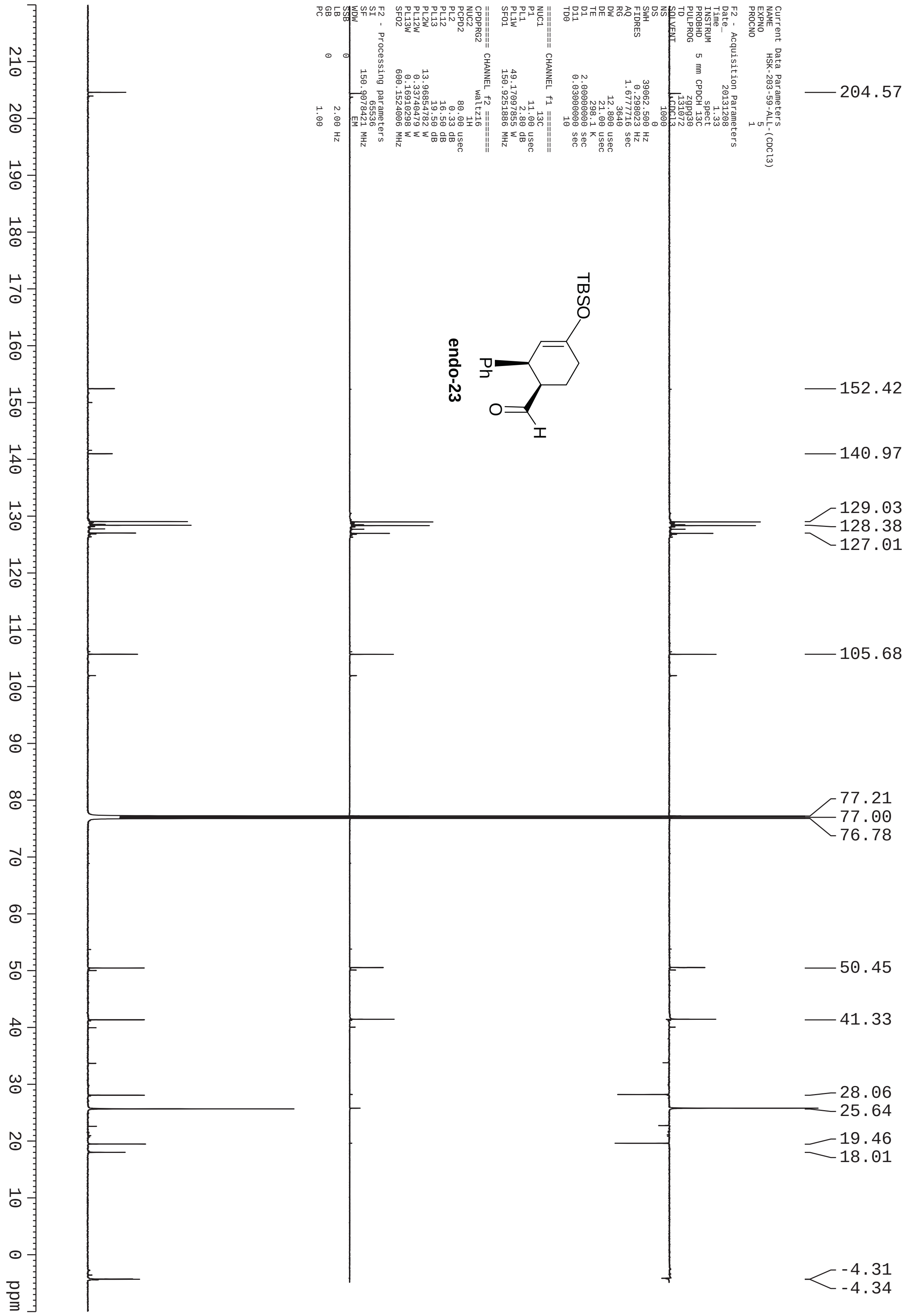

Supplementary Figure 52. ^{13}C and DEPT NMR spectra of compound endo-23.

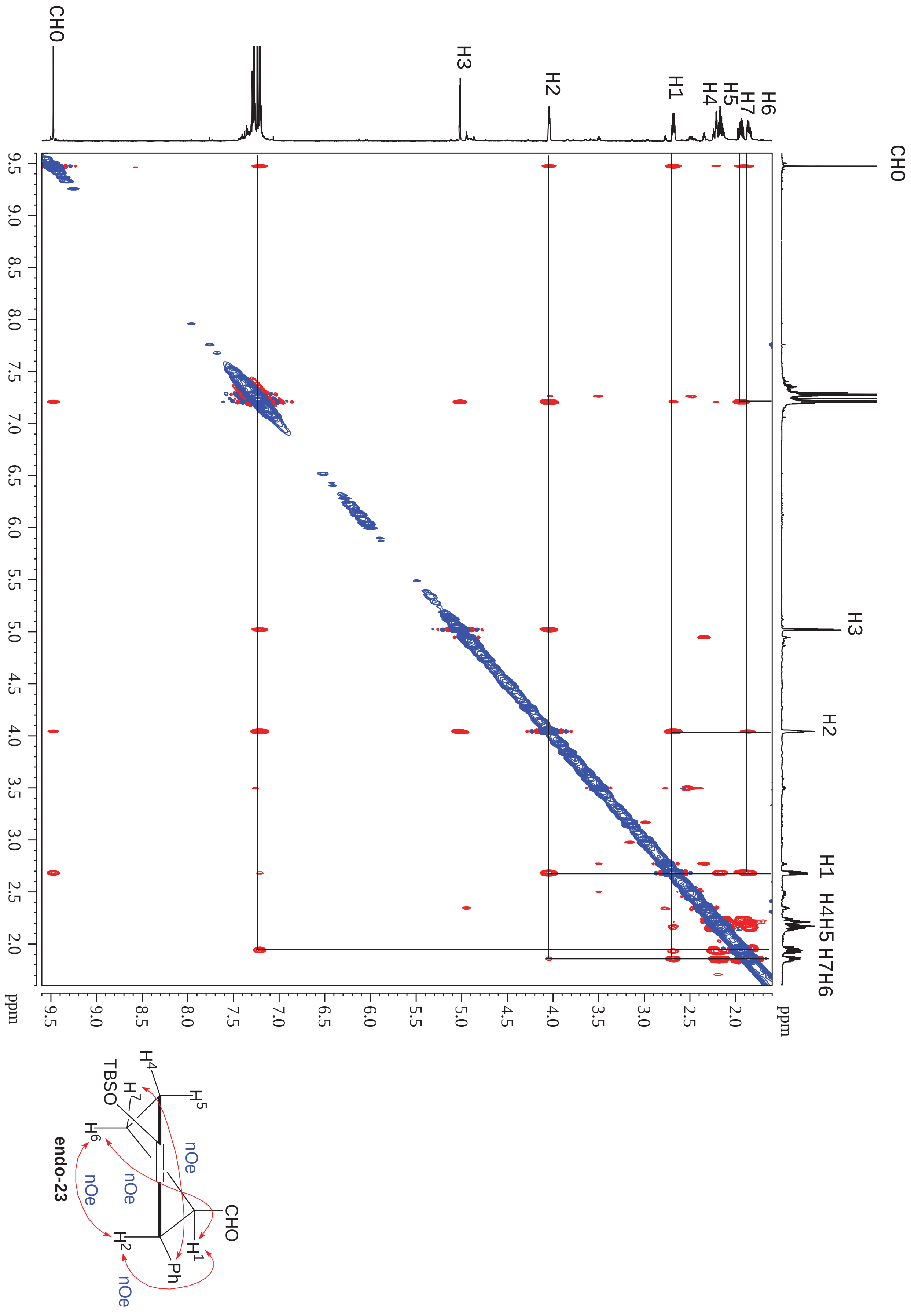

Supplementary Figure 53. NOESY NMR spectrum of compound endo-23.

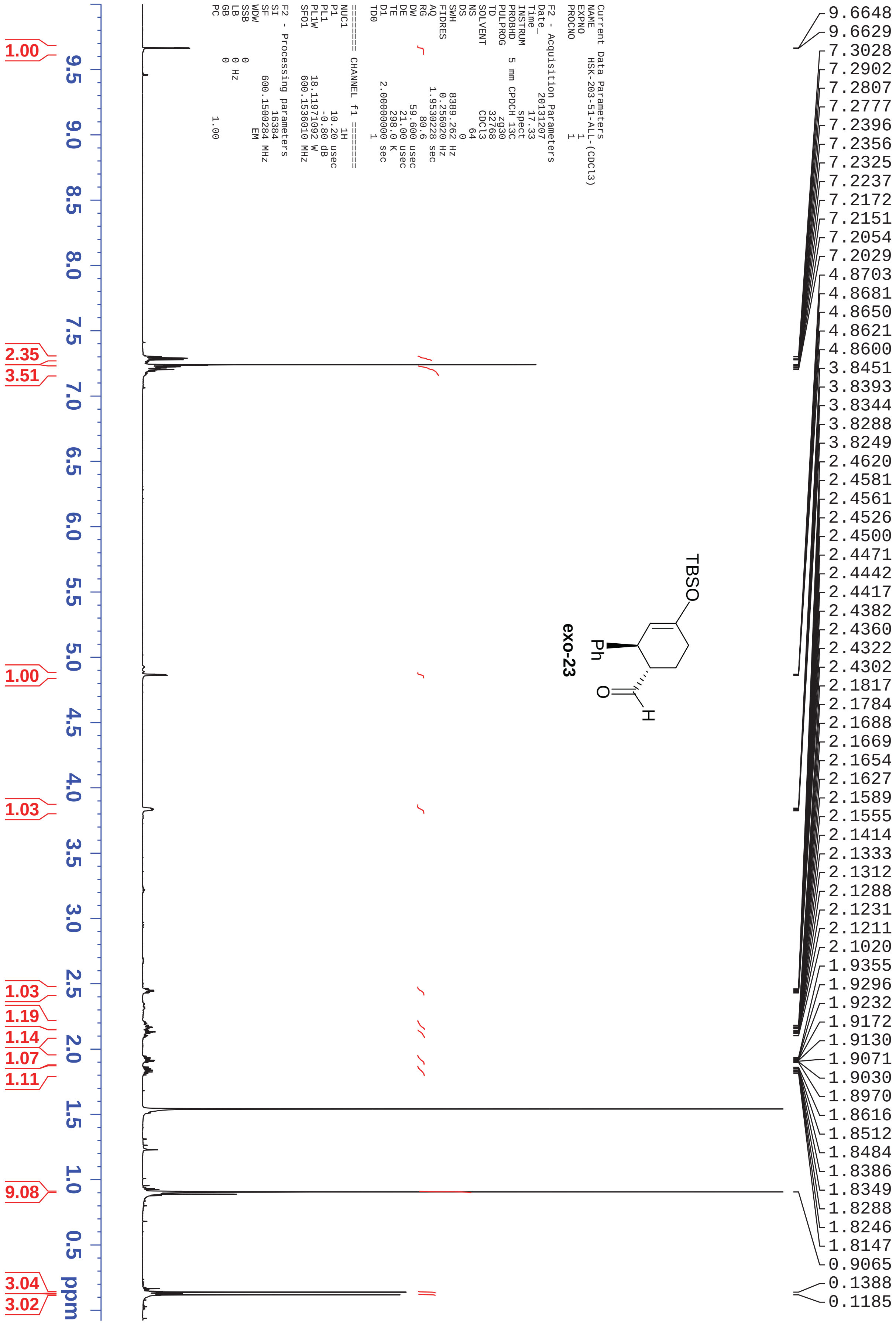

Supplementary Figure 54. **¹H NMR spectrum of compound exo-23.**

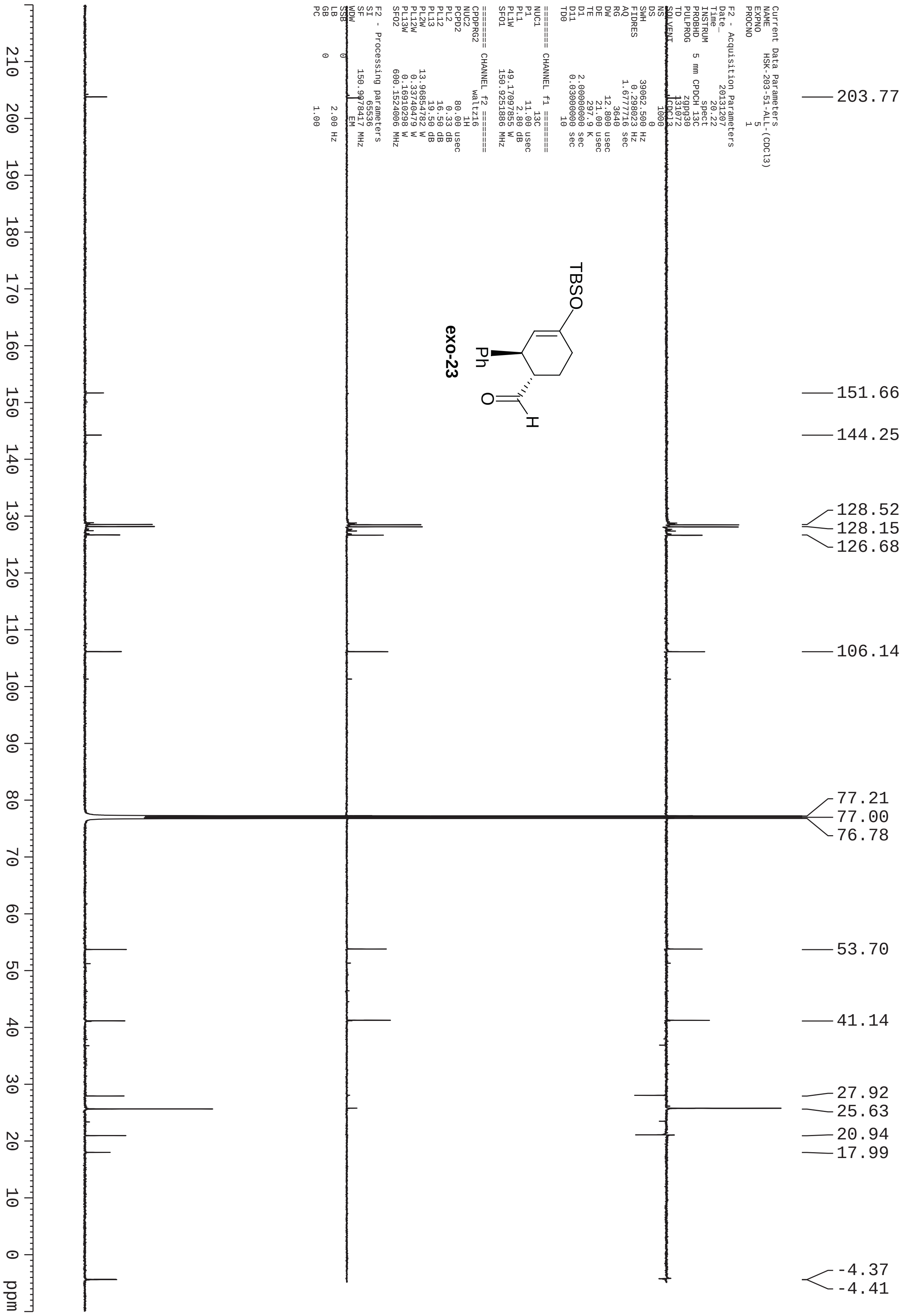

Supplementary Figure 55. ^{13}C and DEPT NMR spectra of compound exo-23.

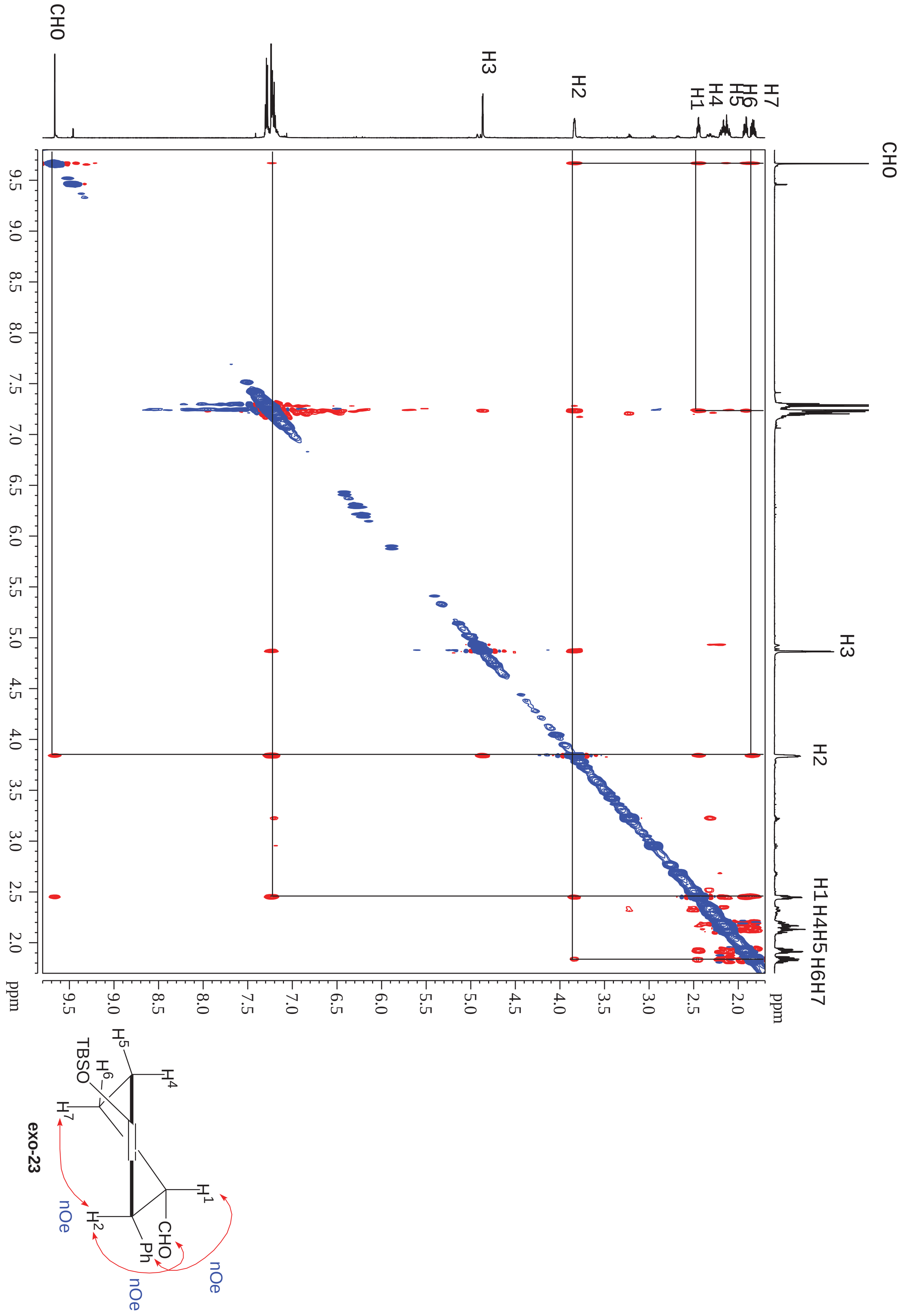

Supplementary Figure 56. NOESY NMR spectrum of compound exo-23.

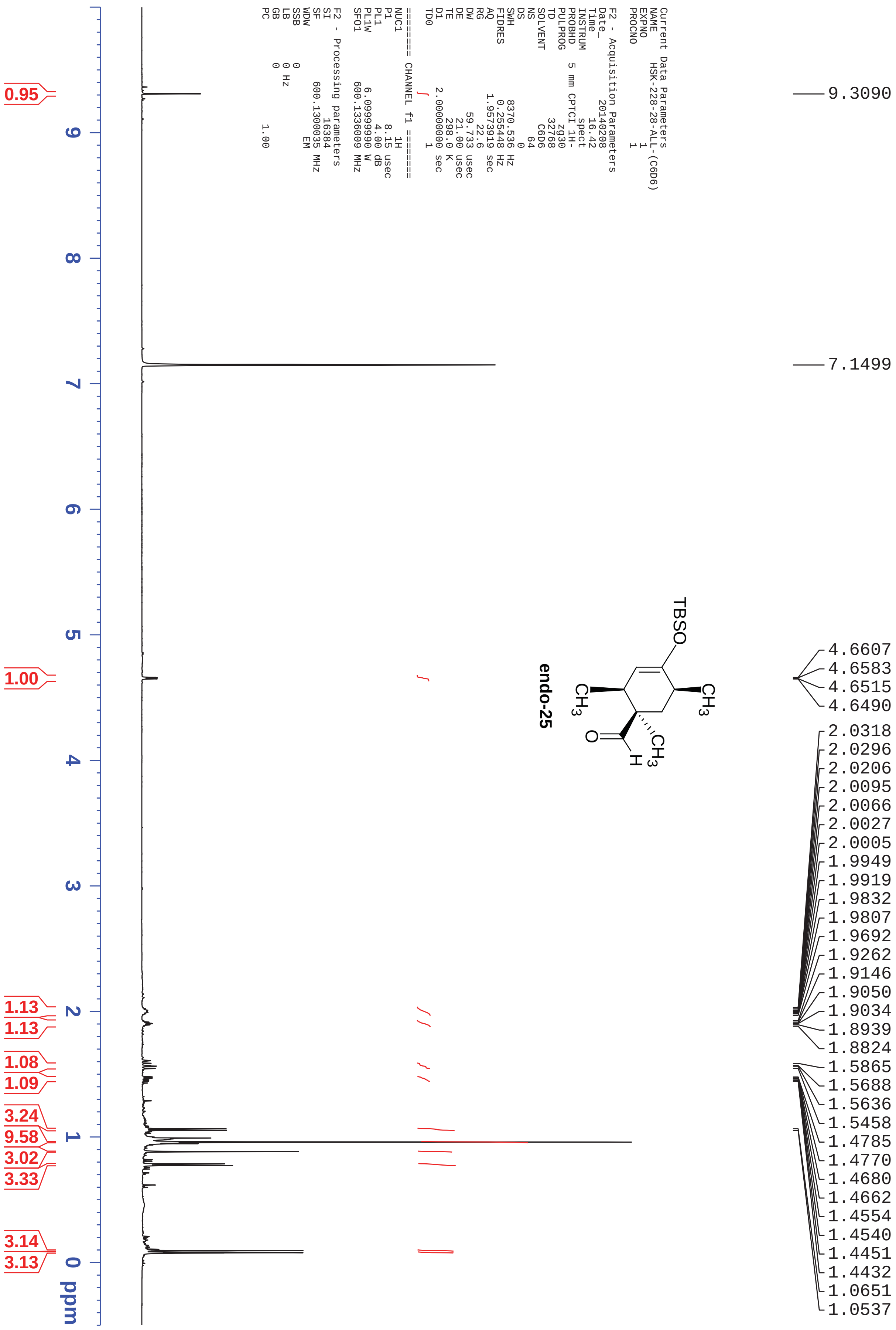

Supplementary Figure 57. ^{1}H NMR spectrum of compound endo-25.

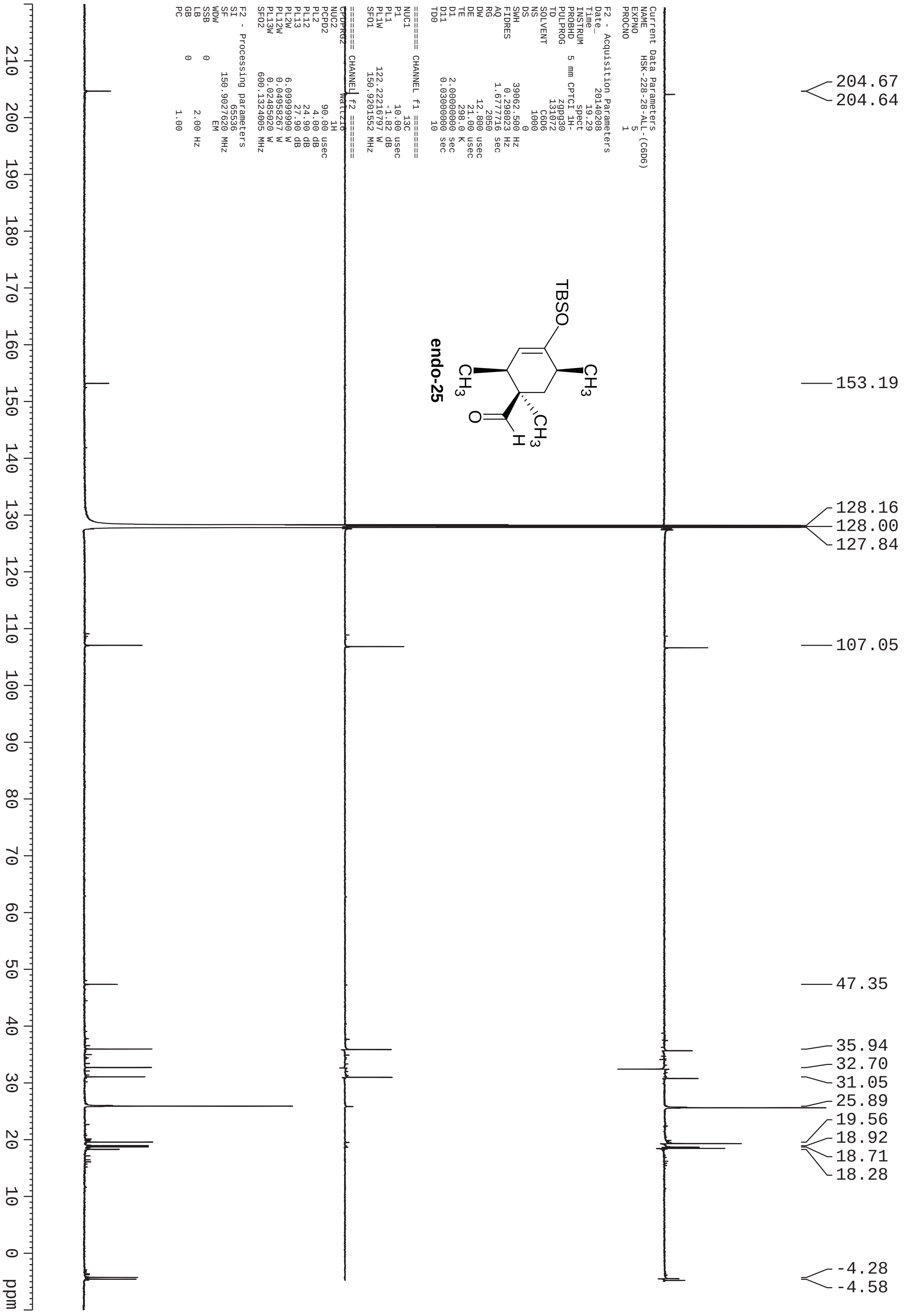

Supplementary Figure 58. ^{13}C and DEPT NMR spectra of compound endo-25.

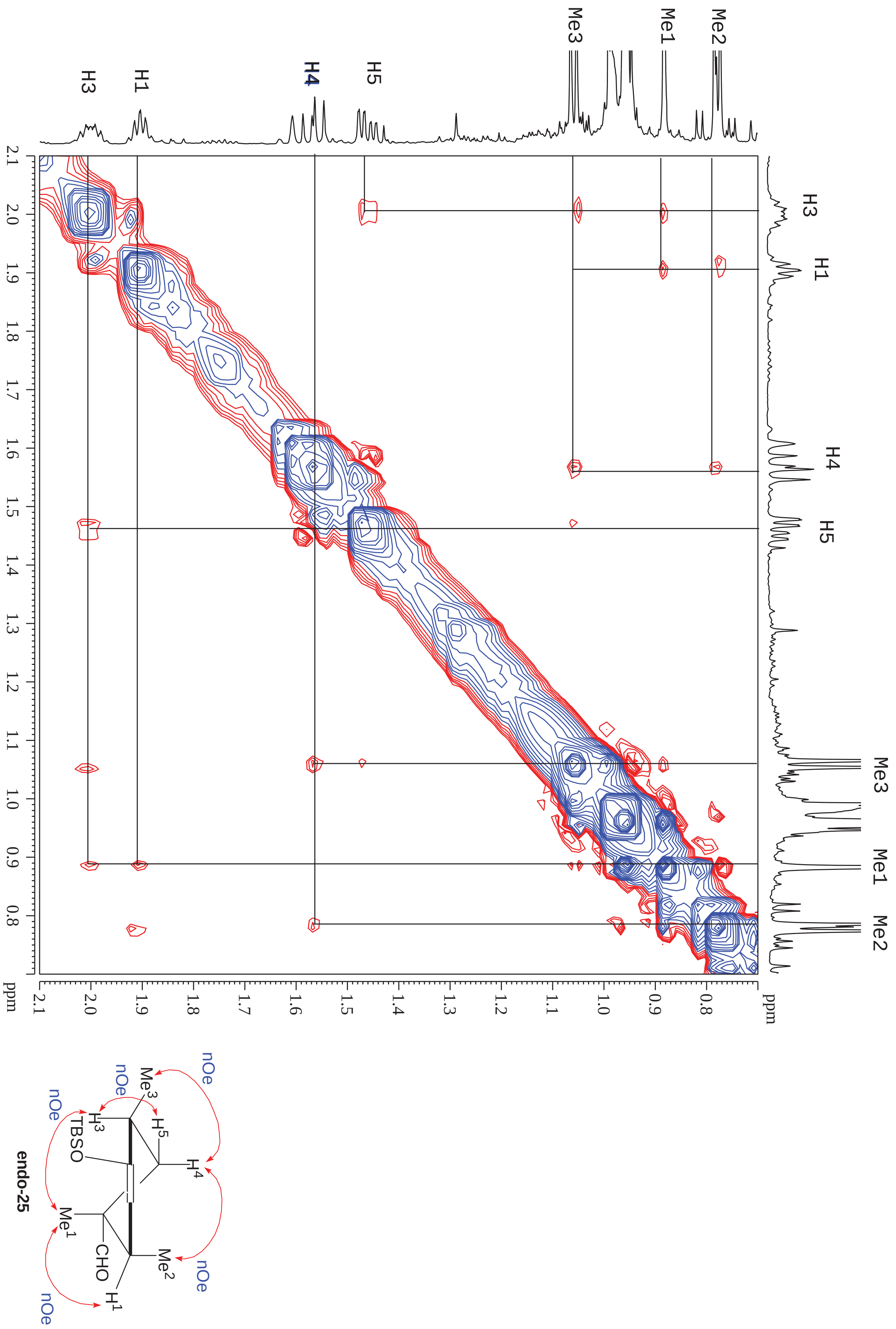

Supplementary Figure 59. NOESY NMR spectrum of compound endo-25.

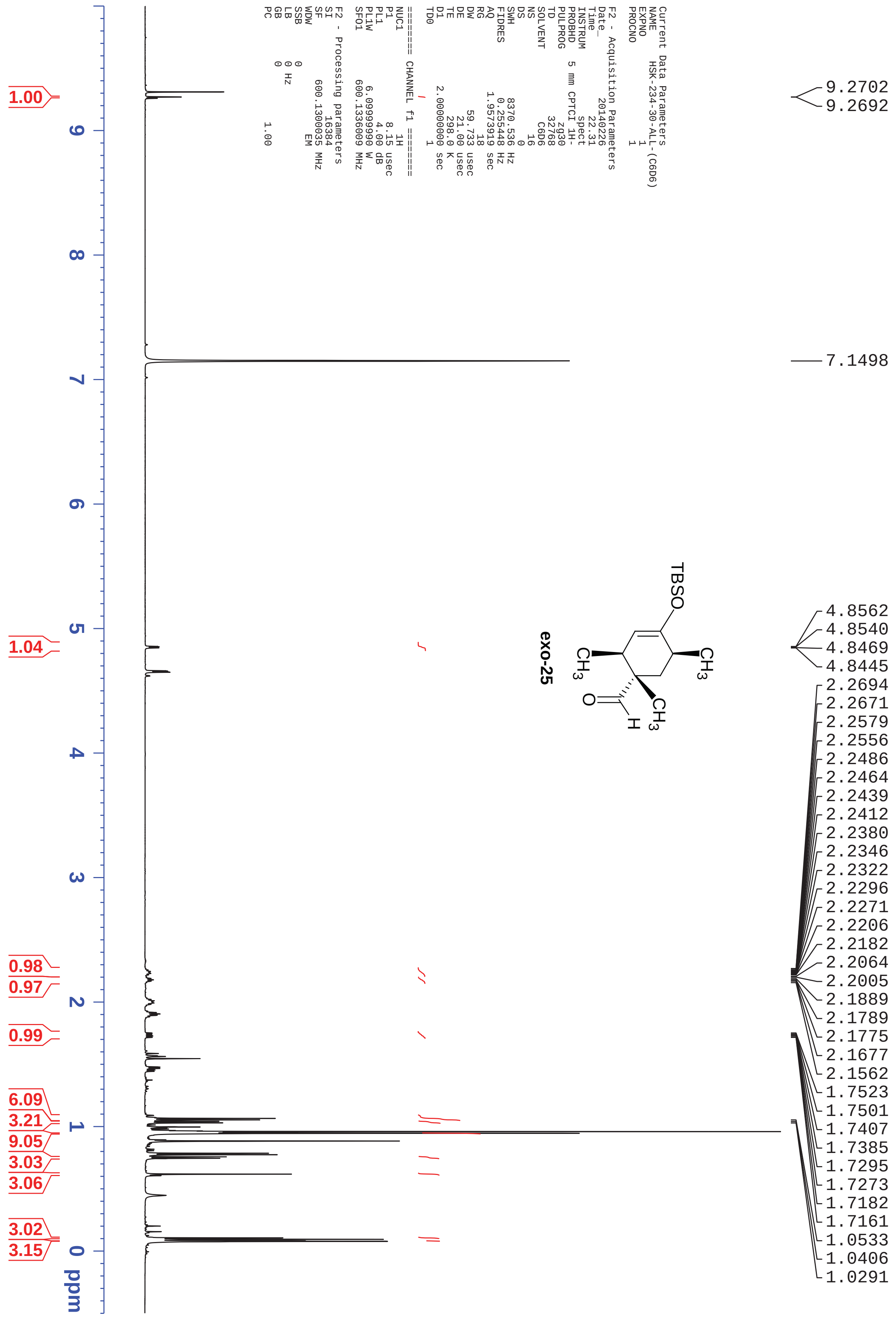

Supplementary Figure 60. ¹H NMR spectrum of compound exo-25.

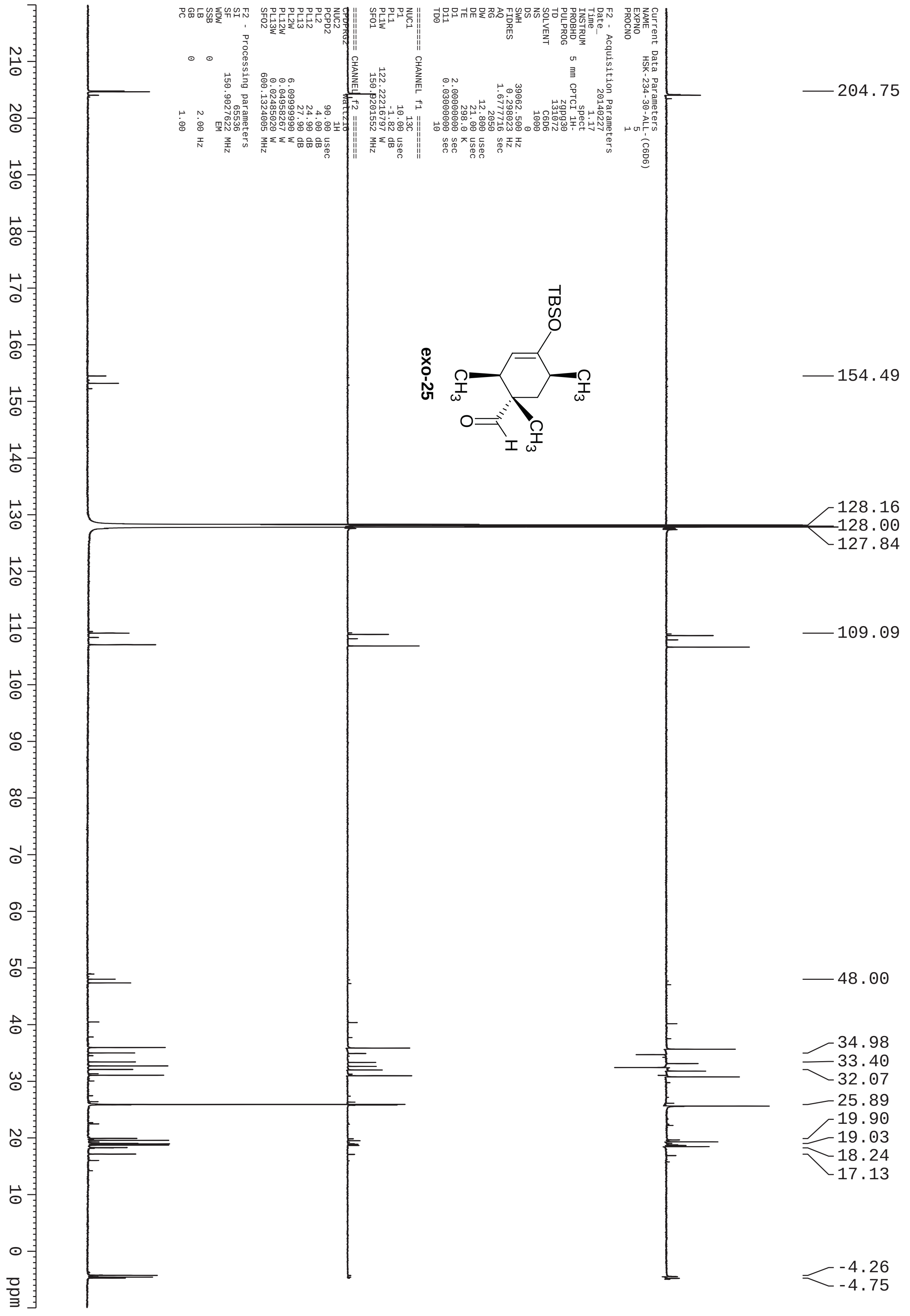

Supplementary Figure 61. ^{13}C and DEPT NMR spectra of compound exo-25.

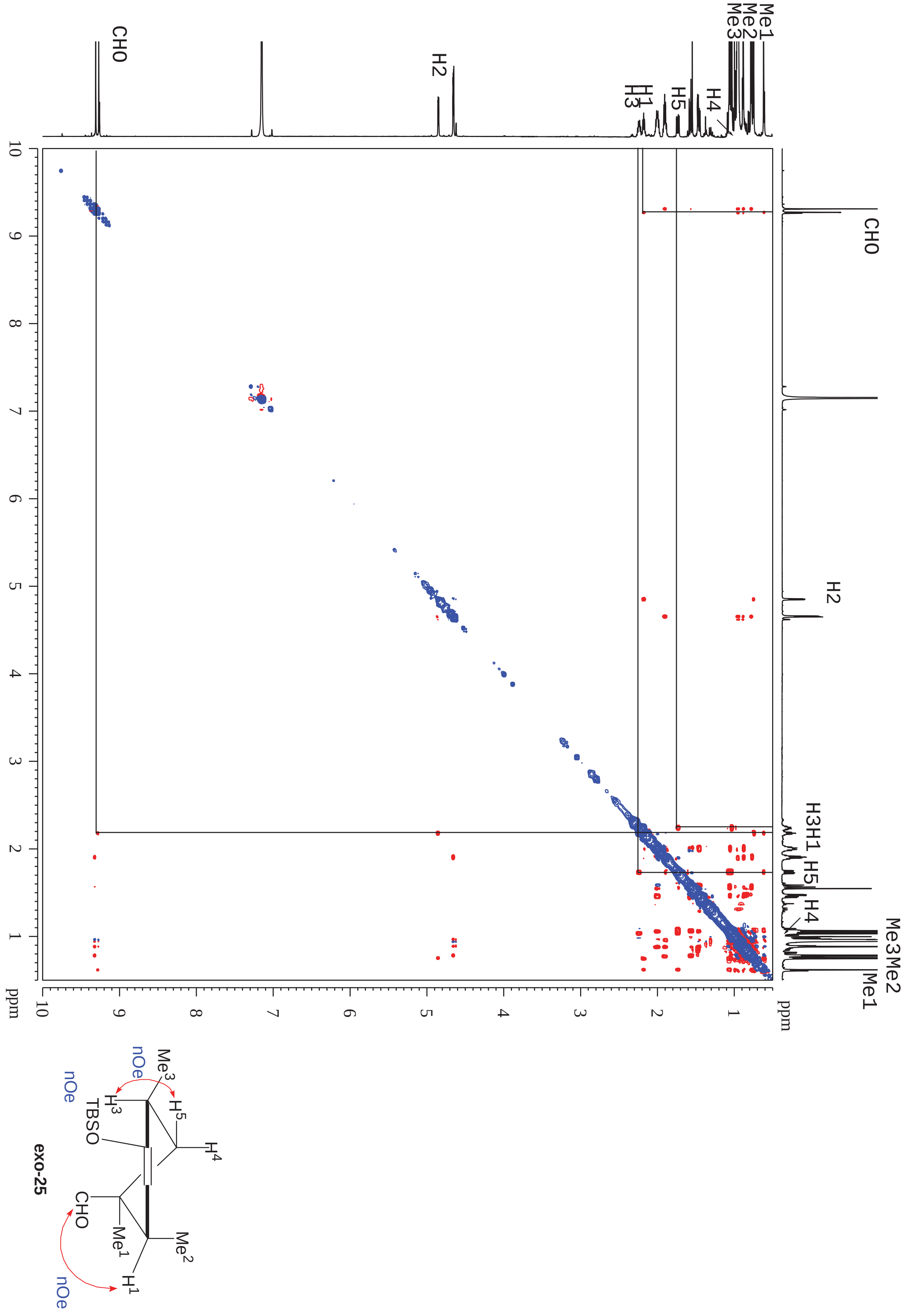

Supplementary Figure 62. NOESY NMR spectrum of compound exo-25.

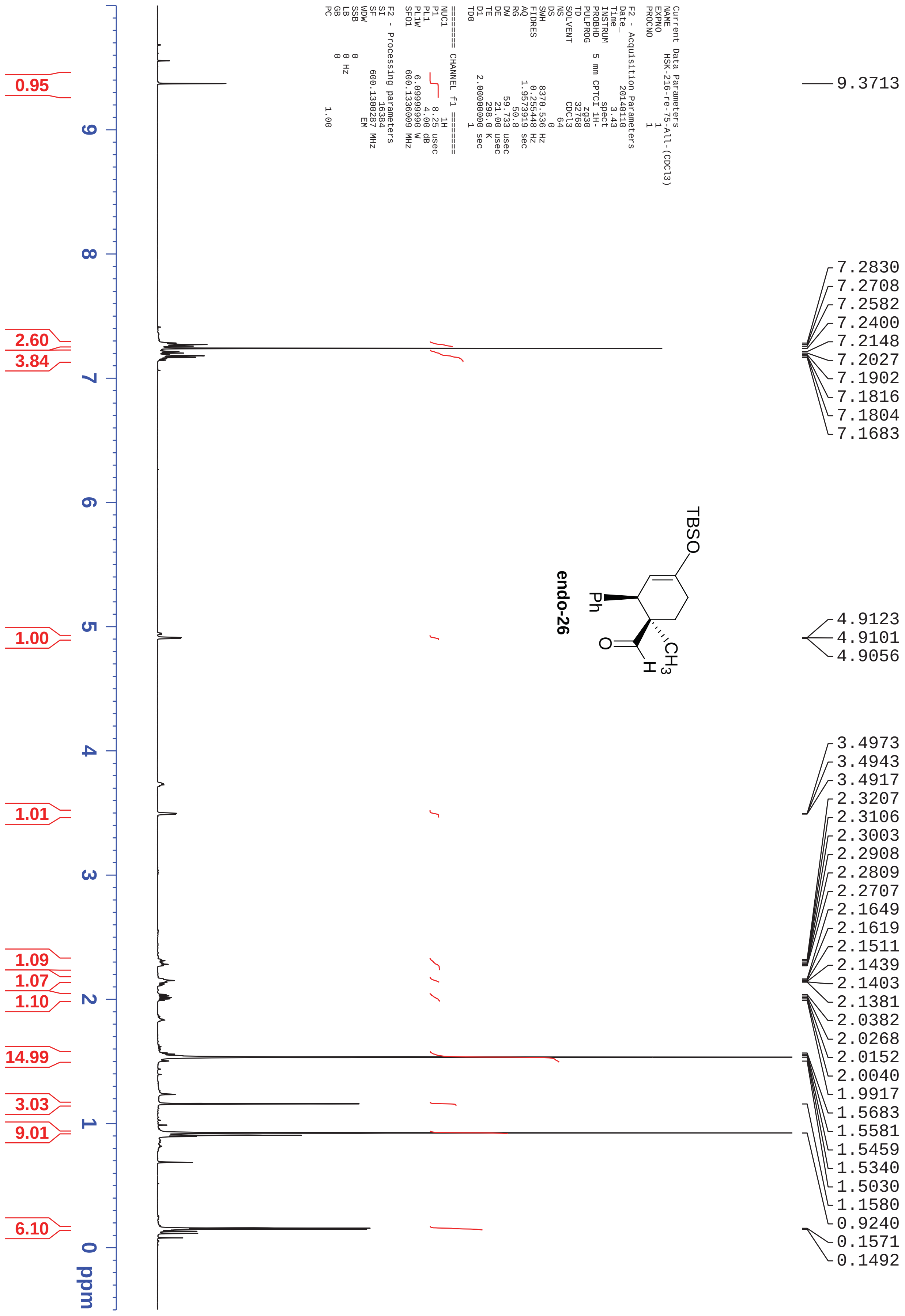

Supplementary Figure 63. ^{1}H NMR spectrum of compound endo-26.

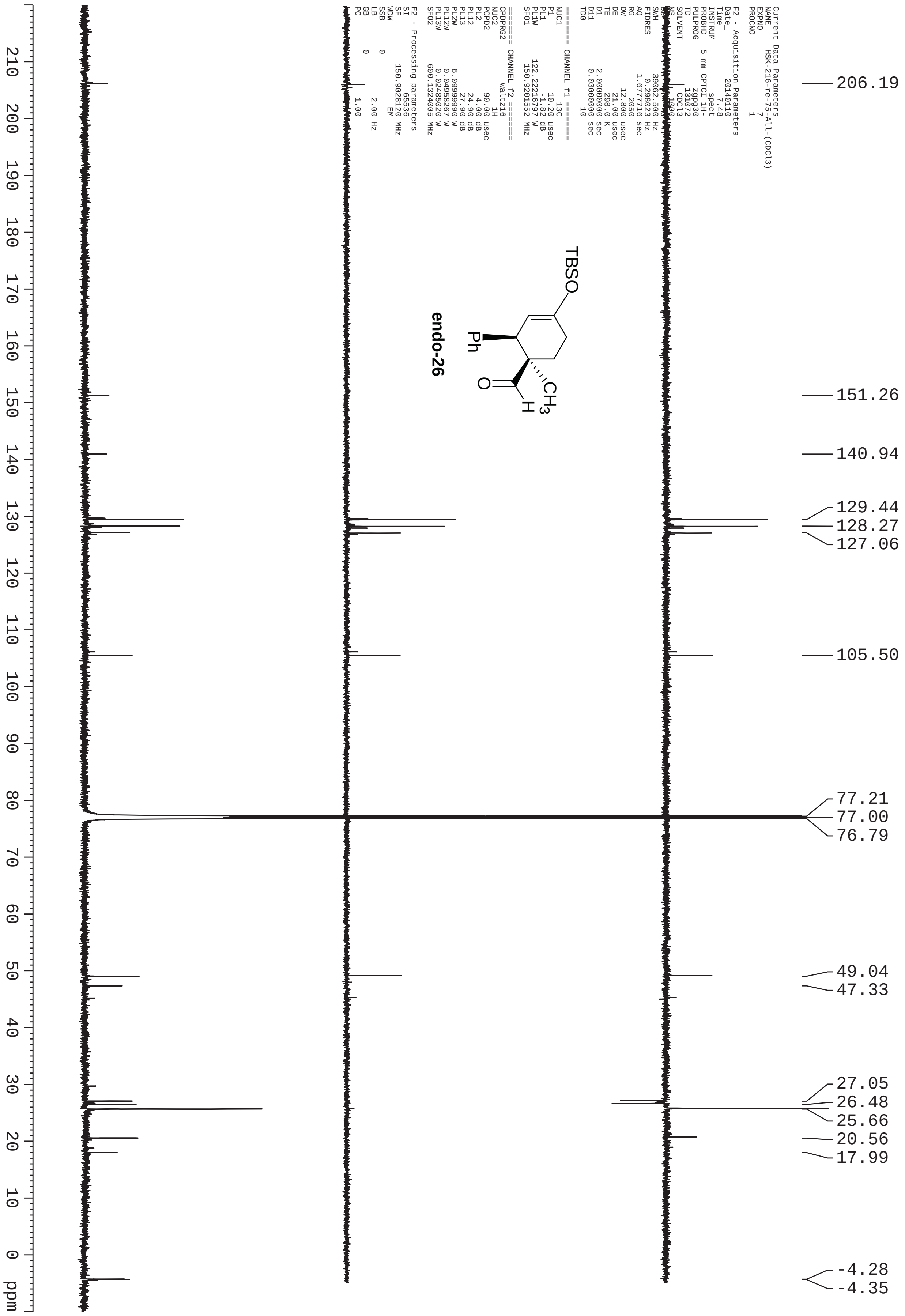

Supplementary Figure 64. ^{13}C and DEPT NMR spectra of compound endo-26.

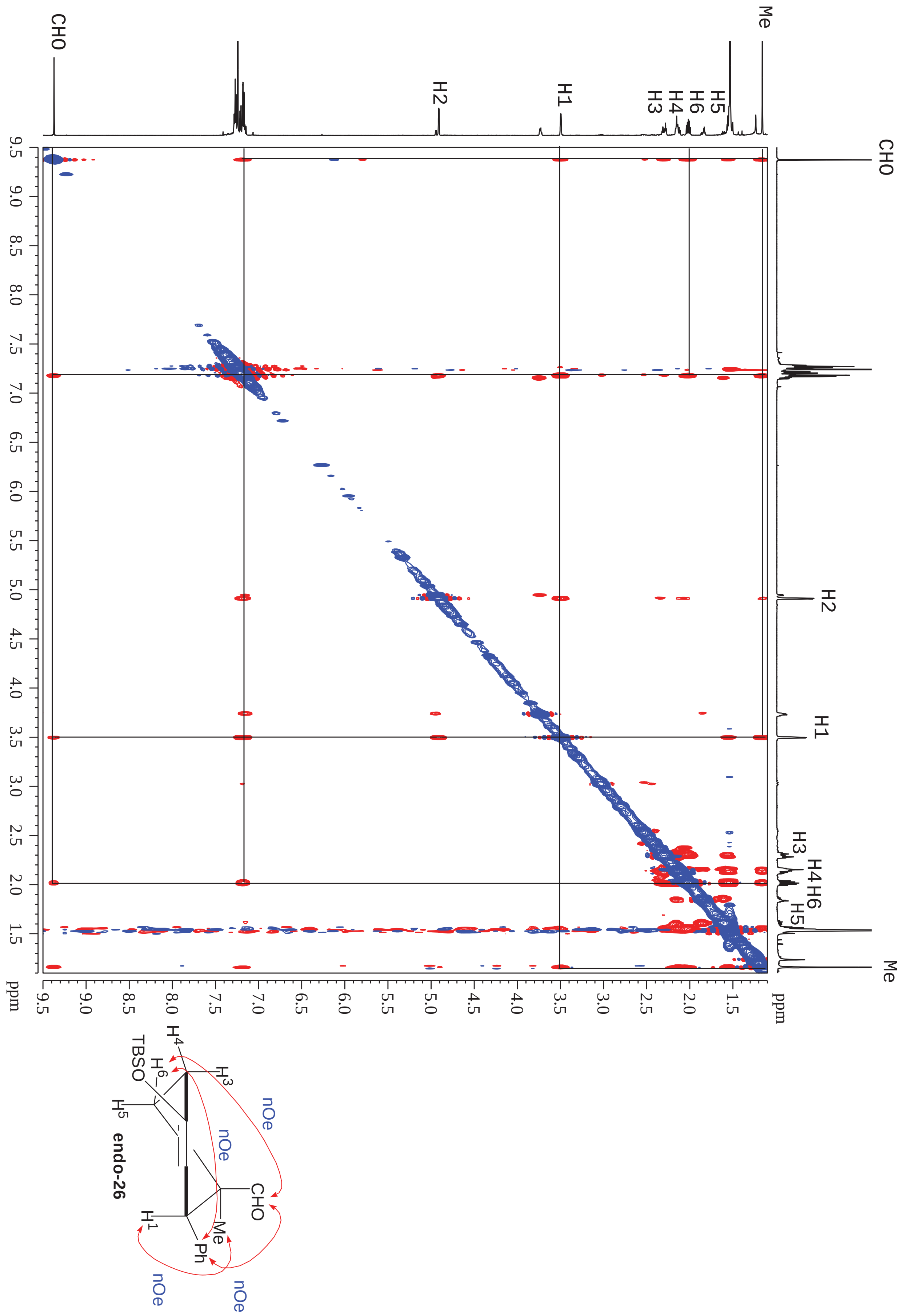

Supplementary Figure 65. NOESY NMR spectrum of compound endo-26.

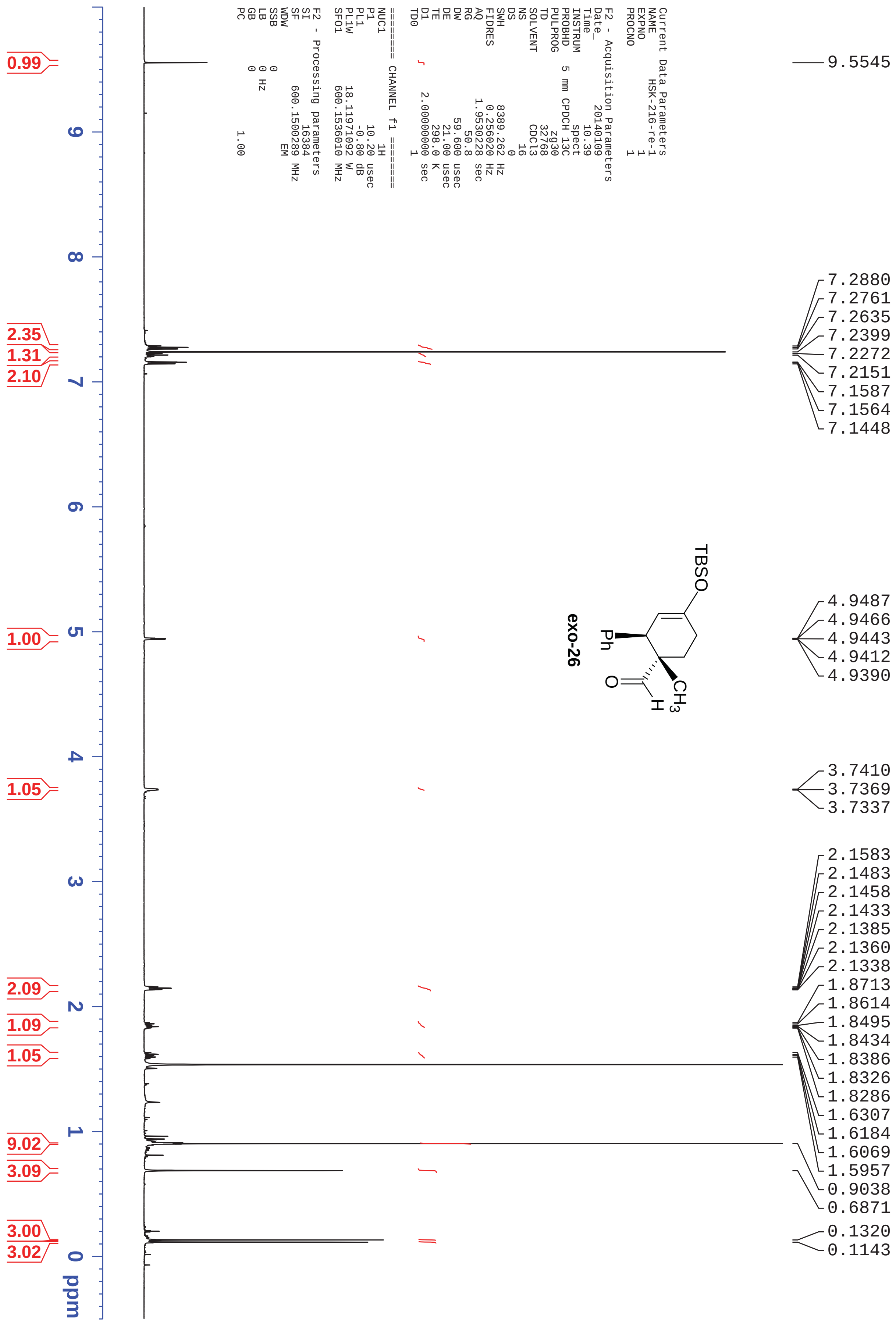

Supplementary Figure 66. ^{1}H NMR spectrum of compound exo-26.

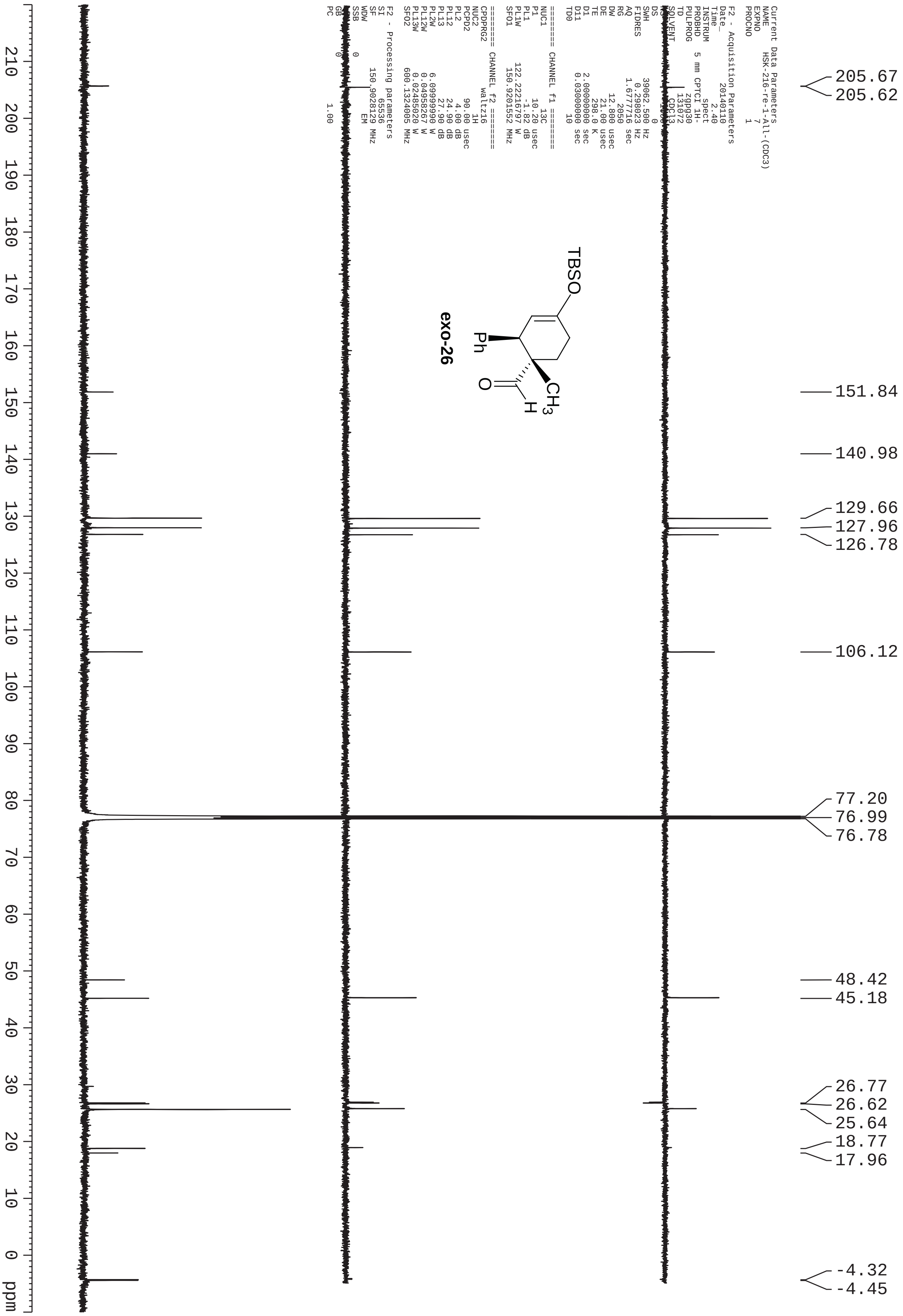

Supplementary Figure 67. ^{13}C and DEPT NMR spectra of compound exo-26.

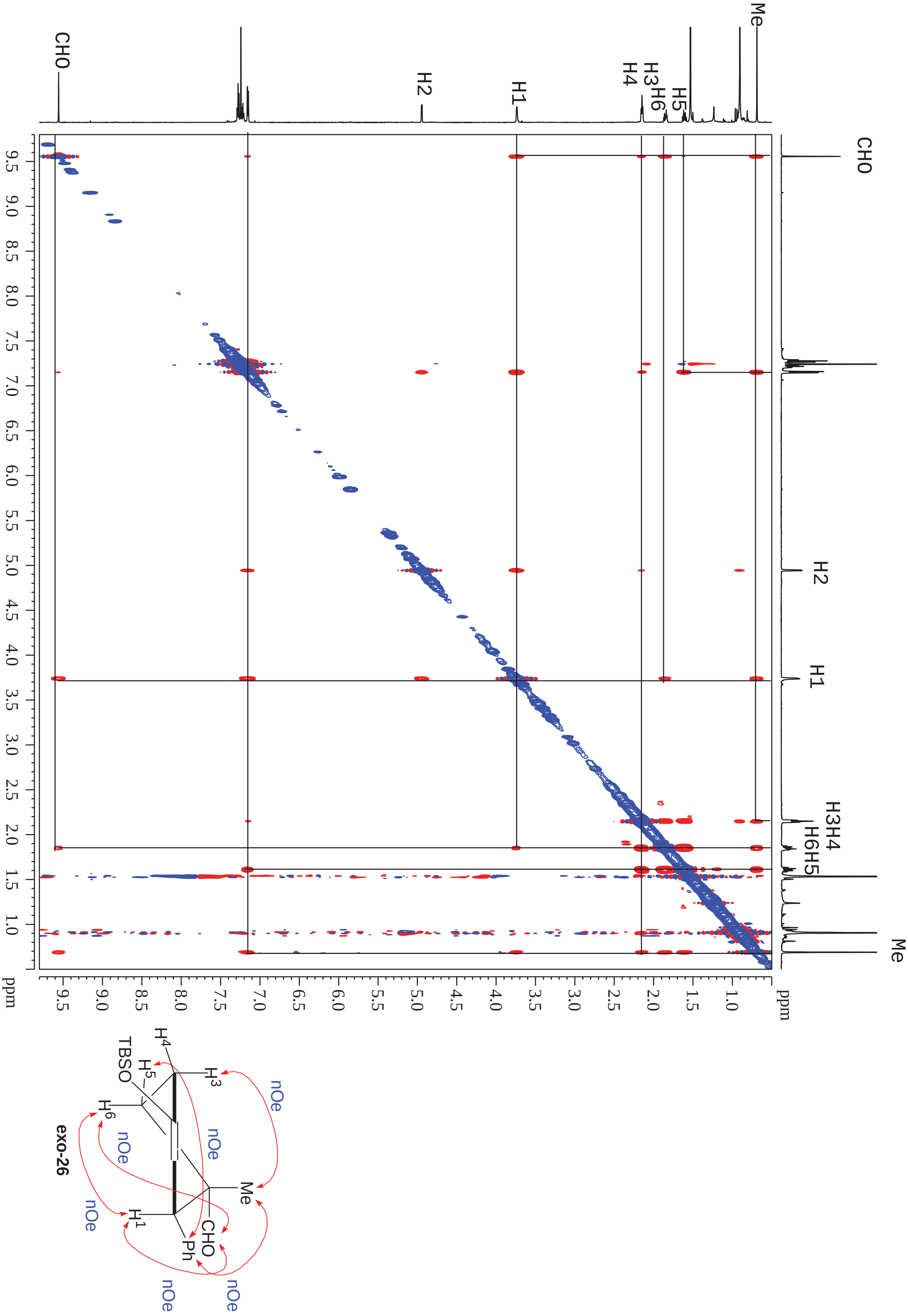

Supplementary Figure 68. NOESY NMR spectrum of compound exo-26.

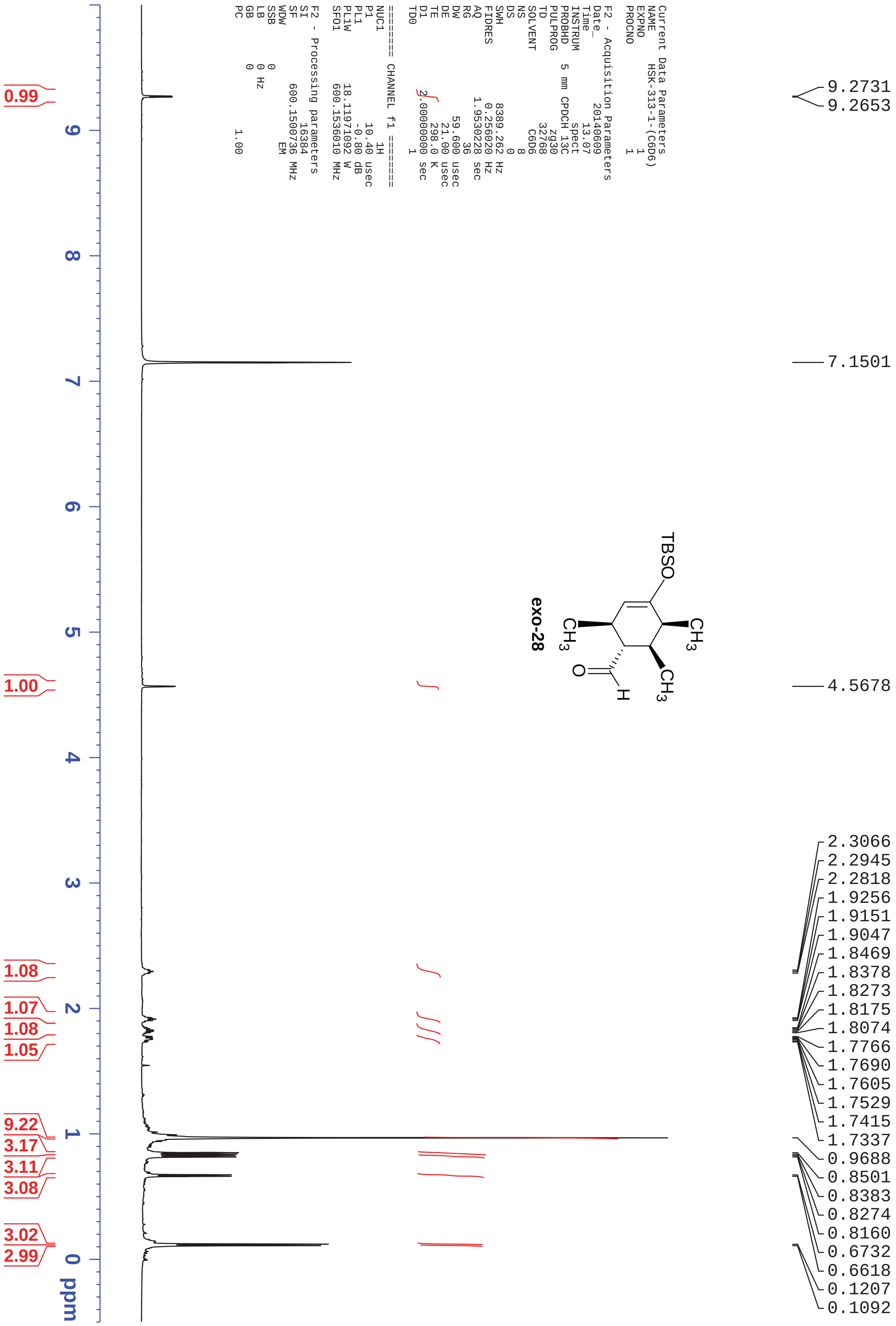

Supplementary Figure 69. ^{1}H NMR spectrum of compound exo-28.

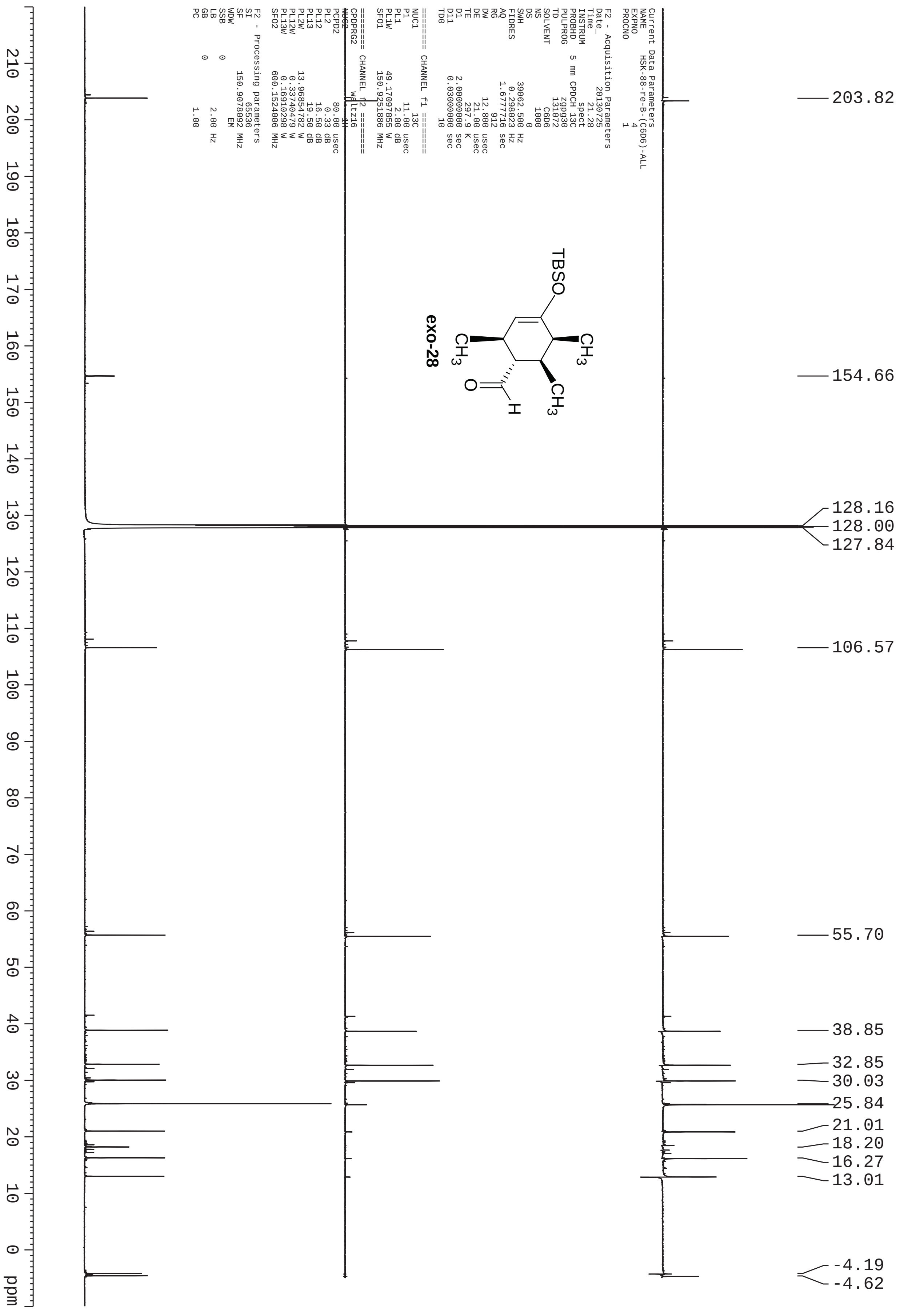

Supplementary Figure 70. ^{13}C and DEPT NMR spectra of compound exo-28.

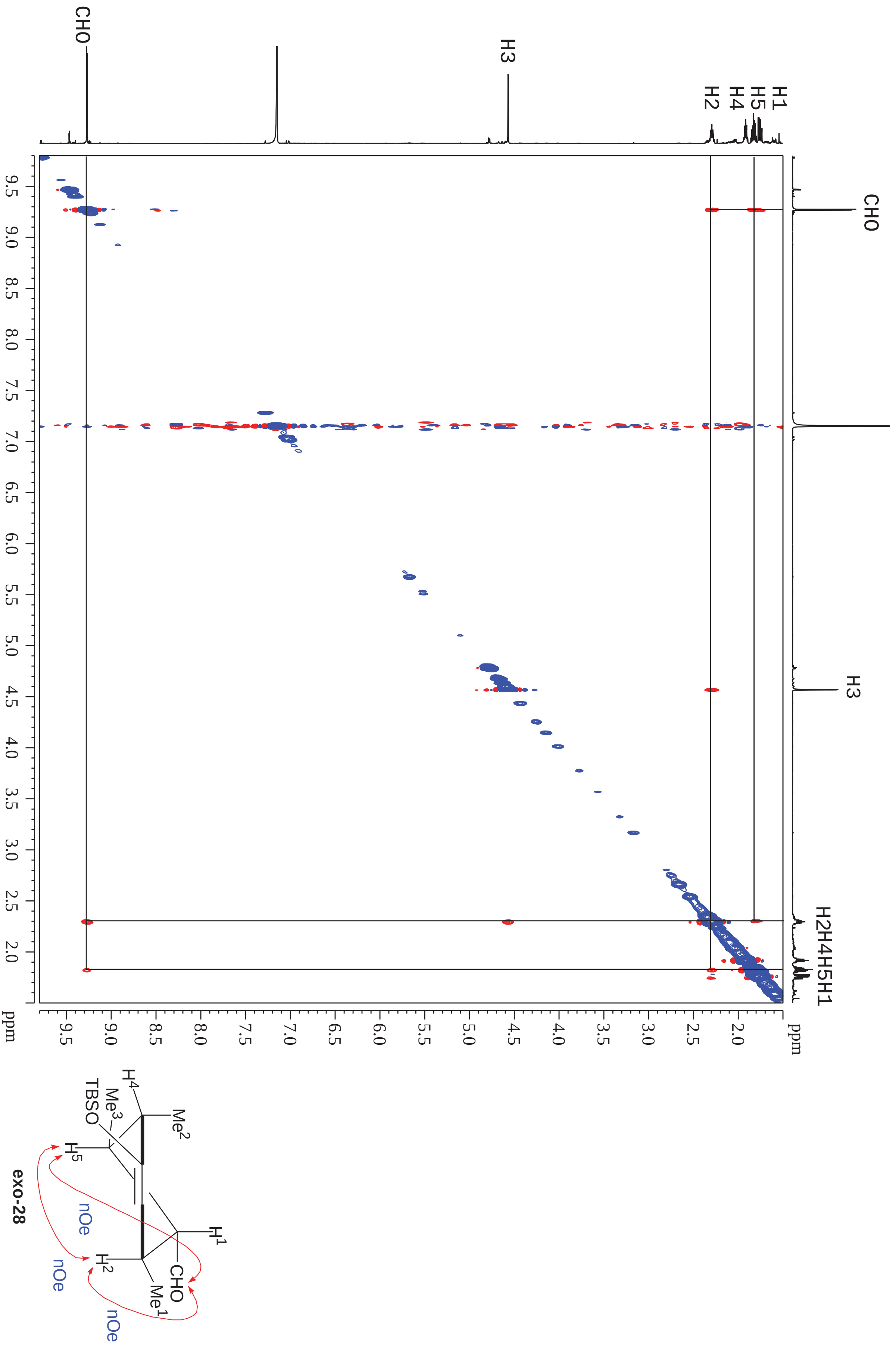

Supplementary Figure 71. NOESY NMR spectrum of compound exo-28.

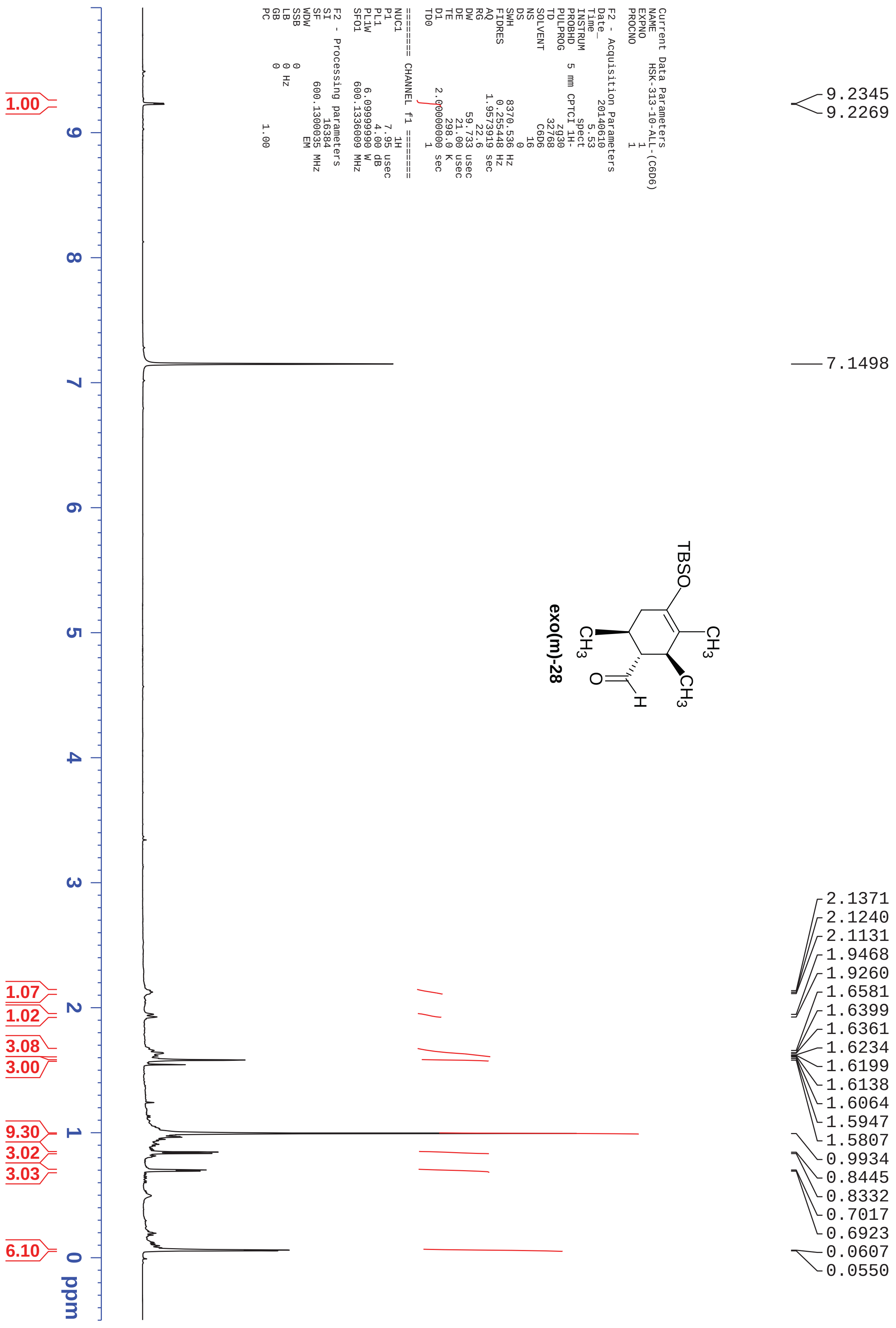

Supplementary Figure 72. **^{1}H NMR spectrum of compound exo(m)-28.**

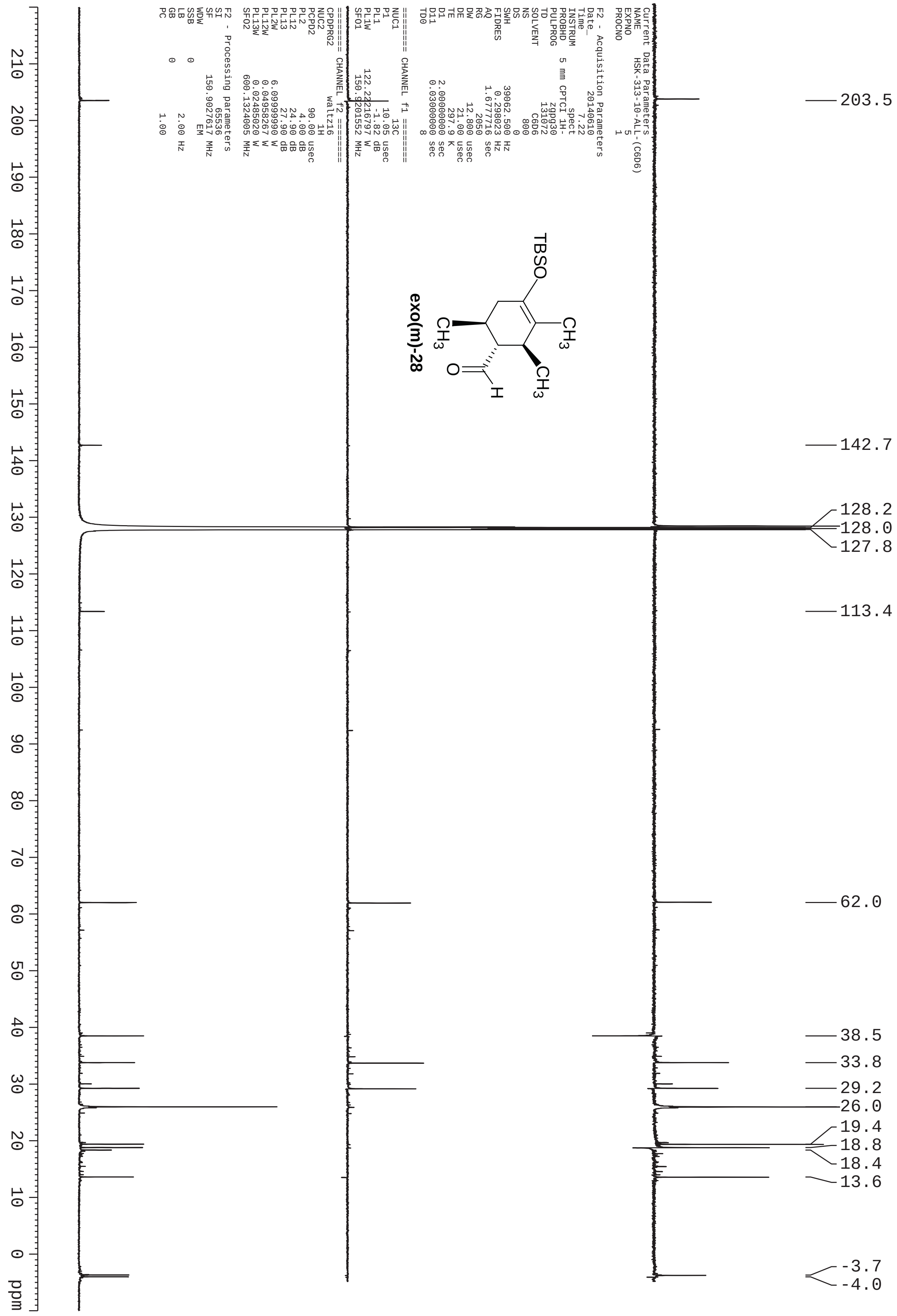

Supplementary Figure 73. ^{13}C and DEPT NMR spectra of compound exo(m)-28.

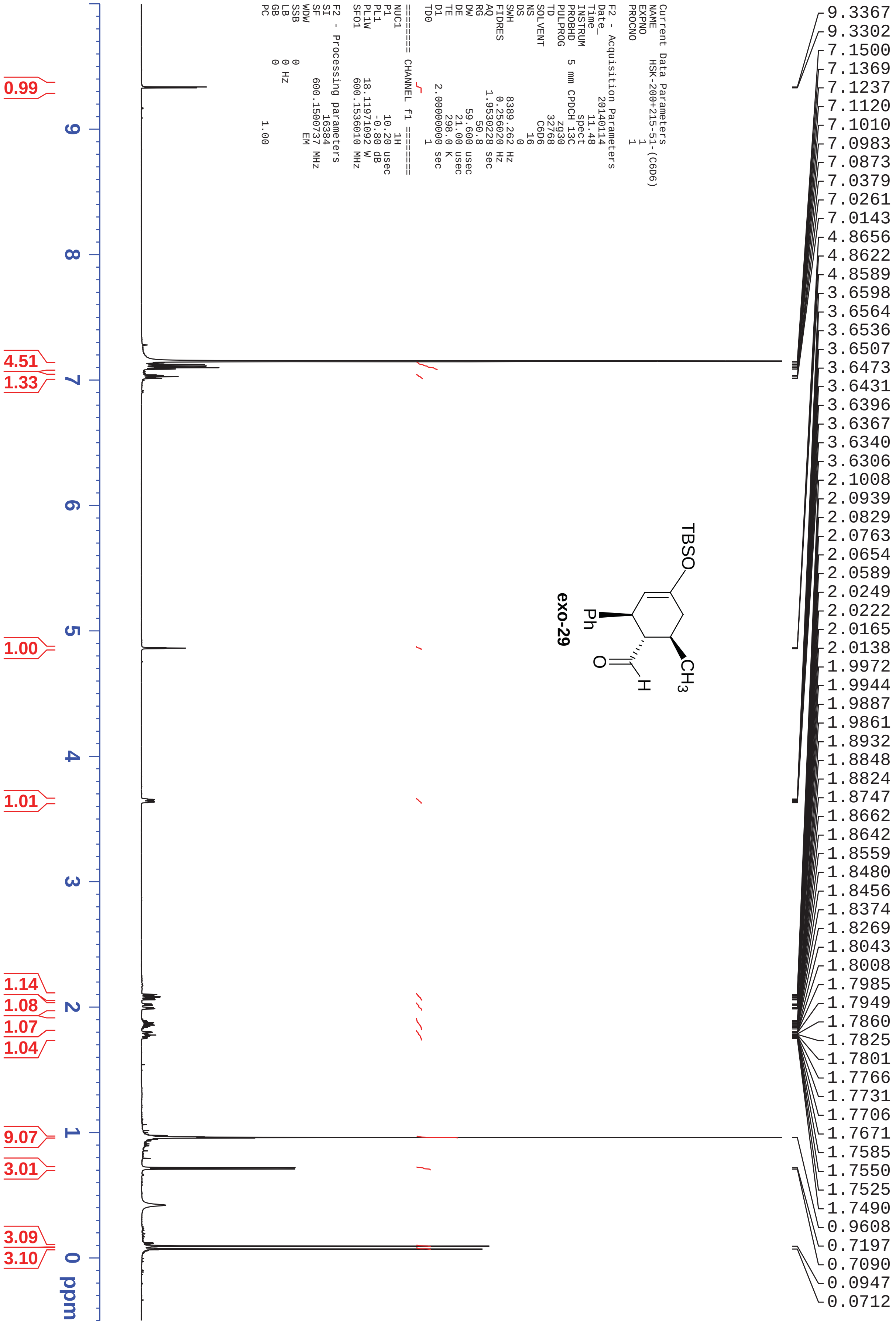

Supplementary Figure 74. ^{1}**H NMR spectrum of compound exo-29.**

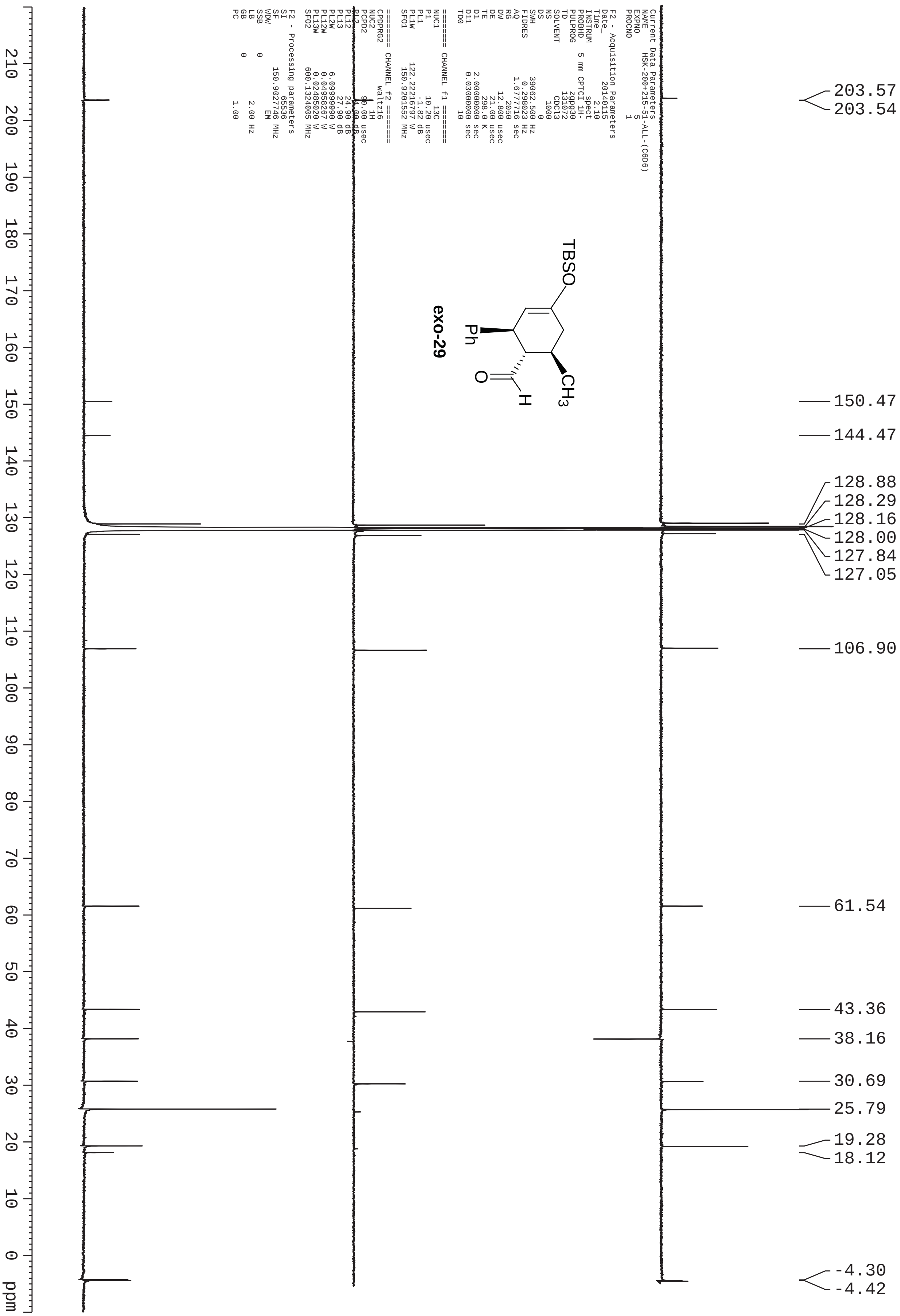

Supplementary Figure 75. ^{13}C and DEPT NMR spectra of compound exo-29.

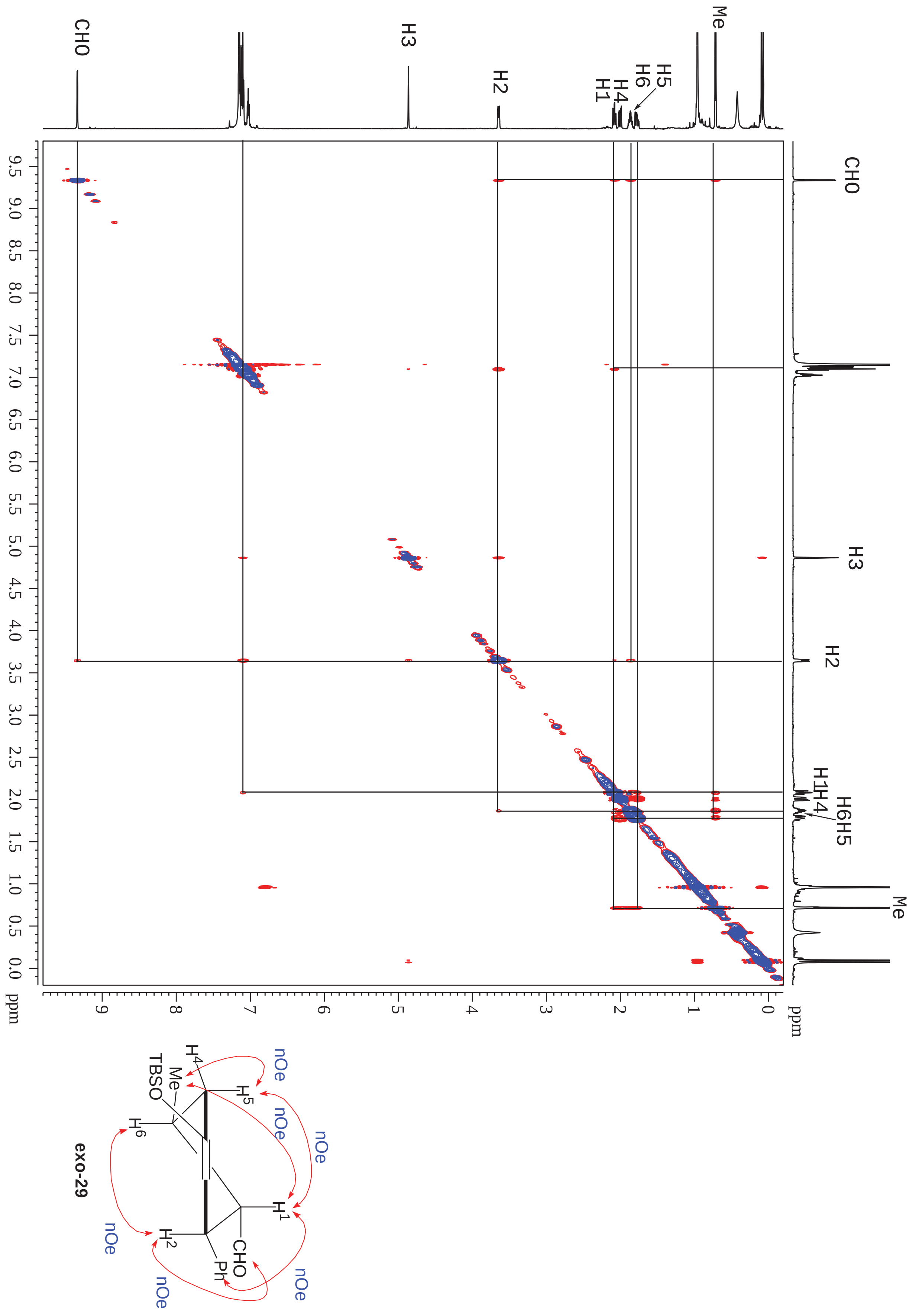

Supplementary Figure 76. NOESY NMR spectrum of compound exo-29.

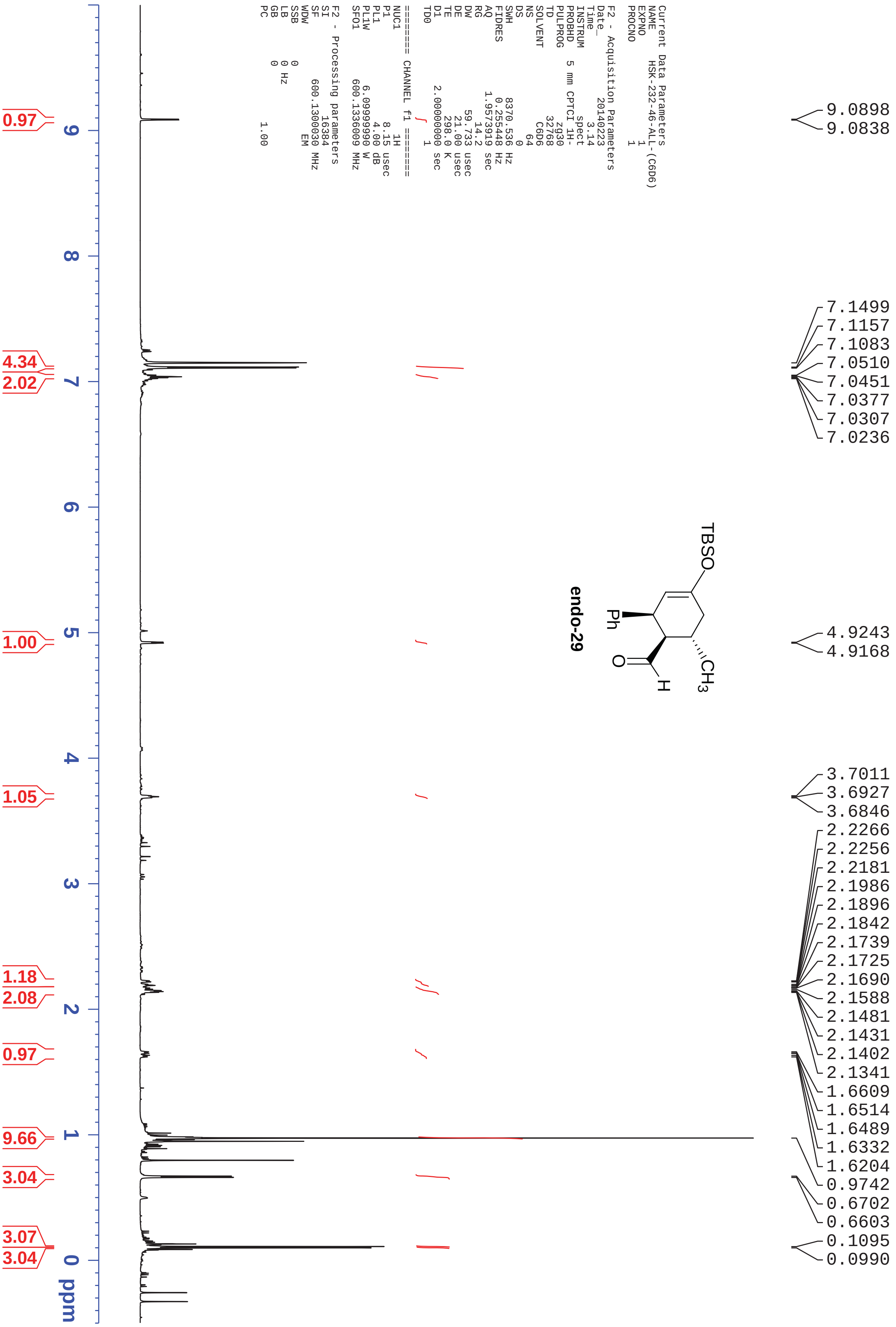

Supplementary Figure 77. ^{1}H NMR spectrum of compound endo-29.

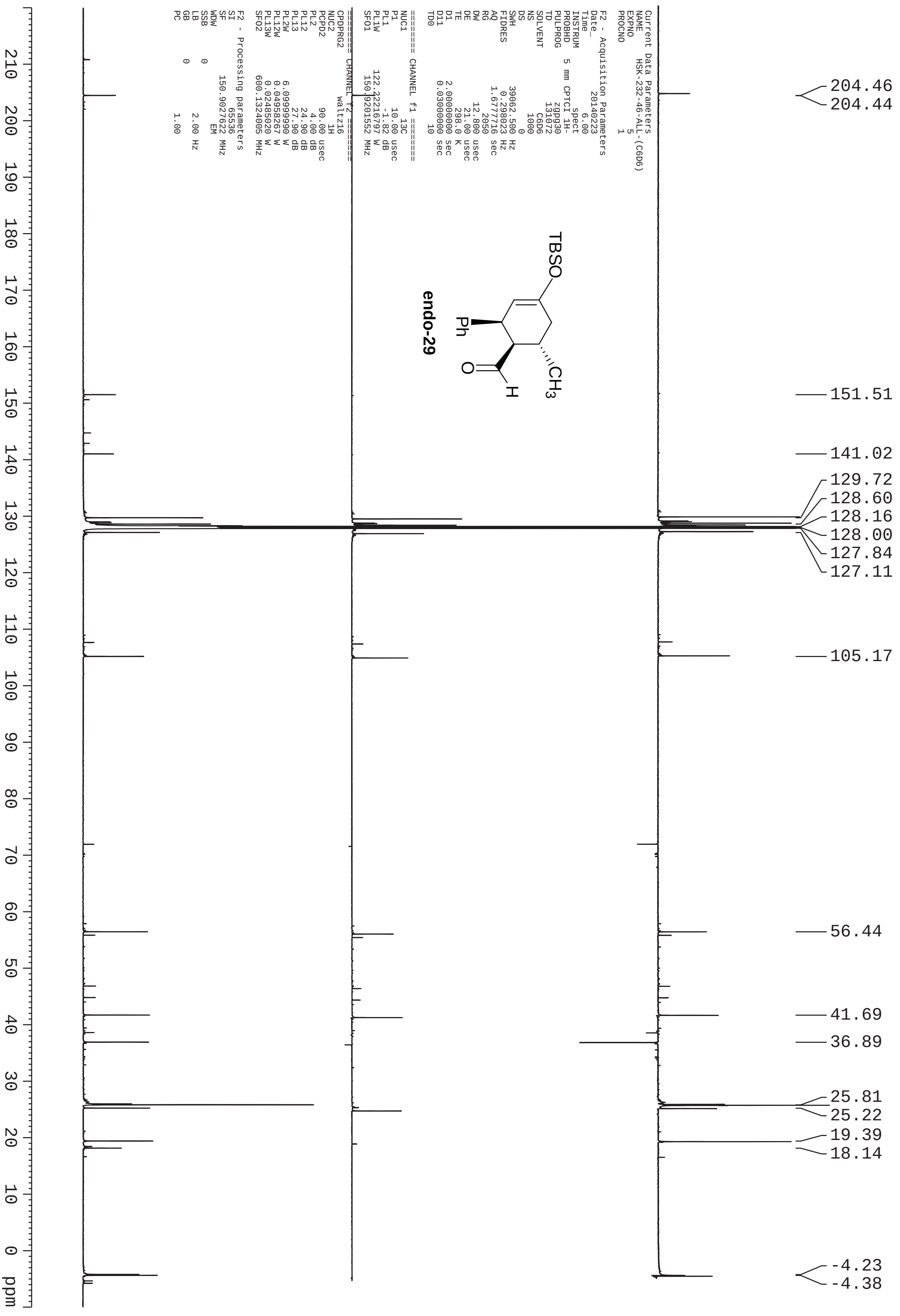

Supplementary Figure 78. ^{13}C and DEPT NMR spectra of compound endo-29.

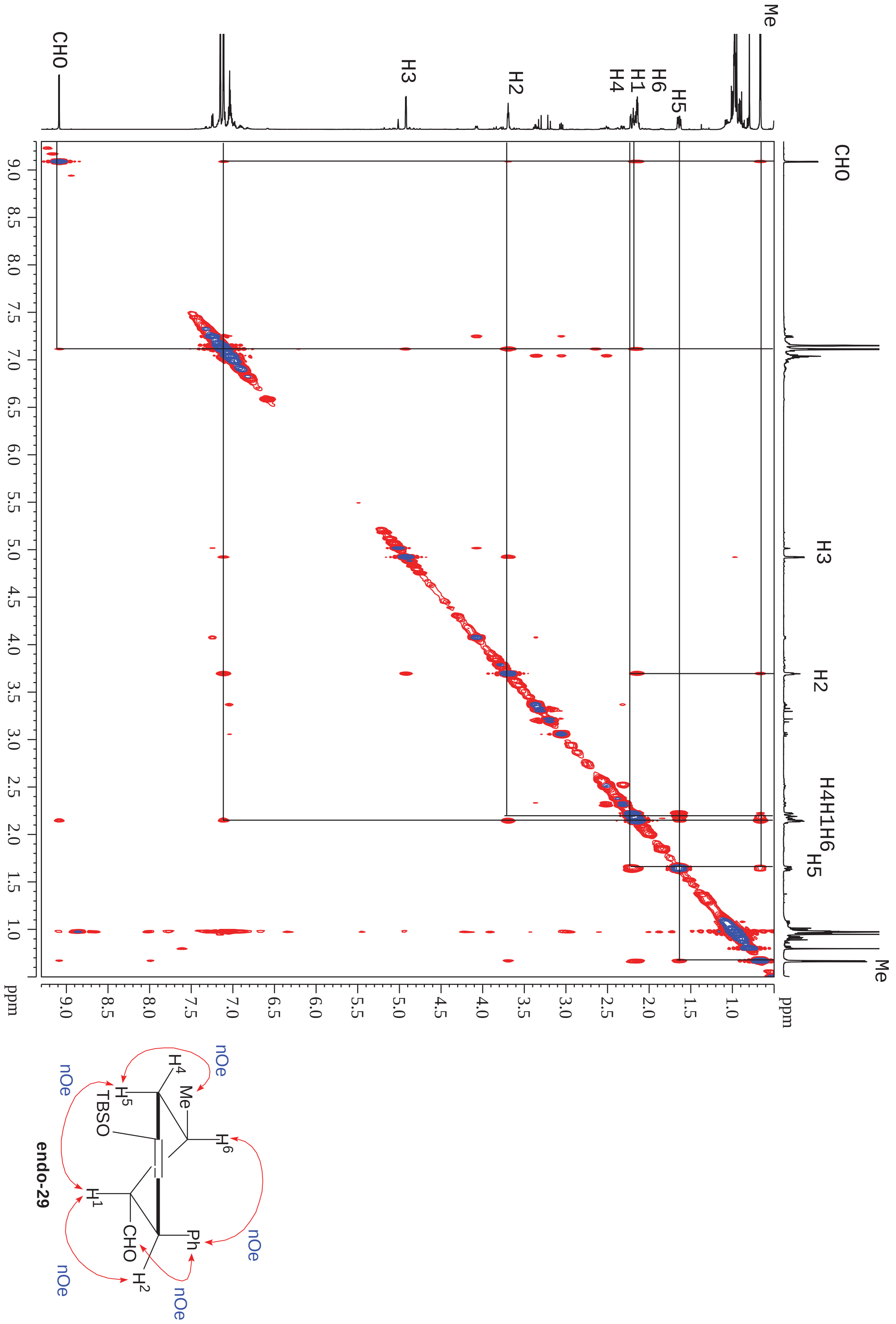

Supplementary Figure 79. NOESY NMR spectrum of compound endo-29.

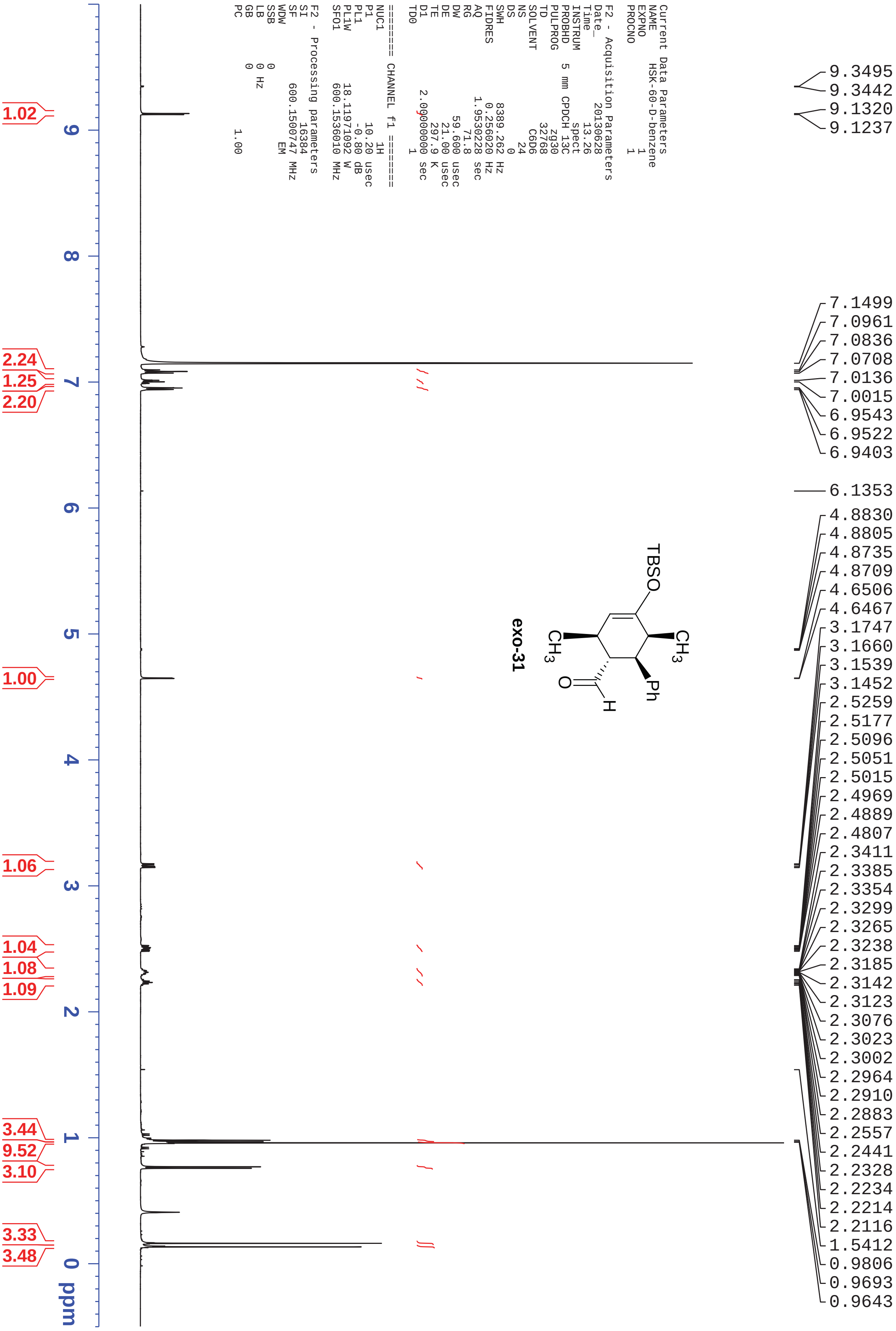

Supplementary Figure 80. ^{1}**H NMR spectrum of compound exo-31.**

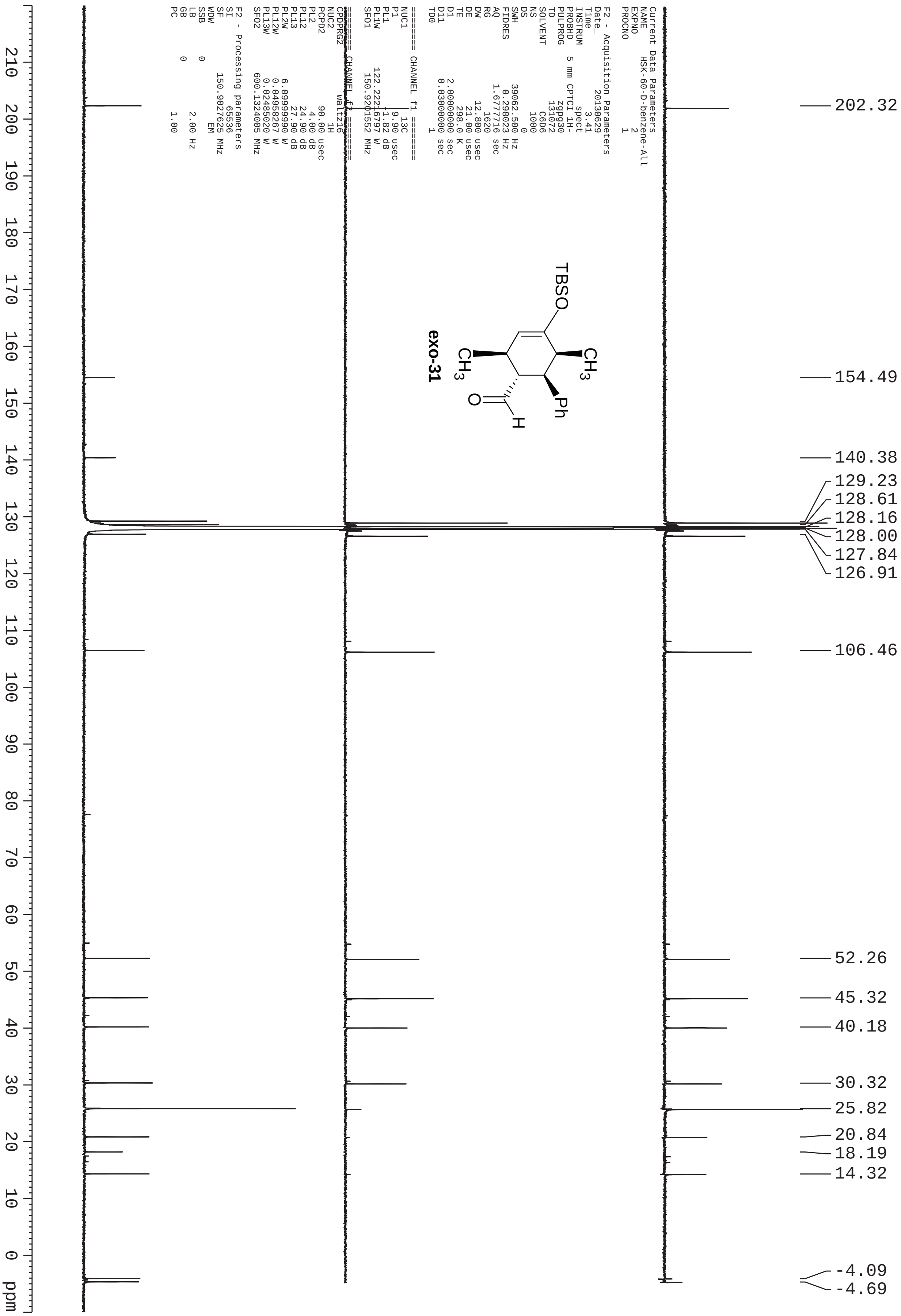

Supplementary Figure 81. ^{13}C and DEPT NMR spectra of compound exo-31.

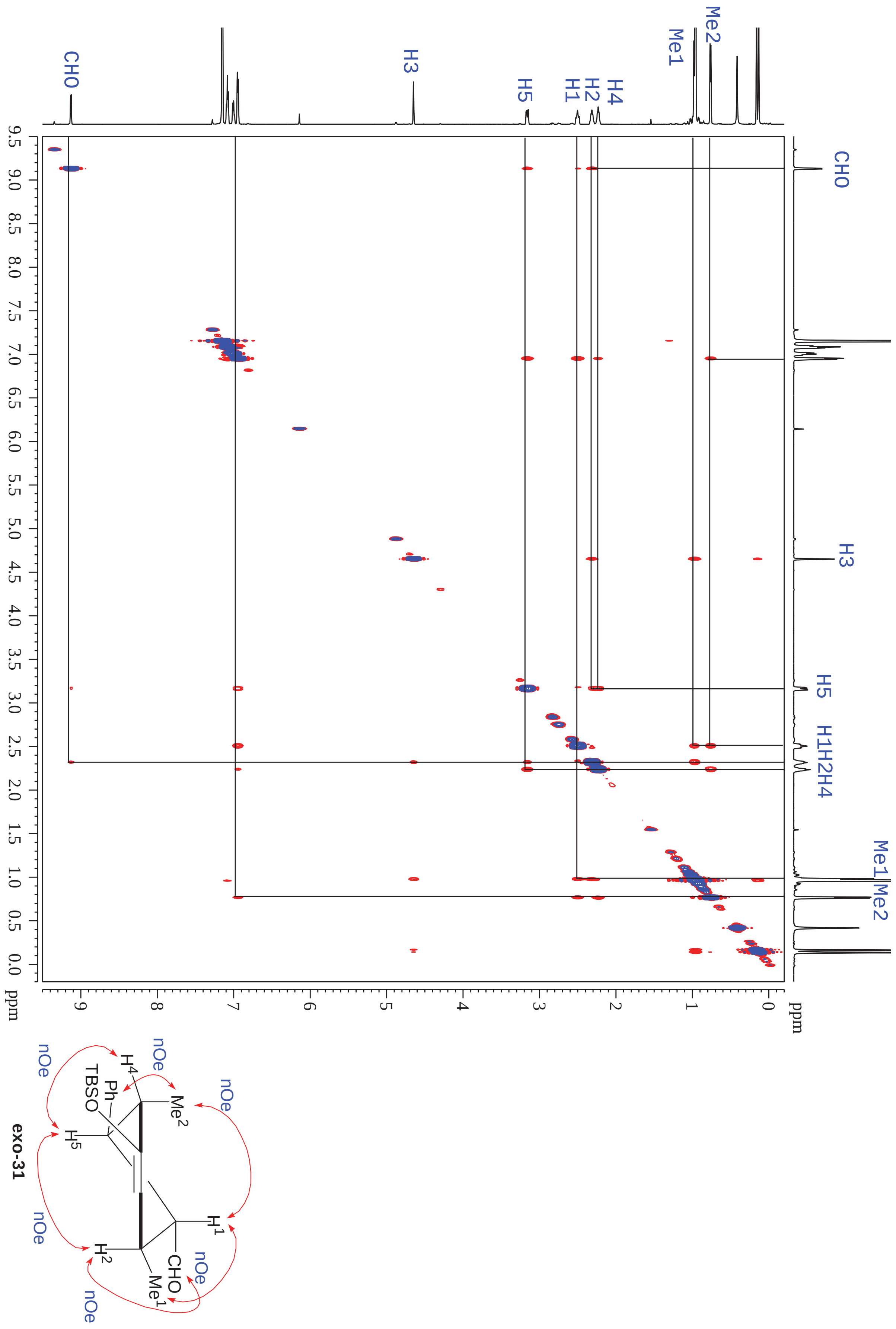

Supplementary Figure 82. NOESY NMR spectrum of compound exo-31.

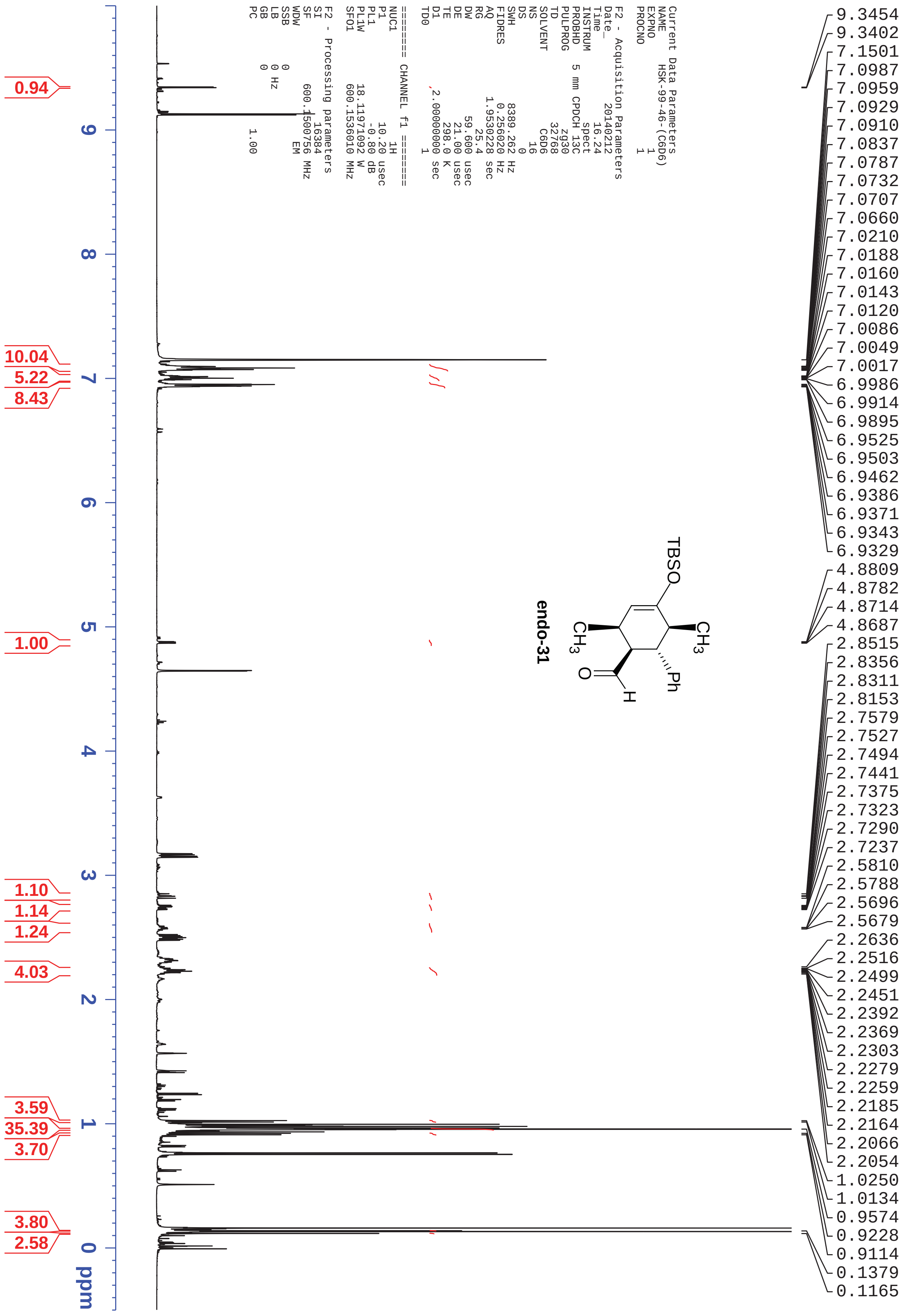

Supplementary Figure 83. **¹H NMR spectrum of compound endo-31.**

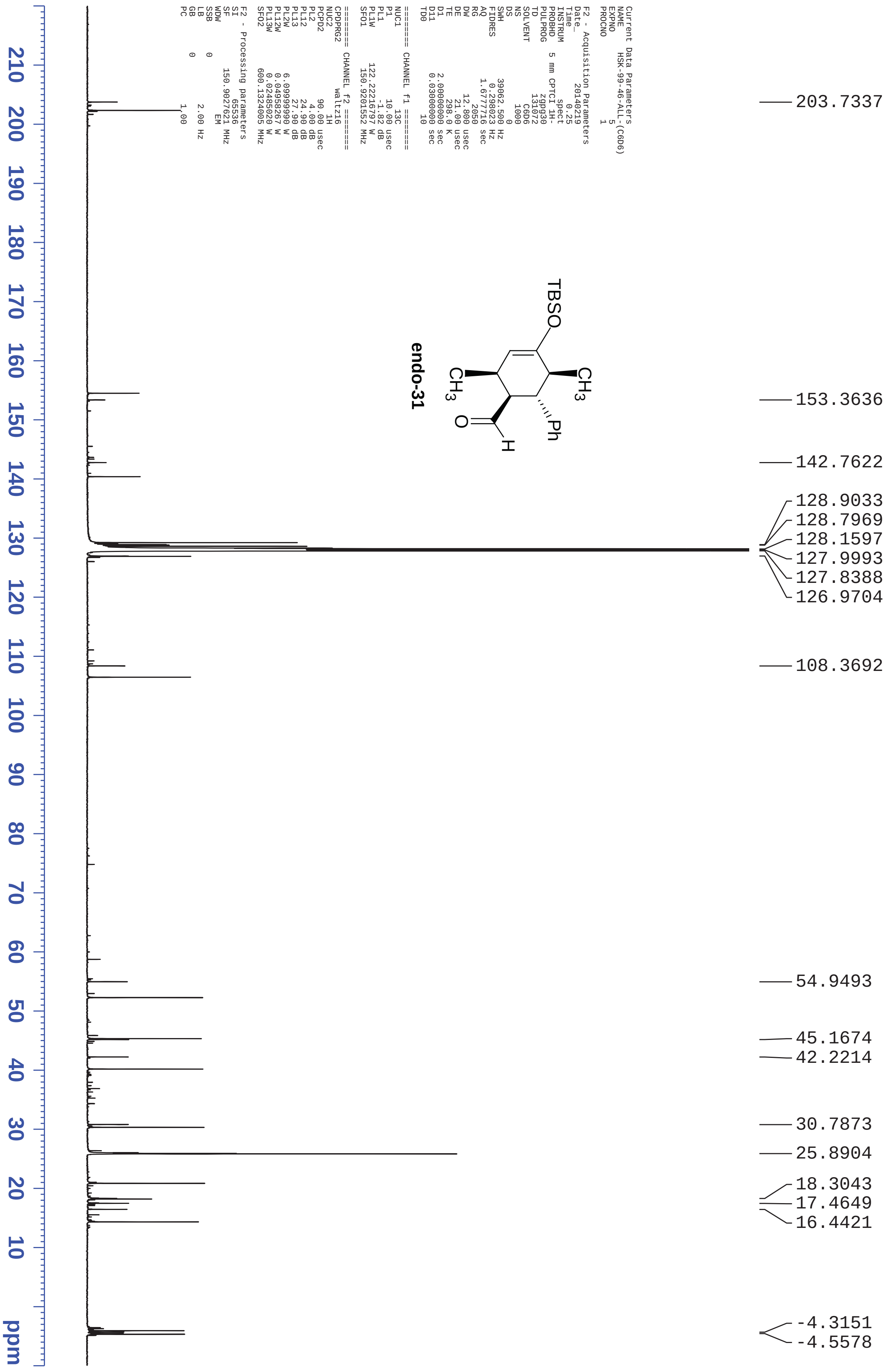

Supplementary Figure 84. ^{13}C and DEPT NMR spectra of compound endo-31.

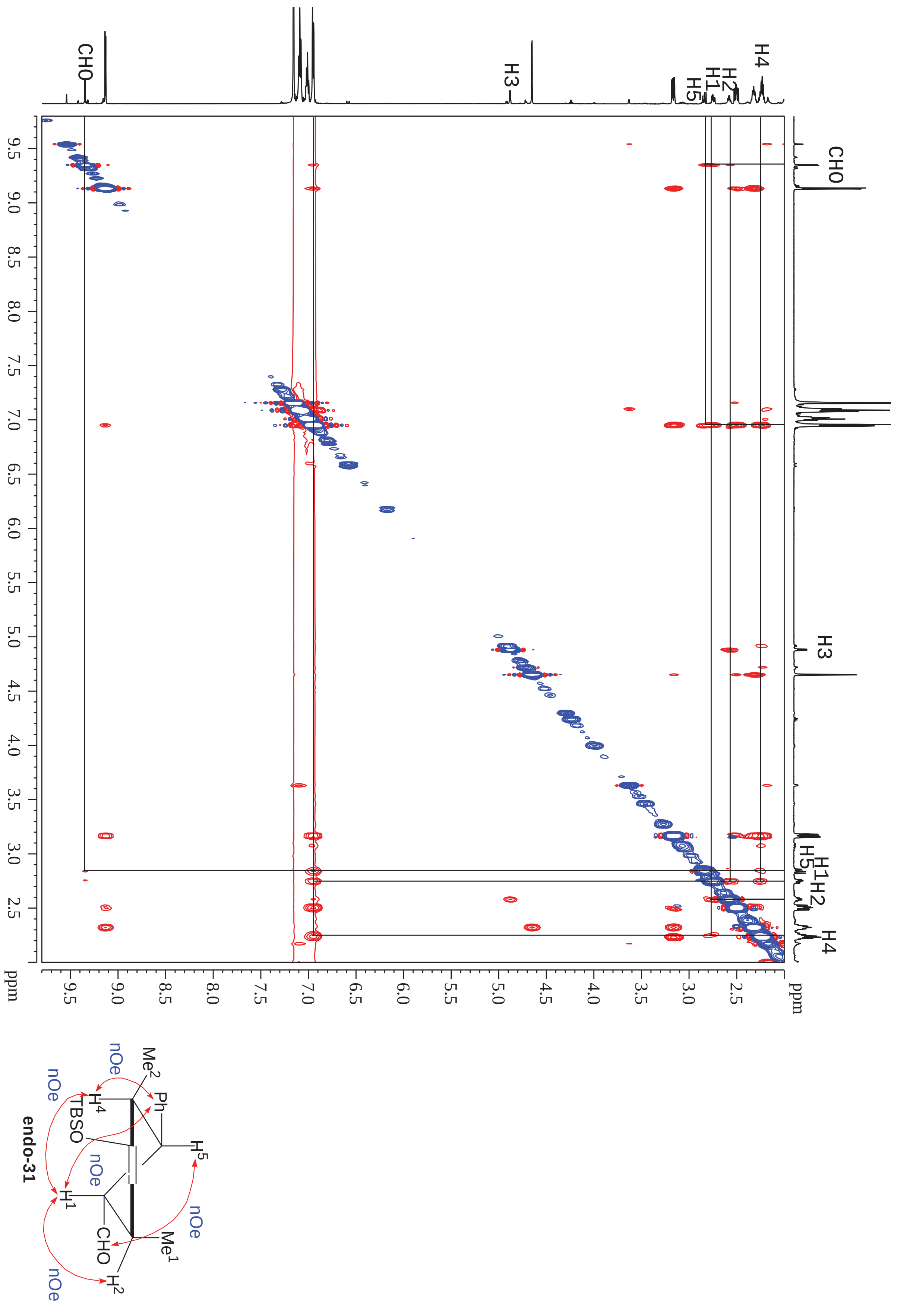

Supplementary Figure 85. NOESY NMR spectrum of compound endo-31.

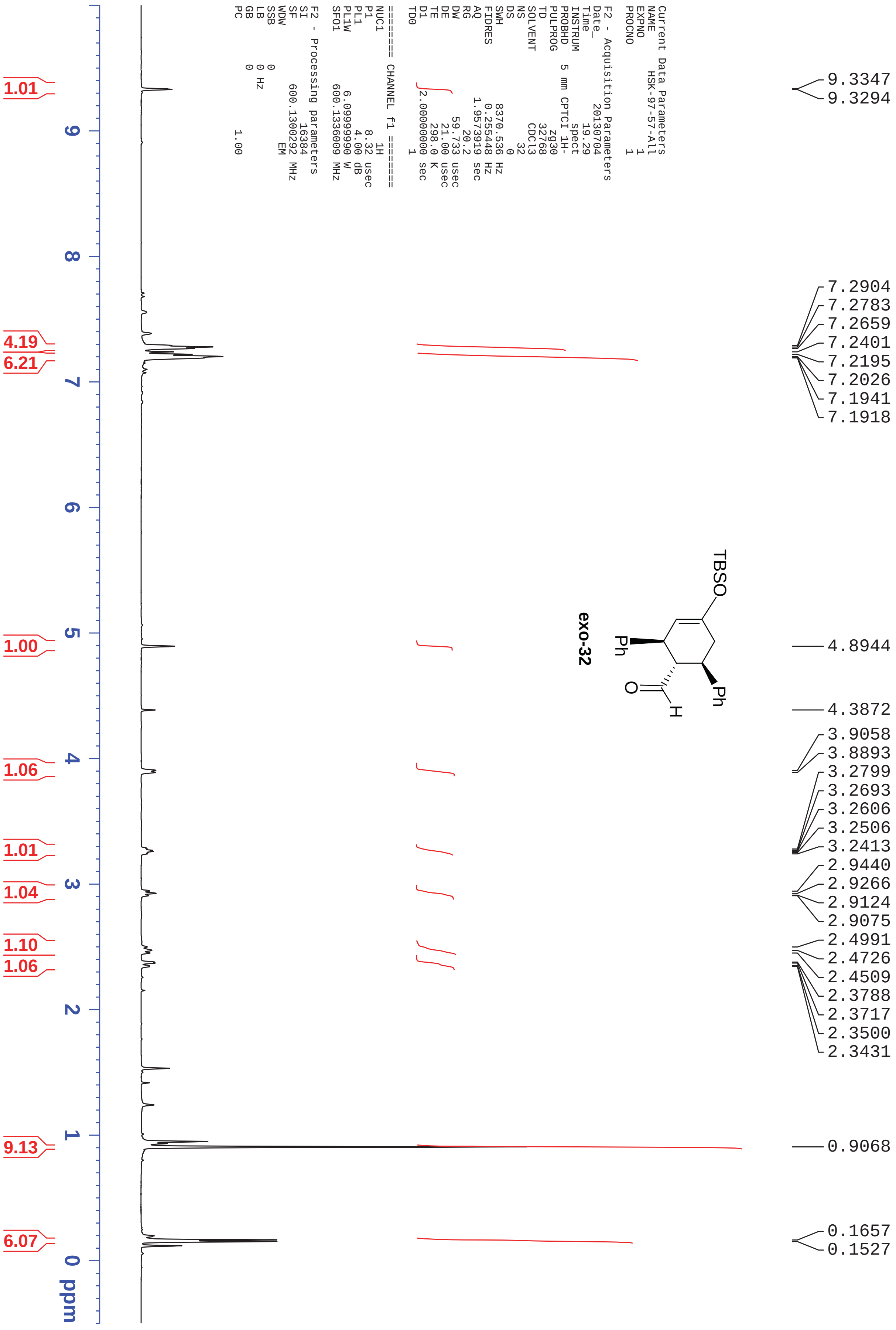

Supplementary Figure 86. **¹H NMR spectrum of compound exo-32.**

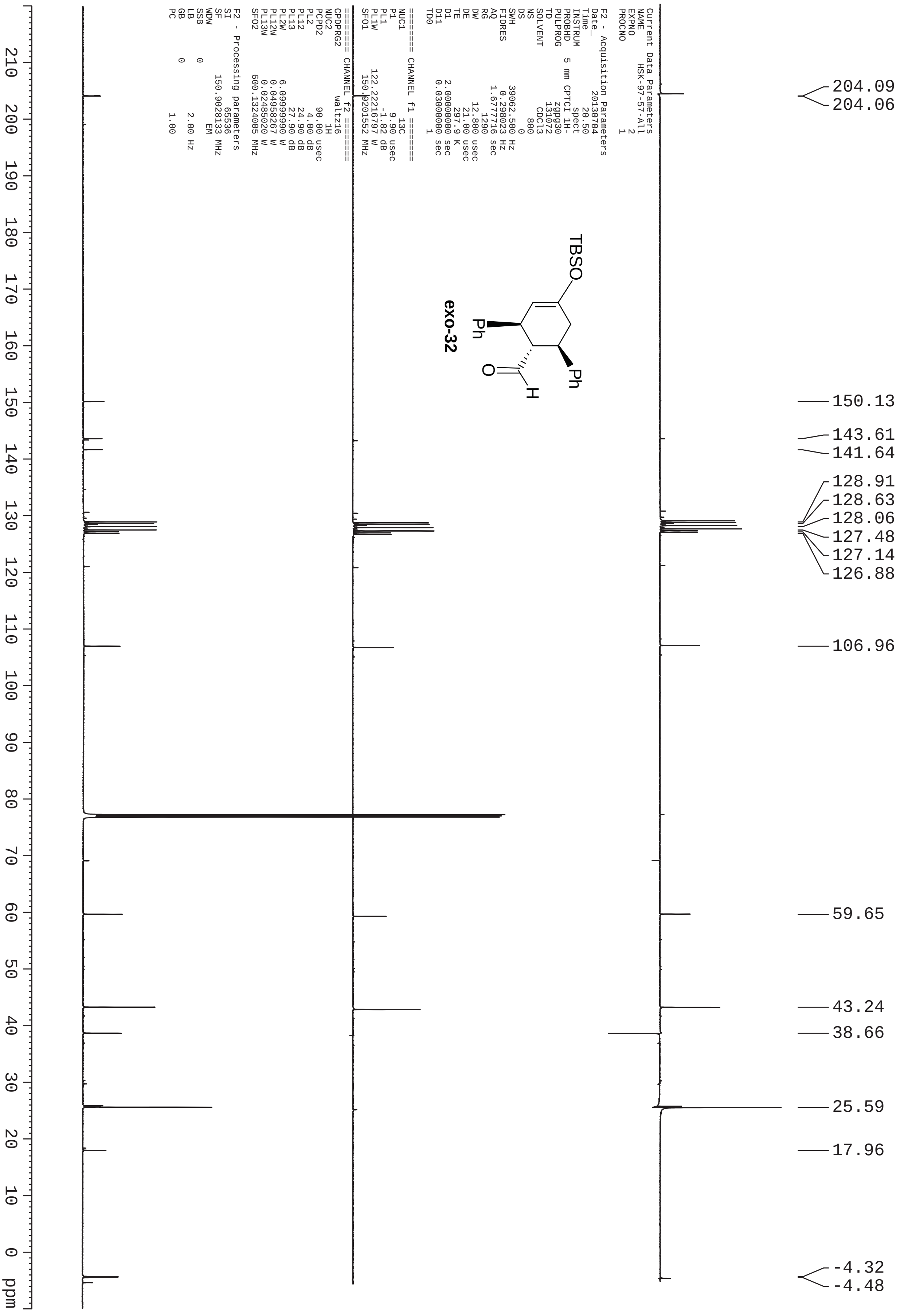

Supplementary Figure 87. ^{13}C and DEPT NMR spectra of compound exo-32.

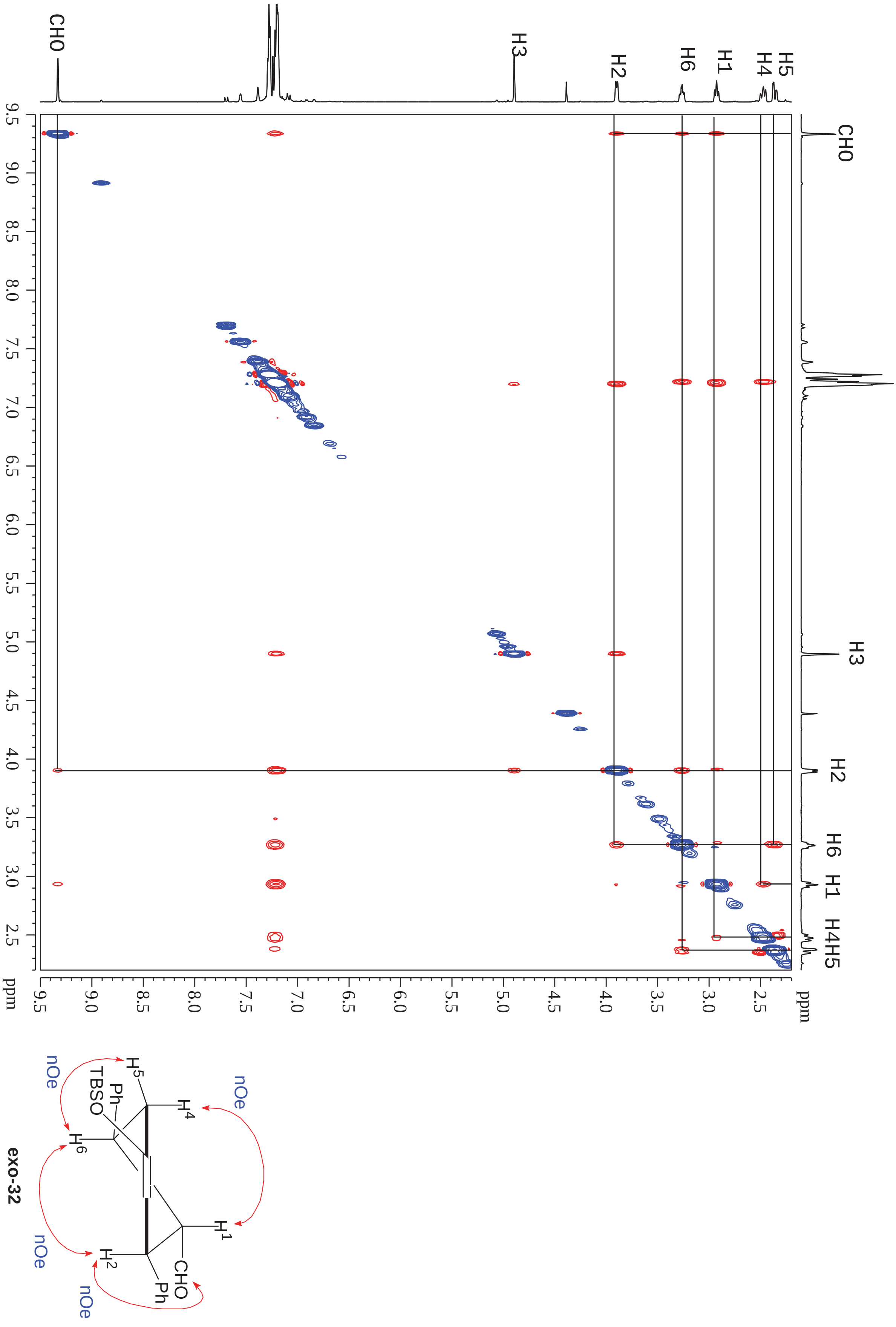

Supplementary Figure 88. NOESY NMR spectrum of compound exo-32.

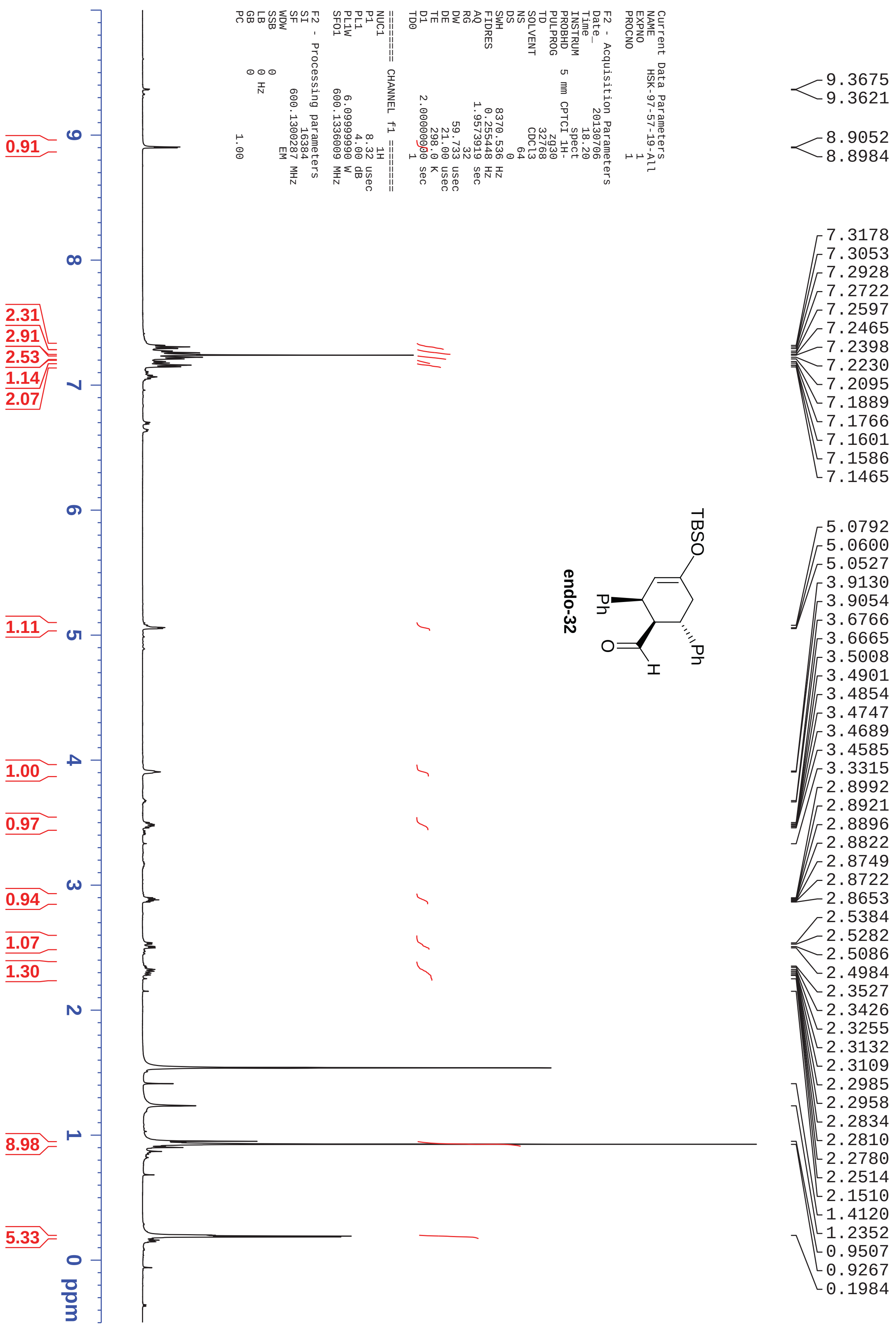

Supplementary Figure 89. ^{1}H NMR spectrum of compound endo-32.

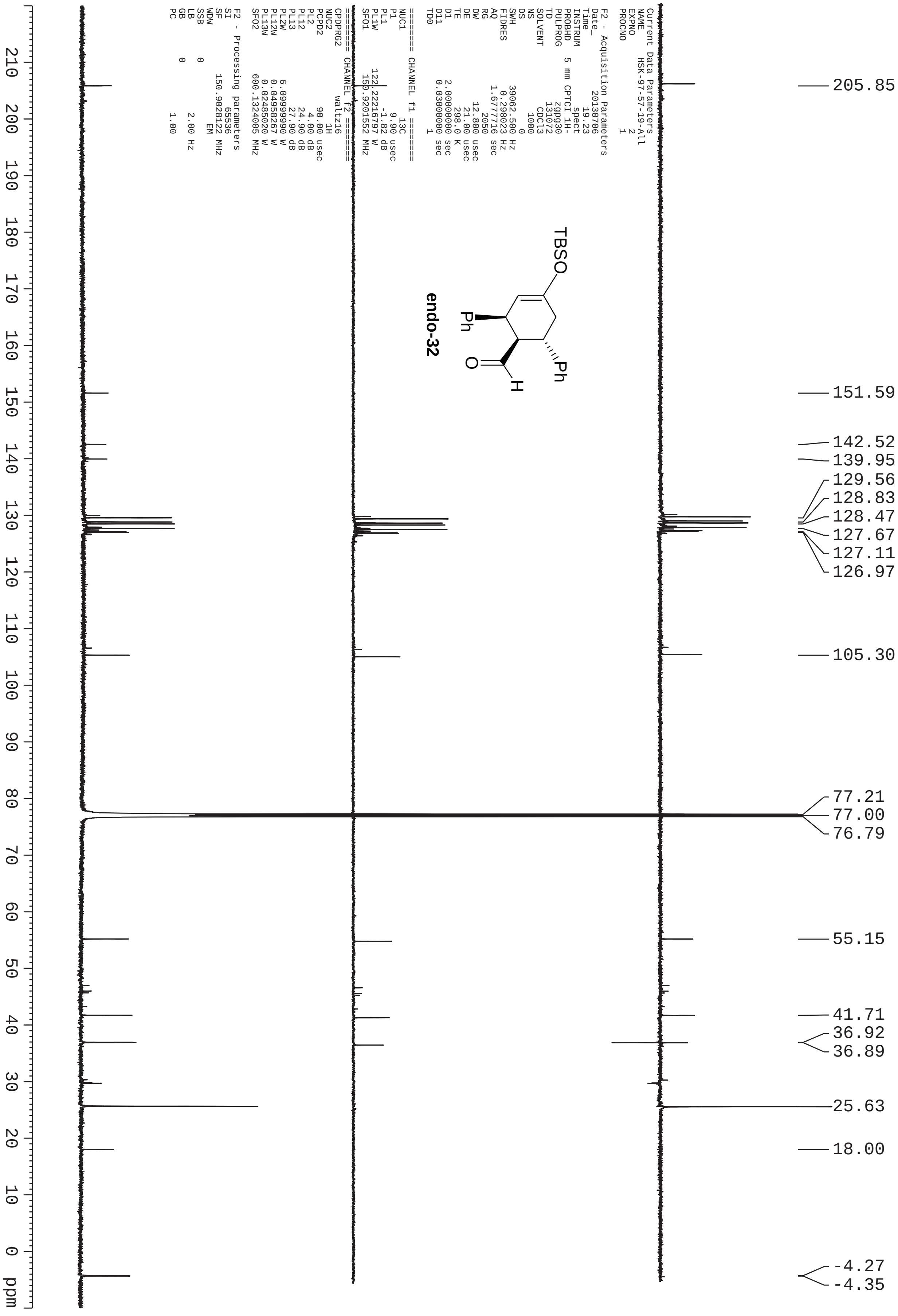

Supplementary Figure 90. ^{13}C and DEPT NMR spectra of compound endo-32.

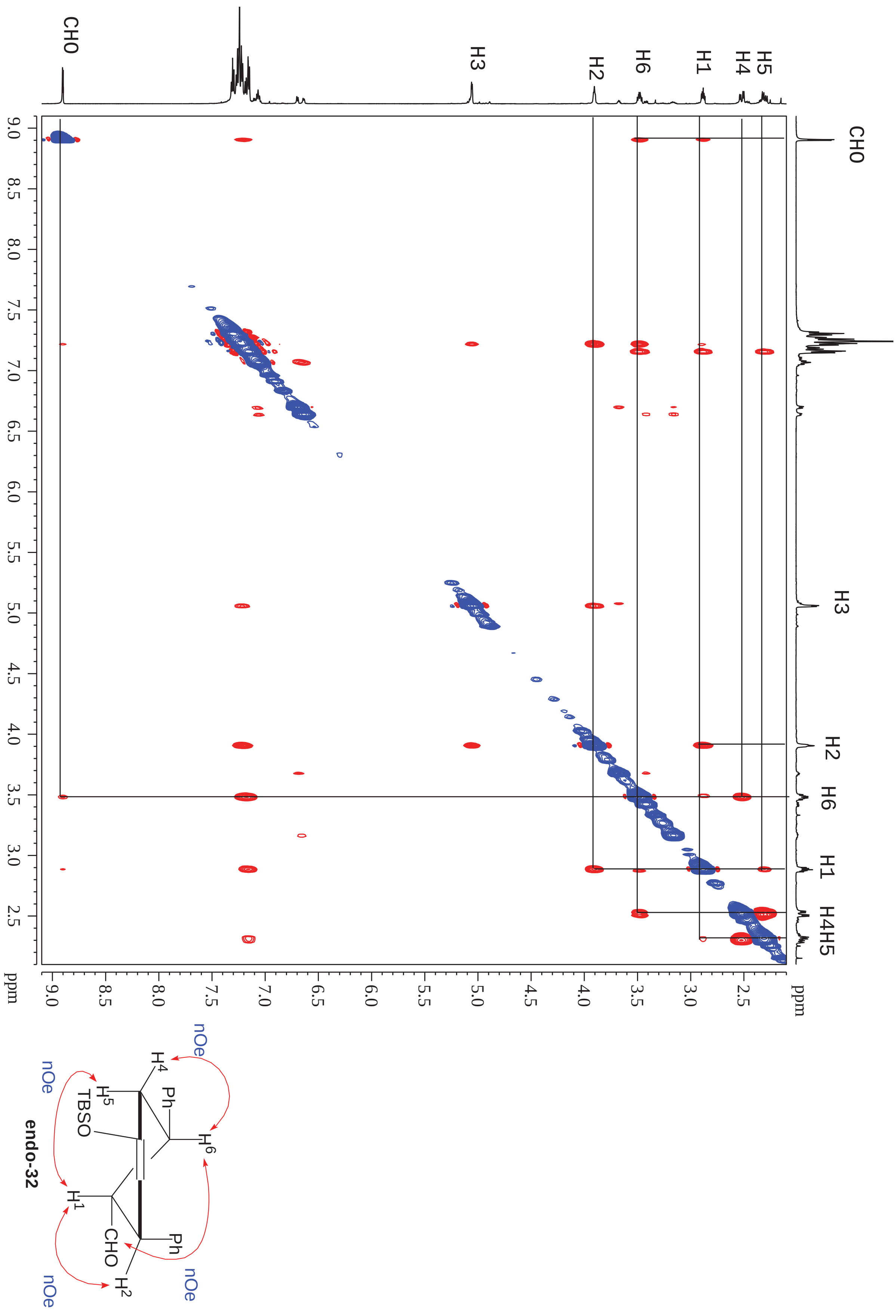

Supplementary Figure 91. NOESY NMR spectrum of compound endo-32.

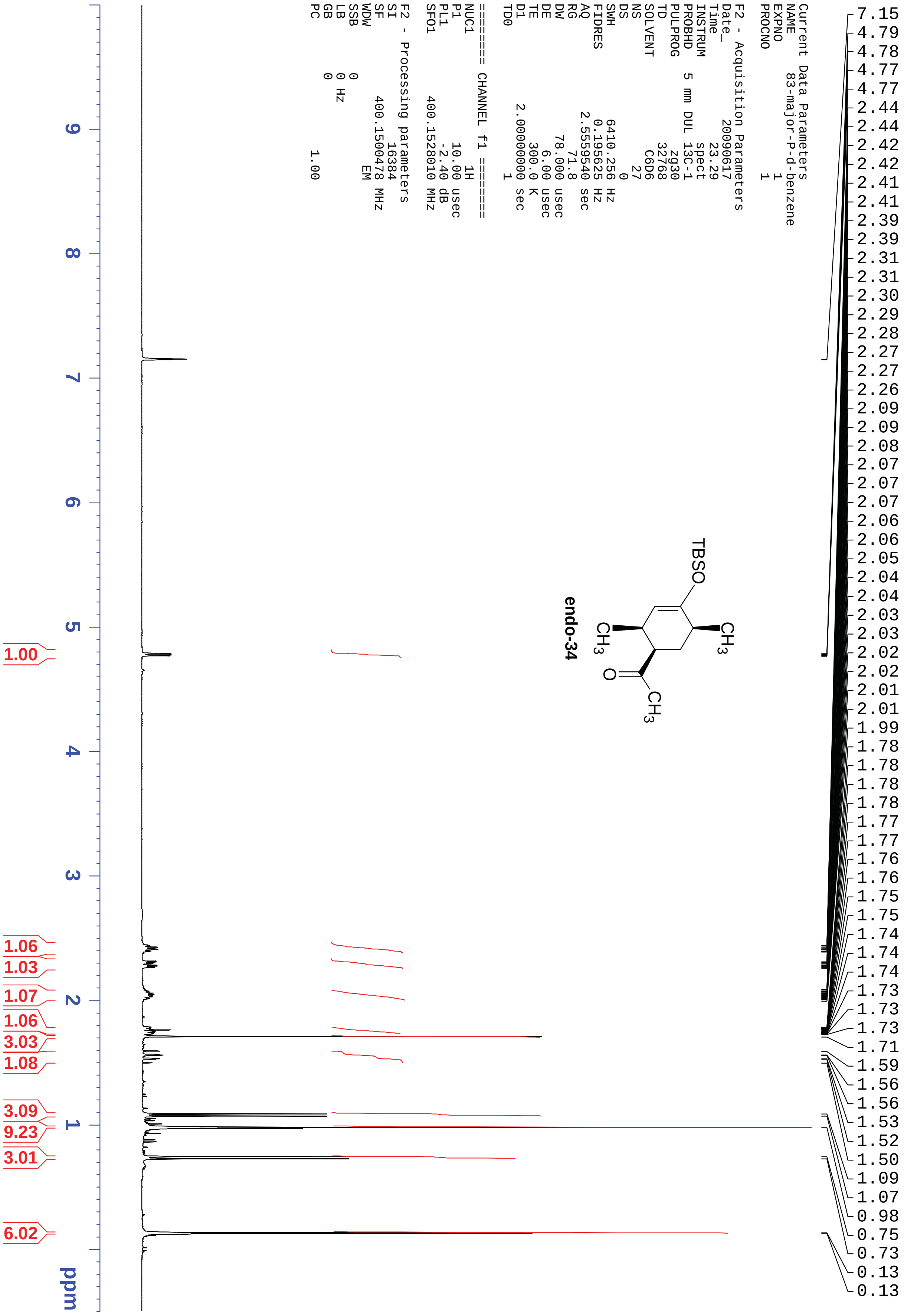

Supplementary Figure 92. ^{1}**H NMR spectrum of compound endo-34.**

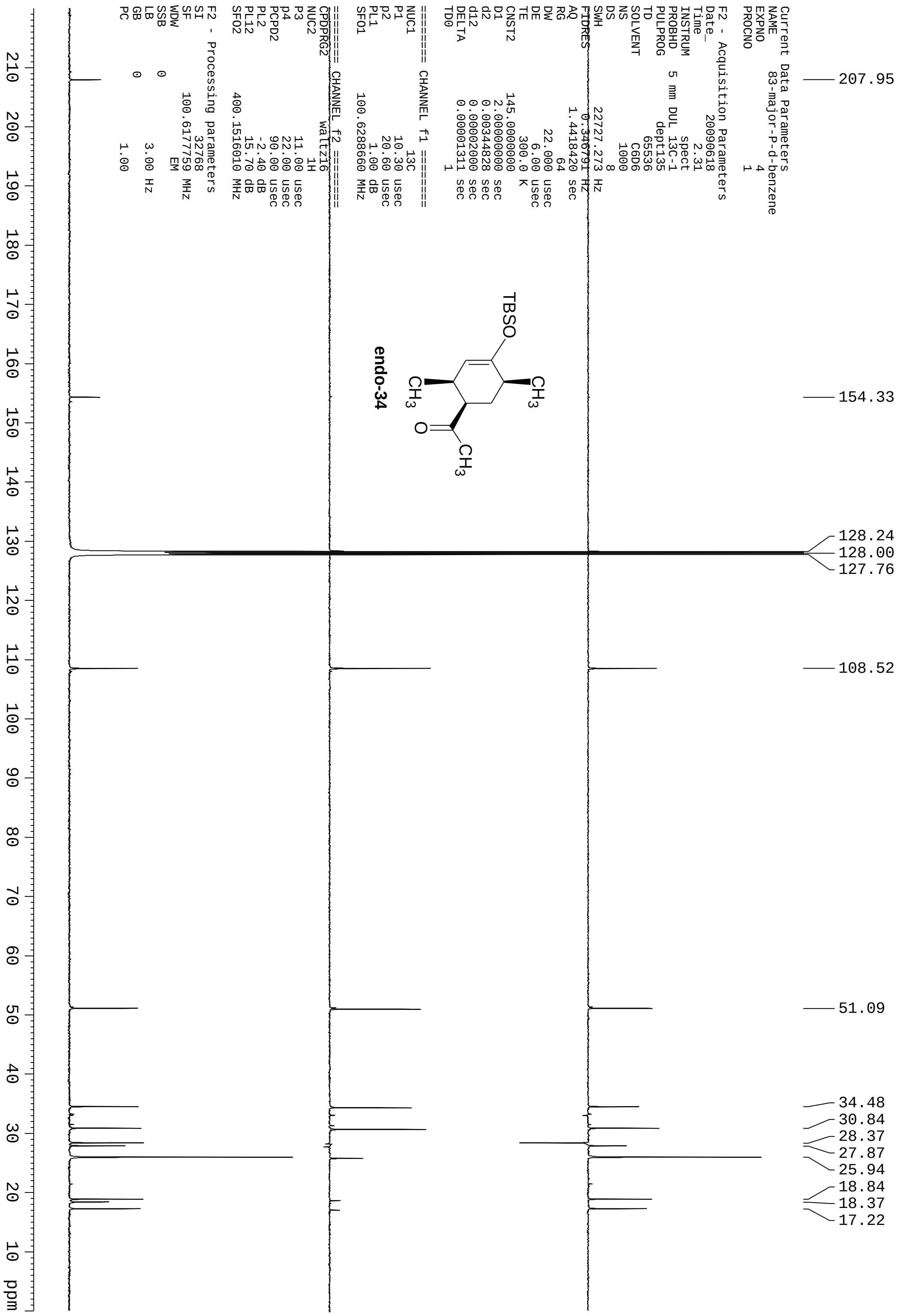

Supplementary Figure 93. ^{13}C and DEPT NMR spectra of compound endo-34.

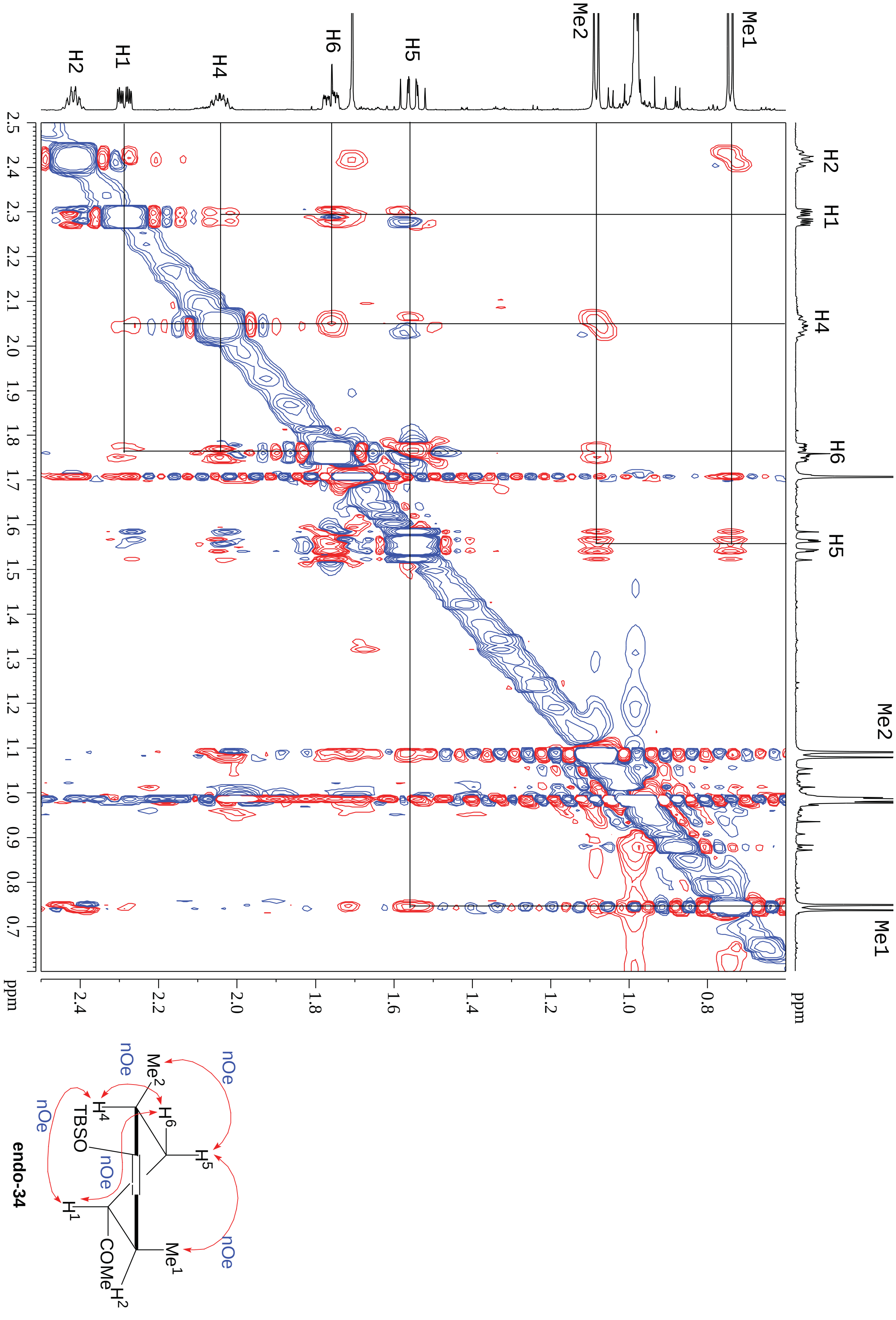

Supplementary Figure 94. NOESY NMR spectrum of compound endo-34.

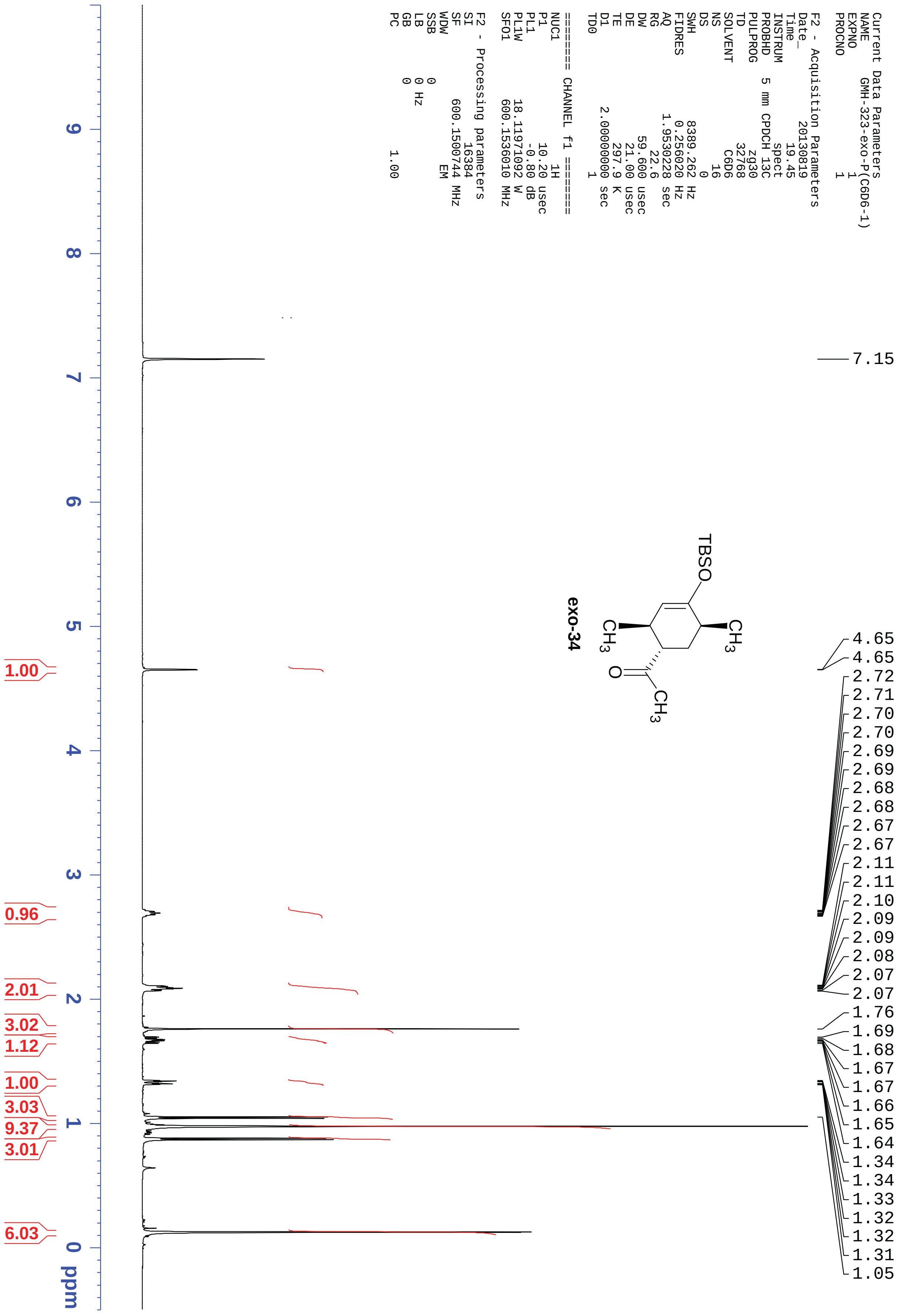

Supplementary Figure 95. ^{1}H NMR spectrum of compound exo-34 in C_6D_6.

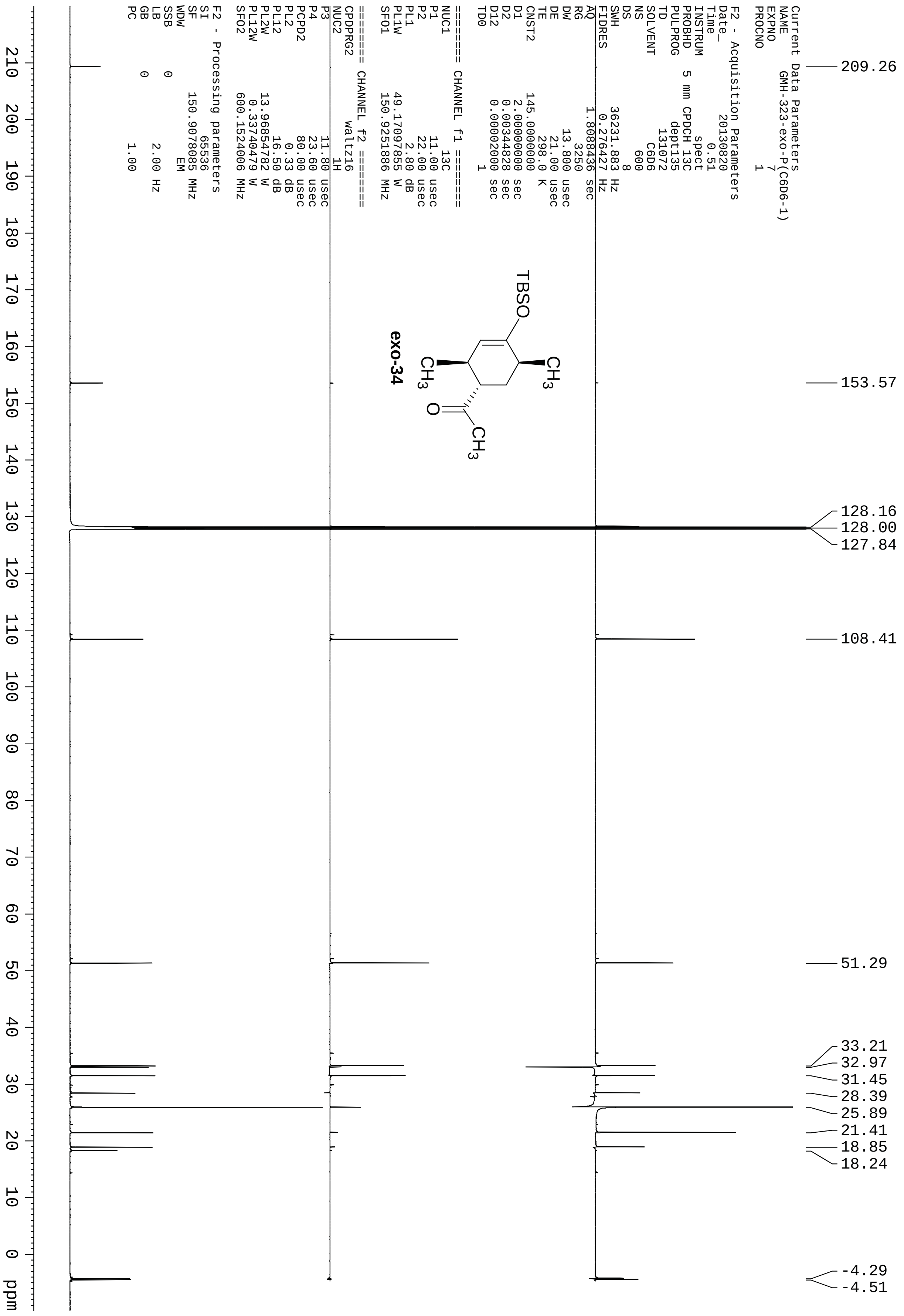

Supplementary Figure 96. ^{13}**C and DEPT NMR spectra of compound exo-34 in** C_6D_6**.**

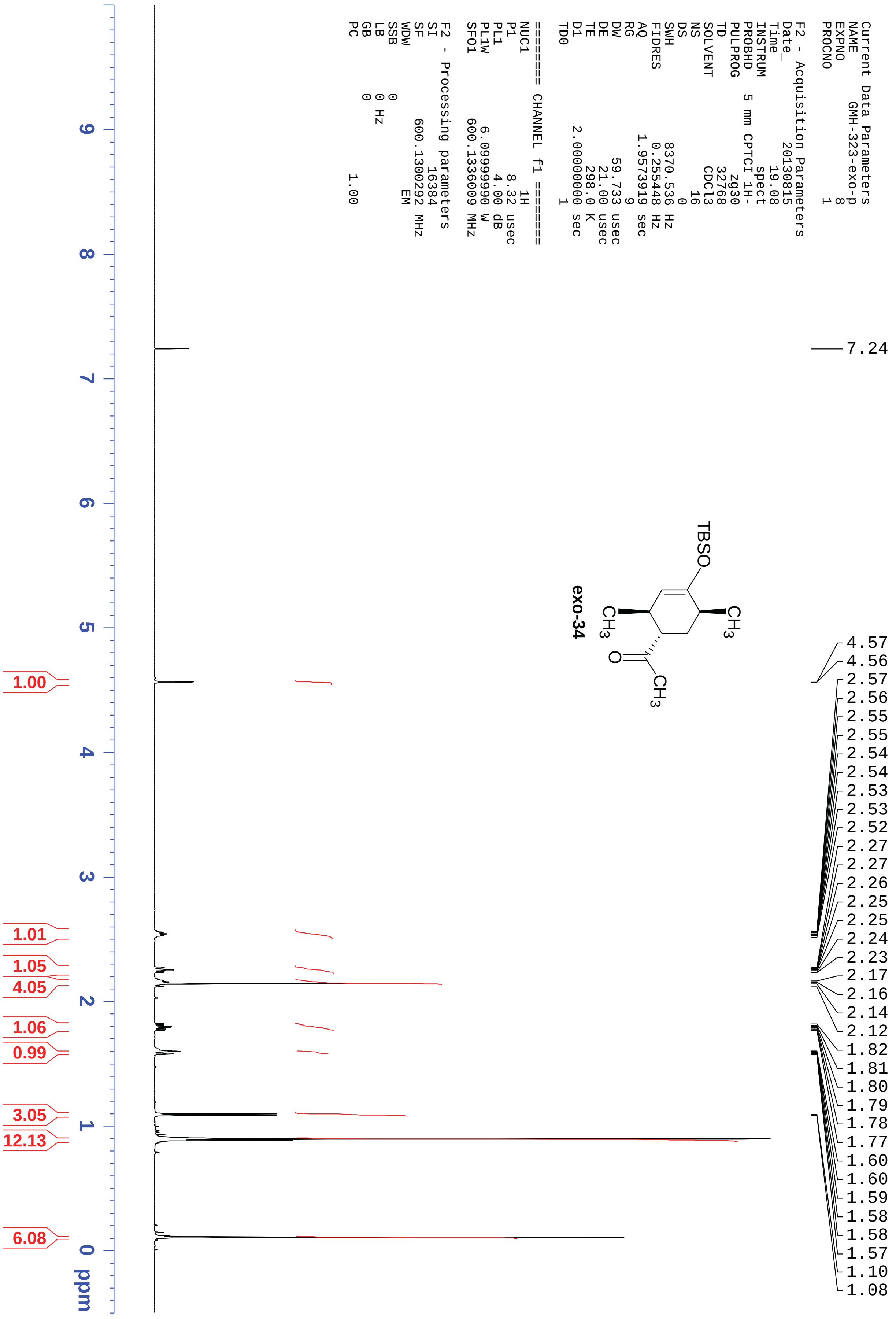

Supplementary Figure 97. **^{1}H NMR spectrum of compound exo-34 in $CDCl_3$.**

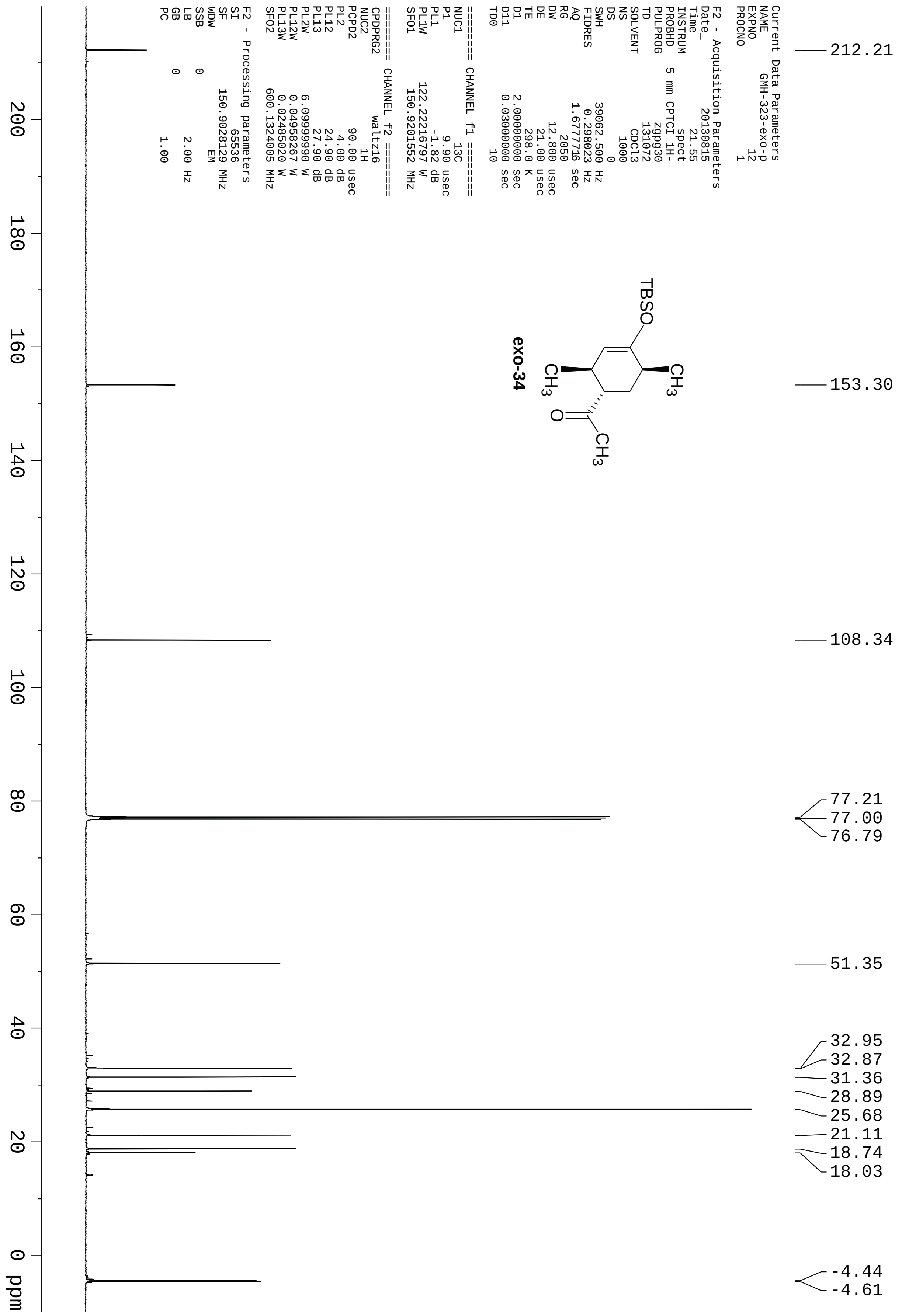

Supplementary Figure 98. ^{13}C spectrum of compound exo-34 in $CDCl_3$.

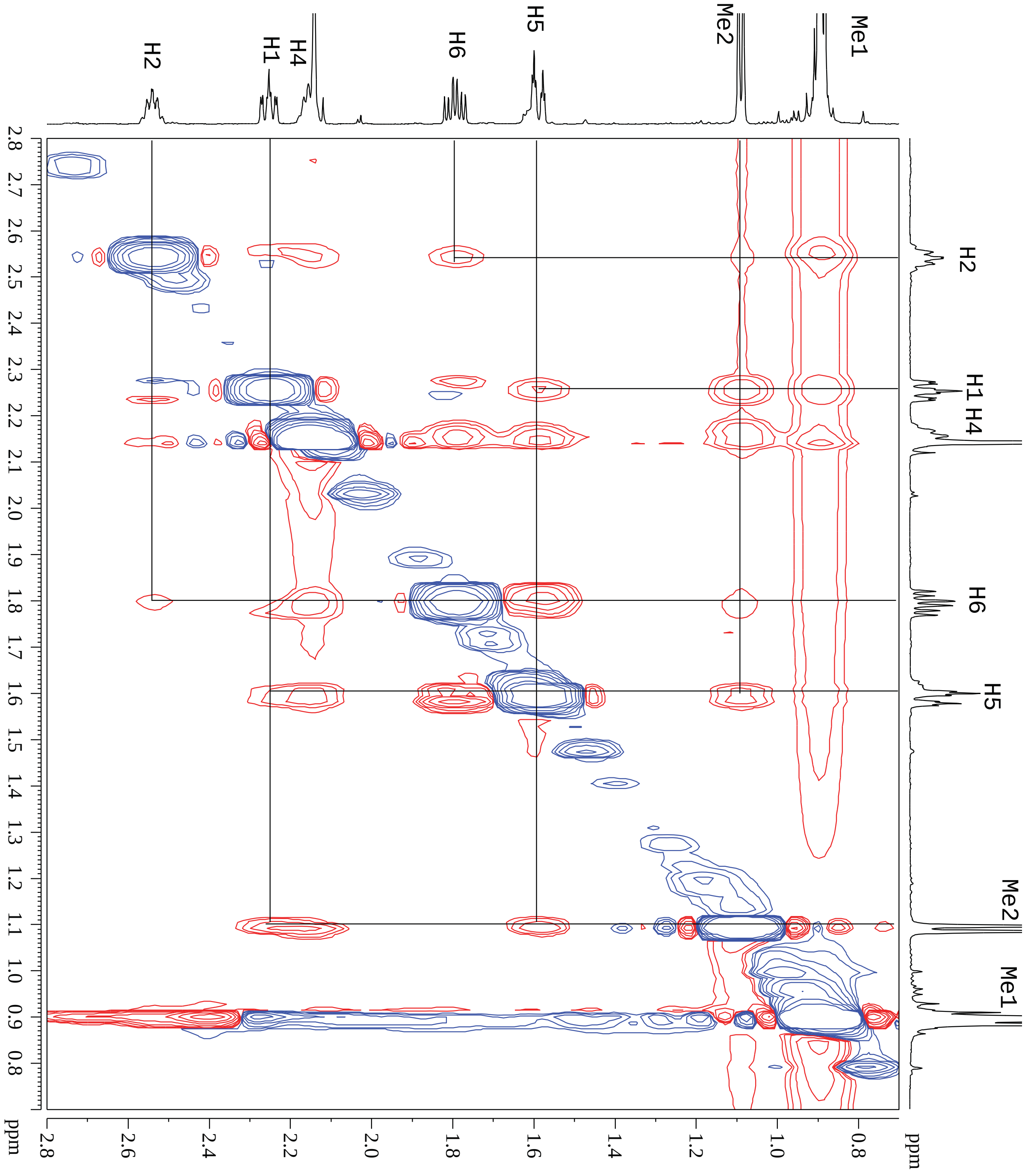

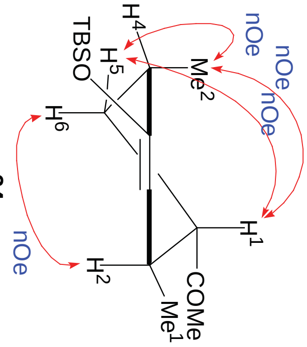

exo-34

Supplementary Figure 99. NOESY NMR spectrum of compound exo-34 in $CDCl_3$.

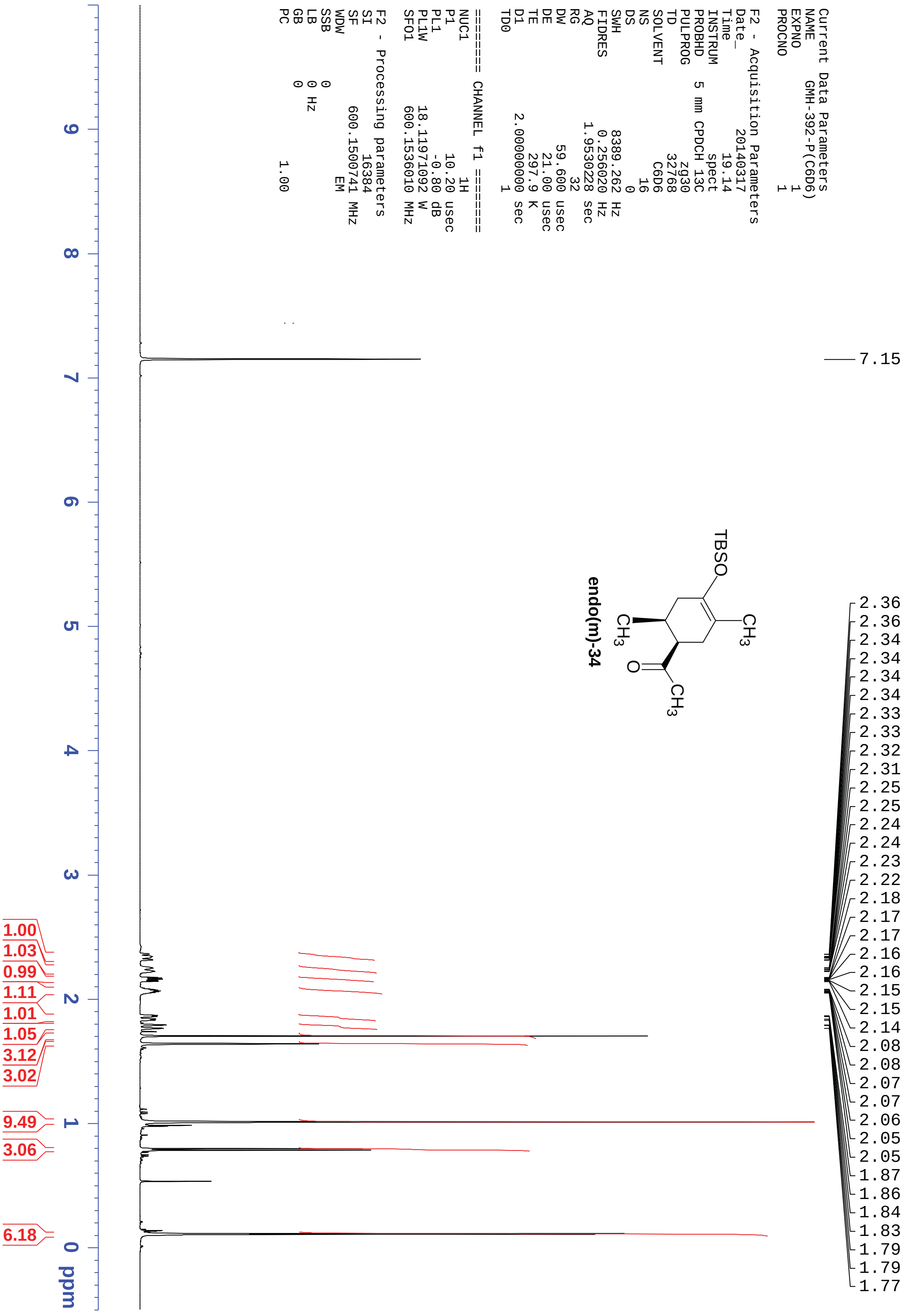

Supplementary Figure 100. ¹H NMR spectrum of compound endo(m)-34.

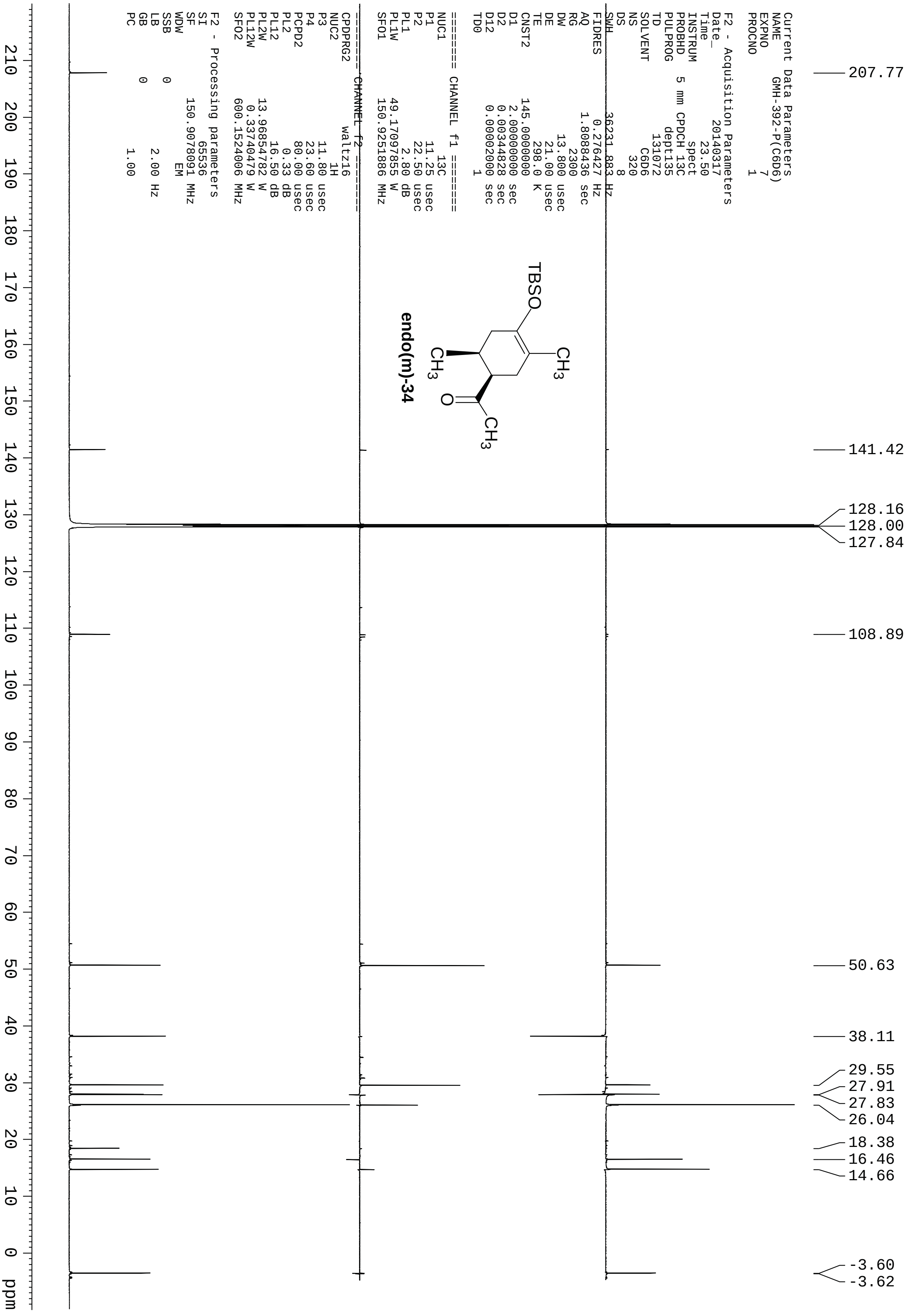

Supplementary Figure 101. ^{13}C and DEPT NMR spectra of compound endo(m)-34.

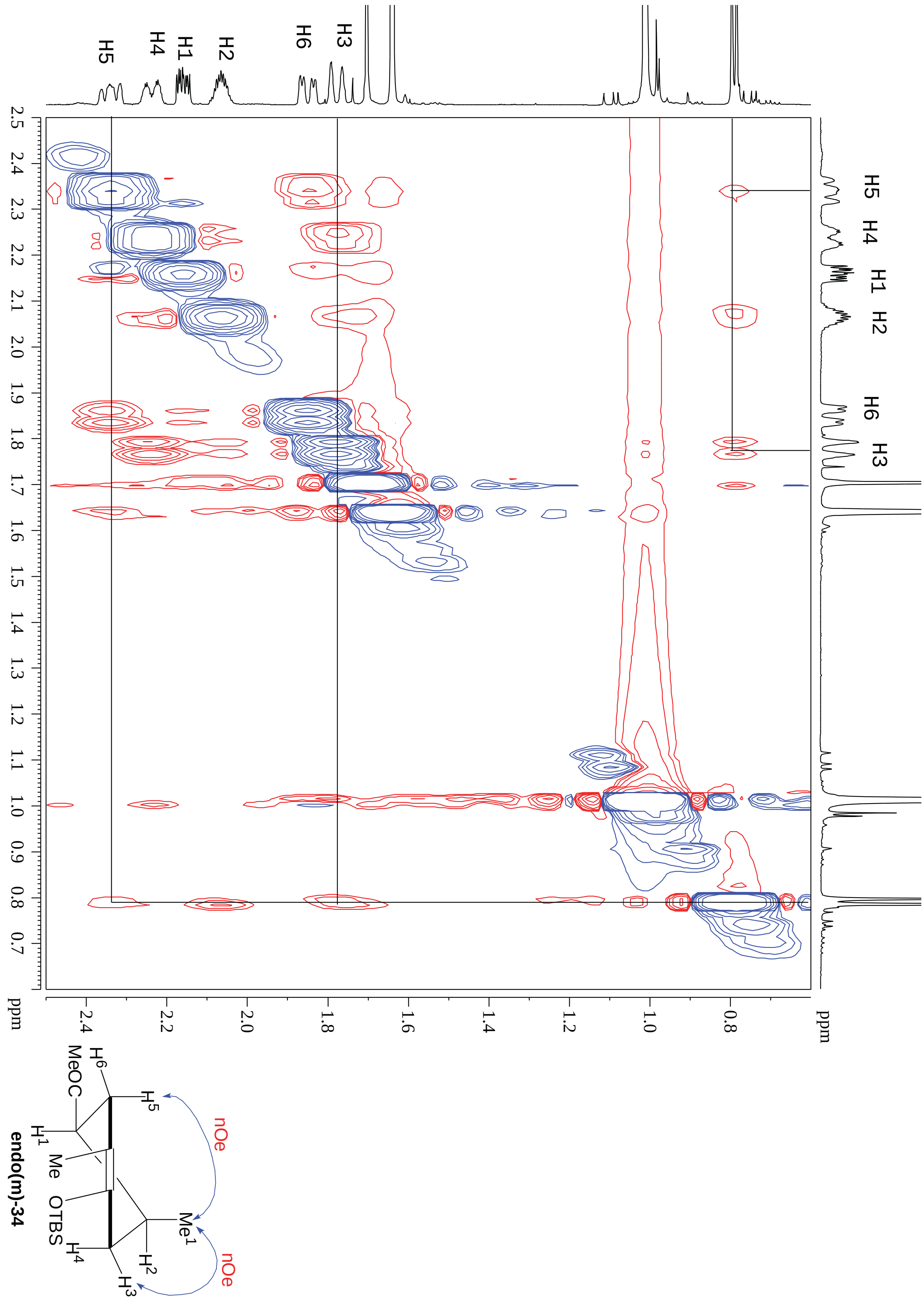

Supplementary Figure 102. NOESY NMR spectrum of compound endo(m)-34.

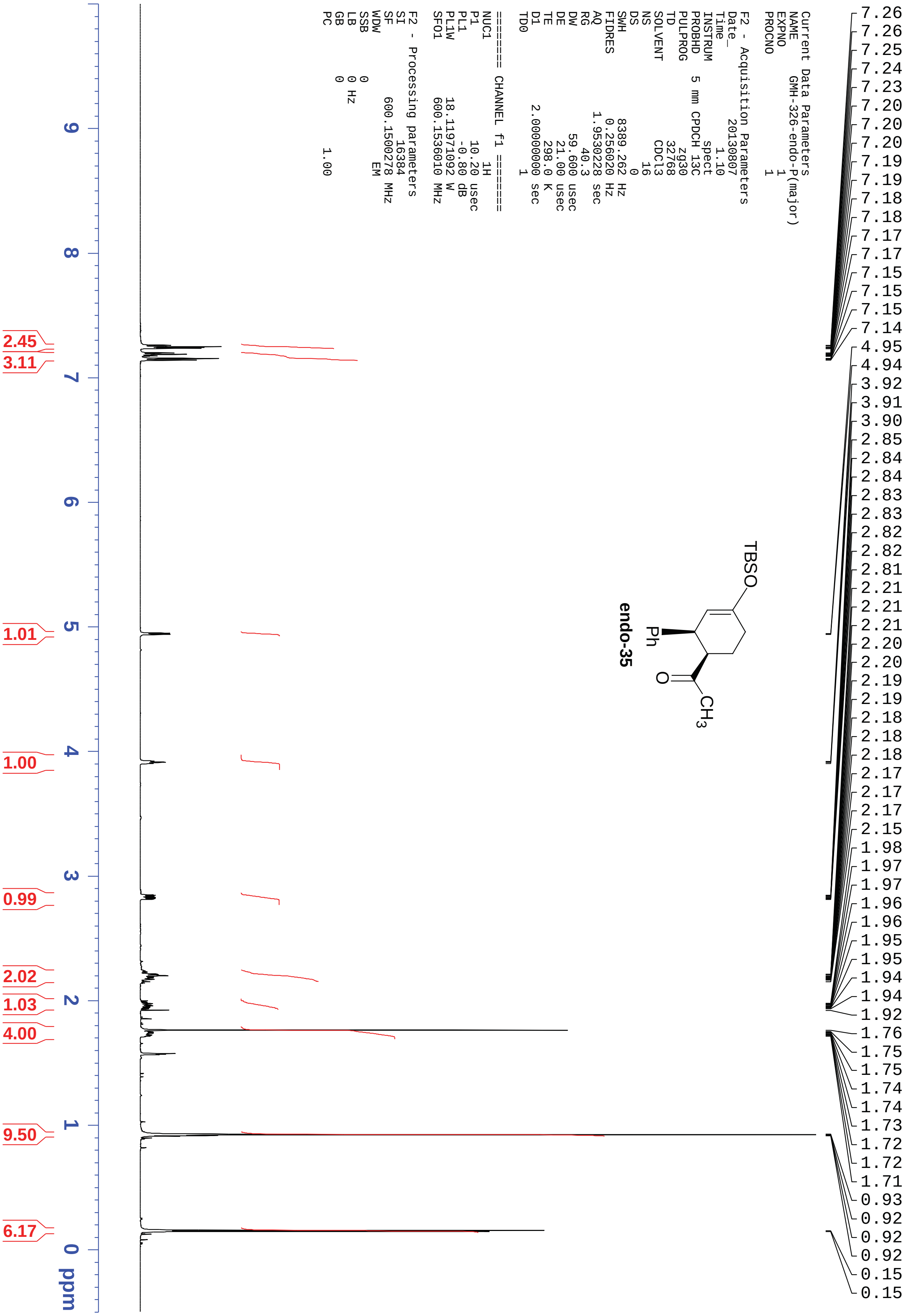

Supplementary Figure 103. ^{1}**H NMR spectrum of compound endo-35 in CDCl$_3$.**

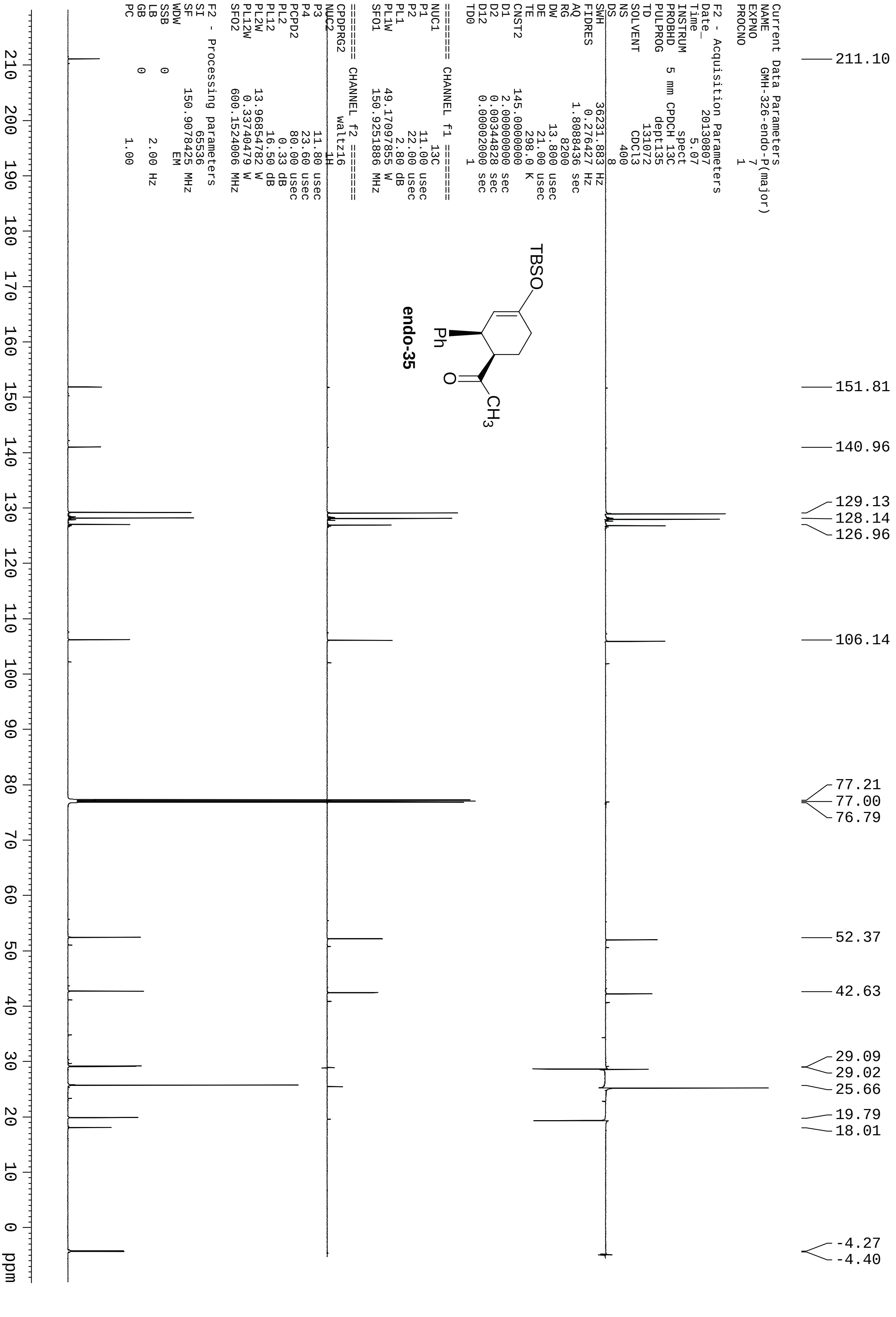

Supplementary Figure 104. ^{13}C and DEPT NMR spectra of compound endo-35 in $CDCl_3$.

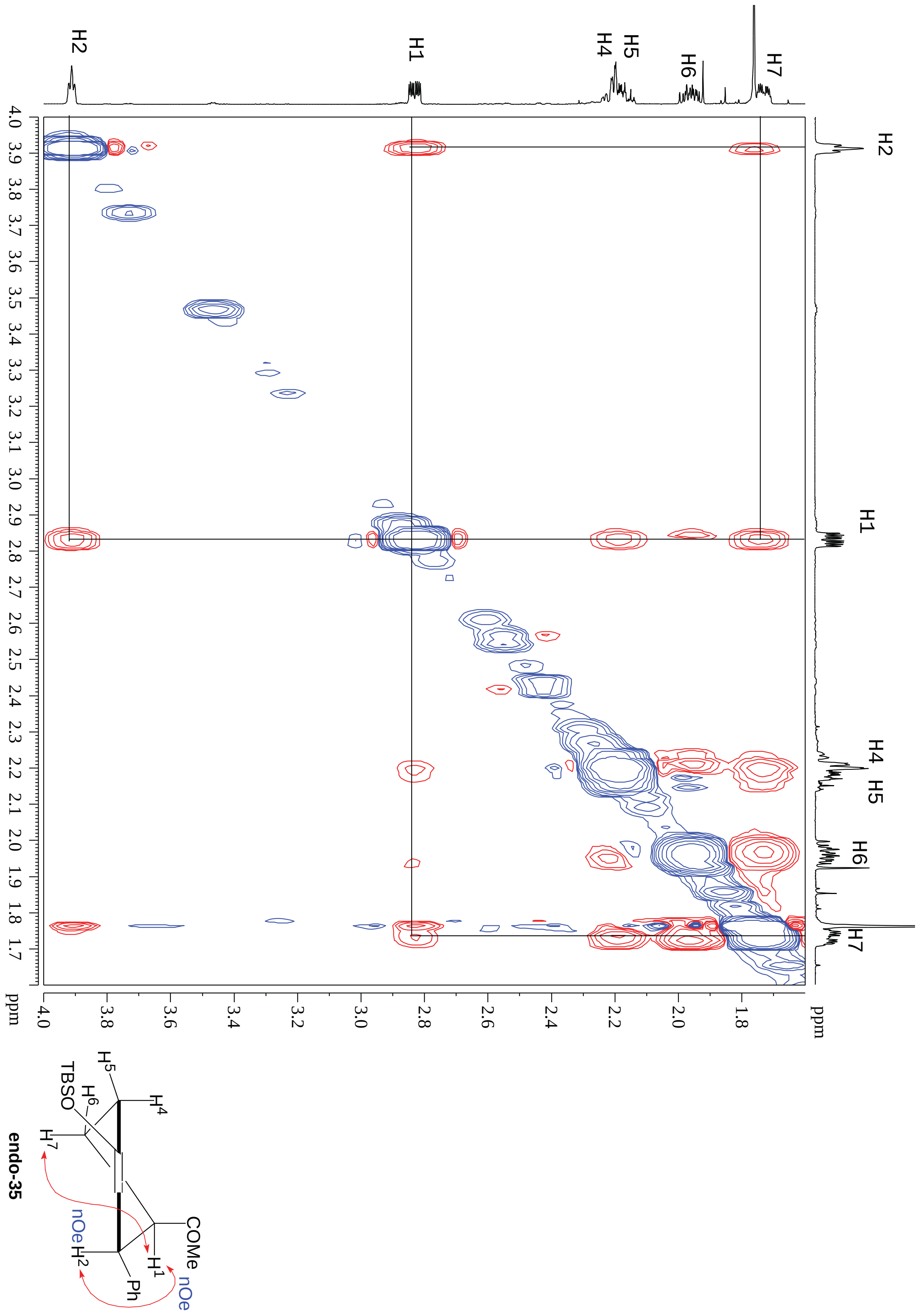

Supplementary Figure 105. NOESY NMR spectrum of compound endo-35 in $CDCl_3$.

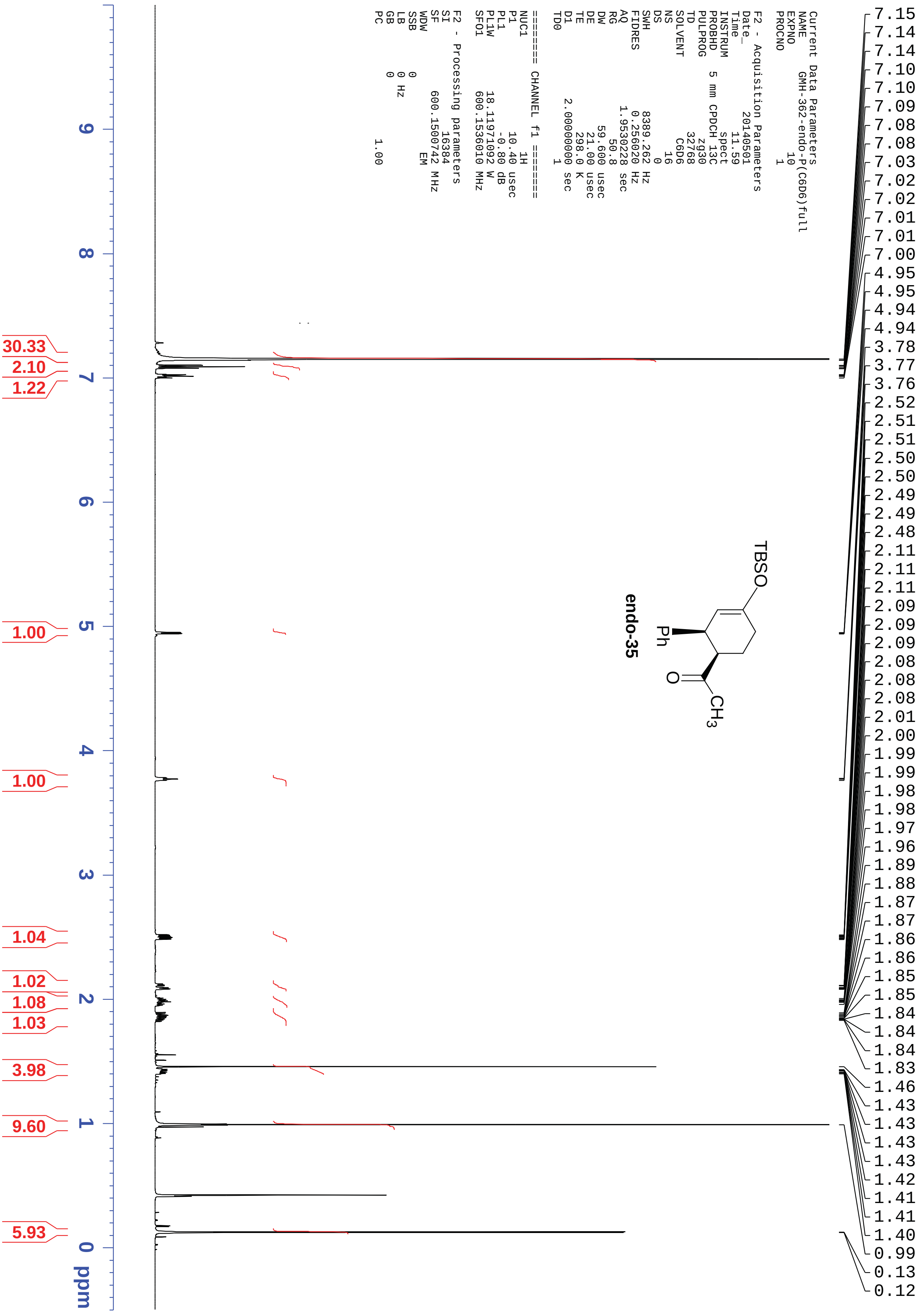

Supplementary Figure 106. ^{1}H NMR spectrum of compound endo-35 in C_6D_6.

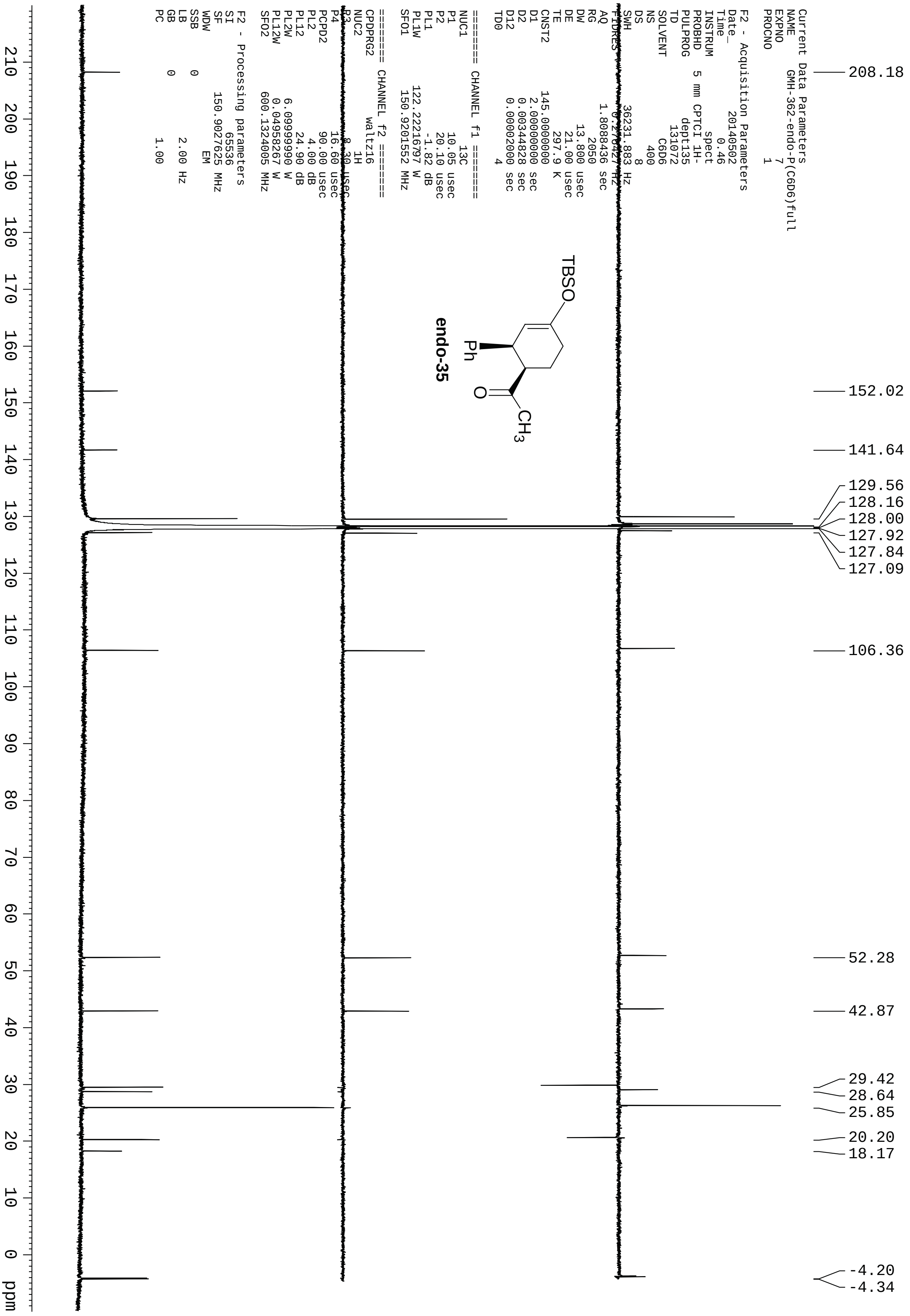

Supplementary Figure 107. ^{13}C and DEPT NMR spectra of compound endo-35 in C_6D_6.

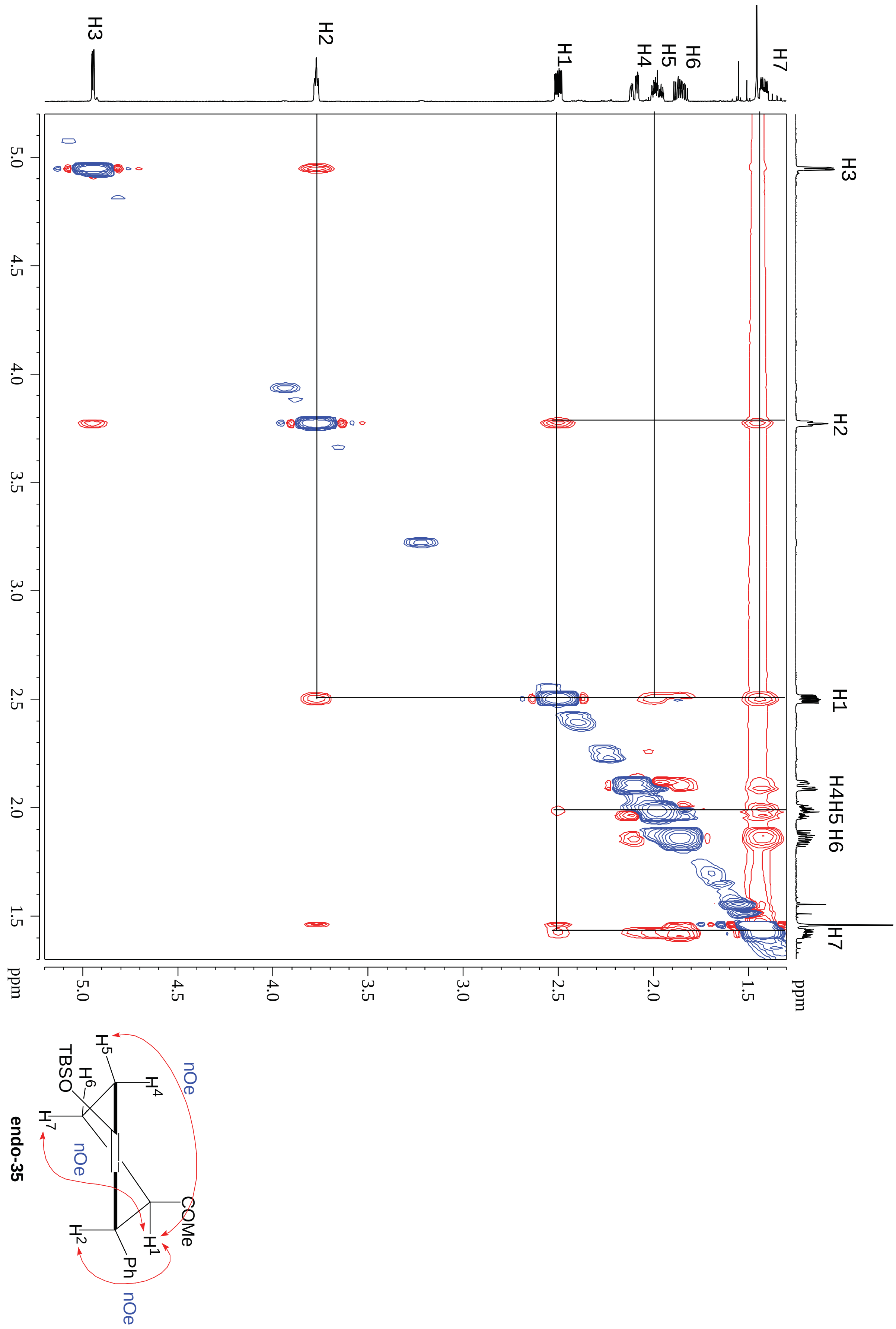

Supplementary Figure 108. NOESY NMR spectrum of compound endo-35 in C_6D_6.

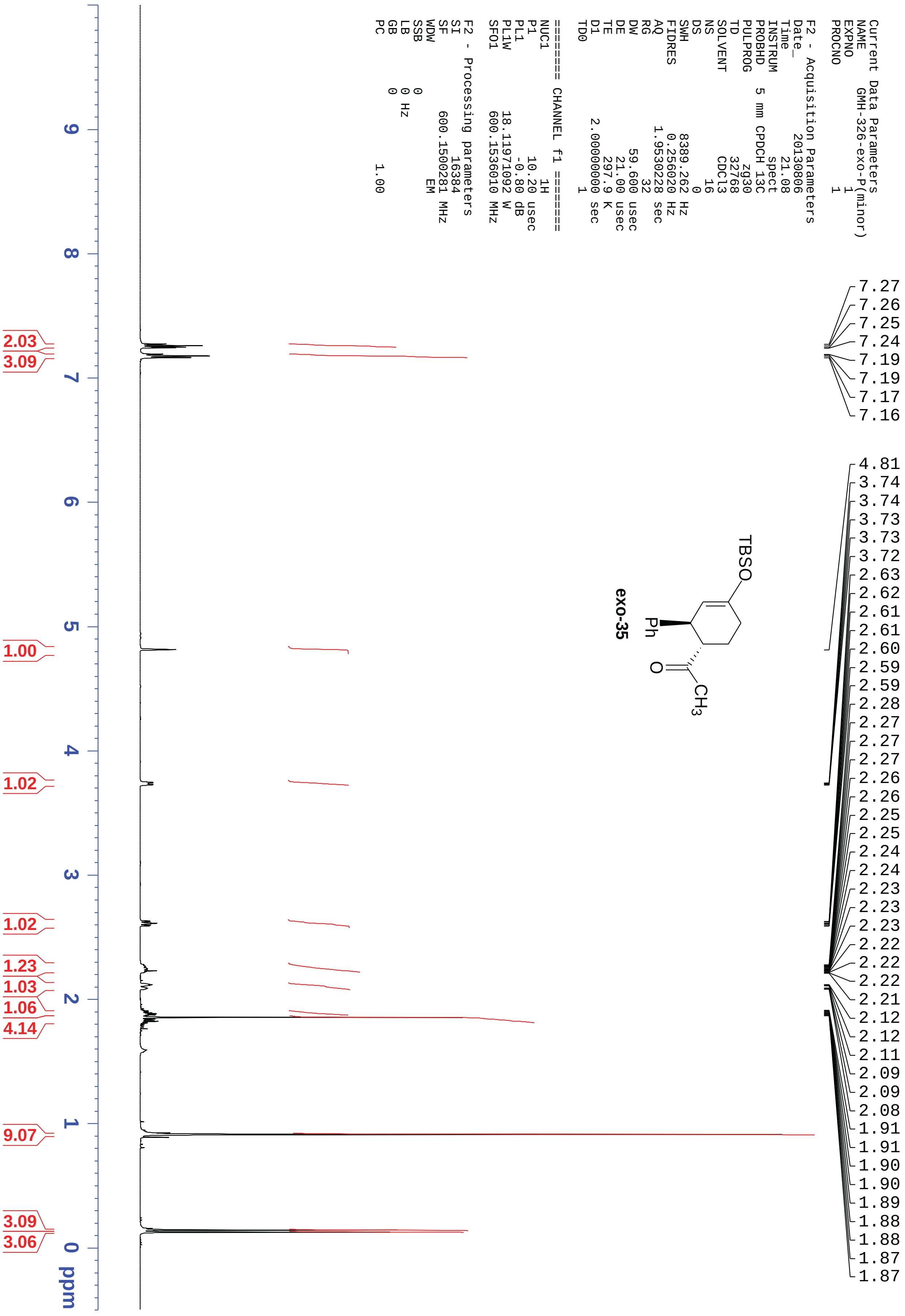

Supplementary Figure 109. **¹H NMR spectrum of compound exo-35.**

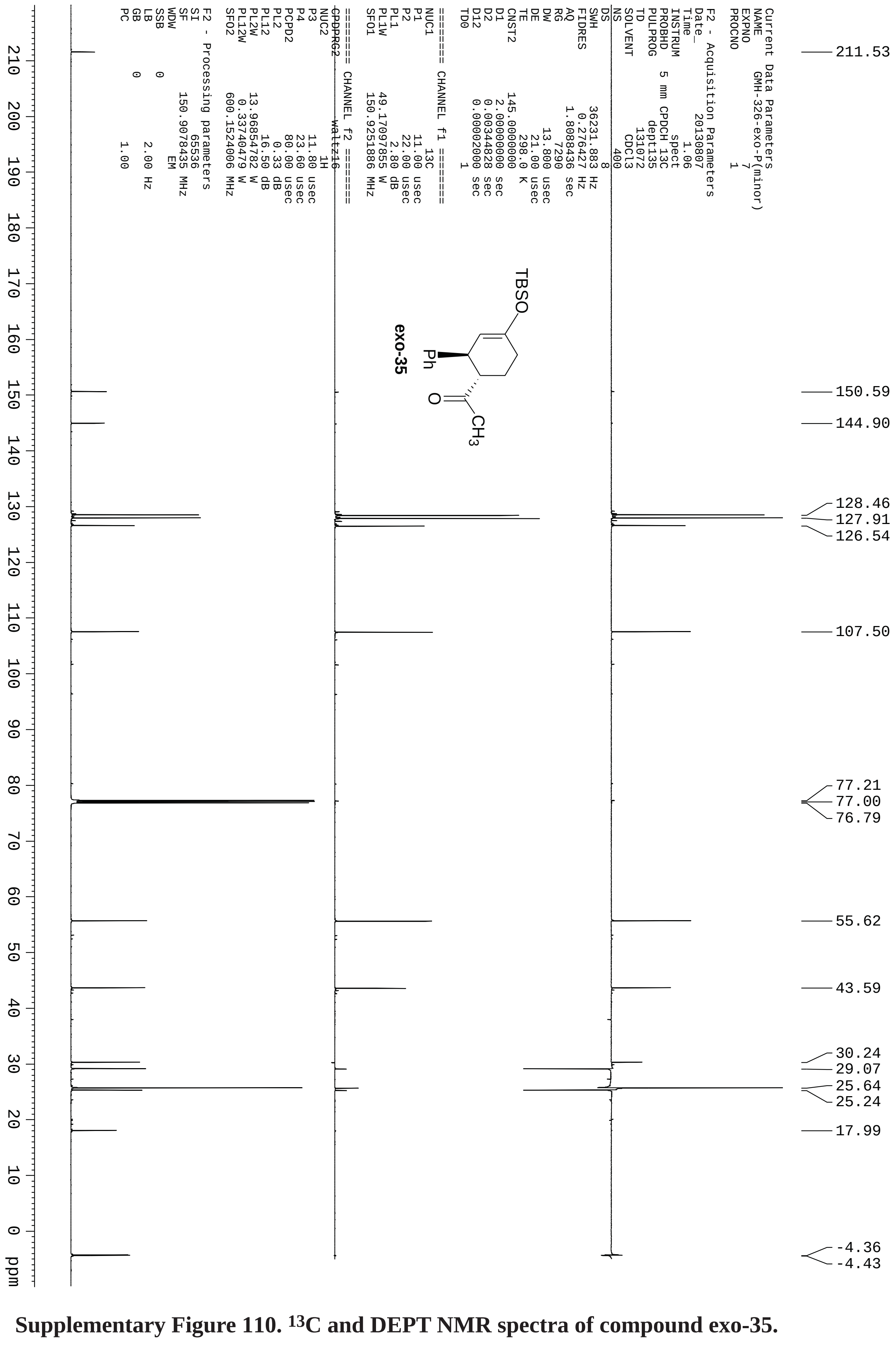

Supplementary Figure 110. ^{13}C and DEPT NMR spectra of compound exo-35.

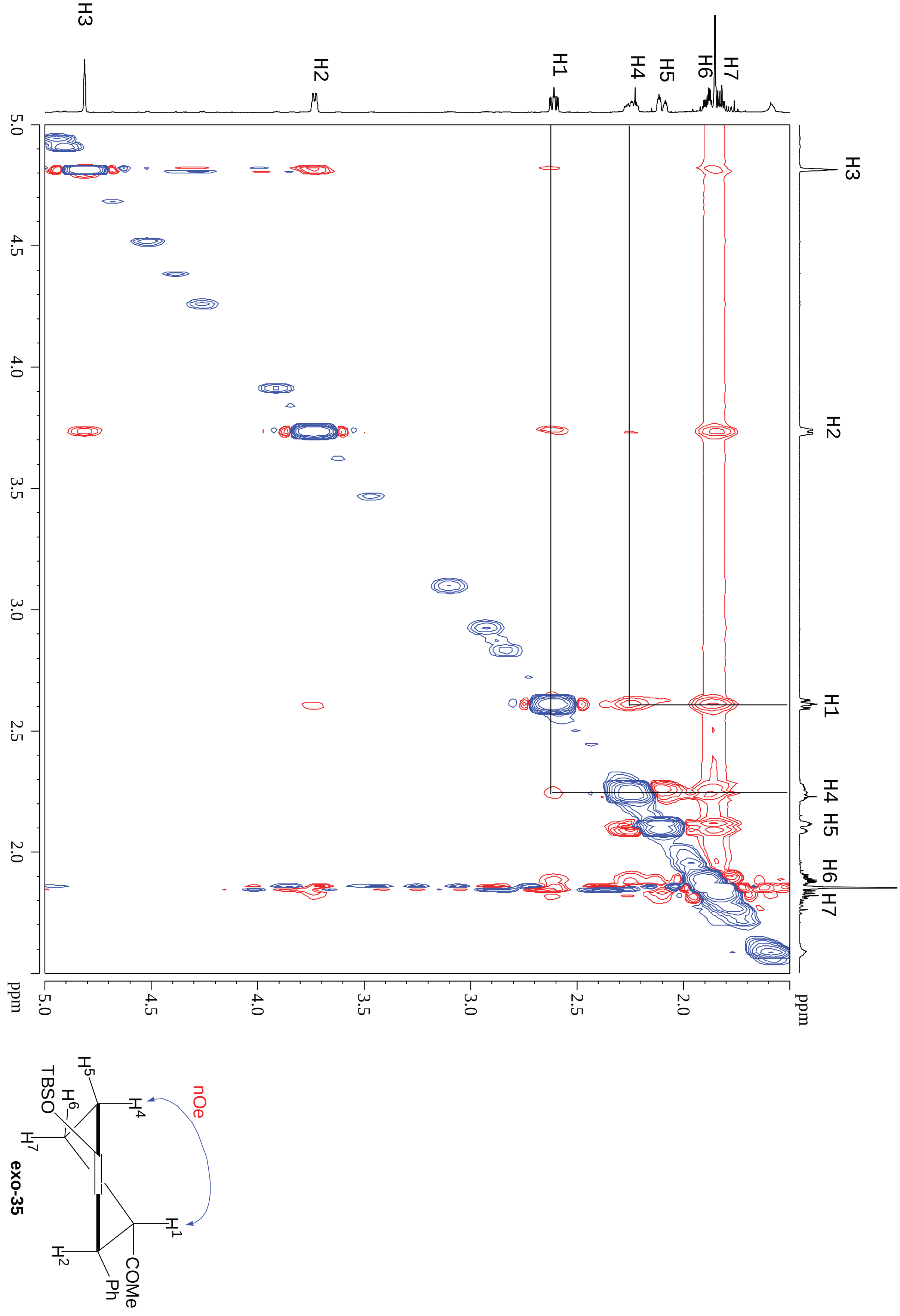

Supplementary Figure 111. NOESY NMR spectrum of compound exo-35.

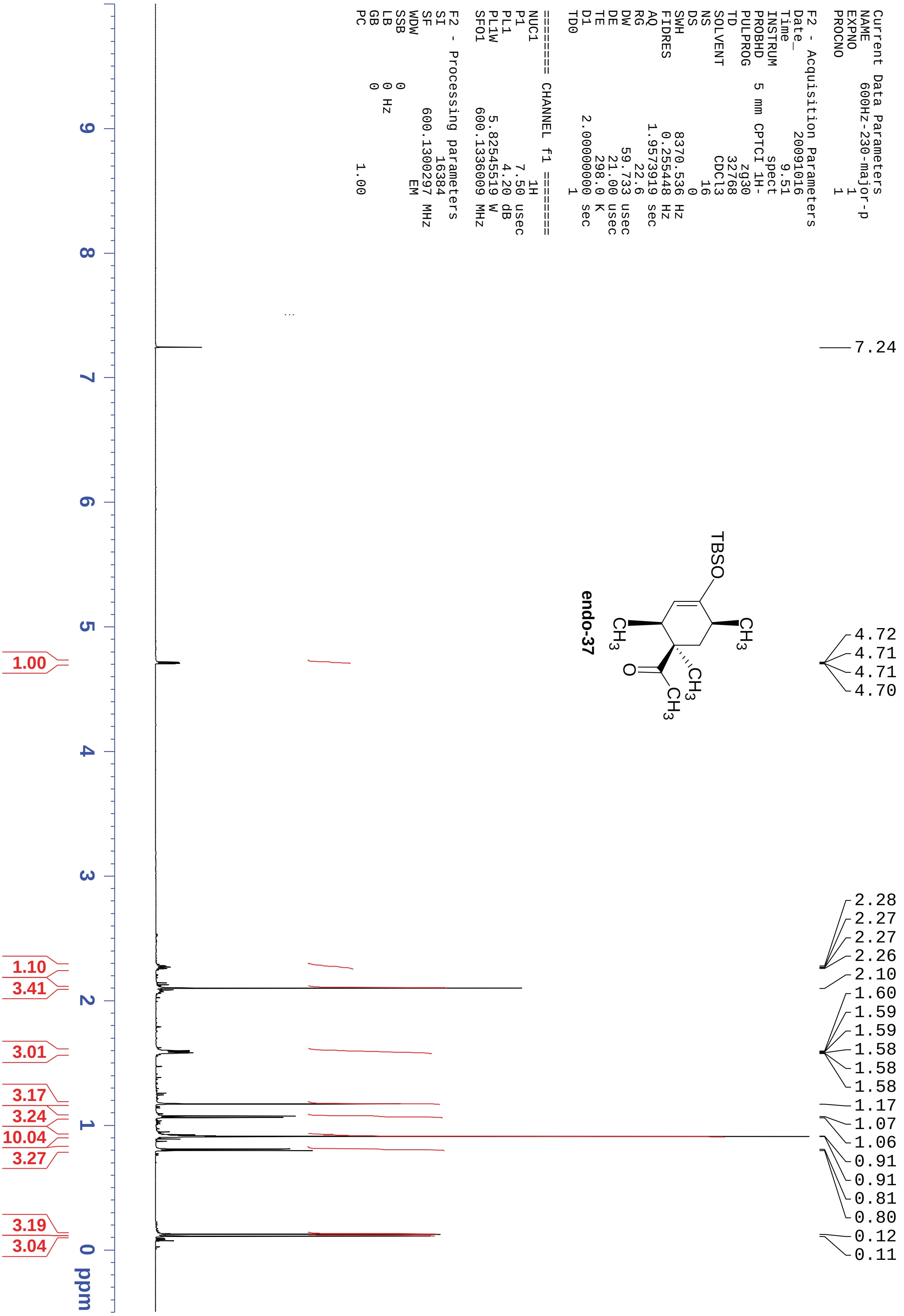

Supplementary Figure 112. **^{1}H NMR spectrum of compound endo-37 in $CDCl_3$.**

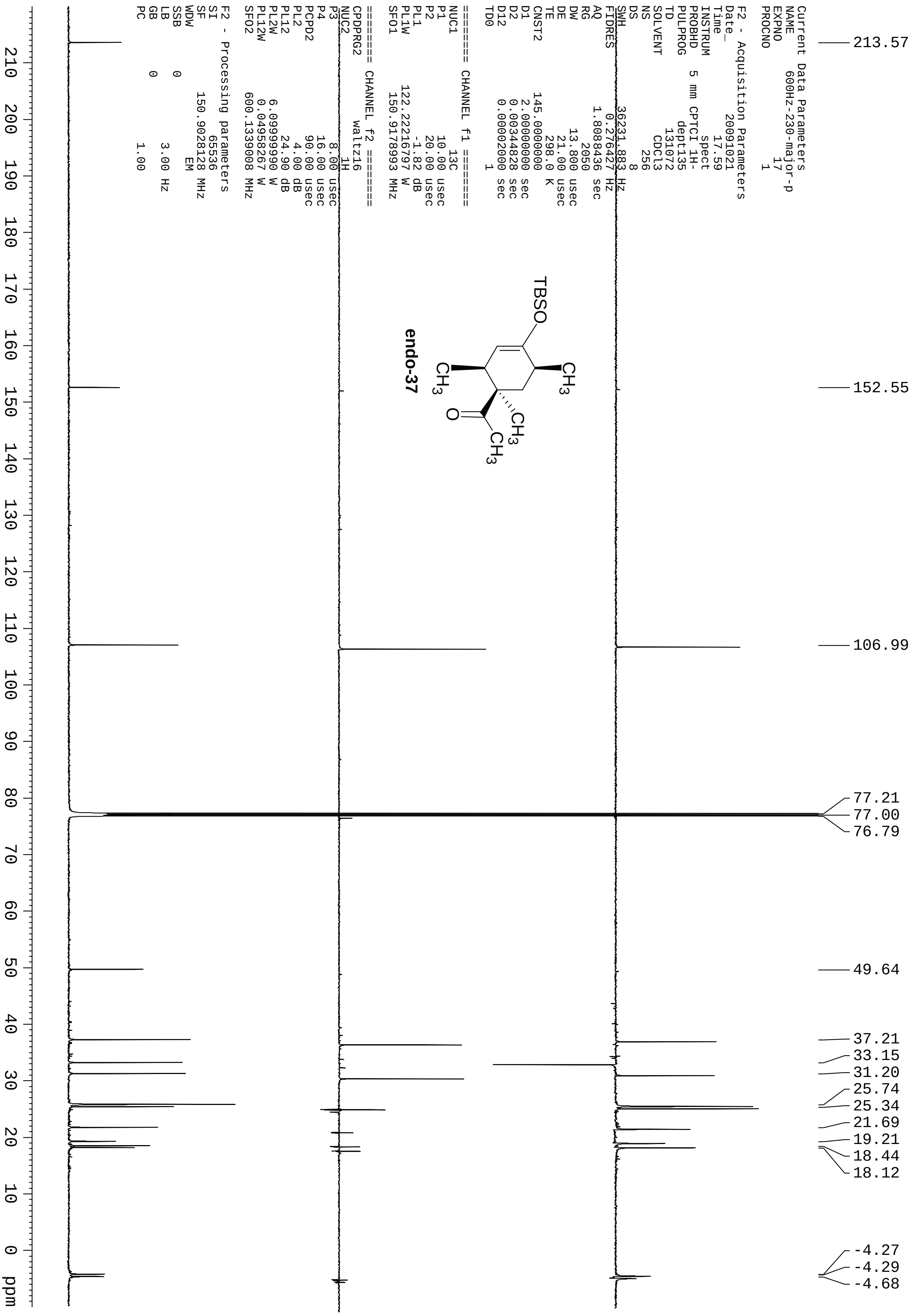

Supplementary Figure 113. ^{13}C and DEPT NMR spectra of compound endo-37 in $CDCl_3$.

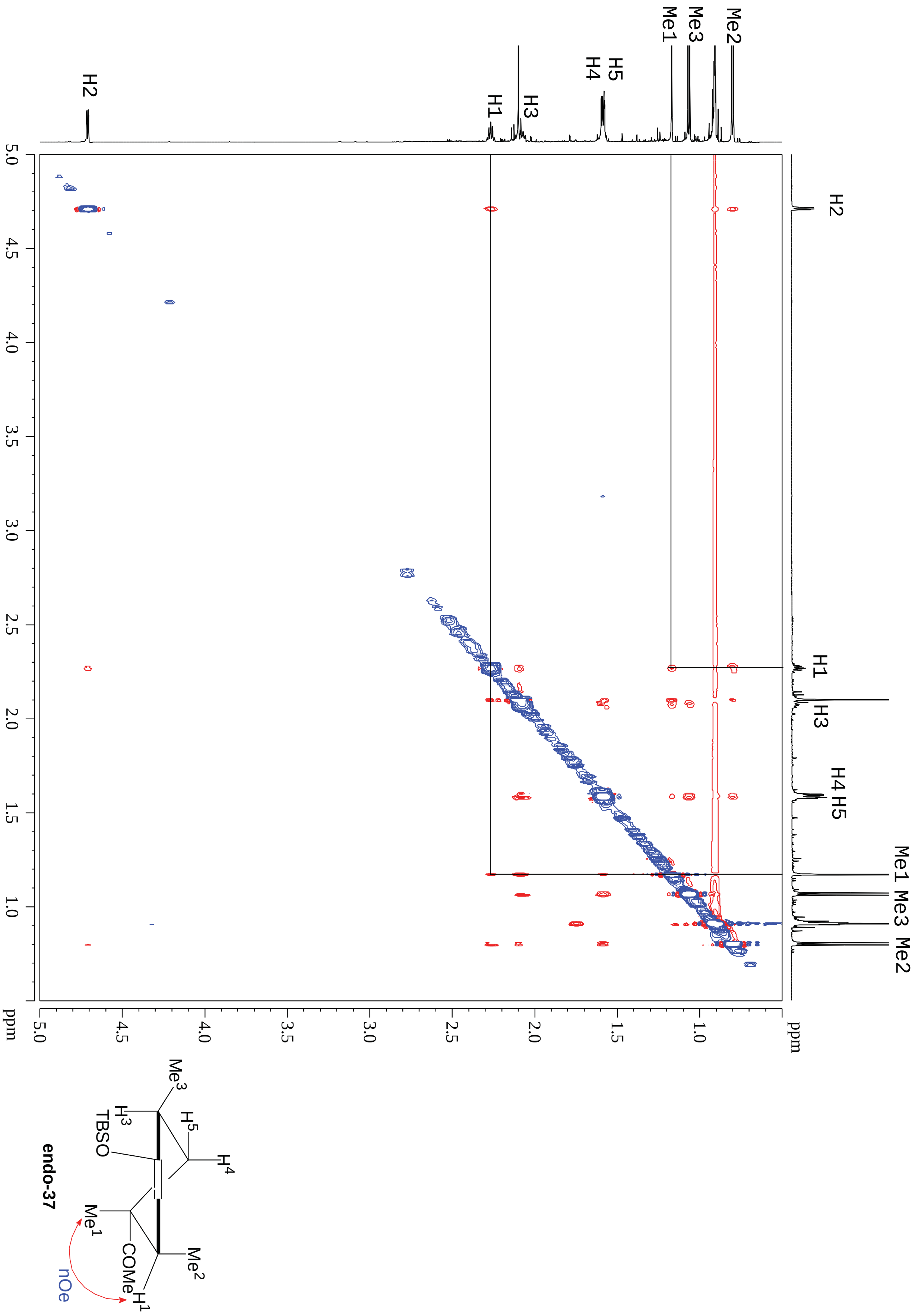

Supplementary Figure 114. NOESY NMR spectrum of compound endo-37 in $CDCl_3$.

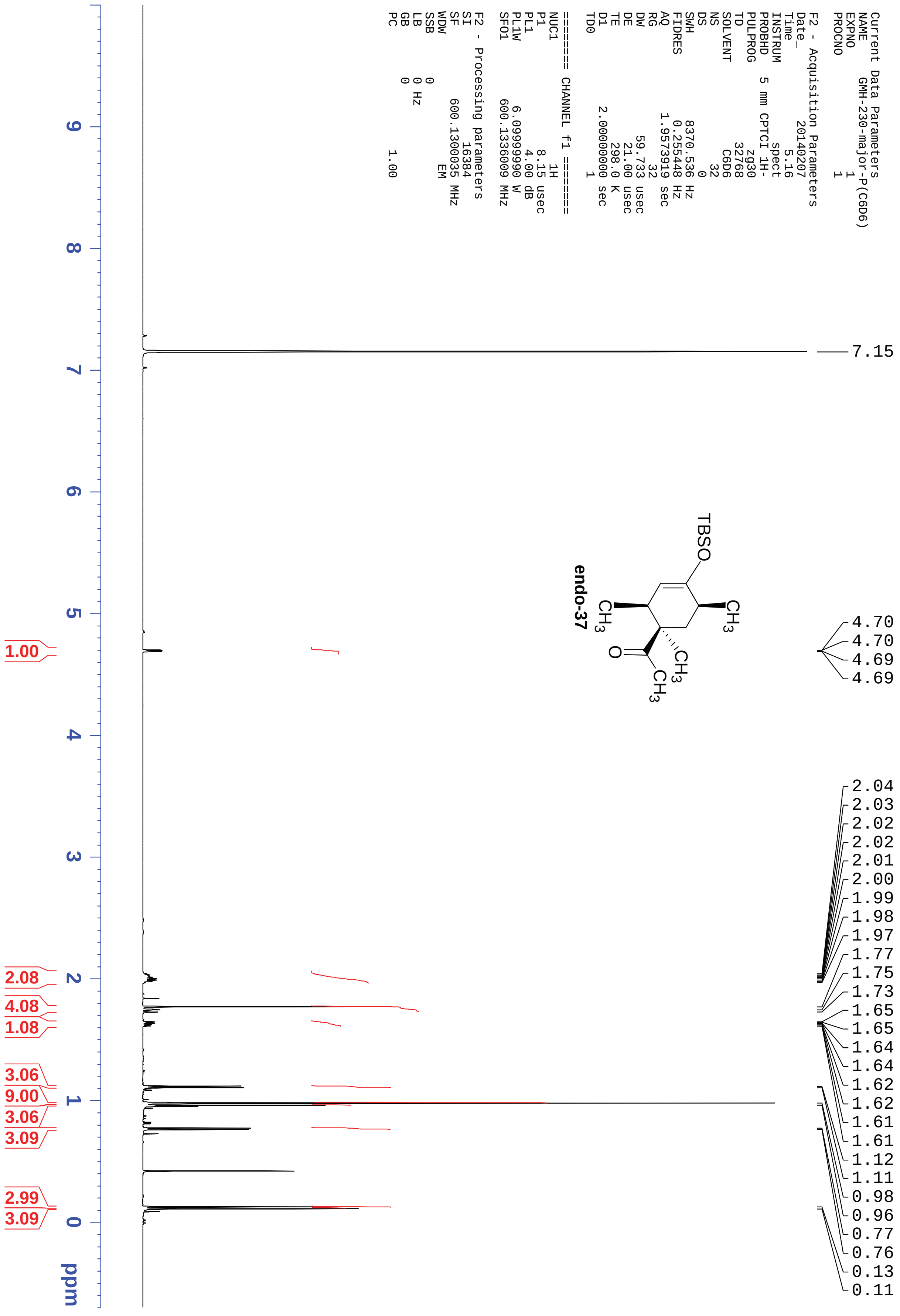

Supplementary Figure 115. **^{1}H NMR spectrum of compound endo-37 in C_6D_6.**

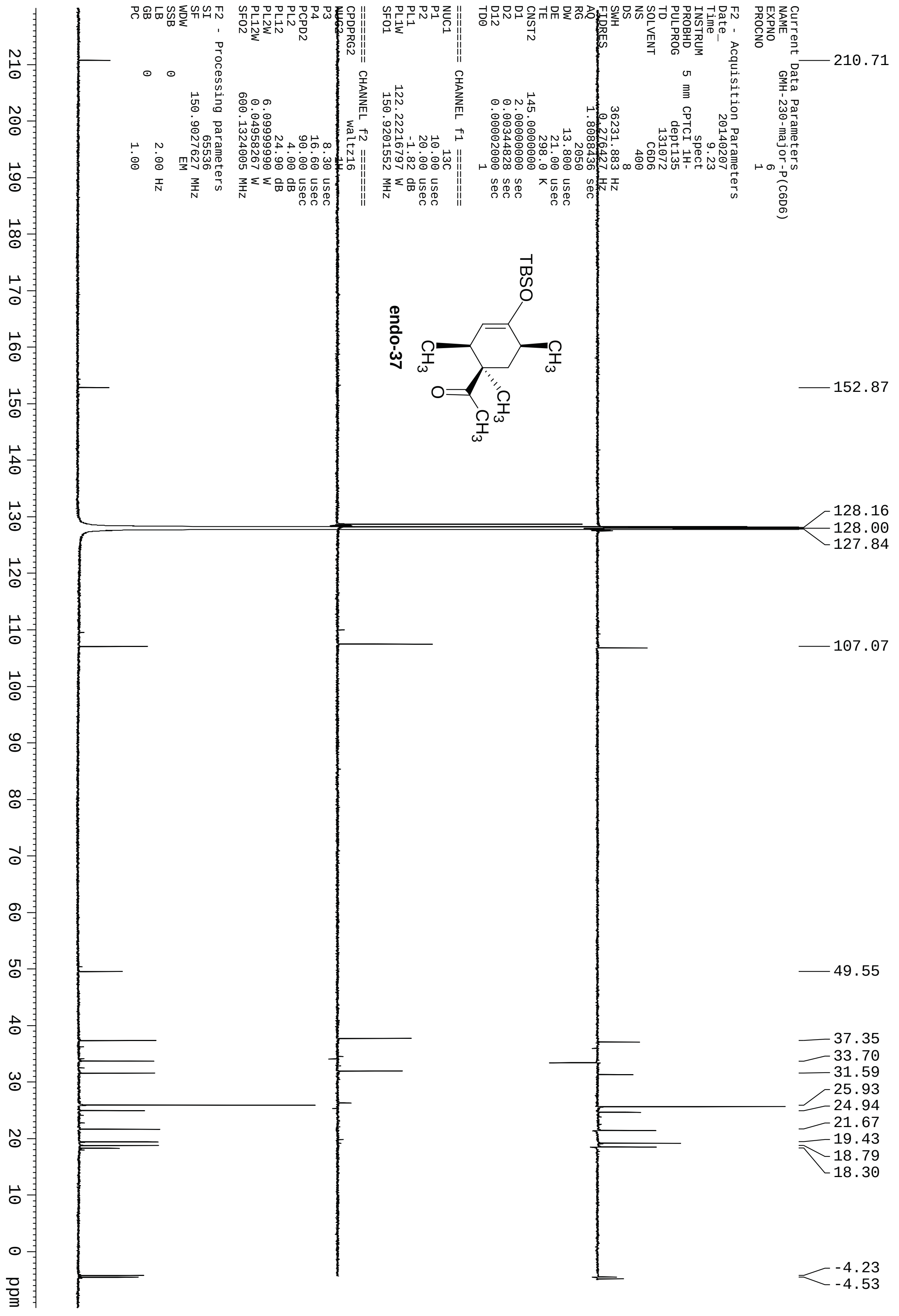

Supplementary Figure 116. ^{13}C and DEPT NMR spectra of compound endo-37 in C_6D_6.

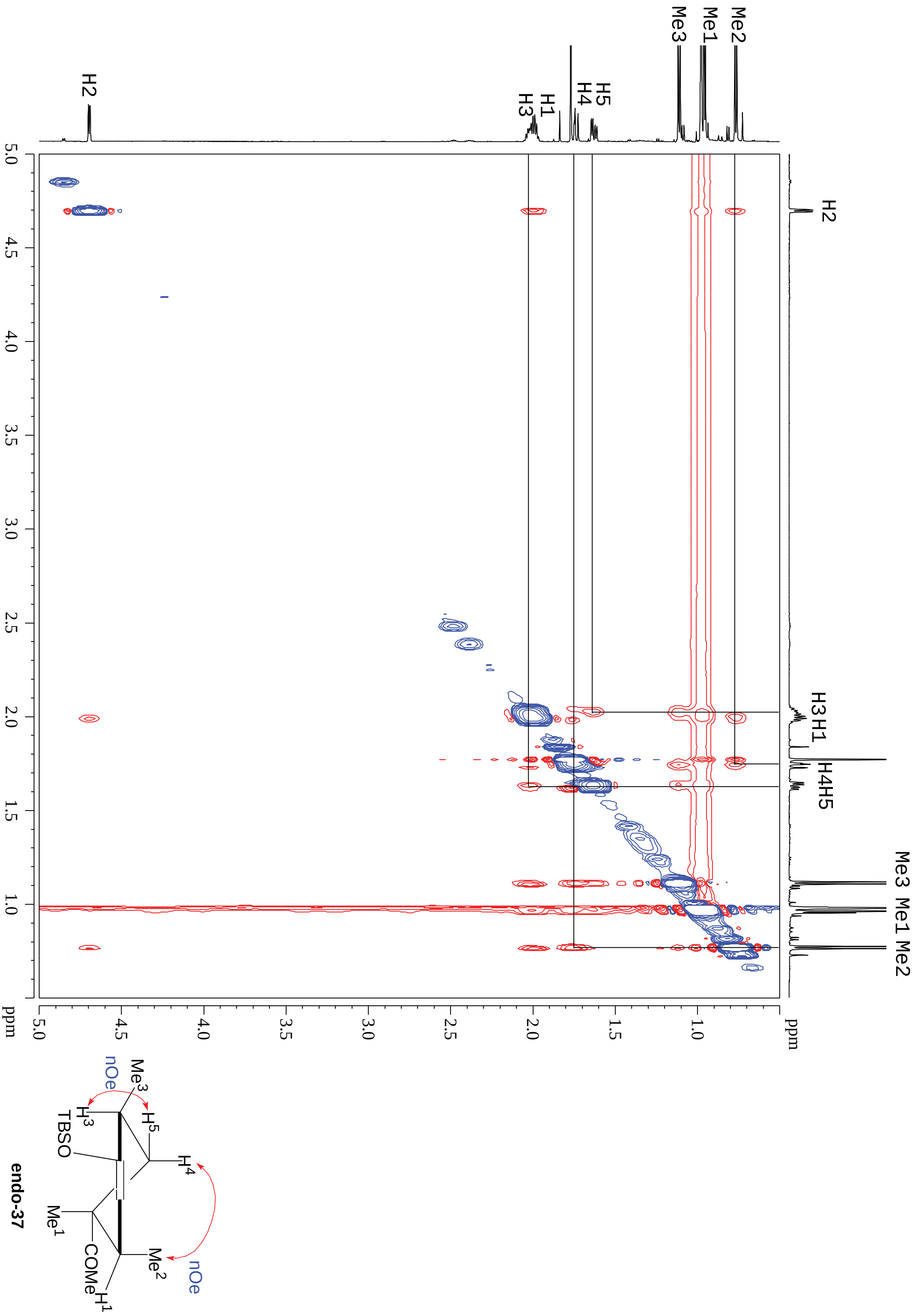

Supplementary Figure 117. NOESY NMR spectrum of compound endo-37 in C_6D_6.

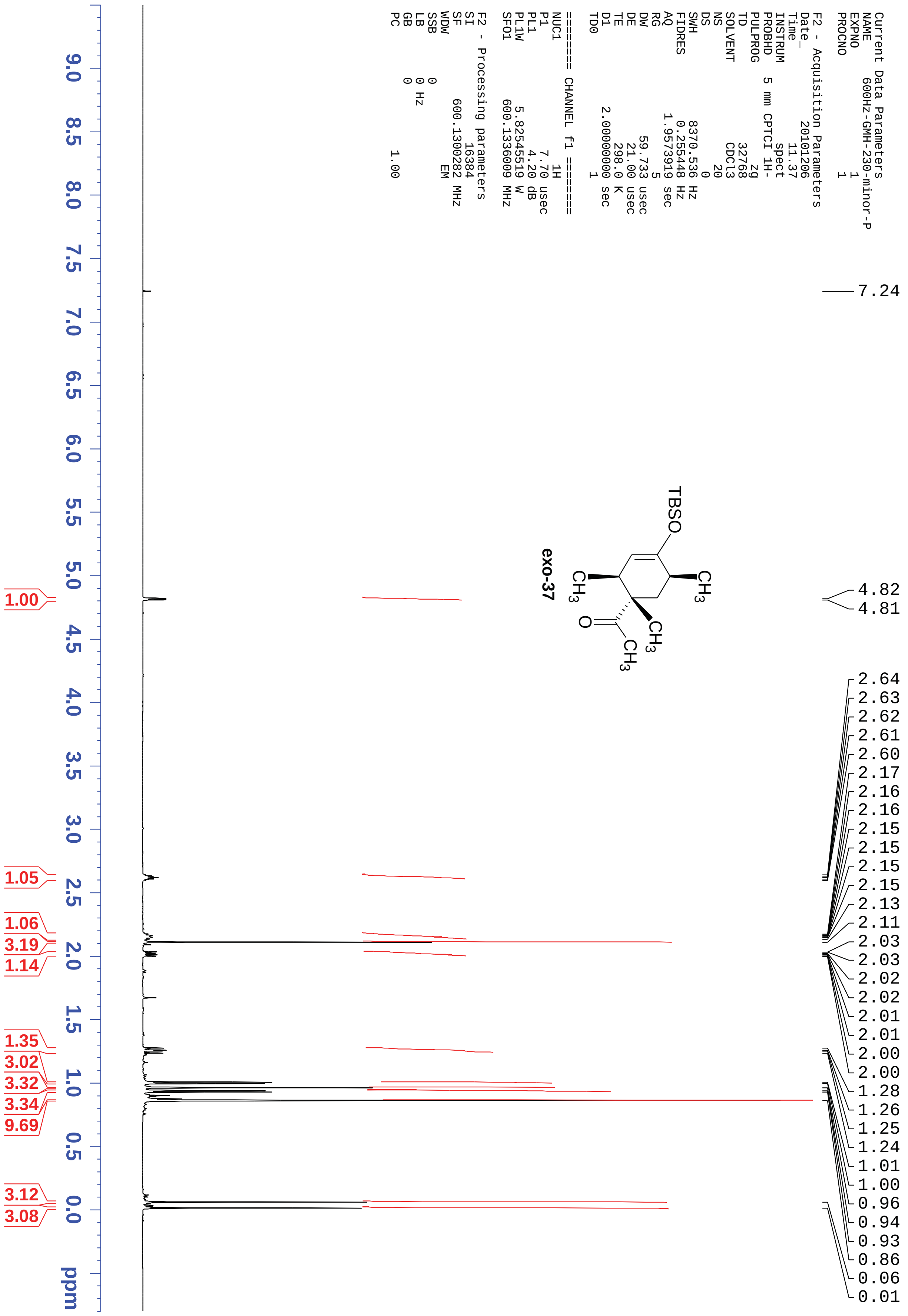

Supplementary Figure 118. ^{1}**H NMR spectrum of compound exo-37.**

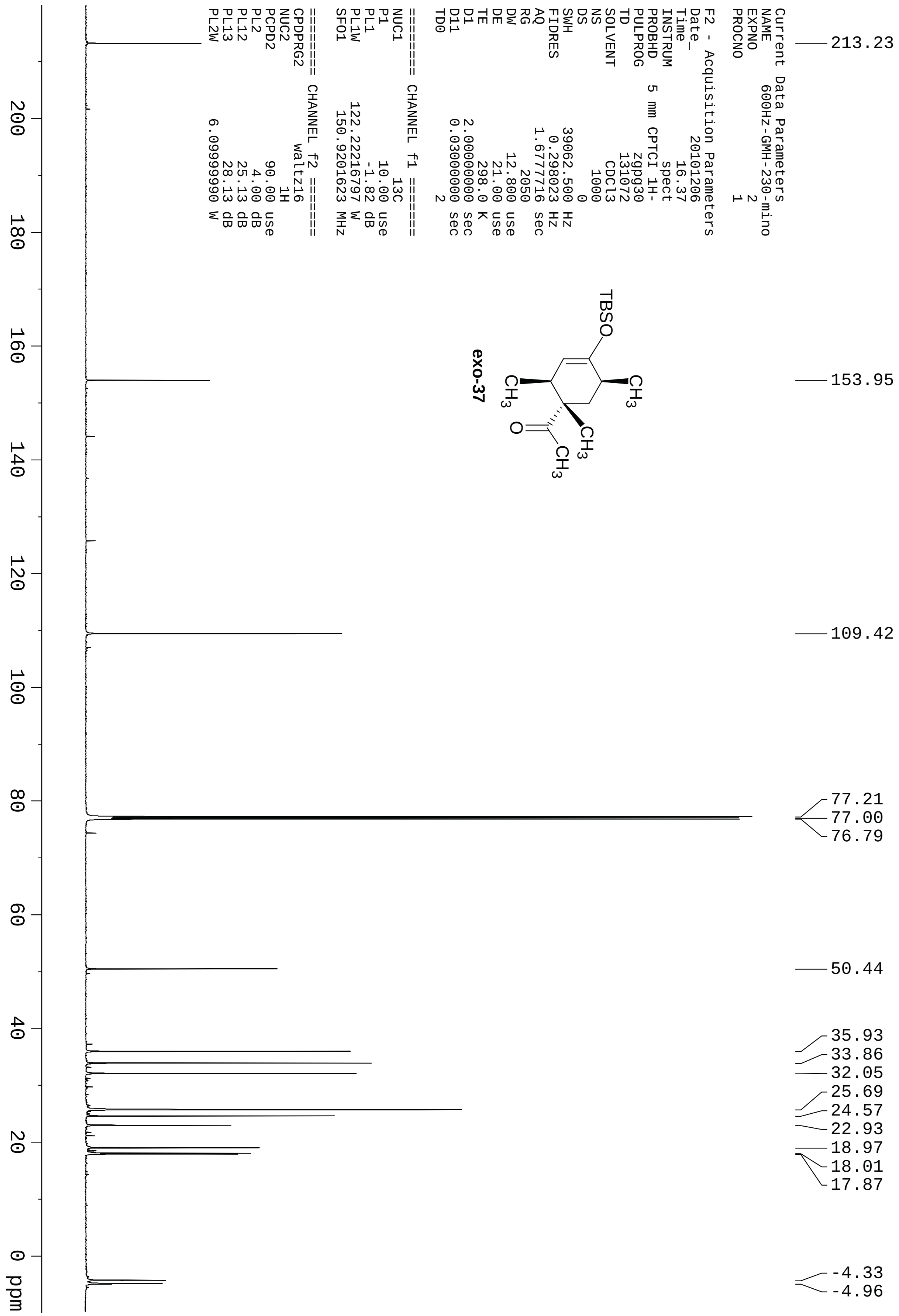

Supplementary Figure 119. ^{13}C NMR spectrum of compound exo-37.

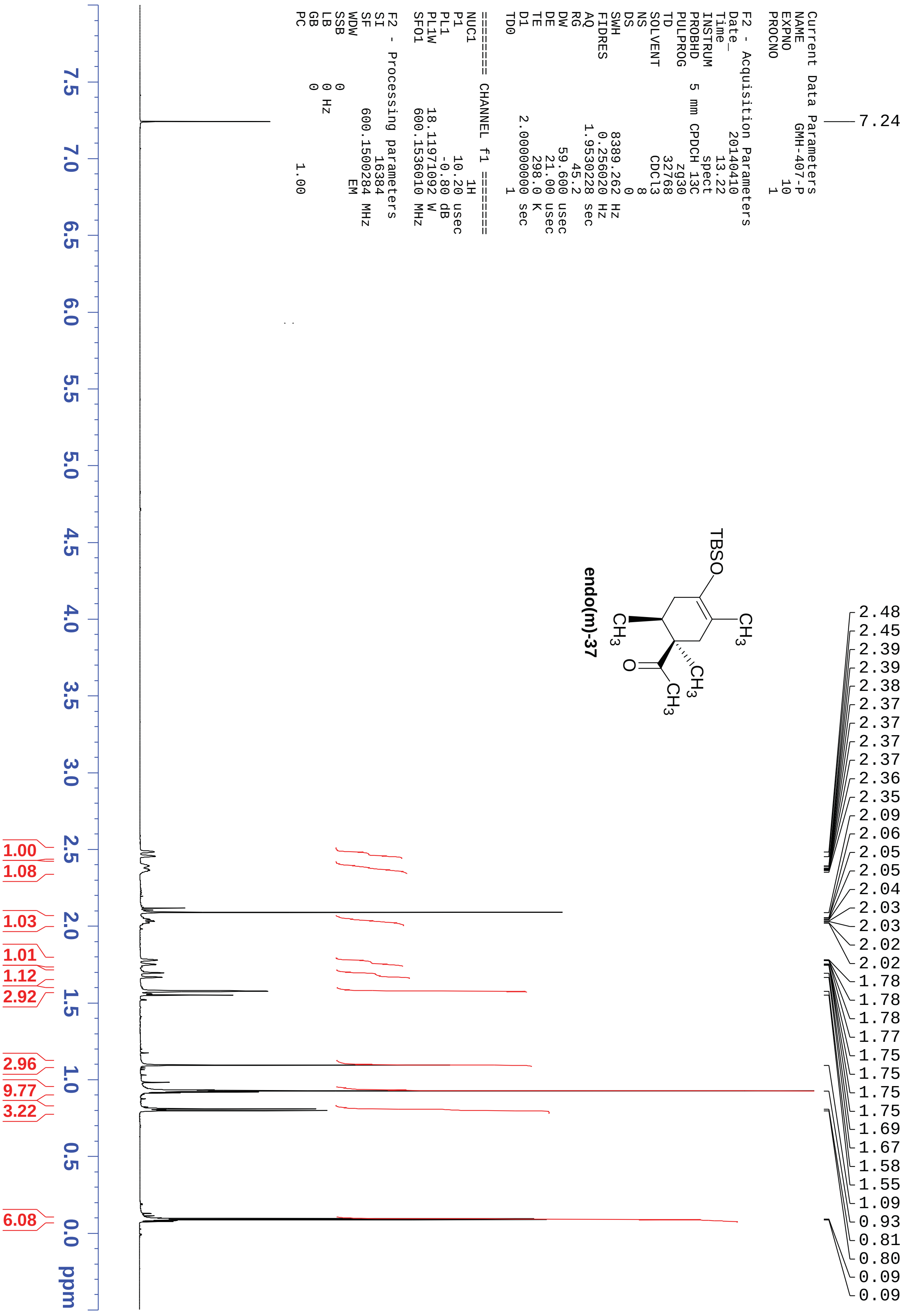

Supplementary Figure 120. ^{1}**H NMR spectrum of compound endo(m)-37.**

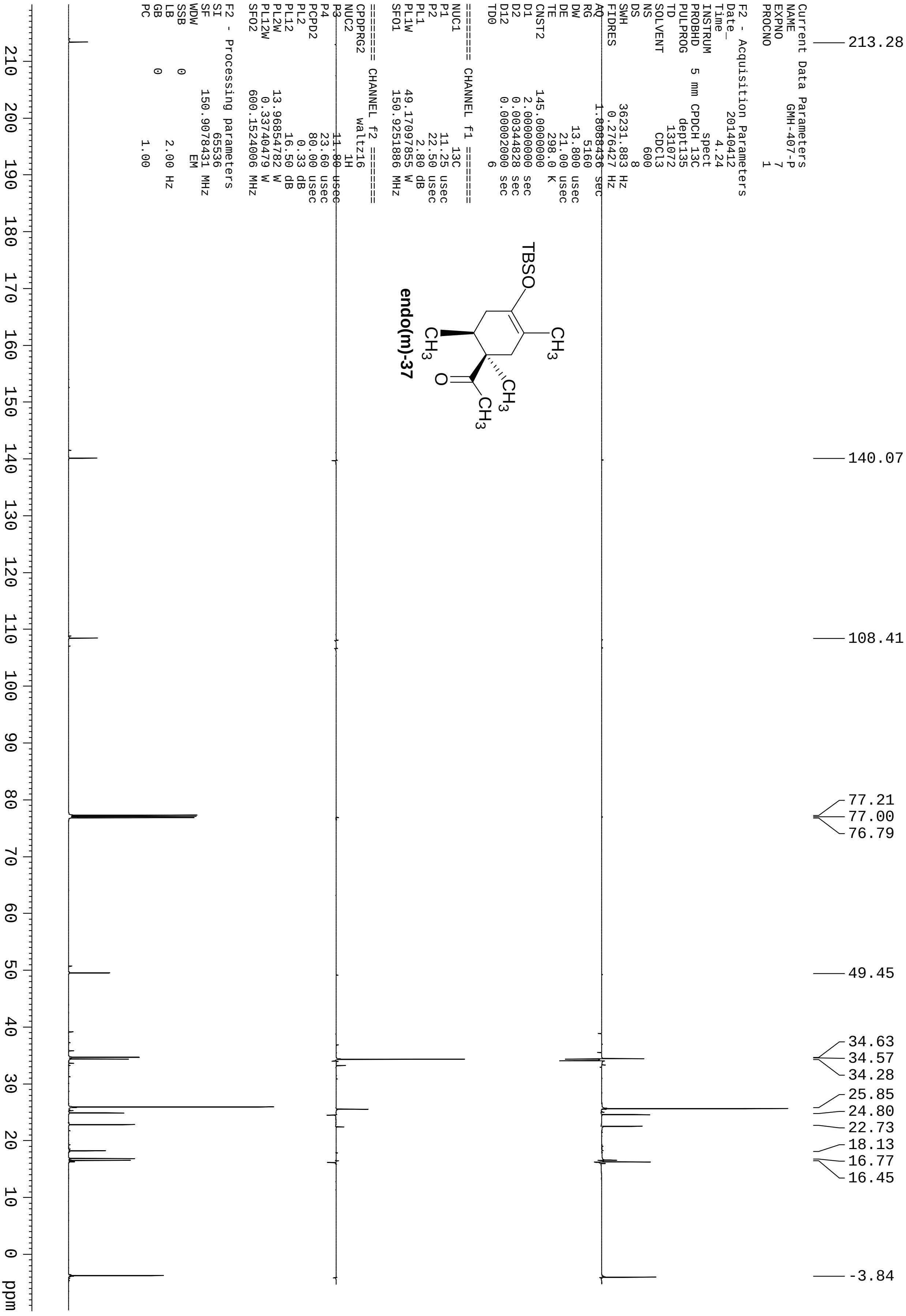

Supplementary Figure 121. ^{13}C and DEPT NMR spectra of compound endo(m)-37.

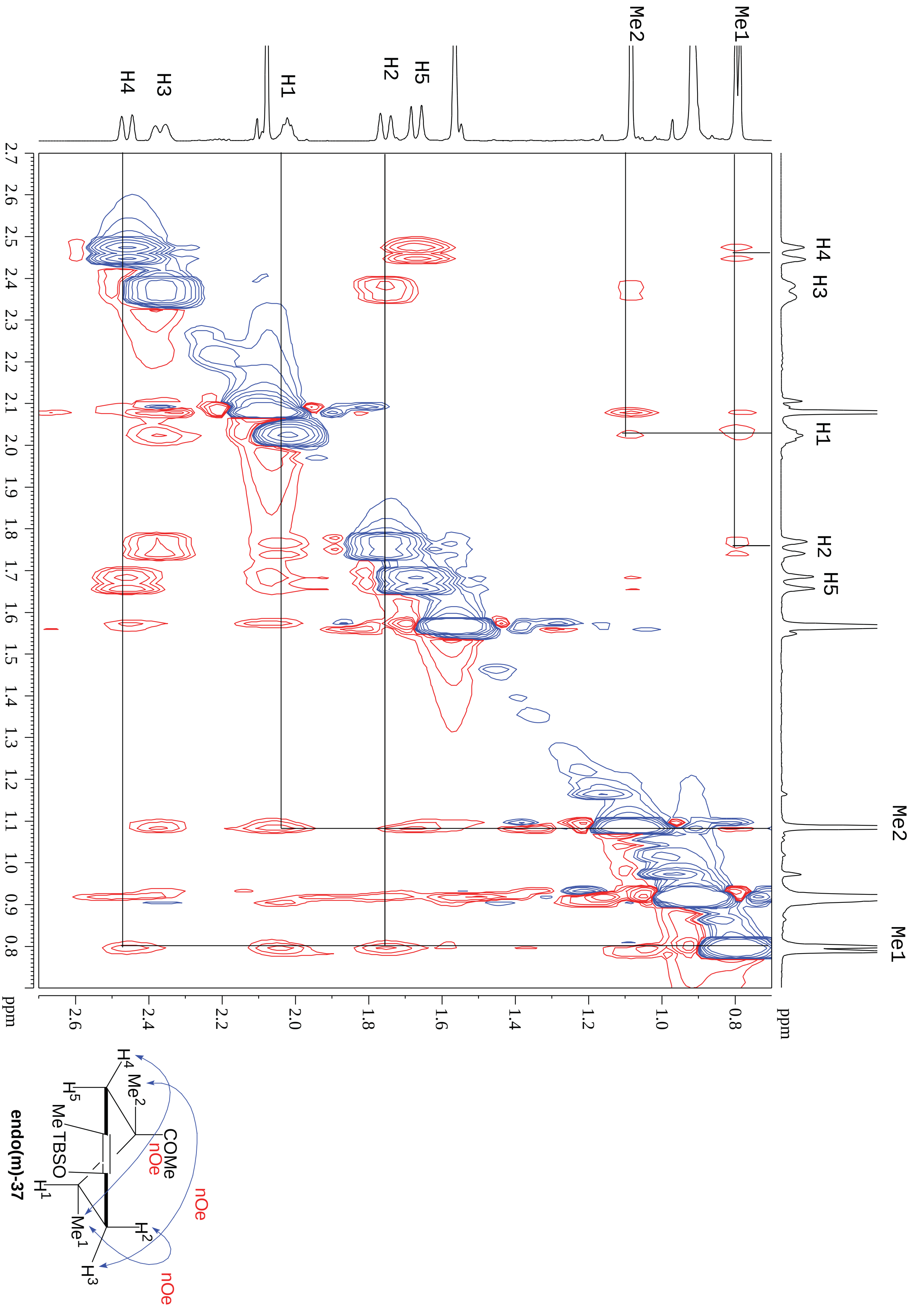

Supplementary Figure 122. NOESY NMR spectrum of compound endo(m)-37.

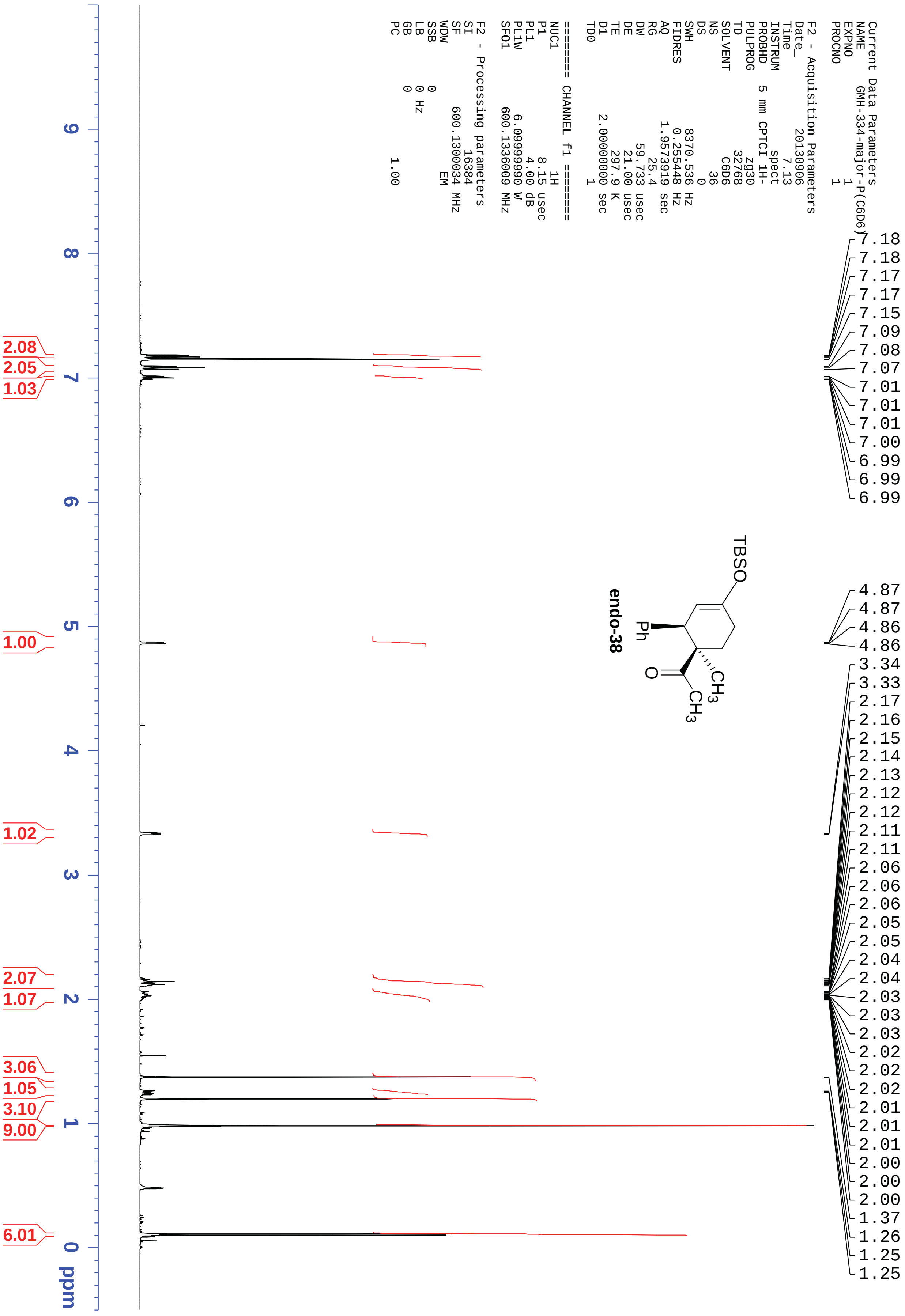

Supplementary Figure 123. ^{1}H NMR spectrum of compound endo-38 in C_6D_6.

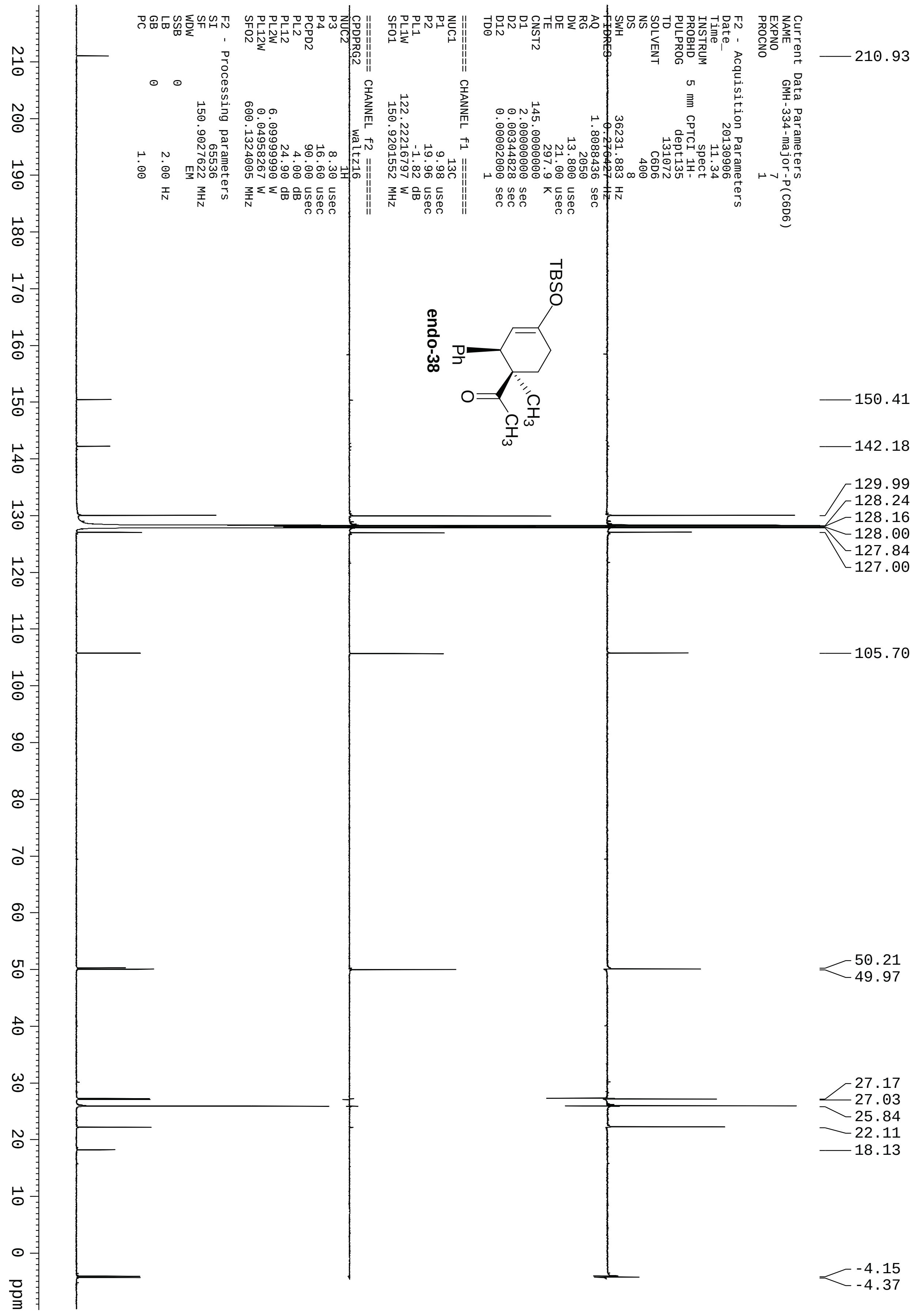

Supplementary Figure 124. ^{13}C and DEPT NMR spectra of compound endo-38 in C_6D_6.

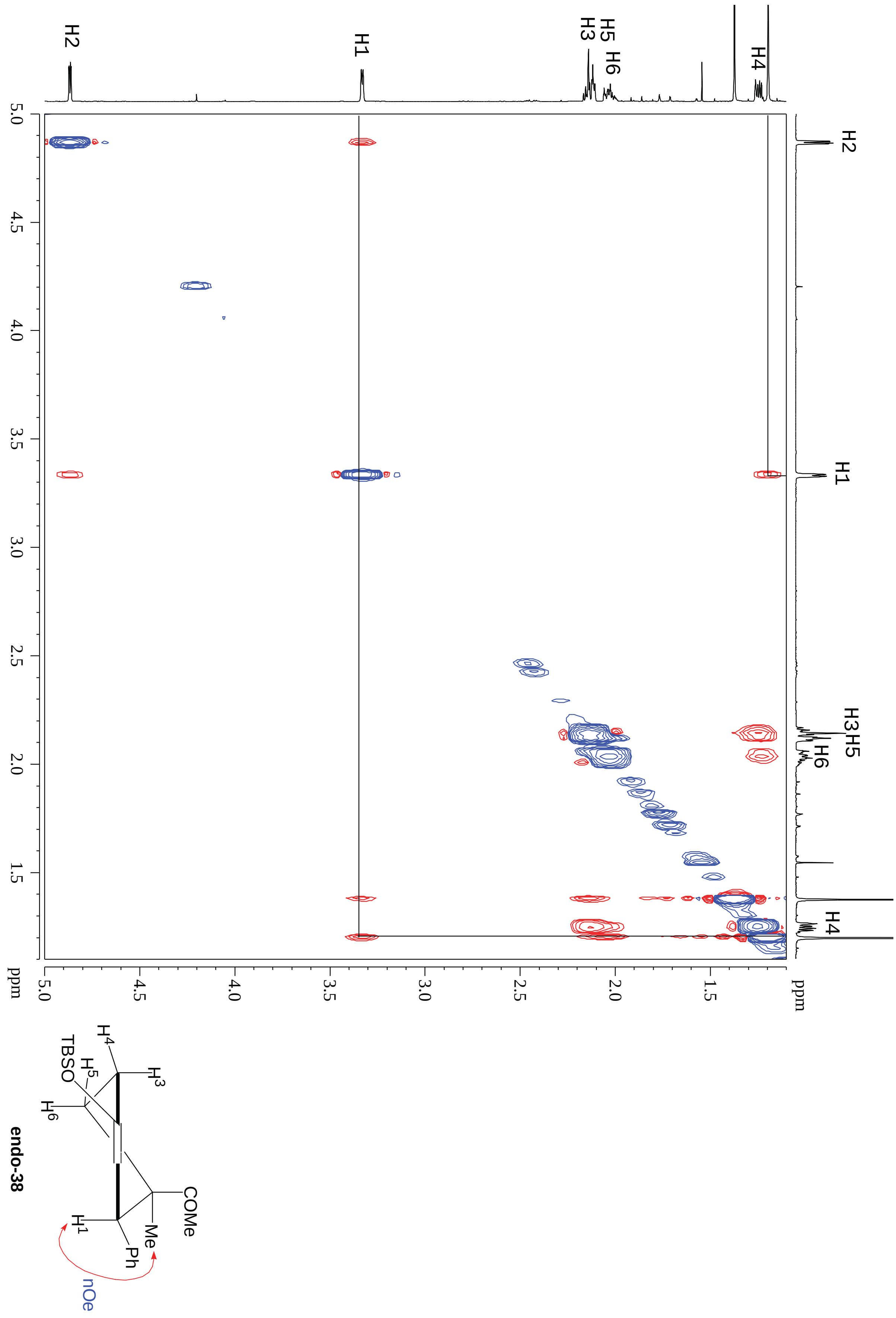

Supplementary Figure 125. NOESY NMR spectrum of compound endo-38 in C_6D_6.

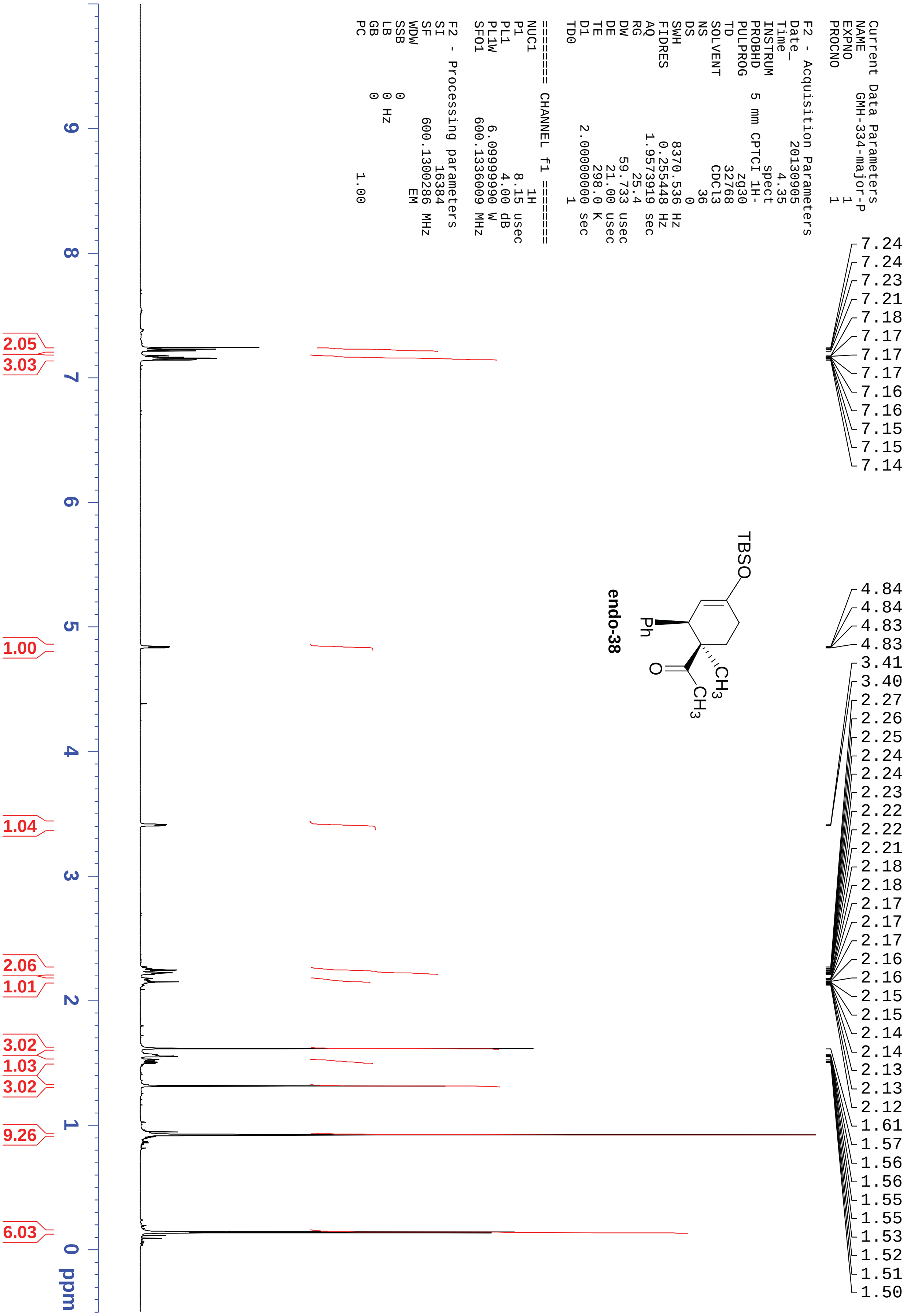

Supplementary Figure 126. **^{1}H NMR spectrum of compound endo-38 in $CDCl_3$.**

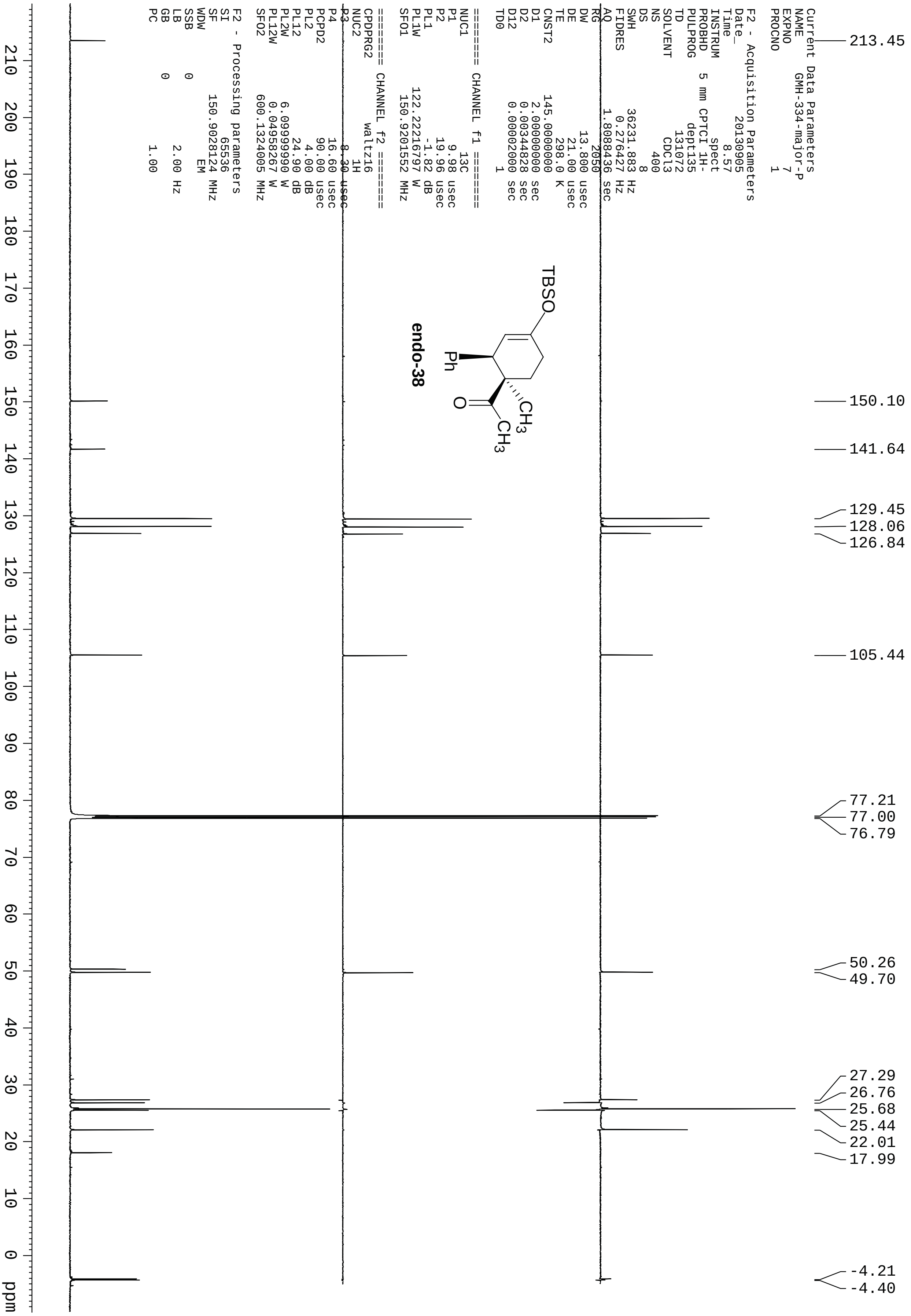

Supplementary Figure 127. ^{13}C and DEPT NMR spectra of compound endo-38 in $CDCl_3$.

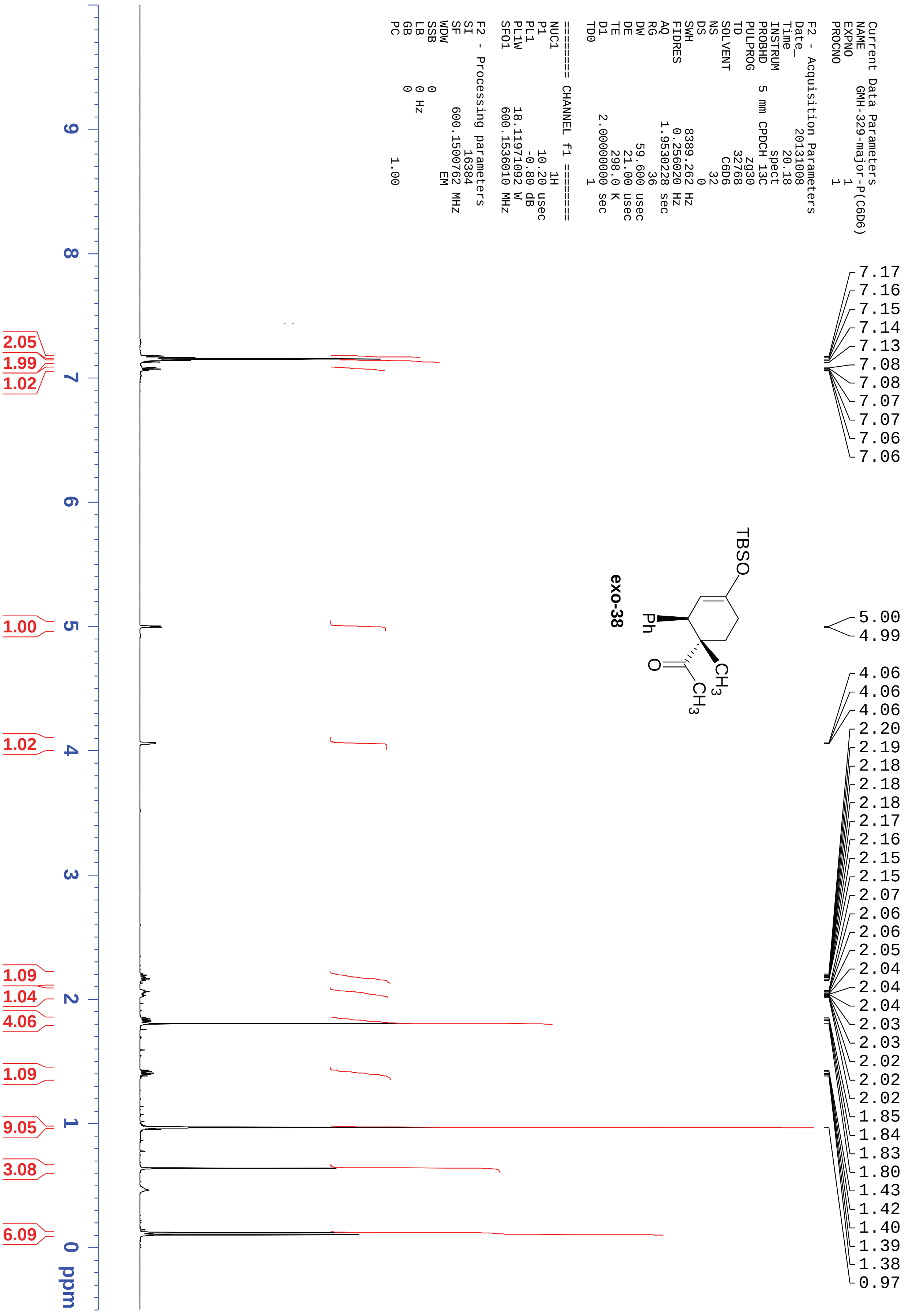

Supplementary Figure 128. **^{1}H NMR spectrum of compound exo-38 in C_6D_6.**

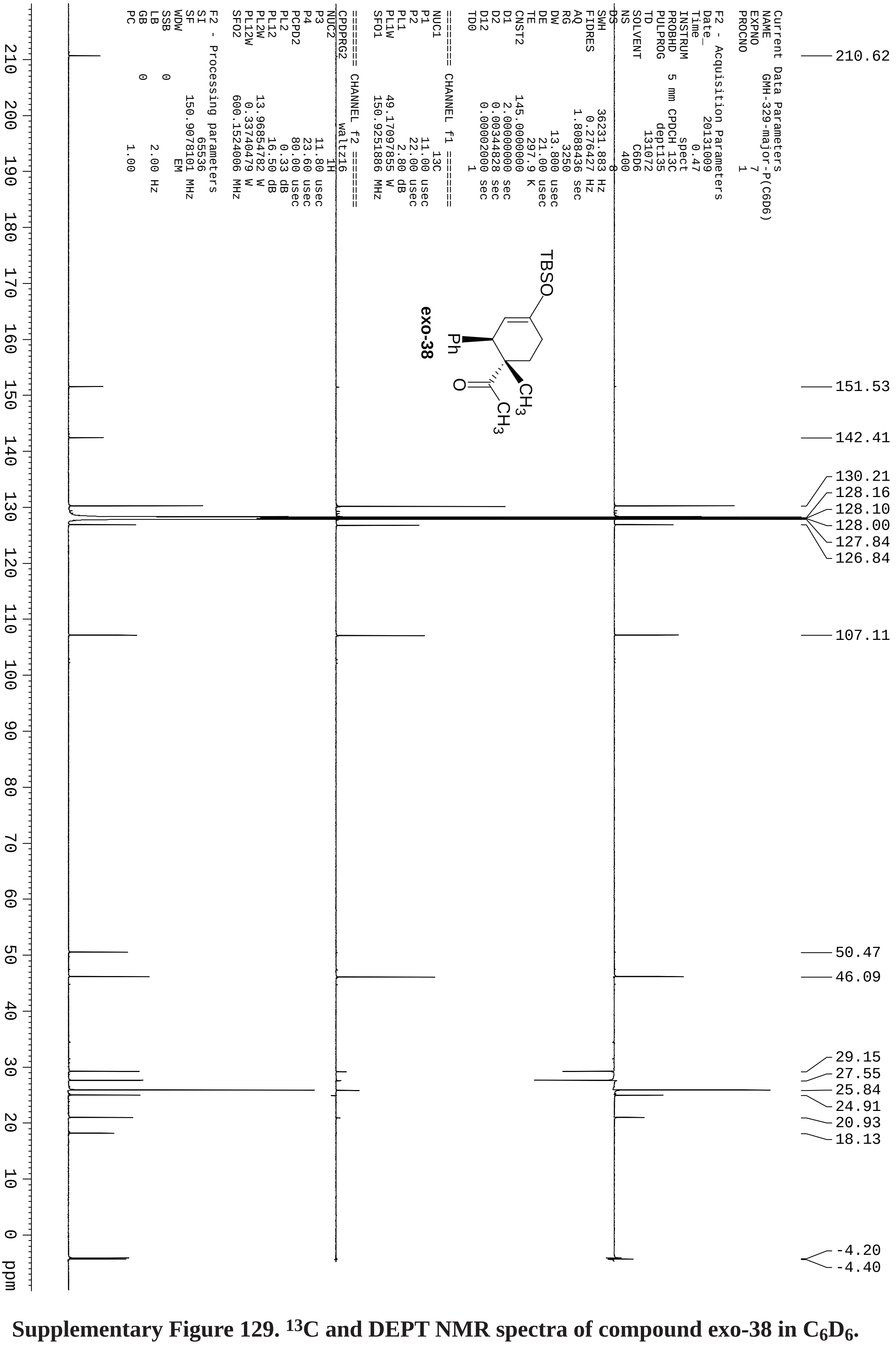

Supplementary Figure 129. ^{13}C and DEPT NMR spectra of compound exo-38 in C_6D_6.

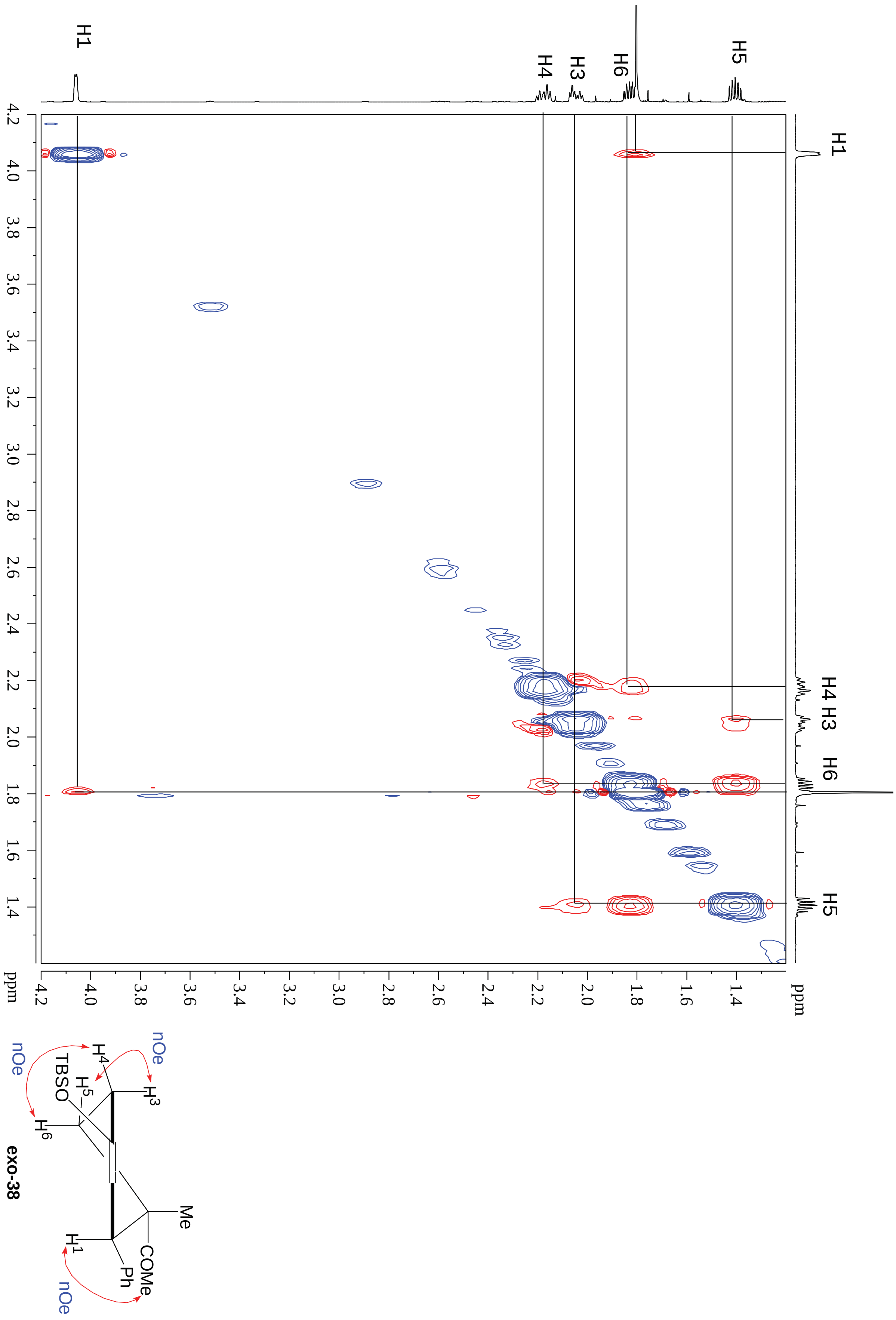

Supplementary Figure 130. NOESY NMR spectrum of compound exo-38 in C_6D_6.

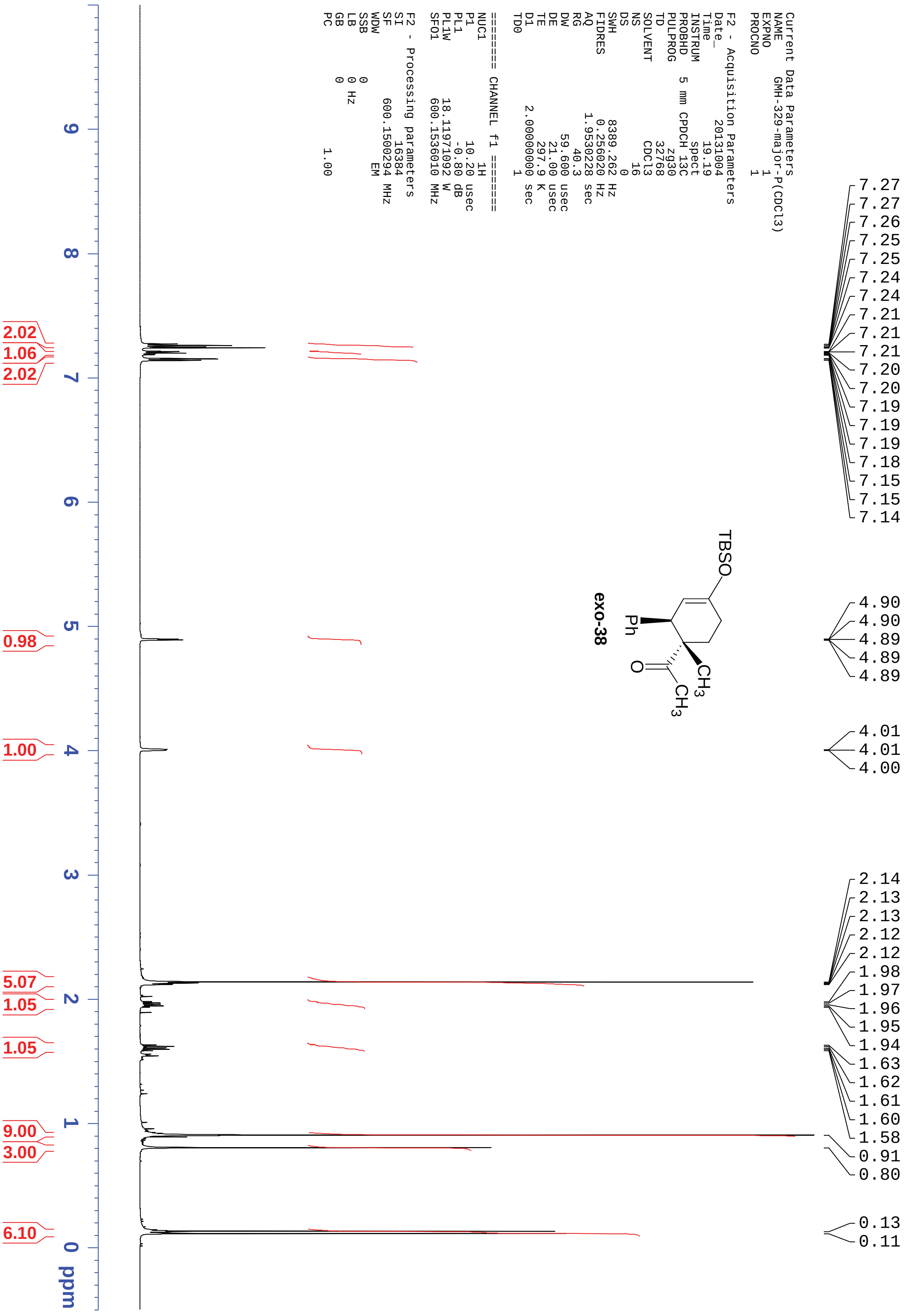

Supplementary Figure 131. ^{1}H NMR spectrum of compound exo-38 in $CDCl_3$.

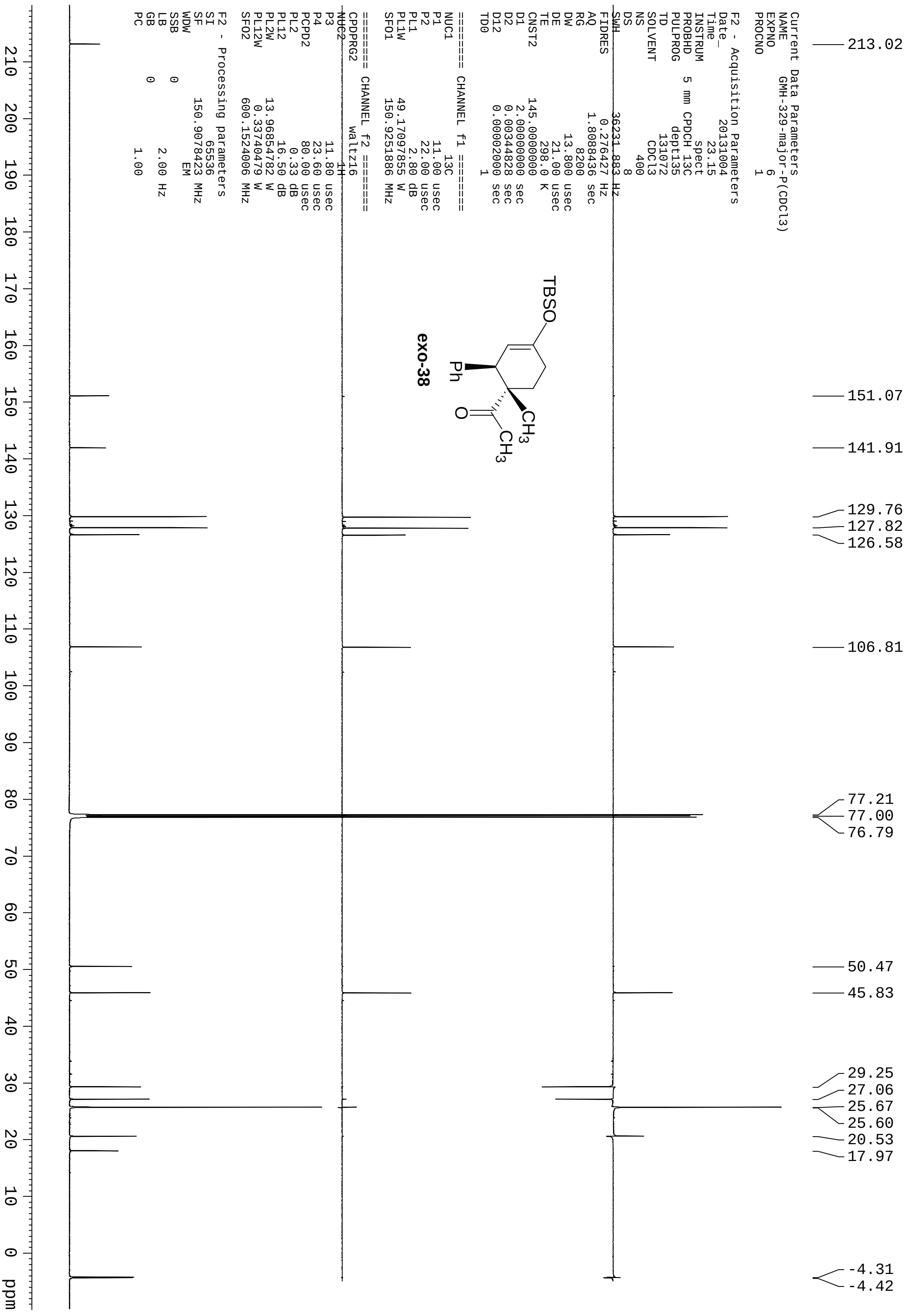

Supplementary Figure 132. ^{13}C and DEPT NMR spectra of compound exo-38 in $CDCl_3$.

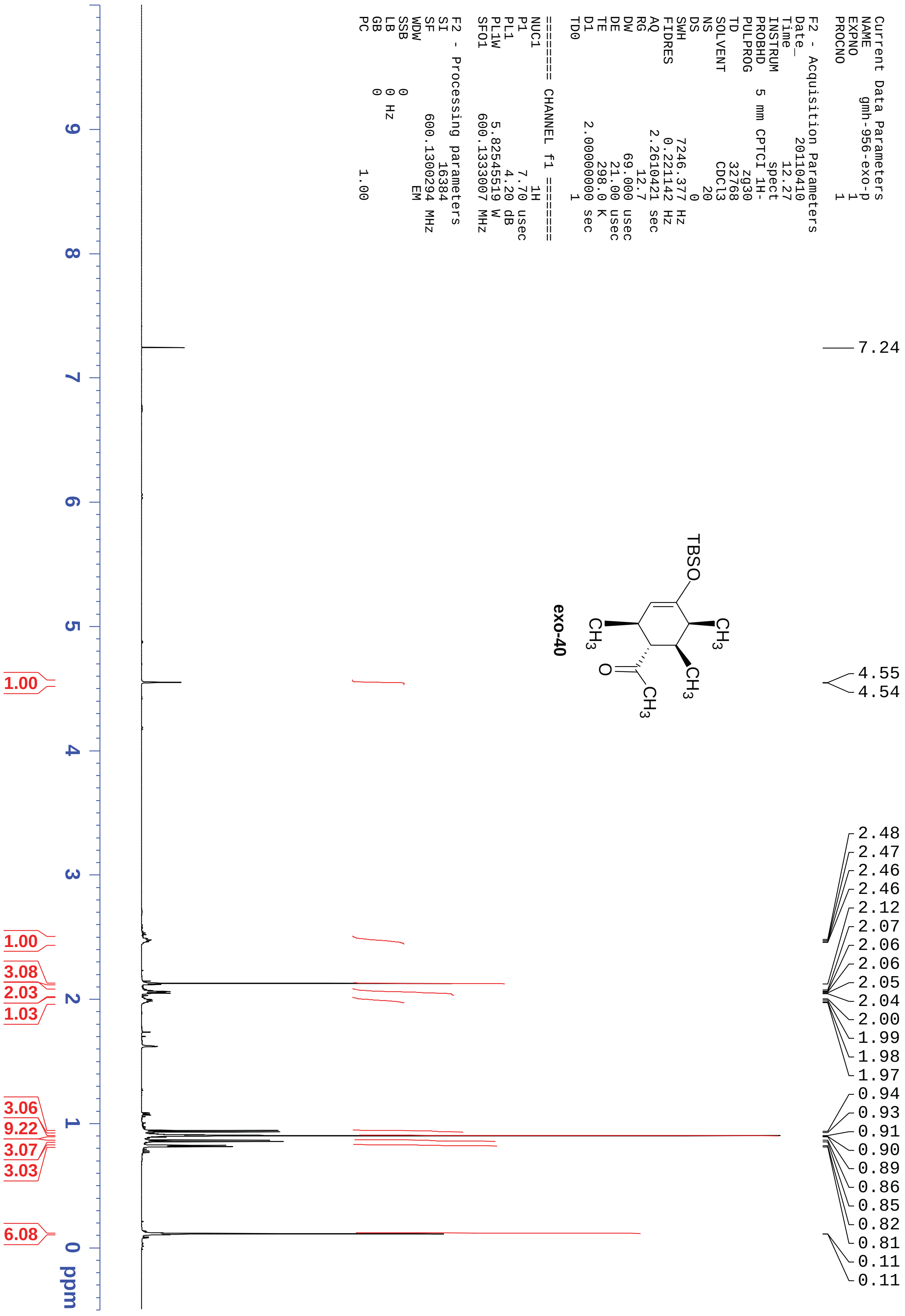

Supplementary Figure 133. ^{1}H NMR spectrum of compound exo-40.

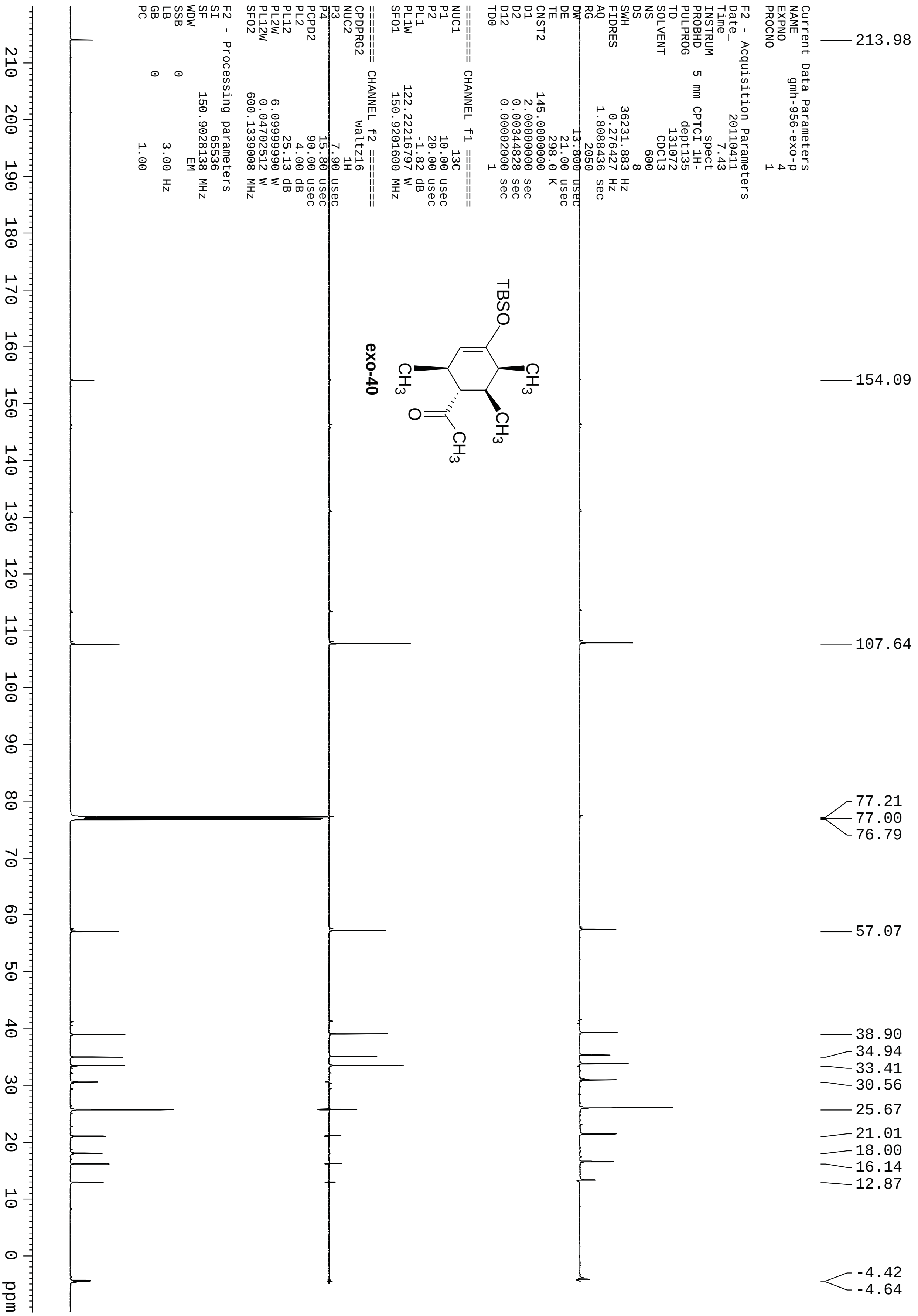

Supplementary Figure 134. ^{13}C and DEPT NMR spectra of compound exo-40.

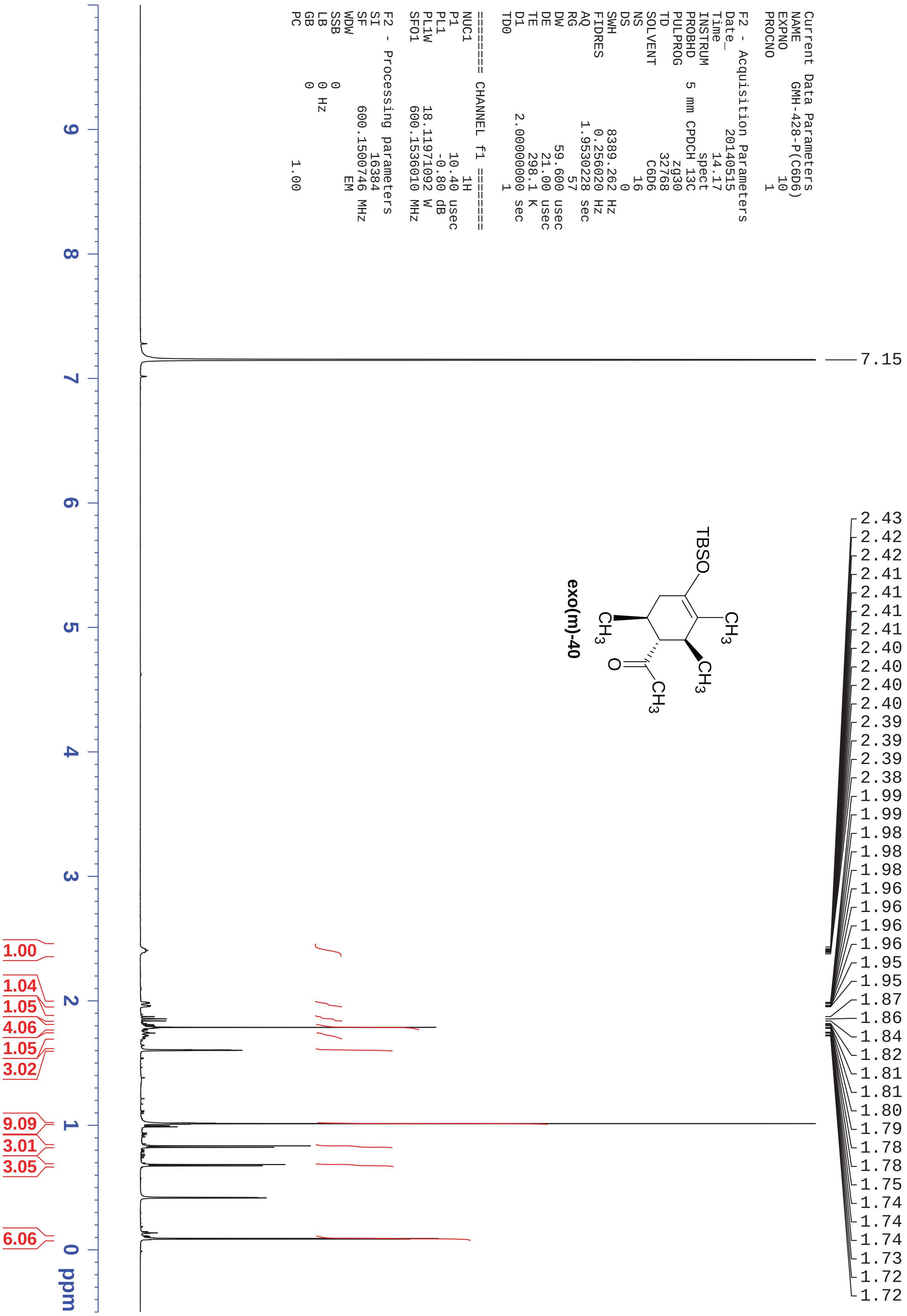

Supplementary Figure 135. ^{1}**H NMR spectrum of compound exo(m)-40.**

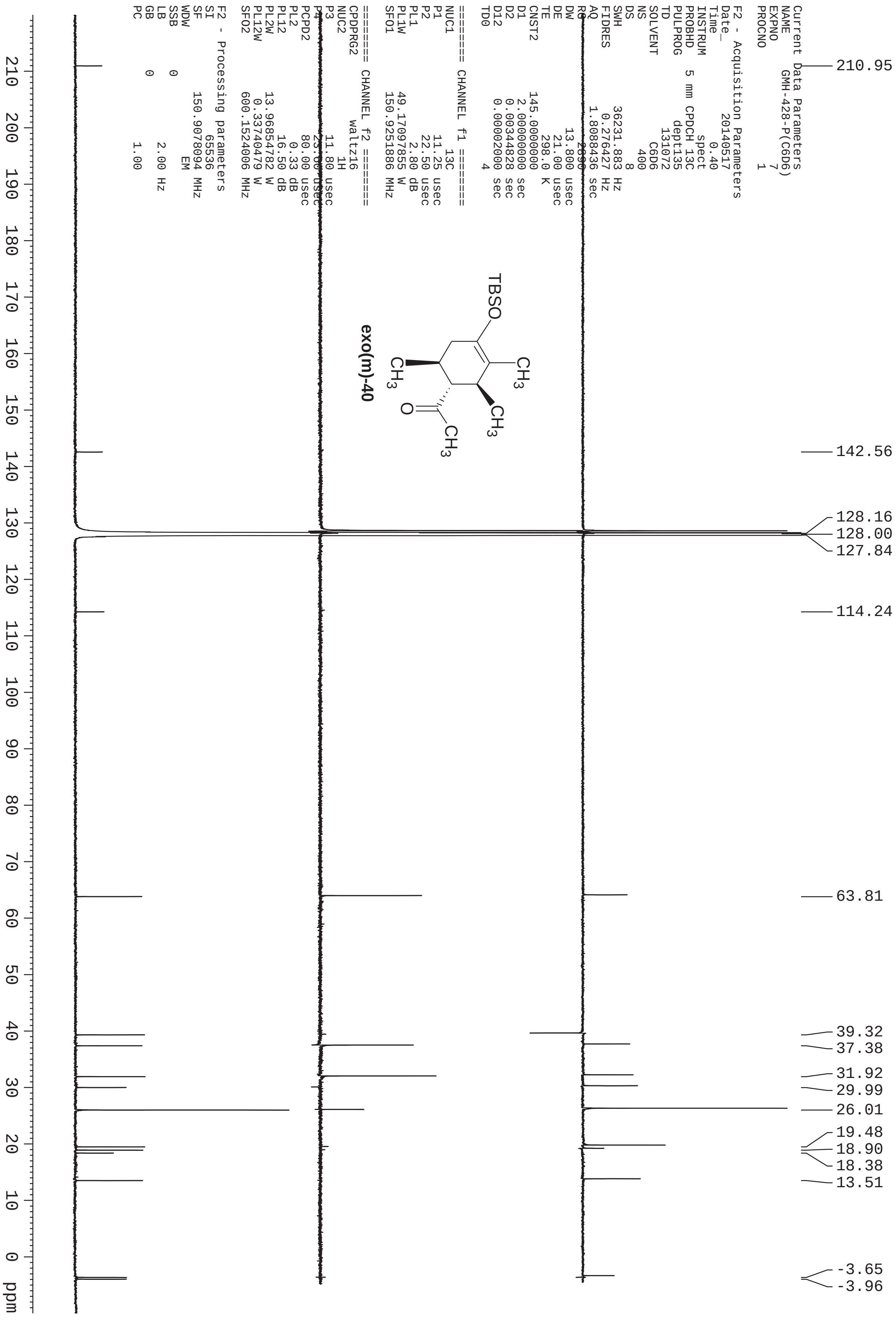

Supplementary Figure 136. ^{13}C and DEPT NMR spectra of compound exo(m)-40.

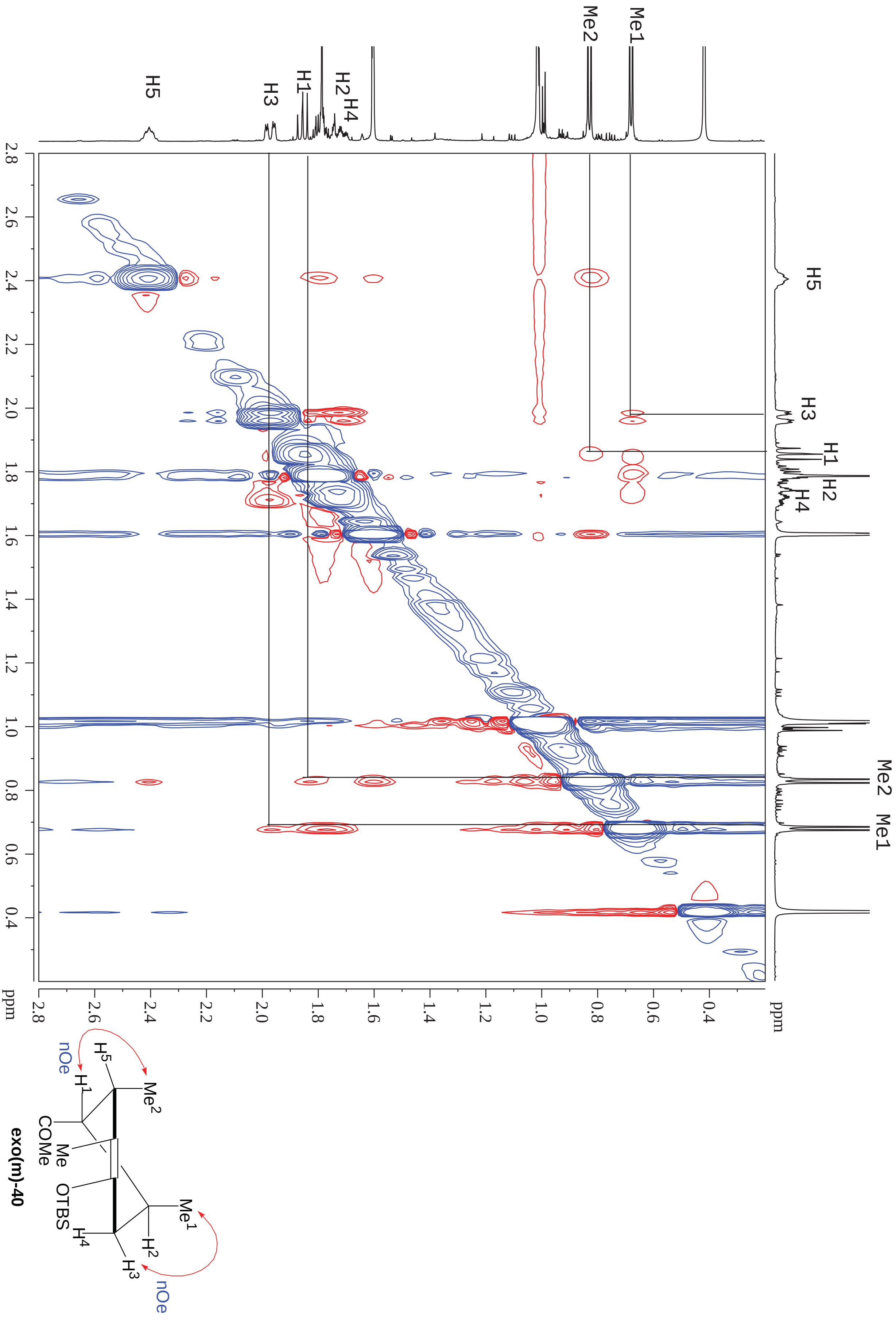

Supplementary Figure 137. NOESY NMR spectrum of compound exo(m)-40.

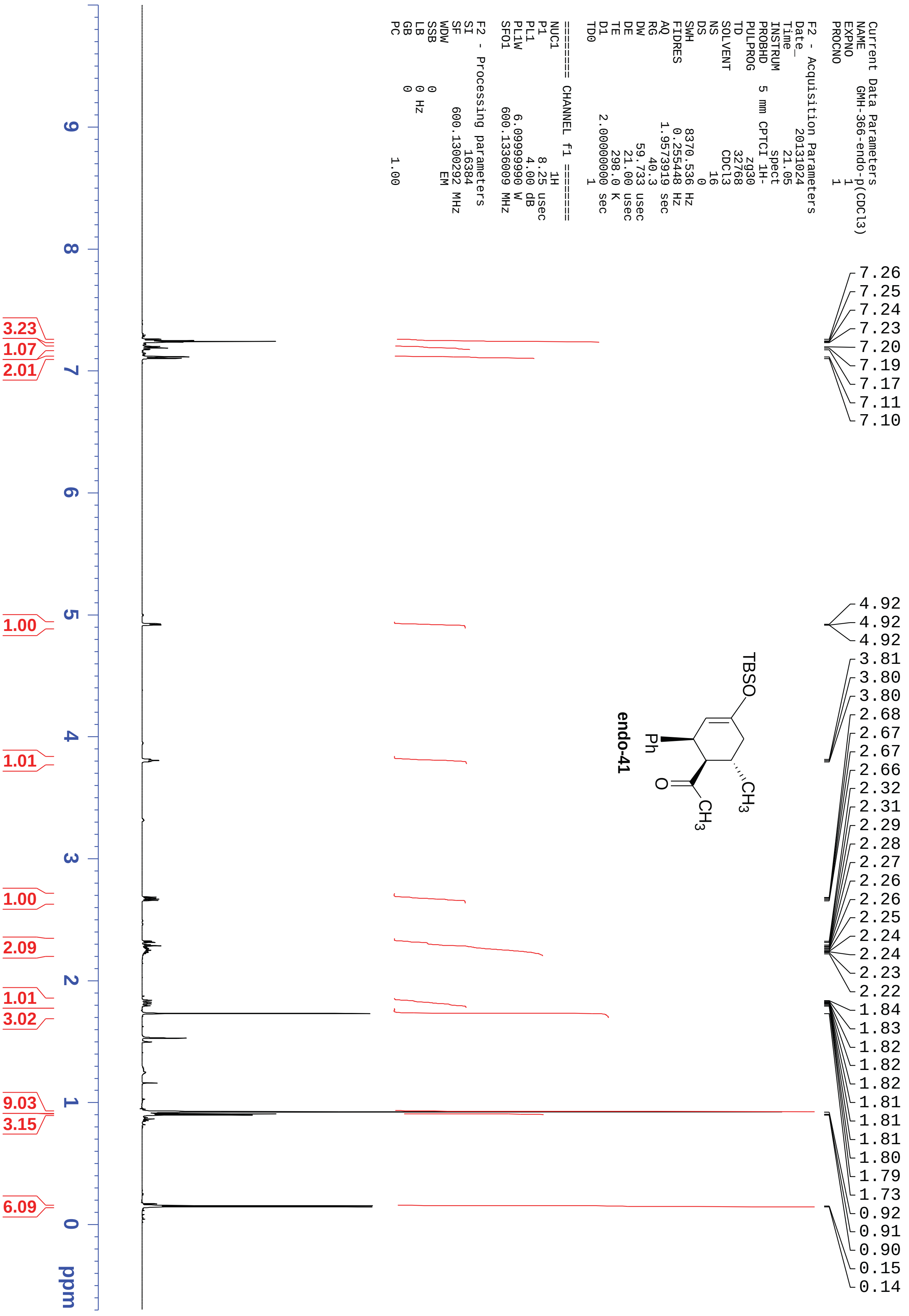

Supplementary Figure 138. ^{1}H NMR spectrum of compound endo-41.

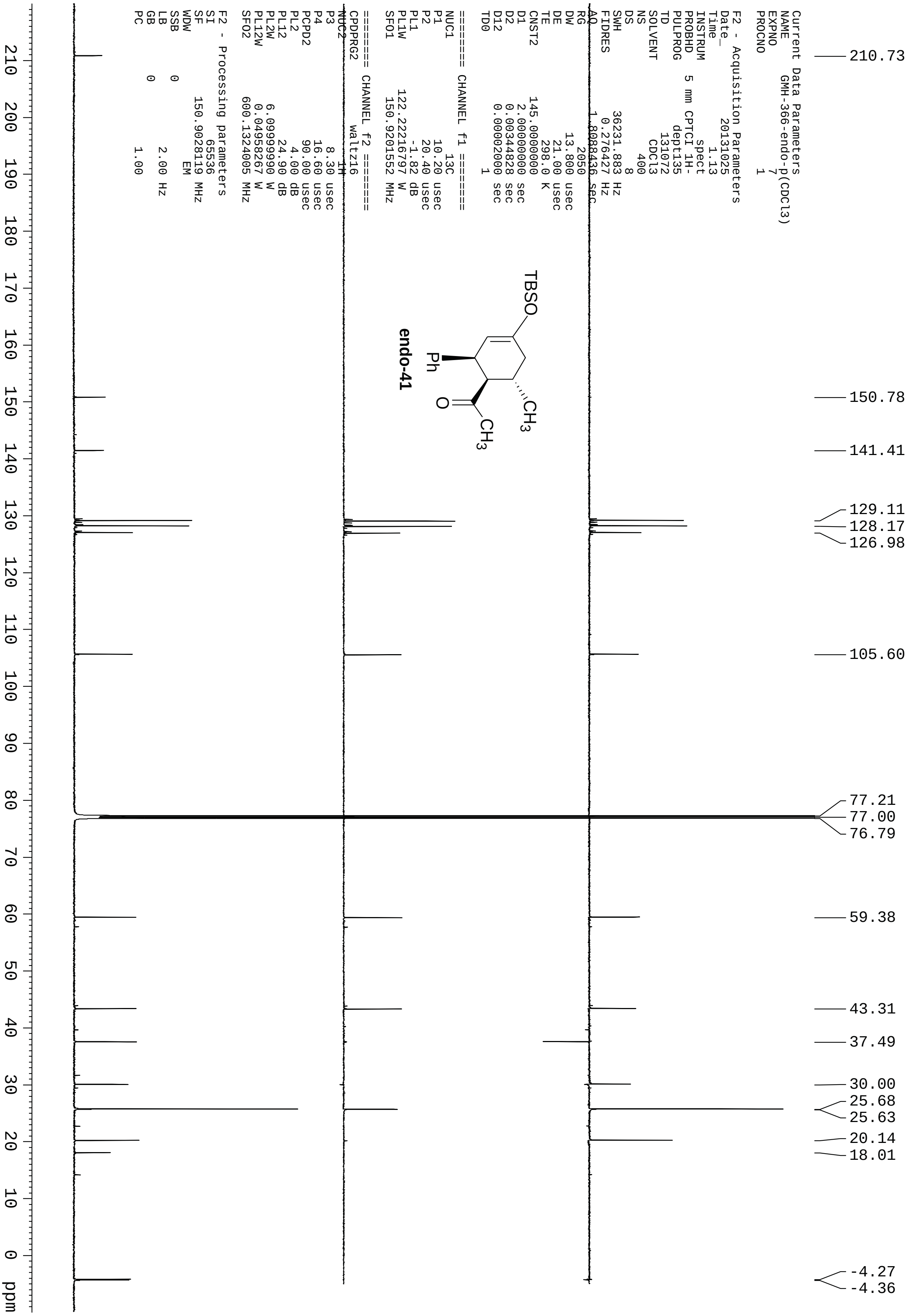

Supplementary Figure 139. ^{13}**C and DEPT NMR spectra of compound endo-41.**

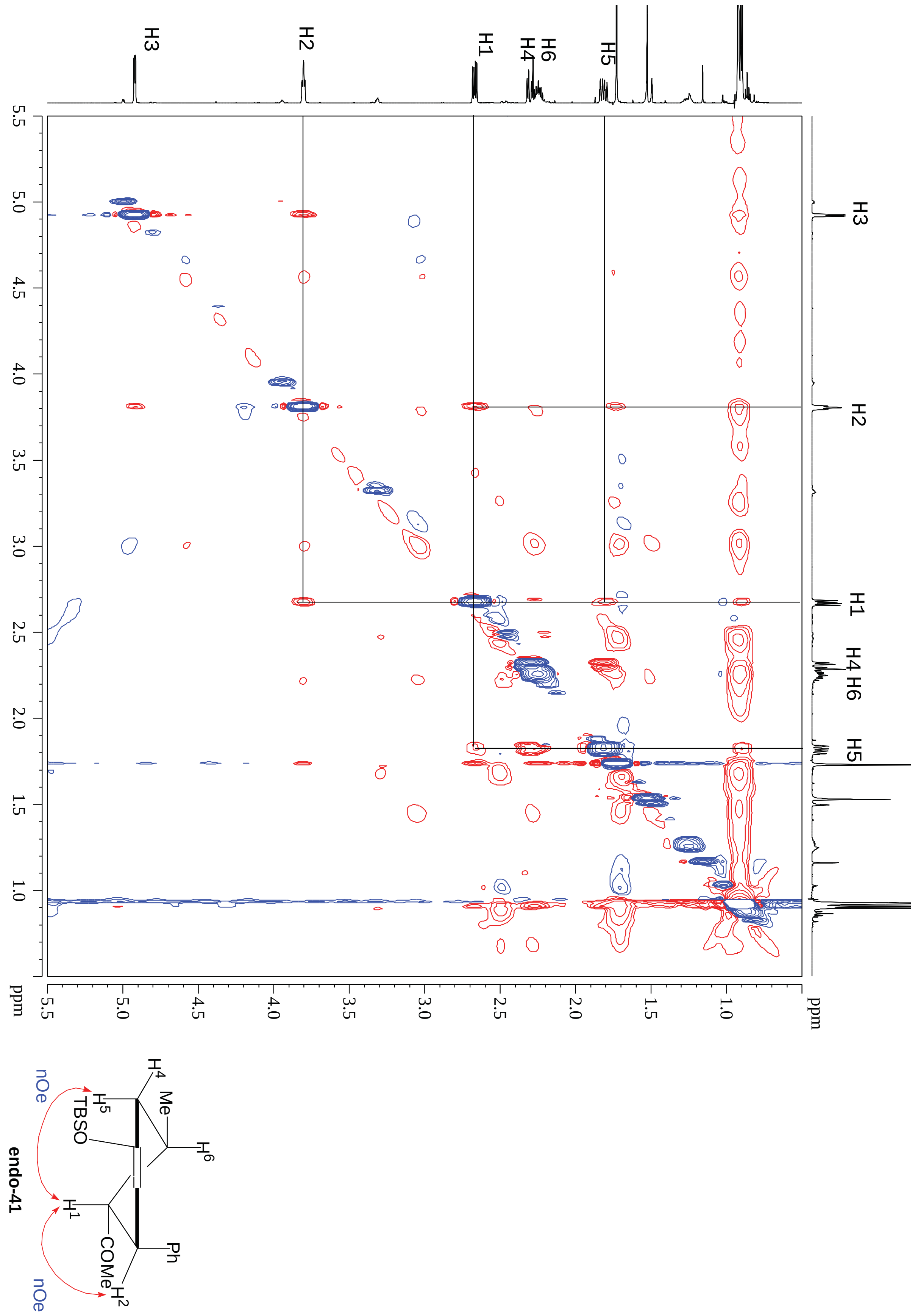

Supplementary Figure 140. NOESY NMR spectrum of compound endo-41.

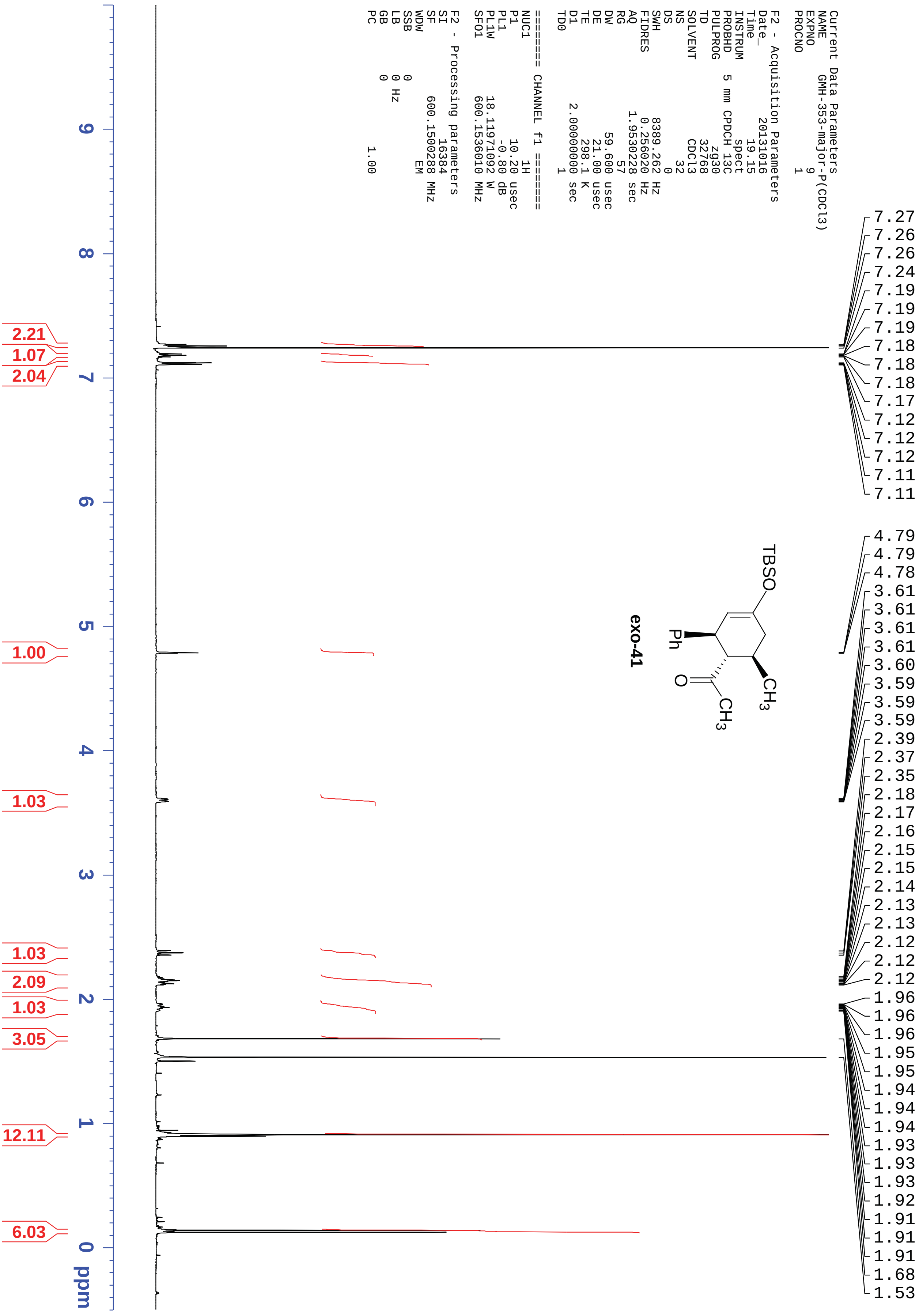

Supplementary Figure 141. ^{1}**H NMR spectrum of compound exo-41 in** $CDCl_3$**.**

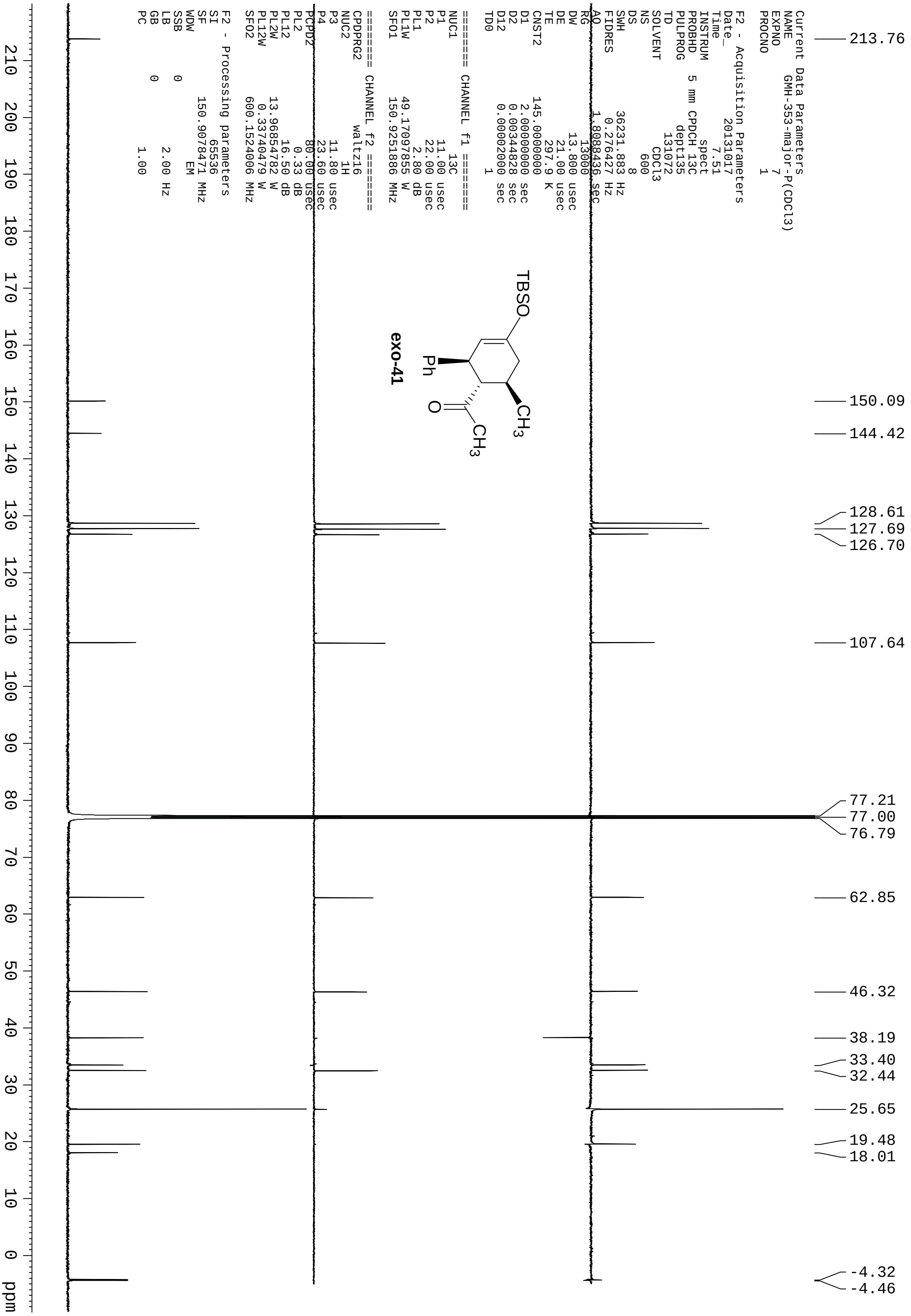

Supplementary Figure 142. ^{13}C and DEPT NMR spectra of compound exo-41 in $CDCl_3$.

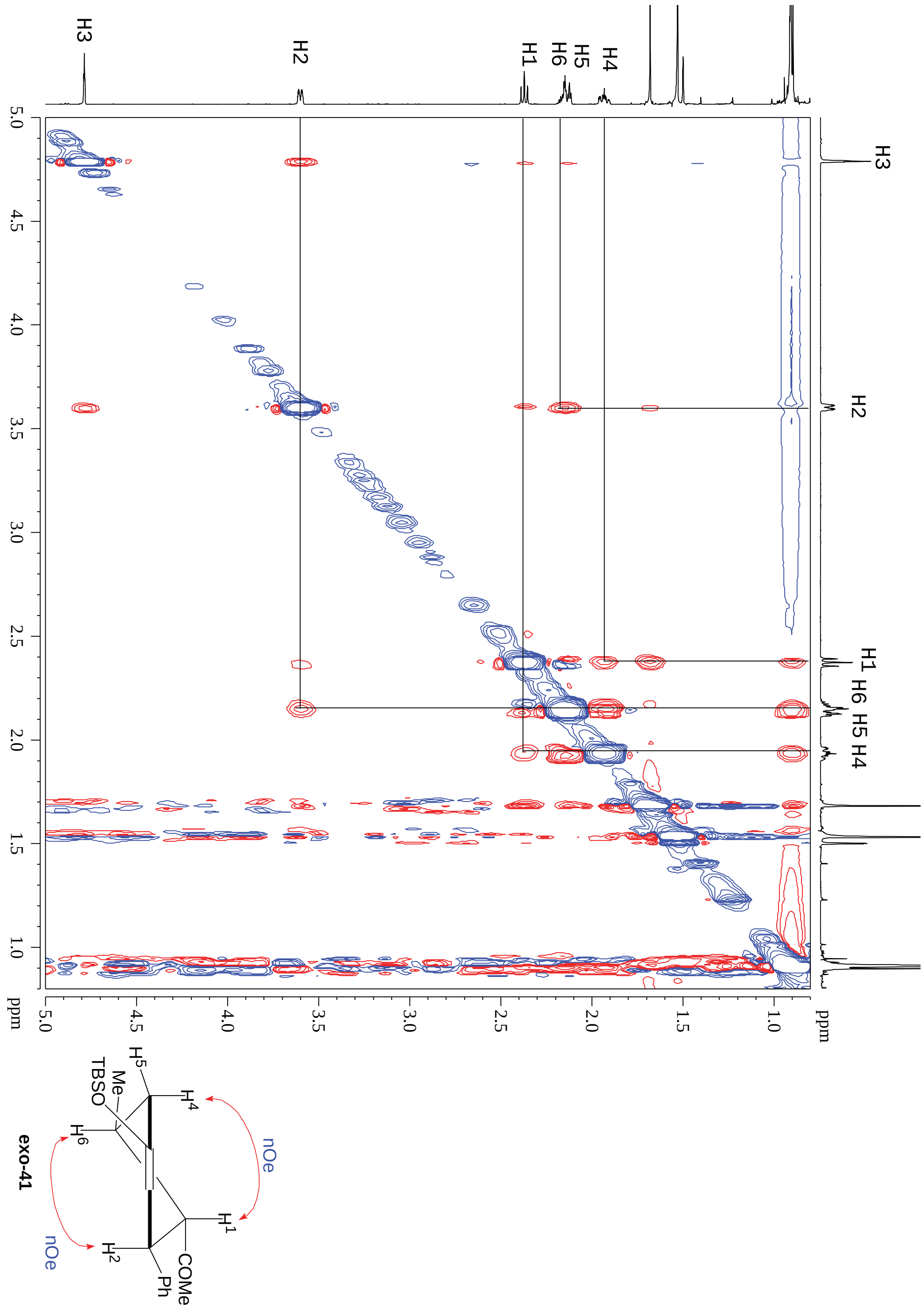

Supplementary Figure 143. NOESY NMR spectrum of compound exo-41 in $CDCl_3$.

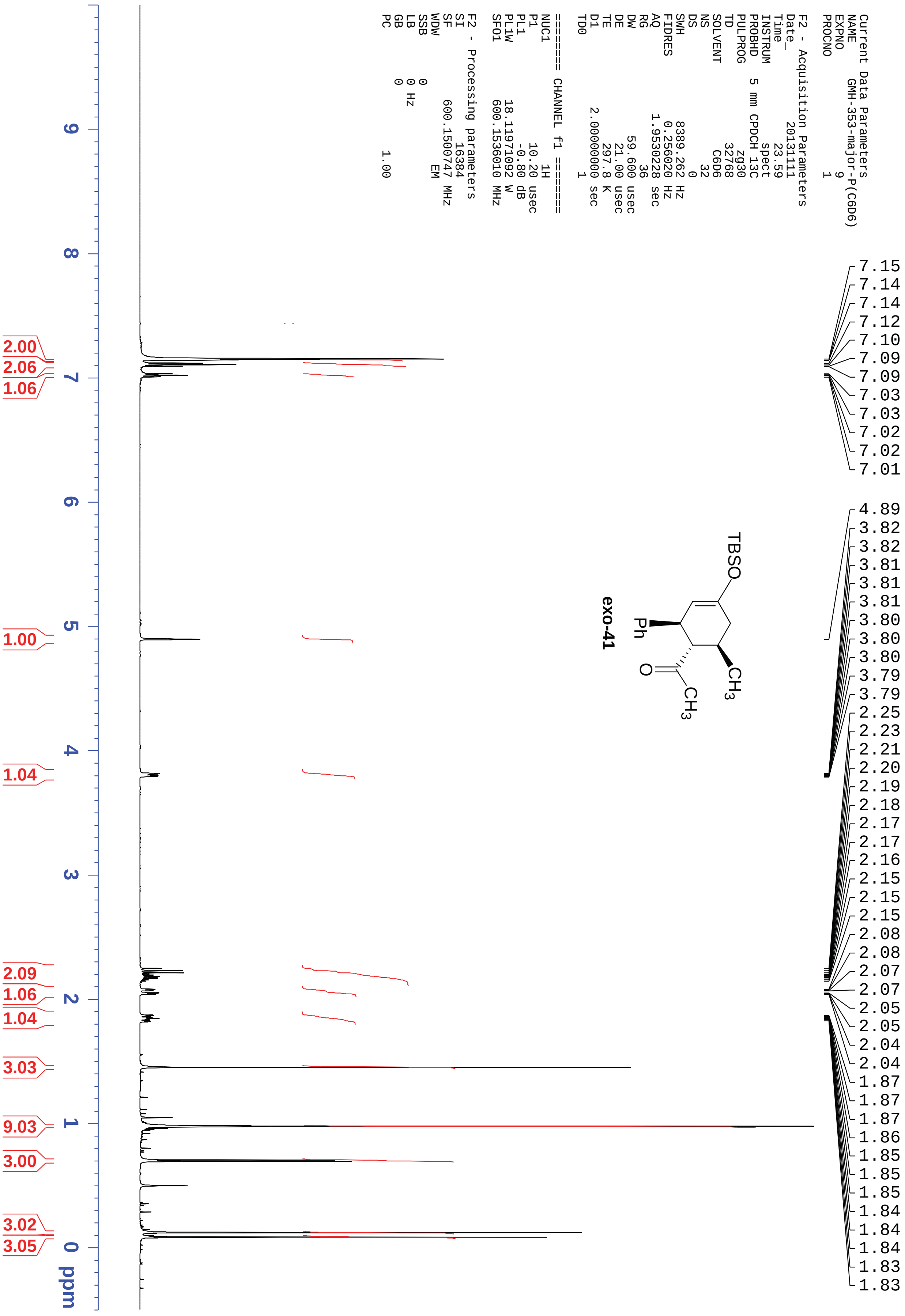

Supplementary Figure 144. ^{1}H **NMR spectrum of compound exo-41 in** C_6D_6**.**

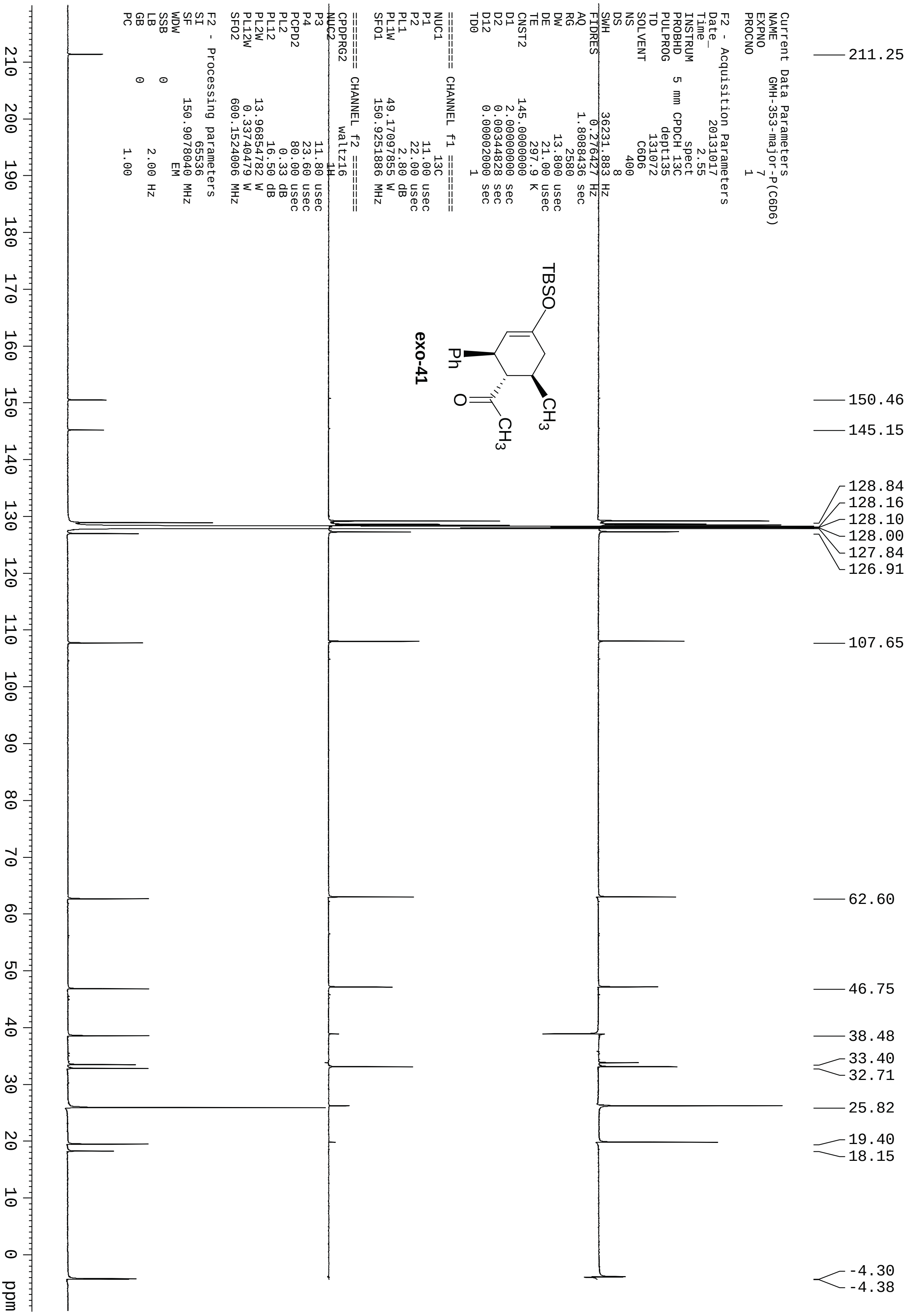

Supplementary Figure 145. ^{13}C and DEPT NMR spectra of compound exo-41 in C_6D_6.

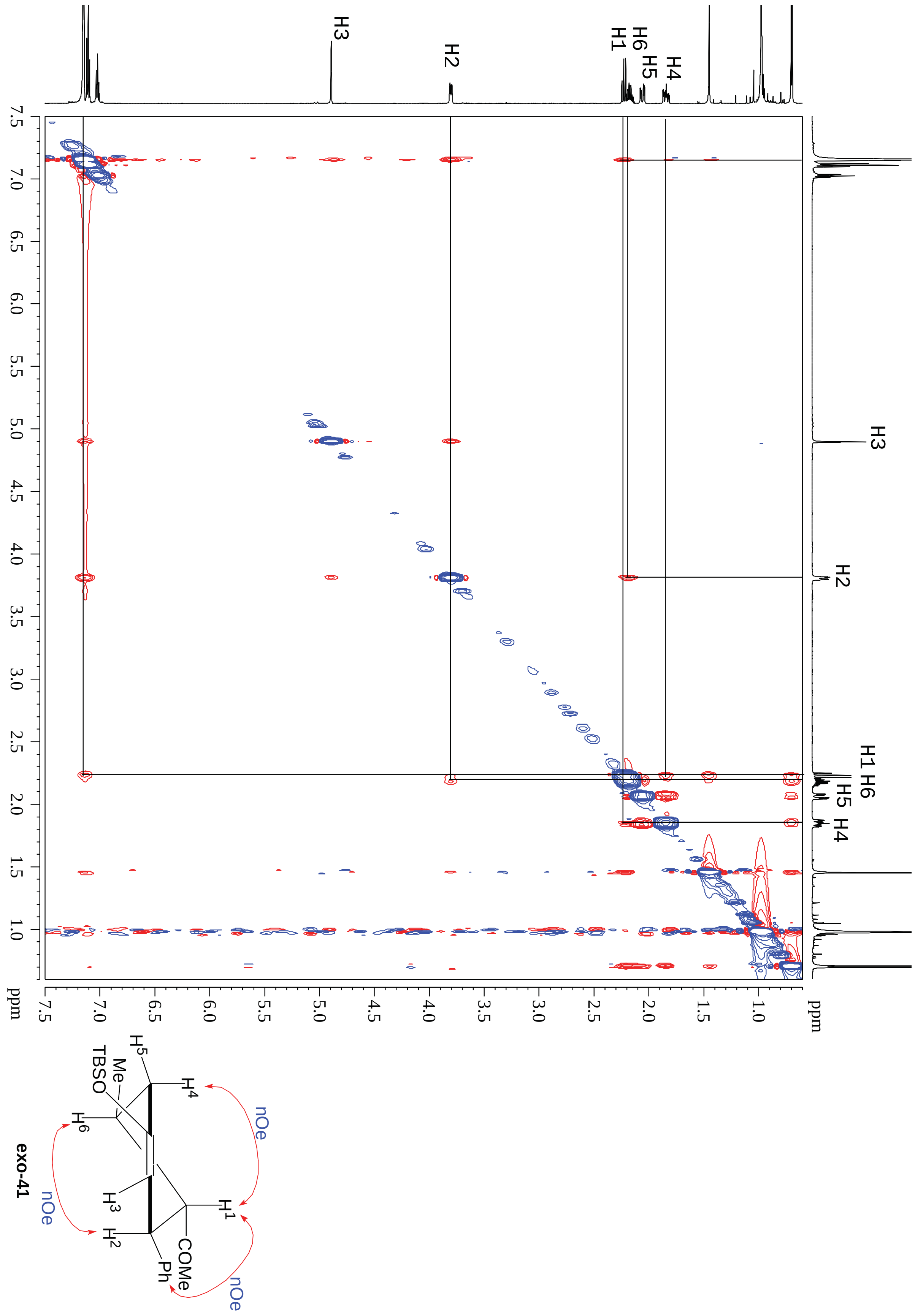

Supplementary Figure 146. NOESY NMR spectrum of compound exo-41 in C_6D_6.

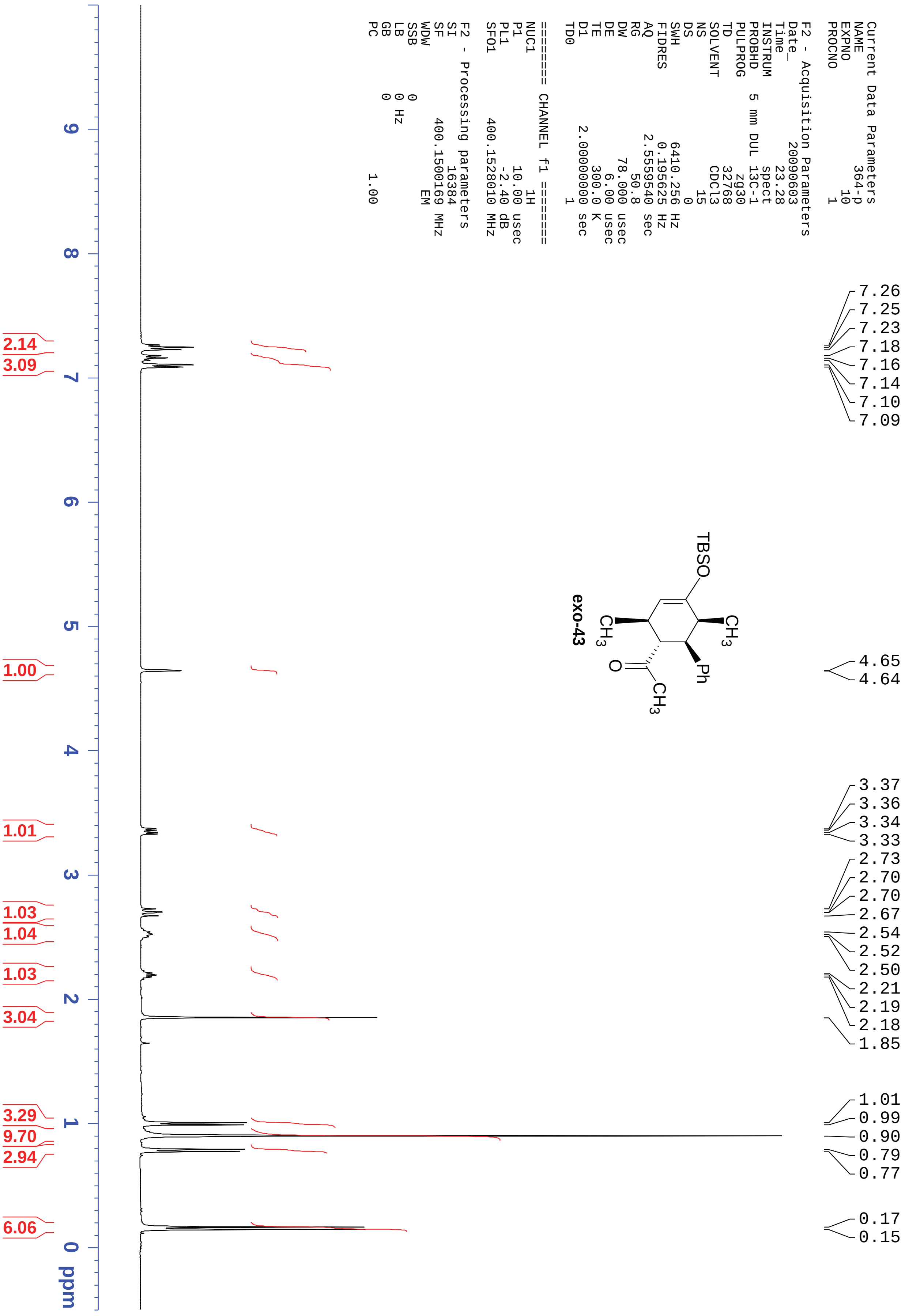

Supplementary Figure 147. ^{1}H NMR spectrum of compound exo-43.

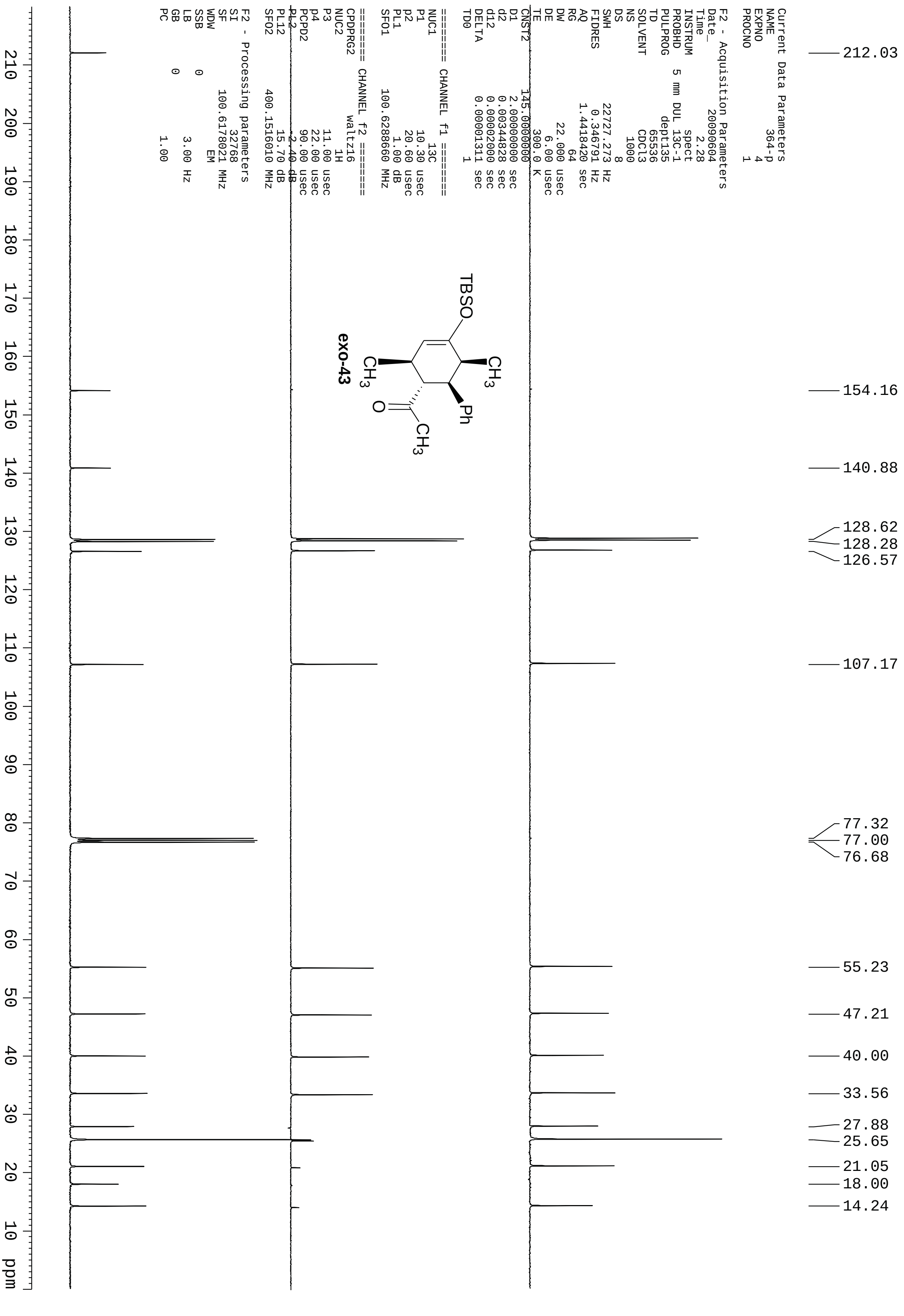

Supplementary Figure 148. ^{13}**C and DEPT NMR spectra of compound exo-43.**

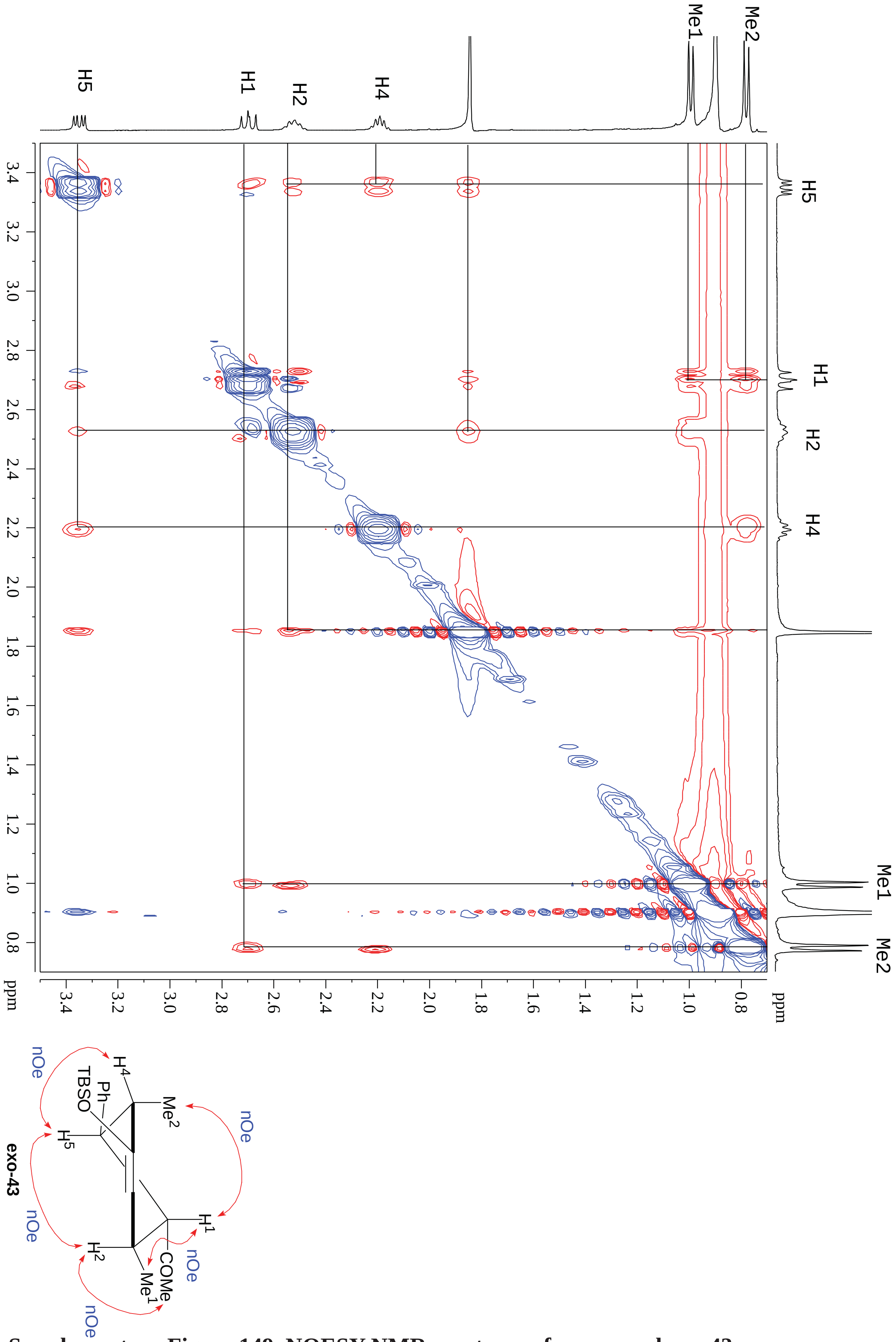

Supplementary Figure 149. NOESY NMR spectrum of compound exo-43.

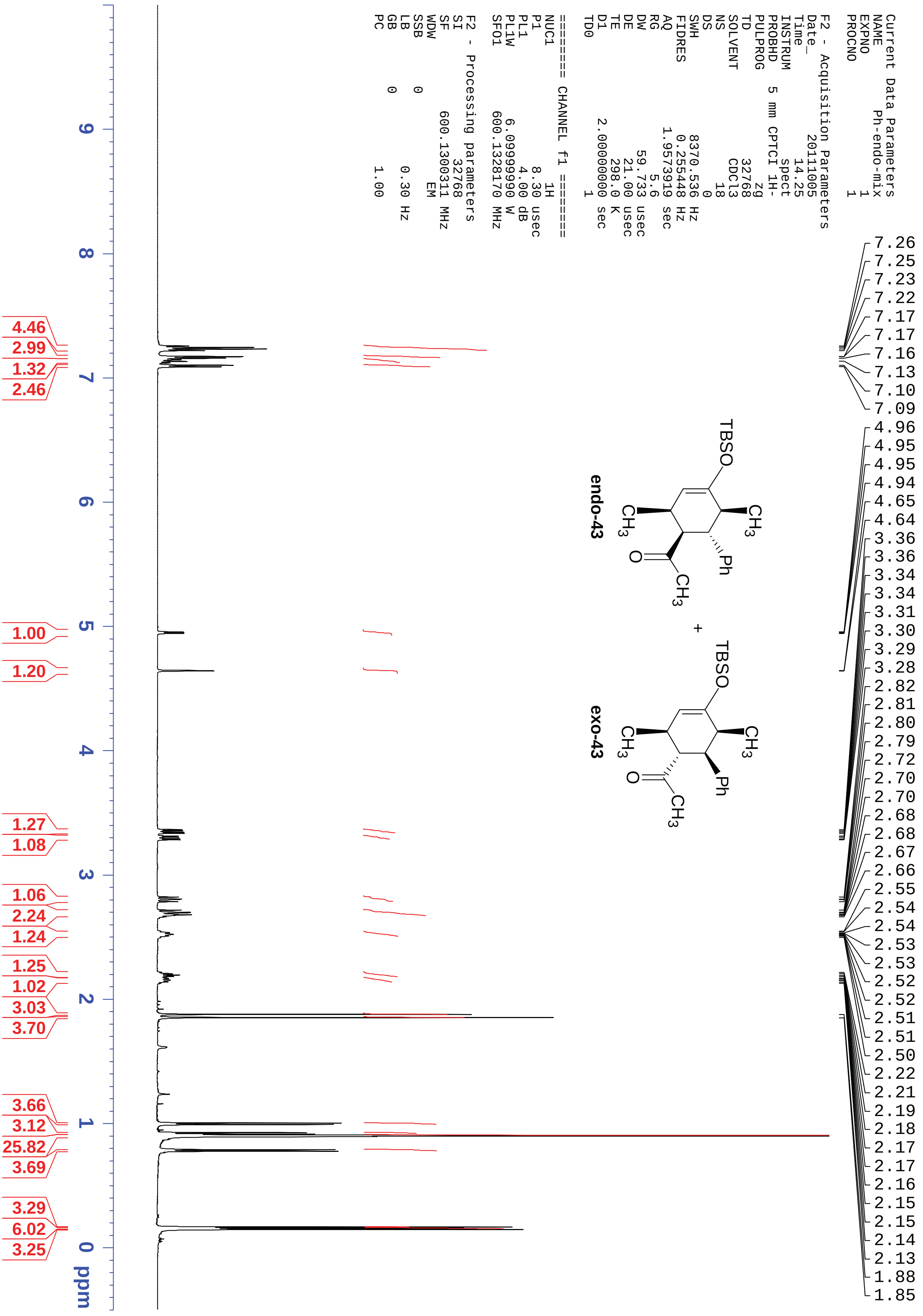

Supplementary Figure 150. ^{1}H **NMR spectrum of mixed compounds endo/exo-43.**

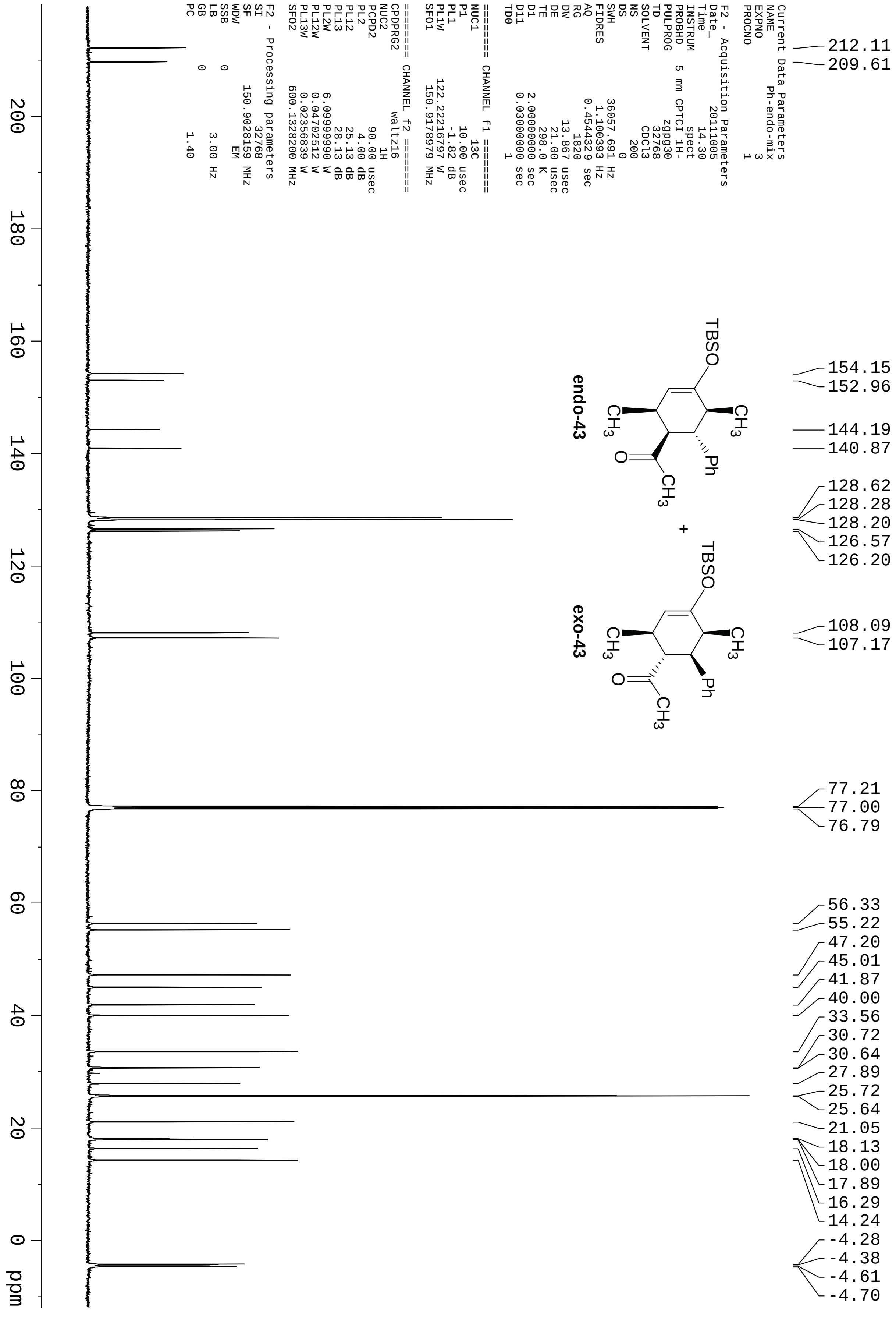

Supplementary Figure 151. ^{13}C NMR spectrum of mixed compounds endo/exo-43.

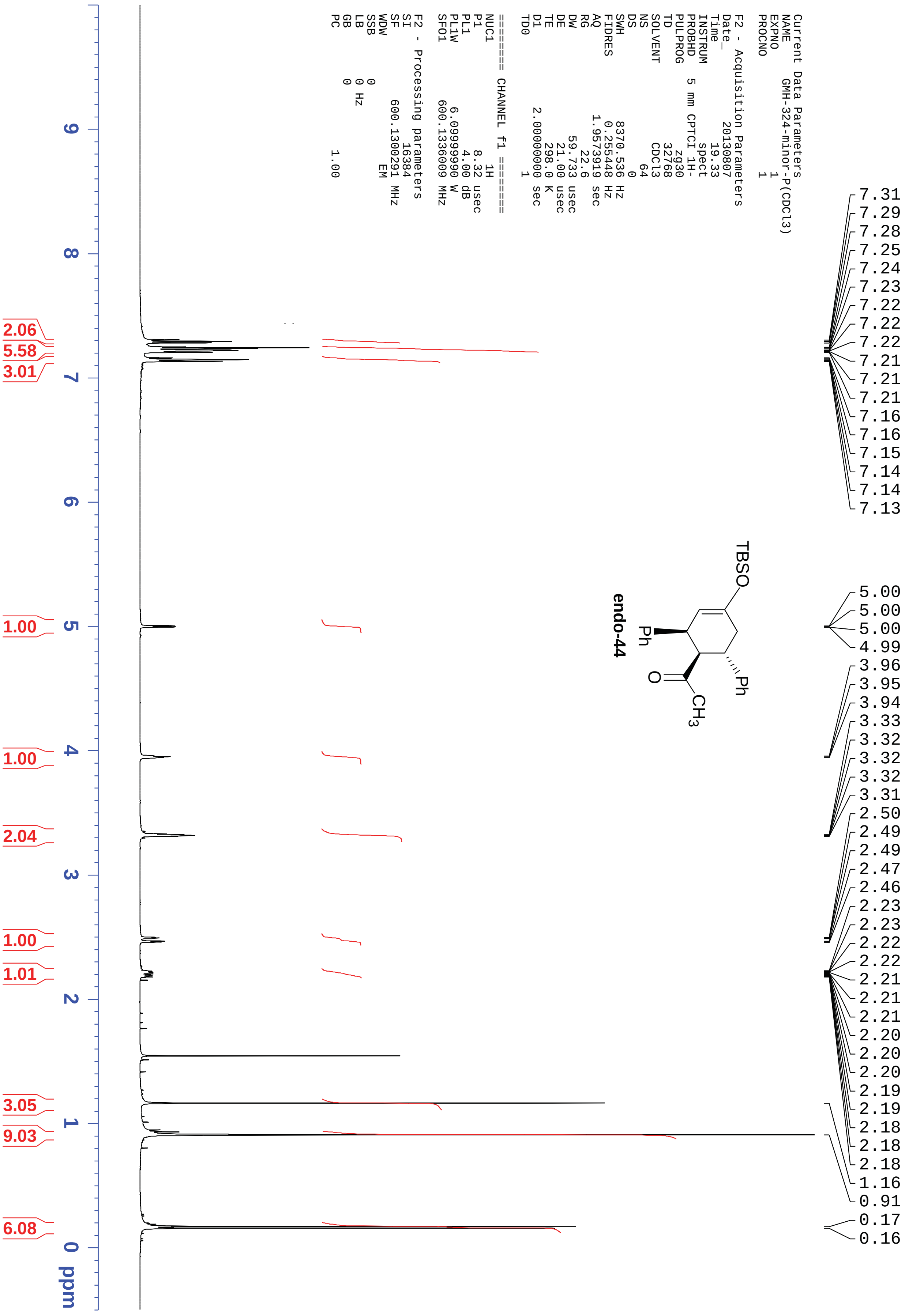

Supplementary Figure 152. ^{1}H **NMR spectrum of compound endo-44 in** $CDCl_3$**.**

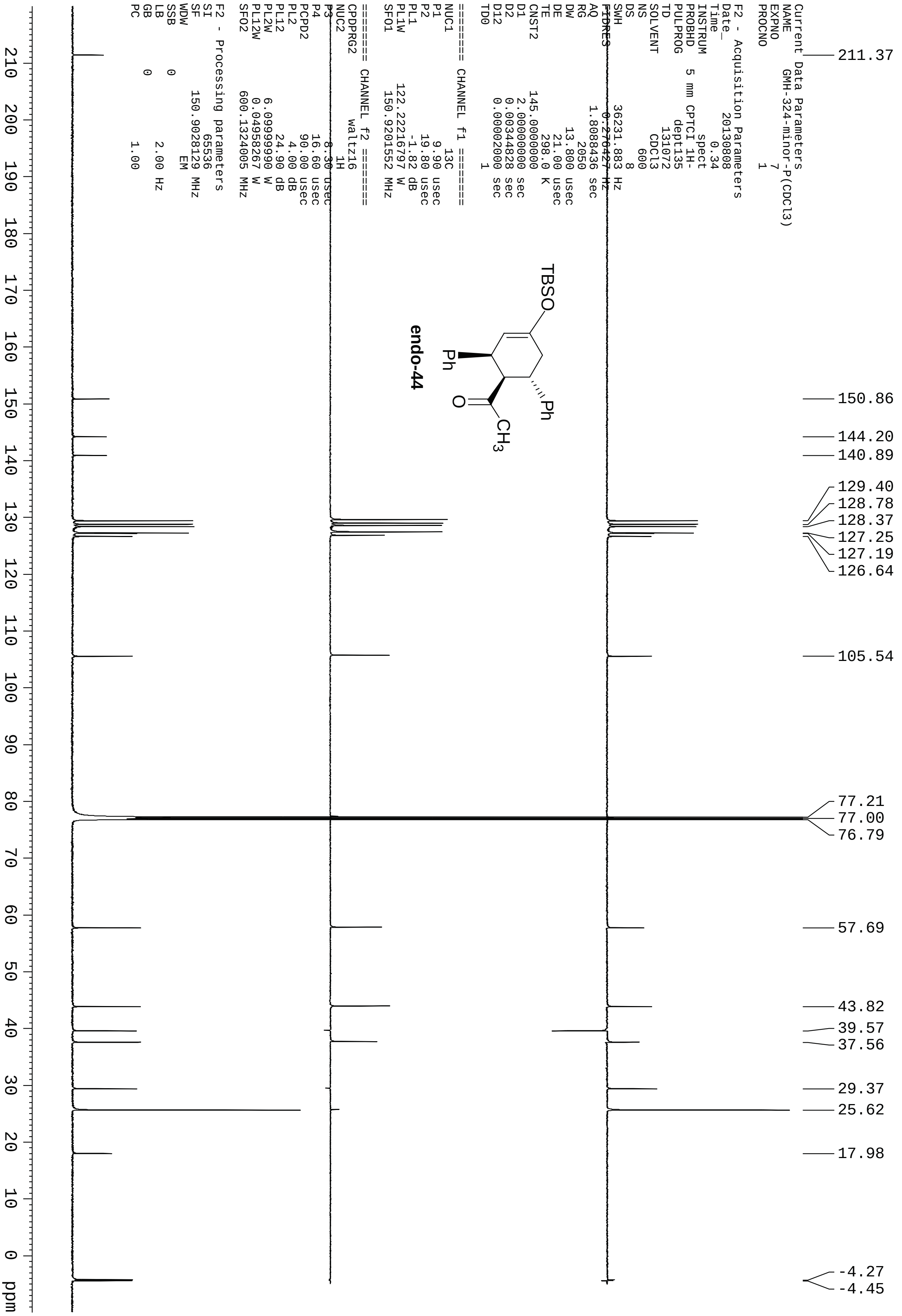

Supplementary Figure 153. ^{13}C and DEPT NMR spectra of compound endo-44 in $CDCl_3$.

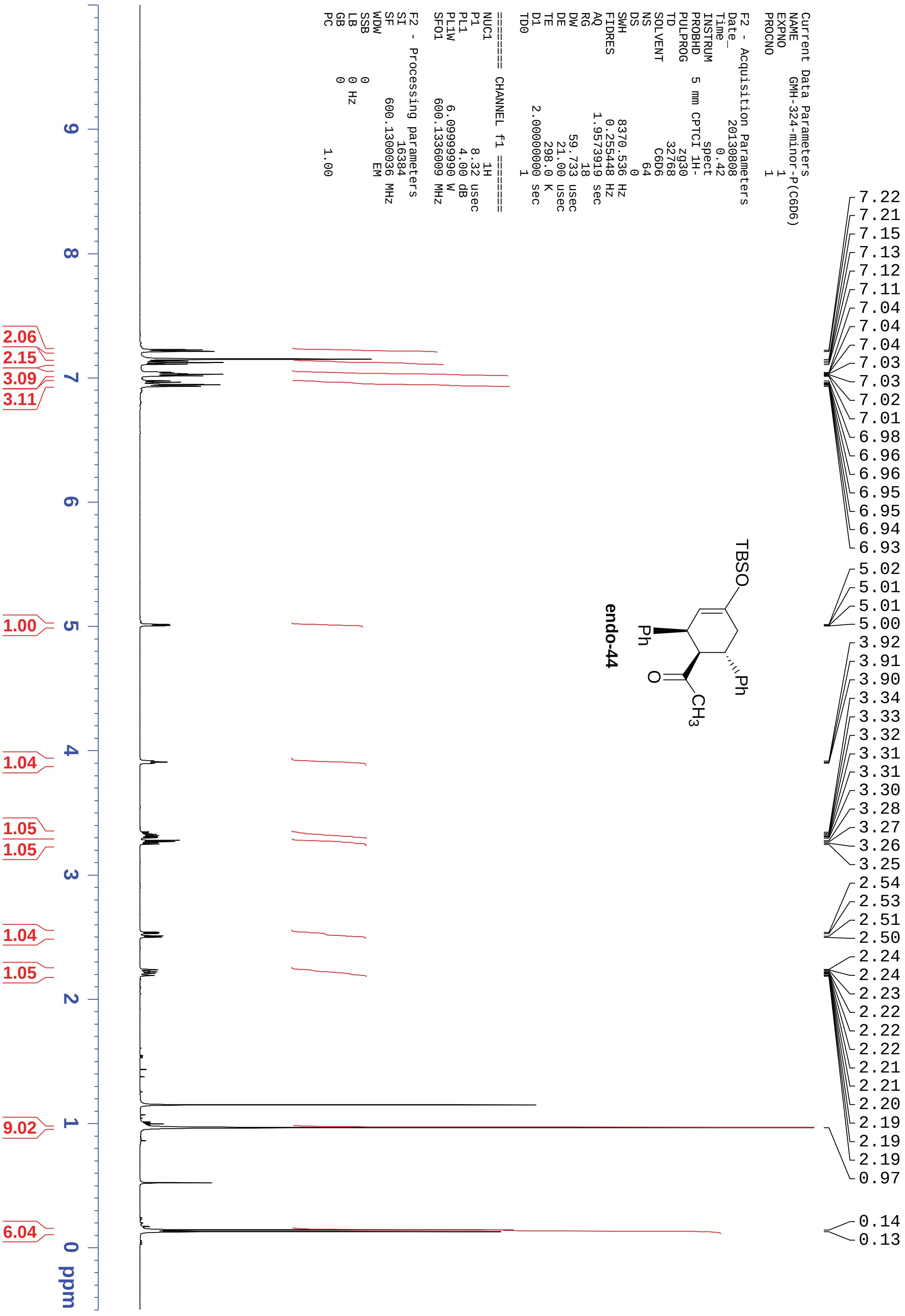

Supplementary Figure 154. **^{1}H NMR spectrum of compound endo-44 in C_6D_6.**

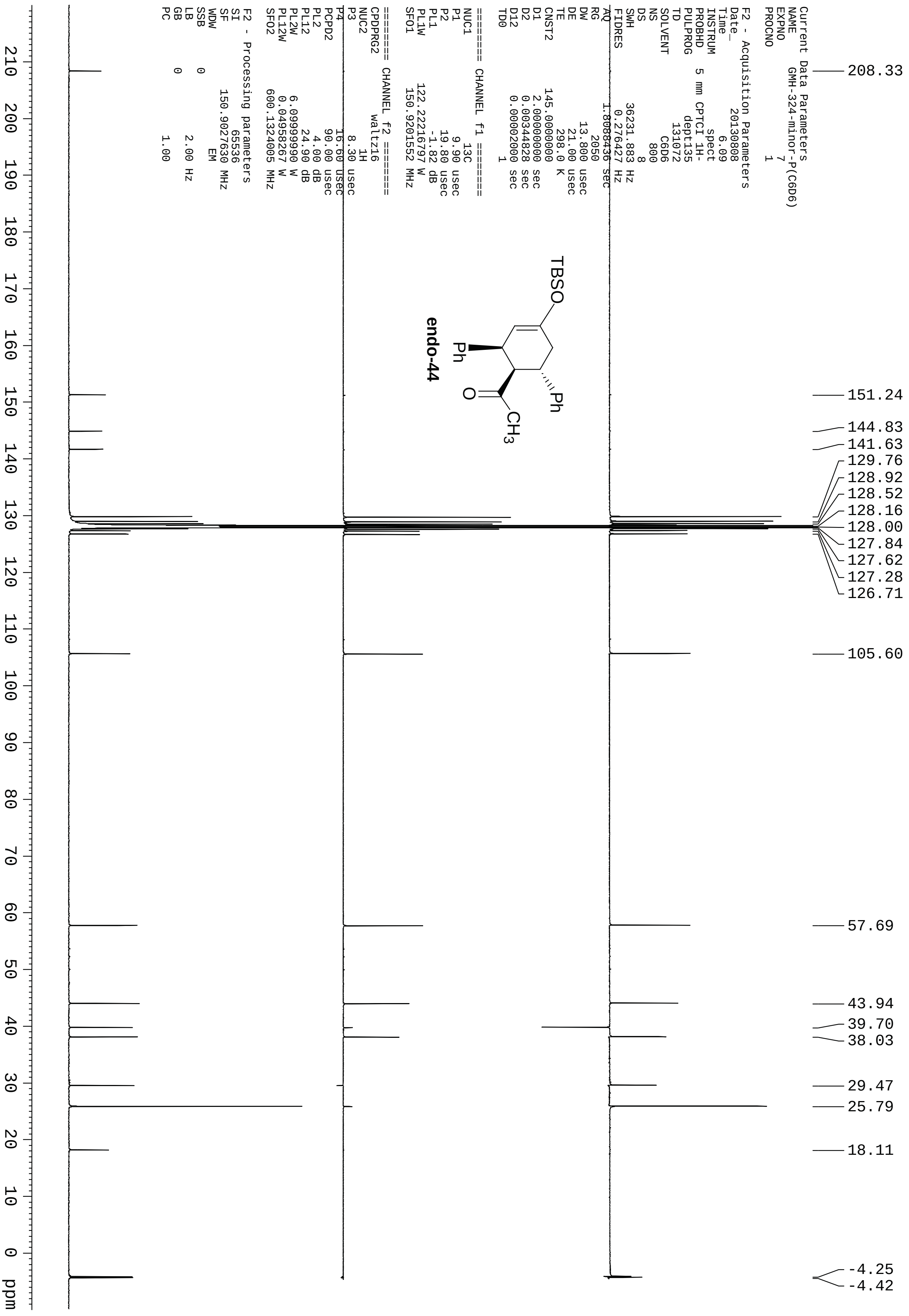

Supplementary Figure 155. ^{13}C and DEPT NMR spectra of compound endo-44 in C_6D_6.

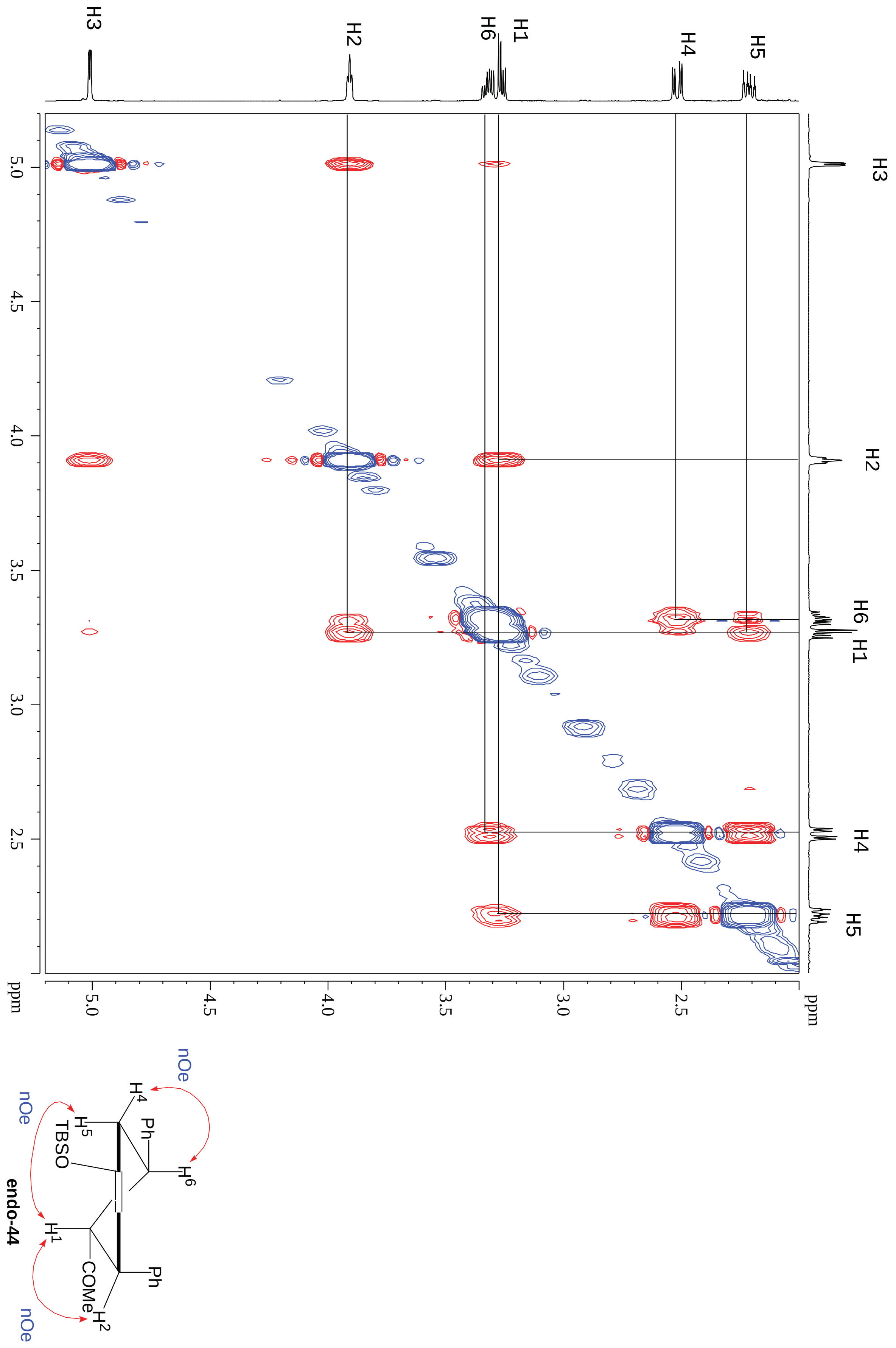

Supplementary Figure 156. NOESY NMR spectrum of compound endo-44 in C_6D_6.

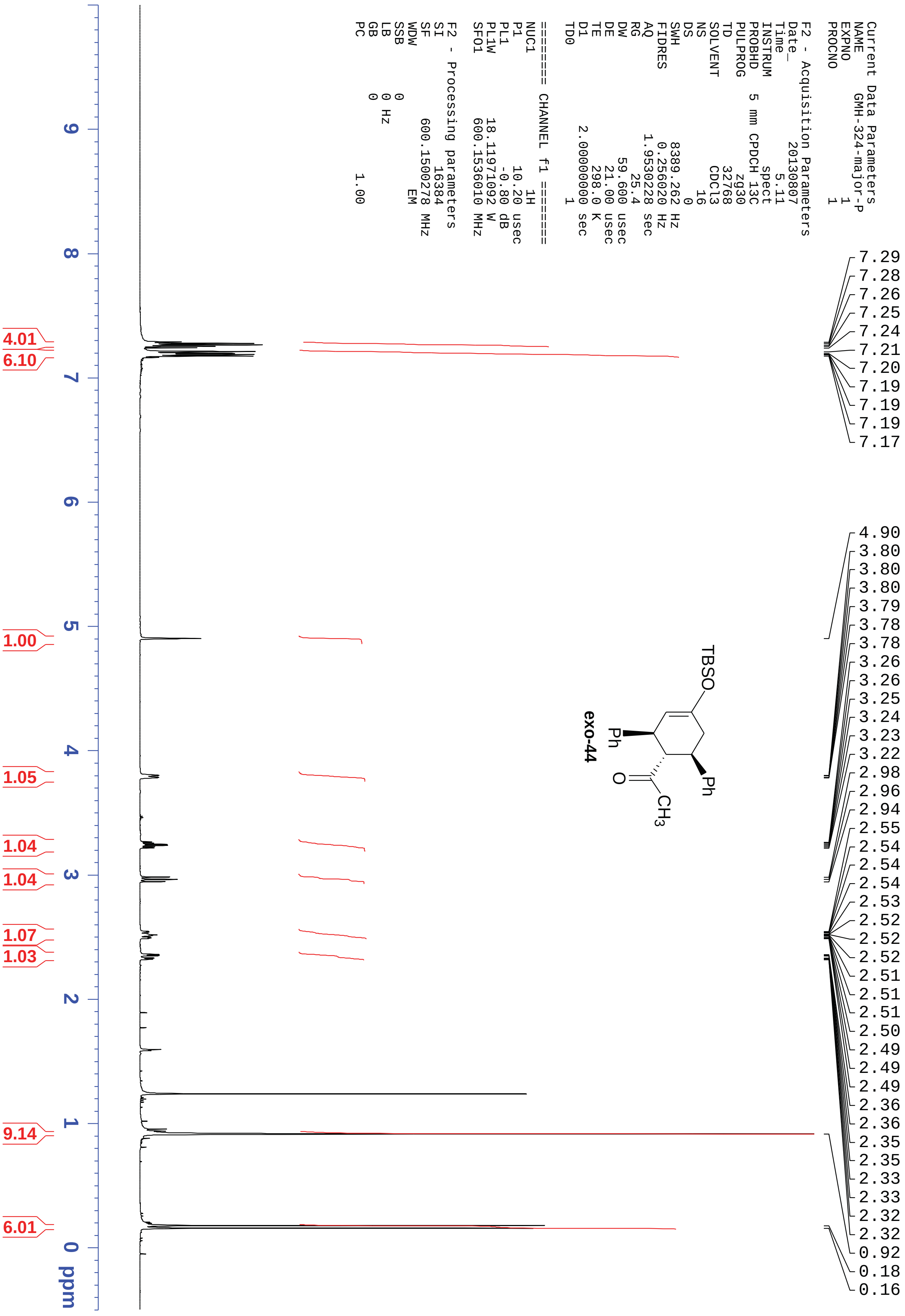

Supplementary Figure 157. ^{1}H NMR spectrum of compound exo-44.

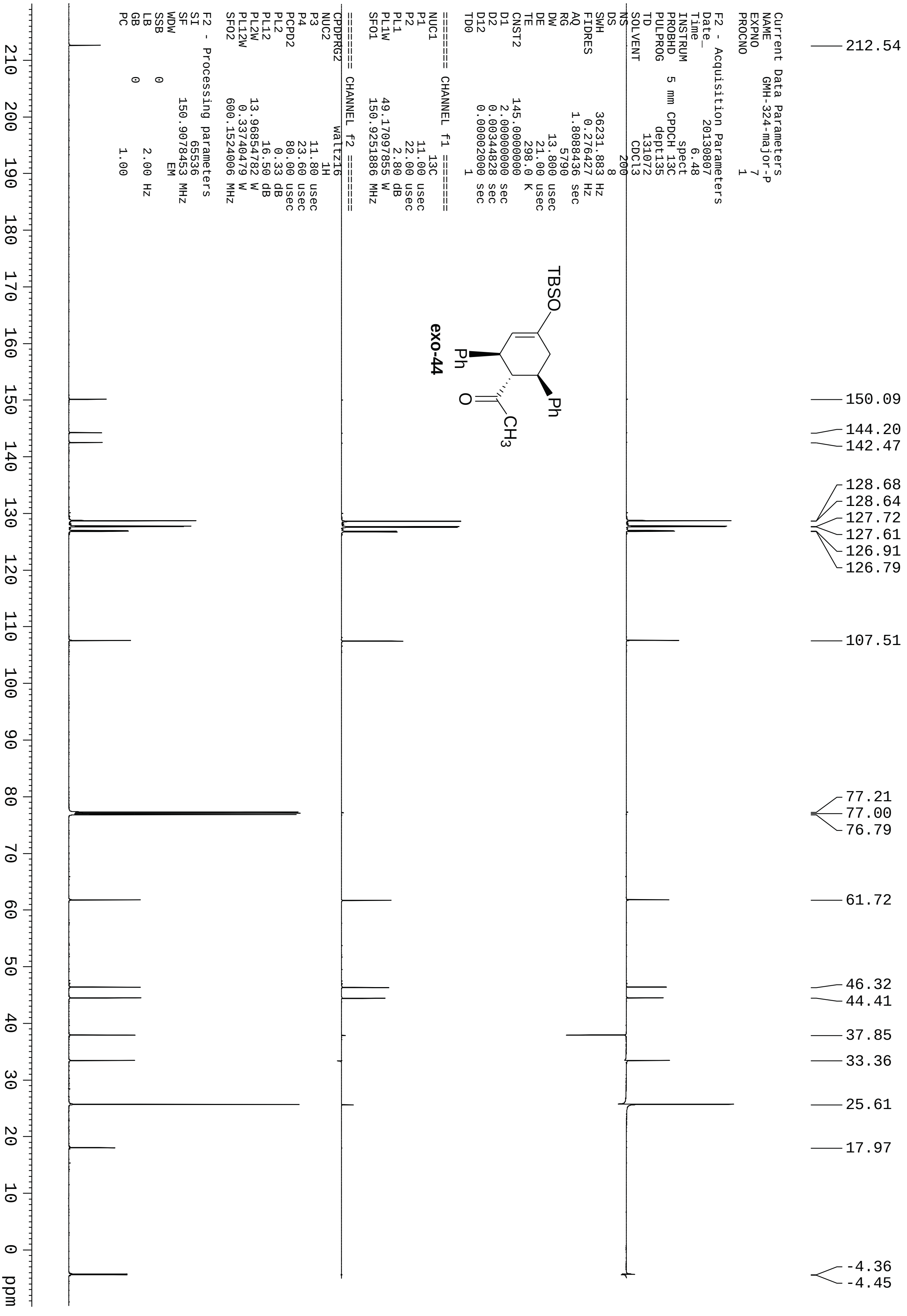

Supplementary Figure 158. ^{13}C and DEPT NMR spectra of compound exo-44.

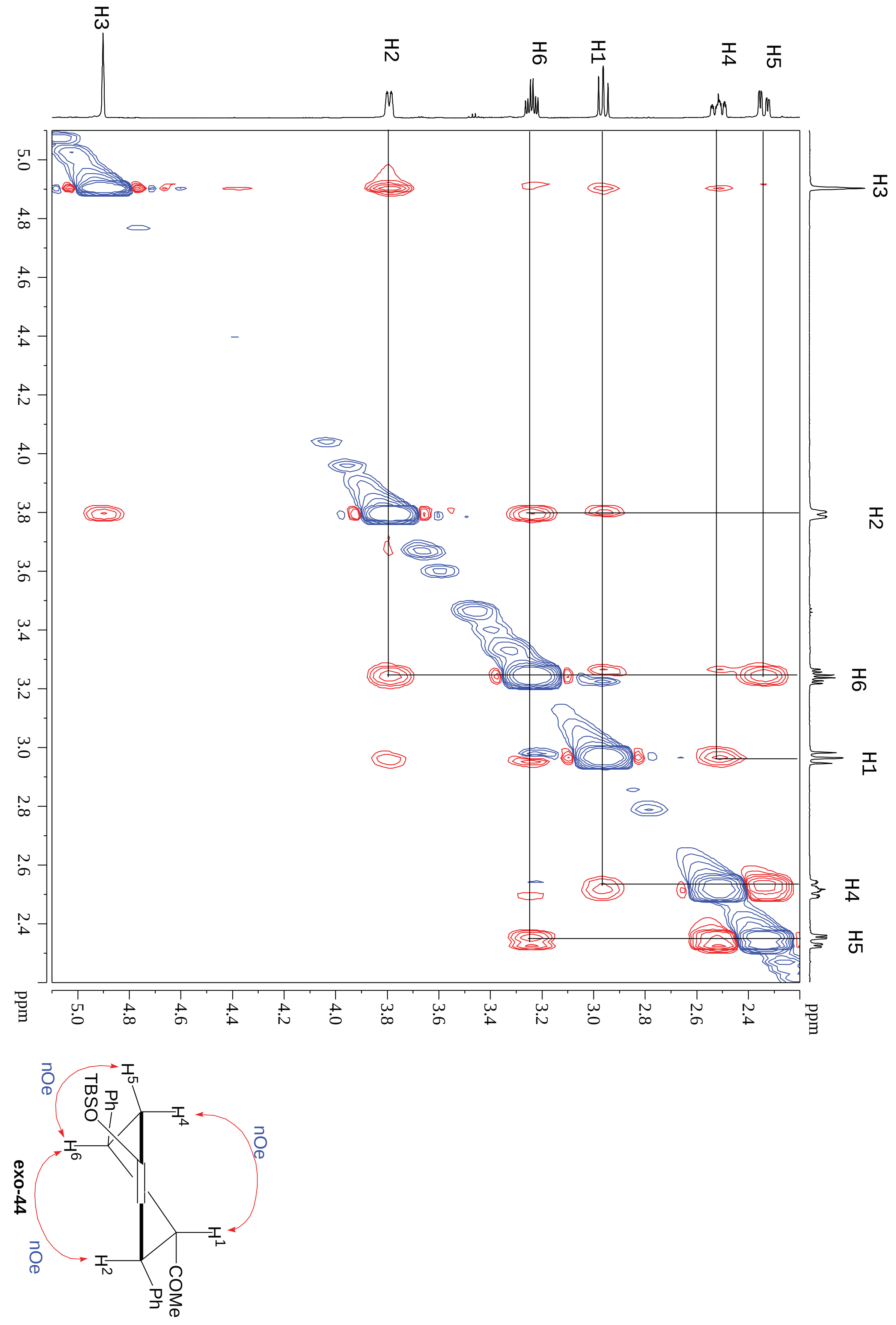

Supplementary Figure 159. NOESY NMR spectrum of compound exo-44.

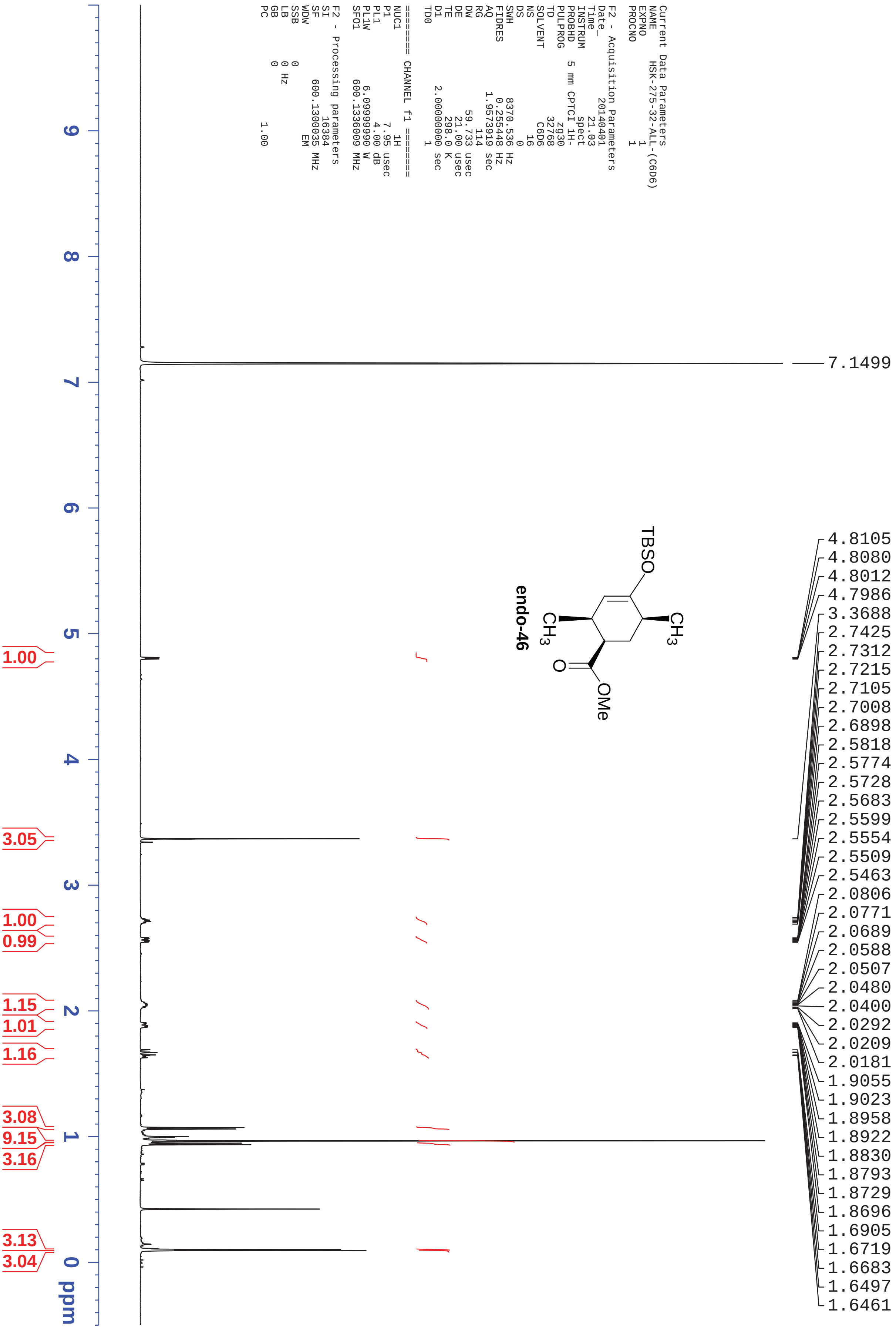

Supplementary Figure 160. ^{1}H NMR spectrum of compound endo-46.

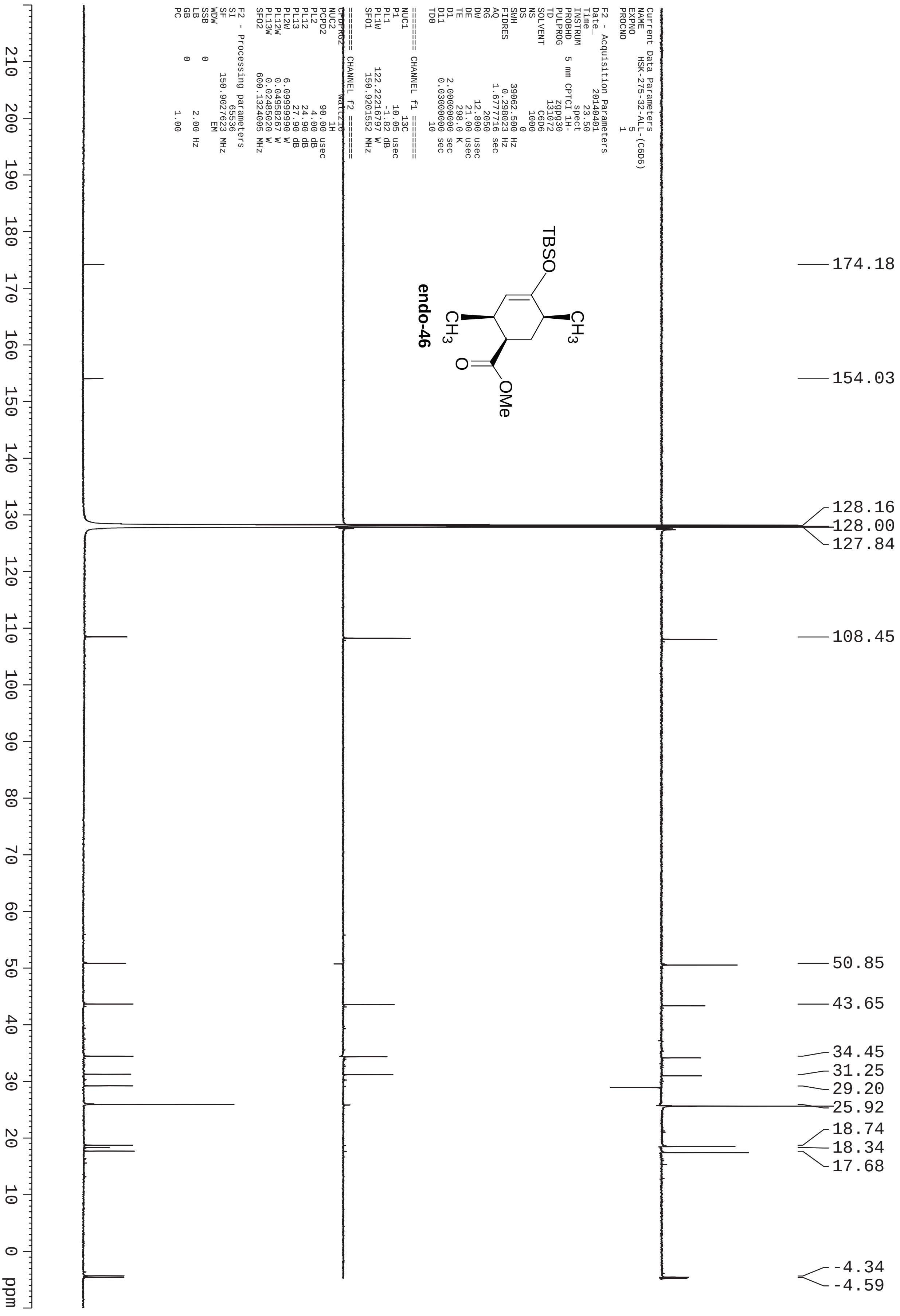

Supplementary Figure 161. ^{13}C and DEPT NMR spectra of compound endo-46.

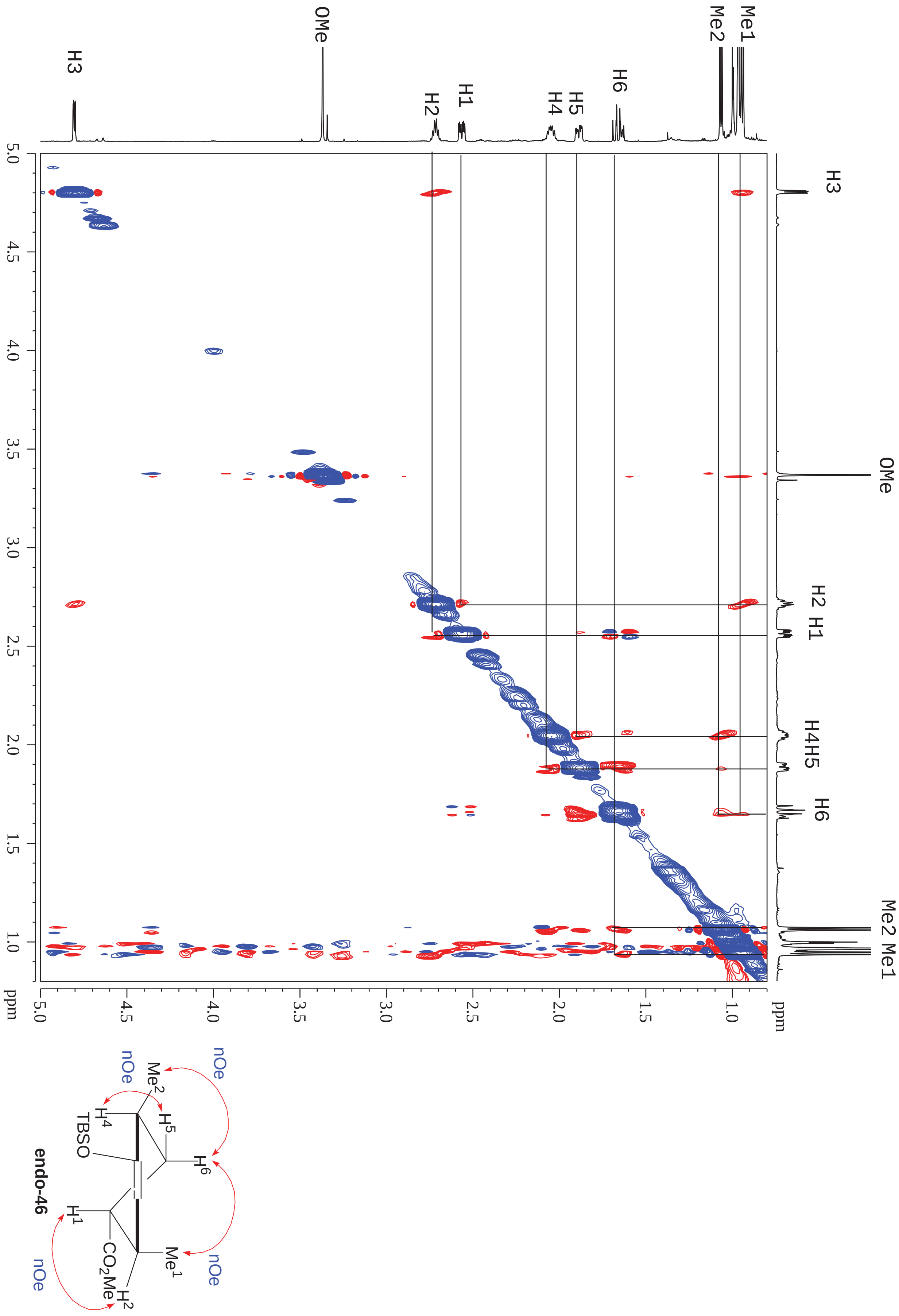

Supplementary Figure 162. NOESY NMR spectrum of compound endo-46.

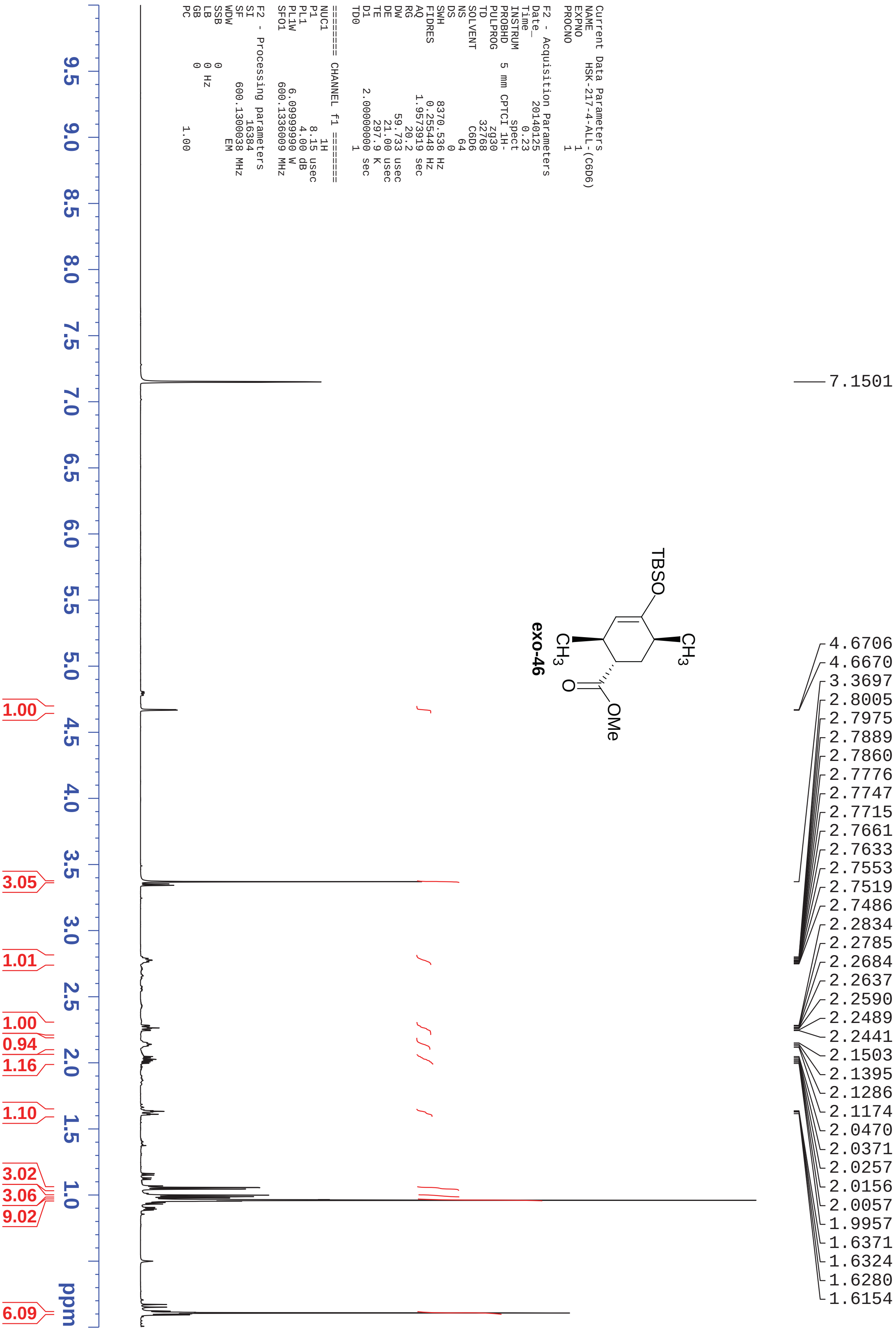

Supplementary Figure 163. ^{1}H NMR spectrum of compound exo-46.

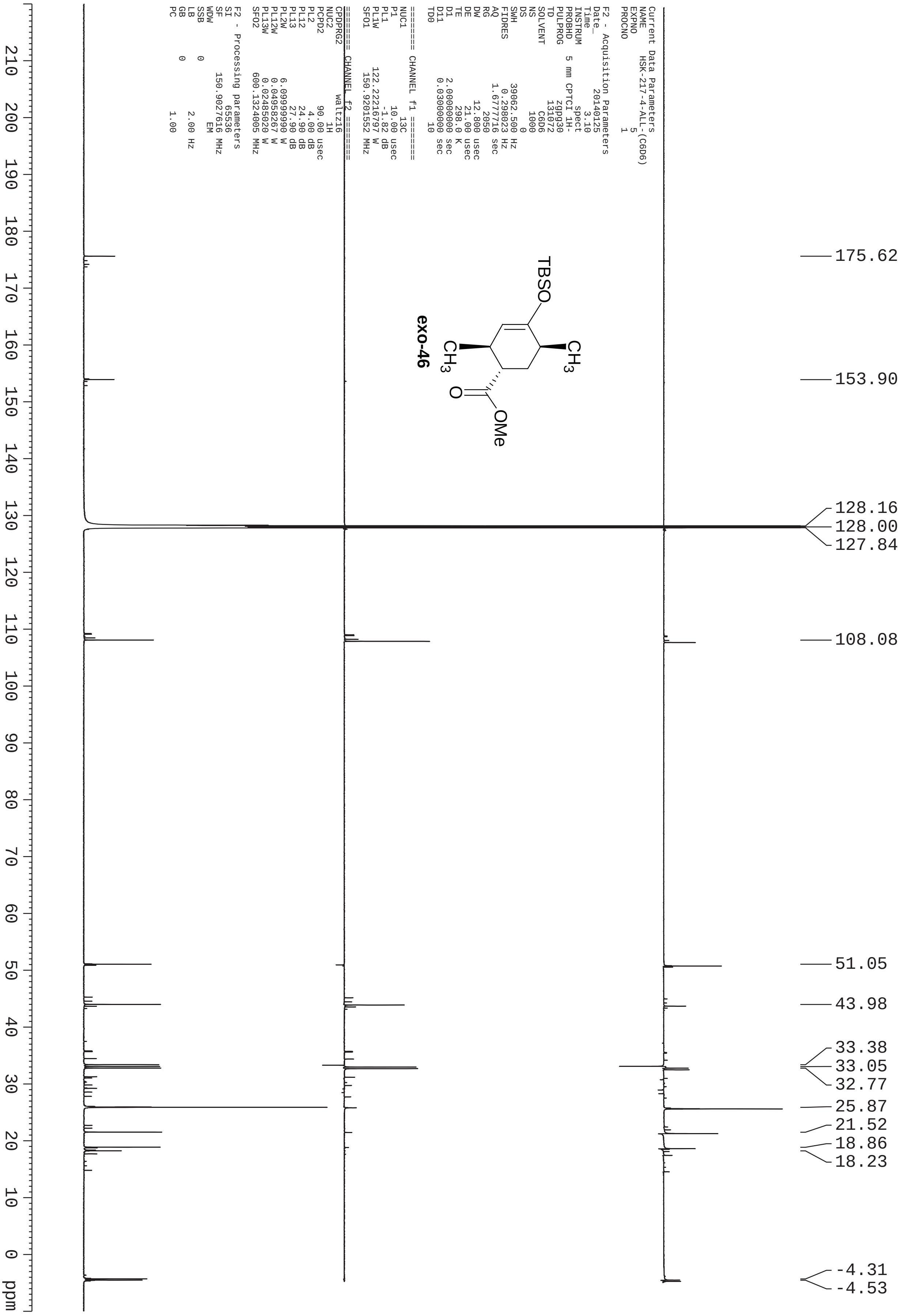

Supplementary Figure 164. ^{13}C and DEPT NMR spectra of compound exo-46.

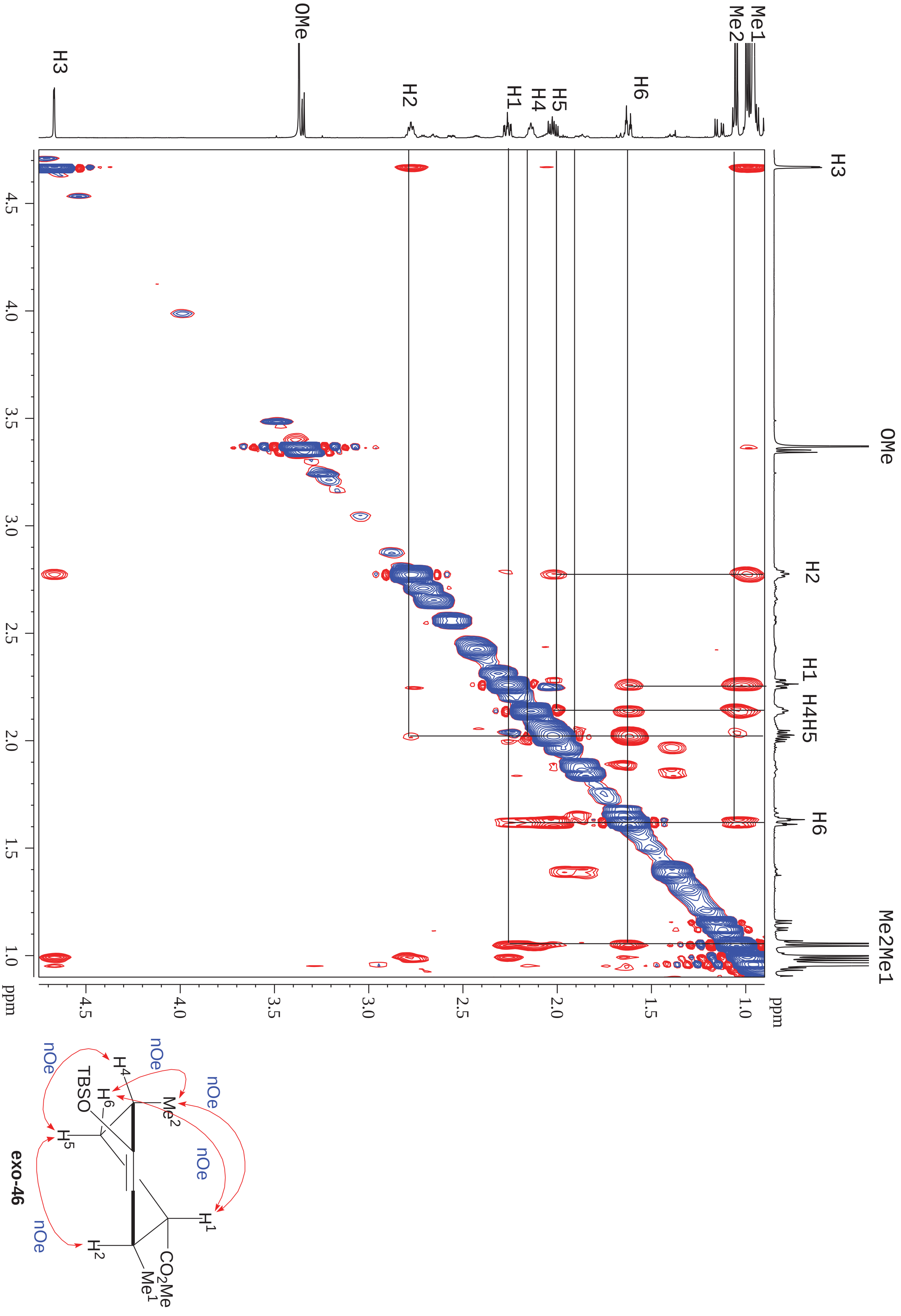

Supplementary Figure 165. NOESY NMR spectrum of compound exo-46.

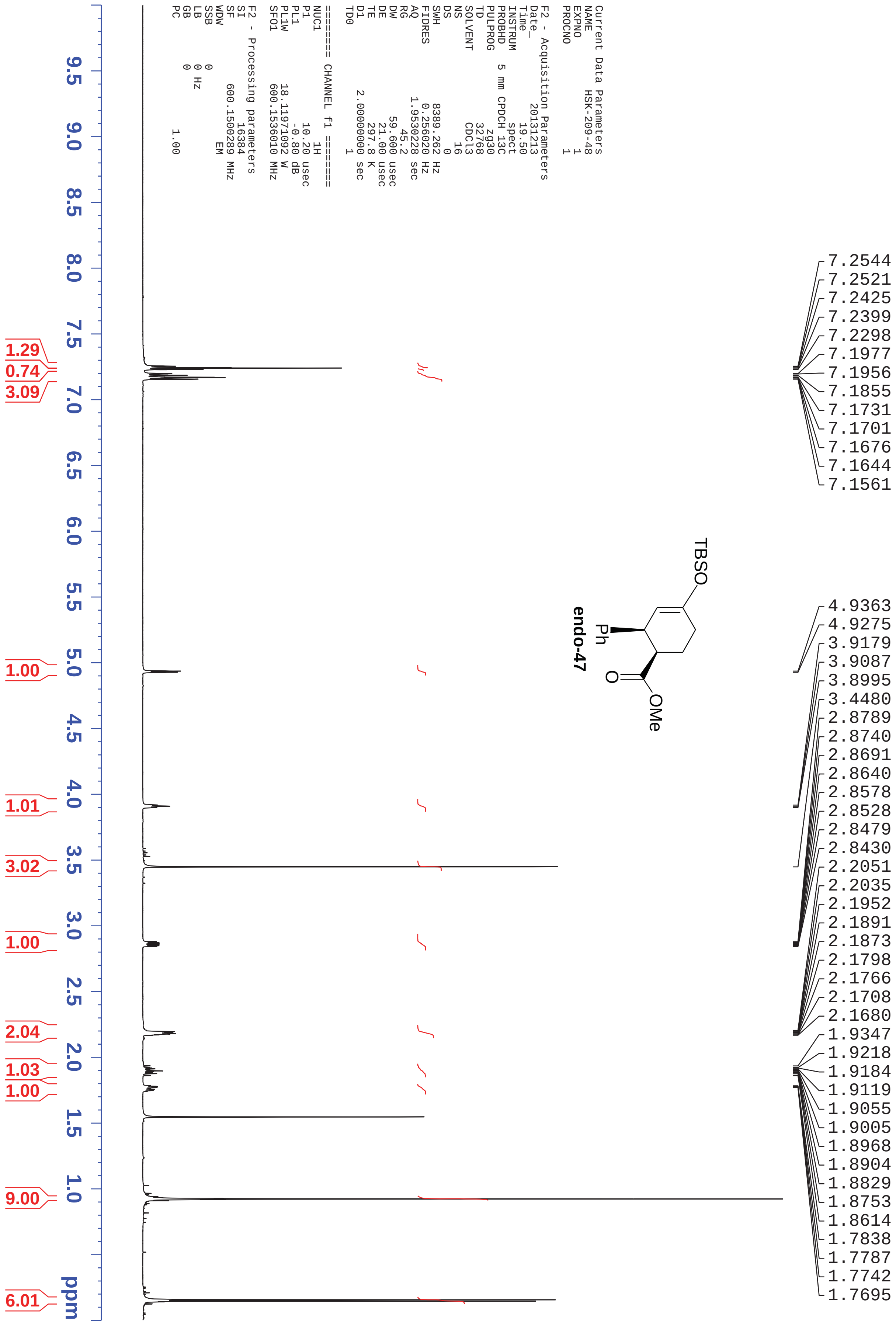

Supplementary Figure 166. ^{1}H NMR spectrum of compound endo-47.

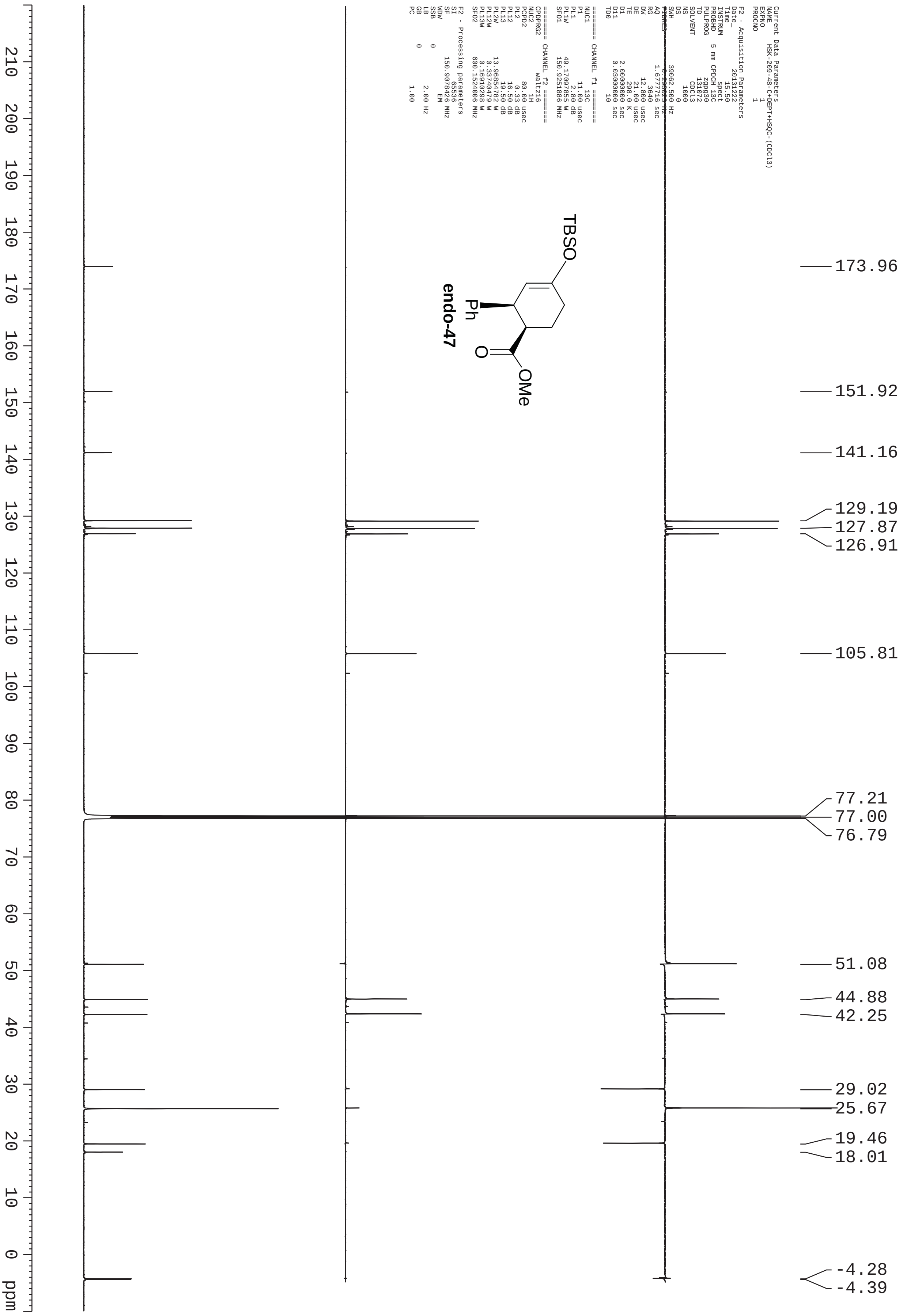

Supplementary Figure 167. ^{13}C and DEPT NMR spectra of compound endo-47.

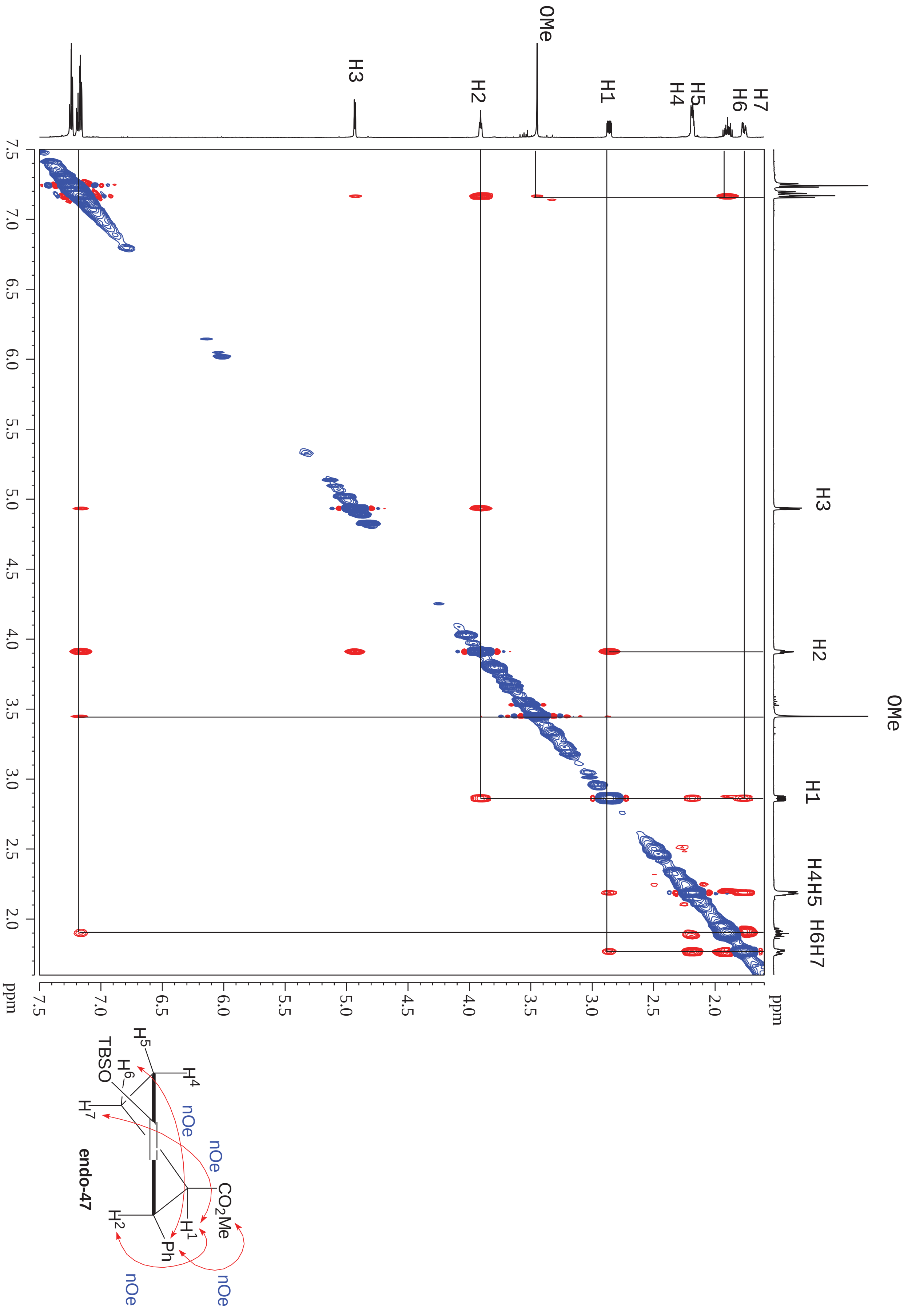

Supplementary Figure 168. NOESY NMR spectrum of compound endo-47.

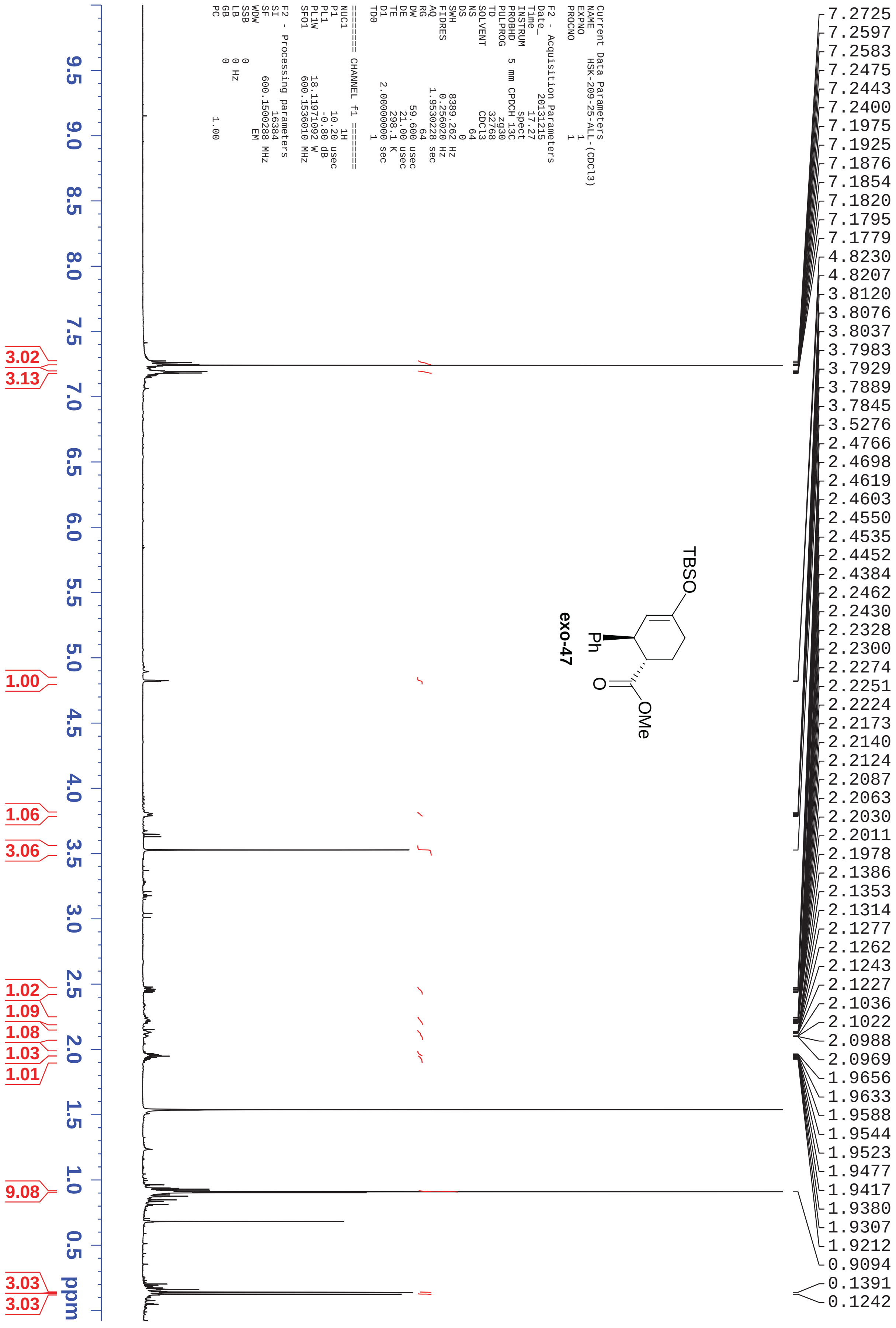

Supplementary Figure 169. ^{1}H NMR spectrum of compound exo-47.

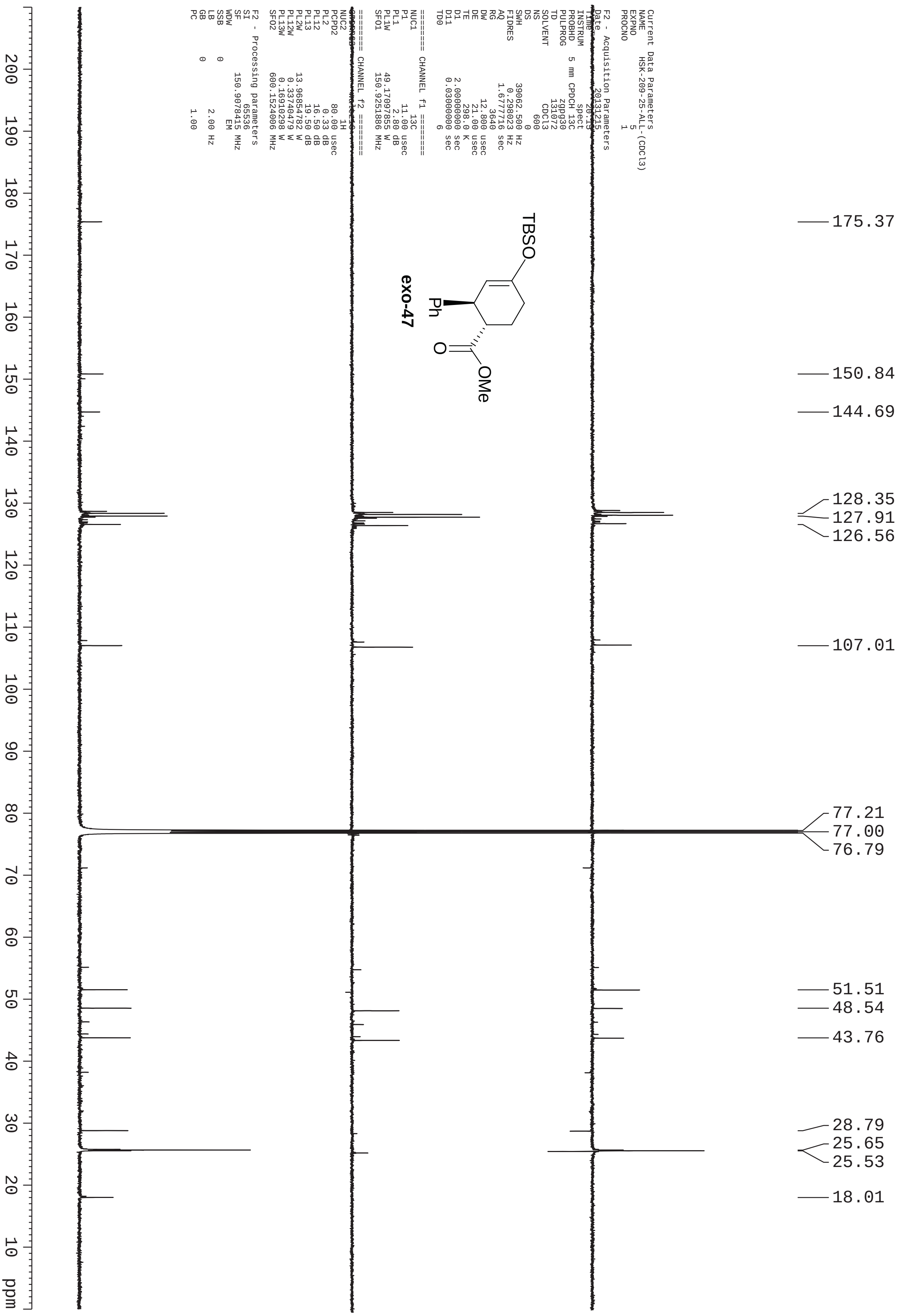

Supplementary Figure 170. ^{13}C and DEPT NMR spectra of compound exo-47.

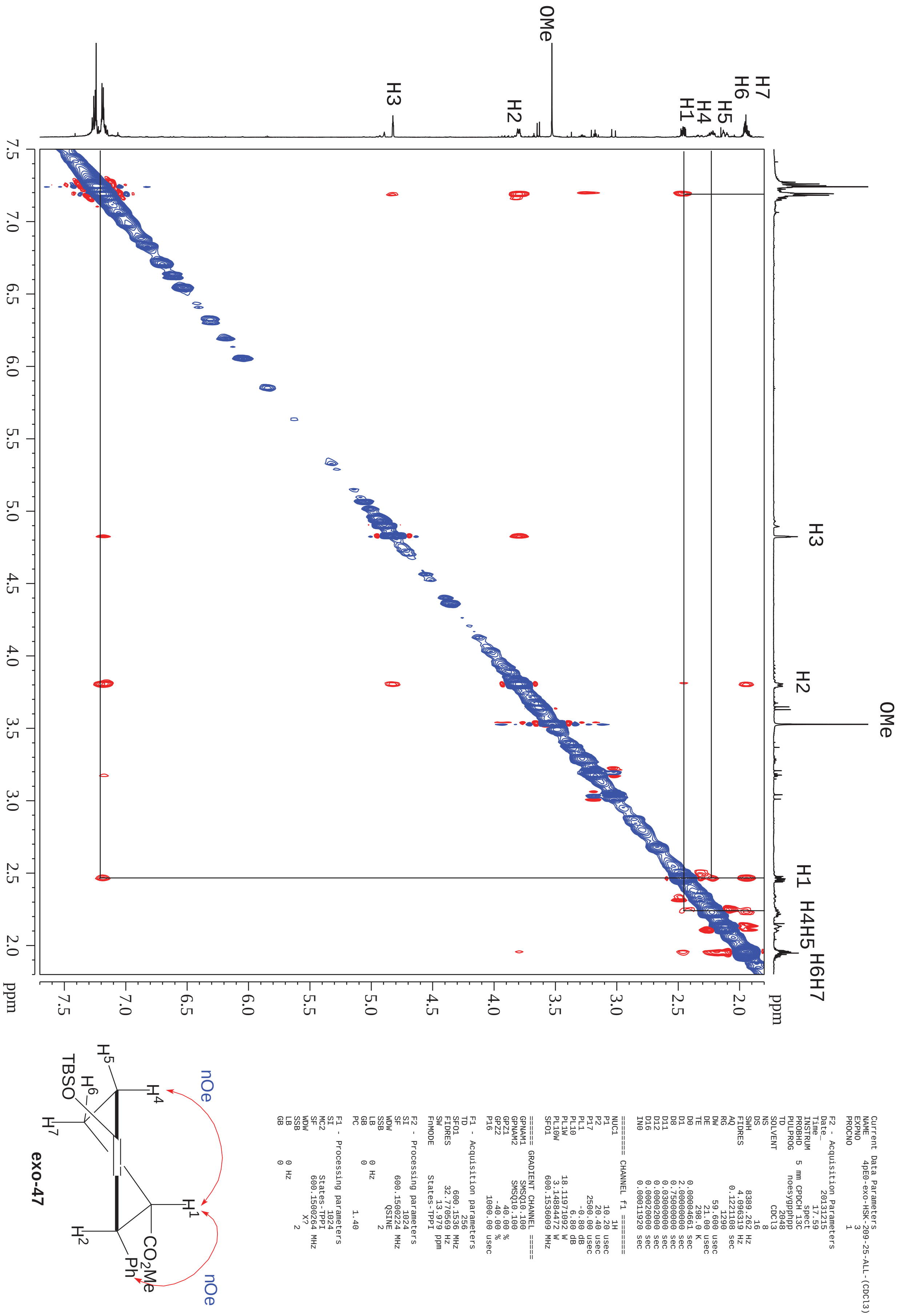

Supplementary Figure 171. NOESY NMR spectrum of compound exo-47.

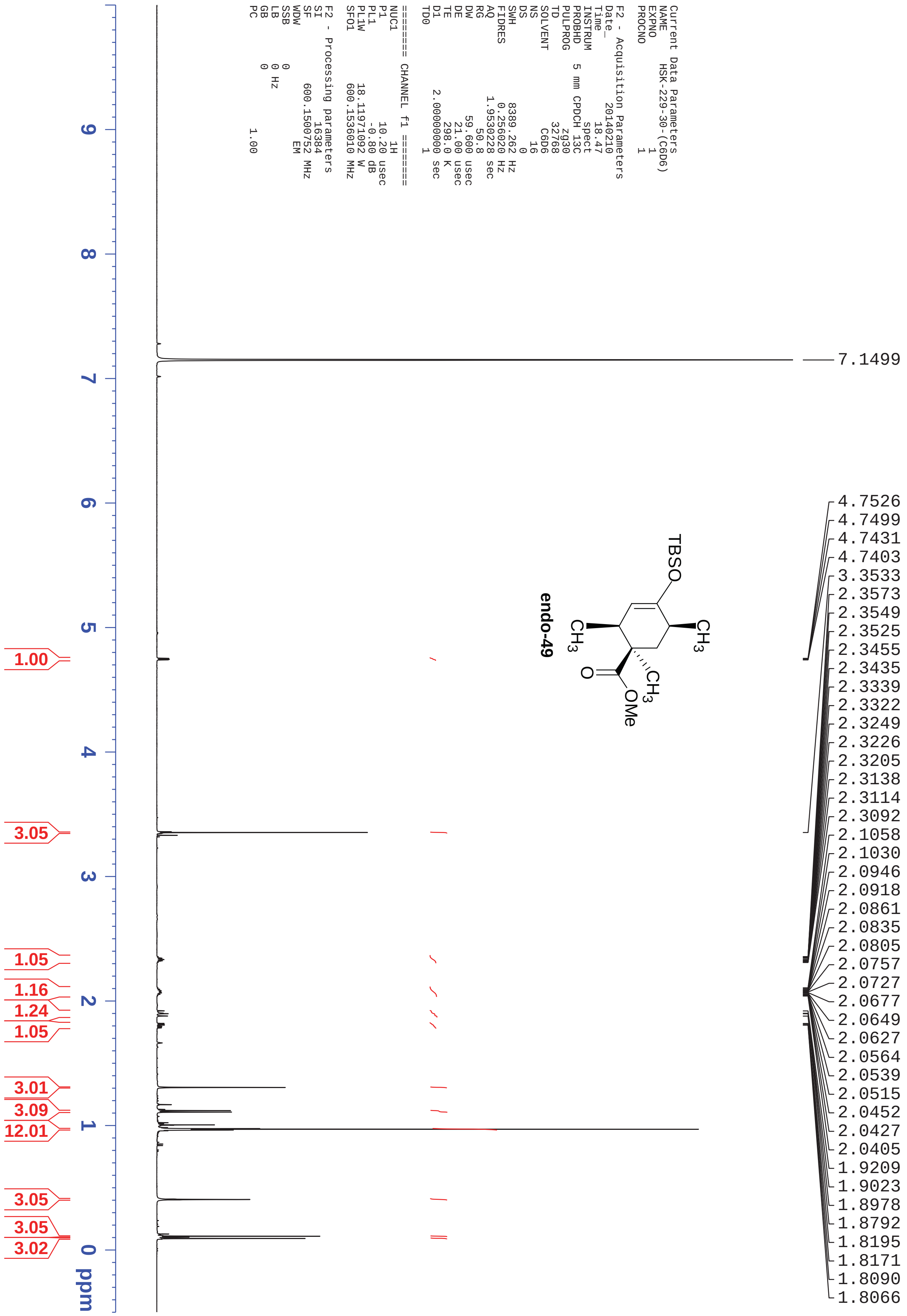

Supplementary Figure 172. ^{1}H NMR spectrum of compound endo-49.

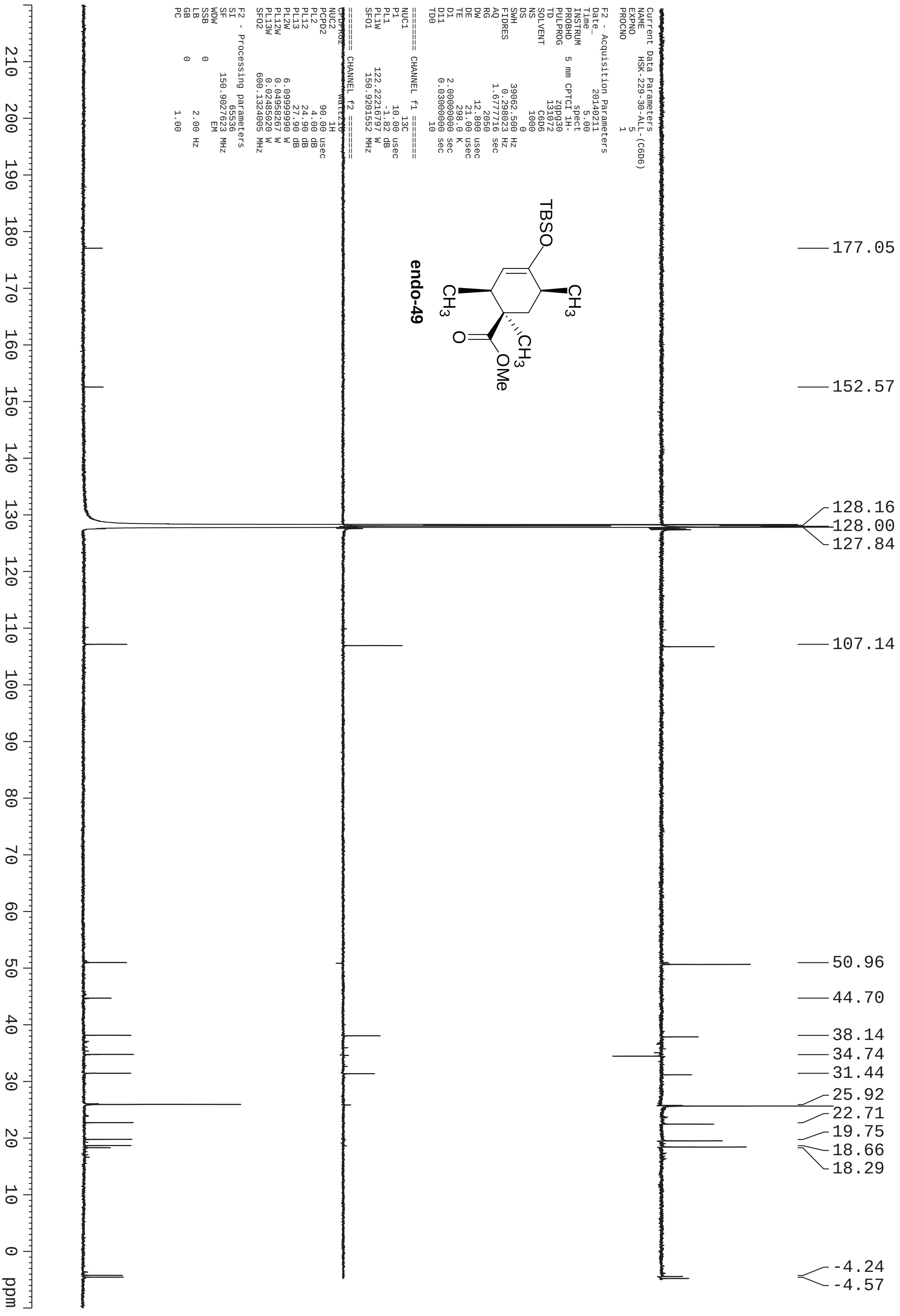

Supplementary Figure 173. ^{13}C and DEPT NMR spectra of compound endo-49.

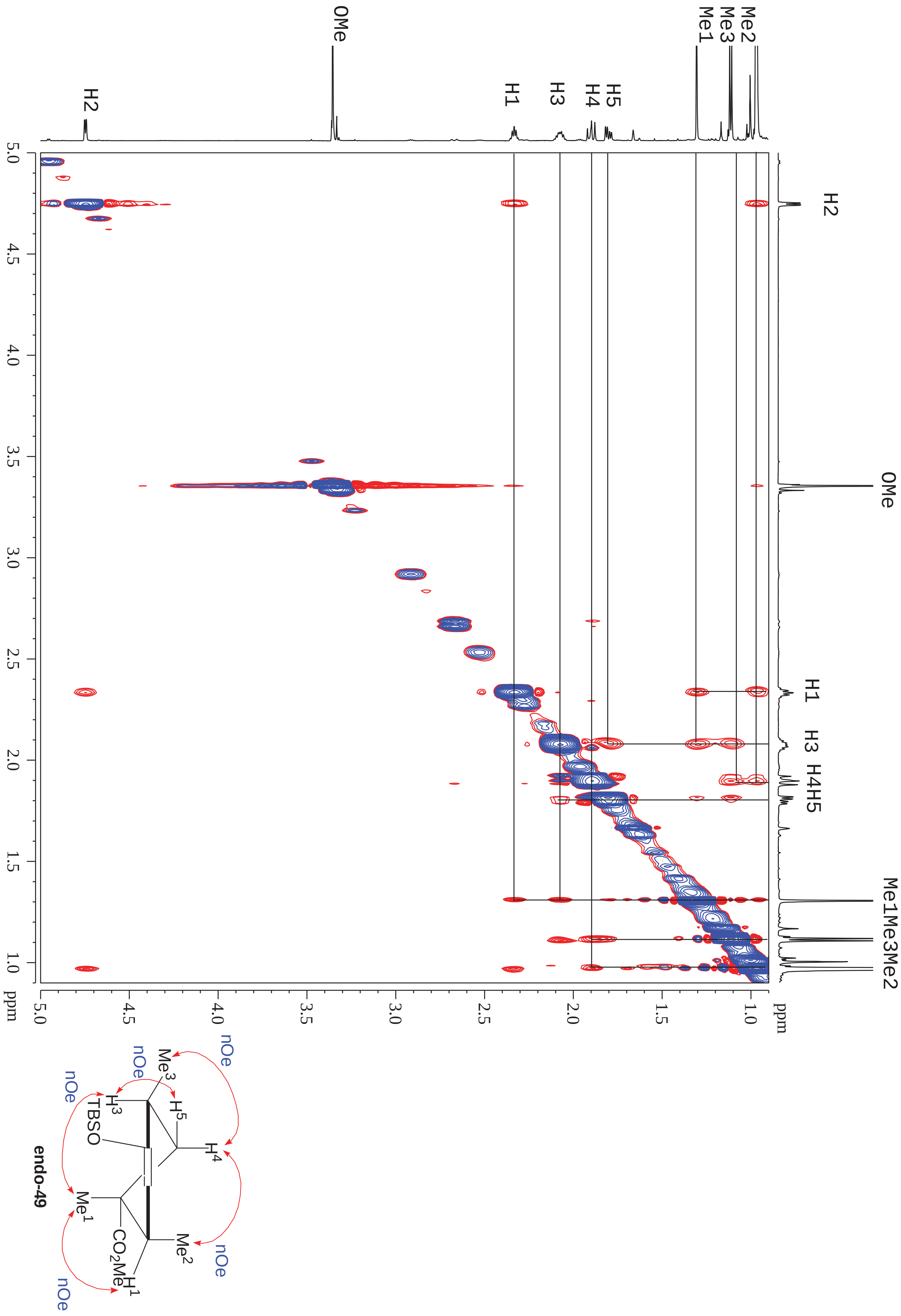

Supplementary Figure 174. NOESY NMR spectrum of compound endo-49.

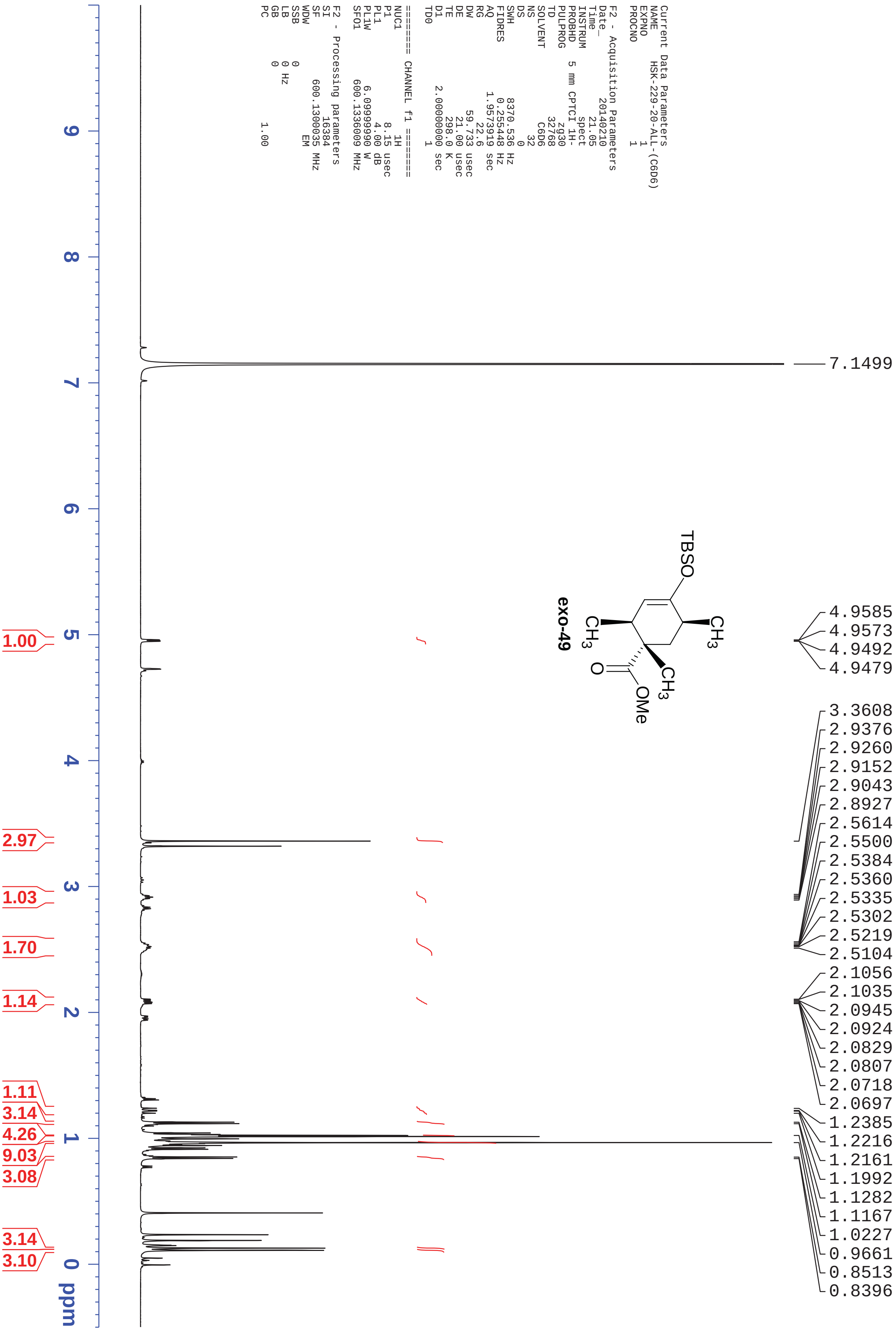

Supplementary Figure 175. ^{1}**H NMR spectrum of compound exo-49.**

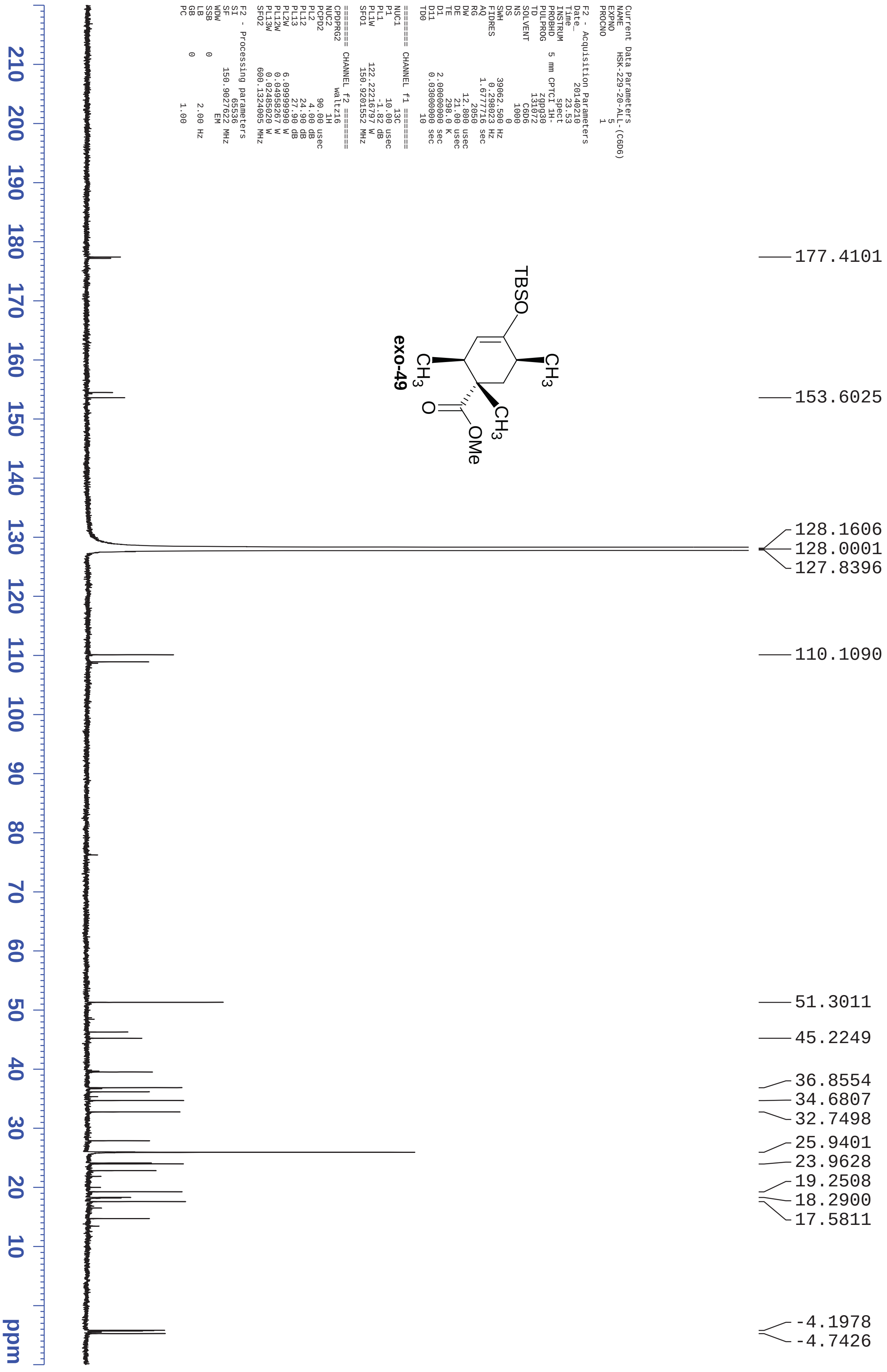

Supplementary Figure 176. ^{13}C NMR spectrum of compound exo-49.

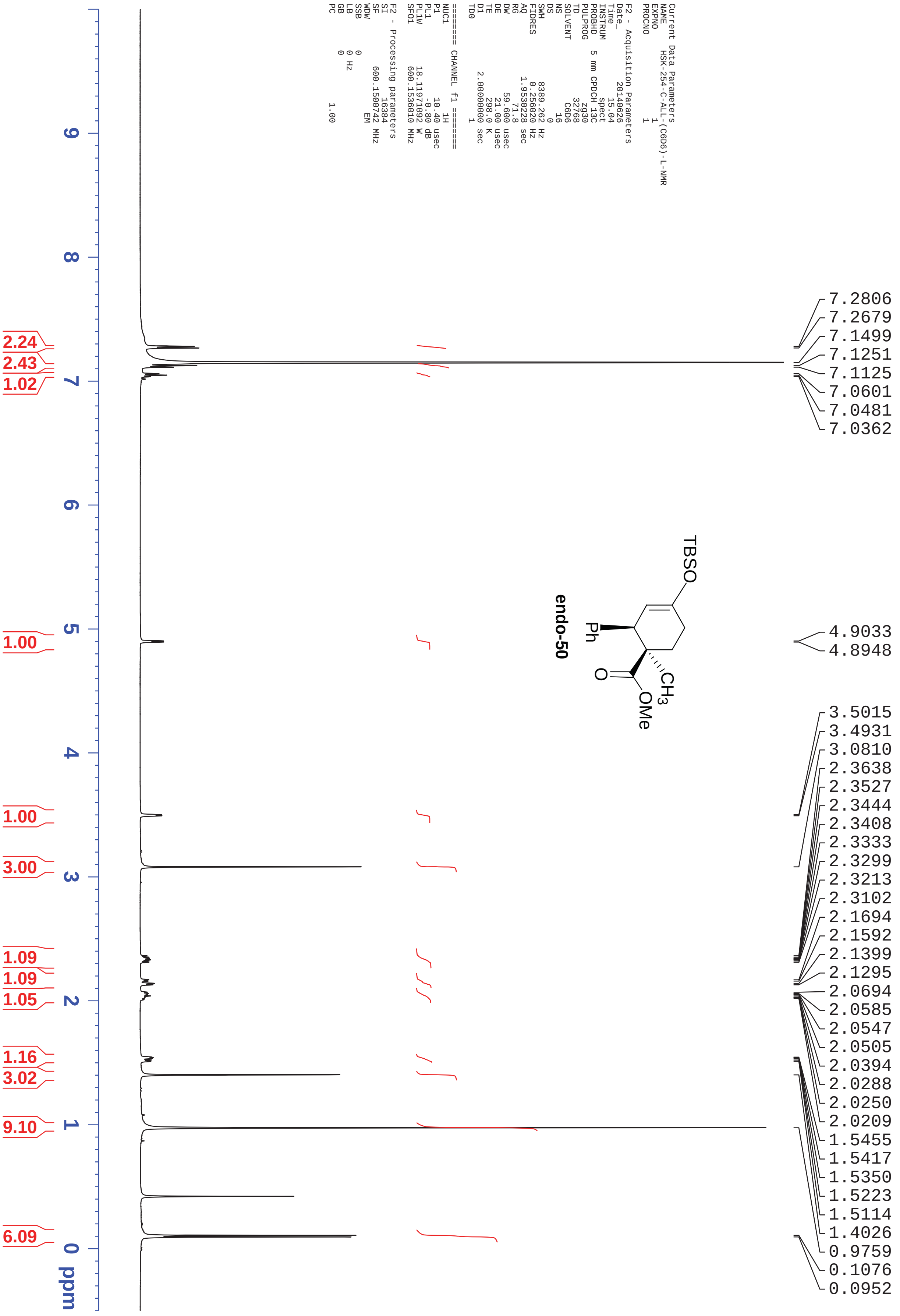

Supplementary Figure 177. ^{1}**H NMR spectrum of compound endo-50.**

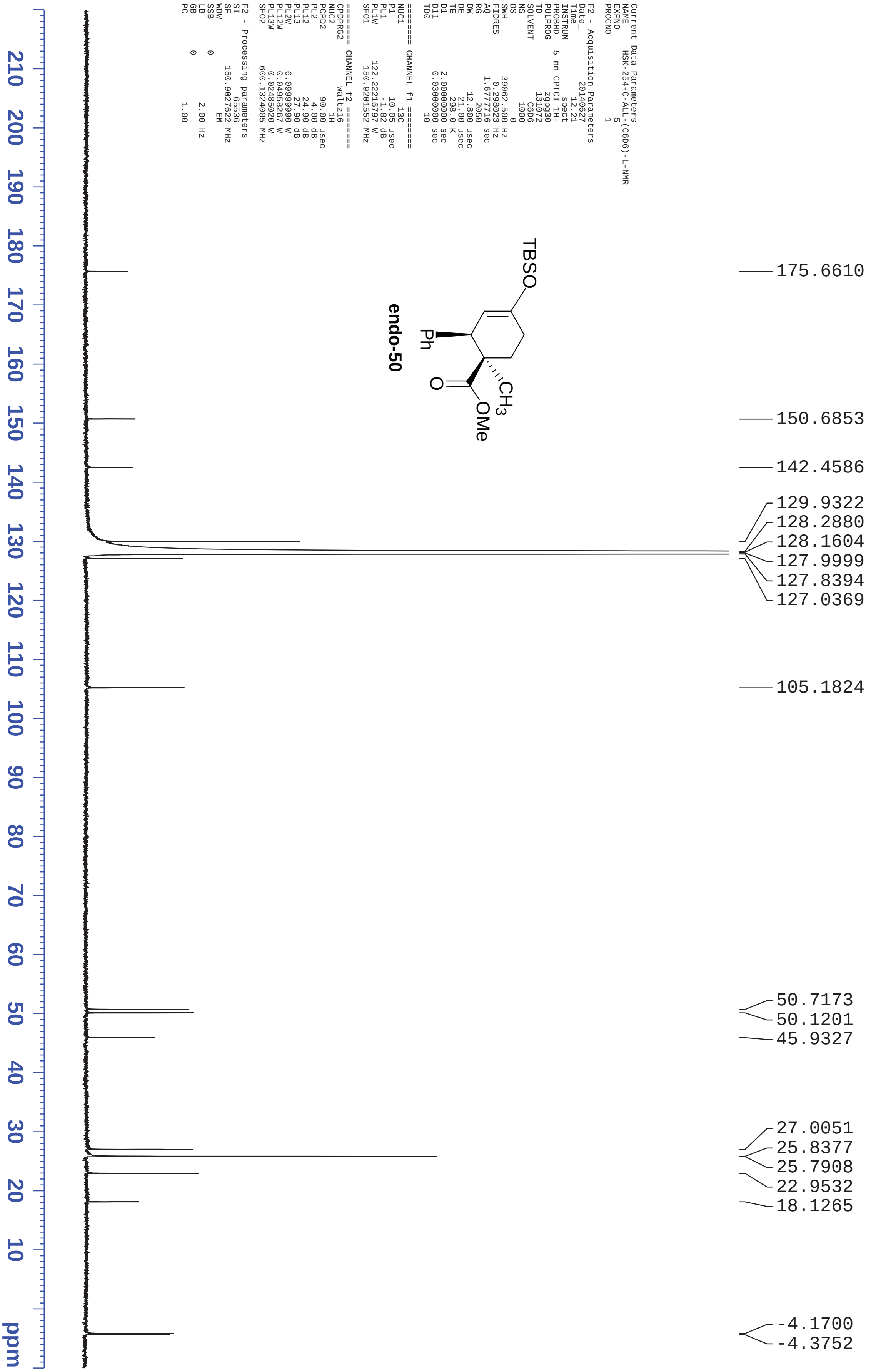

Supplementary Figure 178. ^{13}C NMR spectrum of compound endo-50.

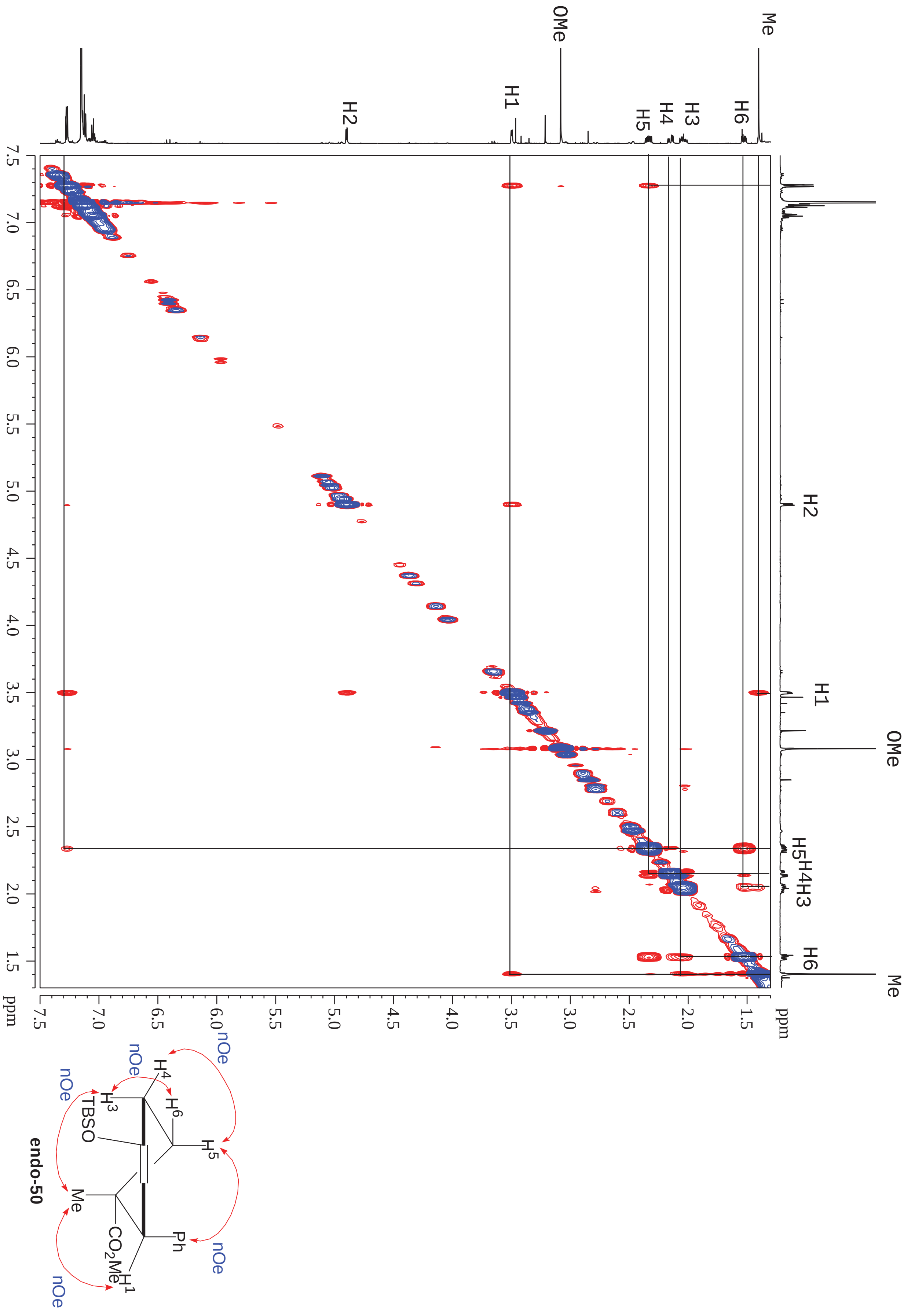

Supplementary Figure 179. NOESY NMR spectrum of compound endo-50.

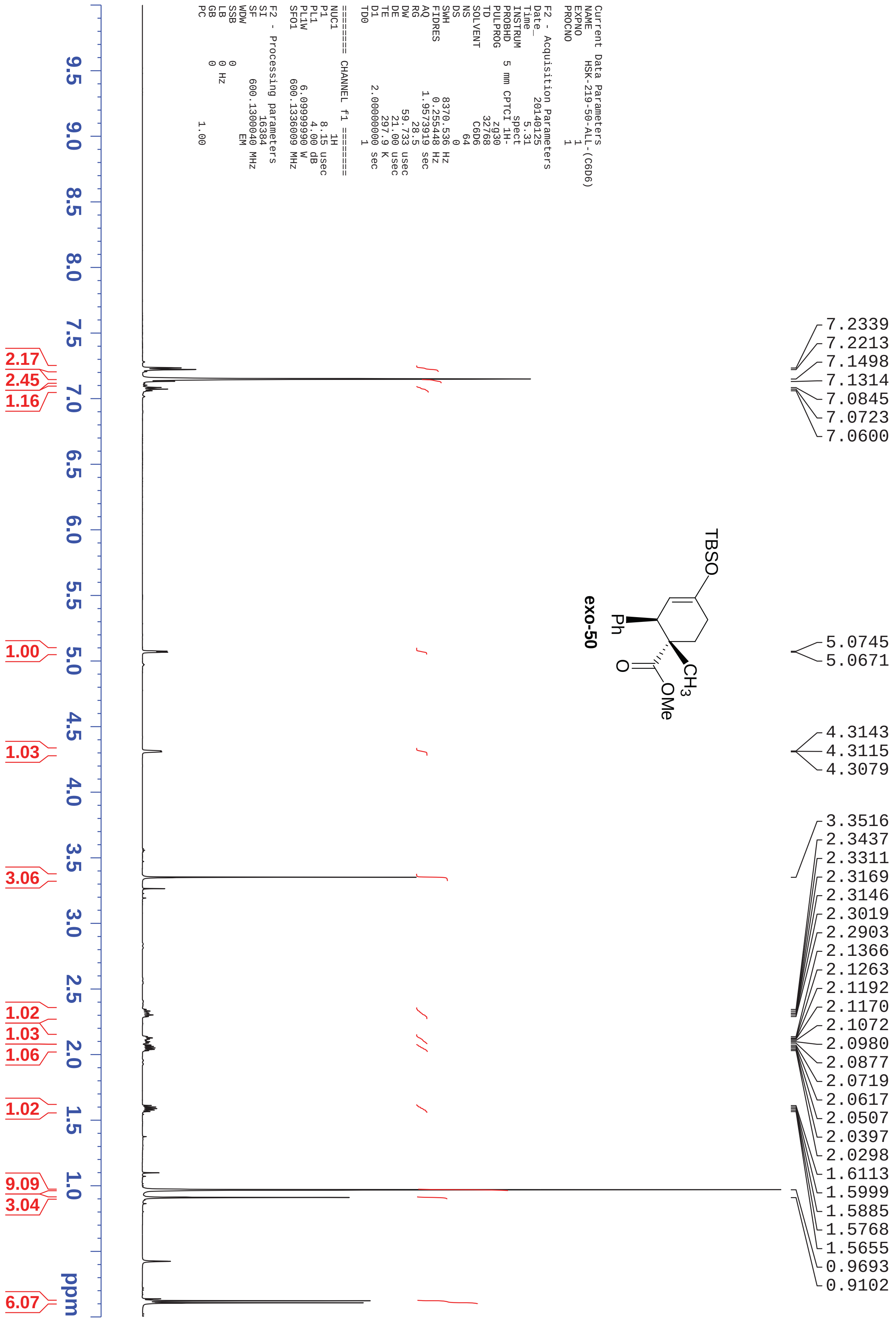

Supplementary Figure 180. ^{1}H NMR spectrum of compound exo-50.

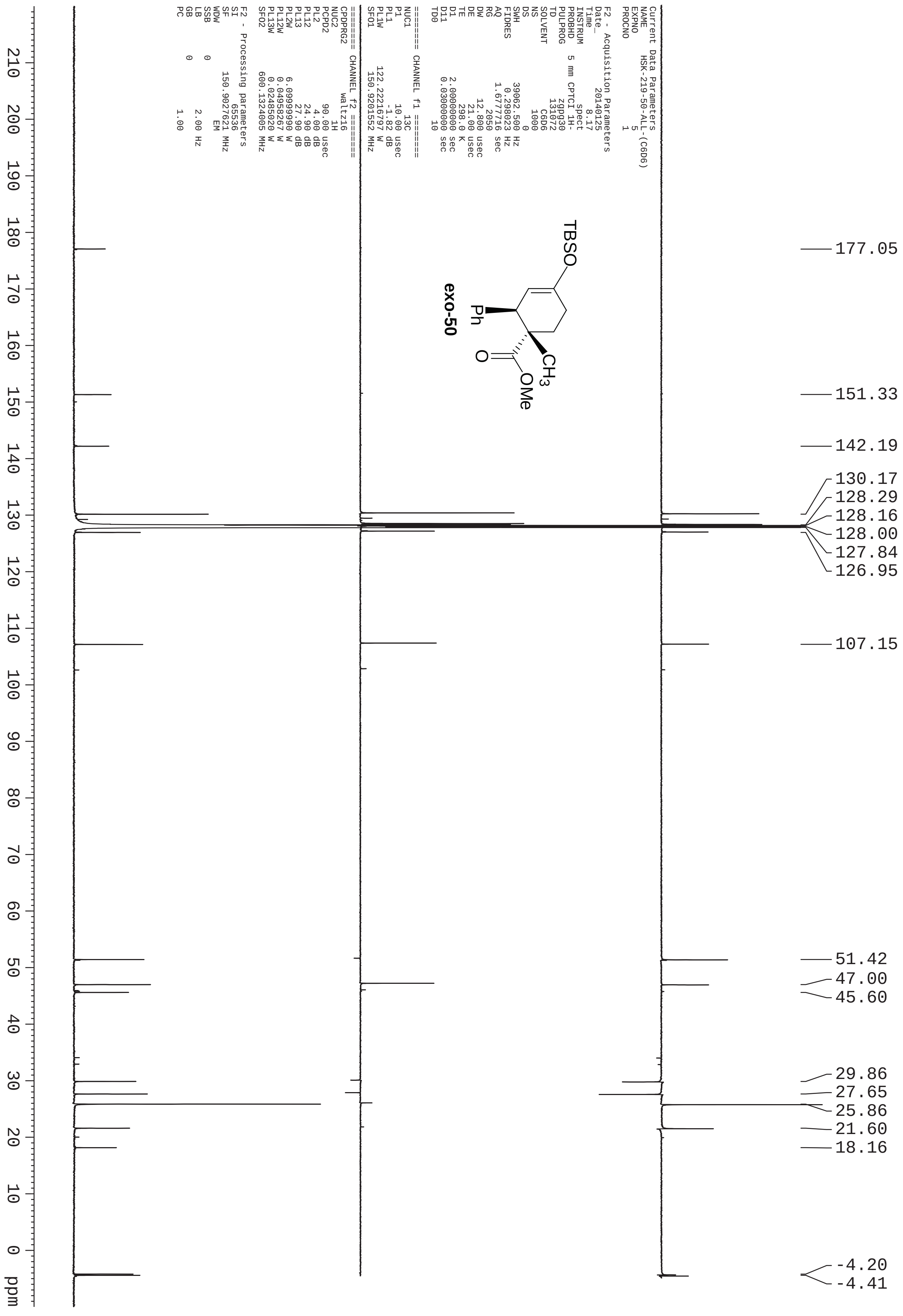

Supplementary Figure 181. ^{13}C and DEPT NMR spectra of compound exo-50.

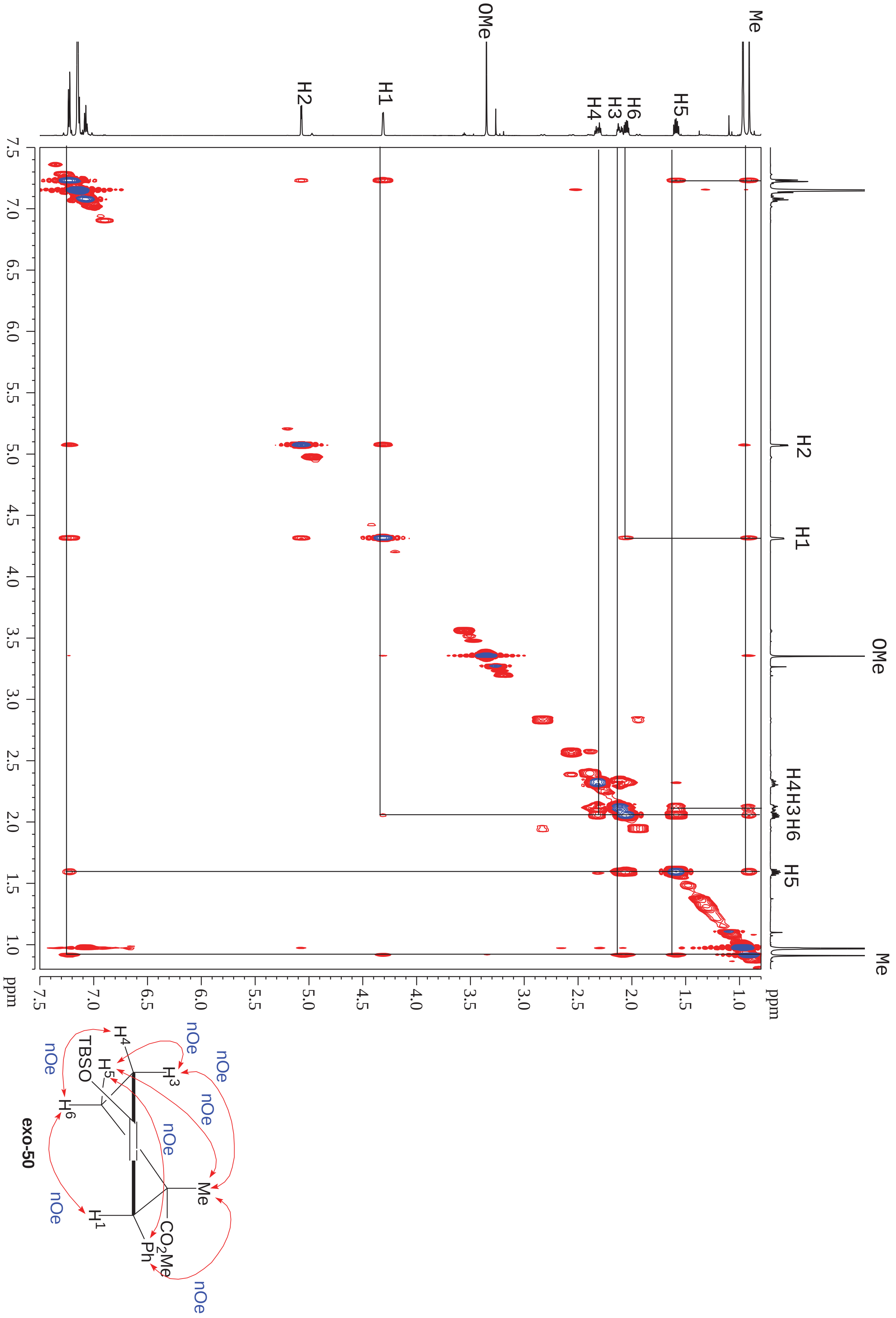

Supplementary Figure 182. NOESY NMR spectrum of compound exo-50.

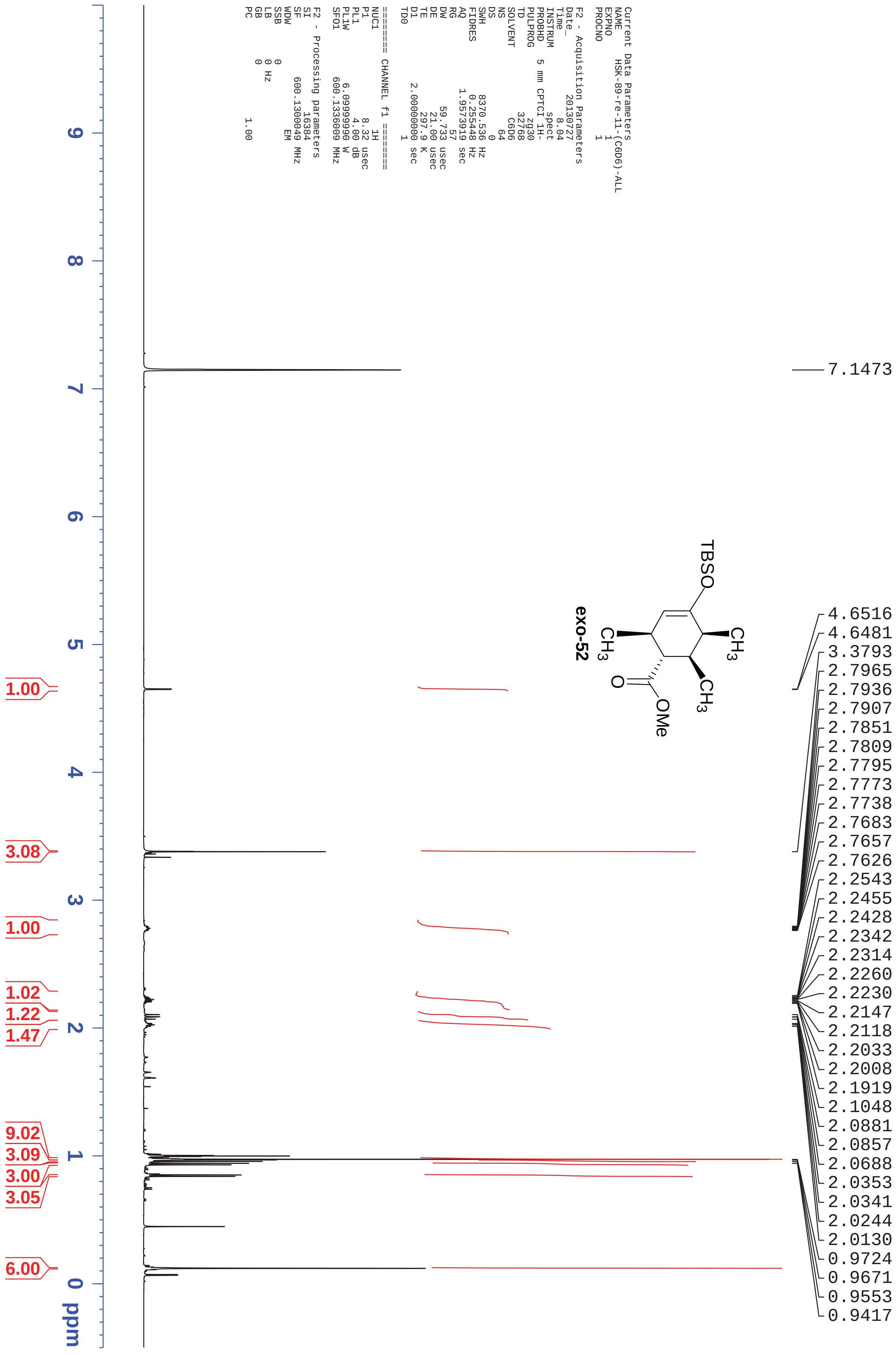

Supplementary Figure 183. ^{1}H NMR spectrum of compound exo-52.

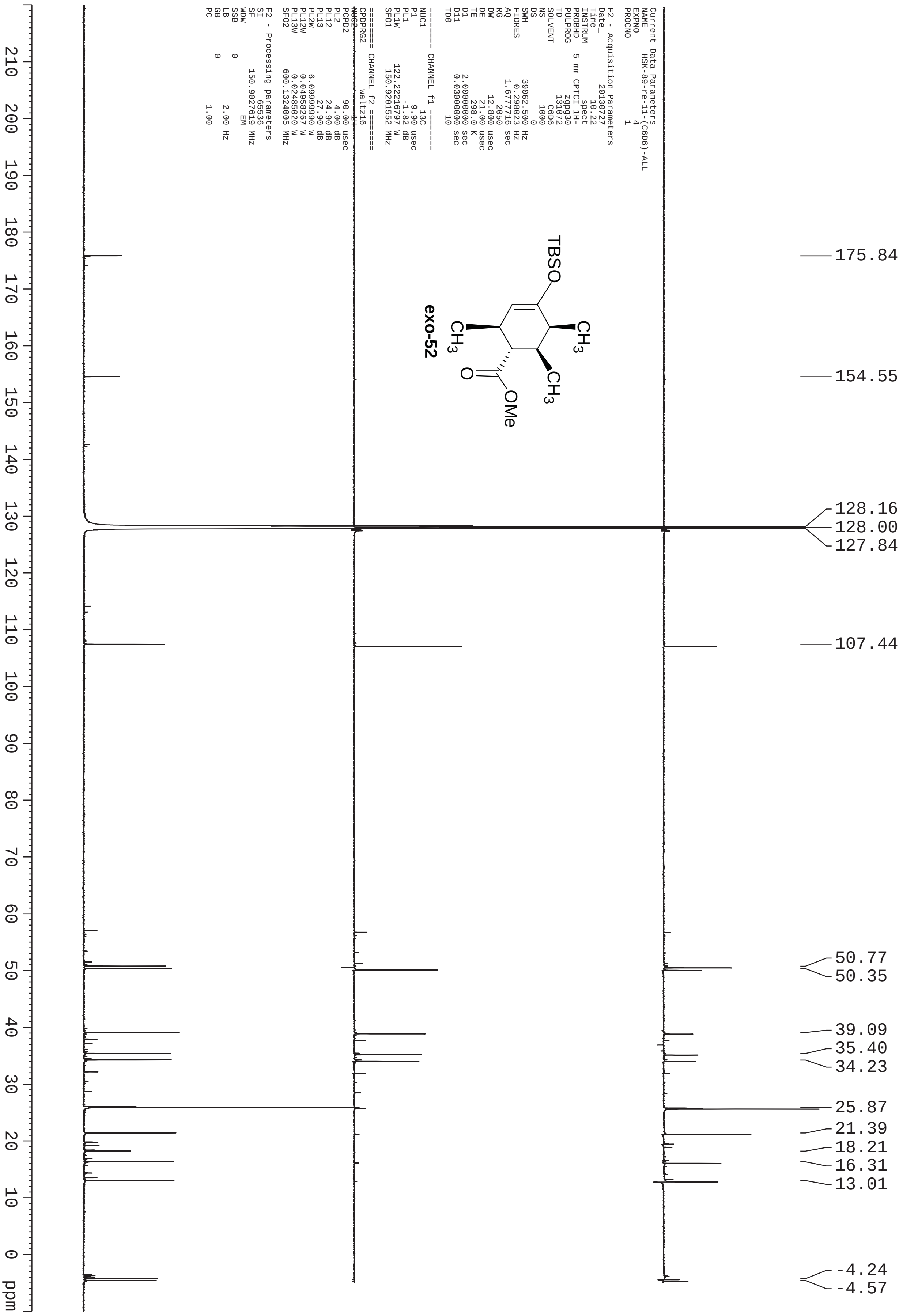

Supplementary Figure 184. ^{13}C and DEPT NMR spectra of compound exo-52.

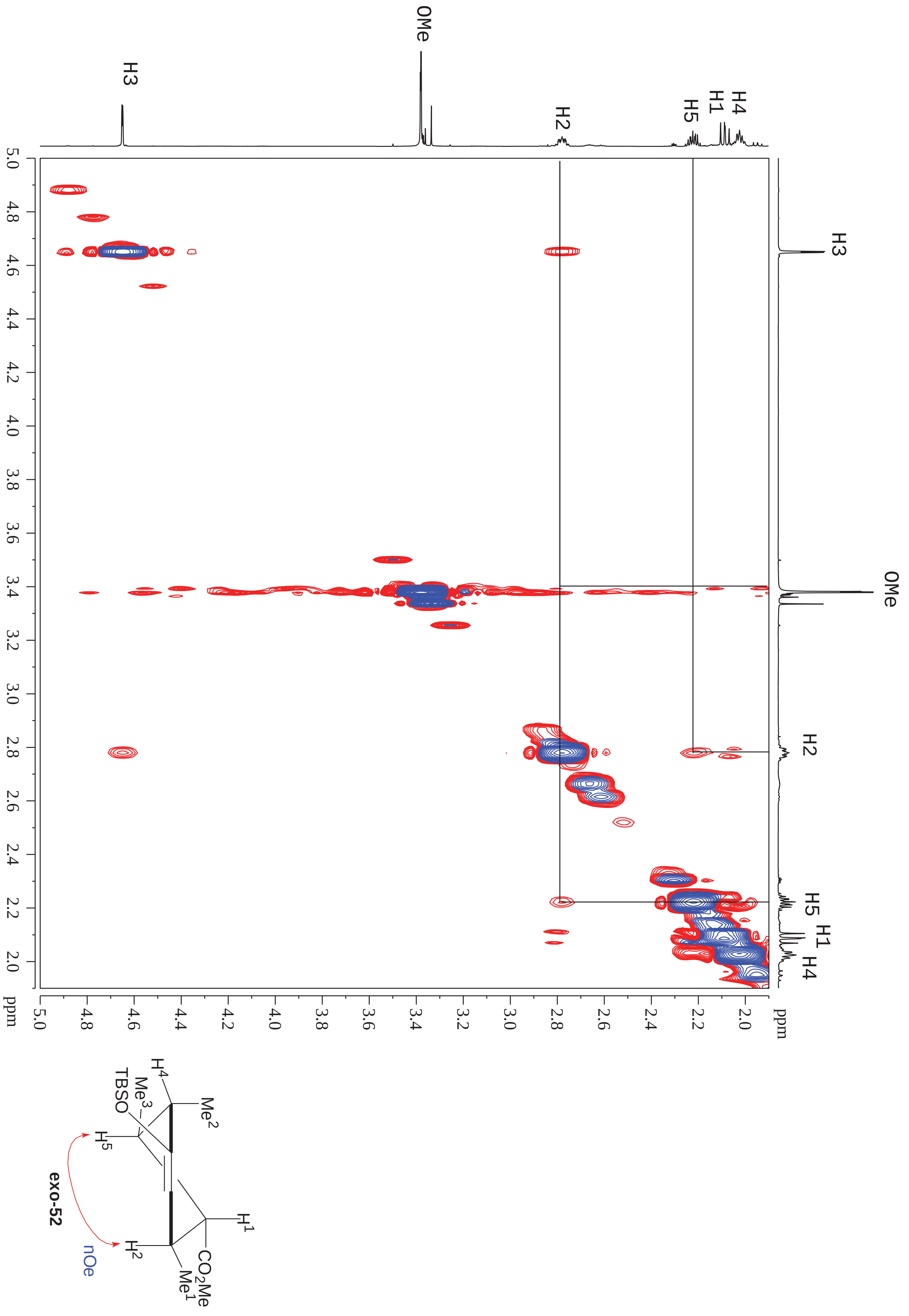

Supplementary Figure 185. NOESY NMR spectrum of compound exo-52.

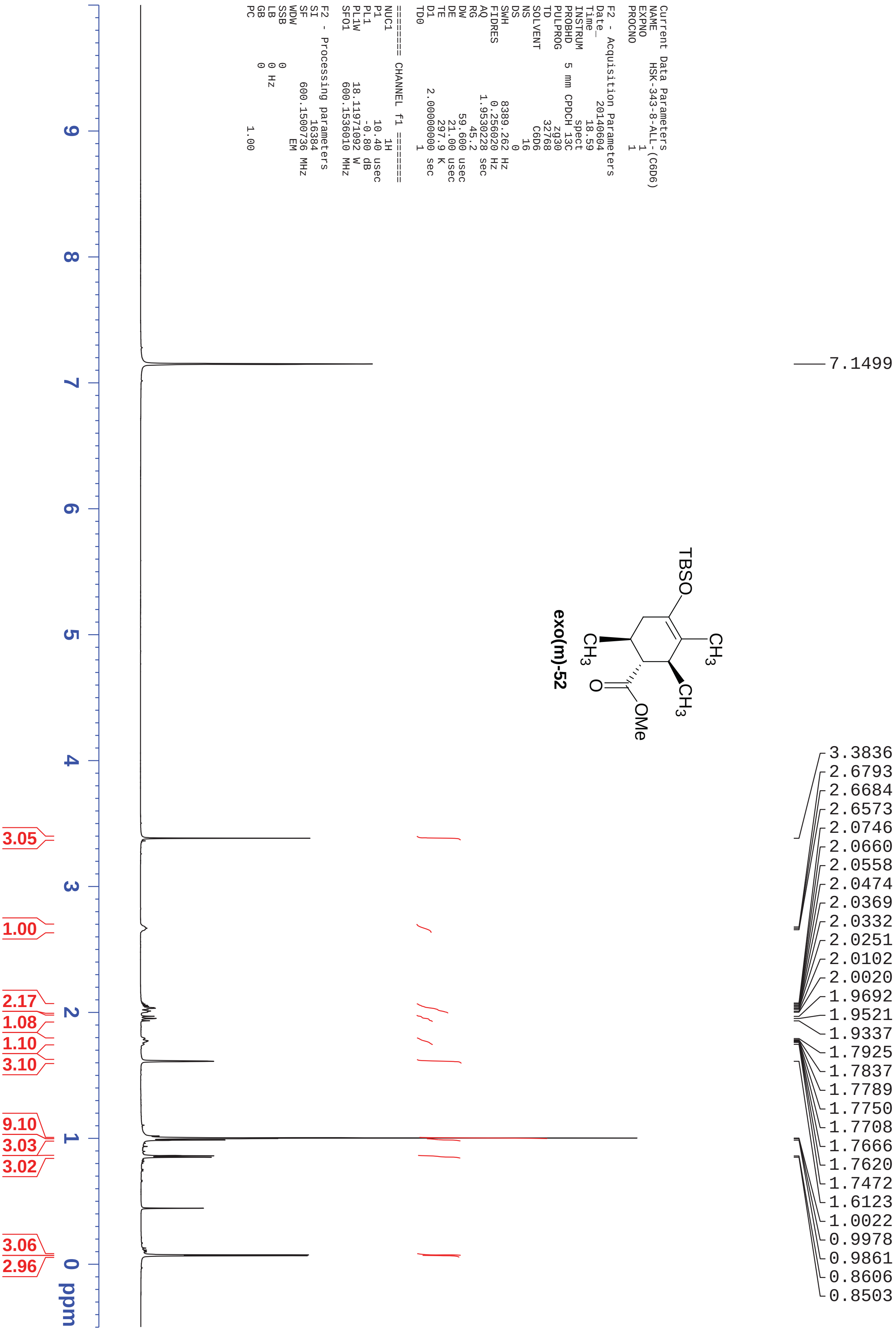

Supplementary Figure 186. ^{1}**H NMR spectrum of compound exo(m)-52.**

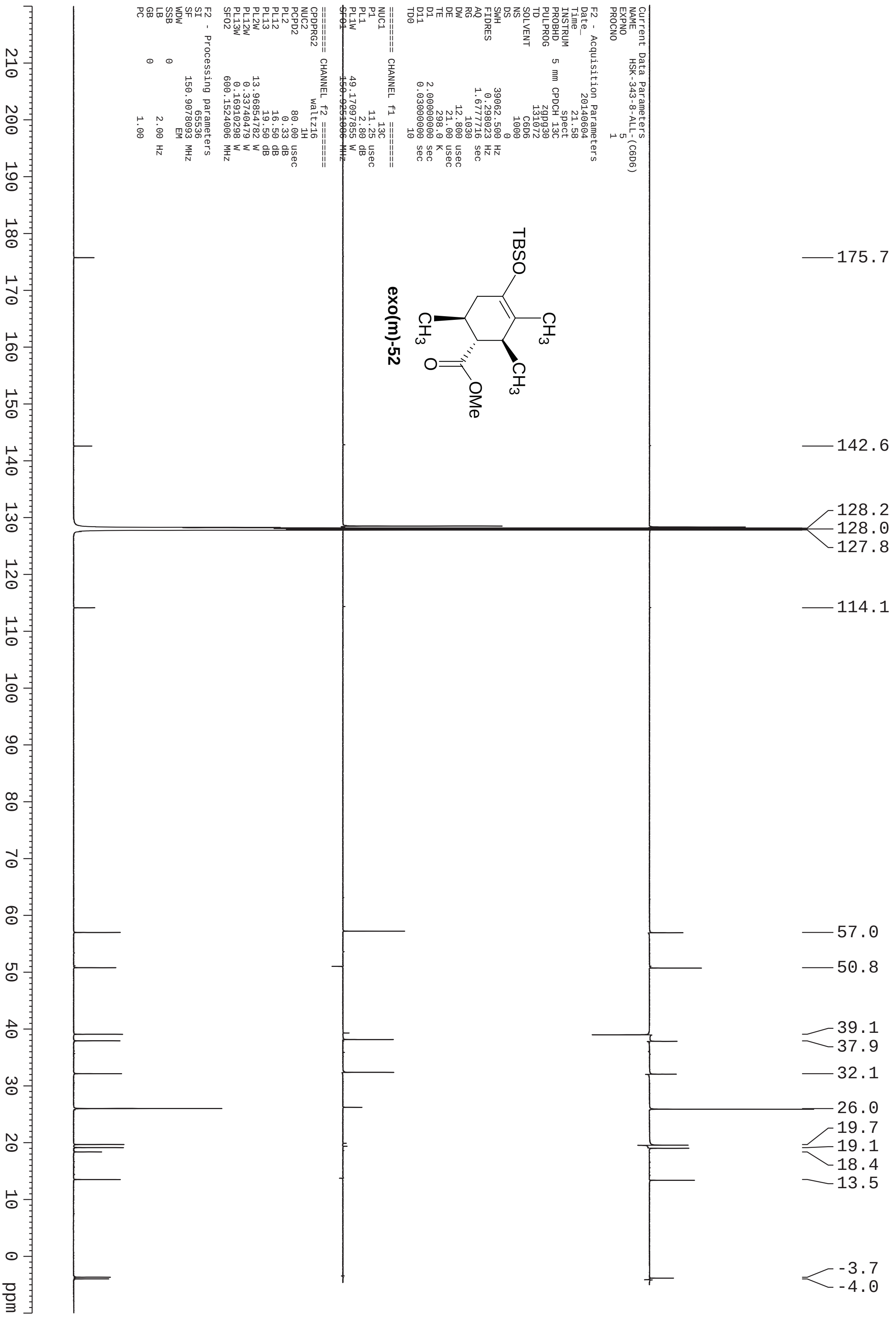

Supplementary Figure 187. ^{13}C and DEPT NMR spectra of compound exo(m)-52.

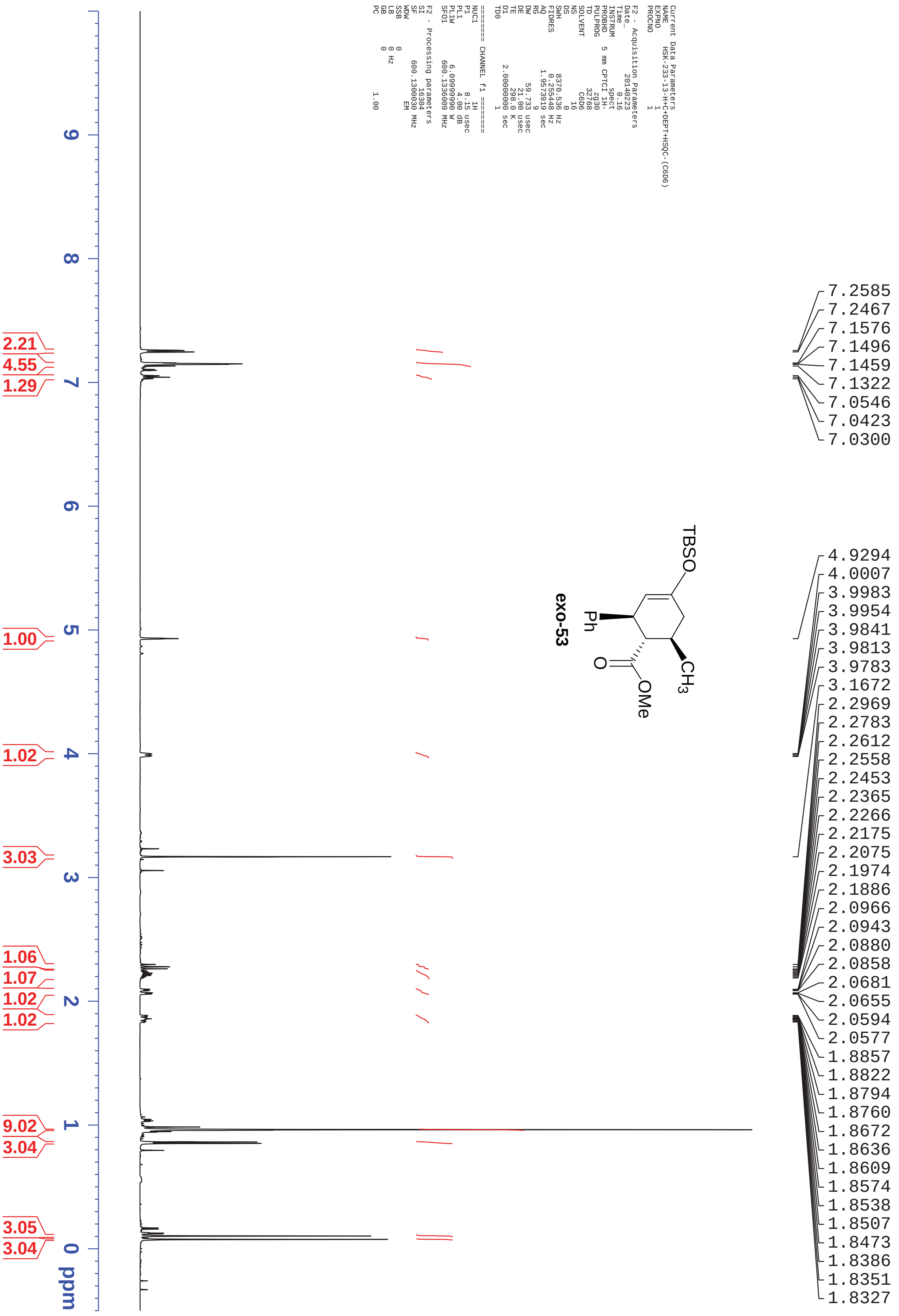

Supplementary Figure 188. ^{1}H NMR spectrum of compound exo-53.

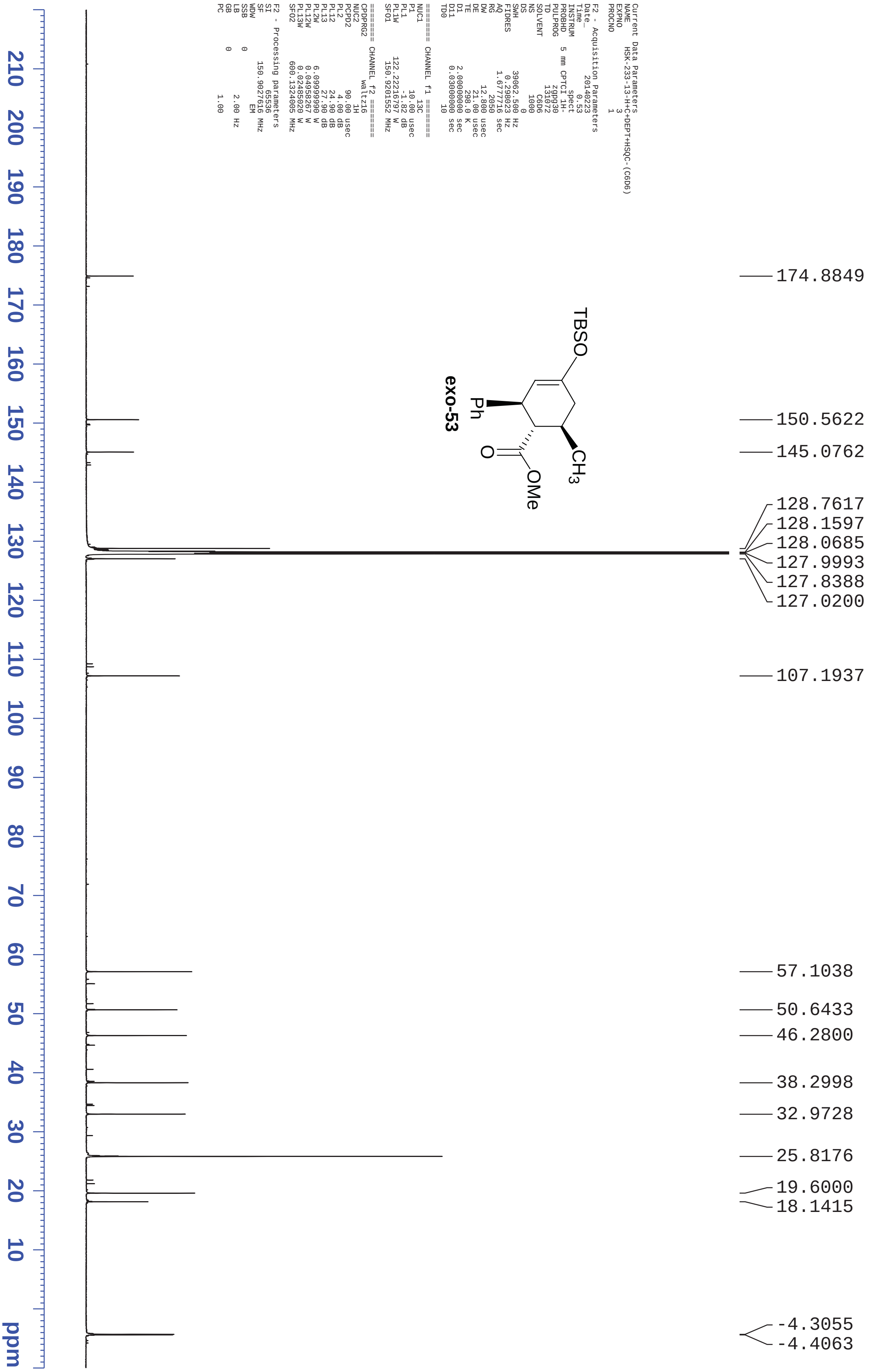

Supplementary Figure 189. ^{13}C NMR spectrum of compound exo-53.

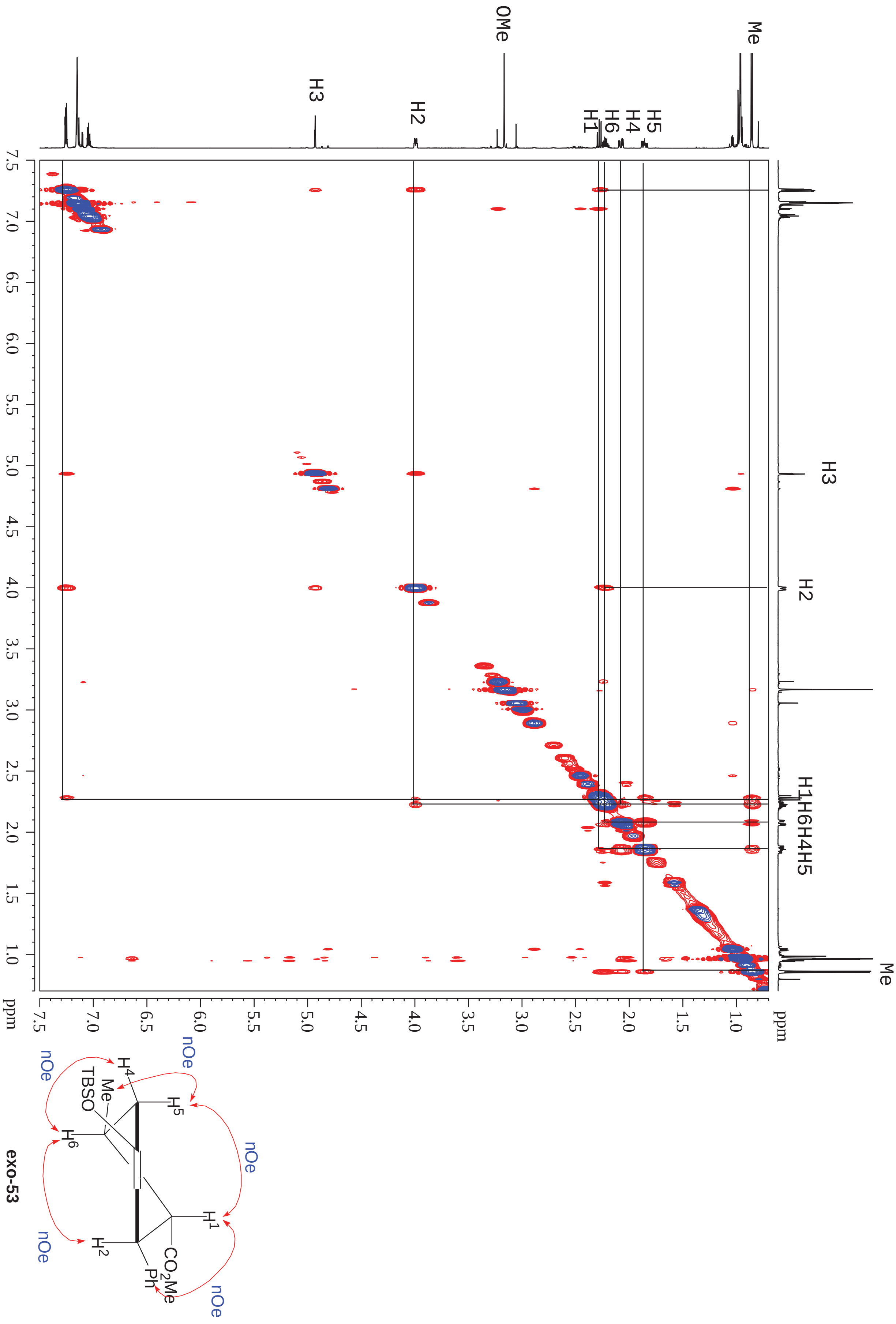

Supplementary Figure 190. NOESY NMR spectrum of compound exo-53.

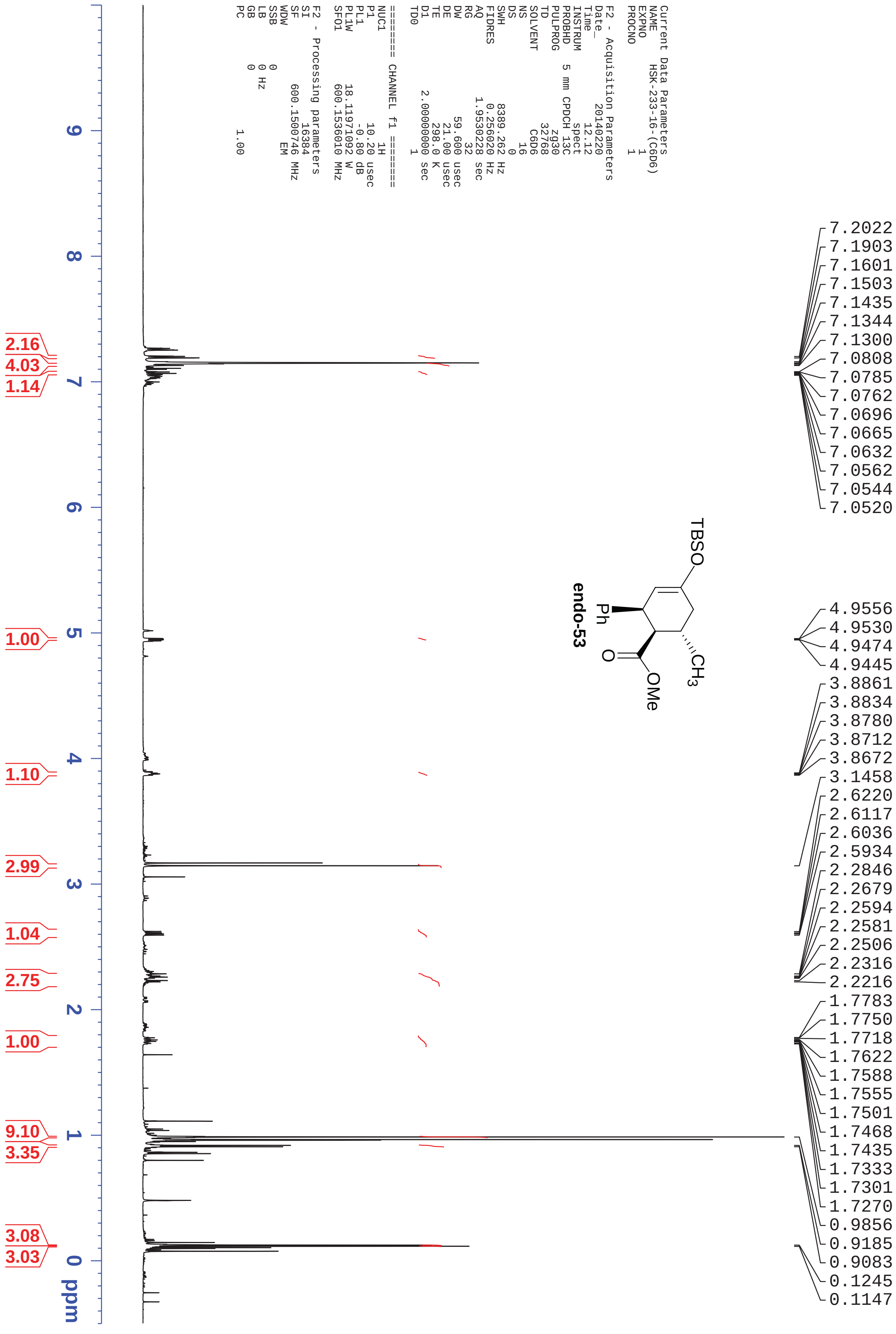

Supplementary Figure 191. ^{1}H NMR spectrum of compound endo-53.

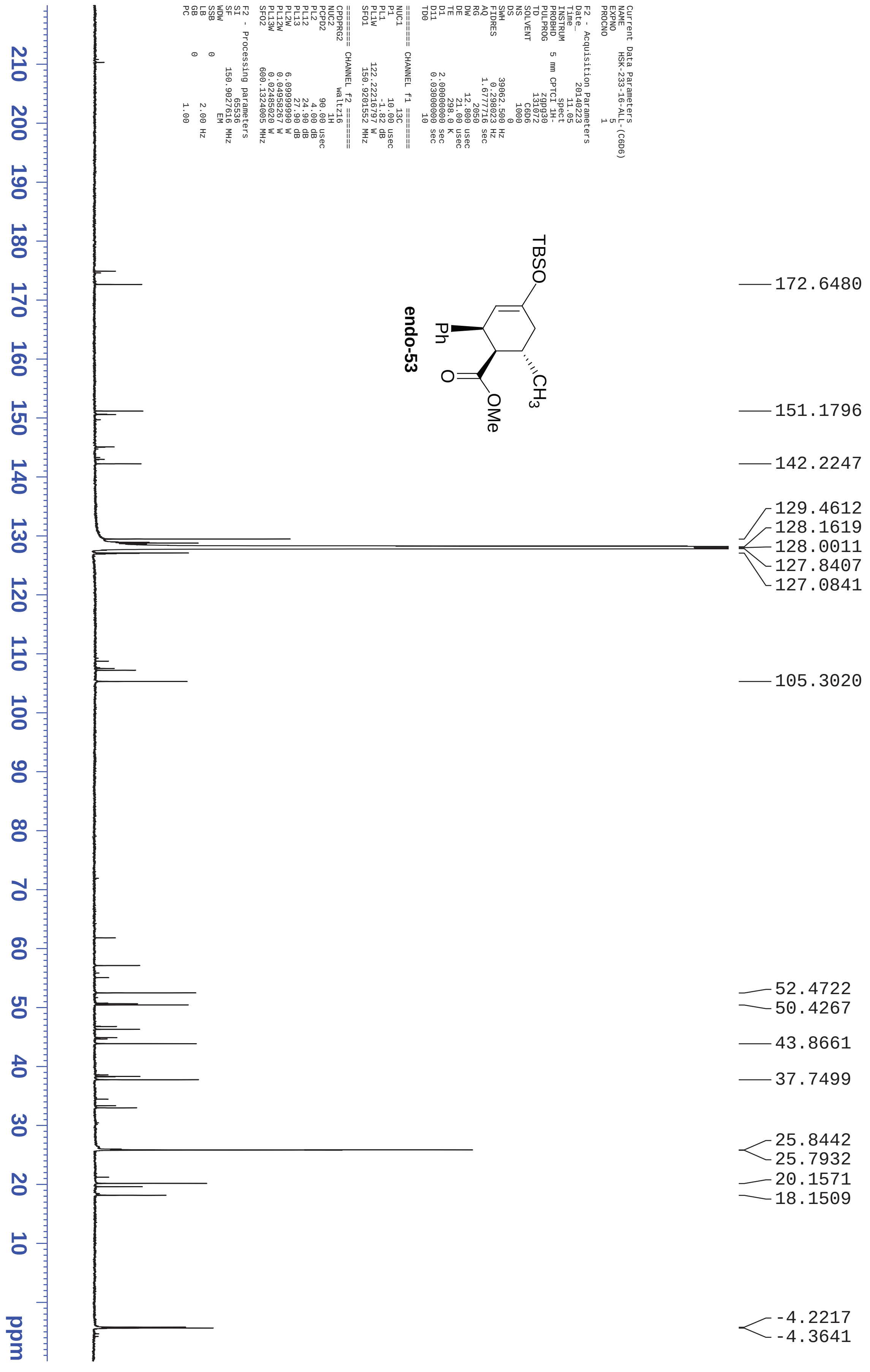

Supplementary Figure 192. ^{13}C NMR spectrum of compound endo-53.

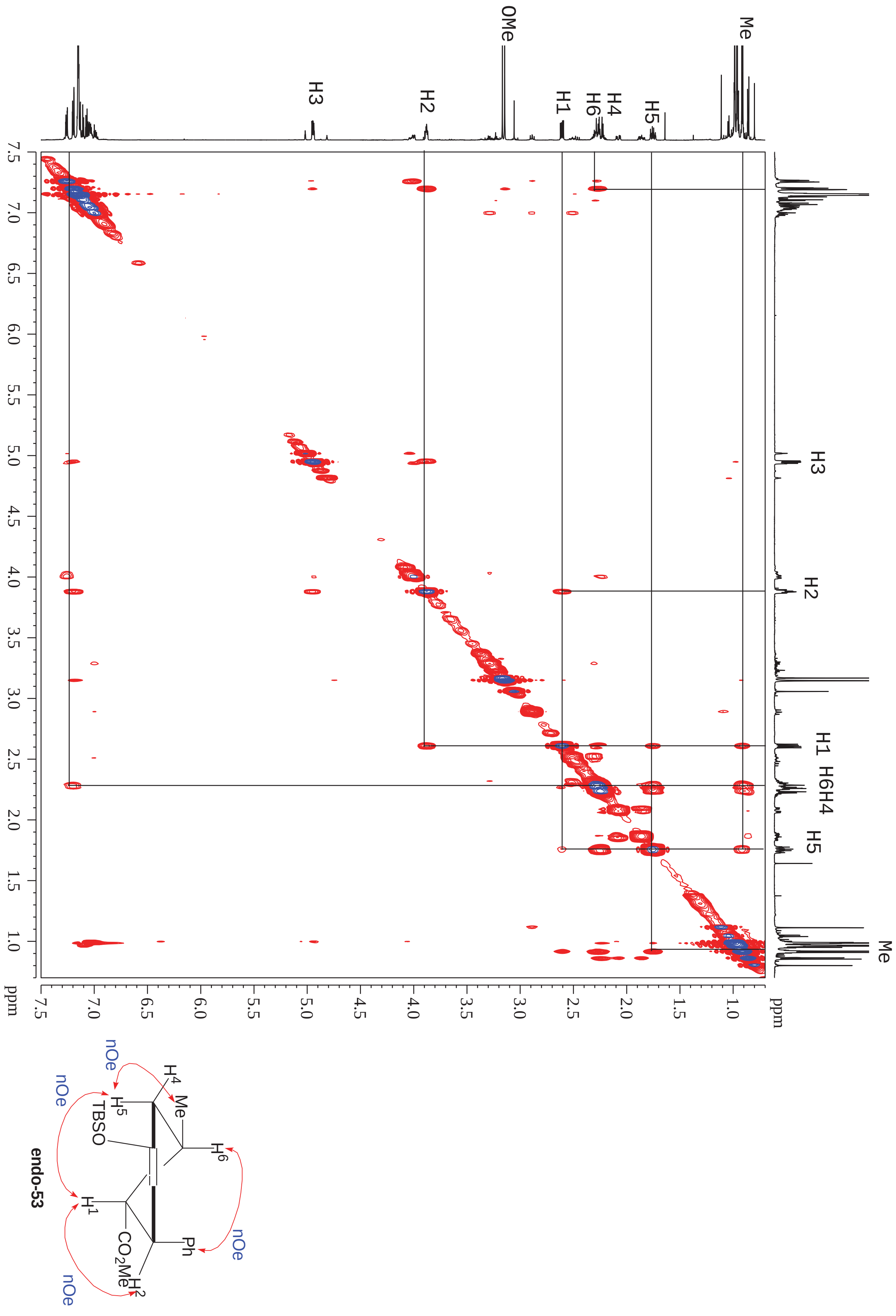

Supplementary Figure 193. NOESY NMR spectrum of compound endo-53.

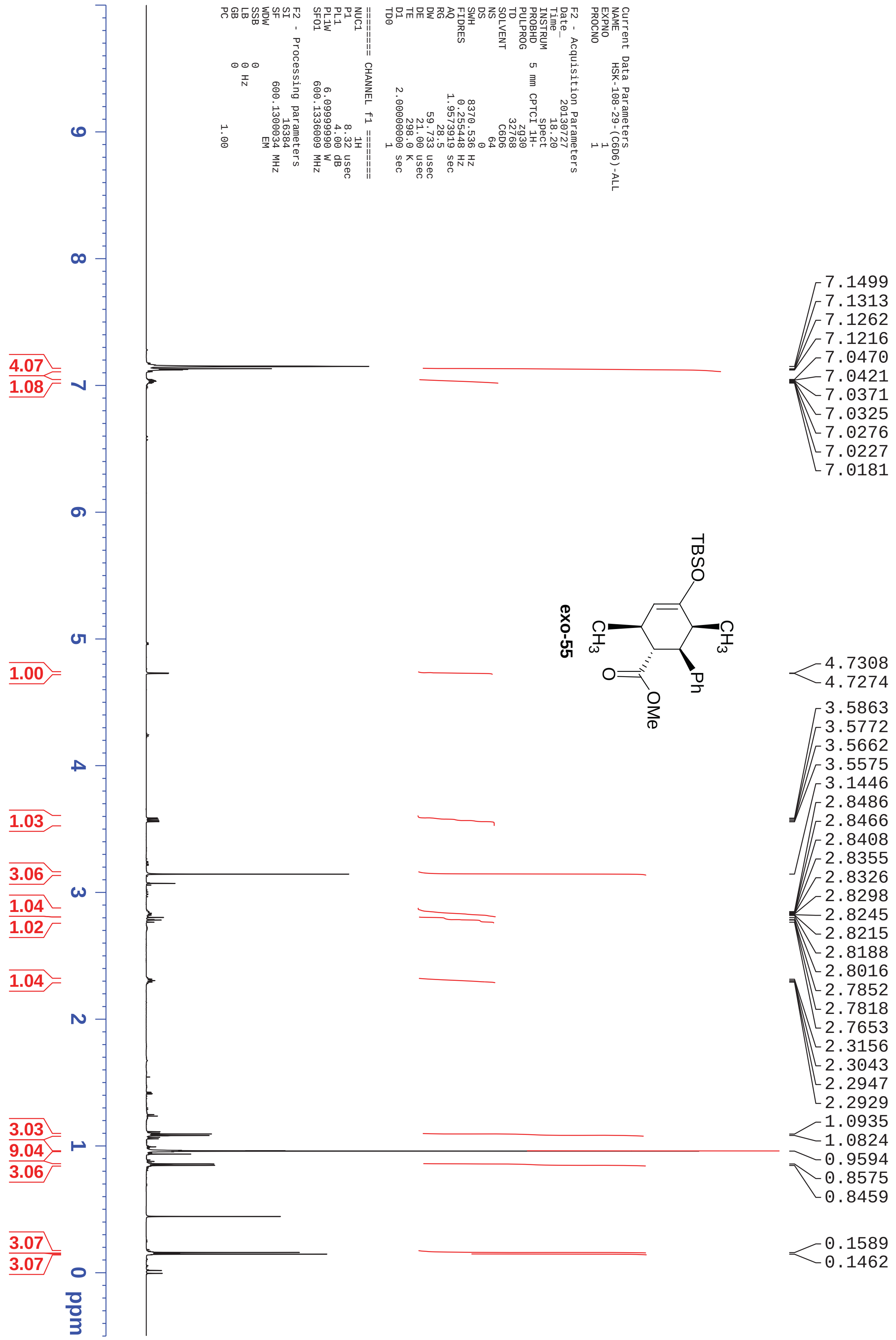

Supplementary Figure 194. ^{1}**H NMR spectrum of compound exo-55.**

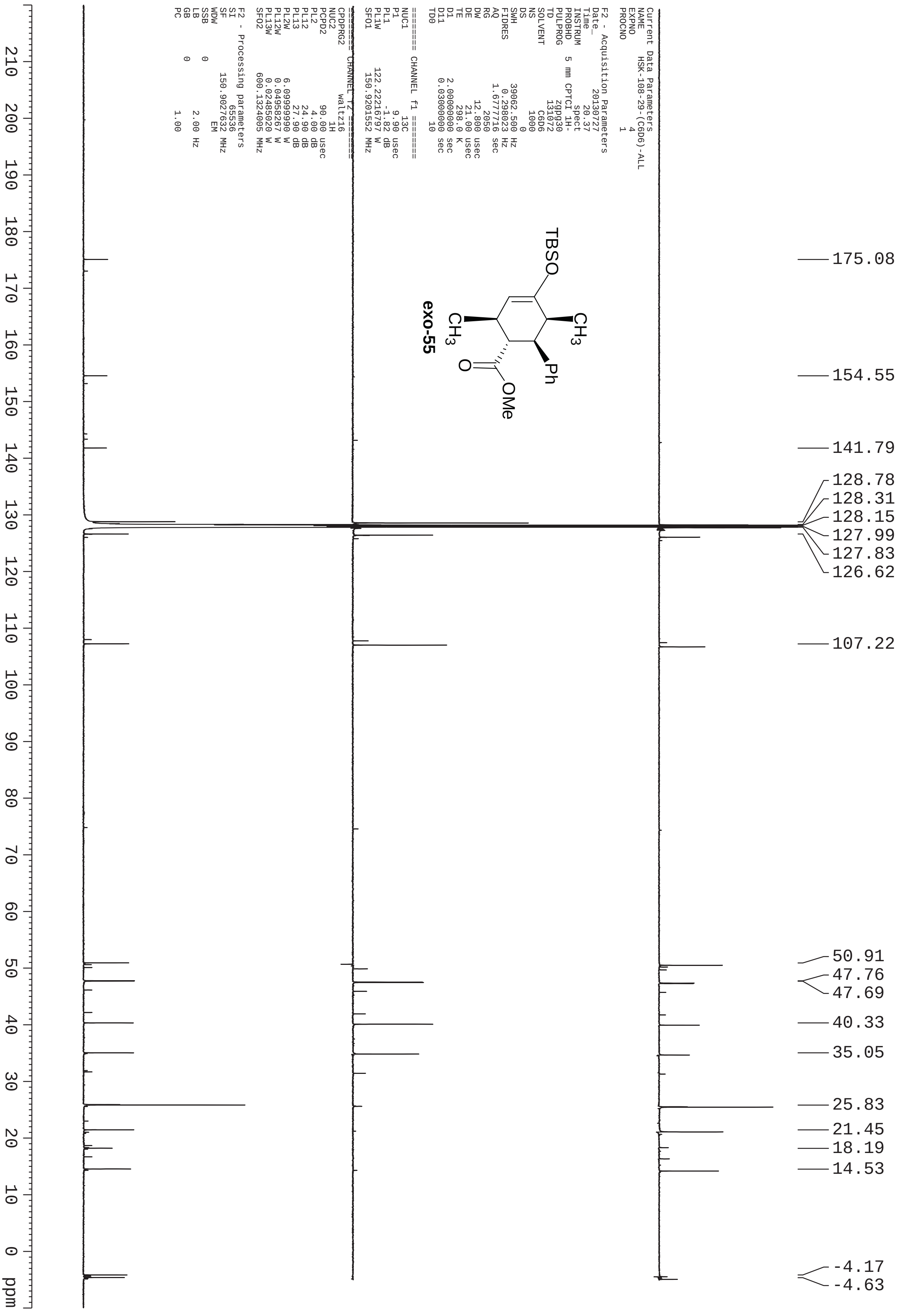

Supplementary Figure 195. ^{13}C and DEPT NMR spectra of compound exo-55.

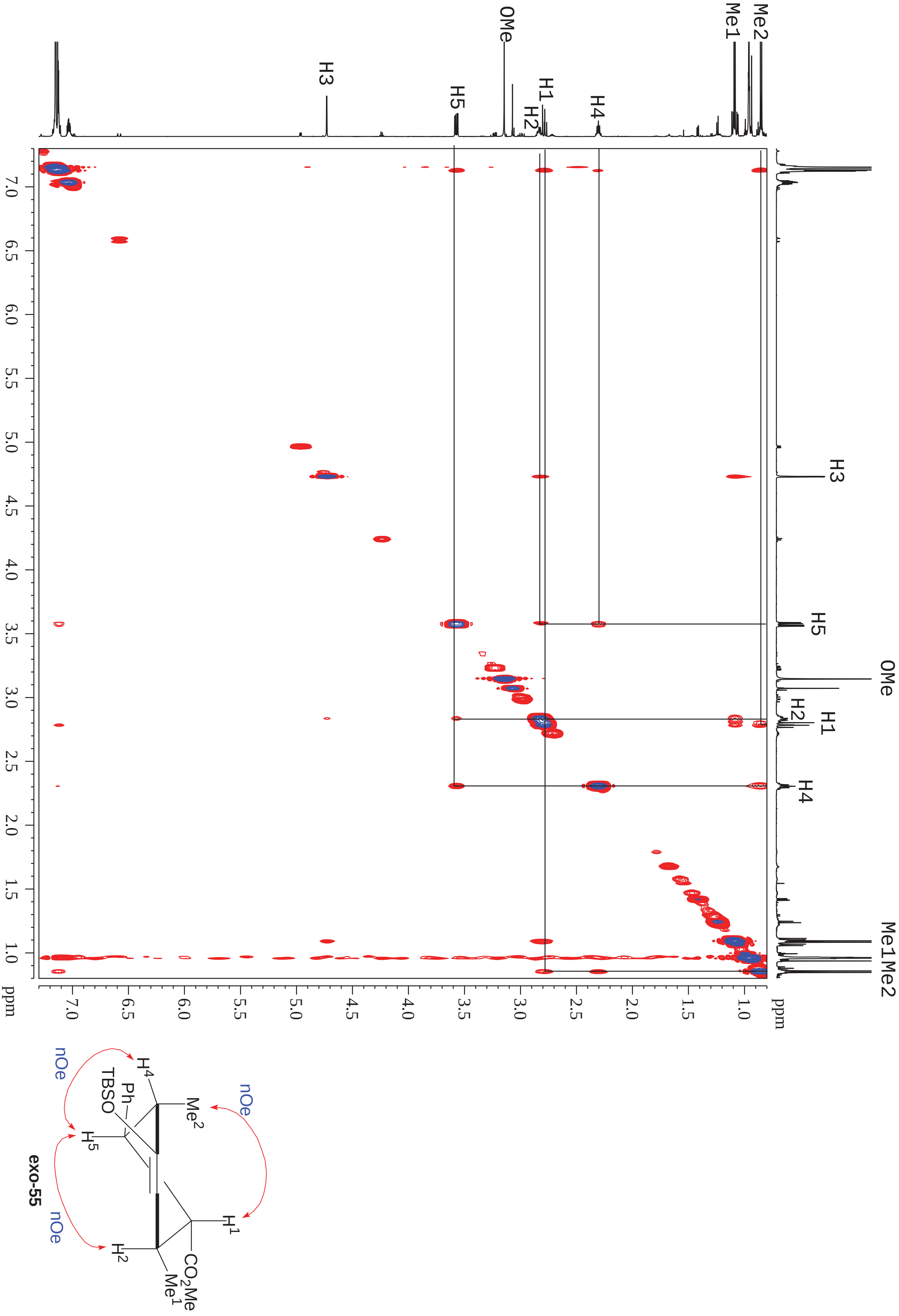

Supplementary Figure 196. NOESY NMR spectrum of compound exo-55.

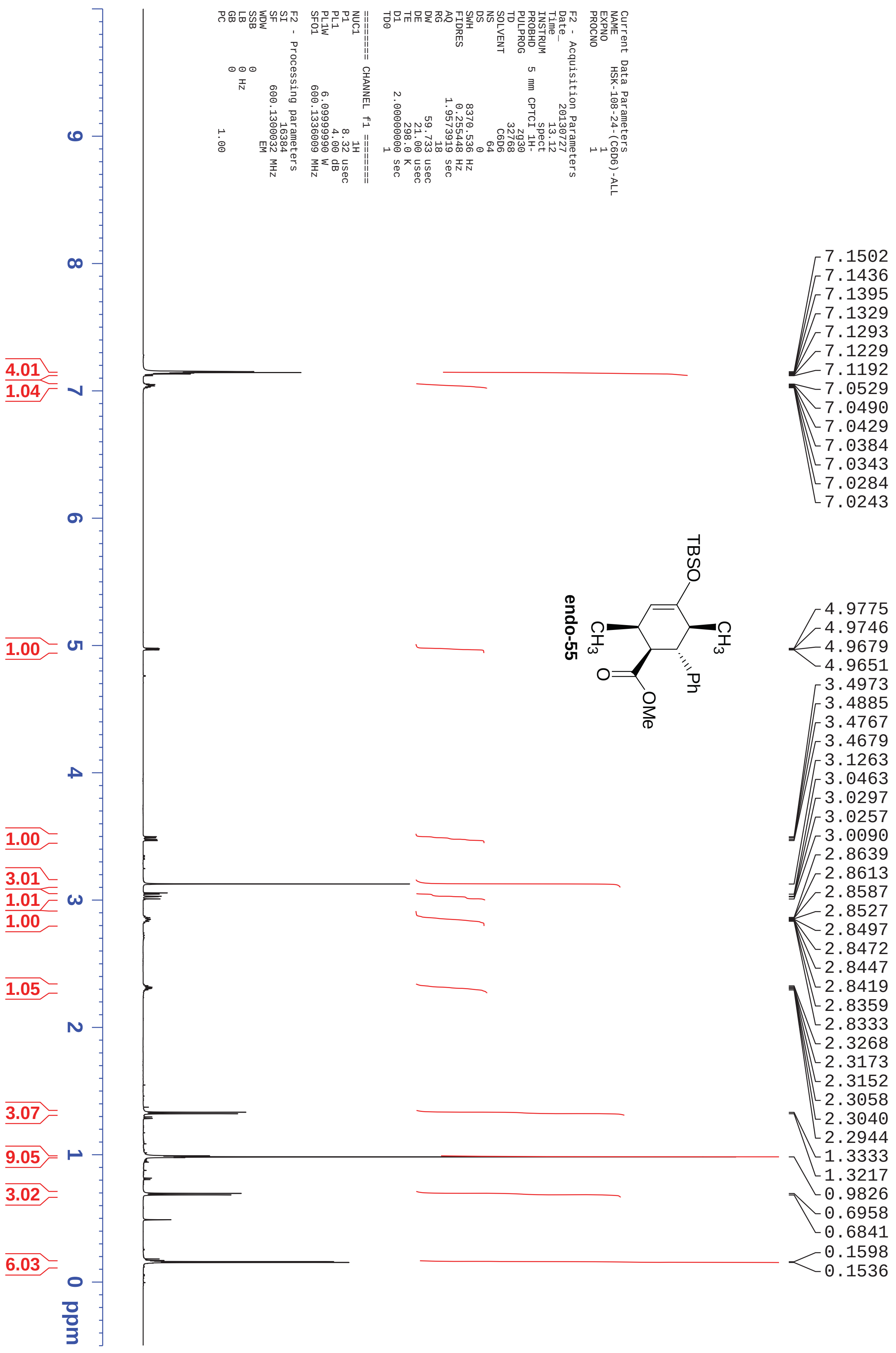

Supplementary Figure 197. ^{1}**H NMR spectrum of compound endo-55.**

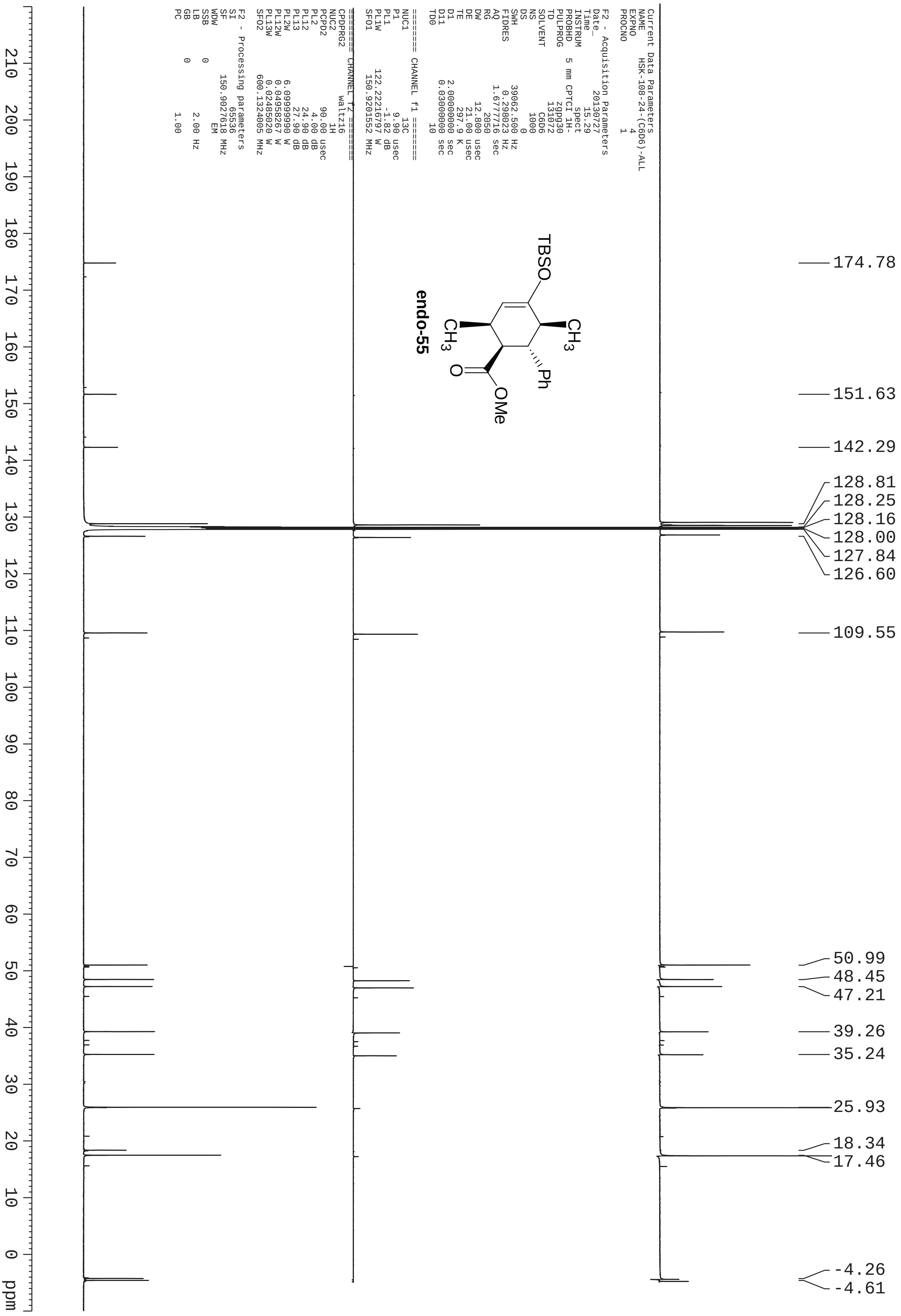

Supplementary Figure 198. ^{13}C and DEPT NMR spectra of compound endo-55.

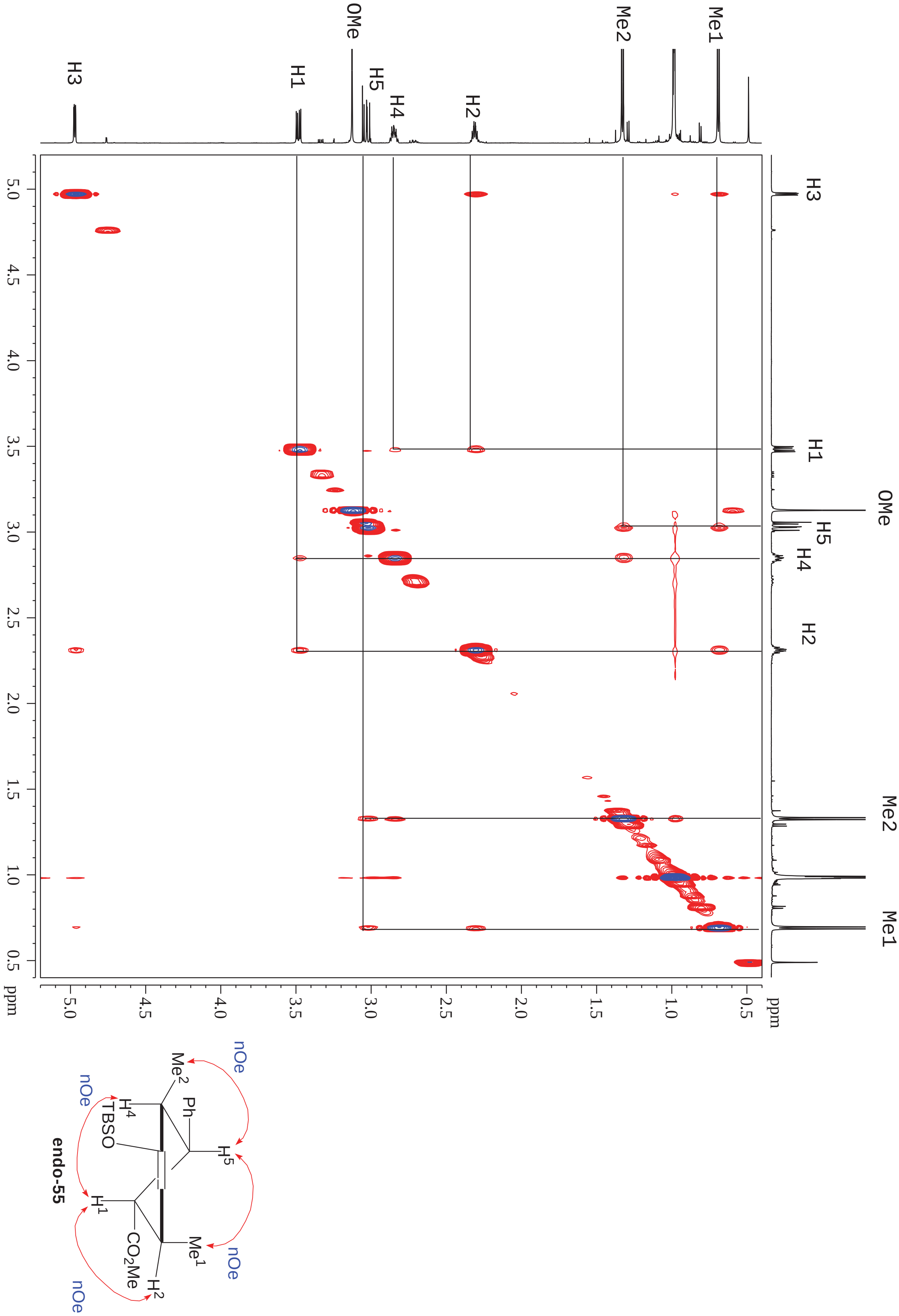

Supplementary Figure 199. NOESY NMR spectrum of compound endo-55.

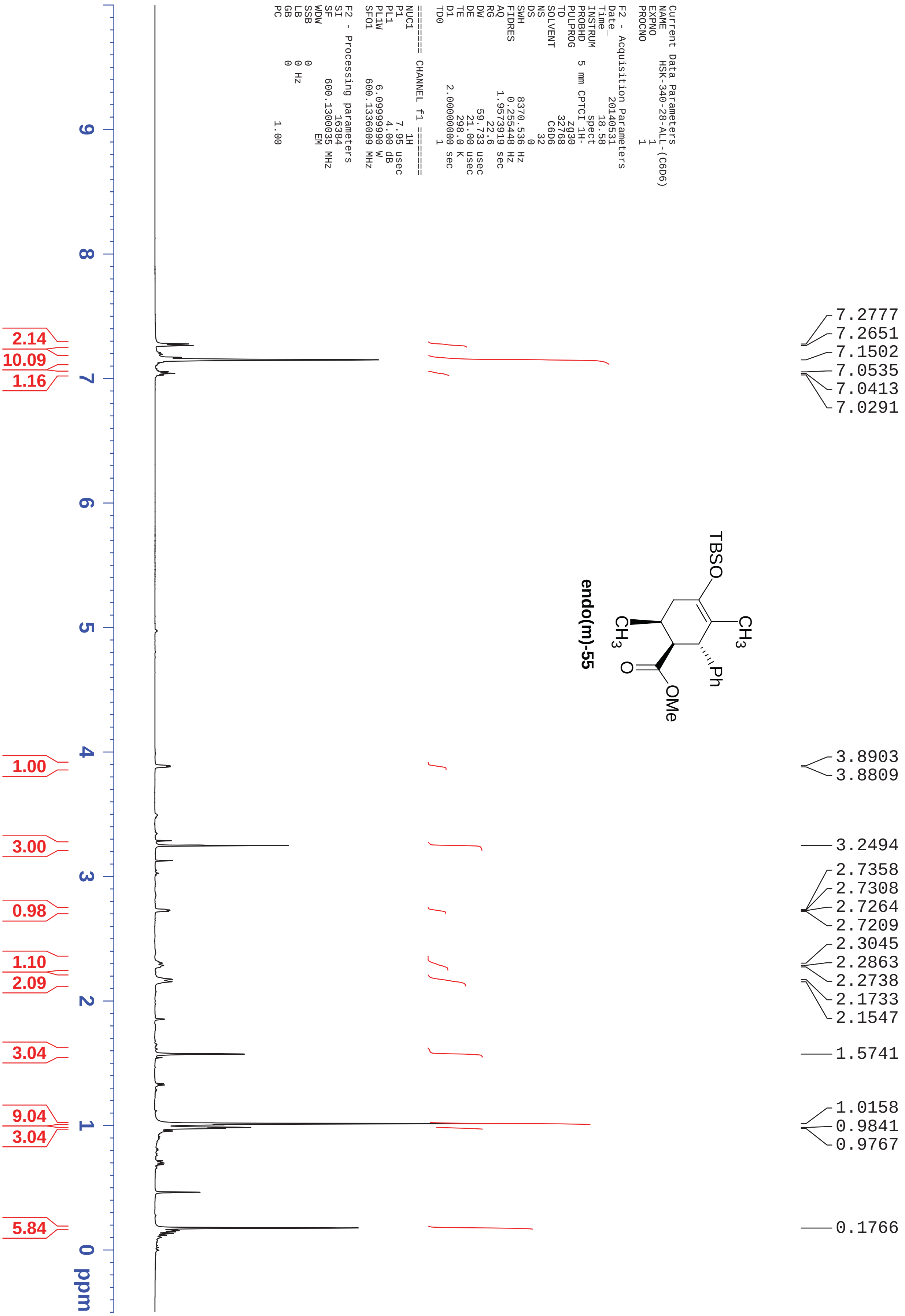

Supplementary Figure 200. ^{1}**H NMR spectrum of compound endo(m)-55.**

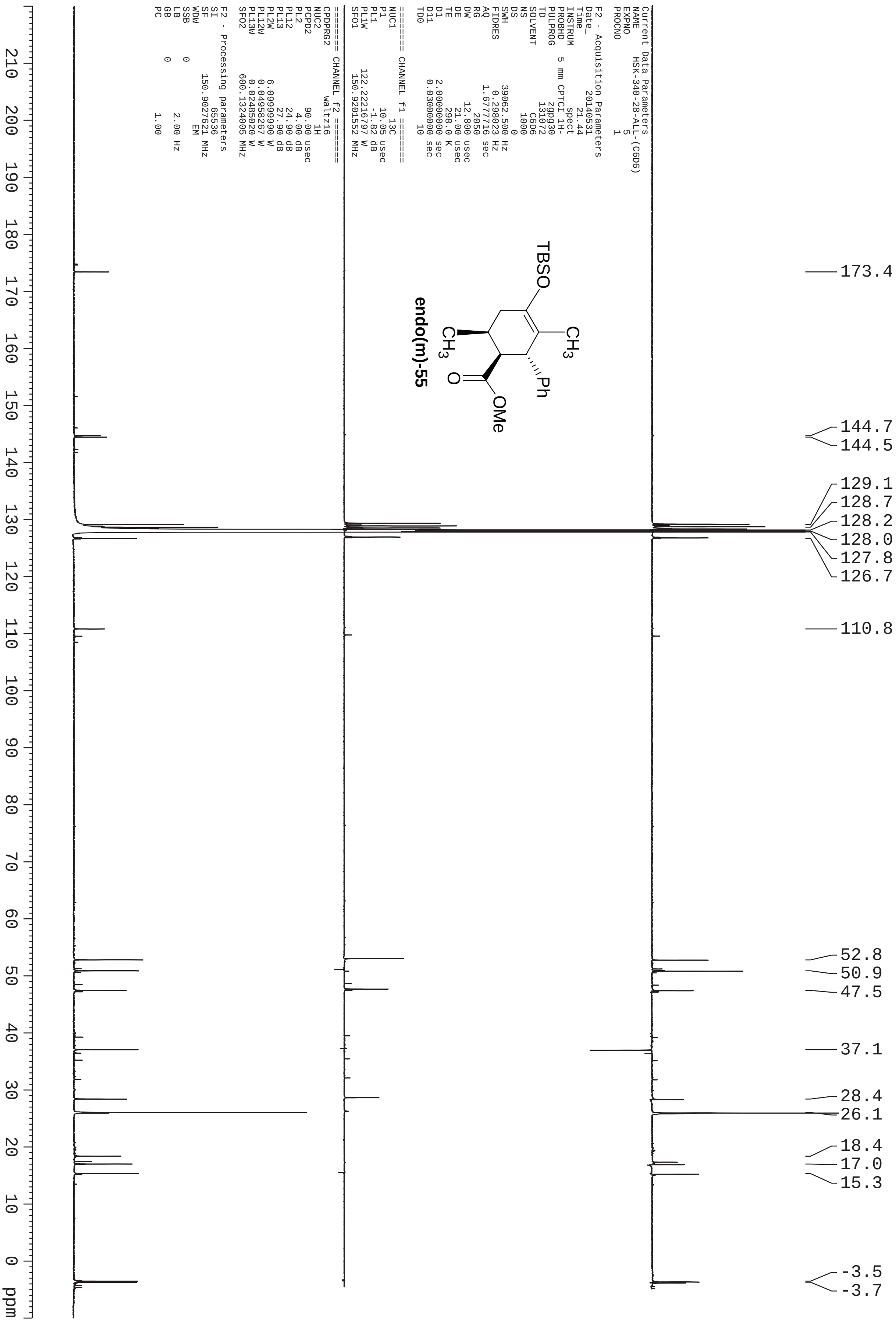

Supplementary Figure 201. ^{13}C and DEPT NMR spectra of compound endo(m)-55.

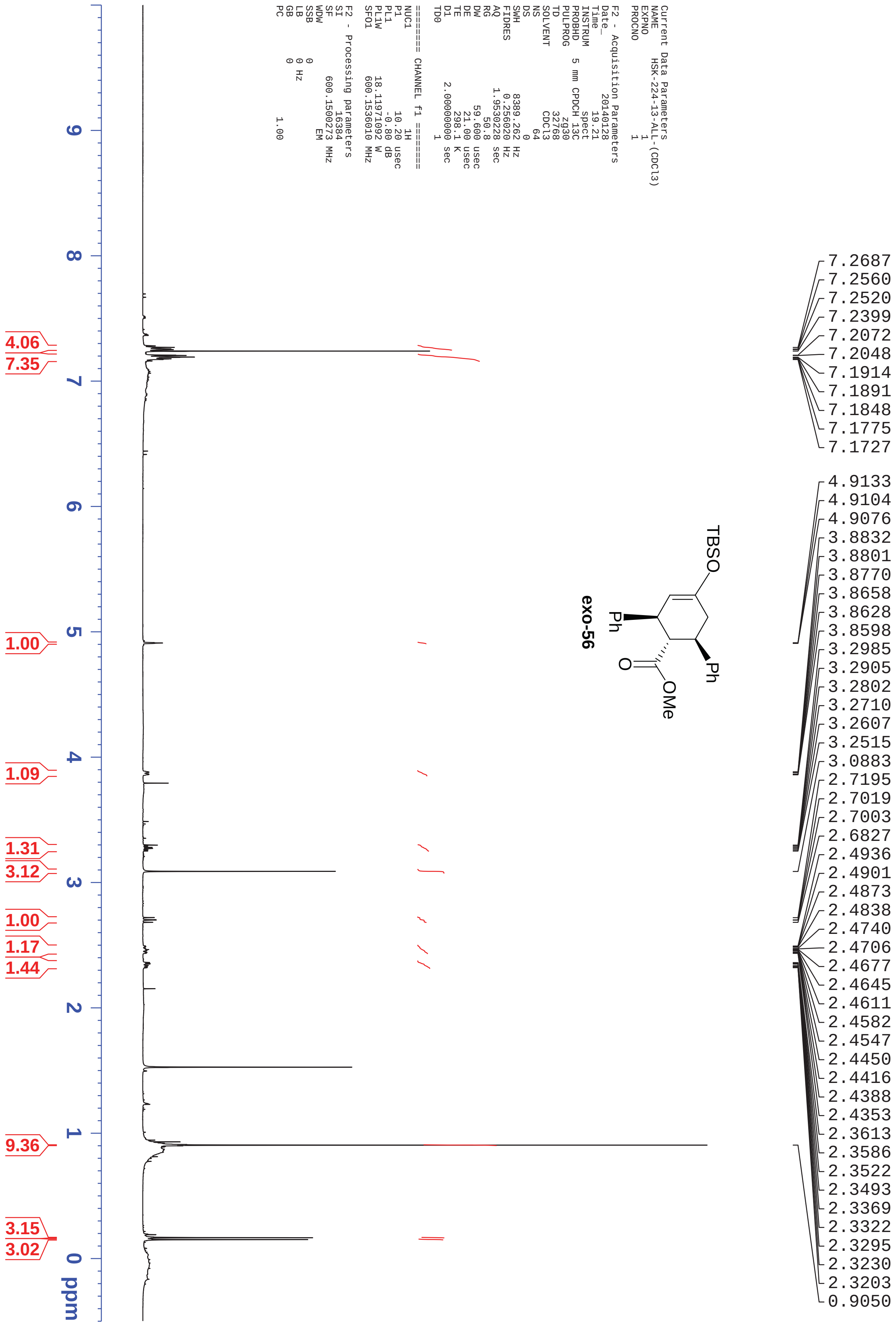

Supplementary Figure 202. ¹H NMR spectrum of compound exo-56.

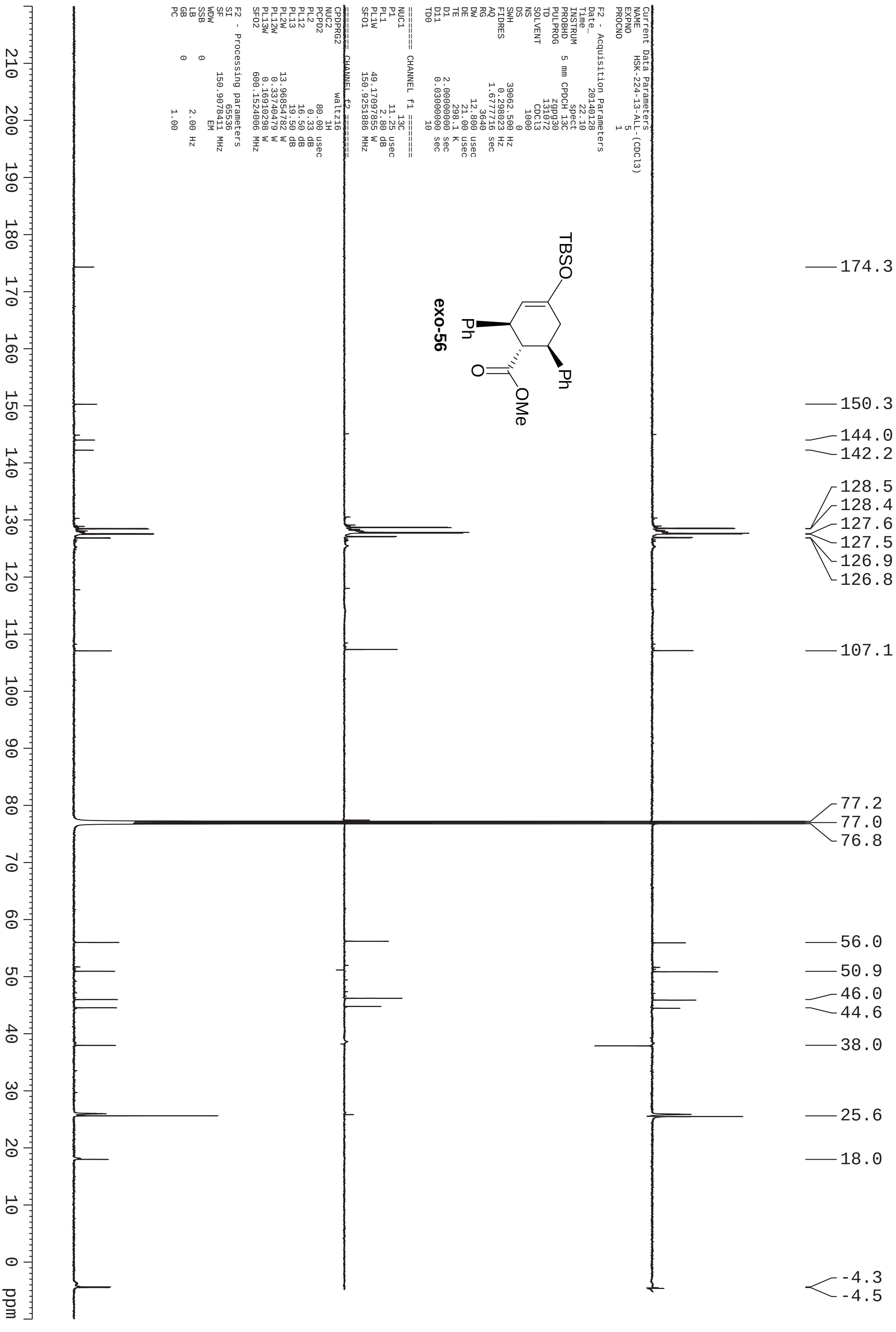

Supplementary Figure 203. ^{13}C and DEPT NMR spectra of compound exo-56.

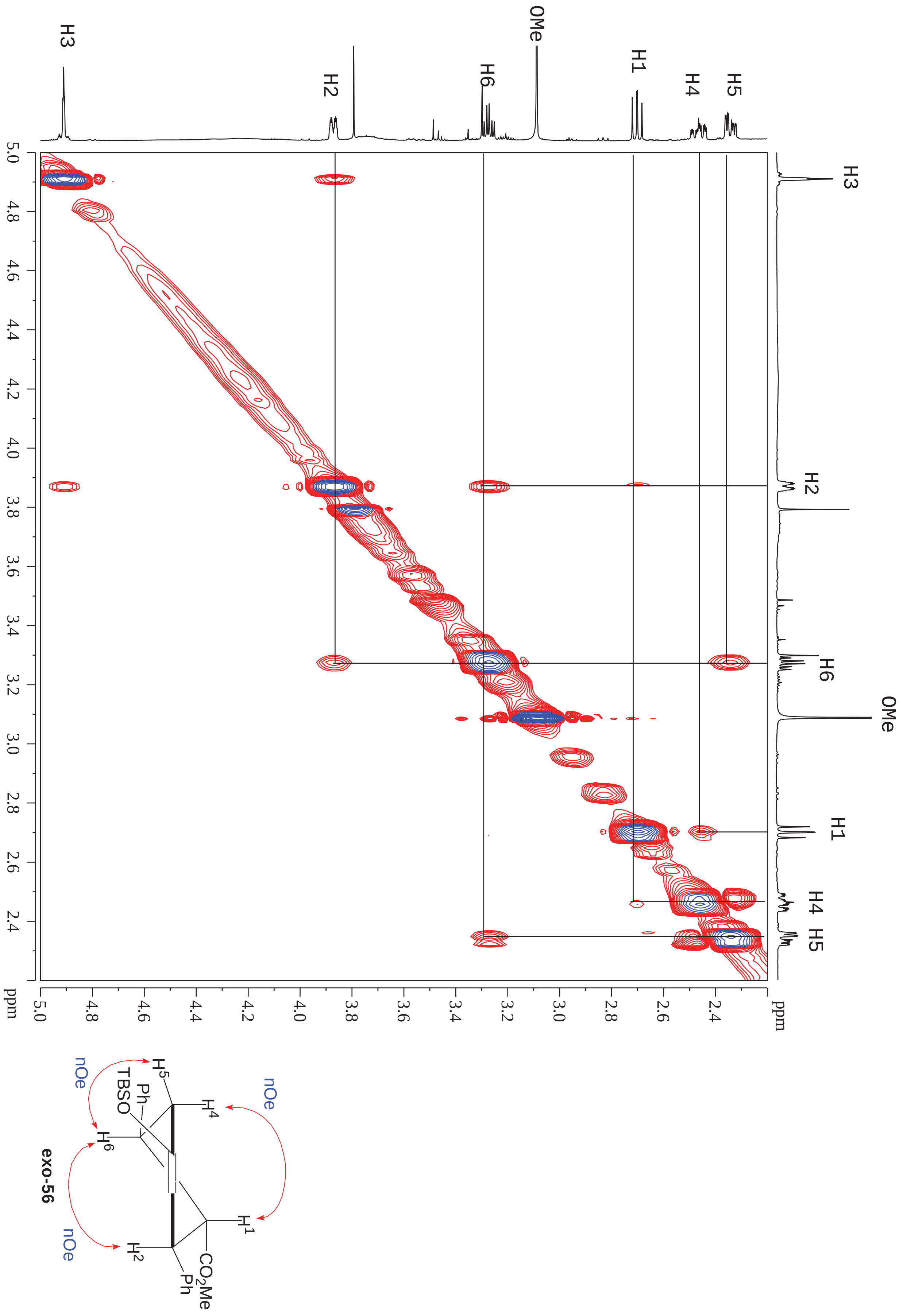

Supplementary Figure 204. NOESY NMR spectrum of compound exo-56.

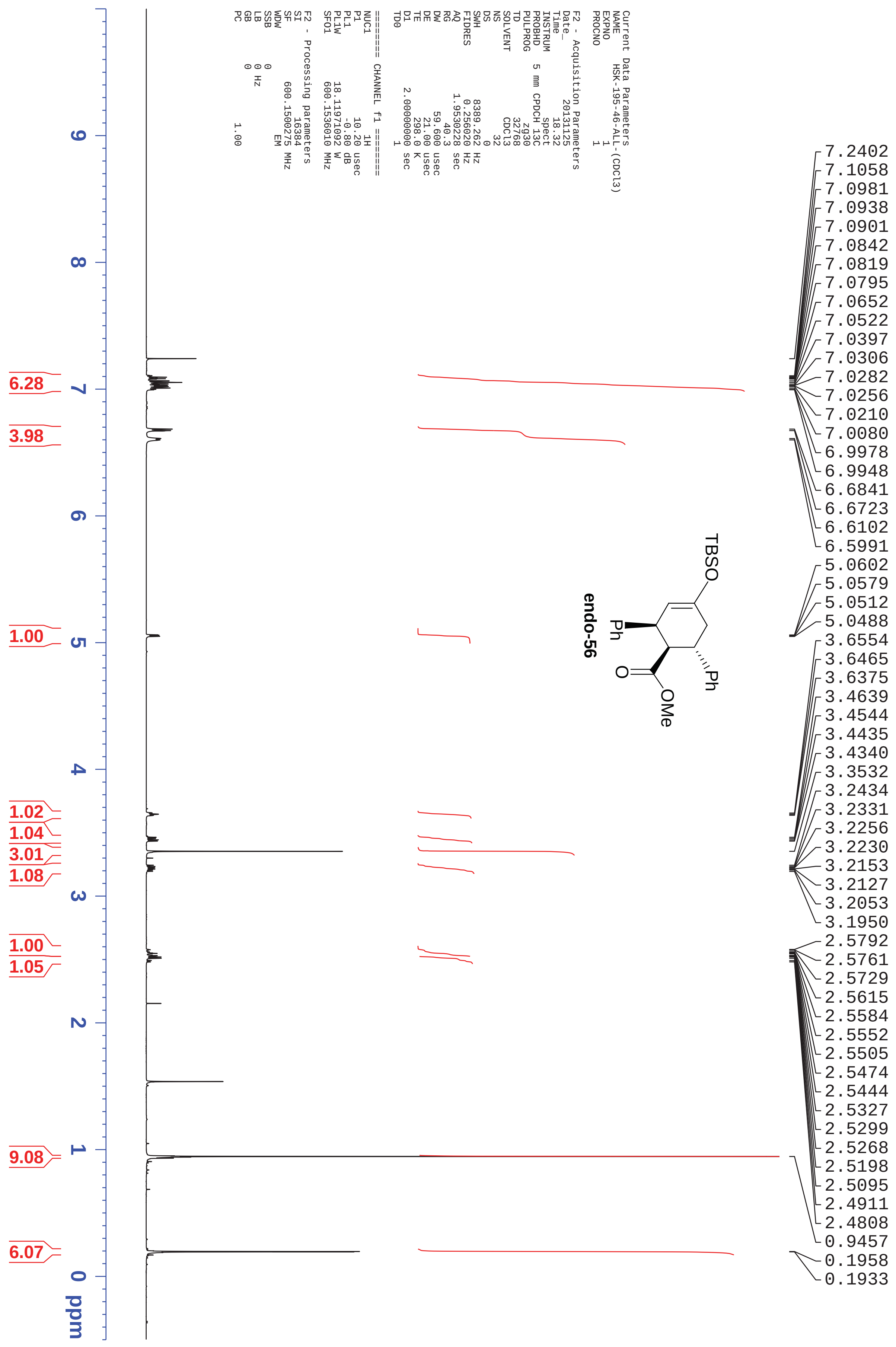

Supplementary Figure 205. ^{1}**H NMR spectrum of compound endo-56.**

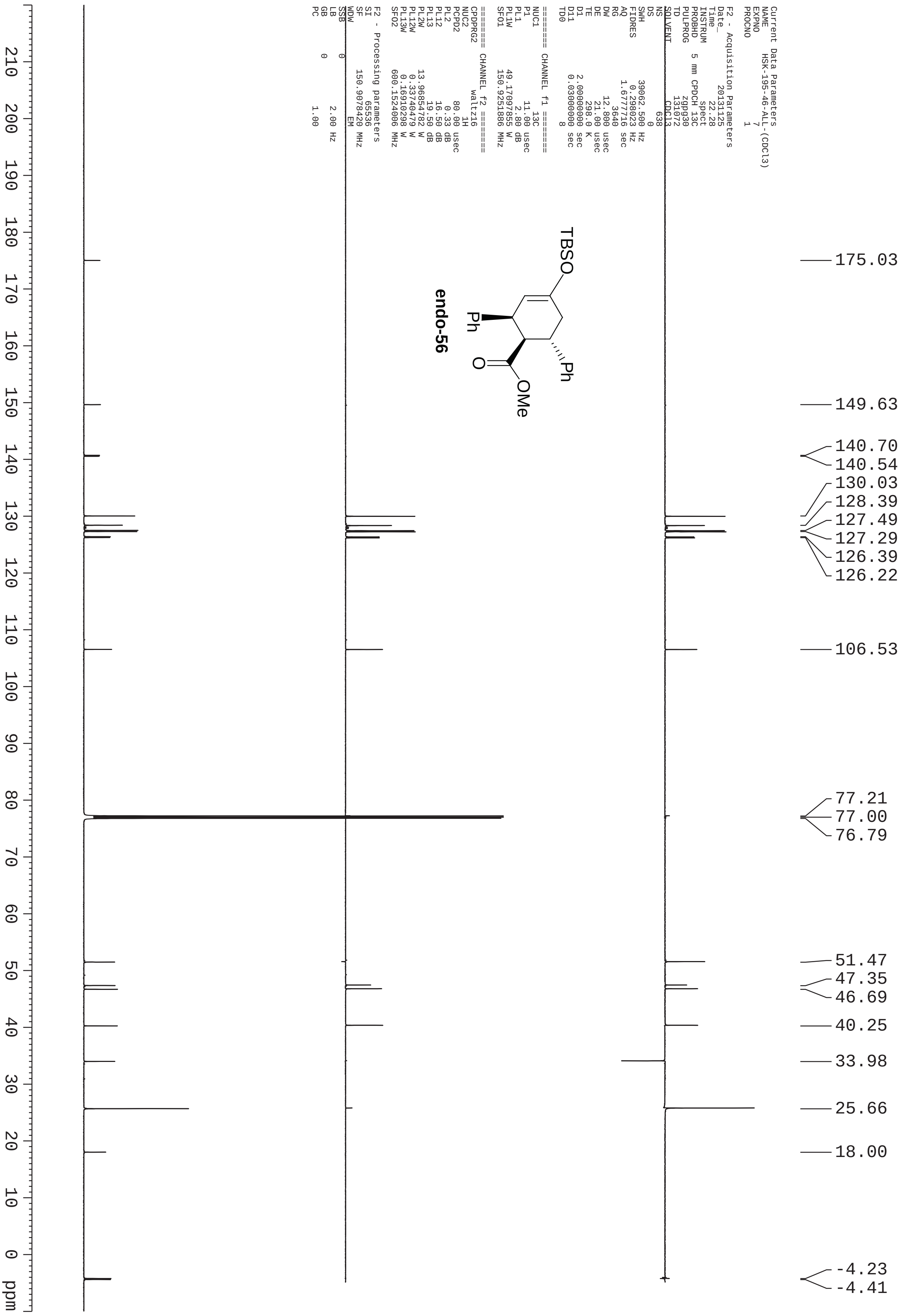

Supplementary Figure 206. ^{13}C and DEPT NMR spectra of compound endo-56.

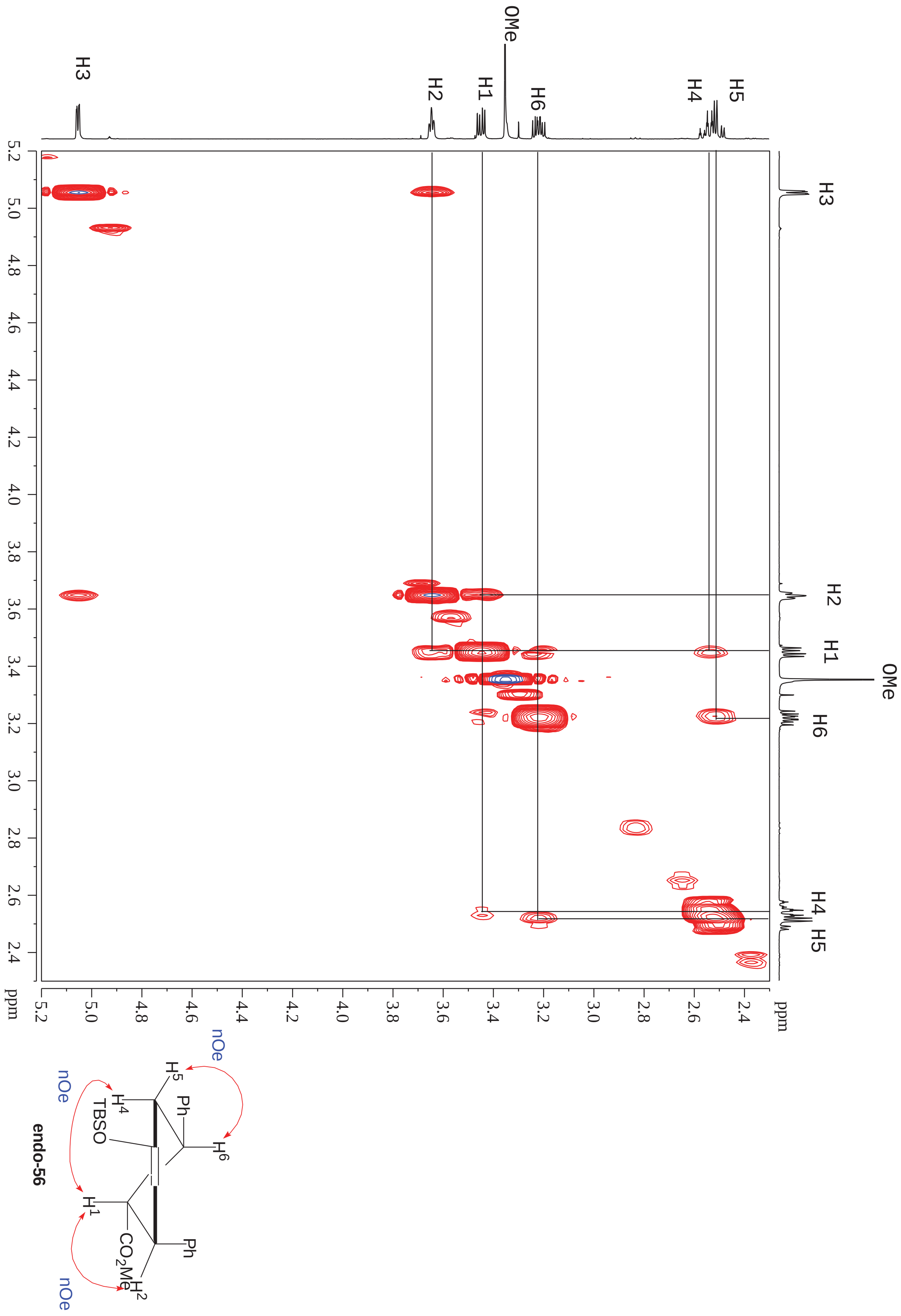

Supplementary Figure 207. NOESY NMR spectrum of compound endo-56.

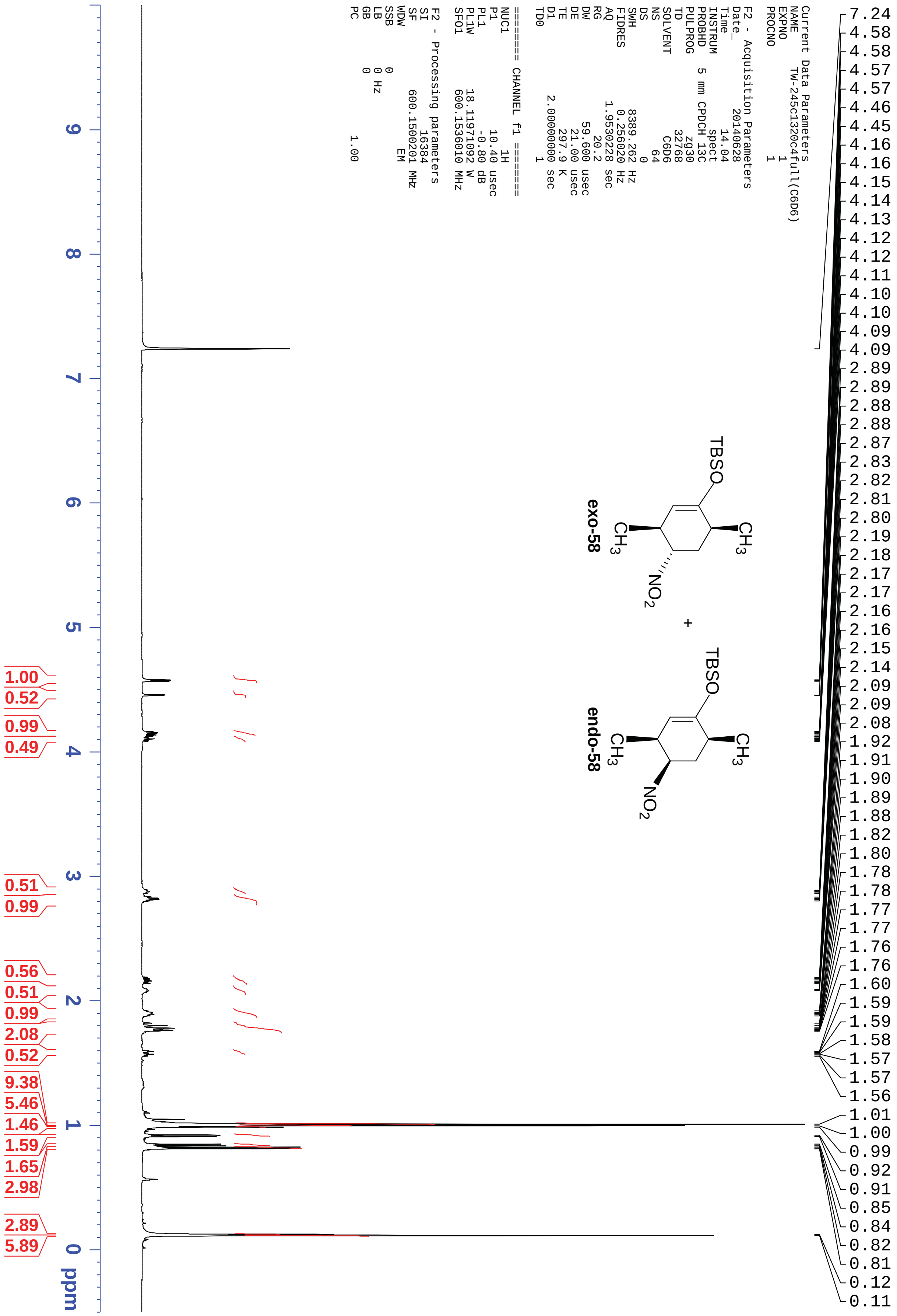

Supplementary Figure 208. ¹H NMR spectrum of mixed compound endo/exo-58.

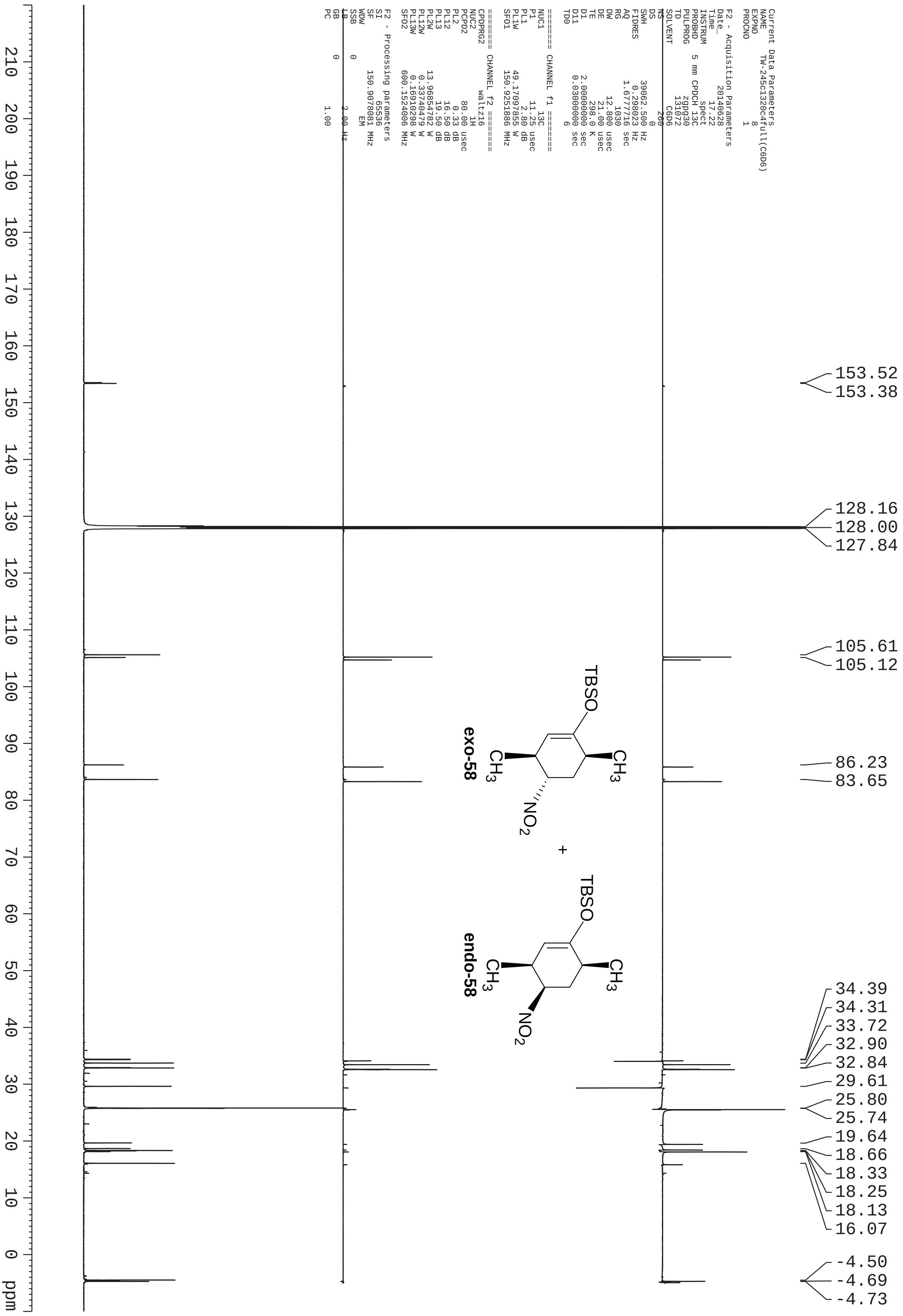

Supplementary Figure 209. ^{13}C **and DEPT NMR spectra of mixed compound endo/exo-58.**

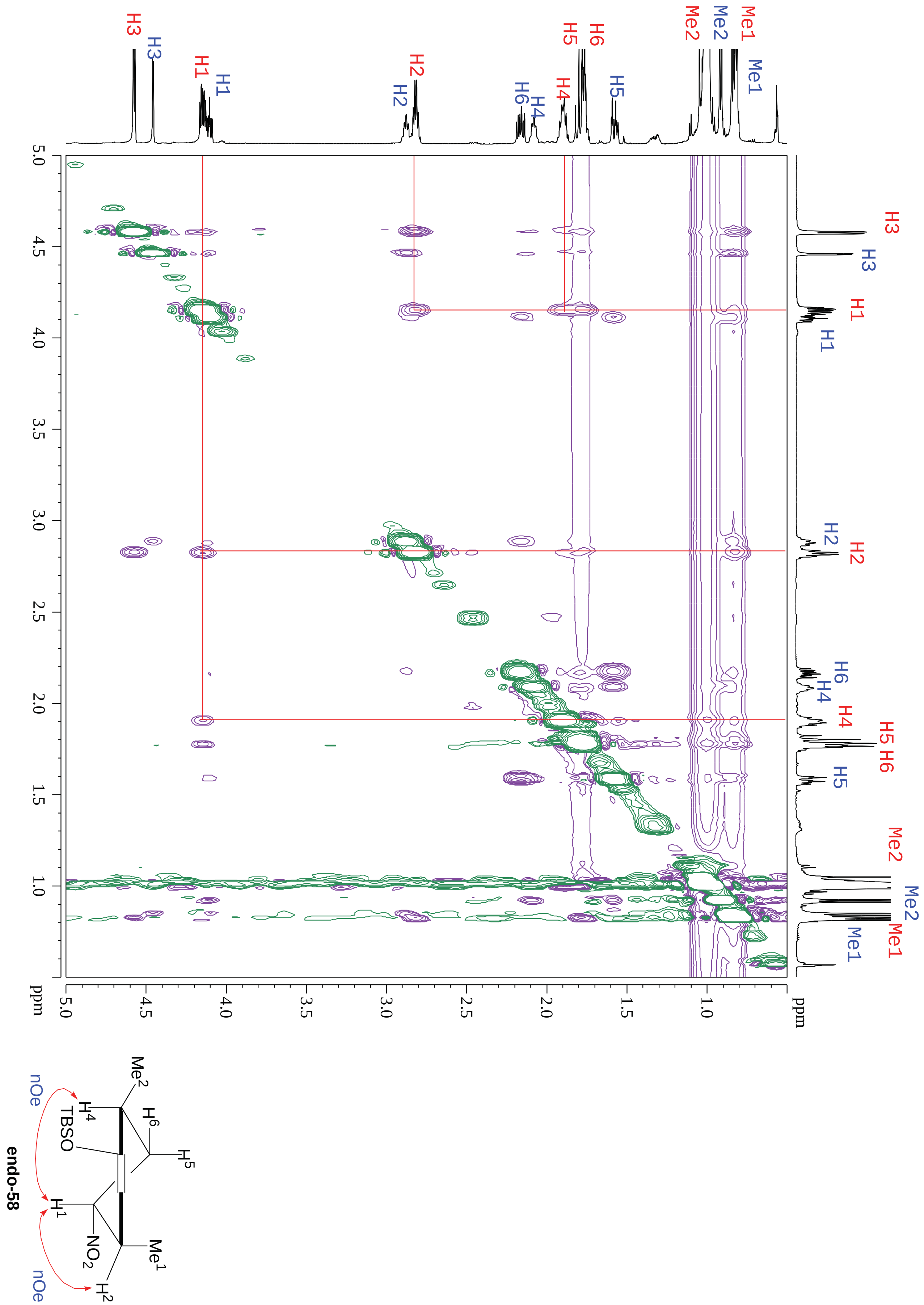

Supplementary Figure 210. NOESY NMR spectrum of mixed compound endo/exo-58 highlighting the correlation for endo-58.

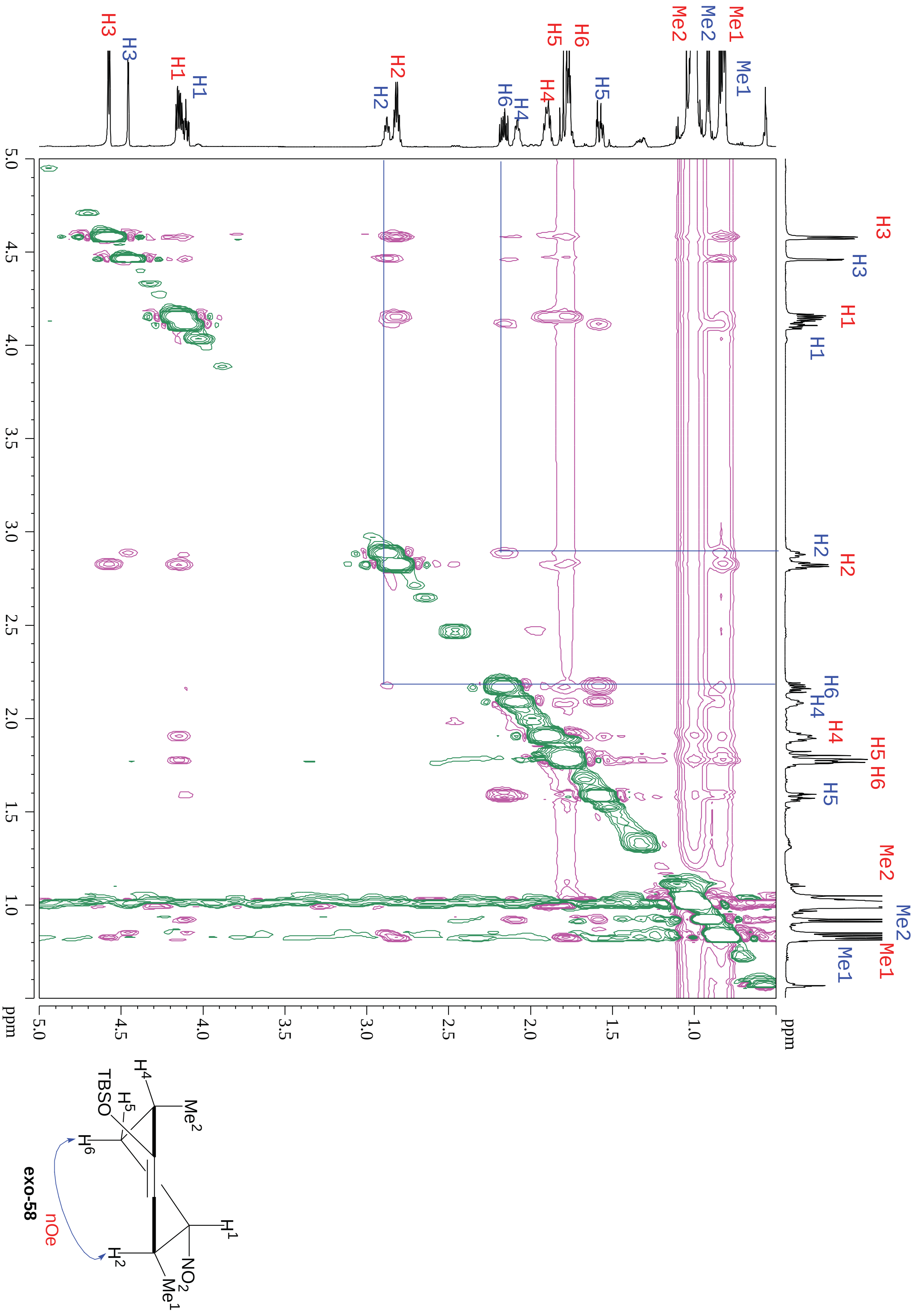

Supplementary Figure 211. NOESY NMR spectrum of mixed compound endo/exo-58 highlighting the correlation for exo-58.

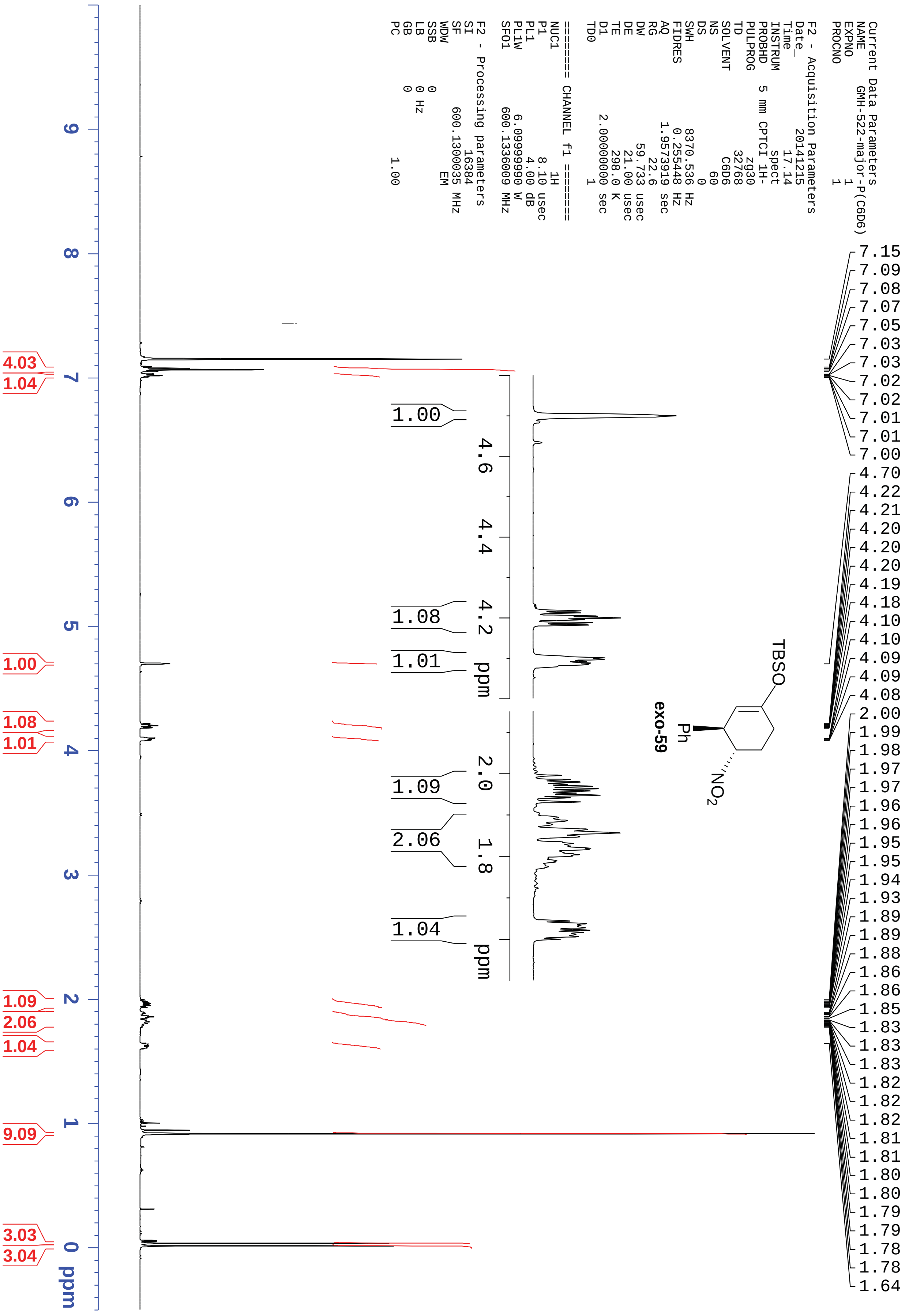

Supplementary Figure 212. **^{1}H NMR spectrum of compound exo-59.**

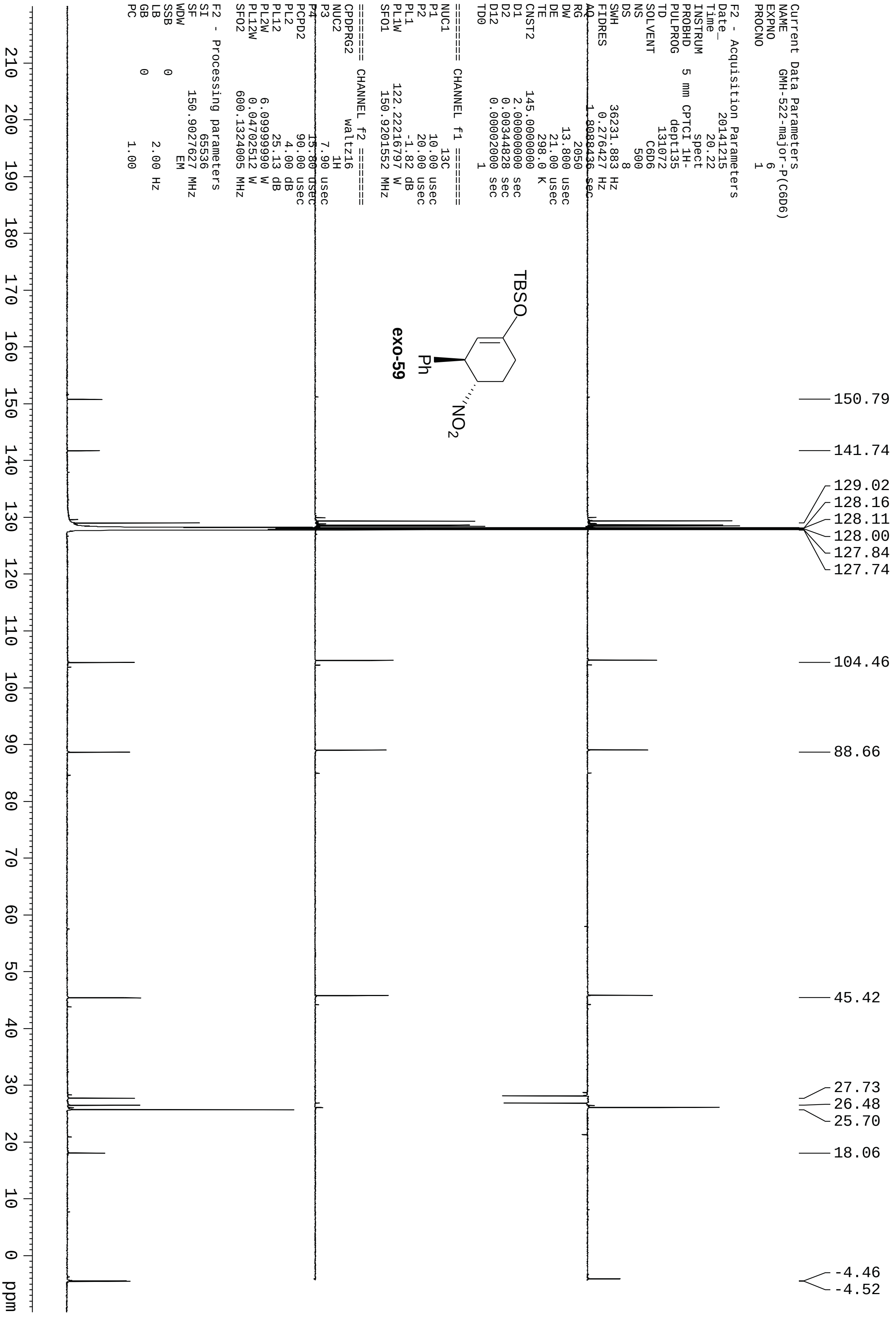

Supplementary Figure 213. ^{13}C and DEPT NMR spectra of compound exo-59.

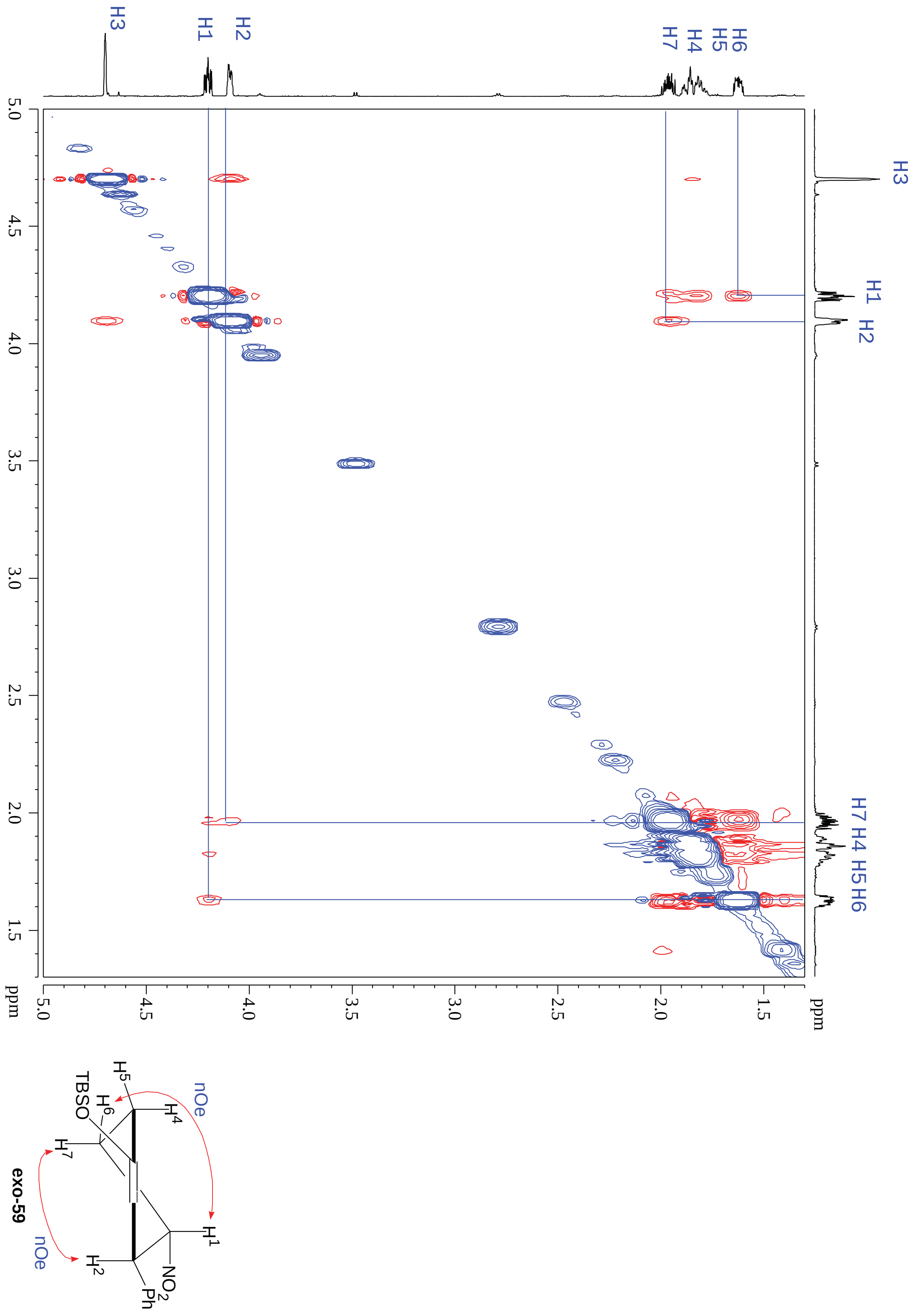

Supplementary Figure 214. NOESY NMR spectrum of compound exo-59.

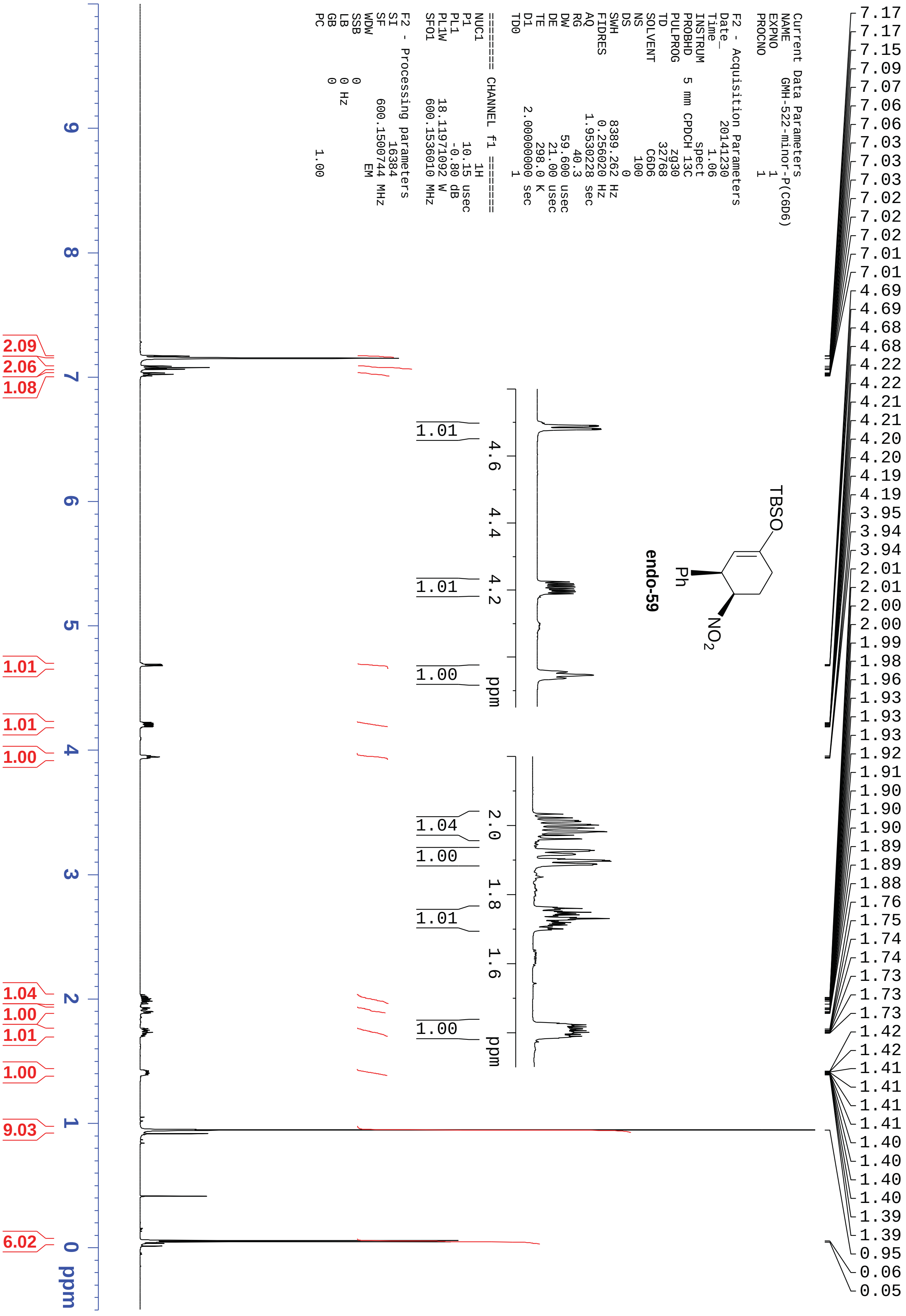

Supplementary Figure 215. ^{1}**H NMR spectrum of compound endo-59.**

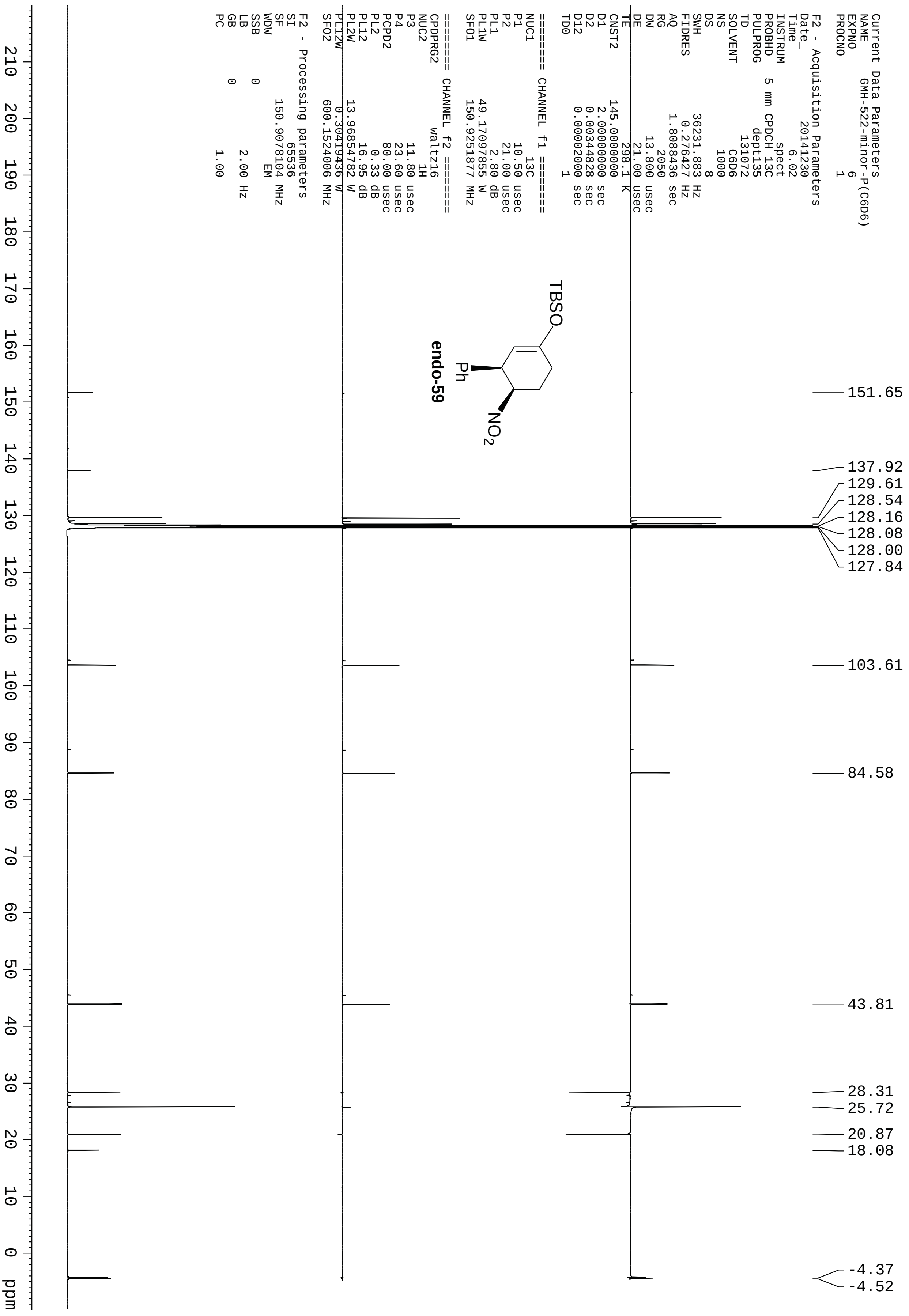

Supplementary Figure 216. ^{13}**C and DEPT NMR spectra of compound endo-59.**

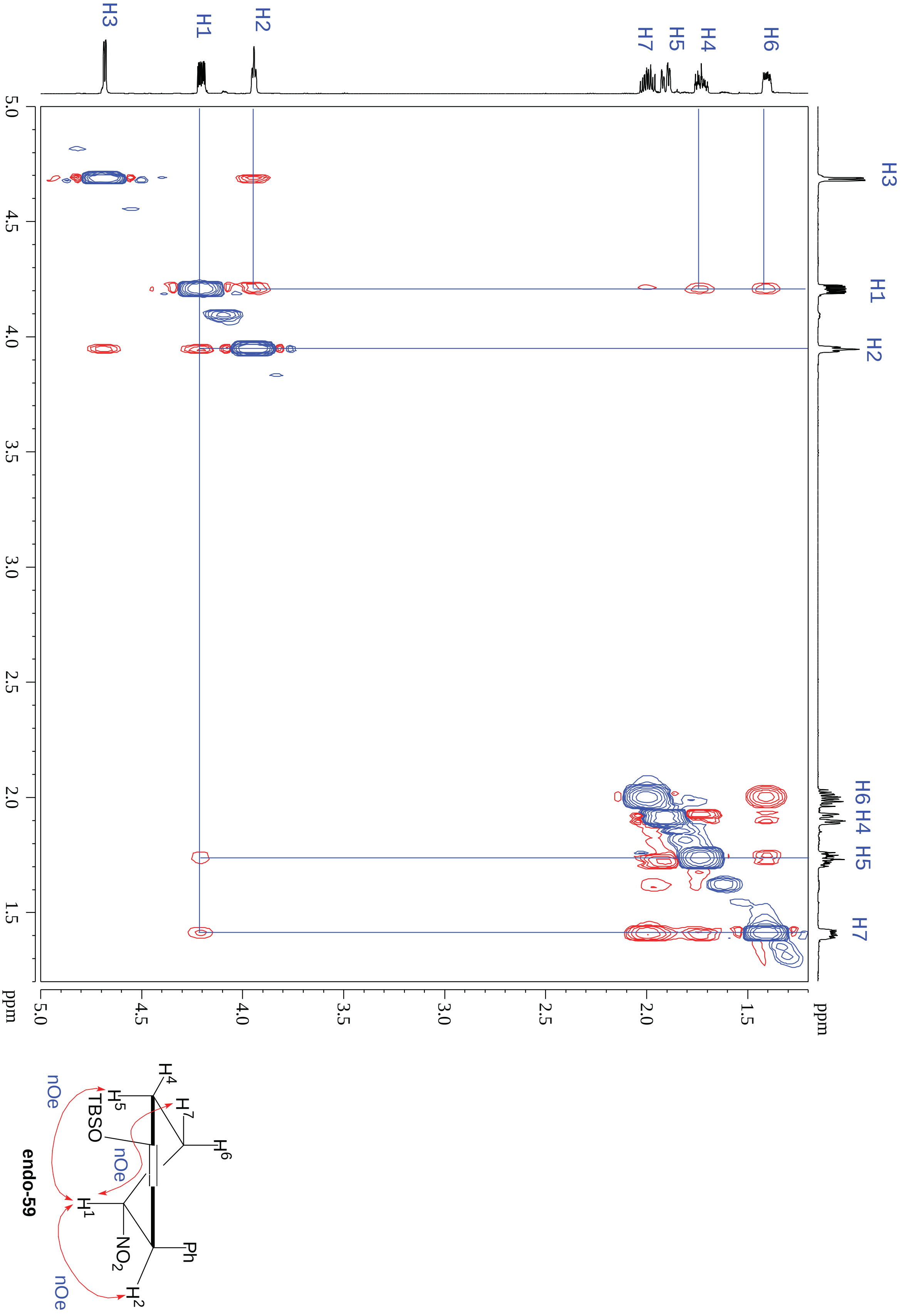

Supplementary Figure 217. NOESY NMR spectrum of compound endo-59.

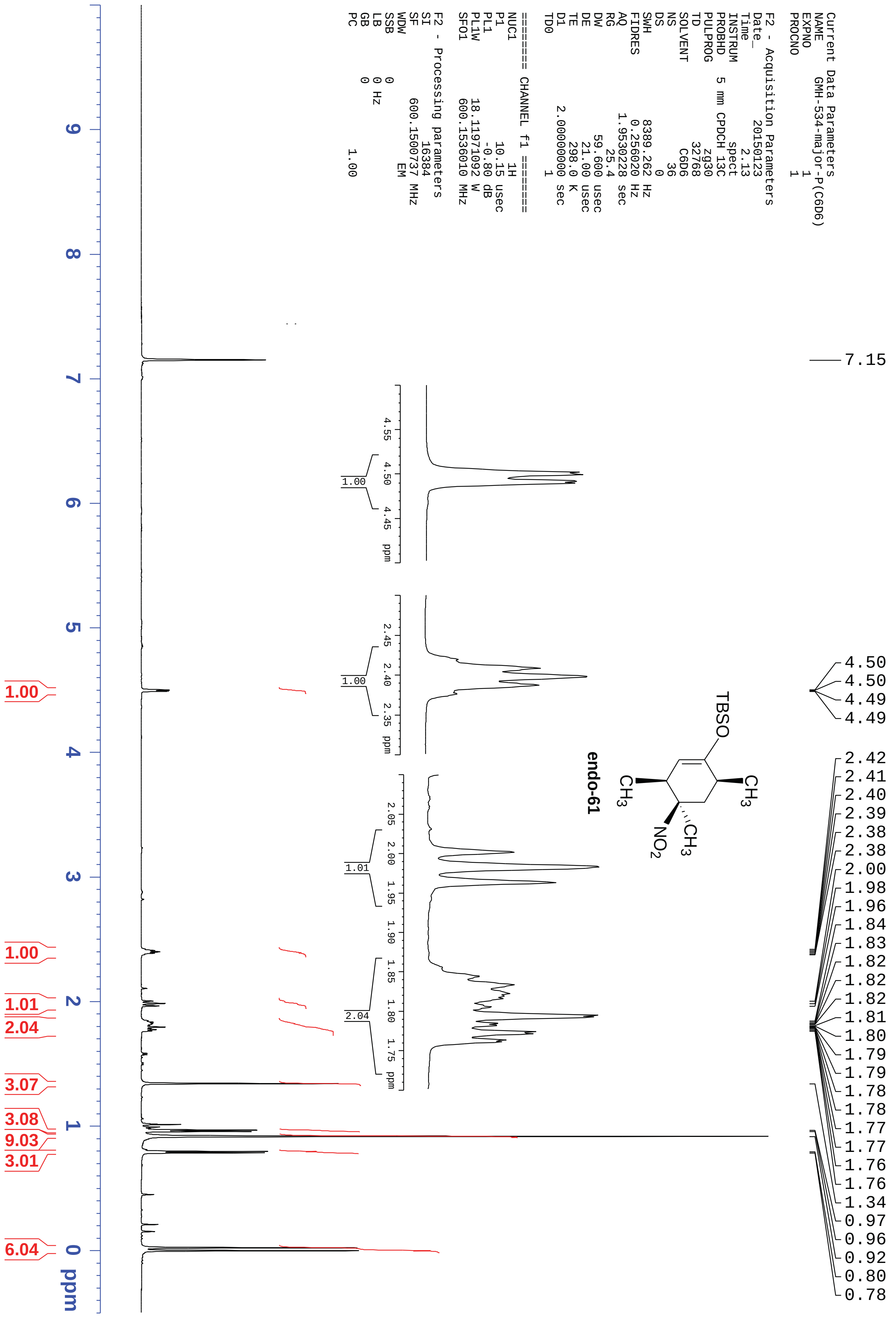

Supplementary Figure 218. **^{1}H NMR spectrum of compound endo-61.**

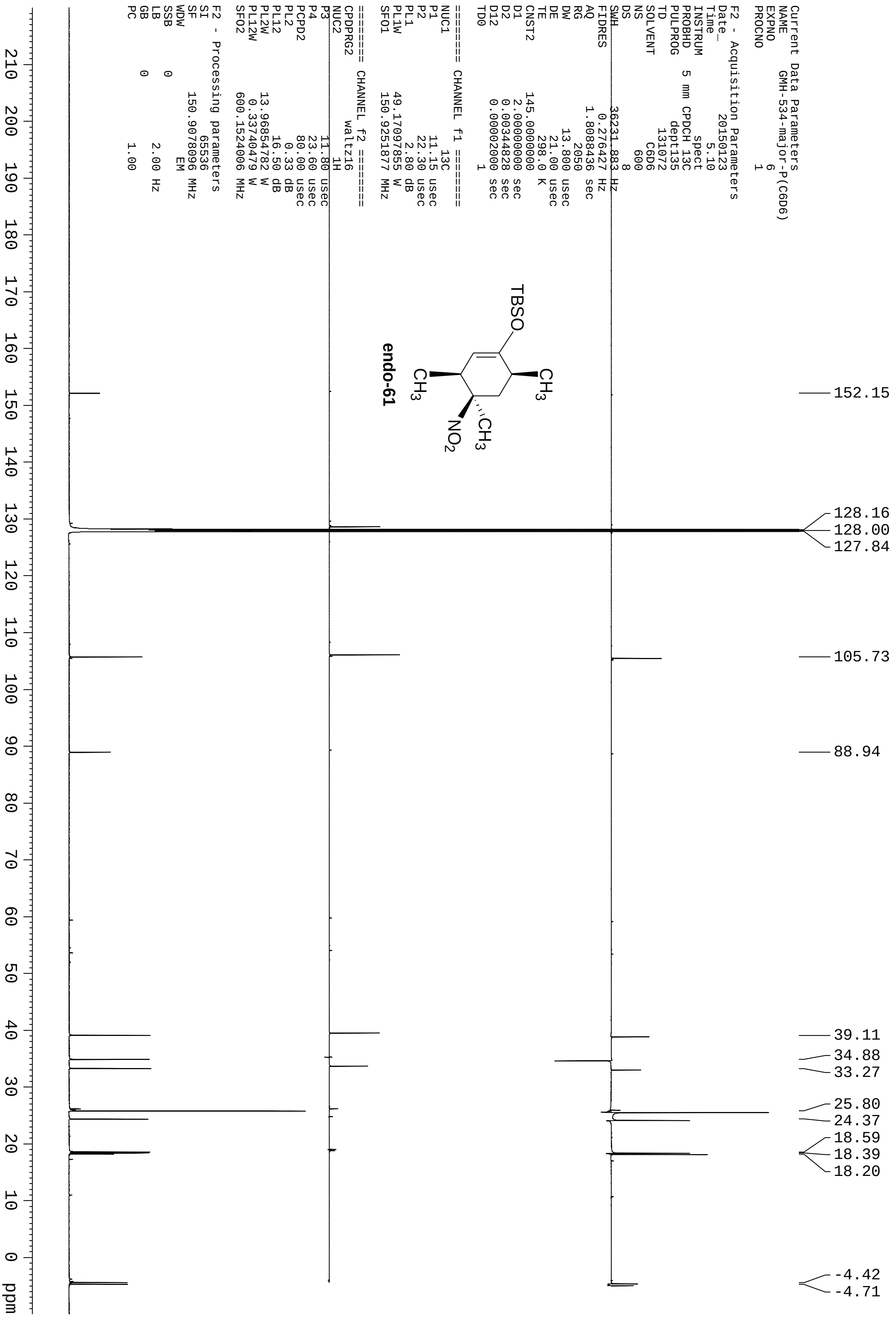

Supplementary Figure 219. ^{13}C and DEPT NMR spectra of compound endo-61.

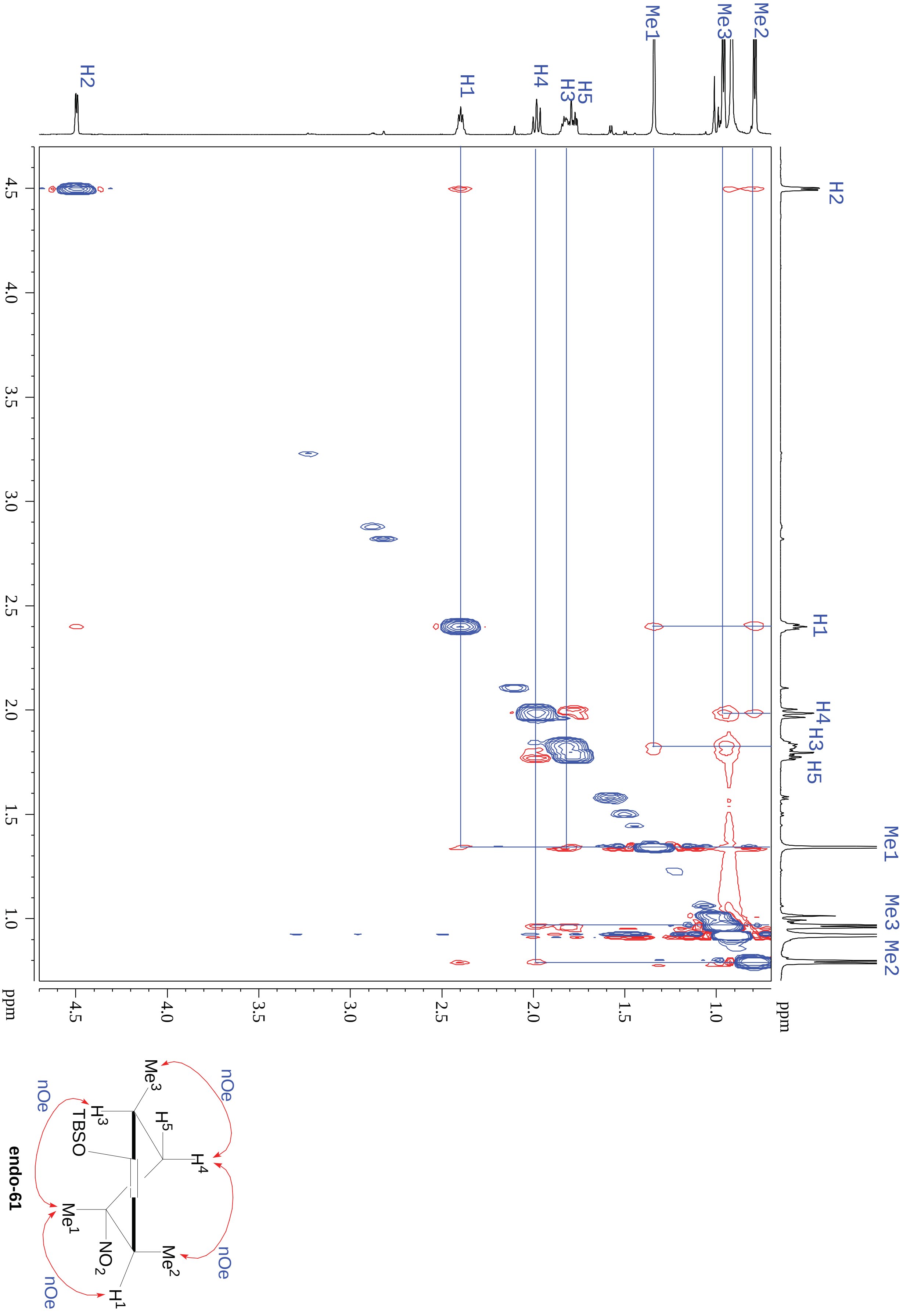

Supplementary Figure 220. NOESY NMR spectrum of compound endo-61.

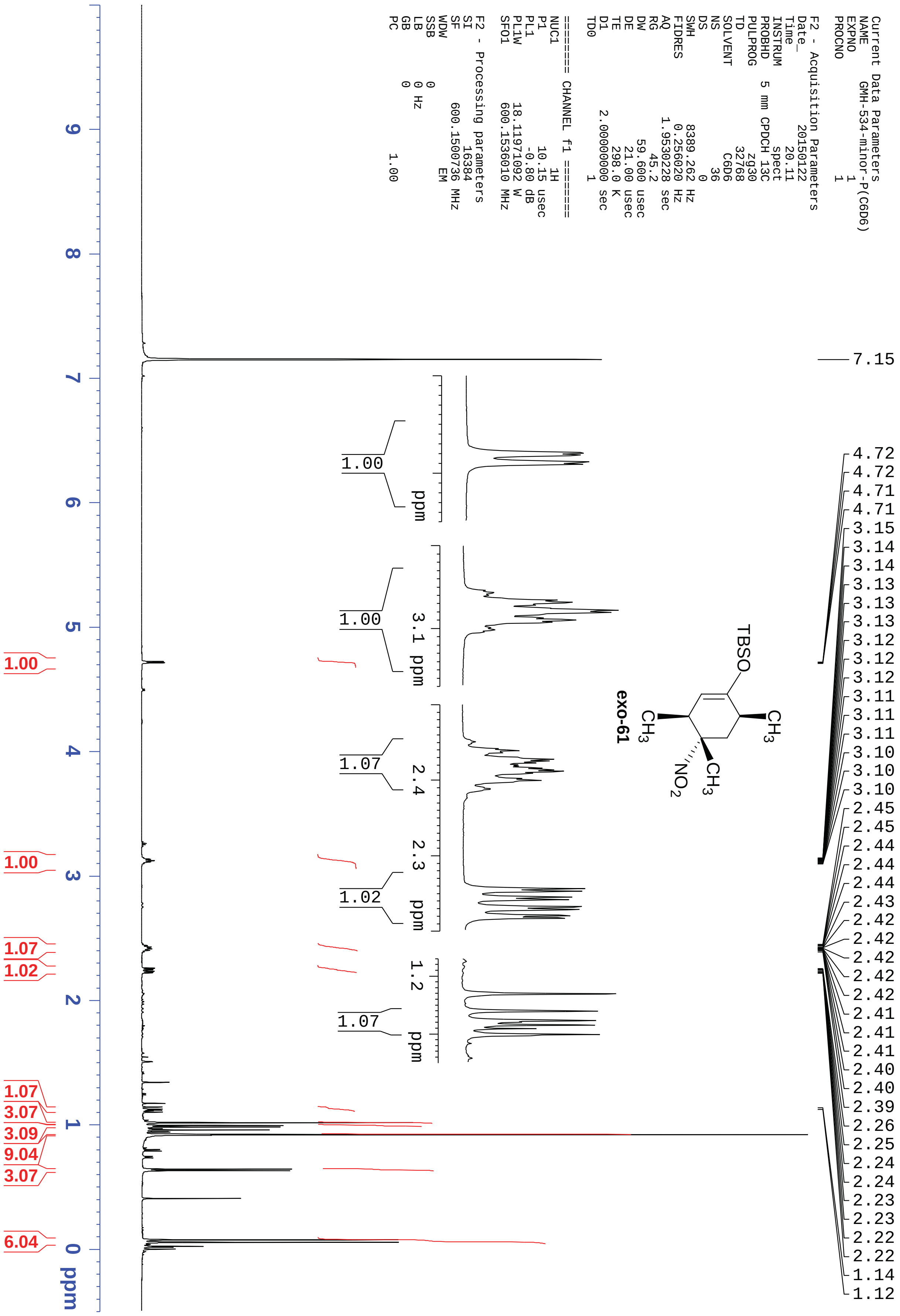

Supplementary Figure 221. ^{1}H NMR spectrum of compound exo-61.

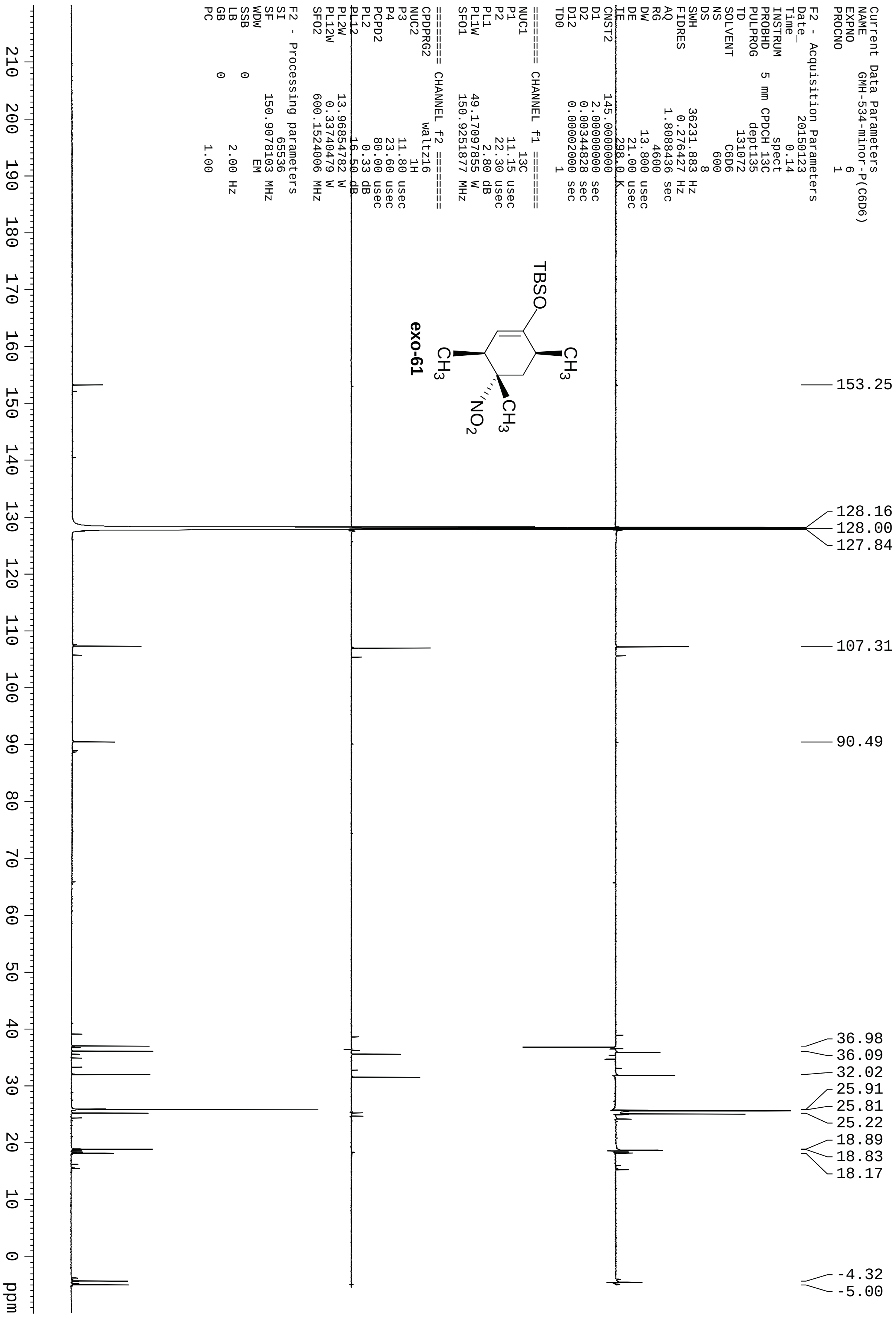

Supplementary Figure 222. ^{13}C and DEPT NMR spectra of compound exo-61.

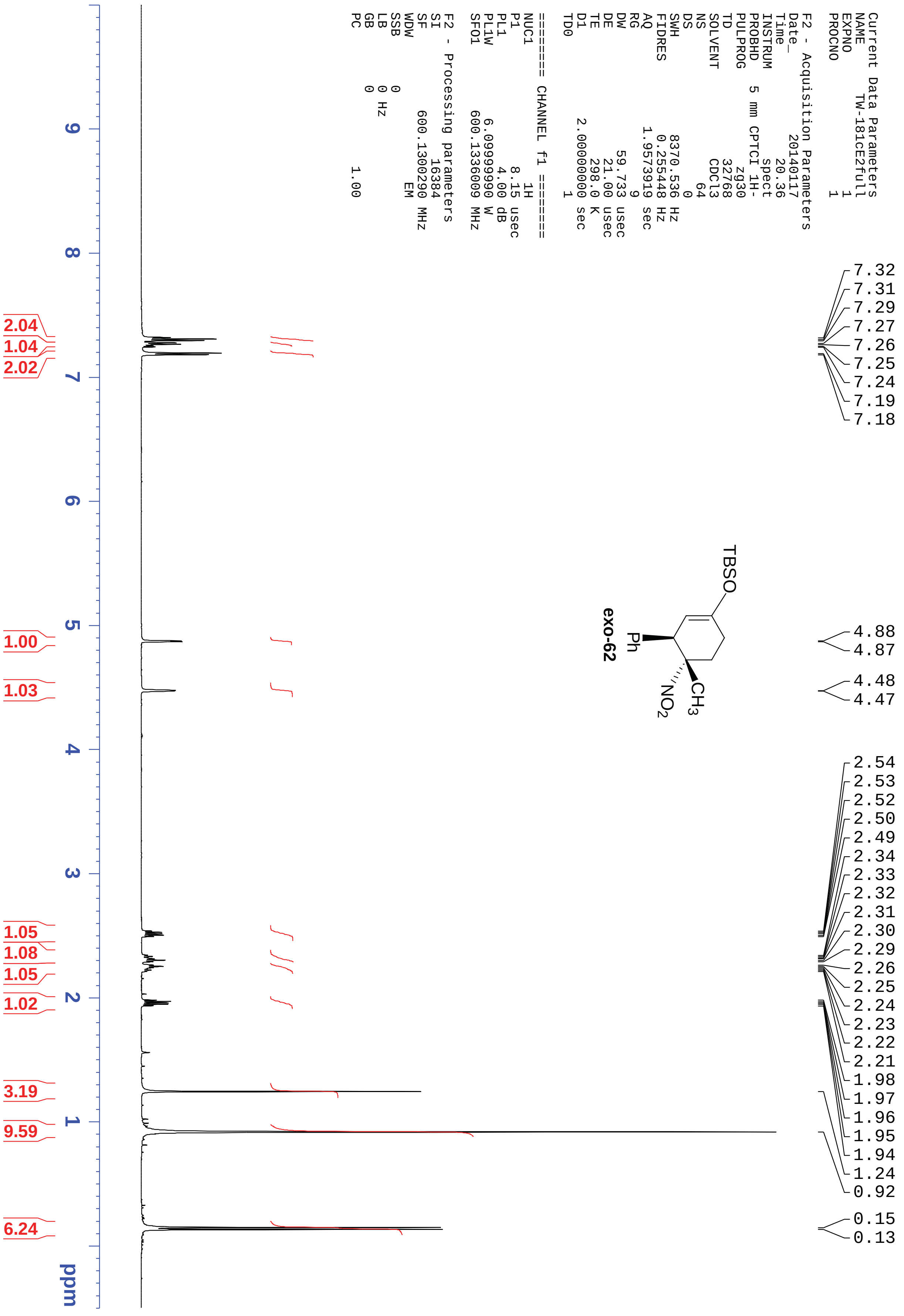

Supplementary Figure 223. ^{1}H NMR spectrum of compound exo-62.

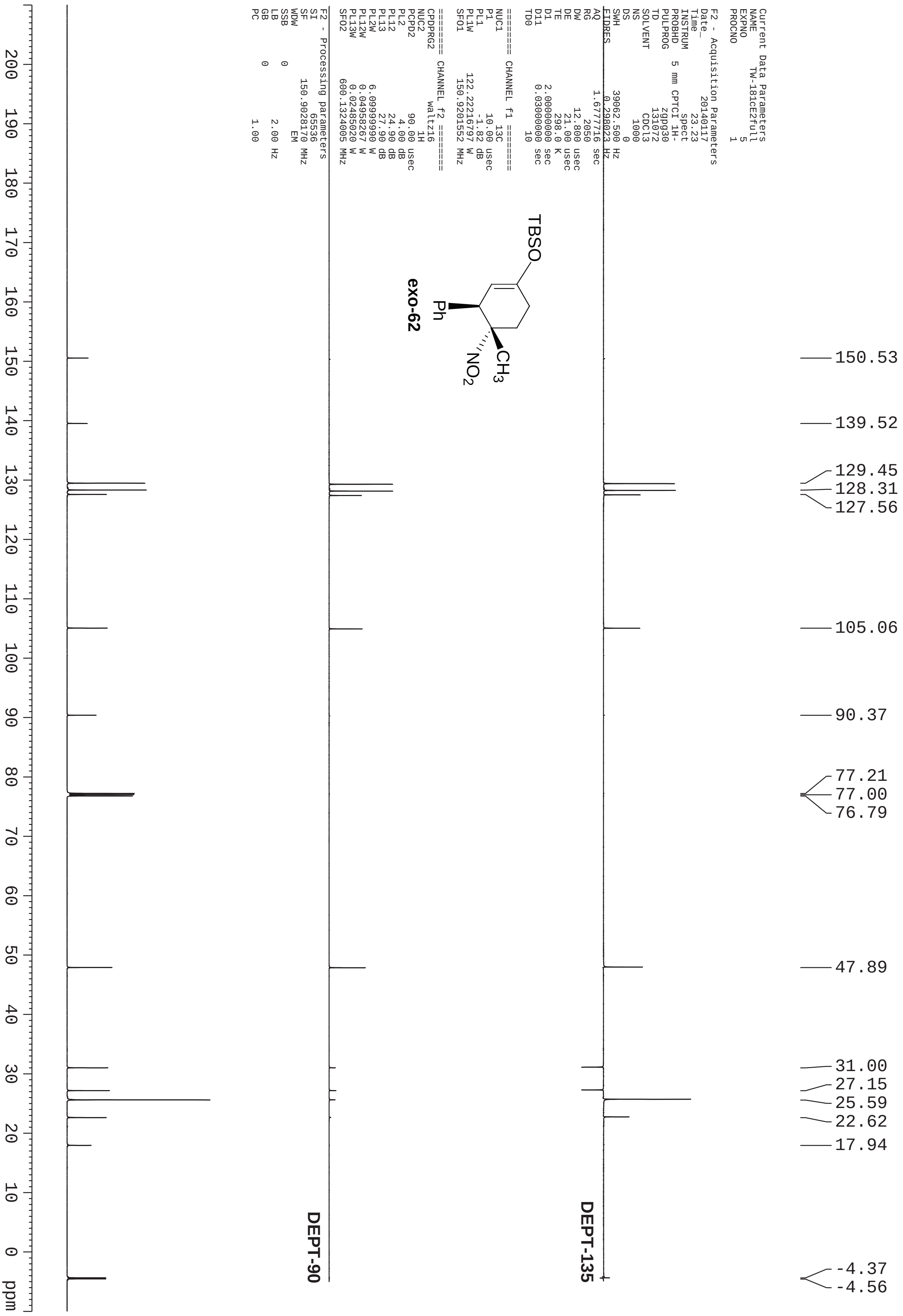

Supplementary Figure 224. ^{13}C and DEPT NMR spectra of compound exo-62.

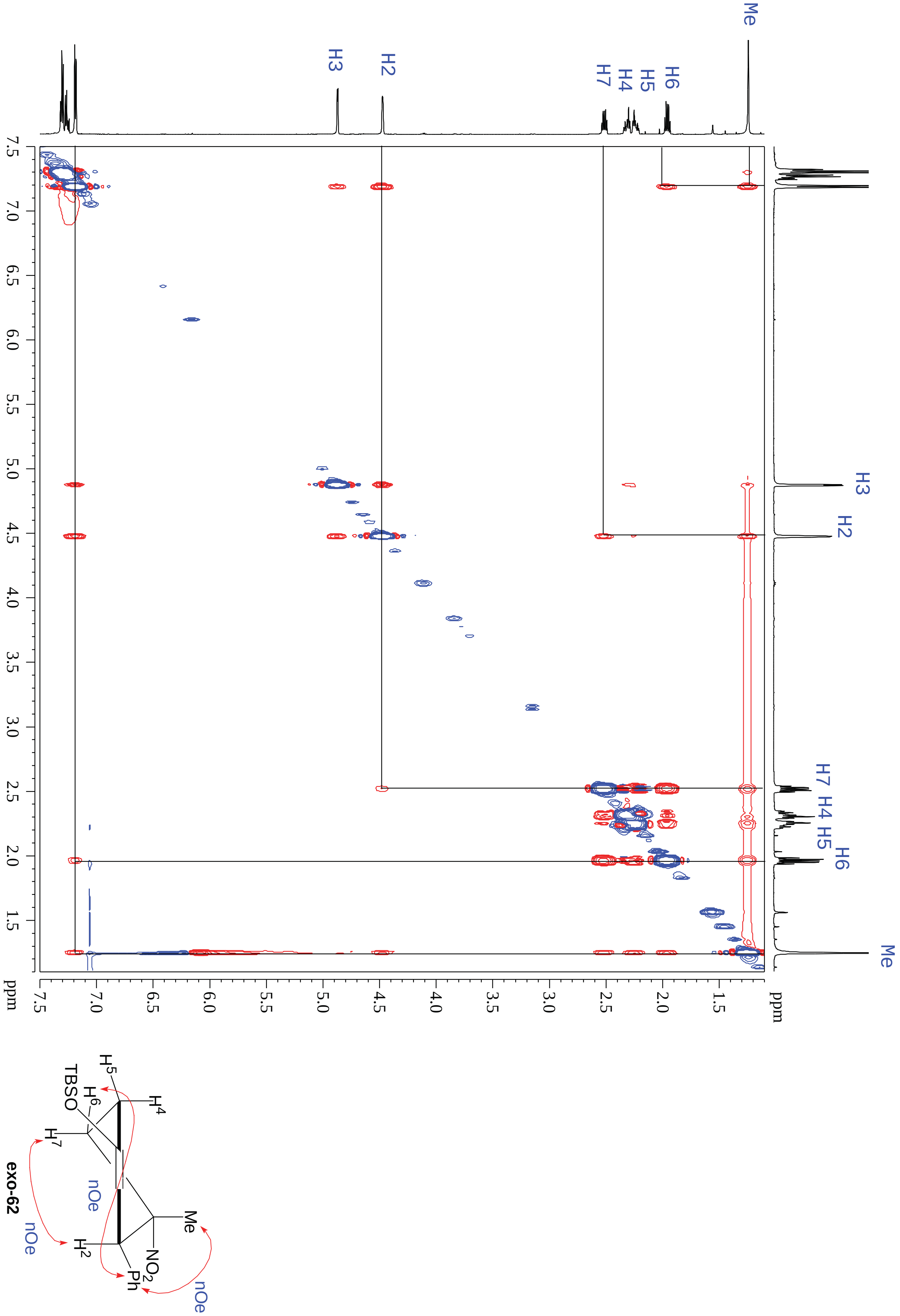

Supplementary Figure 225. NOESY NMR spectrum of compound exo-62.

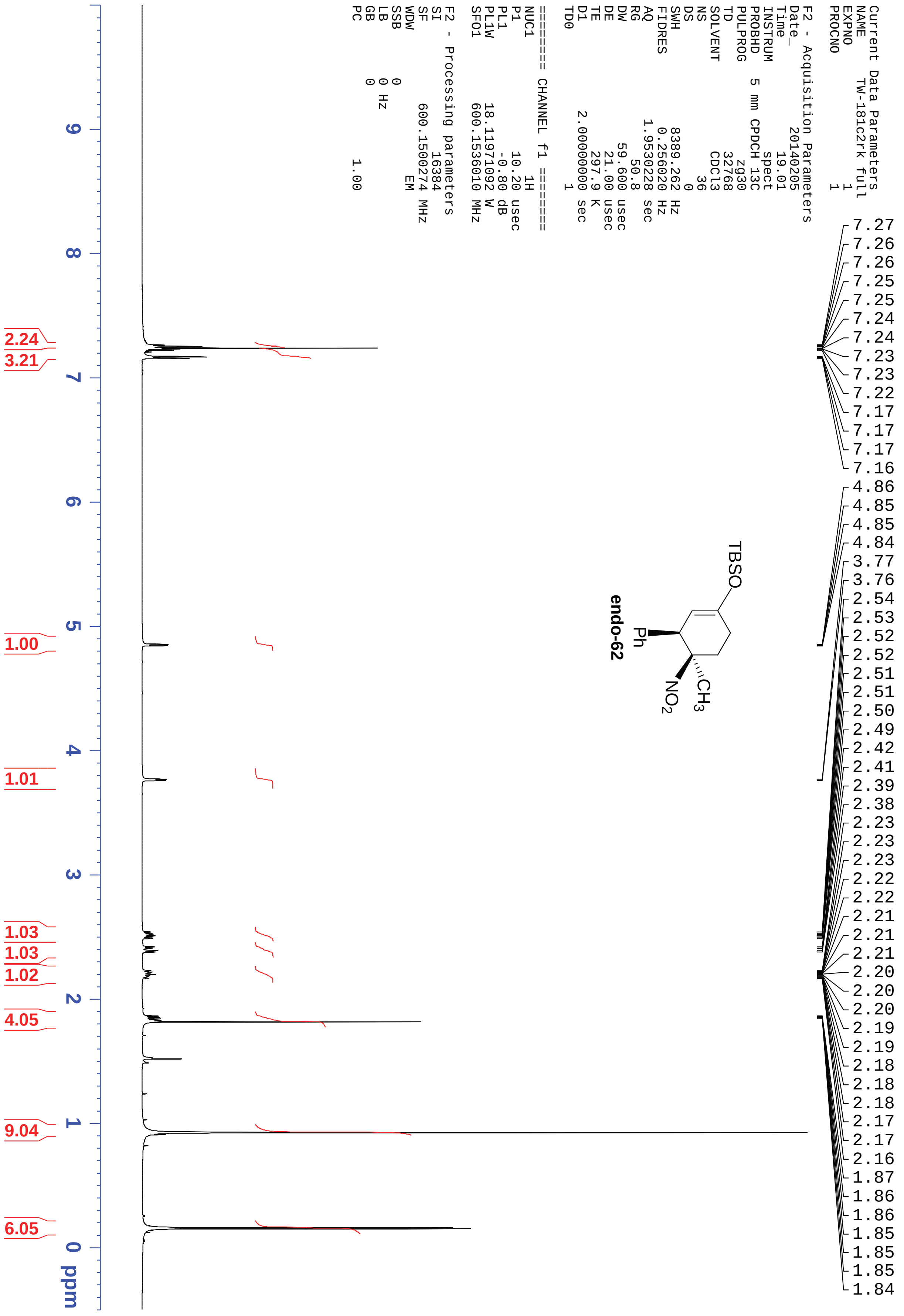

Supplementary Figure 226. **¹H NMR spectrum of compound endo-62.**

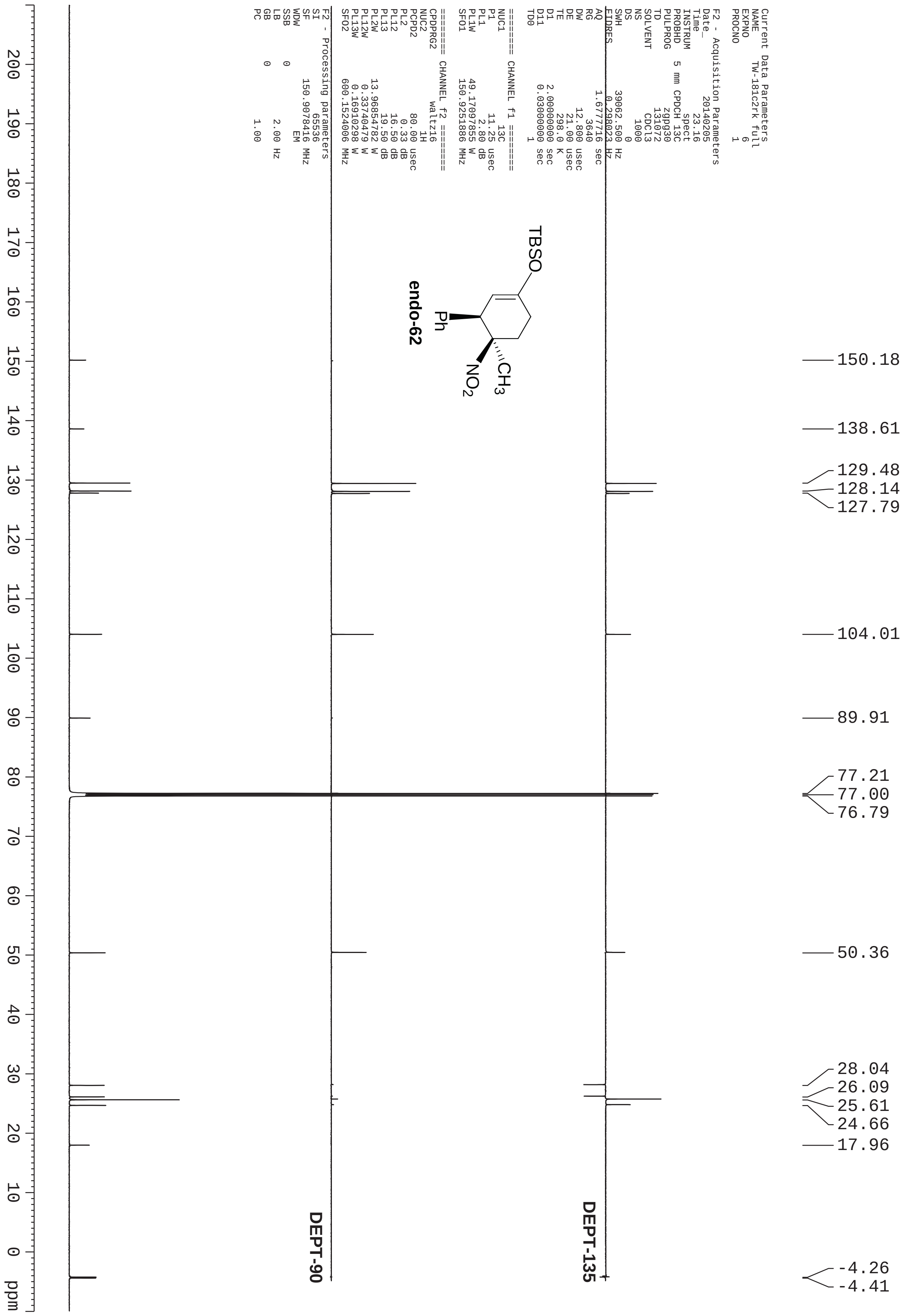

Supplementary Figure 227. ^{13}C and DEPT NMR spectra of compound endo-62.

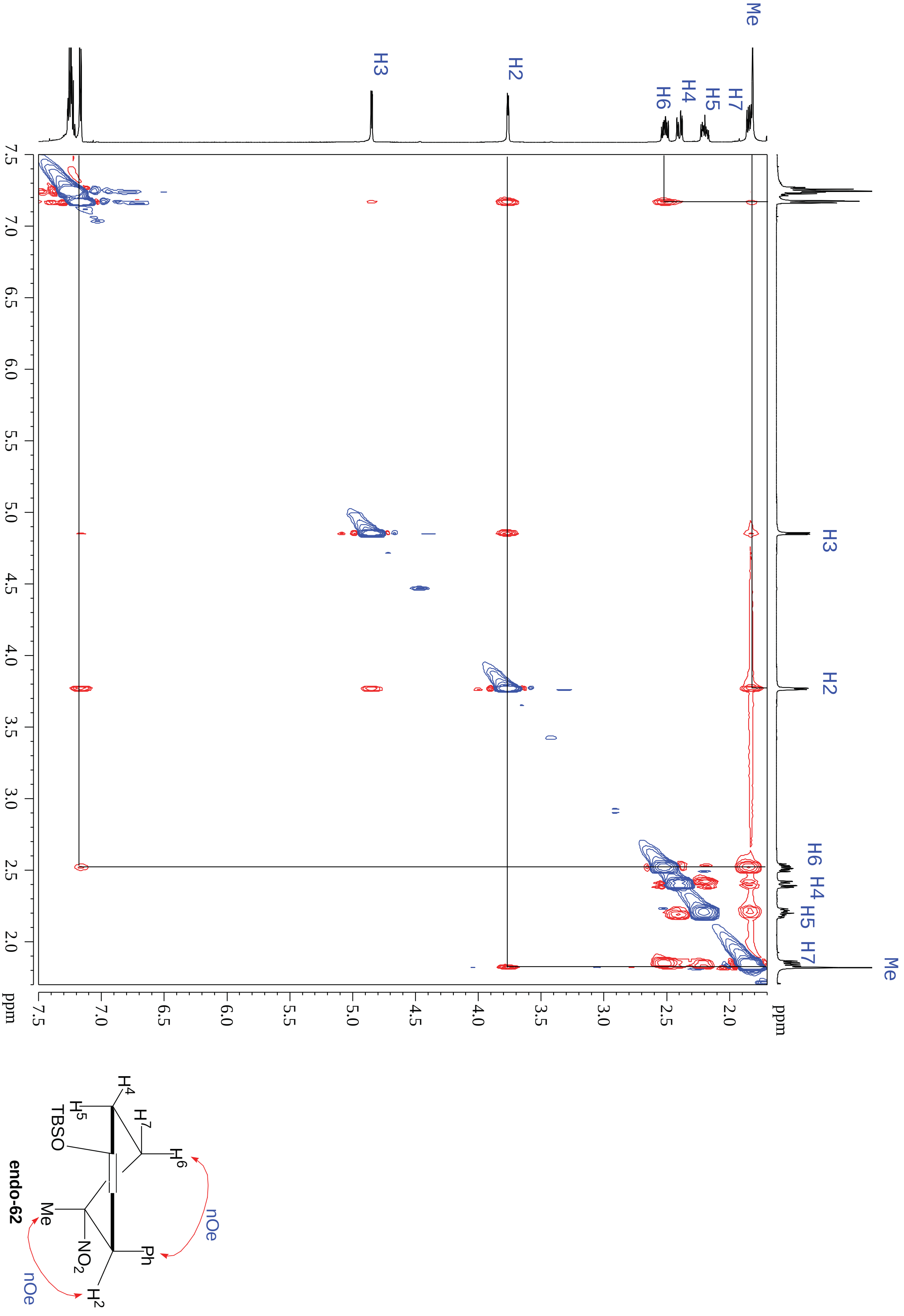

Supplementary Figure 228. NOESY NMR spectrum of compound endo-62.

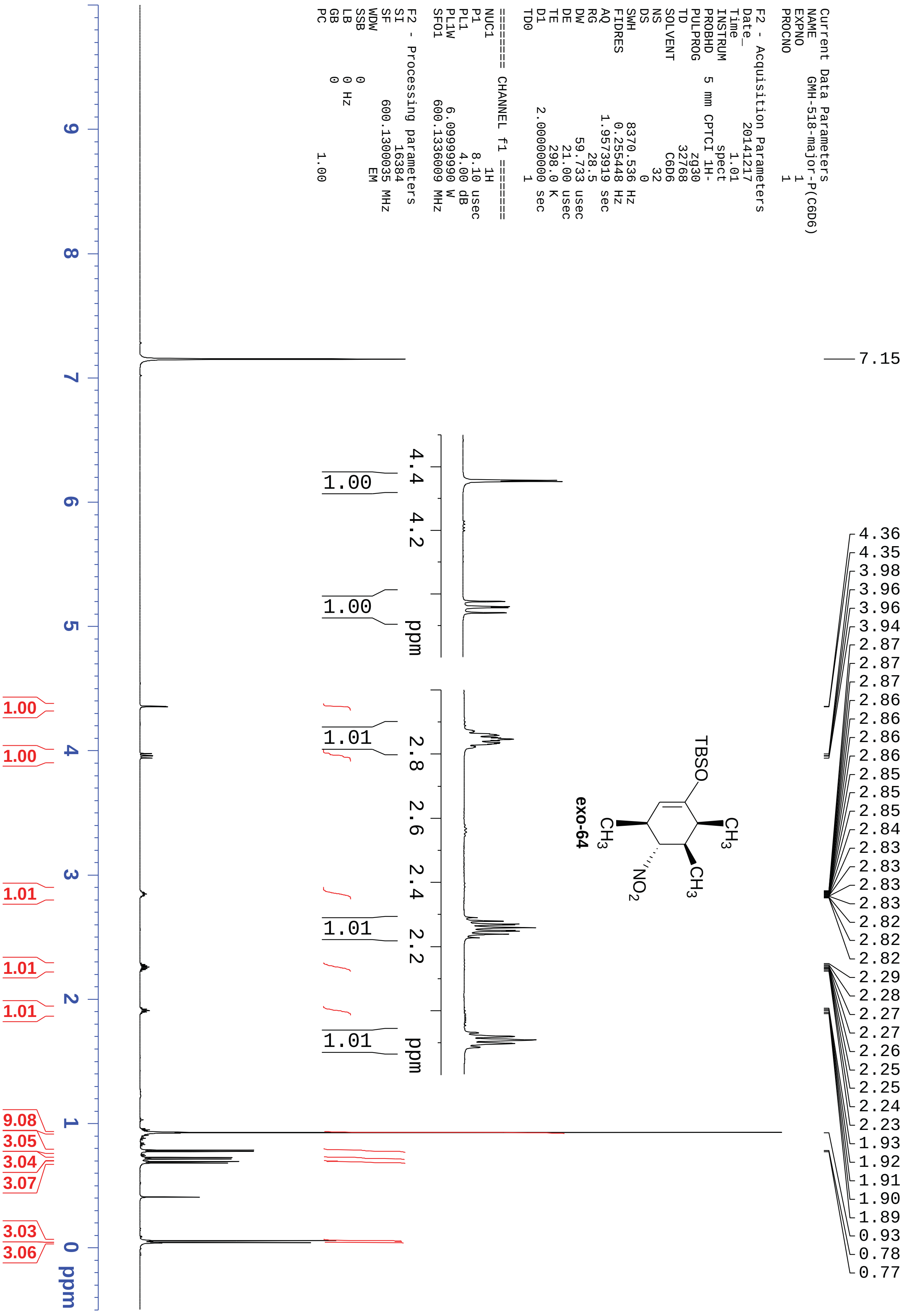

Supplementary Figure 229. **^{1}H NMR spectrum of compound exo-64.**

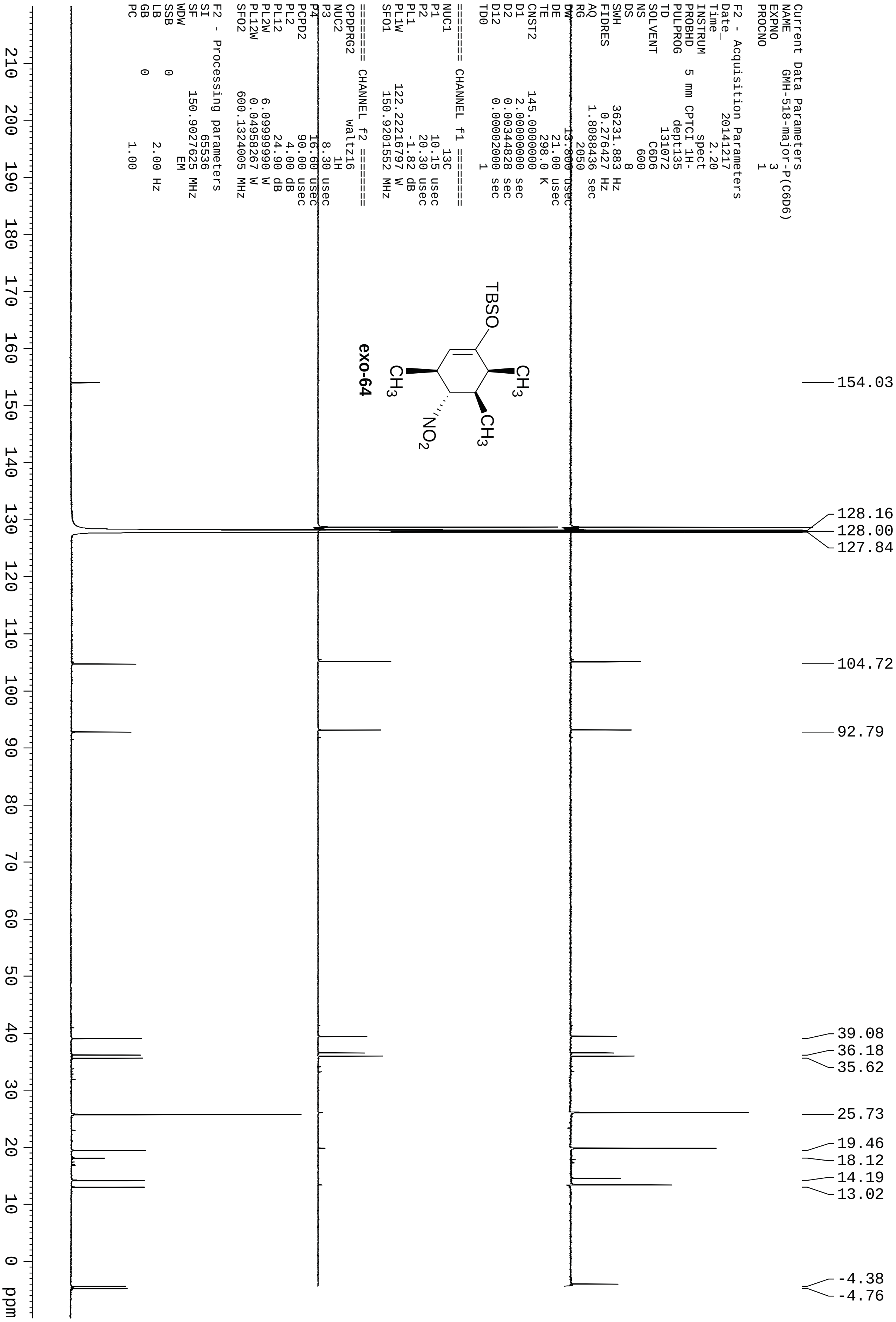

Supplementary Figure 230. **^{13}C and DEPT NMR spectra of compound exo-64.**

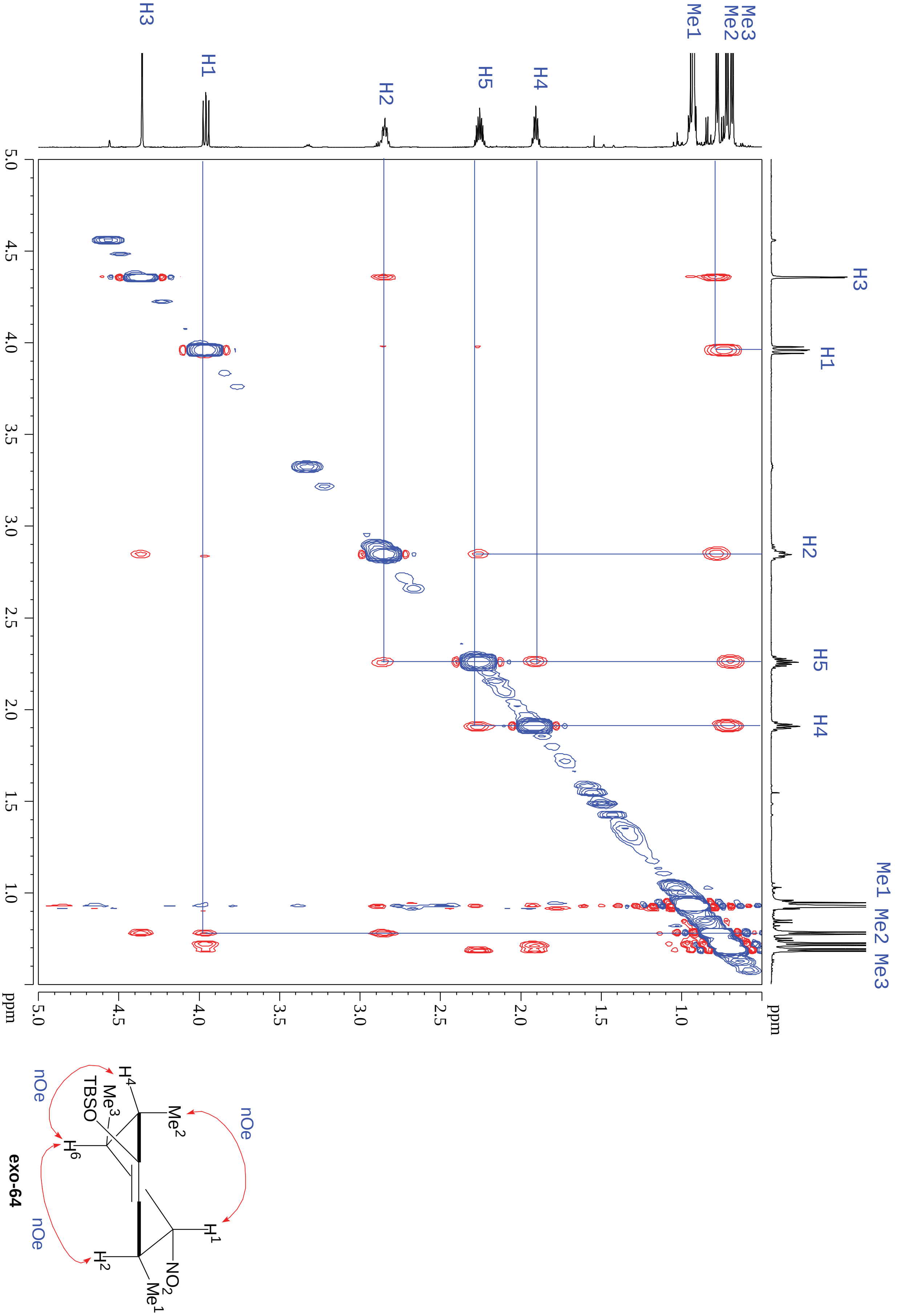

Supplementary Figure 231. NOESY NMR spectrum of compound exo-64.

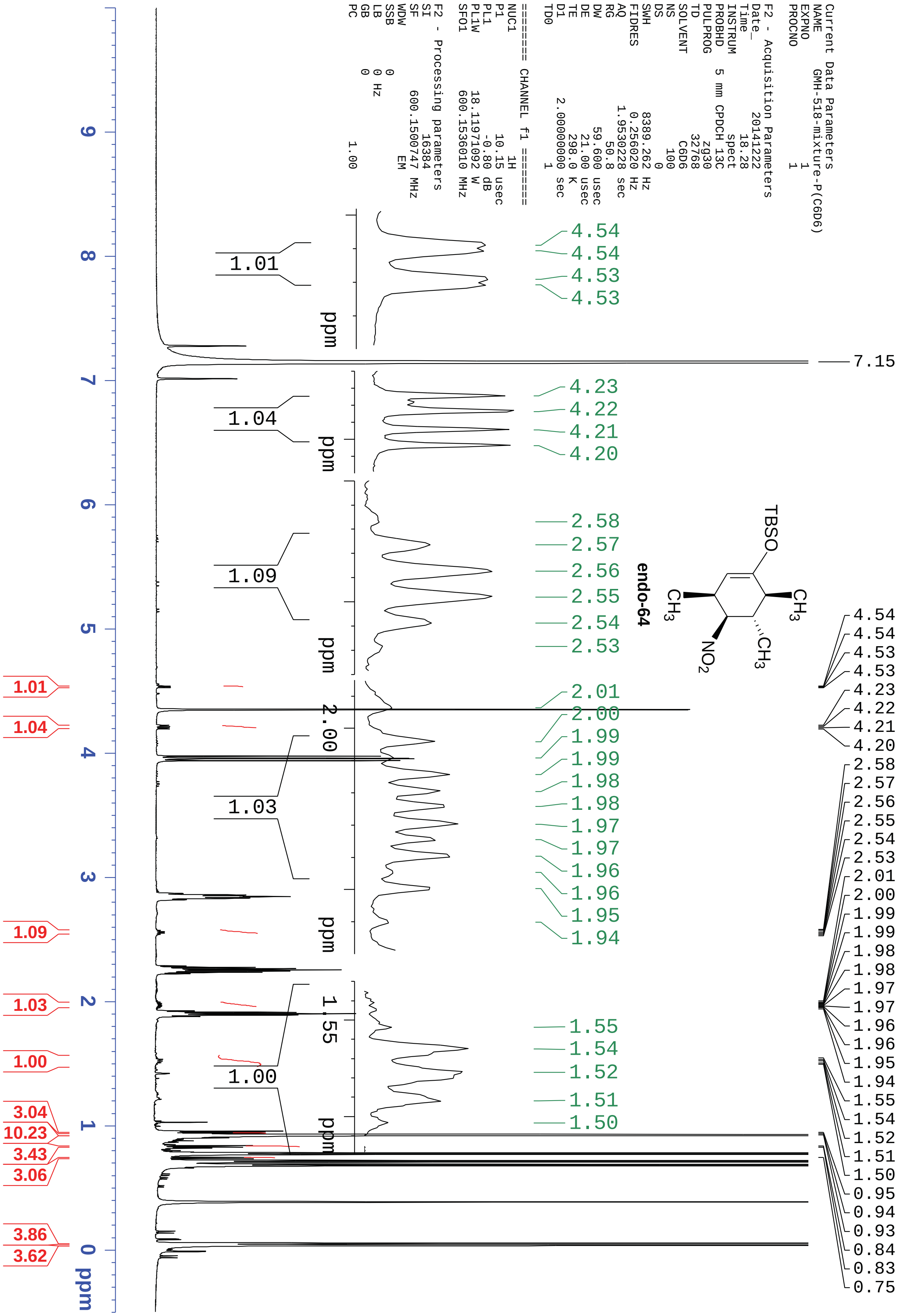

Supplementary Figure 232. ^{1}**H NMR spectrum of compound endo-64.**

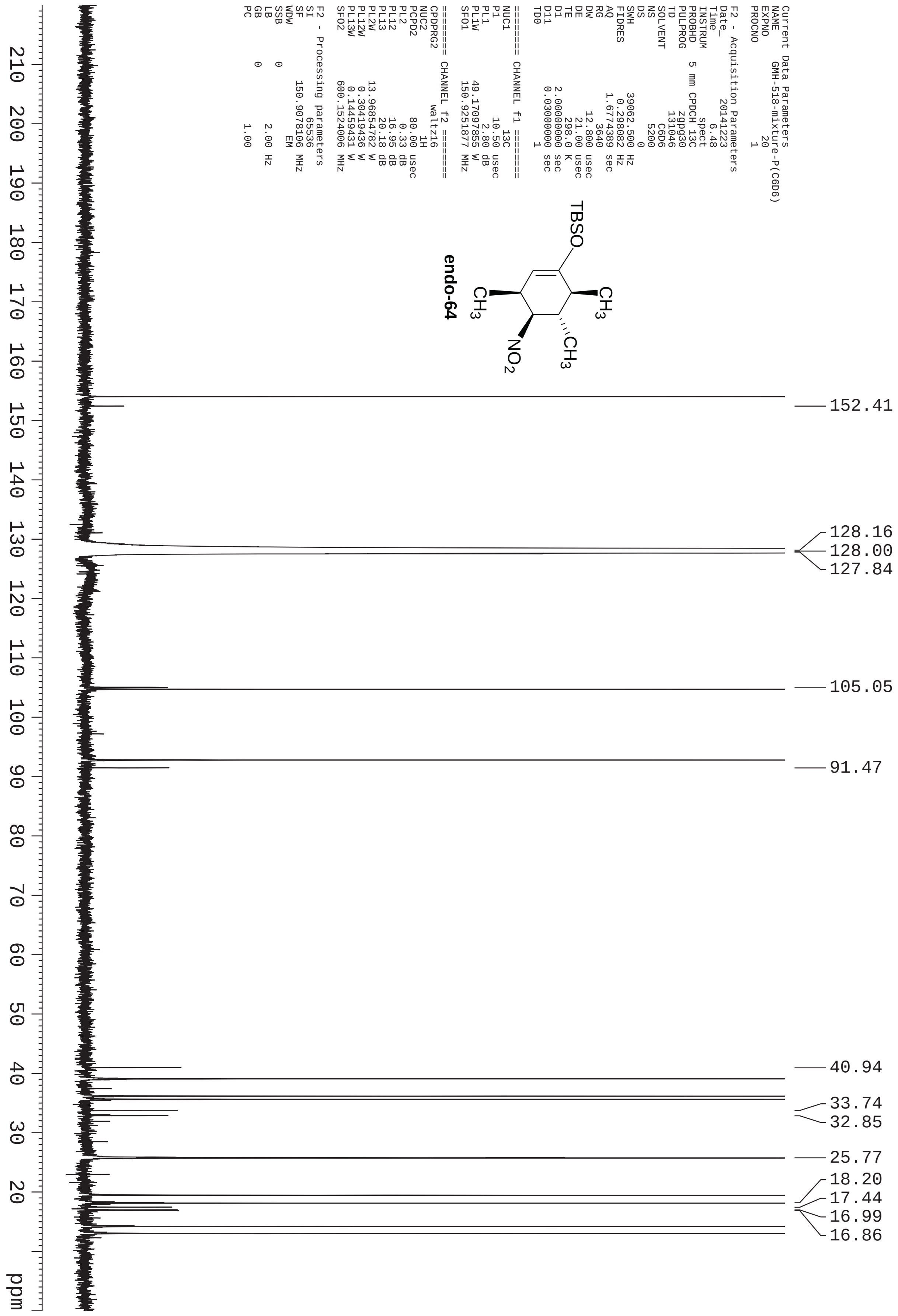

Supplementary Figure 233. ^{13}C NMR spectrum of compound endo-64.

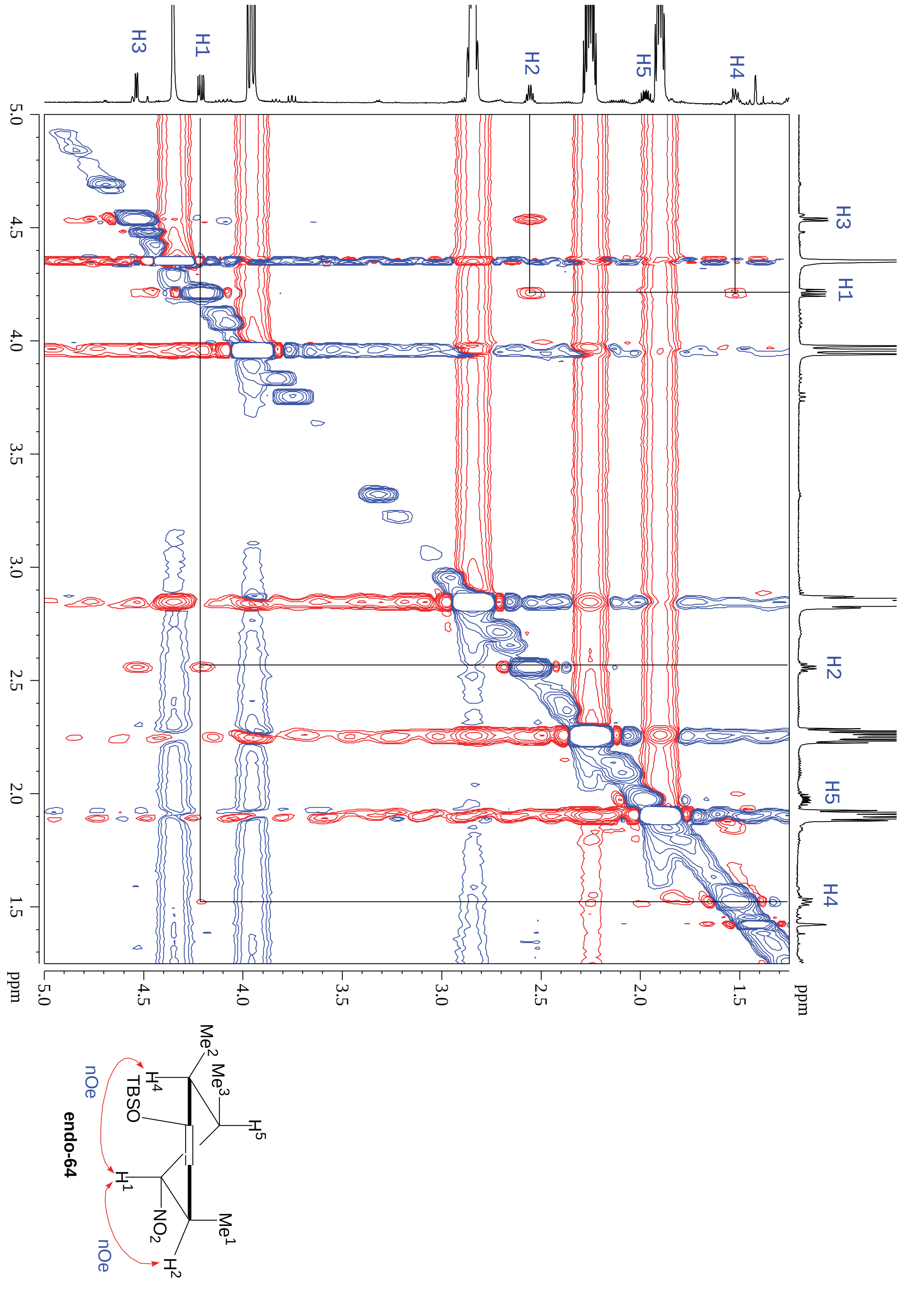

Supplementary Figure 234. NOESY NMR spectrum of mixed compound endo/exo-64 highlighting the correlation for endo-64.

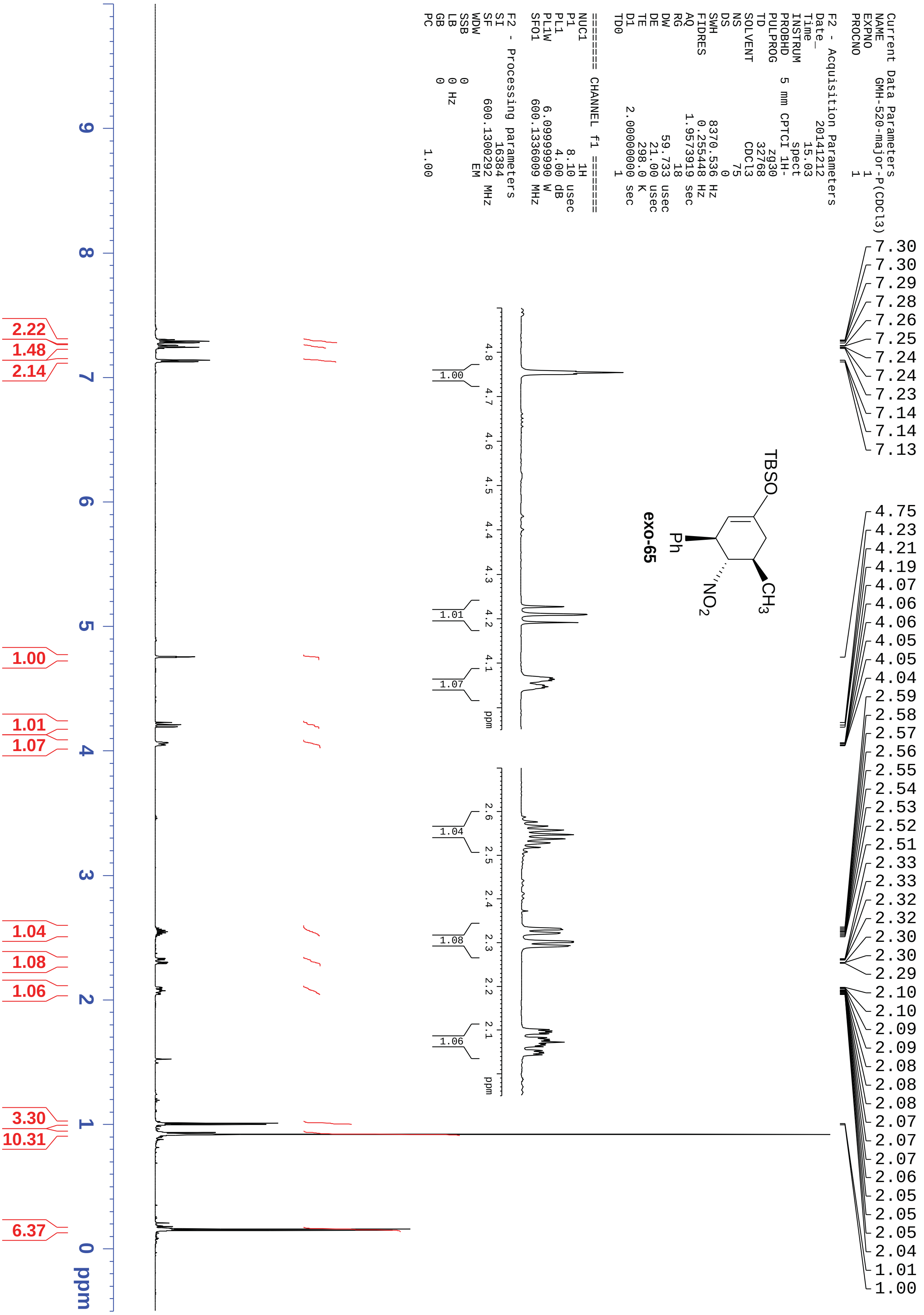

Supplementary Figure 235. ^{1}H NMR spectrum of compound exo-65.

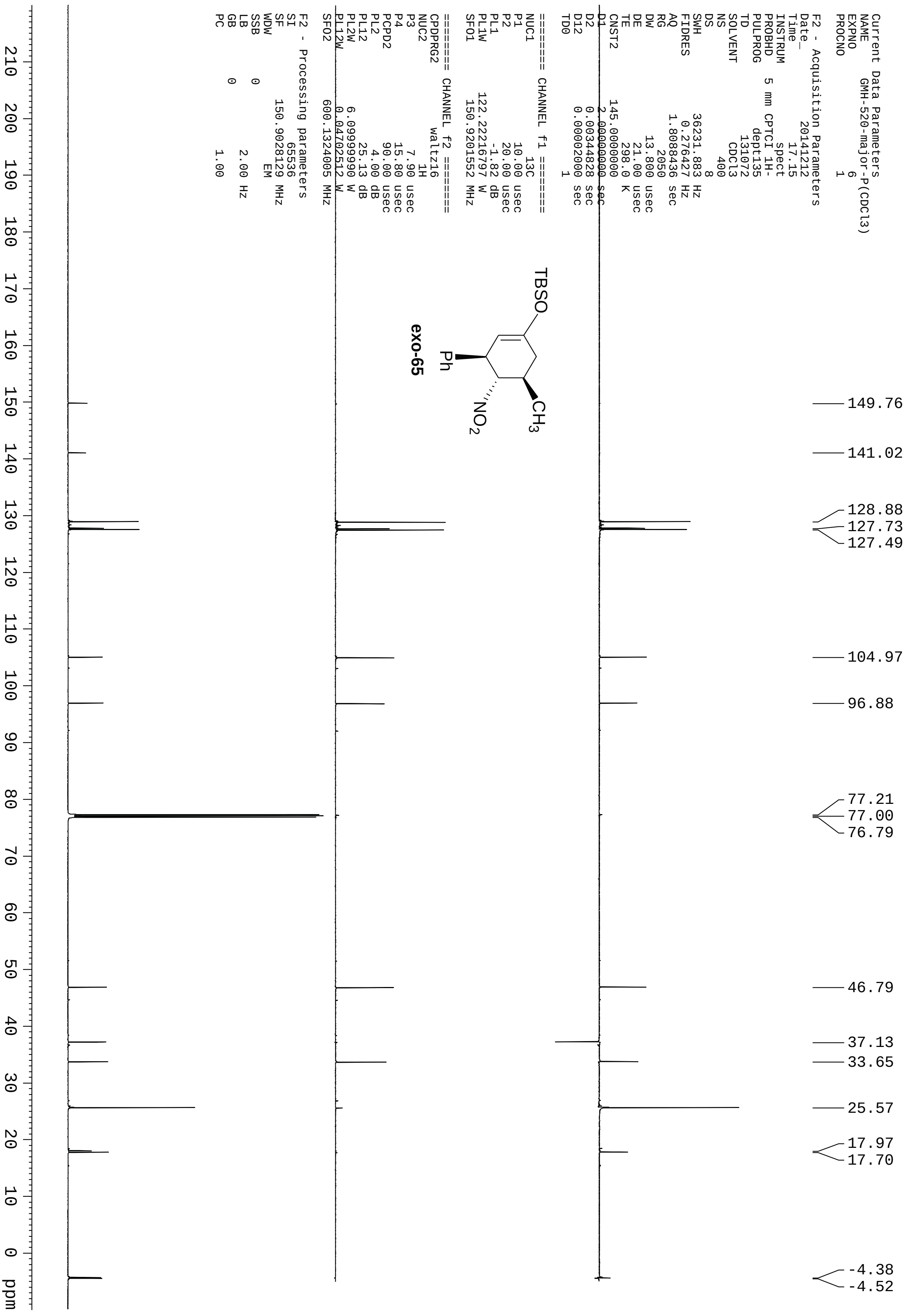

Supplementary Figure 236. ^{13}C and DEPT NMR spectra of compound exo-65.

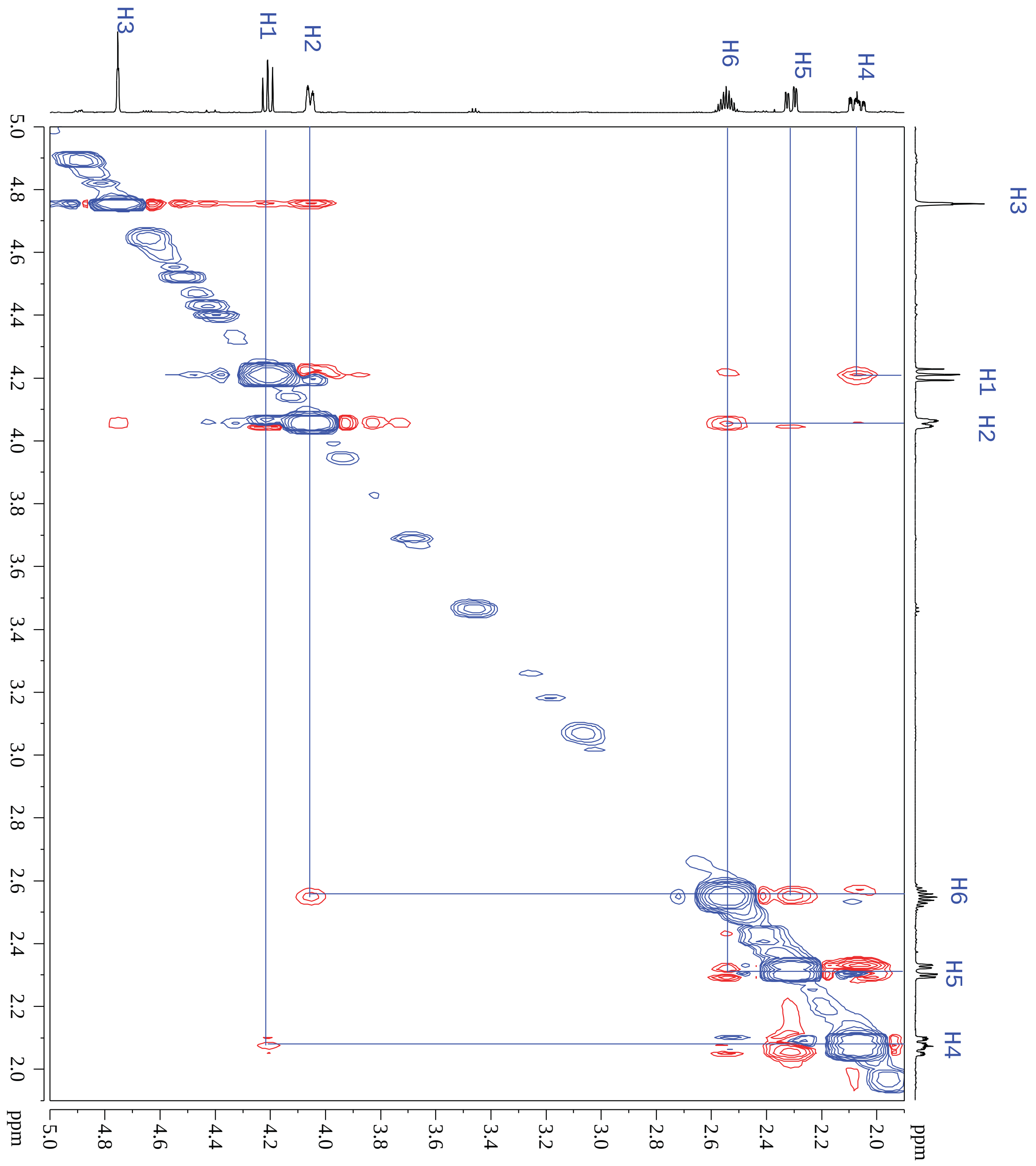

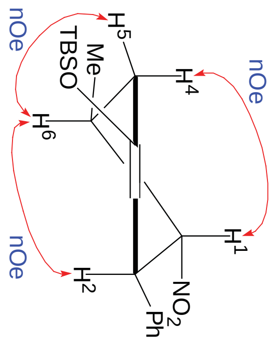

exo-65

Supplementary Figure 237. NOESY NMR spectrum of compound exo-65.

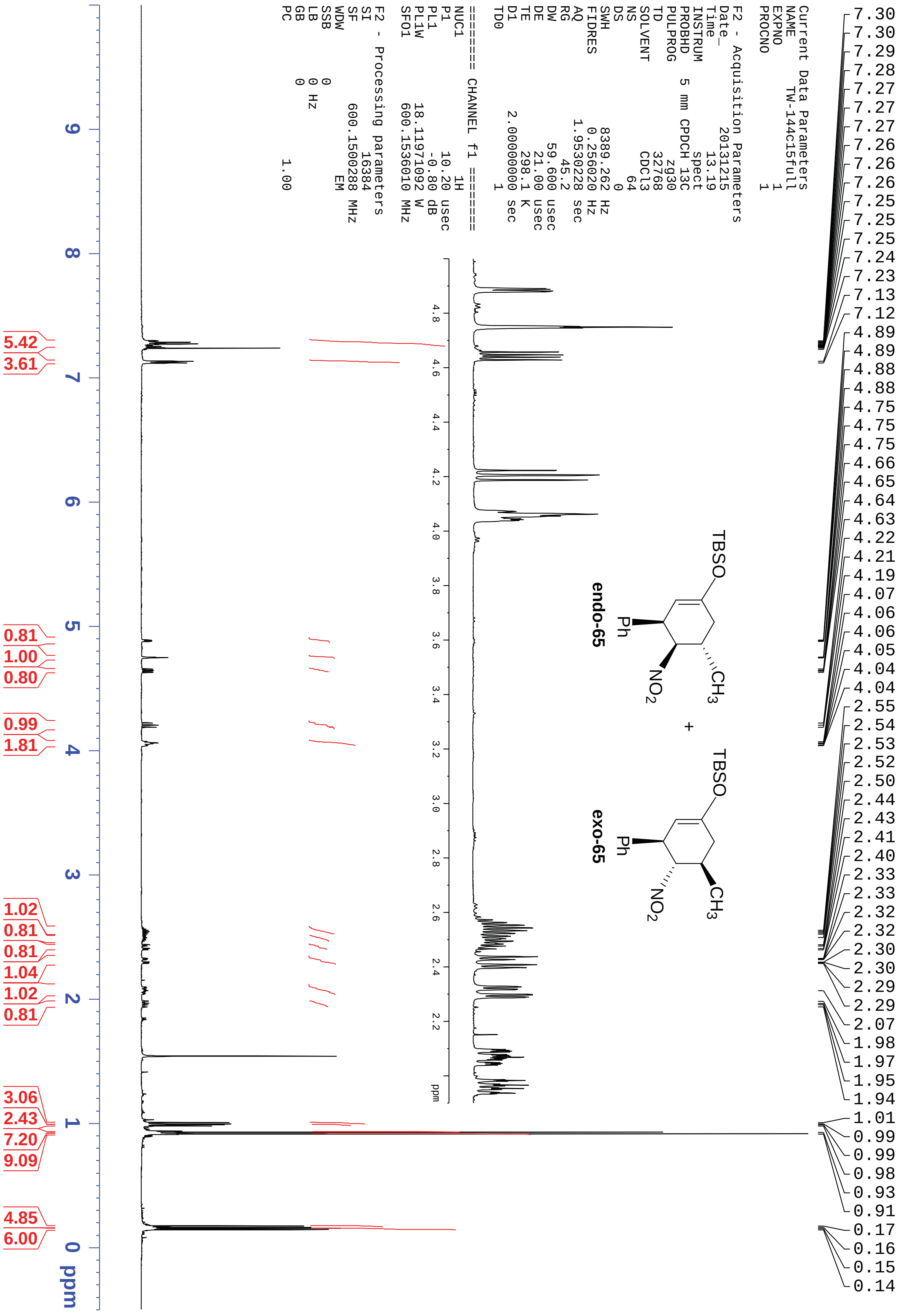

Supplementary Figure 238. ^{1}H NMR spectrum of mixed compound endo/exo-65.

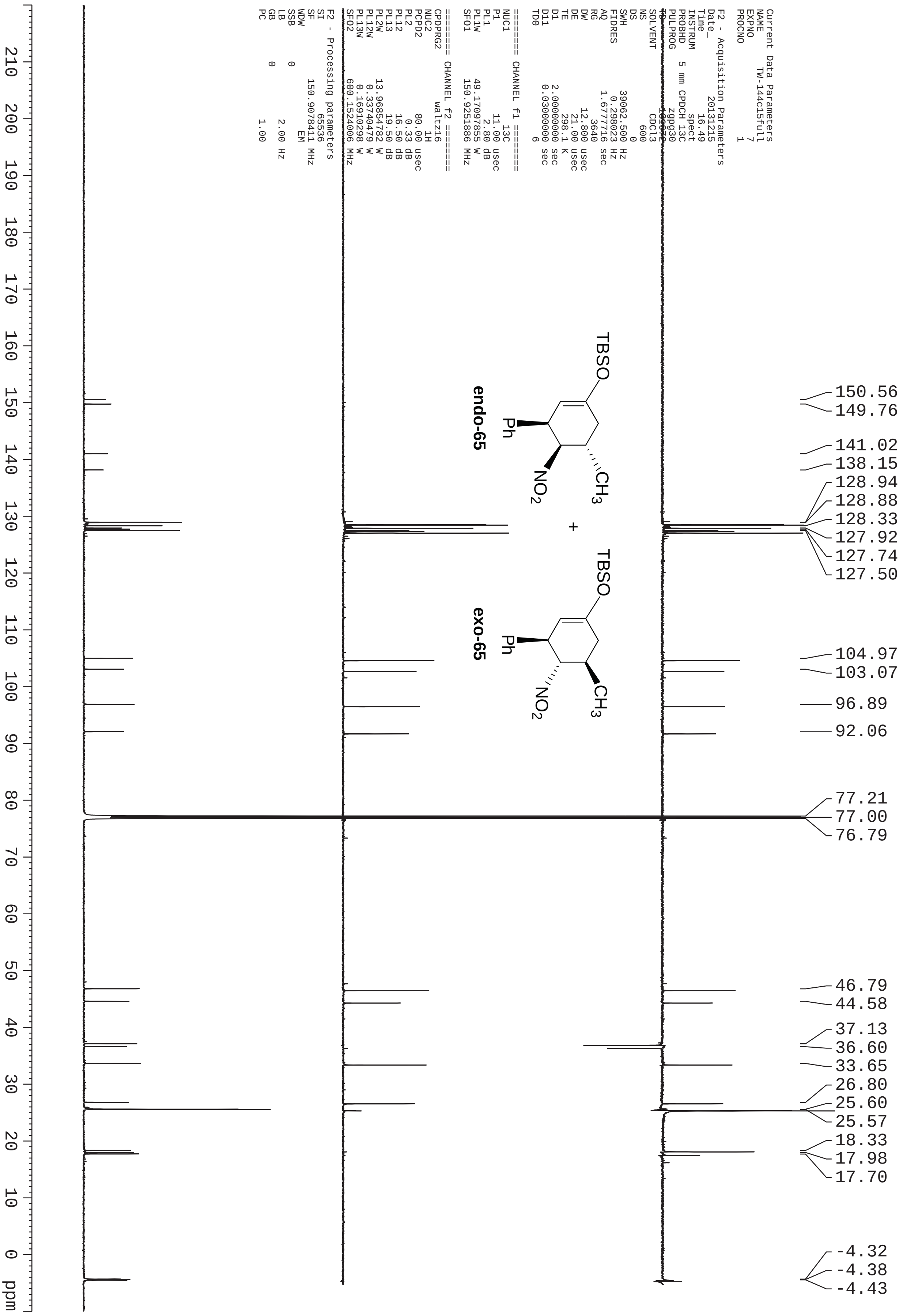

Supplementary Figure 239. ¹³C and DEPT NMR spectra of mixed compound endo/exo-65.

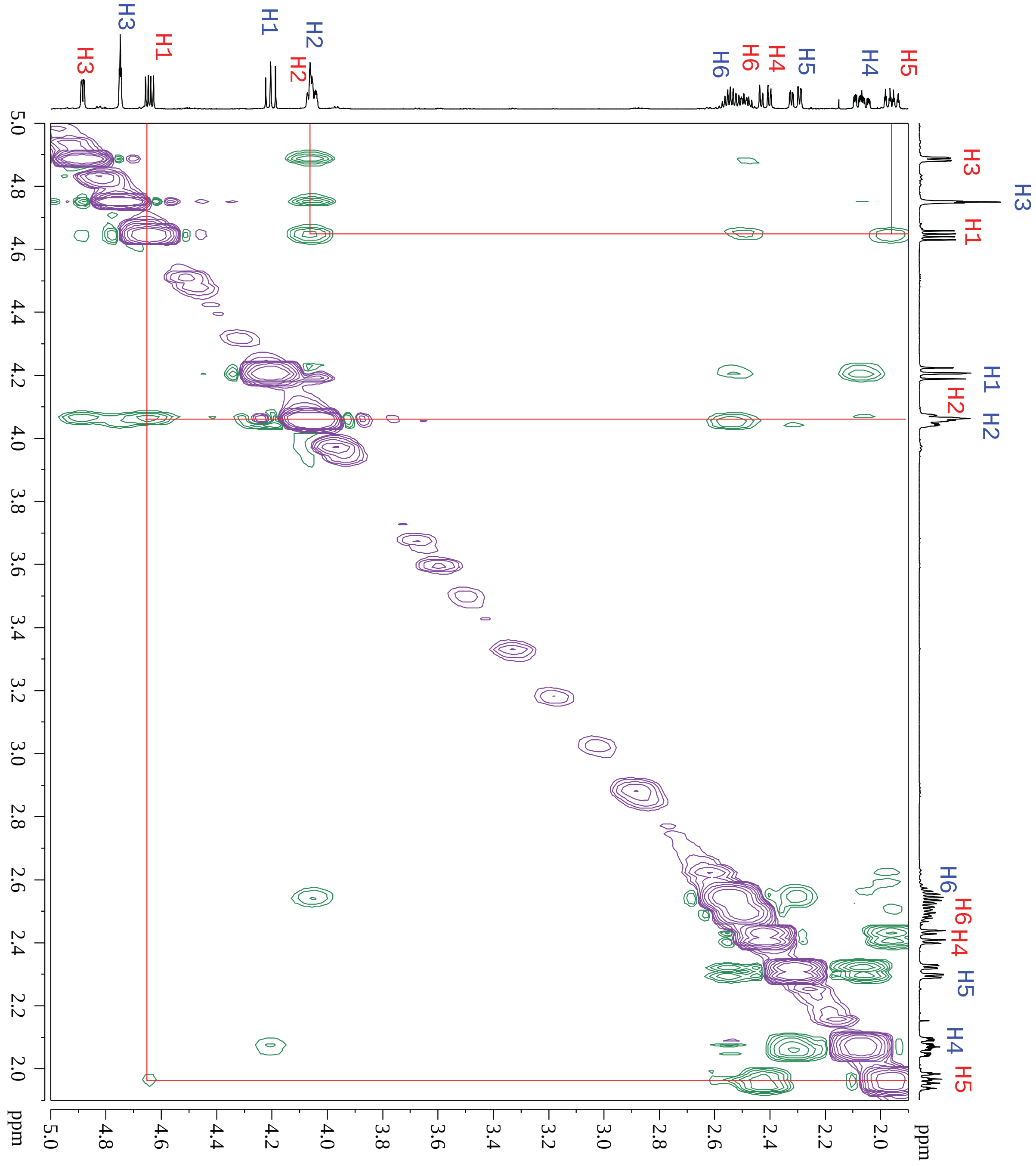

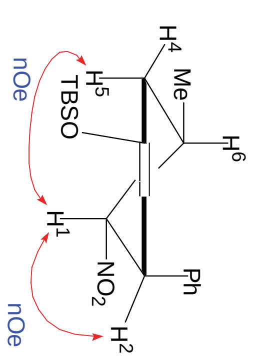

endo-65

Supplementary Figure 240. NOESY NMR spectrum of mixed compound endo/exo-65 highlighting the correlation for endo-65.

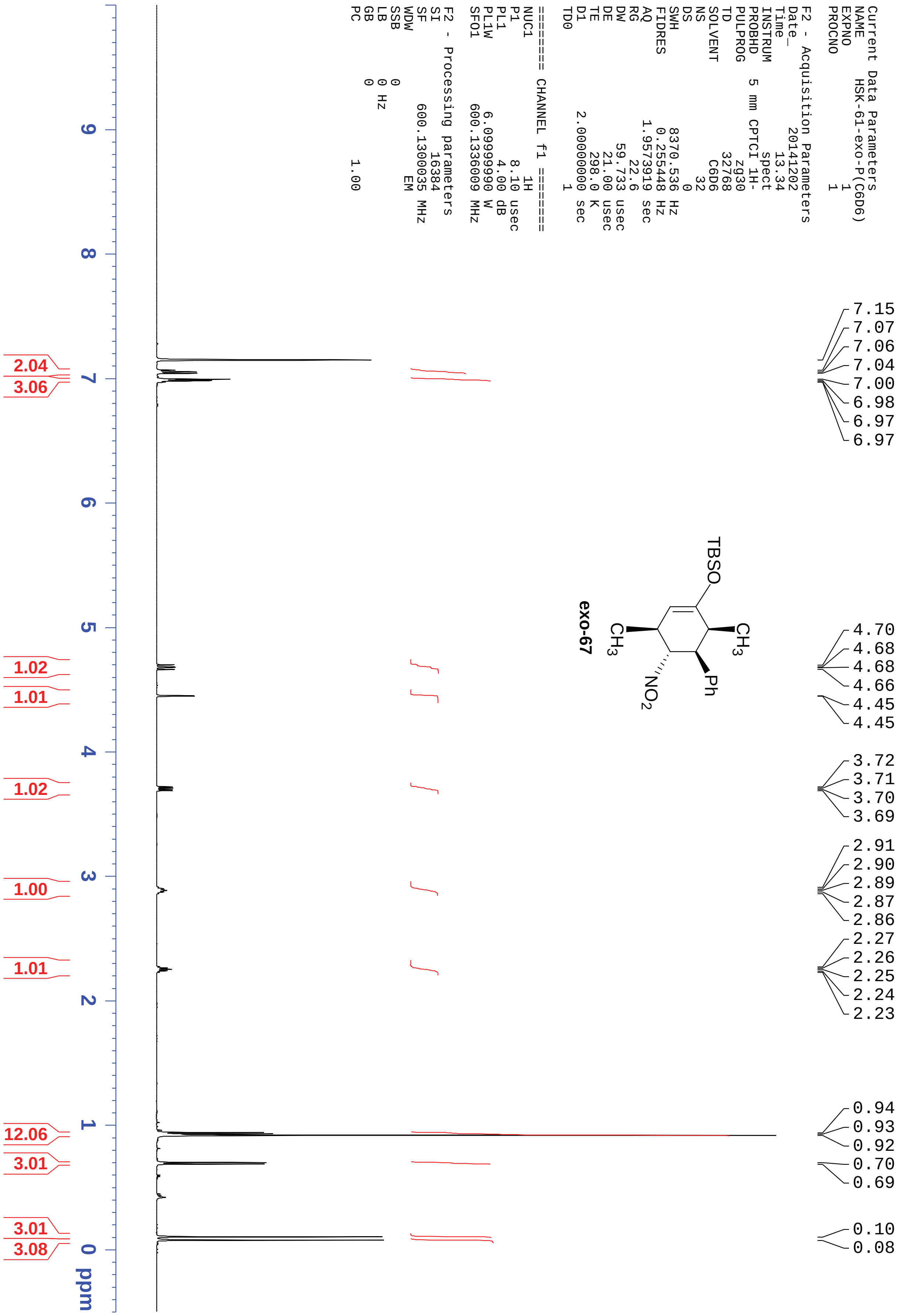

Supplementary Figure 241. ^{1}**H NMR spectrum of compound exo-67.**

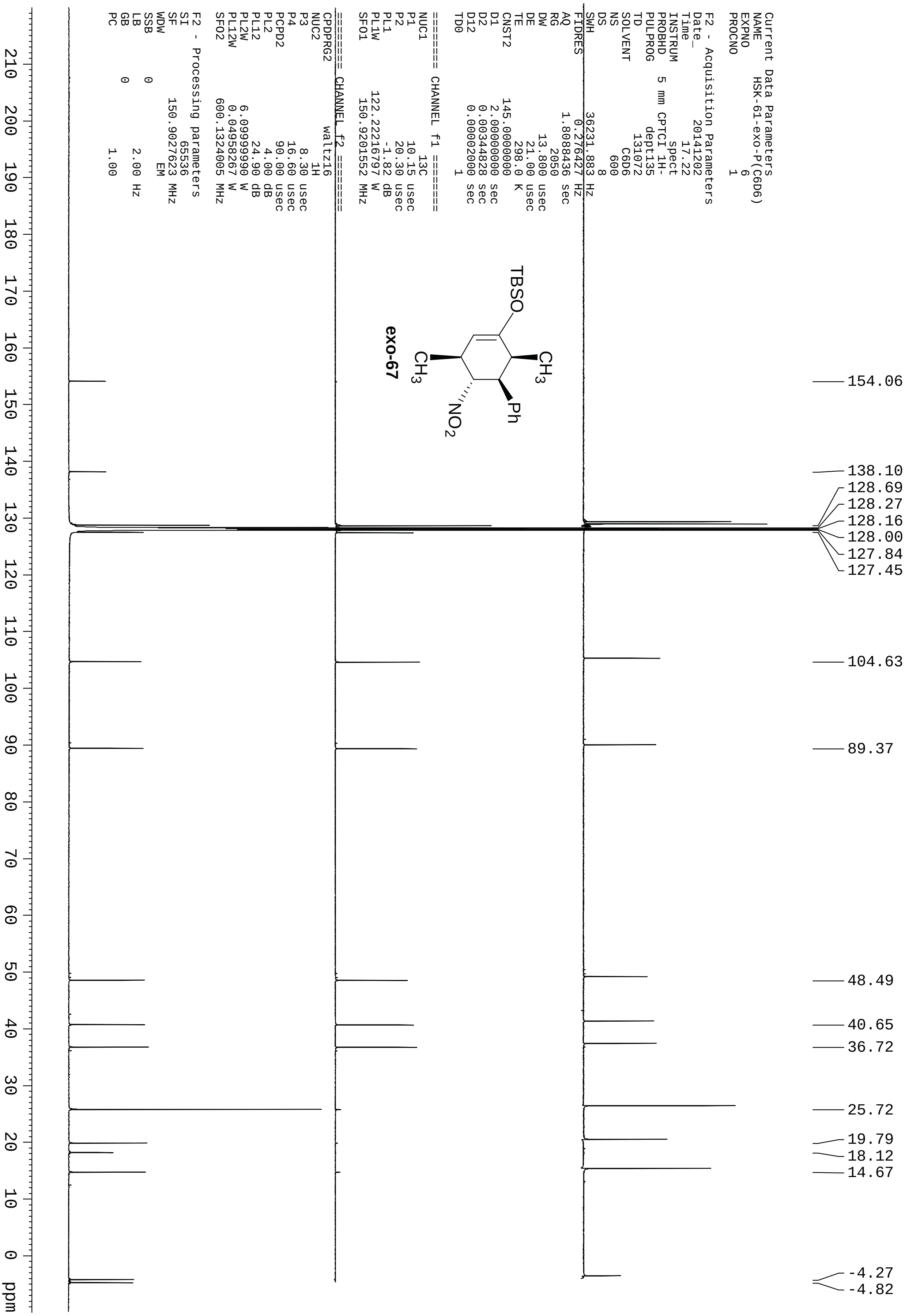

Supplementary Figure 242. ^{13}C and DEPT NMR spectra of compound exo-67.

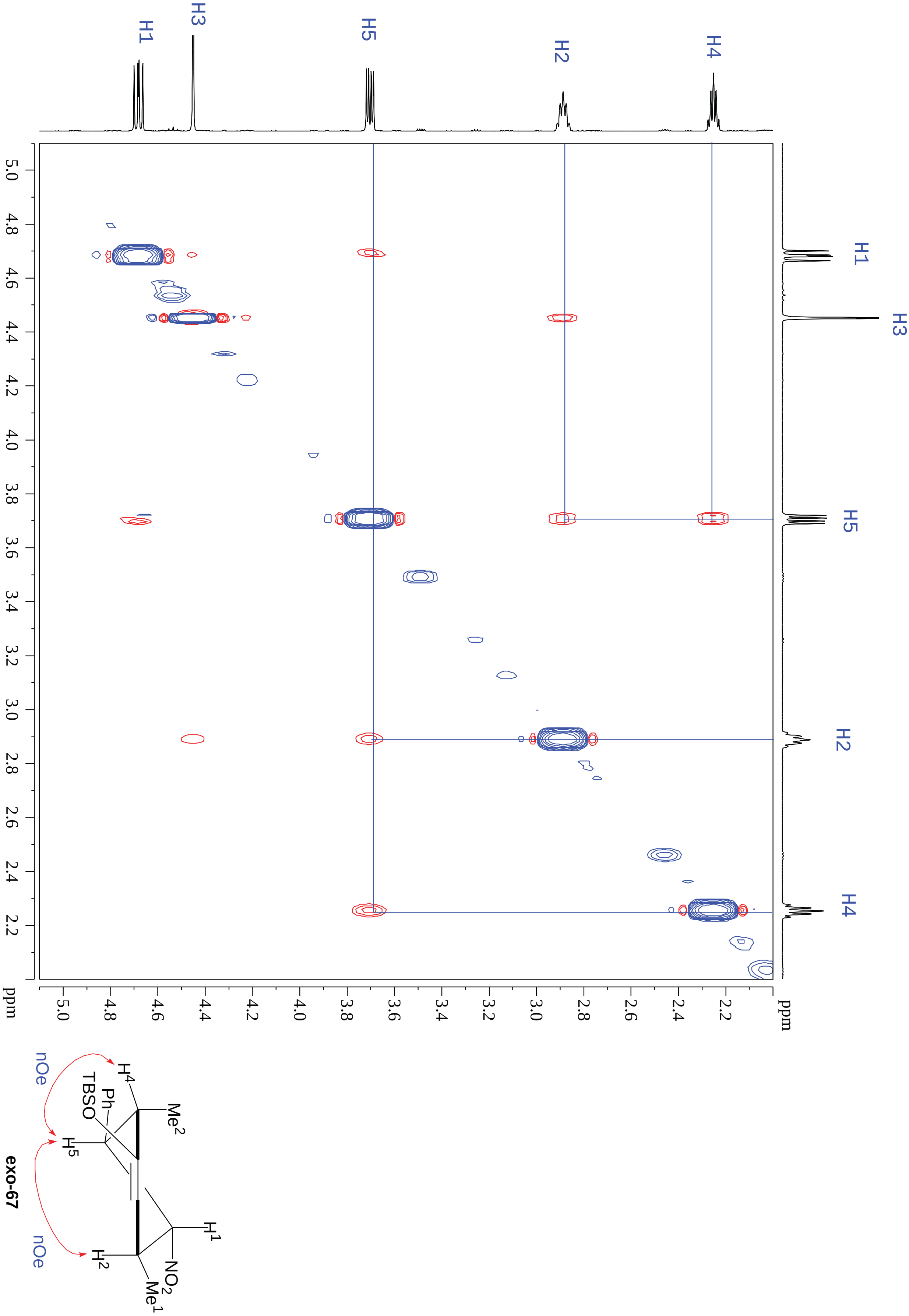

Supplementary Figure 243. NOESY NMR spectrum of compound exo-67.

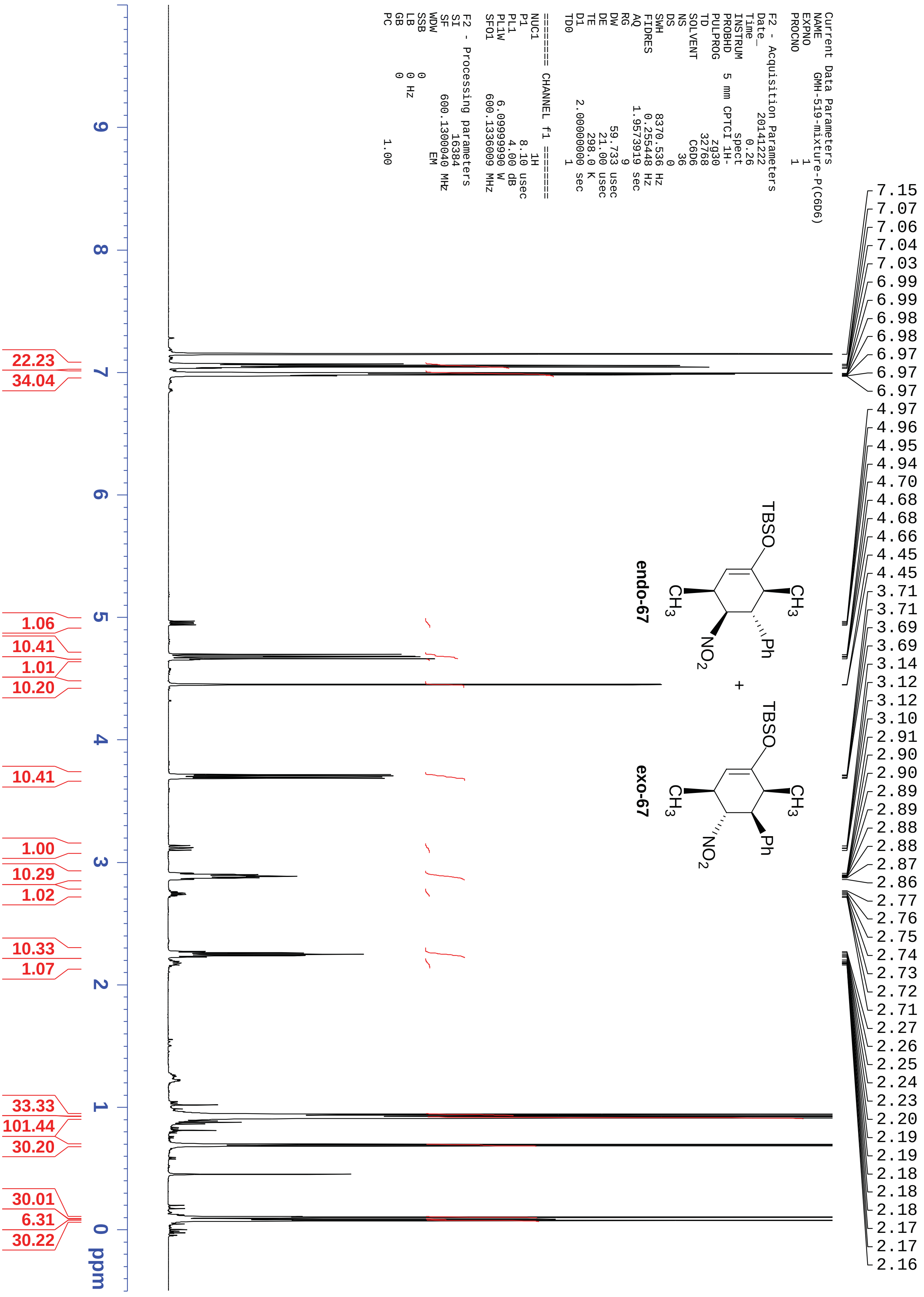

Supplementary Figure 244. ^{1}**H NMR spectrum of mixed compound endo/exo-67.**

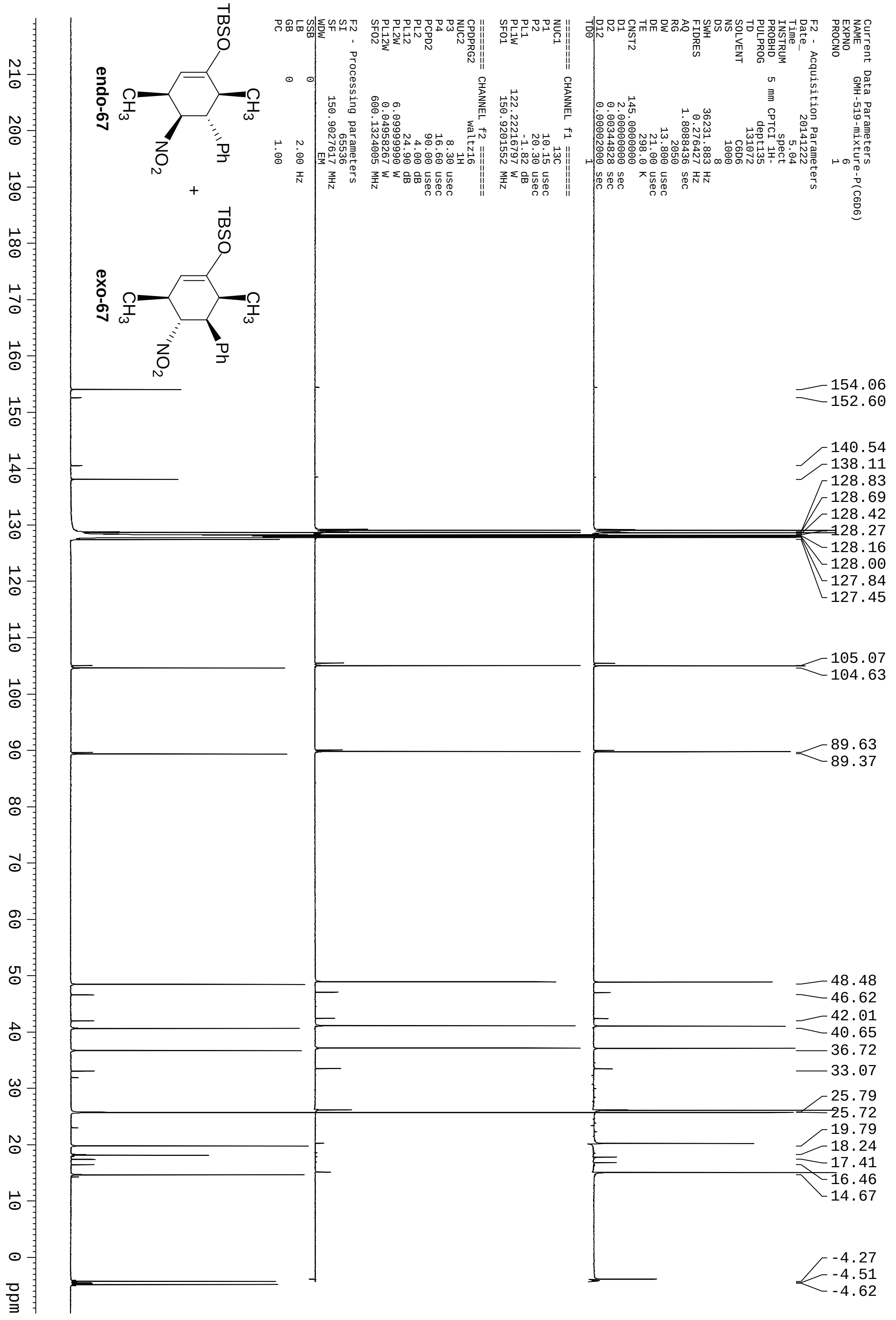

Supplementary Figure 245. ^{13}C and DEPT NMR spectra of mixed compound endo/exo-67.

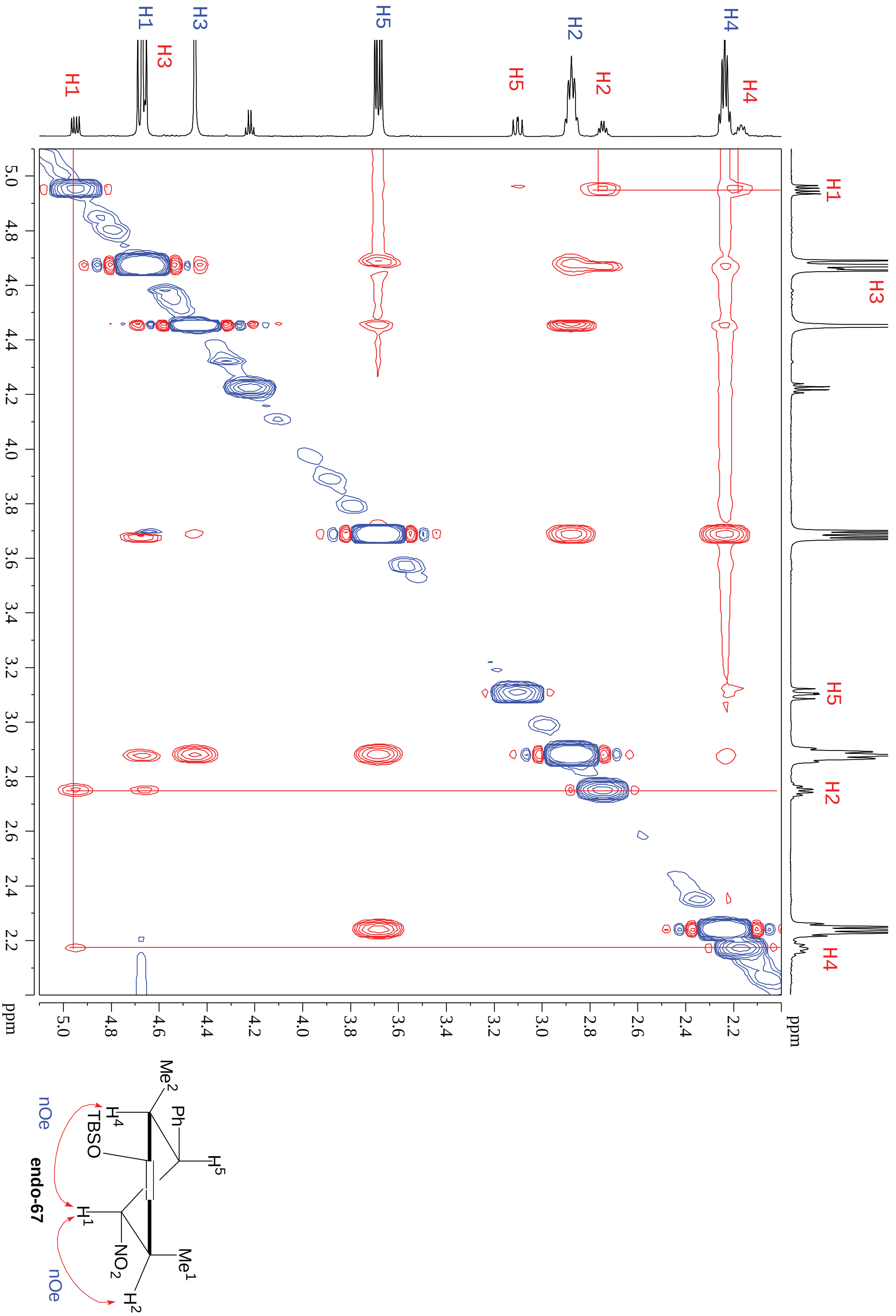

Supplementary Figure 246. NOESY NMR spectrum of mixed compound endo/exo-67 highlighting the correlation for endo-67.

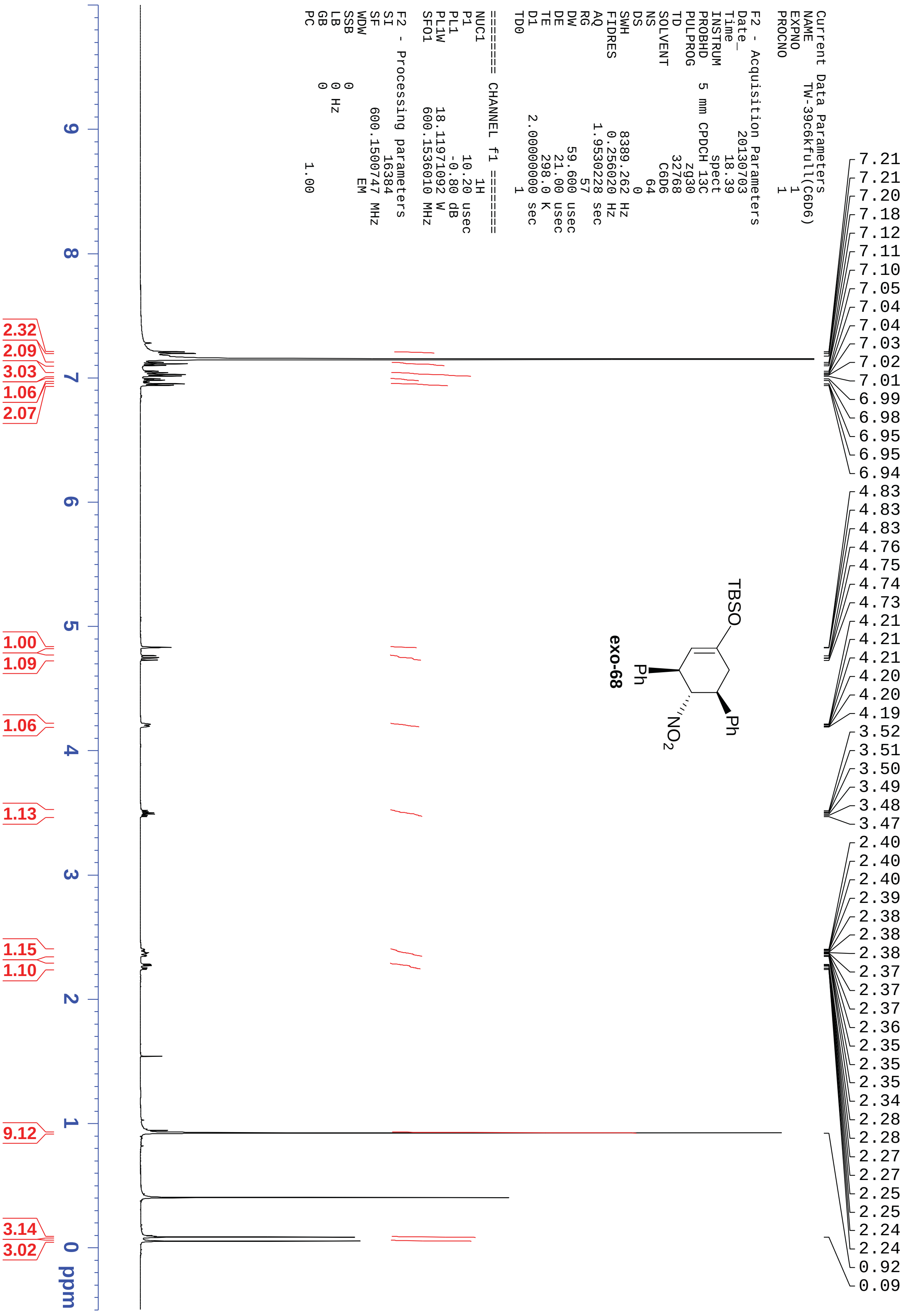

Supplementary Figure 247. ^{1}H NMR spectrum of compound exo-68.

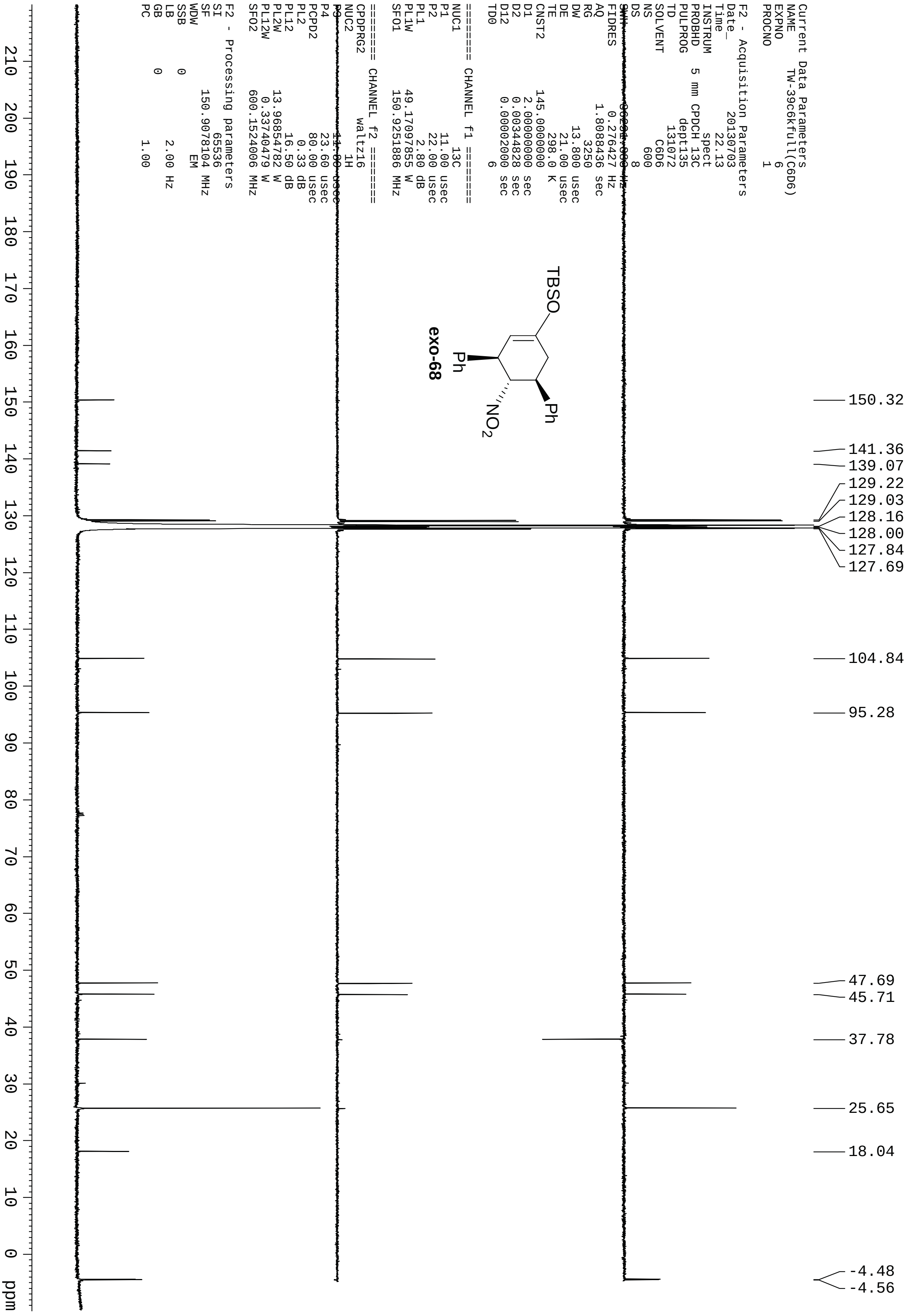

Supplementary Figure 248. ^{13}C and DEPT NMR spectra of compound exo-68.

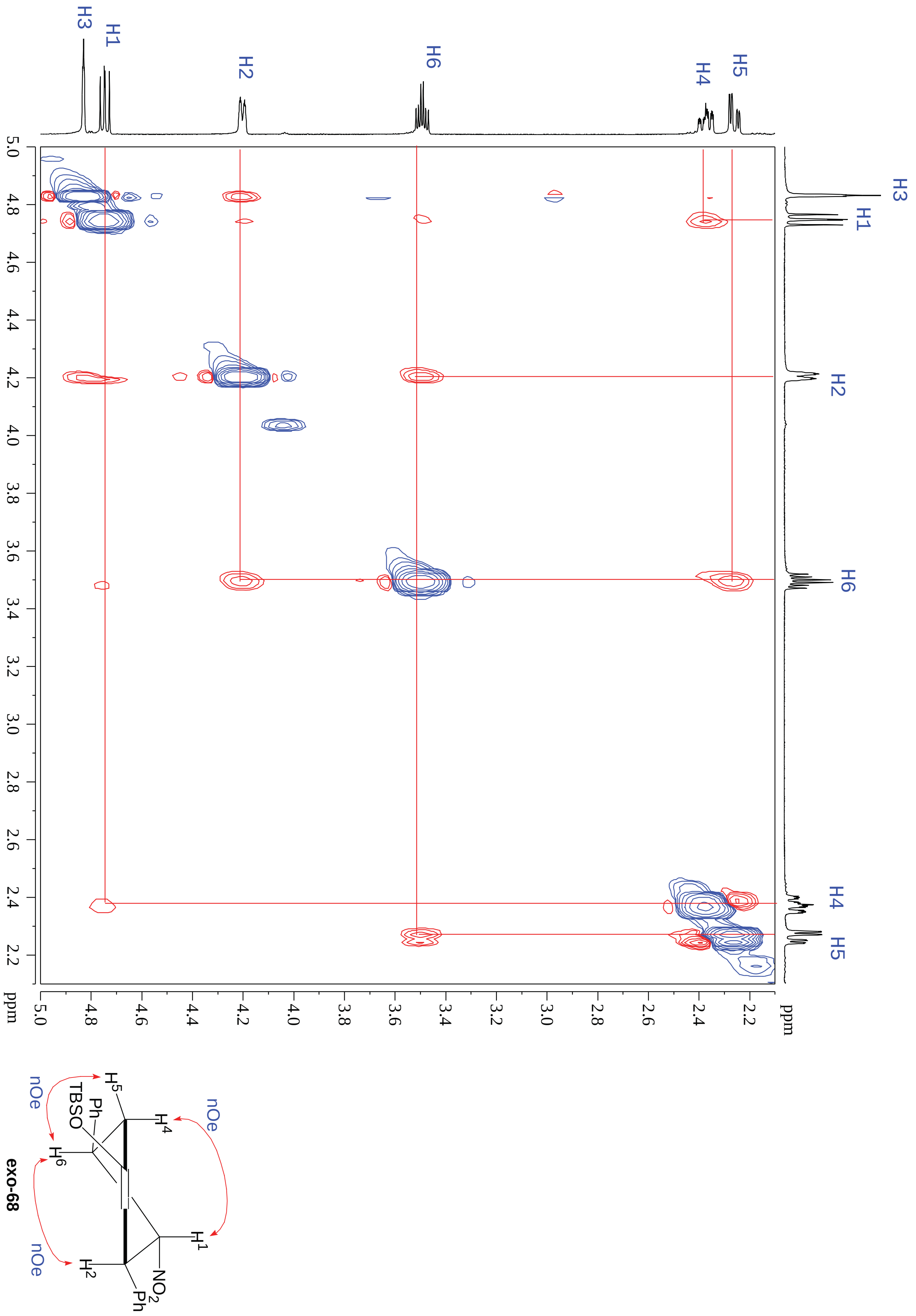

Supplementary Figure 249. NOESY NMR spectrum of compound exo-68.

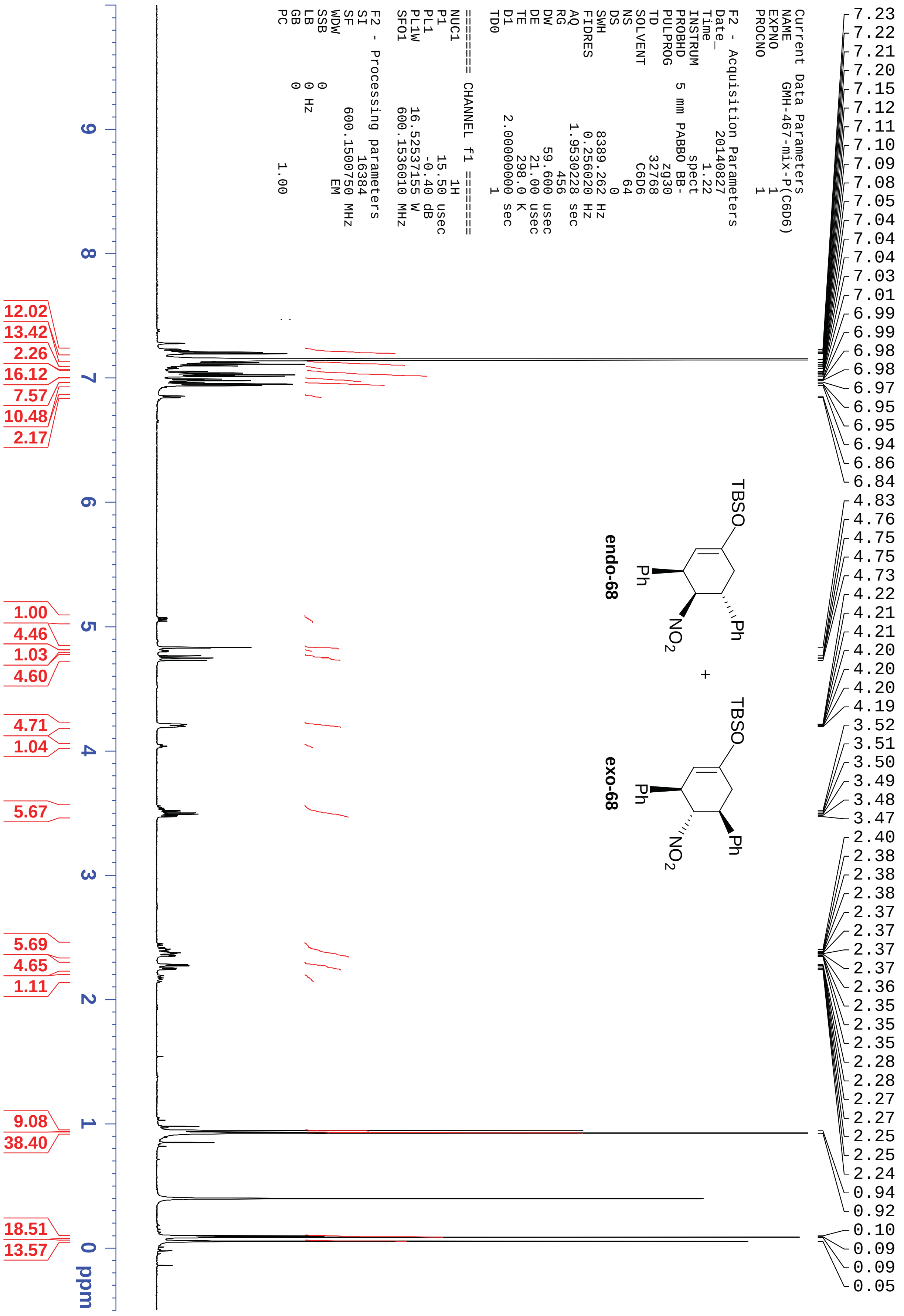

Supplementary Figure 250. ^{1}**H NMR spectrum of mixed compound endo/exo-68.**

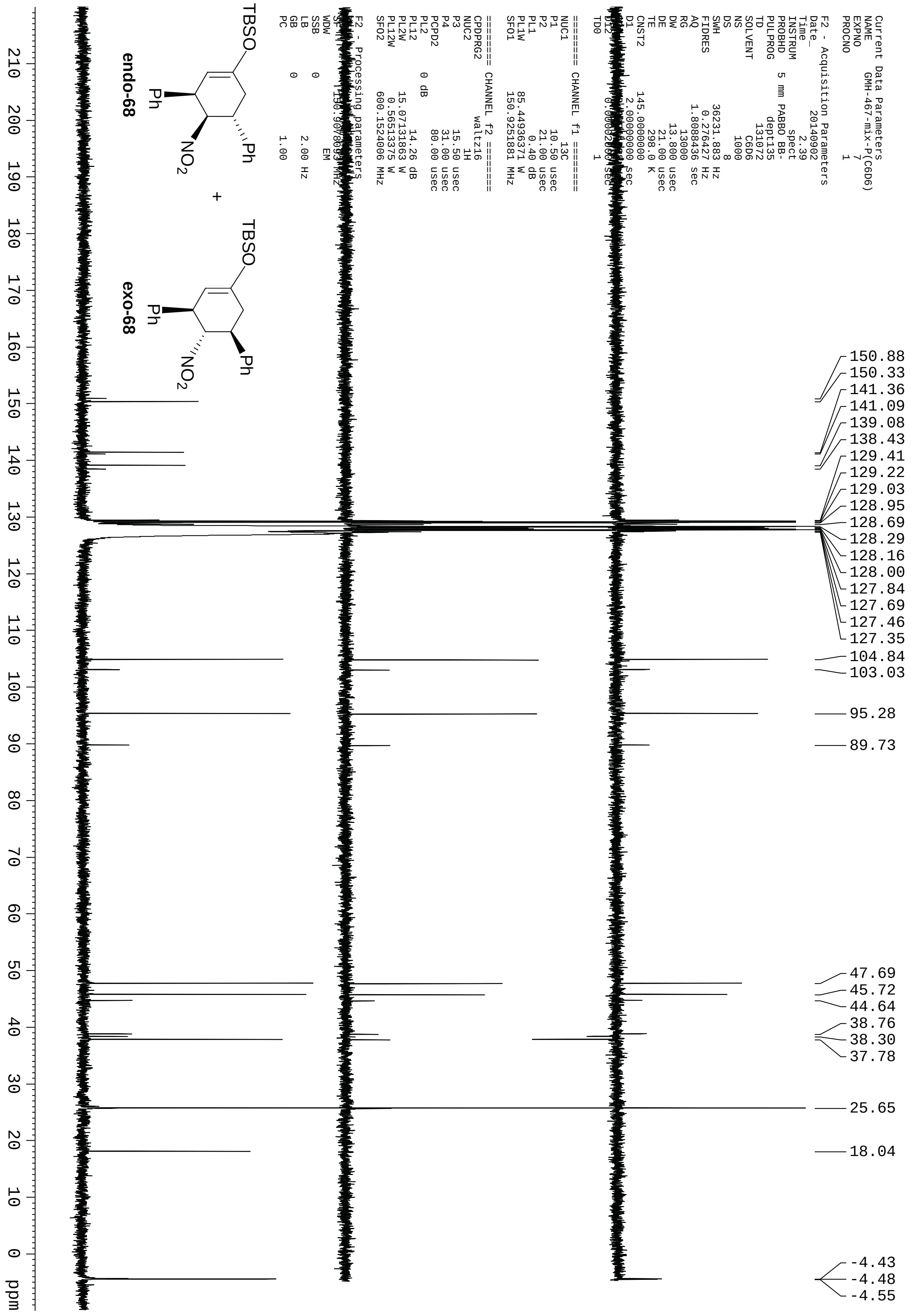

Supplementary Figure 251. ^{13}C and DEPT NMR spectra of mixed compound endo/exo-68.

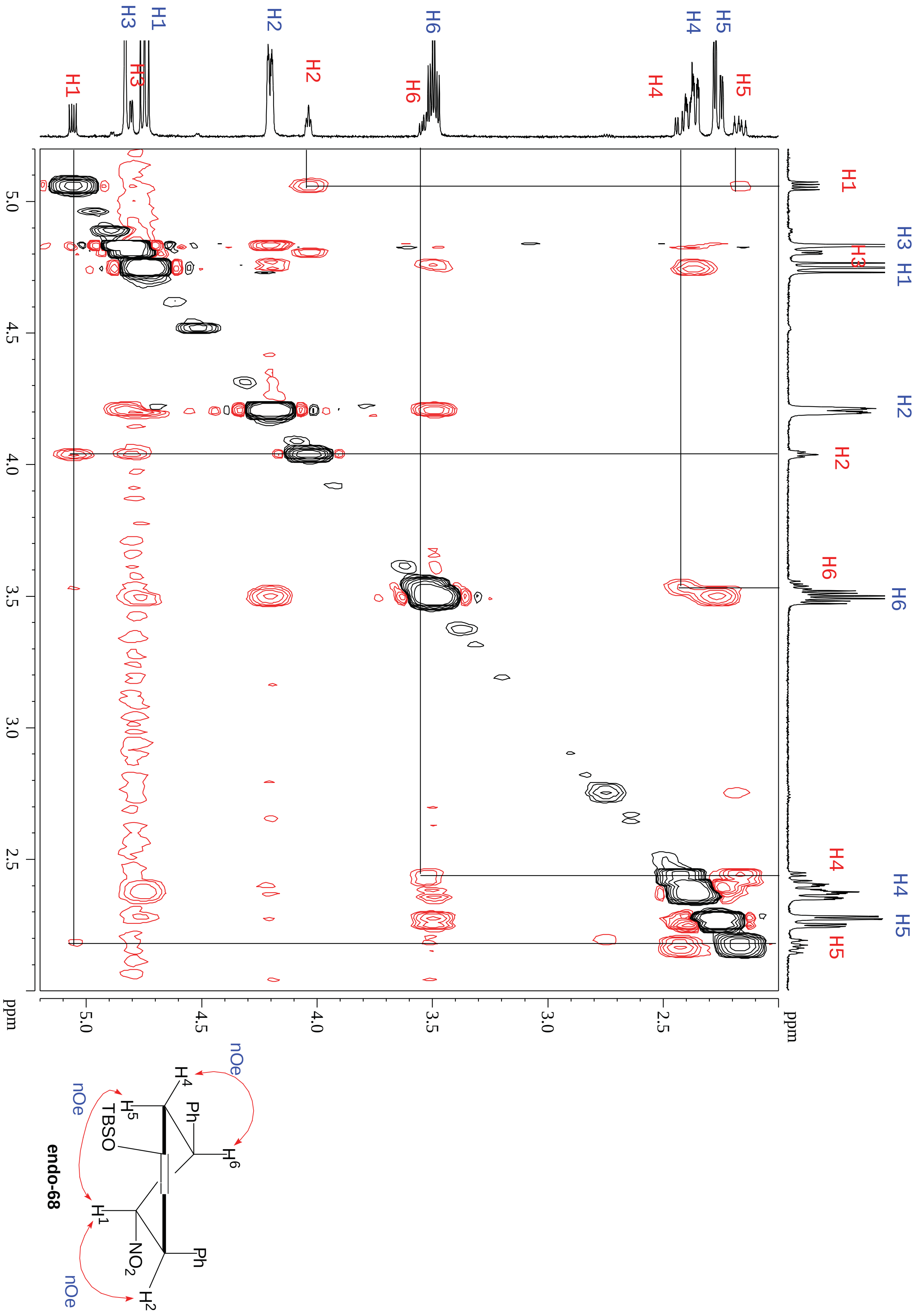

Supplementary Figure 252. NOESY NMR spectrum of mixed compound endo/exo-68 highlighting the correlation for endo-68.

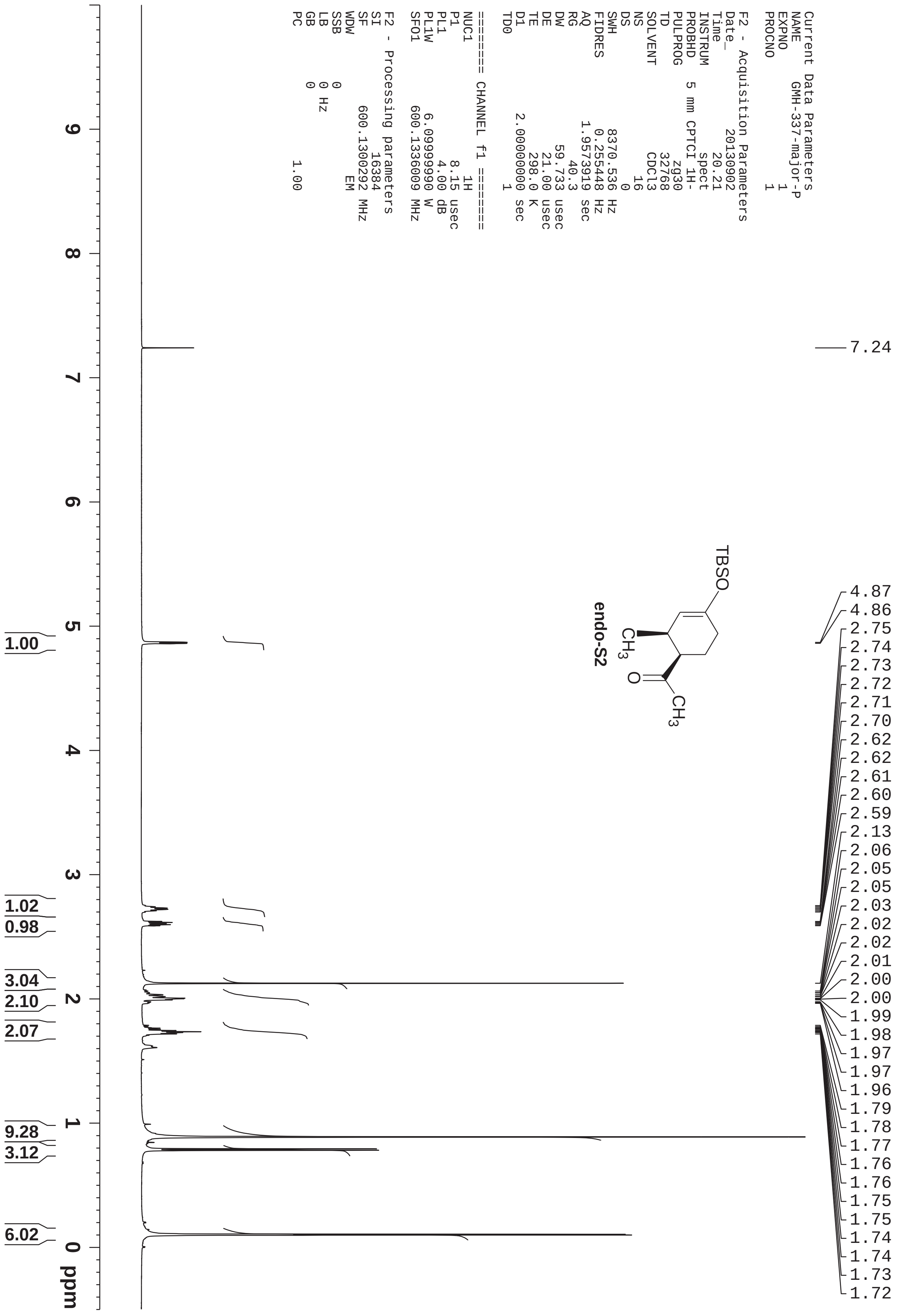

Supplementary Figure 253. ^{1}H NMR spectrum of compound endo-S2.

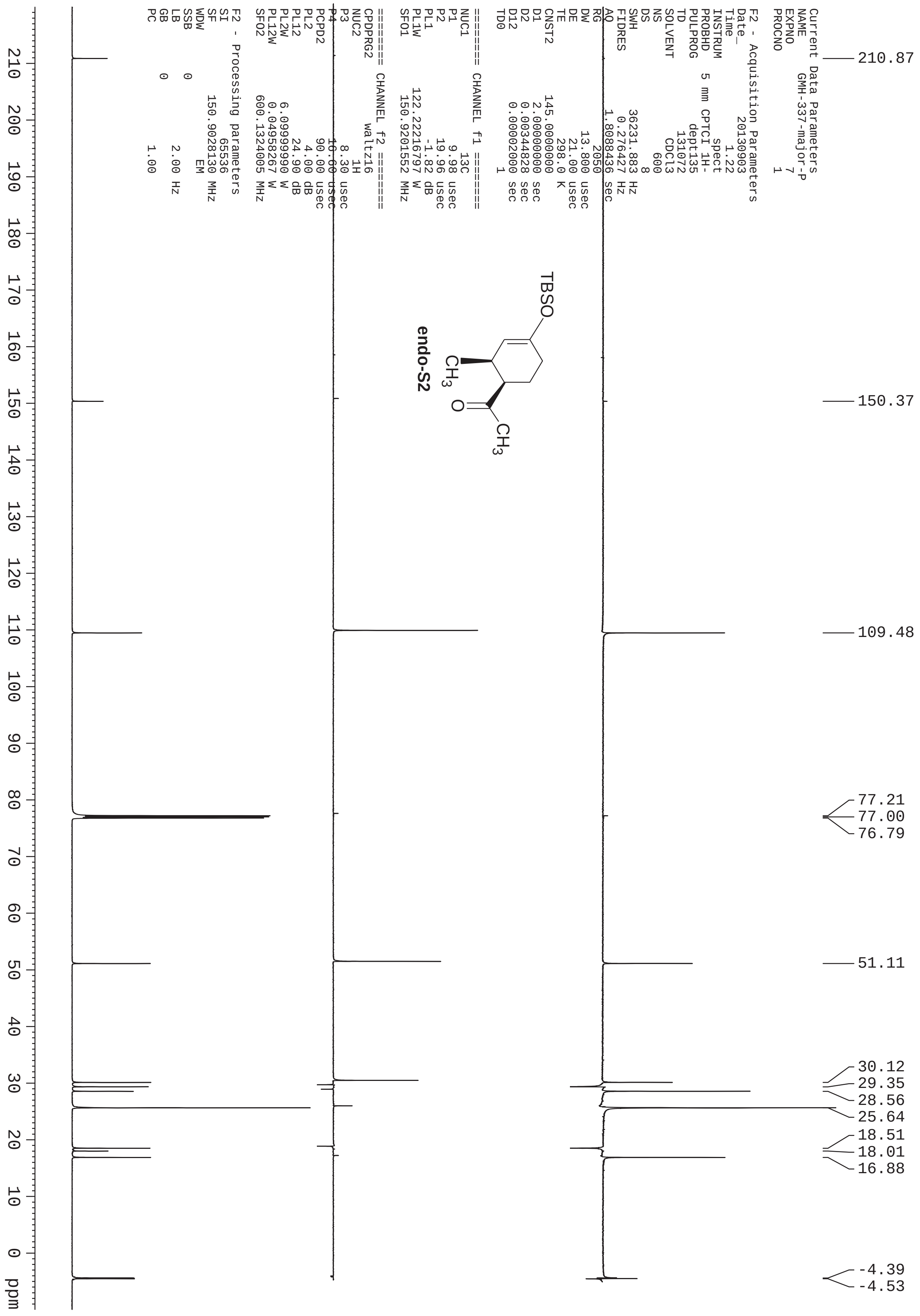

Supplementary Figure 254. ^{13}C and DEPT NMR spectra of compound endo-S2.

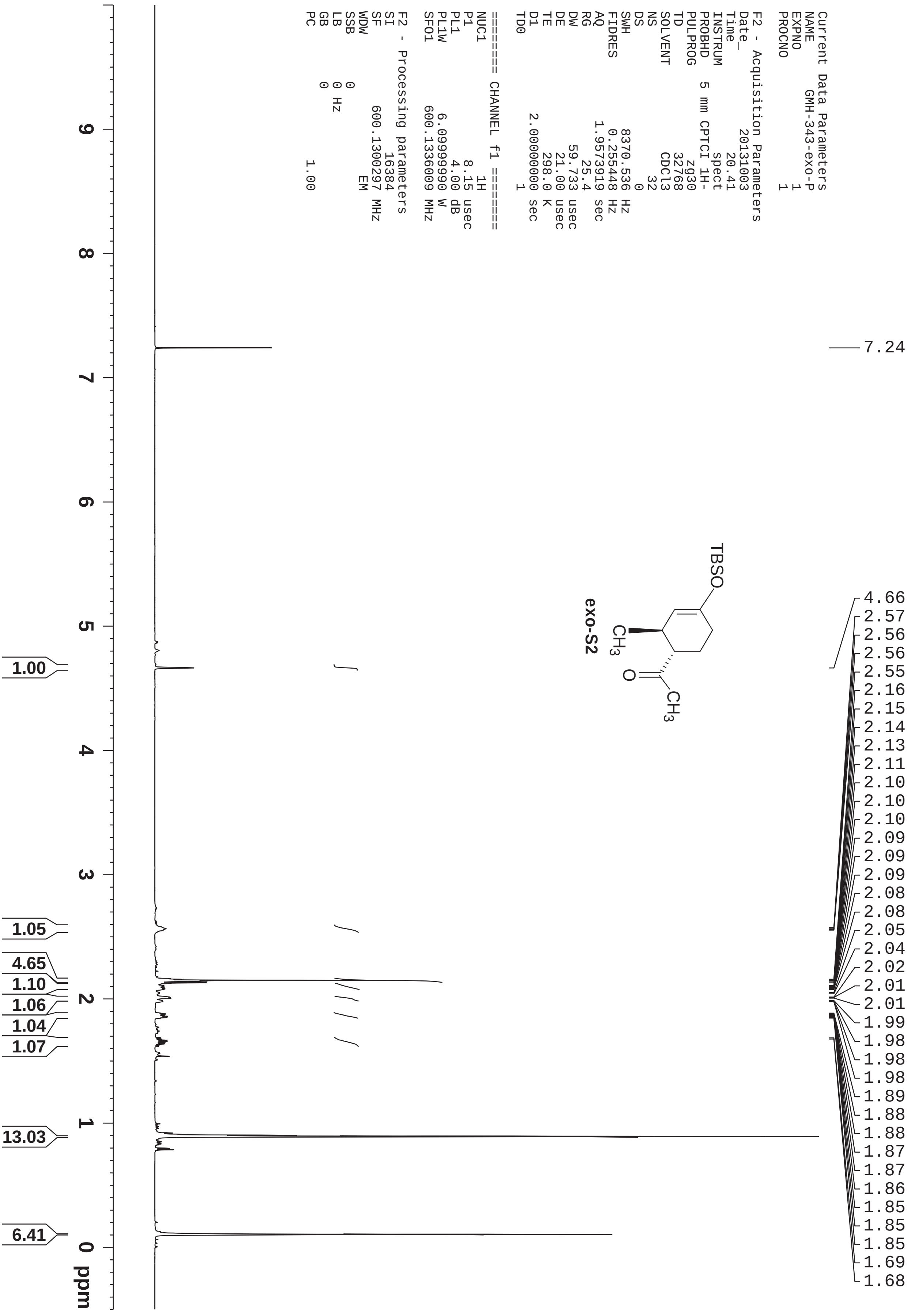

Supplementary Figure 255. ^{1}H NMR spectrum of compound exo-S2.

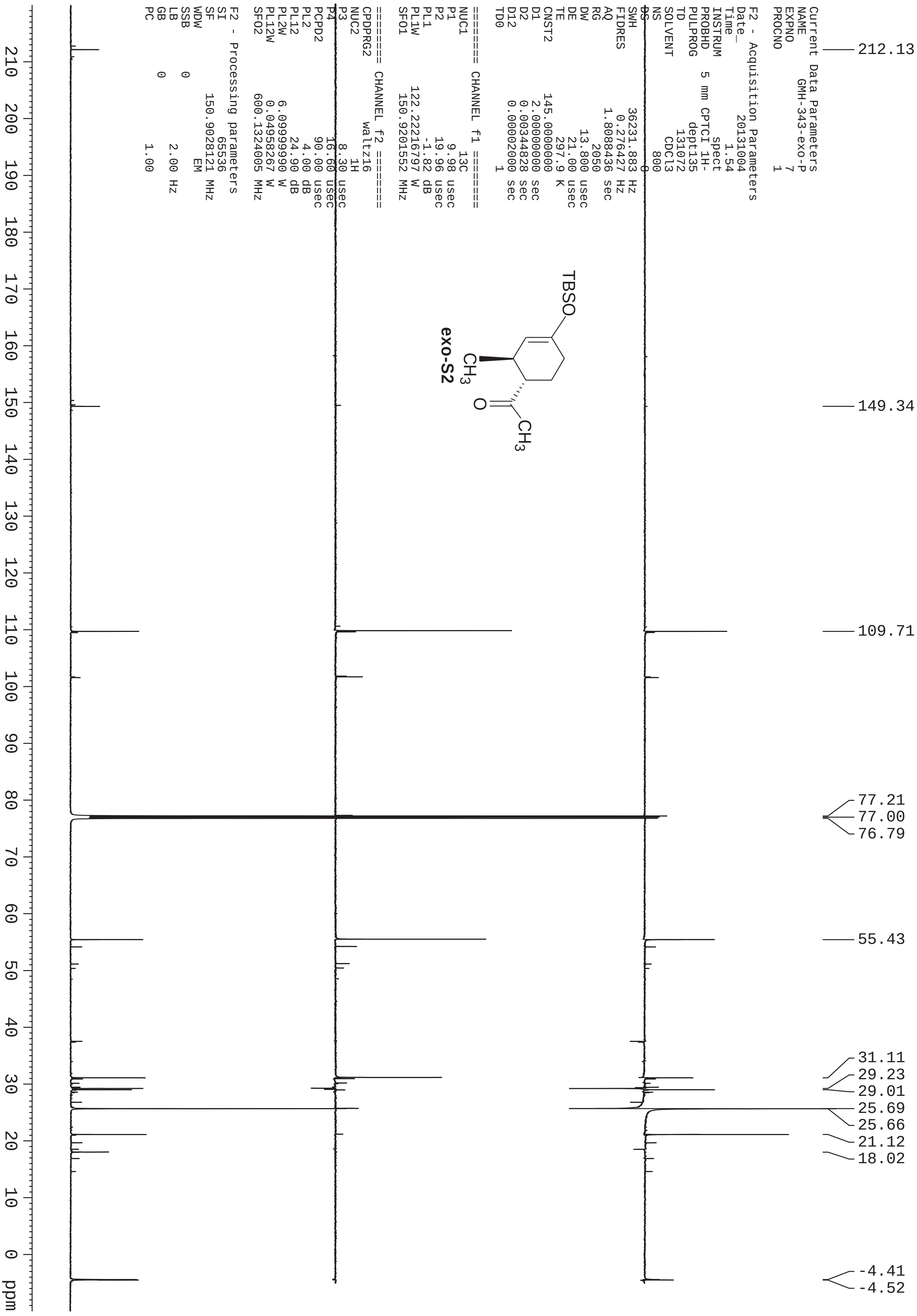

Supplementary Figure 256. ^{13}C and DEPT NMR spectra of compound exo-S2.

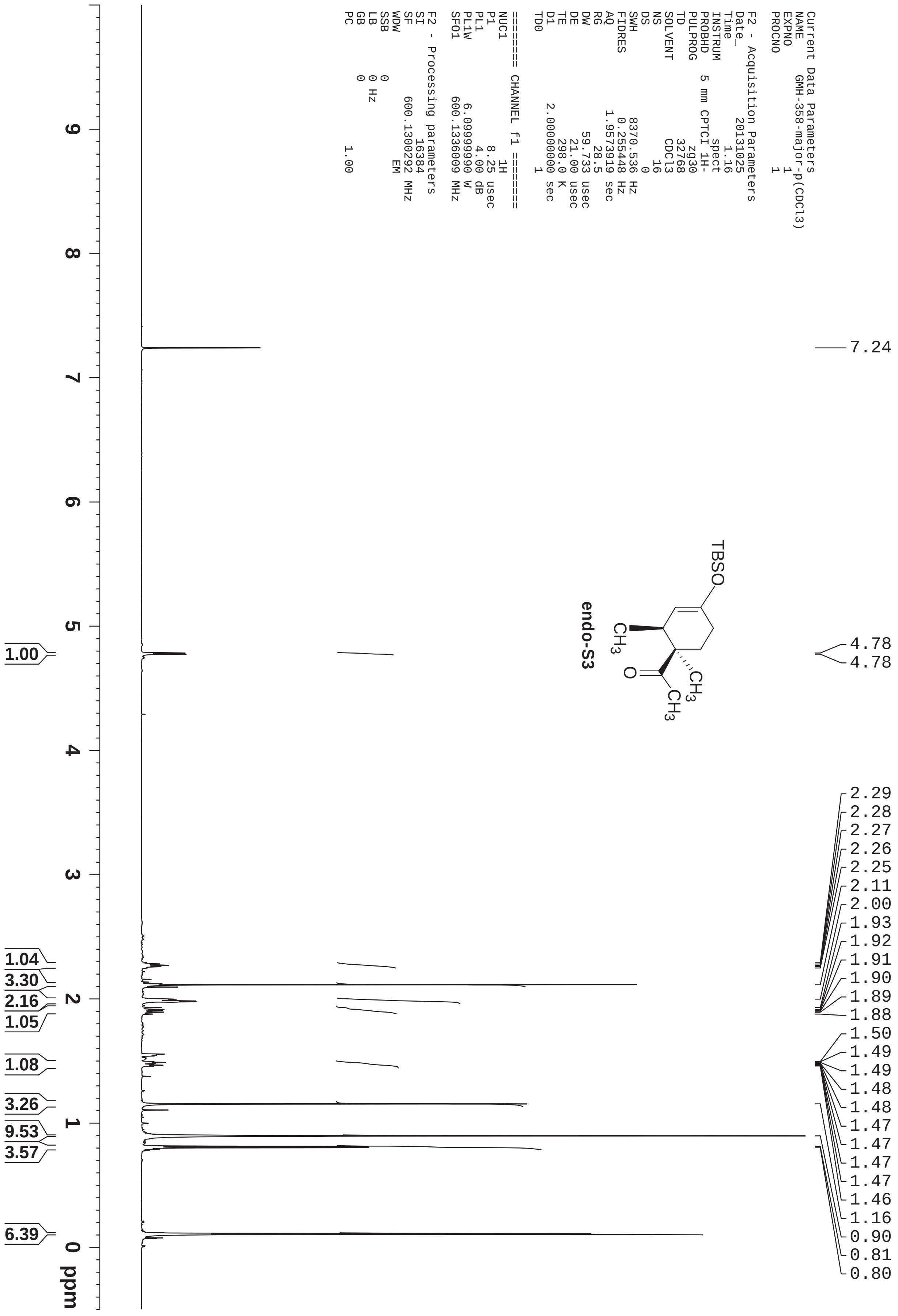

Supplementary Figure 257. ^{1}H NMR spectrum of compound endo-S3.

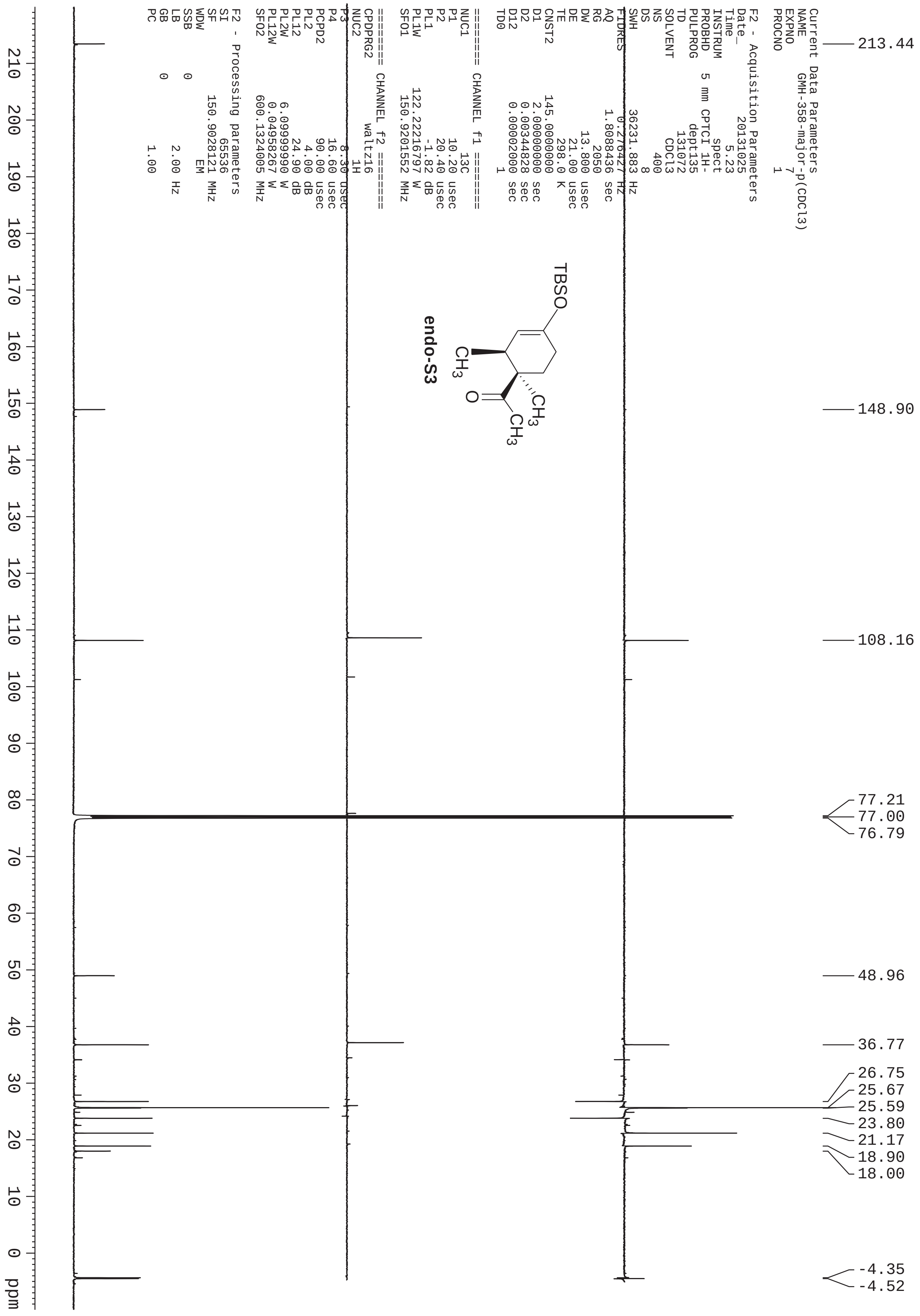

Supplementary Figure 258. ^{13}C and DEPT NMR spectra of compound endo-S3.

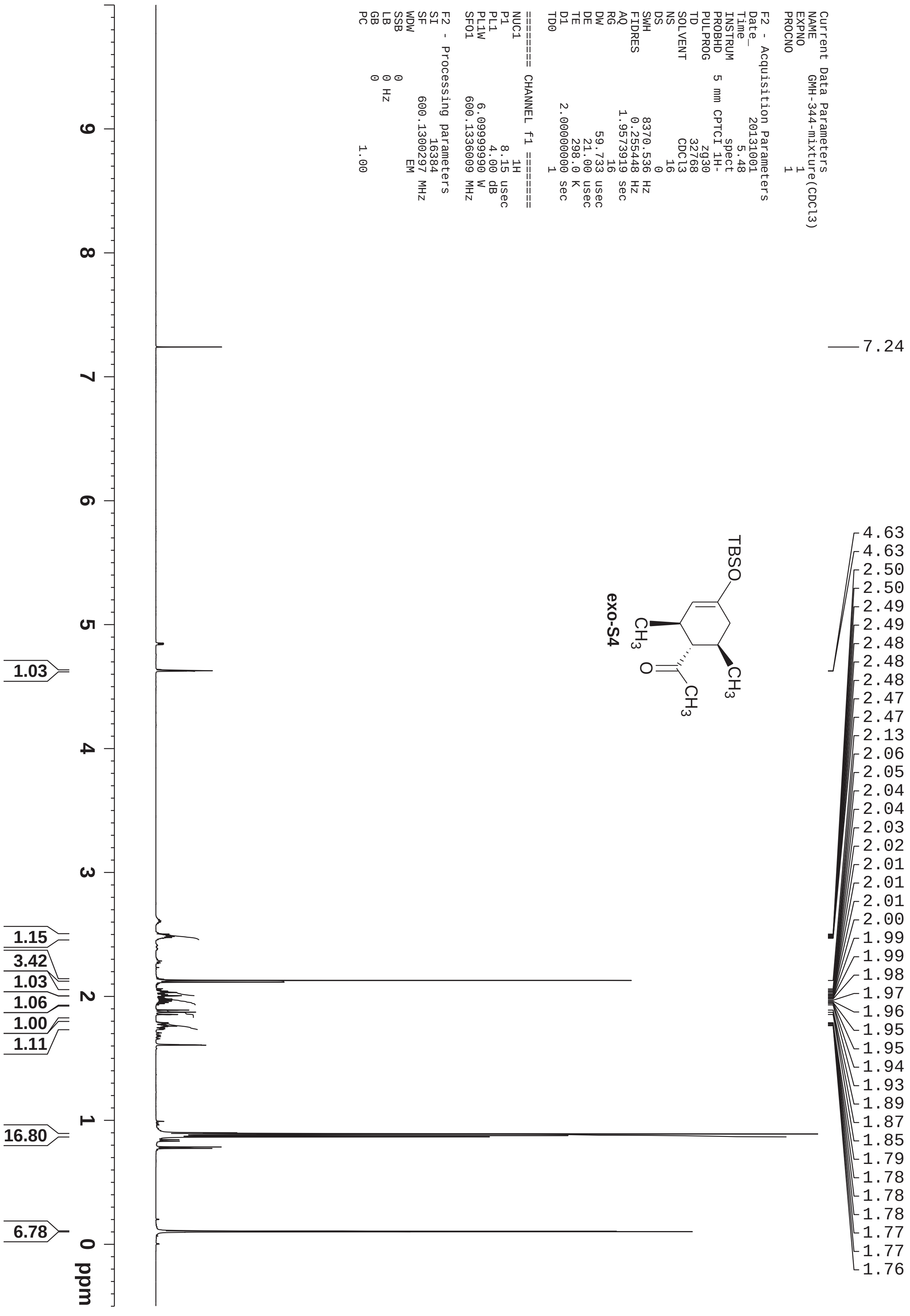

Supplementary Figure 259. ^{1}H NMR spectrum of compound exo-S4.

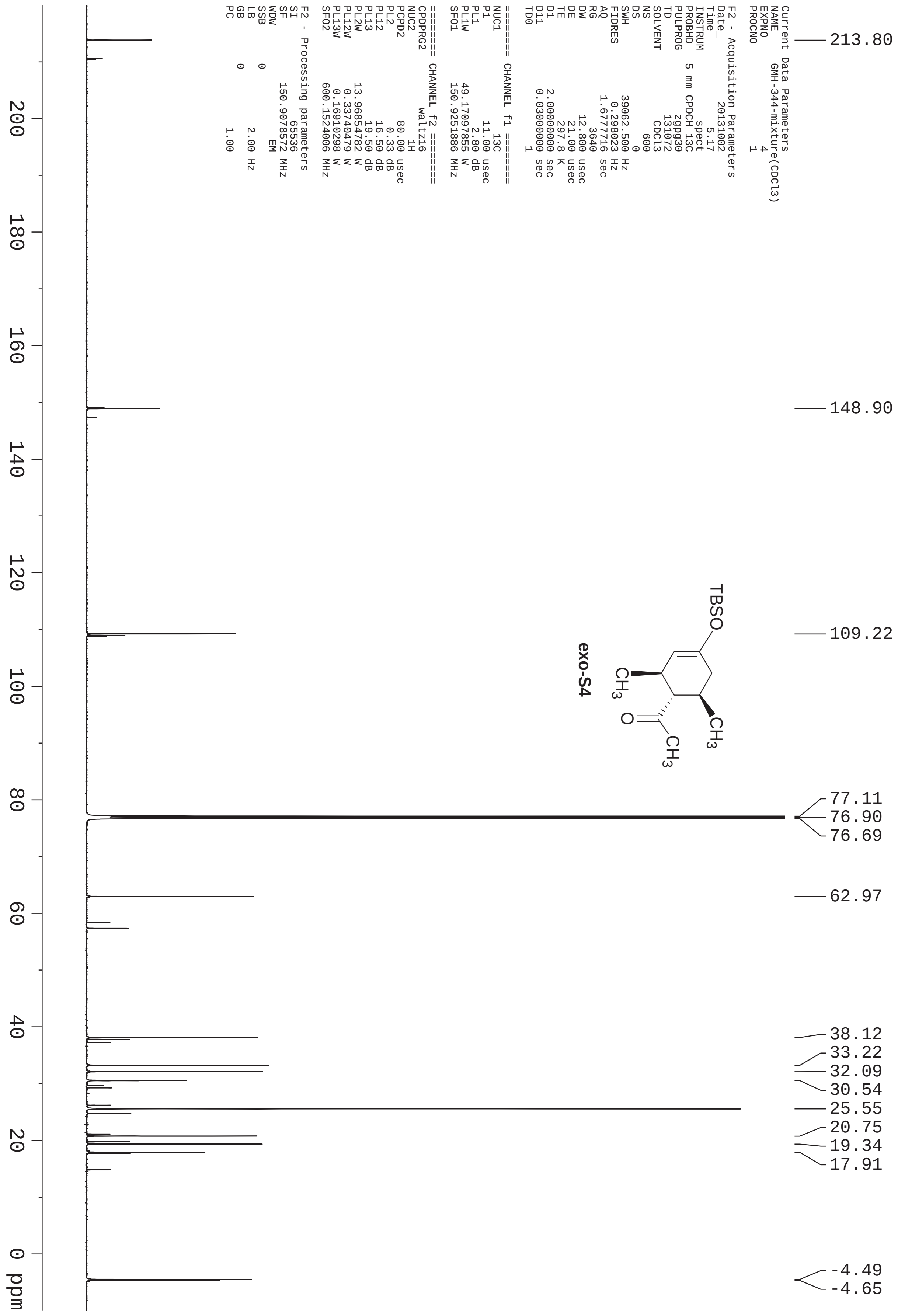

Supplementary Figure 260. ^{13}C NMR spectrum of compound exo-S4.

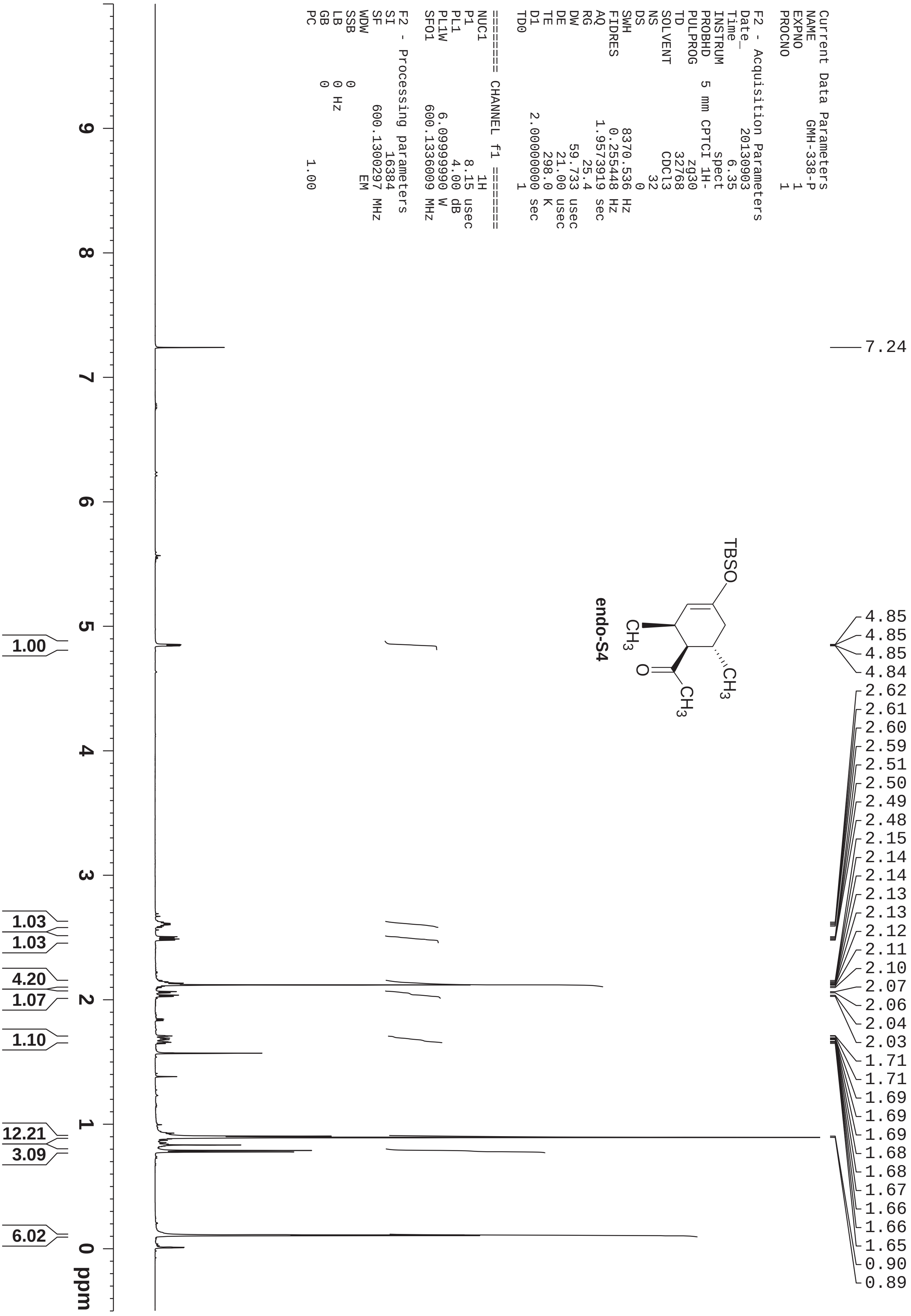

Supplementary Figure 261. ^{1}H NMR spectrum of compound endo-S4.

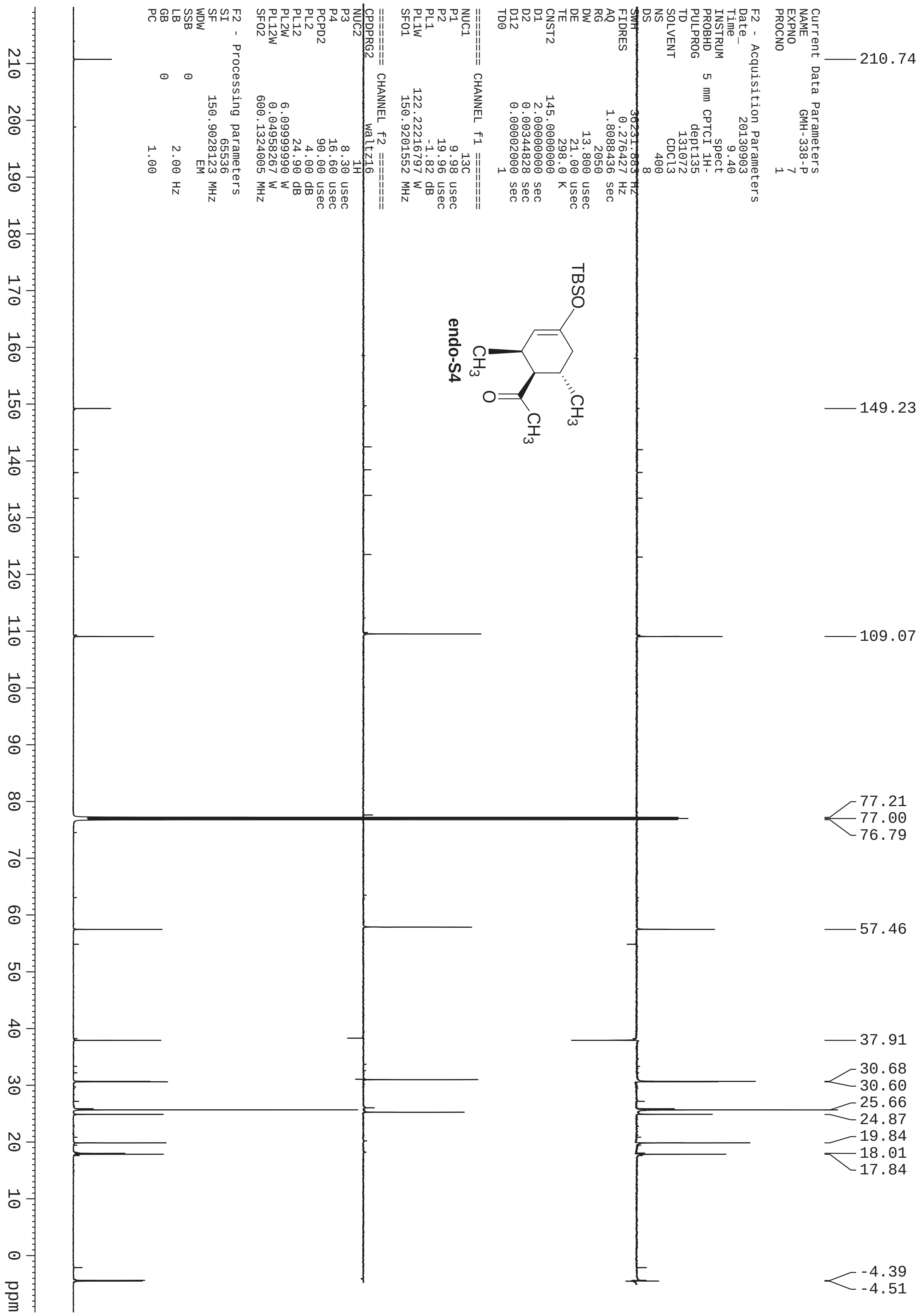

Supplementary Figure 262. ^{13}C and DEPT NMR spectra of compound endo-S4.

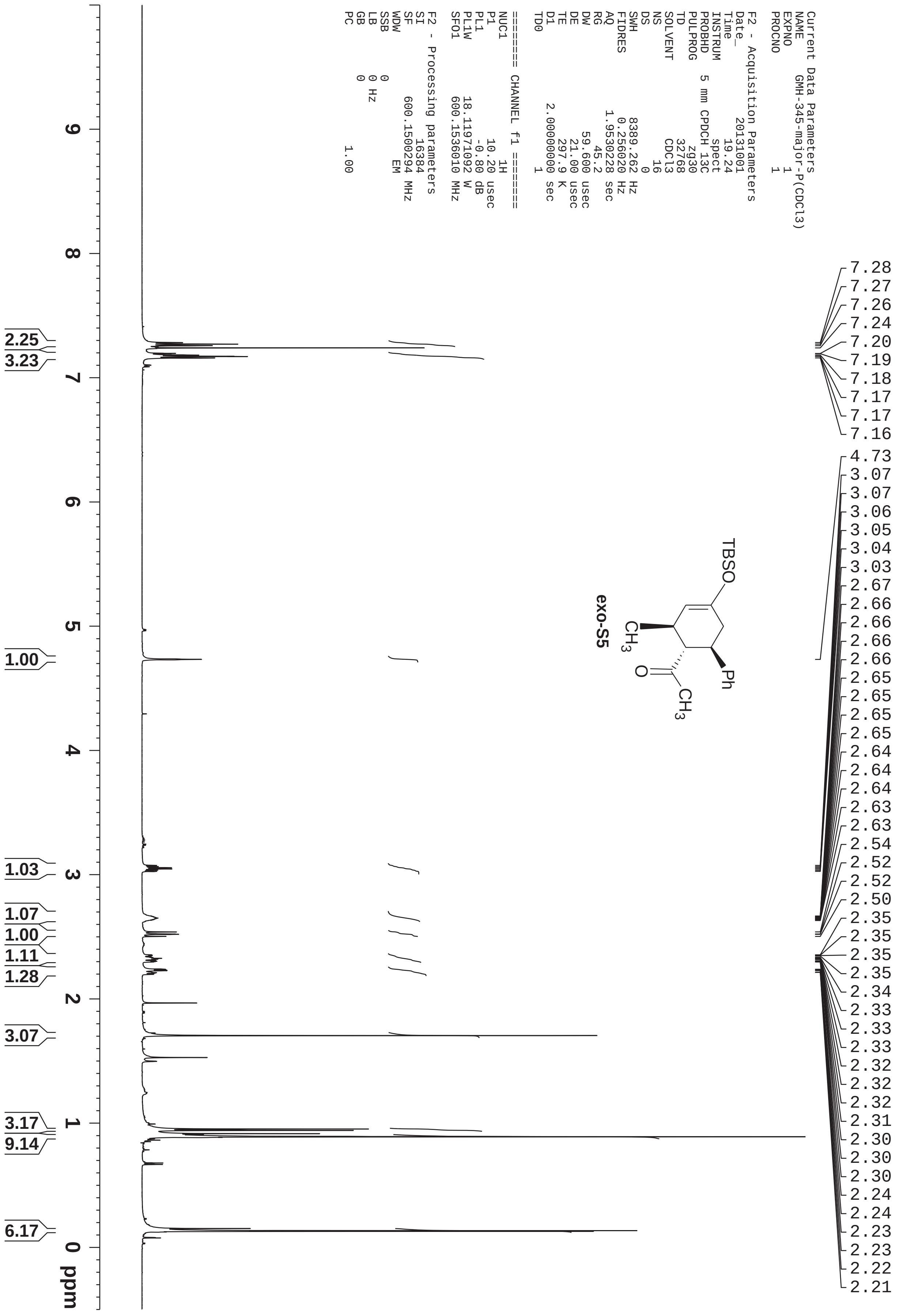

Supplementary Figure 263. 1H NMR spectrum of compound exo-S5.

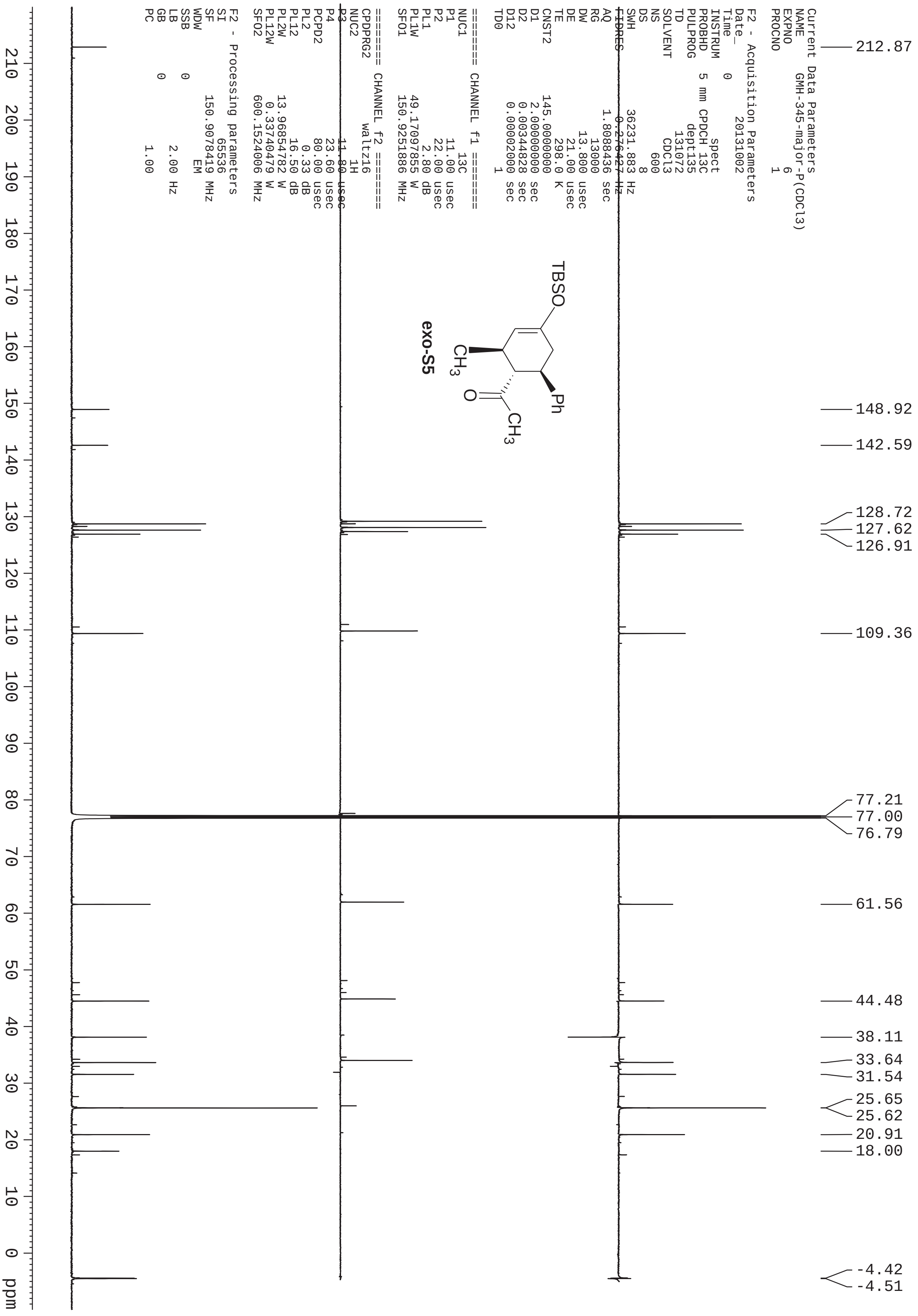

Supplementary Figure 264. ^{13}C and DEPT NMR spectra of compound exo-S5.

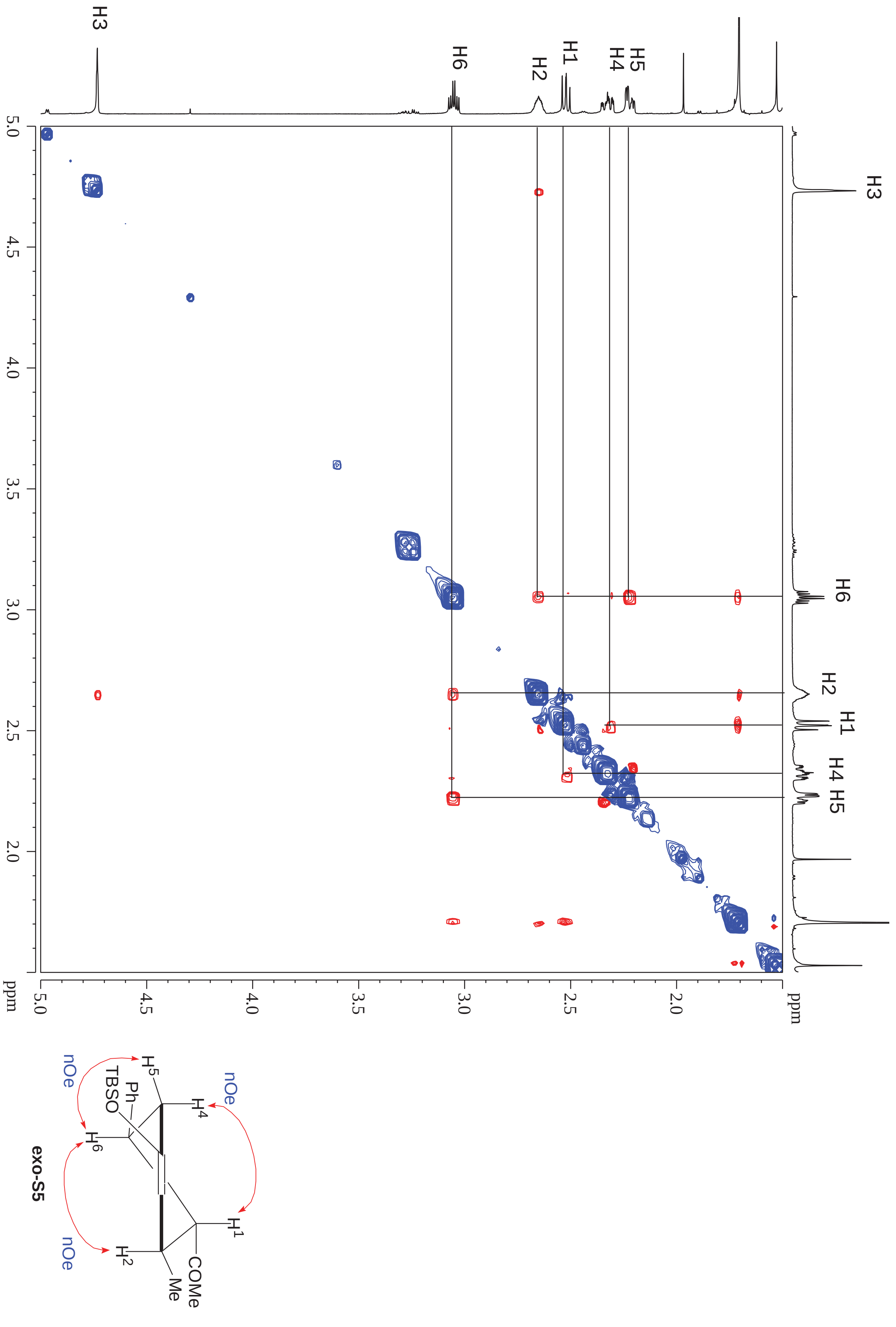

Supplementary Figure 265. NOESY NMR spectrum of compound exo-S5.

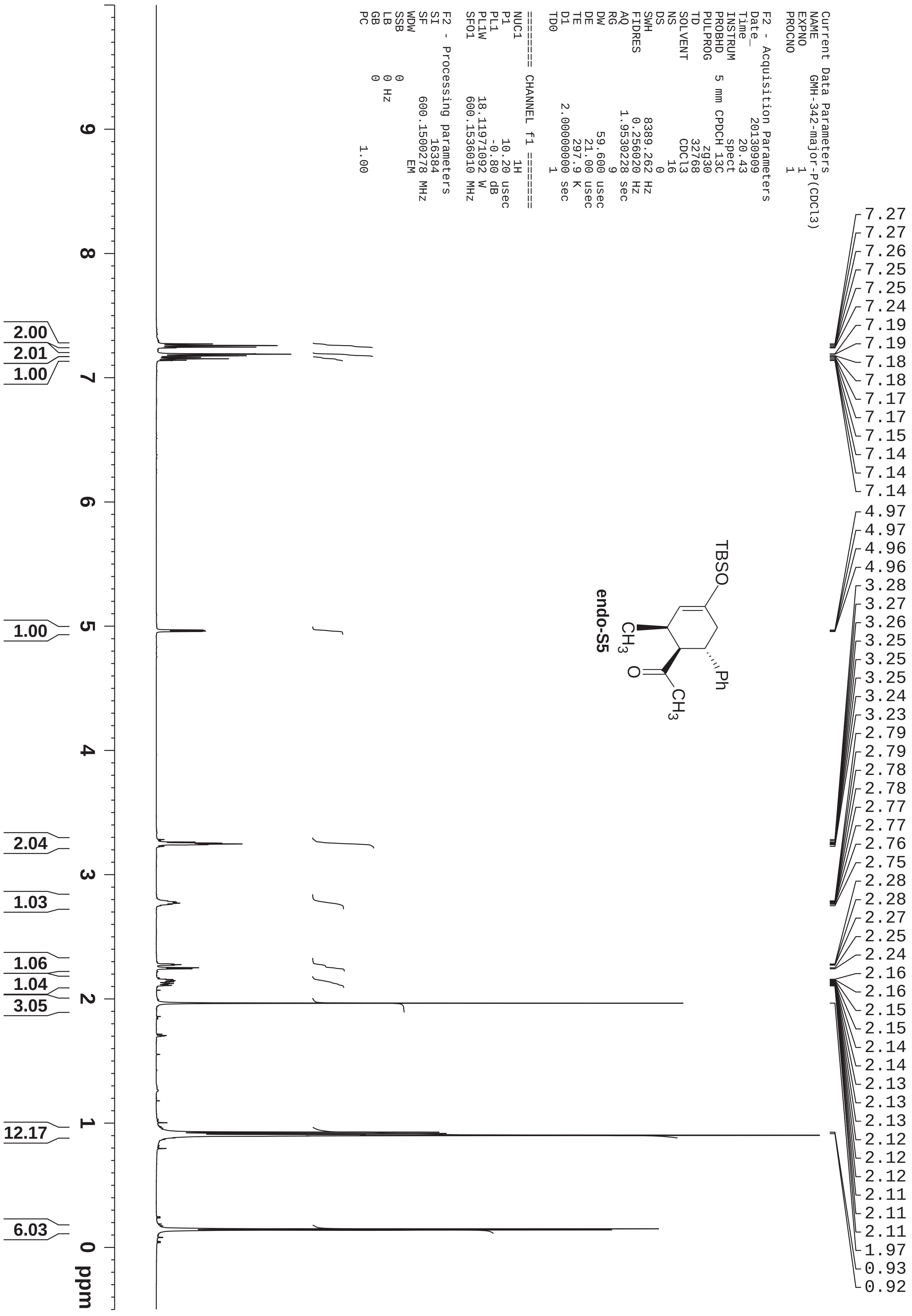

Supplementary Figure 266. ^{1}H NMR spectrum of compound endo-S5 in $CDCl_3$.

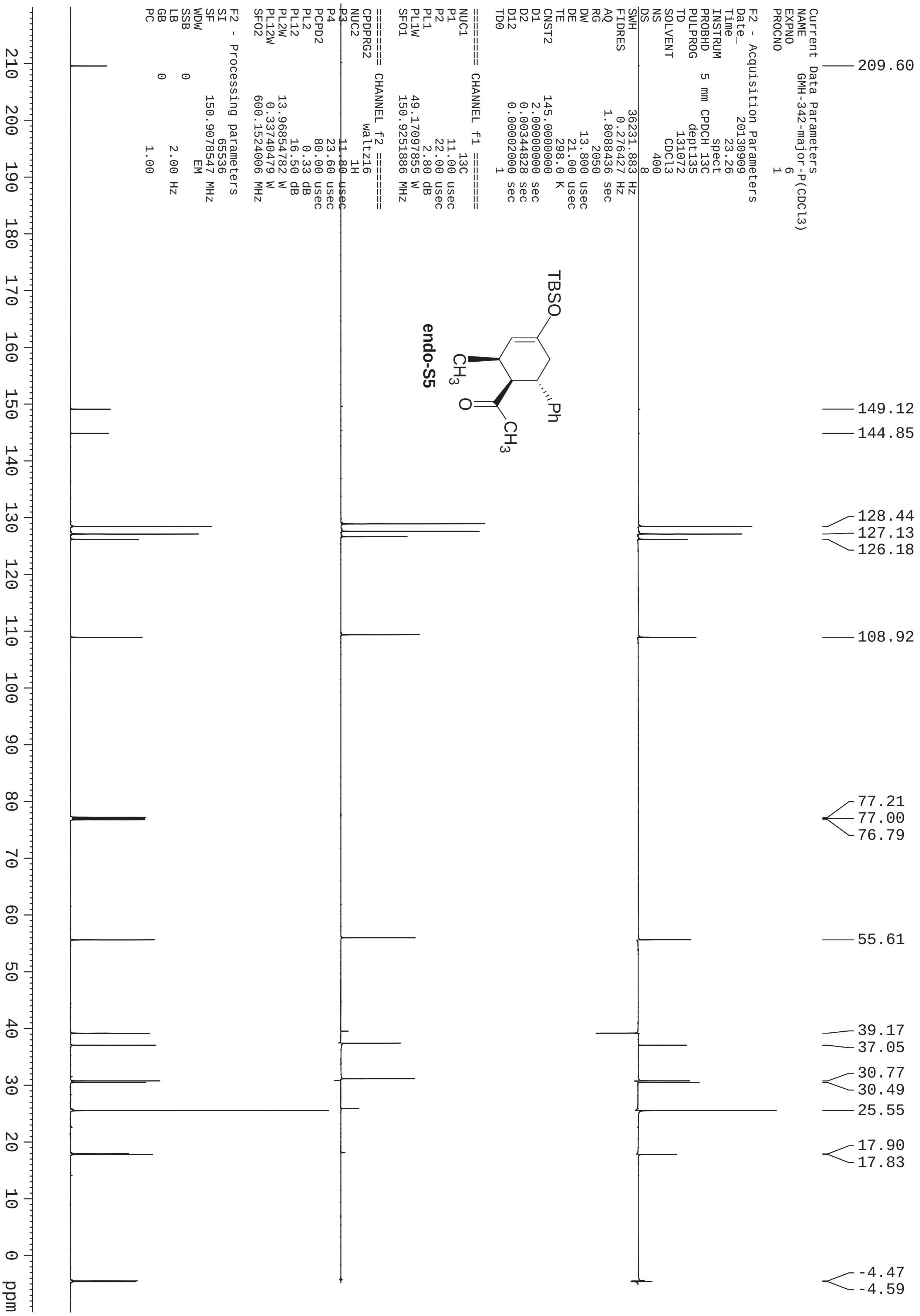

Supplementary Figure 267. ^{13}**C and DEPT NMR spectra of compound endo-S5 in** $CDCl_3$**.**

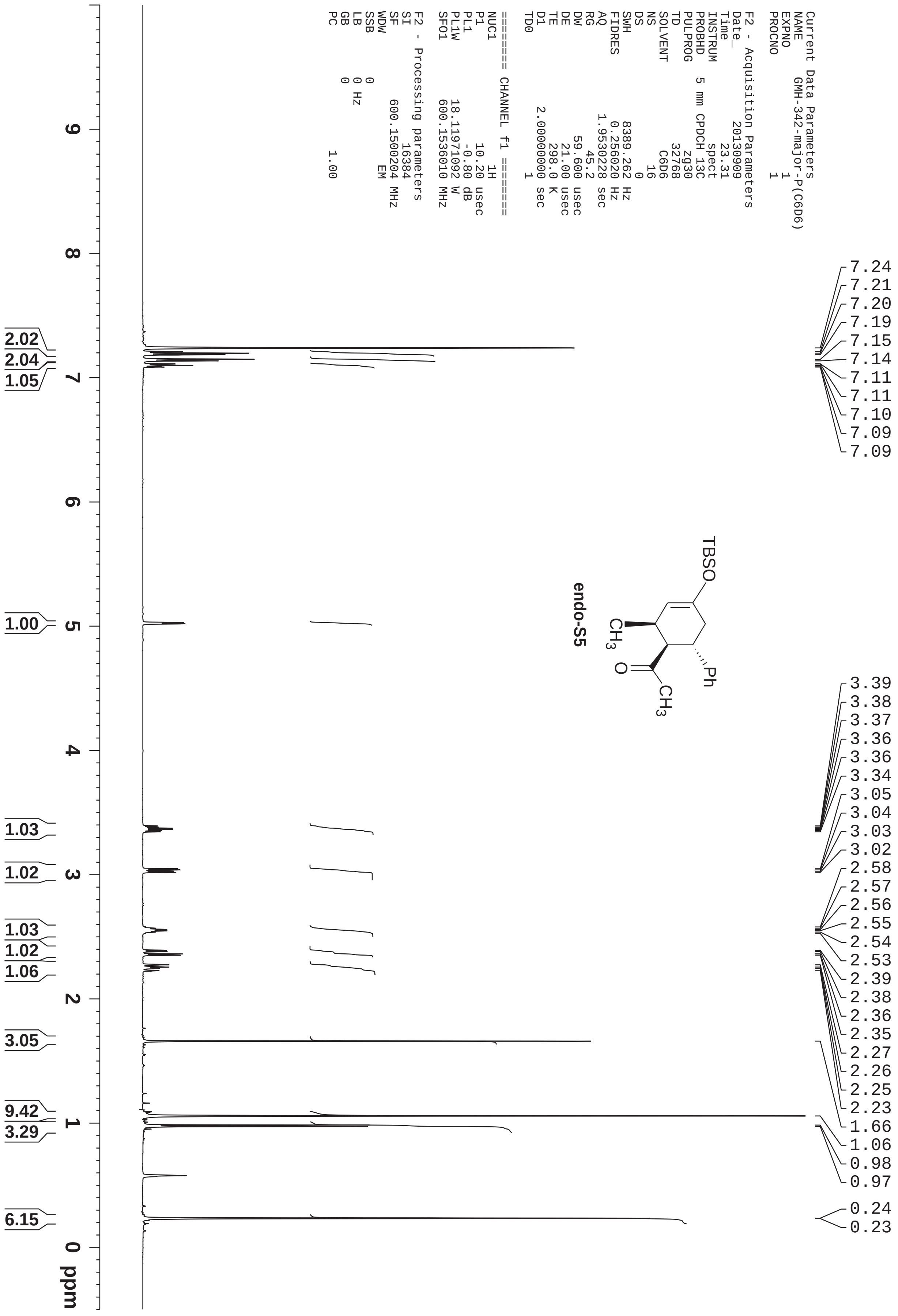

Supplementary Figure 268. **^{1}H NMR spectrum of compound endo-S5 in C_6D_6.**

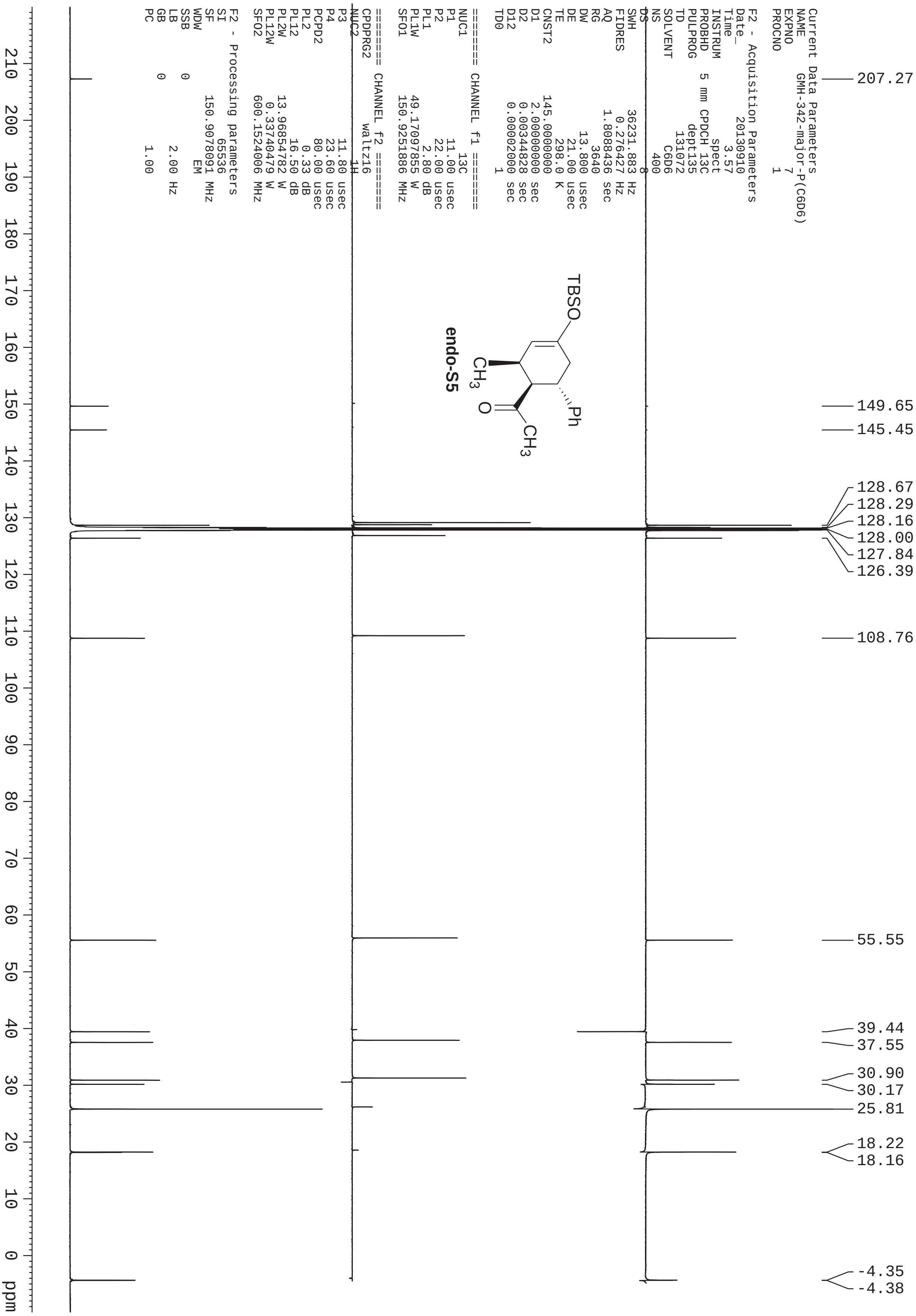

Supplementary Figure 269. ^{13}C and DEPT NMR spectra of compound endo-S5 in C_6D_6.

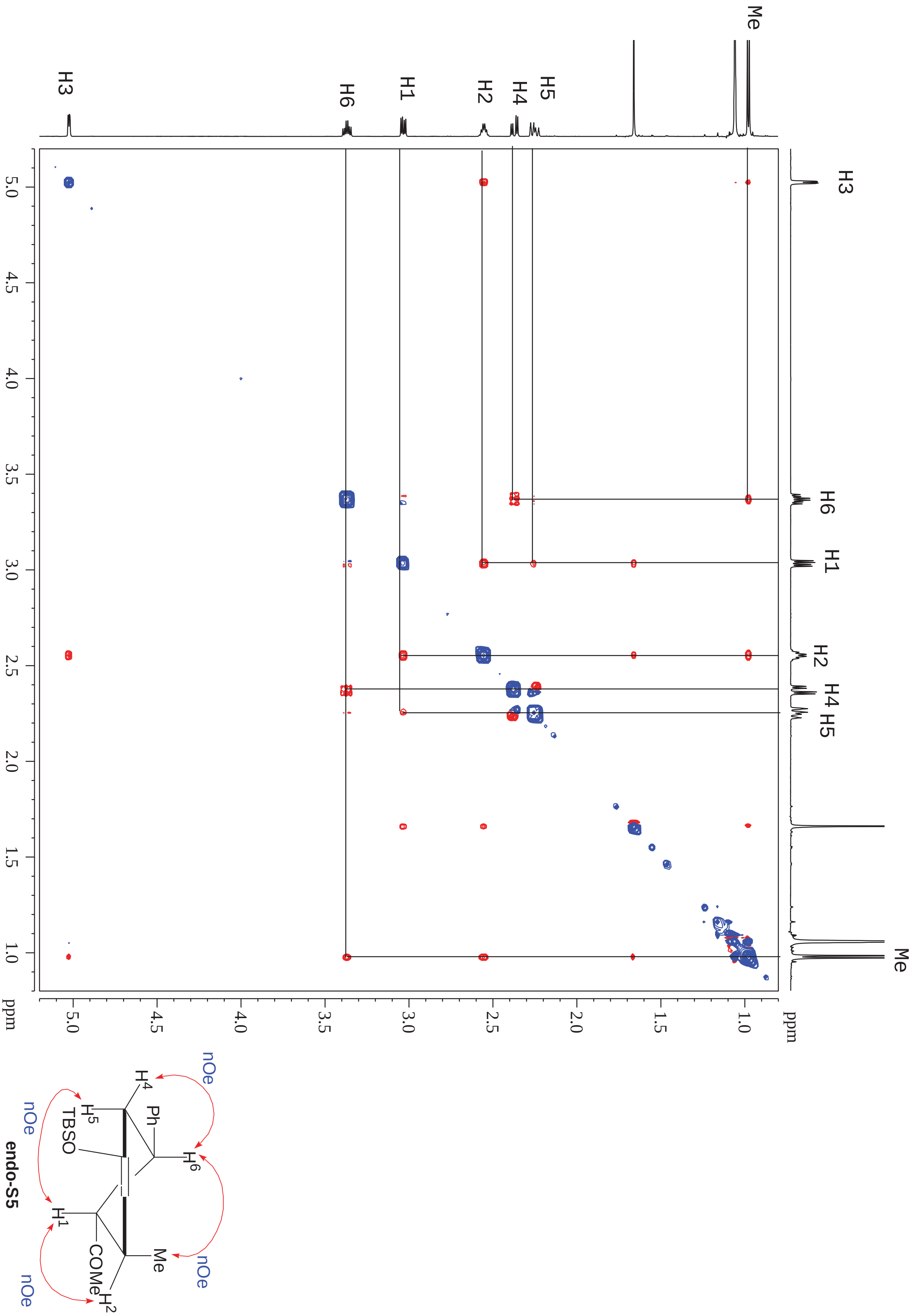

Supplementary Figure 270. NOESY NMR spectrum of compound endo-S5 in C_6D_6.

Supplementary Table 1. Thermal Diels–Alder reactions of diene S1 with various α,β-unsaturated ketones

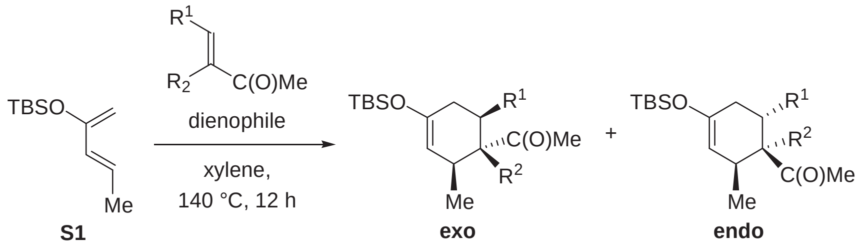

Dienophile	Product	Yield[a]	*exo*/*endo*[b]
**33**: $R^1 = R^2 = H$	**S2**	85%	1.2/1
**36**: $R^1 = H, R^2 = Me$	**S3**	74%	1/3.5
**39**: $R^1 = Me, R^2 = H$	**S4**	49%	3.3/1
**42**: $R^1 = Ph, R^2 = H$	**S5**	50%	2.4/1

[a]Based on the total isolated amount of the isomers. [b]Determined from the ^1H NMR spectra of the crude product mixture.

Supplementary Table 2. Calculated activation energy barriers ($\Delta E_a^{\ddagger}$) and product ratios of the Diels–Alder reaction pathways involving diene 7 by B3LYP and M06-2X functionals[a]

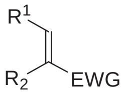	$\Delta E_a^{\ddagger}$ (kcal/mol)		k_{exo}/k_{endo}[b]
	Exo	*Endo*	
**12**: $R^1 = R^2$ = H, EWG = CN	18.7 (12.9)	19.2 (13.1)	1.8/1 (1.3/1)
**15**: R^1 = H, R^2 = Me, EWG = CN	22.6 (15.0)	21.6 (14.6)	1/3.4 (1/1.5)
**18**: R^1 = Me, R^2 = H, EWG = CN	24.4 (15.5)	26.4 (17.5)	12/1 (12/1)
**11**: R^1 = Ph, R^2 = H, EWG = CN	25.7 (11.8	26.8 (14.8)	3.6/1 (40/1)
**21**: $R^1 = R^2$ = H, EWG = C(O)H	17.4 (12.3)	16.6 (11.7)	1/2.7 (1/2.1)
**24**: R^1 = H, R^2 = Me, EWG = C(O)H	20.1 (13.2)	18.5 (12.1)	1/6.8 (1/4.2)
**27**: R^1 = Me, R^2 = H, EWG = C(O)H	22.4 (14.1)	23.8 (15.6)	5.3/1 (6.3/1)
**30**: R^1 = Ph, R^2 = H, EWG = C(O)H	25.8 (12.8)	26.1 (14.9)	1.5/1 (13/1)
**33**: $R^1 = R^2$ = H, EWG = C(O)Me	19.4 (13.3)	19.0 (12.0)	1/1.6 (1/4.8)
**36**: R^1 = H, R^2 = Me, EWG = C(O)Me	21.9 (14.1)	21.0 (12.4)	1/3.1 (1/7.6)
**39**: R^1 = Me, R^2 = H, EWG = C(O)Me	25.3 (16.2)	27.0 (16.6)	7.7/1 (1.6/1)
**42**: R^1 = Ph, R^2 = H, EWG = C(O)Me	27.2 (12.6)	27.8 (14.0)	2.1/1 (5.4/1)
**45**: $R^1 = R^2$ = H, EWG = CO_2Me	19.2 (12.7)	19.6 (12.2)	1.5/1 (1/1.9)
**48**: R^1 = H, R^2 = Me, EWG = CO_2Me	22.0 (13.7)	21.8 (13.4)	1/1.3 (1/1.3)
**51**: R^1 = Me, R^2 = H, EWG = CO_2Me	23.1 (12.7)	25.7 (15.1)	23/1 (18/1)
**54**: R^1 = Ph, R^2 = H, EWG = CO_2Me	26.1 (11.1)	27.7 (14.0)	7.1/1 (35/1)
**57**: $R^1 = R^2$ = H, EWG = NO_2	11.1 (6.4)	10.5 (2.8)	1/2.2 (1/83)
**60**: R^1 = H, R^2 = Me, EWG = NO_2	14.0 (7.5)	12.9 (4.3)	1/3.7 (1/48)
**63**: R^1 = Me, R^2 = H, EWG = NO_2	16.6 (7.7)	17.1 (7.2)	2.0/1 (1/1.8)
**66**: R^1 = Ph, R^2 = H, EWG = NO_2	18.4 (5.2)	18.2 (5.1)	1/1.3 (1/1.1)

[a]The results of the M06-2X calculations are given in parenthesis. [b]Relative reaction rate constants estimated by the Arrhenius equation.

Supplementary Table 3. Calculated Gibbs free energies of activation ($\Delta G_a^{\ddagger}$) and product ratios of the Diels–Alder reaction pathways involving diene 7 by B3LYP and M06-2X functionals[a]

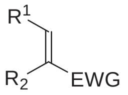	$\Delta G_a^{\ddagger}$ (kcal/mol)		k_{exo}/k_{endo}[b]
	Exo	*Endo*	
**12**: $R^1 = R^2$ = H, EWG = CN	31.5 (25.0)	32.0 (25.6)	1.9/1 (2.1/1)
**15**: R^1 = H, R^2 = Me, EWG = CN	35.6 (28.7)	33.9 (28.0)	1/8.3 (1/2.2)
**18**: R^1 = Me, R^2 = H, EWG = CN	38.0 (29.4)	40.1 (31.1)	12/1 (7.4/1)
**11**: R^1 = Ph, R^2 = H, EWG = CN	40.2 (26.4)	41.2 (27.1)	3.4/1 (2.2/1)
**21**: $R^1 = R^2$ = H, EWG = C(O)H	30.6 (25.3)	30.5 (23.9)	1/1.1 (1/6.1)
**24**: R^1 = H, R^2 = Me, EWG = C(O)H	34.1 (27.0)	32.3 (25.5)	1/9.0 (1/6.1)
**27**: R^1 = Me, R^2 = H, EWG = C(O)H	36.2 (27.4)	37.7 (27.8)	6.5/1 (1.7/1)
**30**: R^1 = Ph, R^2 = H, EWG = C(O)H	40.8 (27.6)	41.0 (29.6)	1.3/1 (12/1)
**33**: $R^1 = R^2$ = H, EWG = C(O)Me	32.9 (26.4)	33.1 (25.9)	1.2/1 (1/1.8)
**36**: R^1 = H, R^2 = Me, EWG = C(O)Me	35.4 (28.1)	35.1 (26.8)	1/1.5 (1/5.1)
**39**: R^1 = Me, R^2 = H, EWG = C(O)Me	40.0 (30.3)	41.4 (31.1)	5.5/1 (2.8/1)
**42**: R^1 = Ph, R^2 = H, EWG = C(O)Me	43.0 (28.1)	43.4 (29.0)	1.6/1 (3.0/1)
**45**: $R^1 = R^2$ = H, EWG = CO_2Me	32.7 (26.7)	34.2 (24.8)	5.6/1 (1/9.4)
**48**: R^1 = H, R^2 = Me, EWG = CO_2Me	36.0 (26.1)	36.4 (27.6)	1.6/1 (6.0/1)
**51**: R^1 = Me, R^2 = H, EWG = CO_2Me	37.4 (26.9)	39.7 (29.0)	18/1 (12/1)
**54**: R^1 = Ph, R^2 = H, EWG = CO_2Me	41.5 (26.5)	42.9 (29.4)	5.5/1 (36/1)
**57**: $R^1 = R^2$ = H, EWG = NO_2	24.3 (19.8)	24.6 (16.5)	1.5/1 (1/59)
**60**: R^1 = H, R^2 = Me, EWG = NO_2	26.9 (21.2)	27.6 (17.9)	2.5/1 (1/56)
**63**: R^1 = Me, R^2 = H, EWG = NO_2	30.6 (21.5)	31.9 (21.4)	5.1/1 (1/1.1)
**66**: R^1 = Ph, R^2 = H, EWG = NO_2	33.9 (21.0)	33.9 (19.2)	1/1 (1/8.3)

[a]The results of the M06-2X calculations are given in parenthesis. [b]Relative reaction rate constants estimated by the Arrhenius equation.

Supplementary Table 4. Calculated Gibbs free energies of activation ($\Delta G_a^{\ddagger}$) and product ratios of the Diels–Alder reaction pathways involving diene 8 by B3LYP and M06-2X functionals[a]

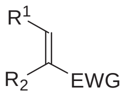	$\Delta G_a^{\ddagger}$ (kcal/mol)		k_{exo}/k_{endo}[b]
	Exo	*Endo*	
**12**: $R^1 = R^2$ = H, EWG = CN	28.9 (26.3)	29.6 (27.4)	2.2/1 (4.0/1)
**15**: R^1 = H, R^2 = Me, EWG = CN	31.9 (28.4)	33.3 (26.2)	5.0/1 (1/1.3)
**18**: R^1 = Me, R^2 = H, EWG = CN	33.9 (29.4)	36.2 (31.2)	16/1 (9.3/1)
**11**: R^1 = Ph, R^2 = H, EWG = CN	37.0 (26.5)	38.2 (29.2)	4.6/1 (25/1)
**21**: $R^1 = R^2$ = H, EWG = C(O)H	29.4 (27.6)	29.6 (26.5)	1.2/1 (1/3.7)
**24**: R^1 = H, R^2 = Me, EWG = C(O)H	32.8 (27.3)	30.8 (26.6)	1/10 (1/2.3)
**27**: R^1 = Me, R^2 = H, EWG = C(O)H	33.5 (29.6)	33.8 (29.7)	1.3/1 (1.2/1)
**30**: R^1 = Ph, R^2 = H, EWG = C(O)H	38.5 (30.8)	38.1 (31.8)	1/1.8 (3.6/1)
**33**: $R^1 = R^2$ = H, EWG = C(O)Me	32.0 (30.0)	32.3 (27.3)	1.6/1 (1/2.1)
**36**: R^1 = H, R^2 = Me, EWG = C(O)Me	34.8 (27.7)	33.7 (26.8)	1/3.7 (1/3.1)
**39**: R^1 = Me, R^2 = H, EWG = C(O)Me	37.6 (29.6)	37.7 (31.0)	1.2/1 (5.9/1)
**42**: R^1 = Ph, R^2 = H, EWG = C(O)Me	39.7 (30.4)	40.2 (31.6)	1.7/1 (4.1/1)
**45**: $R^1 = R^2$ = H, EWG = CO_2Me	31.4 (28.3)	32.2 (27.0)	2.7/1 (1/4.6)
**48**: R^1 = H, R^2 = Me, EWG = CO_2Me	33.2 (27.5)	34.0 (29.6)	2.5/1 (14/1)
**51**: R^1 = Me, R^2 = H, EWG = CO_2Me	33.6 (29.1)	36.4 (30.2)	28/1 (3.7/1)
**54**: R^1 = Ph, R^2 = H, EWG = CO_2Me	38.7 (30.2)	39.9 (31.9)	4.3/1 (7.7/1)
**57**: $R^1 = R^2$ = H, EWG = NO_2	23.3 (19.4)	22.9 (19.9)	1/1.7 (1.7/1)
**60**: R^1 = H, R^2 = Me, EWG = NO_2	25.4 (22.1)	25.5 (20.2)	1.2/1 (1/10)
**63**: R^1 = Me, R^2 = H, EWG = NO_2	27.2 (22.2)	28.6 (21.5)	5.2/1 (1/2.4)
**66**: R^1 = Ph, R^2 = H, EWG = NO_2	30.5 (21.5)	29.8 (21.0)	1/2.3 (1/2.0)

[a]The results of the M06-2X calculations are given in parenthesis. [b]Relative reaction rate constants estimated by the Arrhenius equation.

Supplementary Table 5. Optimized geometries of the *exo* and *endo* transition states involving diene 8 calculated at the B3LYP/6-311++G(d,p) level

Dienophile	*Exo*	*Endo*
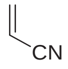 **12**	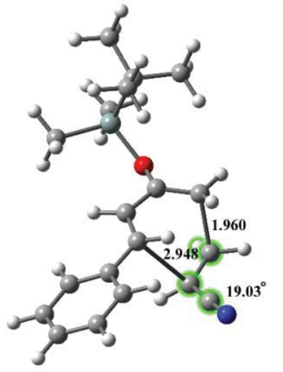	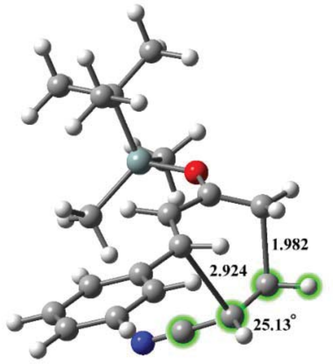
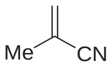 **15**	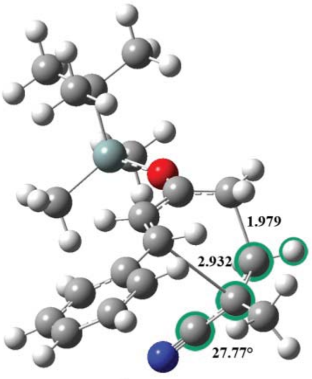	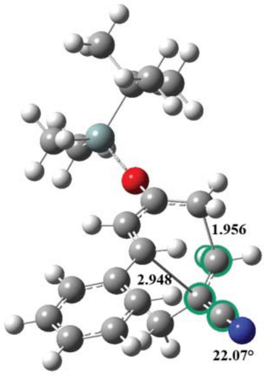
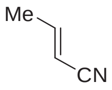 **18**	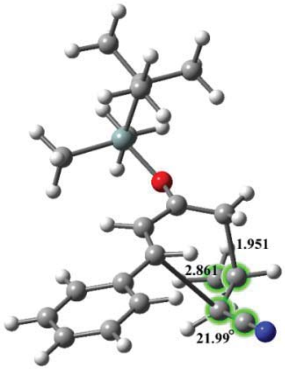	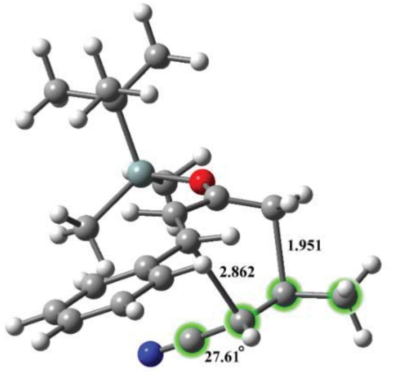
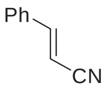 **11**	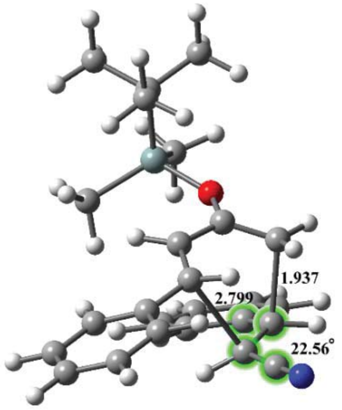	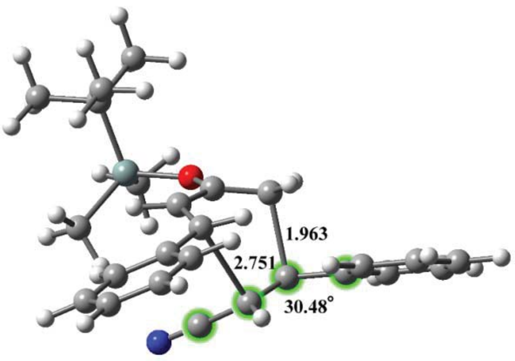

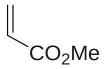
CO2Me

45

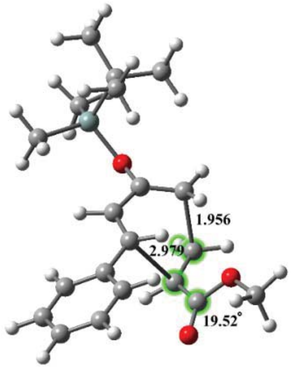
1.956
2.979
19.52°

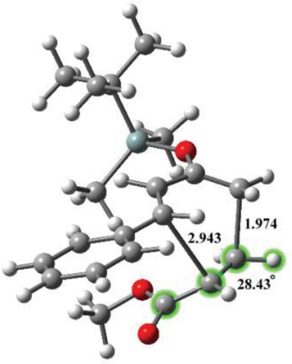
1.974
2.943
28.43°

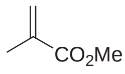
CO2Me

48

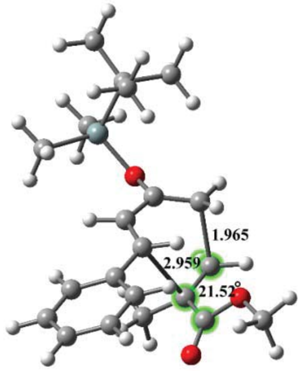
1.965
2.959
21.52°

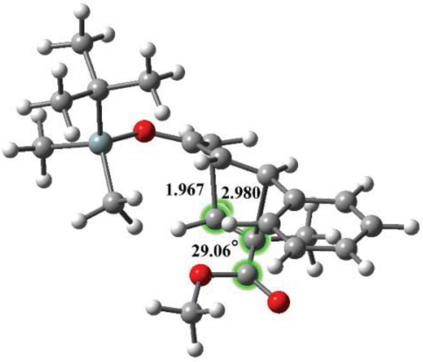
1.967
2.980
29.06°

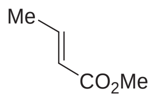
Me
CO2Me

51

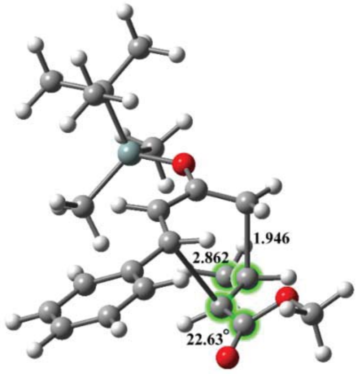
1.946
2.862
22.63°

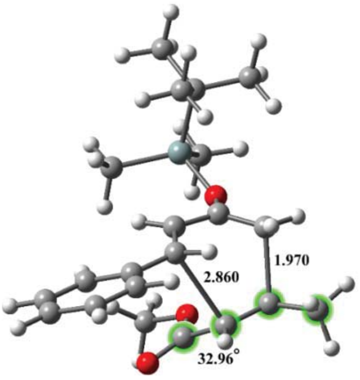
1.970
2.860
32.96°

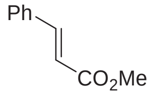
Ph
CO2Me

54

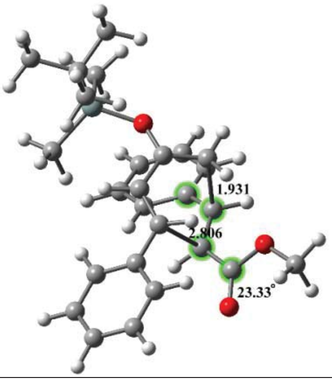
1.931
2.806
23.33°

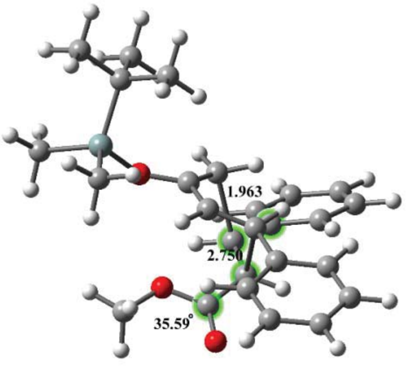
1.963
2.750
35.59°

Supplementary Table 6. Critical geometrical parameters of the *exo* and *endo* transition states of Diels–Alder reactions involving diene 7 calculated at B3LYP/6-311++G(d,p) level

Dienophile (substituent)	Pathway	Forming bond length (Å)[a]		Deviation from planarity[b]	Twist angle[c]
		$d_{1\text{-}\beta}$	$d_{4\text{-}\alpha}$		
**12** (EWG = CN)	*Exo*	1.97	2.75	23°	–11°
	Endo	1.97	2.75	30°	–6°
**15** (α-Me, EWG = CN)	*Exo*	1.97	2.75	27°	0°
	Endo	1.97	2.78	34°	–16°
**18** (β-Me, EWG = CN)	*Exo*	1.99	2.62	25°	–18°
	Endo	2.04	2.53	29°	11°
**11** (β-Ph, EWG = CN)	*Exo*	2.05	2.45	29°	–16°
	Endo	2.12	2.38	35	7°
**45** (EWG = CO_2Me)	*Exo*	1.99	2.71	23°	–7°
	Endo	1.98	2.78	30°	–12°
**48** (α-Me, EWG = CO_2Me)	*Exo*	1.98	2.77	24°	1°
	Endo	1.98	2.82	31°	–17°
**51** (β-Me, EWG = CO_2Me)	*Exo*	1.99	2.62	25°	–16°
	Endo	2.01	2.62	36°	–0°
**54** (β-Ph, EWG = CO_2Me)	*Exo*	2.03	2.47	30°	–14°
	Endo	2.07	2.43	39°	2°

[a]The subscripts represent the carbons involved in bond formation. [b]Deviation from planarity of the dienophile spanning from the EWG to the *trans*-β-function. [c]Deviation of the diene and dienophile from being parallel given by the C4-C1–Cβ-Cα dihedral angle with pivot point at the forming C1-Cβ bond; positive angles represent inward twist by the dienophile, negative angle is outward twist.

Supplementary Methods

1 General methods

Solvents were purified and dried from a safe purification system by passing through activated Al_2O_3 under argon pressure. Flash column chromatography was carried out on Silica Gel 60 (230–400 mesh, E. Merck). TLC was performed on glass plates precoated with Silica Gel 60 F254 (0.25 mm, E. Merck); detection was executed by spraying with a solution of $Ce(NH_4)_2(NO_3)_6$, $(NH_4)_6Mo_7O_{24}$, and H_2SO_4 in water or ninhydrin and acetic acid solution in *n*-butanol and subsequent heating on a hot plate. ^1H and ^{13}C NMR spectra were recorded on 400, 500 and 600 MHz instruments. Chemical shifts are in ppm referenced relative to residual $CDCl_3$ (δ = 7.26 ppm, ^1H; 77.0 ppm, ^{13}C) or C_6D_6 (δ = 7.15 ppm, ^1H; 128.0 ppm, ^{13}C). Proton peak assignments were performed using two-dimensional NMR techniques (^1H-^1H COSY, HMQC and NOESY). The hydrogen multiplicities of carbon peaks were determined using DEPT-90 and DEPT-135 experiments, the spectra of which were herein provided together with the power-gated-decoupled ^{13}C NMR spectrum.

2 Synthetic methods and characterization data

2.1 Preparation of the silyloxy dienes

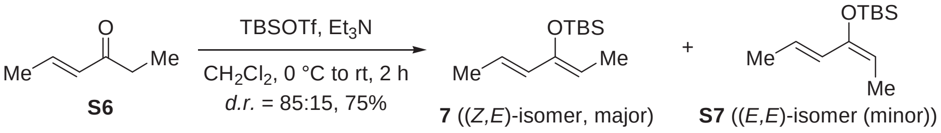

(2*Z*,4*E*)-3-(*tert*-butyldimethylsilyloxy)-2,4-hexadiene (7). Prepared according to literature procedures[1]: (*E*)-4-Hexen-3-one (**S6**, 6.0 g, 61.1 mmol) was added to a solution of Et_3N (38.1 mL, 273.6 mmol) in dry CH_2Cl_2 (122 mL) under a N_2 atmosphere. The stirred mixture was cooled to 0 °C and *tert*-butyldimethylsilyl triflate (TBSOTf, 17.0 mL, 122 mmol) was added dropwise. Once the addition of TBSOTf was complete, the mixture was allowed to warm to room temperature and stirred 2 hours. The mixture was washed successively with saturated aqueous $NaHCO_3$ and brine. The organic phase was collected and dried over $MgSO_4$ and filtered. The solvent was removed under reduced pressure to afford a pale yellow oil, which was purified by column chromatography (hexanes/0.05% Et_3N) to provide a 85:15 mixture of compound **7** and its (*E*,*E*)-isomer **S7** (combined: 9.49 g, 75%). [Note: The isomeric mixture was used directly in the cycloaddition reactions in this paper with the cycloaddition yields adjusted to the amount of **7** (i.e., 85%) in the mixture.] ^1H NMR (600

MHz, $CDCl_3$): δ 5.86 (d, J = 15.4 Hz, 1H), 5.72 (dq, J = 15.4, 6.7 Hz, 1H), 4.72 (q, J = 7.0 Hz, 1H), 1.80 (d, J = 6.7 Hz, 3H), 1.62 (d, J = 7.0 Hz, 3H), 1.01 (s, 9H), 0.11 (s, 3H); ^{13}C NMR (150 MHz, $CDCl_3$) δ 149.1, 129.8, 123.4, 107.0, 26.0 (C × 2), 18.4, 17.6, 11.6, –3.7 (C × 2).

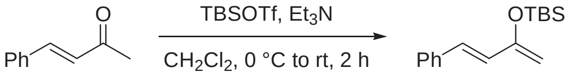

(3*E*)-3-(*tert*-butyldimethylsilyloxy)-4-phenyl-1,3-butadiene (8). Prepared according to a modified procedure[2]: (*E*)-4-Phenyl-3-buten-2-one (**42**, 6.0 g, 41.0 mmol) was added to a solution of Et_3N (11.4 mL, 82.1 mmol) in dry CH_2Cl_2 (82.1 mL) under N_2 atmosphere. The stirred mixture was cooled to 0 °C and TBSOTf (10.1 mL, 43.1 mmol) was added dropwise. Once the addition of TBSOTf was complete, the mixture was allowed to warm to room temperature and stirred 2 hours. The mixture was washed successively with saturated aqueous $NaHCO_3$ and brine. The organic phase was collected and dried over $MgSO_4$ and filtered. The solvent was removed under reduced pressure to afford a pale yellow oil, which was purified by column chromatography (hexanes) to provide the desired diene **8** (10.1 g, 95%). ^{1}H NMR (600 MHz, $CDCl_3$): δ 7.41 (d, J = 7.3 Hz, 2H), 7.32 (t, J = 7.5 Hz, 2H), 7.23 (t, J = 7.4 Hz, 1H), 6.86 (d, J = 15.7 Hz, 1H), 6.58 (d, J = 15.7 Hz), 4.45 (s, 1H), 4.42 (s, 1H), 1.03(s, 9H), 0.23 (s, 6H); ^{13}C NMR (150 MHz, $CDCl_3$): δ 155.3 (CO), 136.9, 129.3, 128.6, 127.6, 126.8, 126.6, 96.7 (C=CHH), 25.9 (Si-C($\underline{C}H_3)_3$), 18.4 (Si-$\underline{C}(CH_3)_3$),4.6 (Si-($\underline{C}H_3)_2$).

2.2 Diels–Alder reactions with α,β-unsaturated nitriles

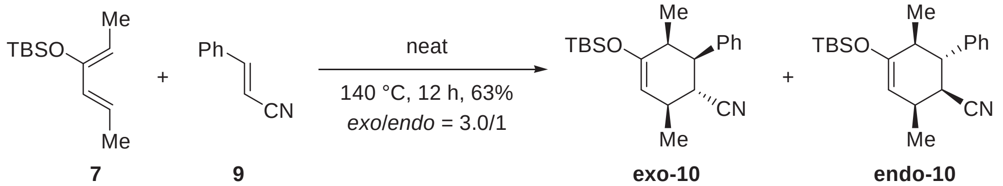

***Rac*-1-[2,5-Dimethyl-6-phenyl-4-(*tert*-butyldimethylsilyloxy)-cyclohex-3-enyl]-carbonitrile (exo-10 and endo-10).** In a 25 mL sealed tube equipped with a magnetic stir bar, cinnamonitrile (**9**, 1.09 g, 8.47 mmol, 3.0 equiv.) and silyloxydiene **7** (0.60 g, 2.40 mmol, 1.0 equiv.) were stirred for 12 h at 140 °C. Afterwards, the mixture was concentrated *in vacuo*. The residue was purified by column chromatography on silica gel (hexanes/ethyl acetate = 20/1) to give the corresponding Diels–Alder cycloadducts **exo-10** and **endo-10** (combined: 0.52 g, 63%) as a colorless oil. The diastereoselectivity of the crude products

was determined by ^{1}H NMR in C_6D_6. Integration of the signal due to the vinylic proton (H^3) of the major isomer **exo-10** (δ_H 4.50 ppm) versus that of the minor isomer **endo-10** (δ_H 4.77 ppm) gave an *exo*/*endo* ratio of 3/1.

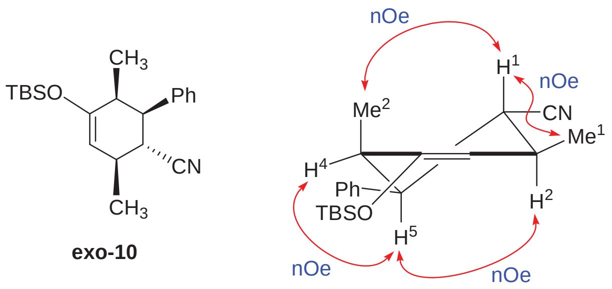

exo-10

$J_{H^2\text{-}H^3}$ = 2.0 Hz
$J_{H^1\text{-}H^5}$ = 12.4 Hz
$J_{H^1\text{-}H^2}$ = 10.0 Hz
$J_{H^4\text{-}H^5}$ = 5.2 Hz

For **exo-10**: mp: 109–110 °C (recrystallized from hexanes/ethyl acetate (4/1)); IR (thin film): ν 3029, 2959, 2930, 2858, 2242, 1666, 1454, 1256, 1192, 1191, 1054, 855, 840, 779, 700 cm^{-1}; ^{1}H NMR (600 MHz, C_6D_6): δ 7.15 (t, J = 7.4, 7.2 Hz, 2H, Ar-H), 7.07 (t, J = 7.4 Hz, 1H, Ar-H), 6.87 (d, J = 7.2 Hz, 2H, Ar-H), 4.50 (d, J = 2.1 Hz, 1H, H^3), 3.07 (dd, J = 12.3, 5.2 Hz, 1H, H^1), 2.44 (dddq, J = 10.5, 6.7, 2.1, 1.2 Hz, 1H, H^2), 2.33 (dd, J = 12.3, 10.5 Hz, 1H, H^5), 2.14 (ddq, J = 7.1, 5.2, 1.2 Hz, 1H, H^4), 1.08 (d, J = 6.7 Hz, 3H, Me^1), 0.92 (s, 9H, Si-$C(CH_3)_3$), 0.61 (d, J = 7.1 Hz, 3H, Me^2), 0.09 (s, 3H, Si-CH_3), 0.07 (s, 3H, Si-CH_3); ^{13}C NMR (150 MHz, C_6D_6): δ 154.5 (C), 139.8 (C), 128.6 (CH × 2), 128.5 (CH × 2), 127.2 (CH), 120.9 (C), 105.9 (CH), 47.1 (CH), 40.0 (CH), 35.2 (CH), 33.6 (CH), 25.7 (CH_3 × 3), 21.5 (CH_3), 18.1 (C), 13.9 (CH_3), –4.2 (CH_3), –4.7 (CH_3); HRMS (ESI): *m/z* calcd for $C_{21}H_{32}NOSi$ ($[M + H]^+$): 342.2253, found: 342.2254. The relative stereochemistry was determined by single-crystal X-ray diffraction data (racemic mixture), NOESY experiment and coupling constant analysis.

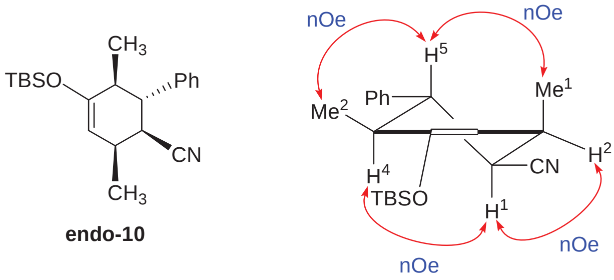

endo-10

$J_{H^1\text{-}H^2}$ = 5.2 Hz
$J_{H^1\text{-}H^5}$ = 12.4 Hz
$J_{H^4\text{-}H^5}$ = 10.5 Hz

For **endo-10**: IR (thin film): ν 3029, 2959, 2930, 2858, 2242, 1666, 1454, 1256, 1192, 1191, 1054, 855, 840, 779, 700 cm^{-1}; ^{1}H NMR (600 MHz, C_6D_6): δ 7.20–7.18 (m, 2H, Ar-H), 7.08 (dd, J = 7.4, 7.2 Hz, 1H, Ar-H), 6.87 (d, J = 7.2 Hz, 2H, Ar-H), 4.77 (dd, J = 5.7, 1.7 Hz, 1H, H^3), 3.01 (dd, J = 12.4, 5.2 Hz, 1H, H^1), 2.61 (dd, J = 12.4, 10.5 Hz, 1H, H^5), 2.51–2.45 (m, 1H, H^4), 2.14–2.08 (m, 1H, H^2), 1.31 (d, J = 6.9 Hz, 3H, Me^2), 0.95 (s, 9H, Si-$C(CH_3)_3$), 0.43 (d, J = 6.9 Hz, 3H, Me^1), 0.07 (s, 6H, Si-CH_3 × 2); ^{13}C NMR (150 MHz, C_6D_6): δ 150.3 (C),

140.4 (C), 128.5 (CH × 2), 128.4 (CH × 2), 127.2 (CH), 120.9 (C), 109.3 (CH), 46.6 (CH), 39.5 (CH), 34.7 (CH), 34.5 (CH), 25.8 (CH_3 × 3), 18.2 (CH_3), 17.6 (C), 16.8 (CH_3), –4.3 (CH_3), –4.7 (CH_3); HRMS (ESI): *m/z* calcd for $C_{21}H_{32}NOSi$ ($[M + H]^+$): 342.2253, found: 342.2254. The relative stereochemistry was determined by NOESY experiment and coupling constant analysis.

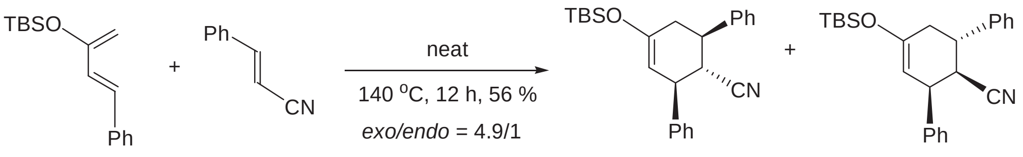

8 **9** **exo-11** **endo-11**

***Rac*-1-[2,6-diphenyl-4-(*tert*-butyldimethylsilyloxy)-cyclohex-3-enyl]-carbonitriles (exo-11 and endo-11).** In a 25 mL sealed tube equipped with a magnetic stir bar, cinnamonitrile (**9**, 0.89 g, 6.91 mmol, 3.0 equiv.) and silyloxydiene **8** (0.60 g, 2.30 mmol, 1.0 equiv.) were stirred for 12 h at 140 °C. Afterwards, the mixture was concentrated *in vacuo*. The residue was purified by column chromatography on silica gel (hexanes/ethyl acetate = 20/1) to give the corresponding Diels-Alder cycloadducts **exo-11** and **endo-11** (combined: 0.50 g, 56%) as a white solid. The diastereoselectivity of the crude products was determined by ^1H NMR in C_6D_6. Integration of the signal due to the vinylic proton (H^3) of the major isomer **exo-11** (δ_H 4.84 ppm) versus that of the minor isomer **endo-11** (δ_H 4.90 ppm) gave an *exo*/*endo* ratio of 4.9/1.

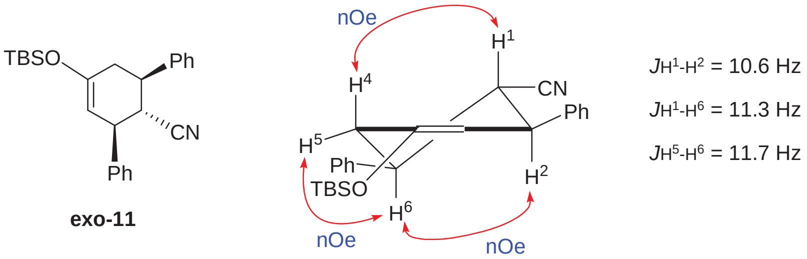

exo-11

For **exo-11**: m.p. 114–116 °C; IR (thin film): ν 3062, 3030, 2955, 2930, 2858, 2218, 1619, 1207, 967, 836, 749, 701 cm^{-1}; ^1H NMR (600 MHz, C_6D_6): δ 7.35 (d, *J* = 7.2 Hz, 2H, Ar-H), 7.21 (dd, *J* = 7.6, 7.2 Hz, 2H, Ar-H), 7.12–7.10 (m, 3H, Ar-H), 7.07 (d, *J* = 7.2 Hz, 1H, Ar-H), 6.88 (d, *J* = 7.6 Hz, 2H, Ar-H), 4.84 (s, 1H, H^3), 3.60 (d, *J* = 10.6 Hz, 1H, H^2), 2.89 (ddd, *J* = 11.7, 11.3, 5.9 Hz, 1H, H^6), 2.53 (dd, *J* = 11.3, 10.6 Hz, 1H, H^1), 2.23–2.13 (m, 2H, H^4 and H^5), 0.91 (s, 9H, Si-C(CH_3)$_3$), 0.07 (s, 3H, Si-CH_3), 0.04 (s, 3H, Si-CH_3); ^{13}C NMR (150 MHz, C_6D_6): δ 150.9 (C), 143.0 (C), 141.6 (C), 129.1 (CH × 2), 129.0 (CH × 2), 128.3 (CH × 2), 127.9 (CH), 127.7 (CH), 127.5 (CH × 2), 105.6 (CH), 46.5 (CH), 44.5 (CH), 42.4 (CH),

38.0 (CH_2), 25.6 (CH_3 × 3), 18.0 (C), –4.4 (CH_3), –4.5 (CH_3); HRMS (ESI): *m/z* calcd for $C_{25}H_{31}NOSiNa$ ($[M + Na]^+$): 412.2073, found: 412.2066. The relative stereochemistry was determined by single-crystal X-ray diffraction data (racemic mixture), NOESY experiment and coupling constant analysis.

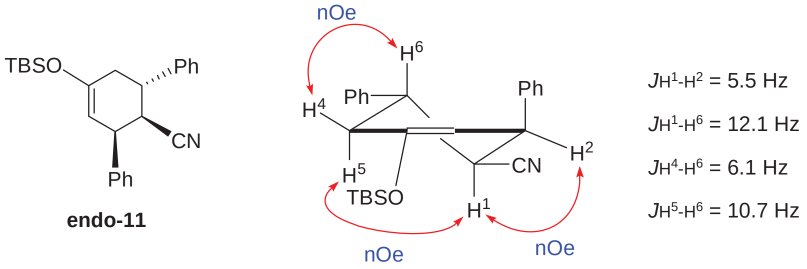

endo-11

For **endo-11**: IR (thin film): ν 3060, 3029, 2955, 2928, 2857, 2217, 1618, 1208, 969, 832, 747, 700 cm^{-1}; ^1H NMR (600 MHz, C_6D_6): δ 6.96–6.94 (m, 4H, Ar-H), 6.91 (dd, *J* = 7.6, 7.2 Hz, 2H, Ar-H), 6.52 (d, *J* = 7.2 Hz, 2H, Ar-H), 6.48–6.47 (m, 2H, Ar-H), 4.90 (d, *J* = 5.3 Hz, 1H, H^3), 3.40 (dd, *J* = 5.5, 5.3 Hz, 1H, H^2), 3.09 (dd, *J* = 12.1, 5.5 Hz, 1H, H^1), 2.91 (ddd, *J* = 12.1, 10.7, 6.1 Hz, 1H, H^6), 2.44 (dd, *J* = 17.3, 10.7 Hz, 1H, H^5), 2.37 (dd, *J* = 17.3, 6.1 Hz, 1H, H^4), 0.98 (s, 9H, Si-C(CH_3)$_3$), 0.09 (s, 3H, Si-CH_3 × 2); ^{13}C NMR (150 MHz, C_6D_6): δ 148.7 (C), 139.7 (C), 139.4 (C), 130.1 (CH × 2), 128.7 (CH × 2), 128.1 (CH × 2), 127.9 (CH × 2), 127.6 (CH), 127.0 (CH), 106.4 (CH), 47.7 (CH), 46.3 (CH), 34.2 (CH_2), 26.5 (CH), 25.7 (CH_3 × 3), 18.0 (C), –4.3 (CH_3), –4.5 (CH_3); HRMS (ESI): *m/z* calcd for $C_{25}H_{31}NOSiNa$ ($[M + H]^+$): 412.2073, found: 412.2082. The relative stereochemistry was determined by NOESY experiment and coupling constant analysis.

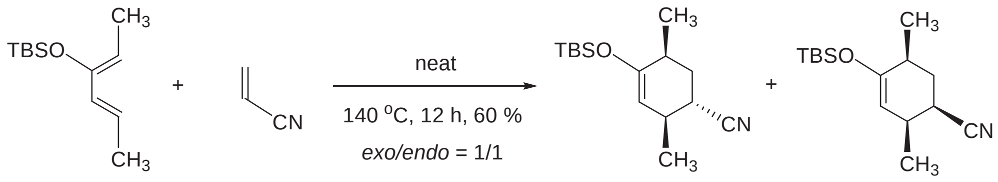

7 **12** **exo-13** **endo-13**

***Rac*-1-[2,5-dimethyl-4-(*tert*-butyldimethylsilyloxy)-cyclohex-3-enyl]-1-carbonitriles (endo-13) and (exo-13).** In a 25 mL sealed tube equipped with a magnetic stir bar, acrylonitrile (**12**, 0.45 g, 8.47 mmol, 3.0 equiv.) and silyloxydiene **7** (0.60 g, 2.40 mmol, 1.0 equiv.) were stirred for 12 h at 140 °C. Afterwards, the mixture was concentrated *in vacuo*. The residue was purified by column chromatography on silica gel (hexanes/ethyl acetate = 20/1) to give the corresponding Diels-Alder cycloadducts **exo-13** and **endo-13** (0.38 g, 60%) as a colorless oil. The diastereoselectivity of the crude products was determined by ^1H NMR

in C_6D_6. Integration of the signal due to the vinylic proton (H^3) of **exo-13** (δ_H 4.44 ppm) versus that of **endo-13** (δ_H 4.50 ppm) gave an *exo*/*endo* ratio of 1/1.

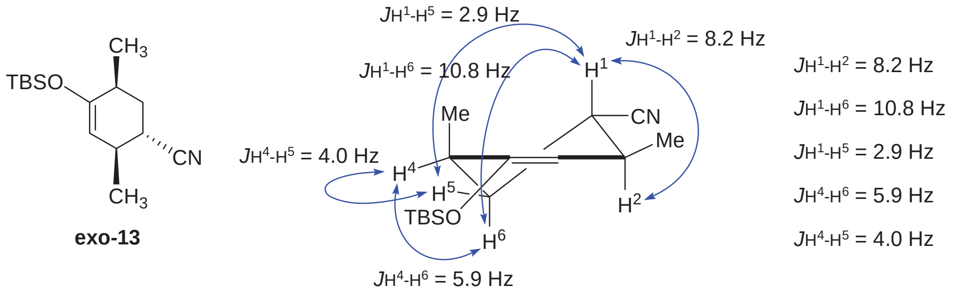

exo-13

For **exo-13**: IR (thin film): ν 2960, 2931, 2859, 2239, 1660, 1463, 1254, 1198, 1182, 841, 779 cm^{-1}; ^1H NMR (600 MHz, C_6D_6): δ 4.44 (d, *J* = 3.1 Hz, 1H, H^3), 2.17 (dqd, *J* = 8.2, 7.0, 3.1 Hz, 1H, H^2), 1.98 (dqd, *J* = 7.2, 5.9, 4.0 Hz, 1H, H^4), 1.81 (ddd, *J* = 10.8, 8.2, 2.9 Hz, 1H, H^1), 1.55 (ddd, *J* = 13.3, 10.8, 5.9 Hz, 1H, H^6), 1.33 (ddd, *J* = 13.3, 4.0, 2.9 Hz, 1H, H^5), 0.92 (s, 9H, Si-$C(CH_3)_3$), 0.81 (d, *J* = 7.0 Hz, 3H, Me^1), 0.80 (d, *J* = 7.1 Hz, 3H, Me^2), 0.06 (s, 3H, Si-CH_3), 0.05 (s, 3H, Si-CH_3); ^{13}C NMR (150 MHz; C_6H_6): δ 153.9 (C), 121.9 (C), 106.5 (CH), 33.9 (CH), 32.4 (CH_2), 32.2 (CH), 30.0 (CH), 25.8 (CH_3 × 3), 21.1 (CH_3), 18.3 (CH_3), 18.1 (C), –4.4 (CH_3), –4.5 (CH_3); HRMS (APCI): *m/z* calcd for $C_{15}H_{28}NOSi$ ($[M + H]^+$): 266.1940, found: 266.1934. The relative stereochemistry was determined by coupling constant analysis.

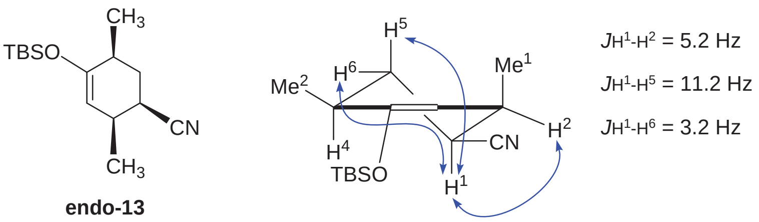

endo-13

For **endo-13**: IR (thin film): ν 2957, 2931, 2855, 2237, 1658, 1463, 1380, 1359, 1254, 1198, 855, 839, 780 cm^{-1}; ^1H NMR (400 MHz, C_6D_6): δ 4.50 (dd, *J* = 5.0, 1.4 Hz, 1H, H^3), 2.08 (ddd, *J* = 11.2, 5.2, 3.2 Hz, 1H, H^1) , 2.03–2.00 (m, 1H, H^2), 1.79–1.75 (m, 1H, H^4), 1.38 (ddd, *J* = 13.3, 6.1, 3.2 Hz, 1H, H^6), 1.33–1.28 (m, 1H, H^5), 0.99 (d, *J* = 7.0 Hz, 3H, Me^1), 0.93 (d, *J* = 6.9 Hz, 3H, Me^2), 0.92 (s, 9H, Si-$C(CH_3)_3$), 0.04 (s, 3H, Si-CH_3), 0.03 (s, 3H, Si-CH_3); ^{13}C NMR (100 MHz, C_6D_6): δ 153.3 (C), 121.0 (C), 106.6 (CH), 33.5 (CH), 30.8 (CH_2), 30.5 (CH), 29.9 (CH), 25.8 (CH_3 × 3), 18.5 (CH_3), 18.2 (CH_3), 18.1 (C), –2.8 (CH_3), –4.5 (CH_3); HRMS (APCI): *m/z* calcd for $C_{15}H_{28}NOSi$ ($[M + H]^+$): 266.1940, found: 266.1934. The relative stereochemistry was determined by coupling constant analysis.

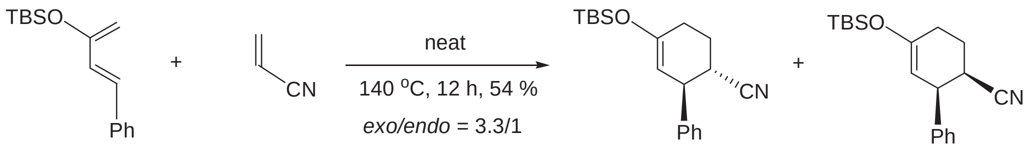

8 **12** **exo-14** **endo-14**

***Rac*-1-[2-phenyl-4-(*tert*-butyldimethylsilyloxy)-cyclohex-3-enyl]-carbonitriles (exo-14 and endo-14).** In a 25 mL sealed tube equipped with a magnetic stir bar, acrylonitrile (**12**, 0.37 g, 6.91 mmol, 3.0 equiv.) and silyloxydiene **8** (0.60 g, 2.30 mmol, 1.0 equiv.) were stirred for 12 h at 140 °C. Afterwards, the mixture was concentrated *in vacuo*. The residue was purified by column chromatography on silica gel (hexanes/ethyl acetate = 20/1) to give the corresponding Diels-Alder cycloadducts **exo-14** and **endo-14** (combined: 0.39 g, 54%) as a colorless oil. The diastereoselectivity of the crude products was determined by ^{1}H NMR in C_6D_6. Integration of the signal due to the vinylic proton (H^3) of the major isomer **exo-14** (δ_H 4.75 ppm) versus that of the minor isomer **endo-14** (δ_H 4.85 ppm) gave an *exo*/*endo* ratio of 3.3/1.

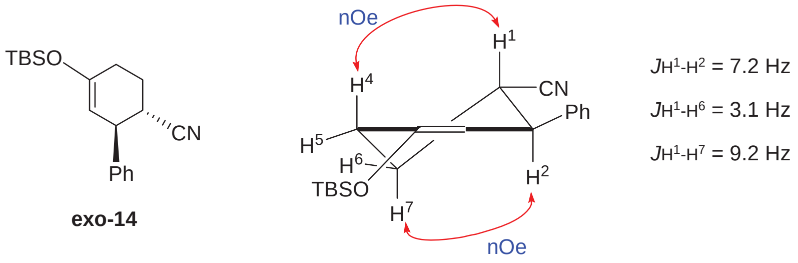

For **exo-14**: IR (thin film): ν 3029, 2956, 2932, 2860, 2242, 1718, 1496, 1454, 1253, 1202, 878, 839, 760, 700 cm^{-1}; ^{1}H NMR (600 MHz, C_6D_6): δ 7.13–7.11 (m, 2H, Ar-H), 7.07–7.04 (m, 3H, Ar-H), 4.75 (d, J = 3.0 Hz, 1H, H^3), 3.42 (dd, 1H, J = 7.2, 3.0 Hz, 1H, H^2), 2.03 (ddd, J = 9.2, 7.2, 3.1 Hz, 1H, H^1), 1.92 (ddd, J = 17.2, 5.6, 5.5 Hz, 1H, H^5), 1.76 (ddd, J = 17.2, 5.6, 5.5 Hz, H^4), 1.47 (dddd, J = 13.3, 5.6, 5.5, 3.1 Hz, 1H, H^6), 1.38 (dddd, J =13.3, 9.2, 5.6, 5.5 Hz, 1H, H^7), 0.94 (s, 9H, Si-C(CH_3)$_3$), 0.07 (s, 3H, Si-CH_3), 0.07 (s, 3H, Si-CH_3); ^{13}C NMR (150 MHz, C_6D_6): δ 151.7 (C), 143.0 (C), 128.8 (CH × 2), 128.3 (CH × 2), 128.2 (CH), 121.1 (CN), 105.0 (CH), 44.6 (CH), 34.0 (CH), 27.9 (CH_2), 25.7 (CH_3 × 3), 24.3 (CH_2), 18.1 (C), –4.3 (CH_3), –4.4 (CH_3); HRMS (ESI): *m/z* calcd for $C_{19}H_{28}NOSi$ ([M + H]$^+$): 314.1940, found 314.1931. The relative stereochemistry was determined by NOESY experiments and coupling constant analysis.

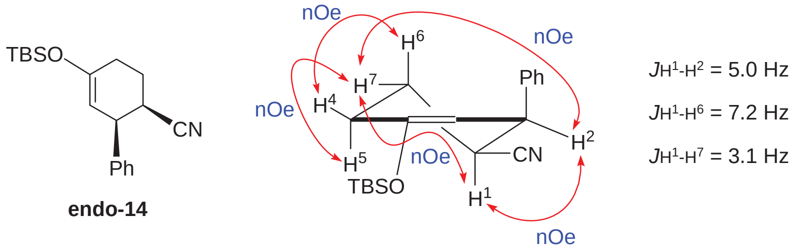

For **endo-14**: m.p. 95–96 °C; IR (thin film): ν 3028, 2952, 2929, 2955, 2232, 1665, 1450, 1366, 1181, 887, 699 cm^{-1}; ^{1}H NMR (600 MHz, C_6D_6): δ 7.26 (d, *J* = 7.3Hz, 2H, Ar-H), 7.20 (d, *J* = 7.3Hz, 2H, Ar-H), 7.10 (d, *J* = 7.3 Hz, 1H, Ar-H), 4.85 (d, *J* = 2.5 Hz, 1H, H^3), 3.25 (dd, *J* = 5.0, 2.5 Hz, 1H, H^2), 2.31 (ddd, *J* = 7.2, 5.0, 3.1 Hz, 1H, H^1), 2.20 (ddd, *J* = 17.6, 8.6, 6.2 Hz, 1H, H^4), 1.78 (ddd, *J* = 17.6, 6.2, 5.1 Hz, 1H, H^5), 1.58–1.49 (m, 1H, H^6), 1.27–1.21 (m, 1H, H^7), 0.96 (s, 9H, Si-C(CH_3)$_3$), 0.16 (s, 3H, Si-CH_3), 0.12 (s, 3H, Si-CH_3); ^{13}C NMR (150 MHz; C_6D_6): δ 151.8 (C), 141.2 (C), 129.0 (CH × 2), 128.6 (CH × 2), 127.7 (CH), 119.6 (CN), 105.0 (CH), 42.7 (CH), 33.2 (CH), 27.4 (CH_2), 25.7 (CH_3 × 3), 24.4 (CH_2), 18.1 (C), –4.2 (CH_3), –4.3 (CH_3); HRMS (ESI): m/z calcd for $C_{19}H_{27}NOSiNa$ ([M + Na]$^+$): 336.1760, found: 336.1752. The relative stereochemistry was determined by NOESY experiments and coupling constant analysis.

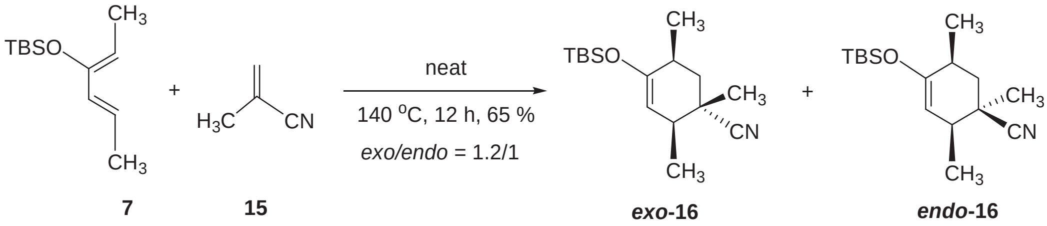

***Rac*-1-[1,2,5-trimethyl-4-(*tert*-butyldimethylsilyloxy)-cyclohex-3-enyl]-1-carbonitriles (exo-16 and endo-16).** In a 25 mL sealed tube equipped with a magnetic stir bar, methacrylonitrile **(15**, 0.57 g, 8.49 mmol, 3.0 equiv) and silyloxydiene **7** (0.60 g, 2.40 mmol, 1.0 equiv) were stirred for 12 h at 140 °C. Afterwards, the mixture was concentrated *in vacuo*. The residue was purified by column chromatography on silica gel (hexanes/ethyl acetate = 20/1) to give the corresponding Diels-Alder cycloadducts **exo-16** and **endo-16** (combined: 0.44 g, 65%) as a colorless oil. The diastereoselectivity of the crude products was determined by ^{1}H NMR in C_6D_6. Integration of the signal due to the vinylic proton (H^2) of **exo-16** (δH 4.75 ppm) versus that of **endo-16** (δH 4.49 ppm) gave an *exo*/*endo* ratio of 1.2/1.

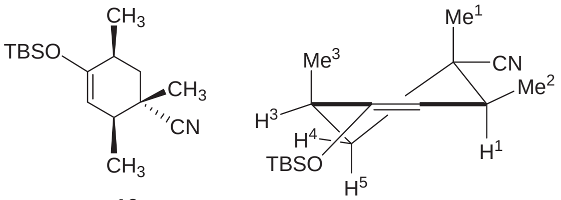

exo-16

For **exo-16**: IR (thin film): ν 2957, 2932, 2852, 2238, 1657, 1463, 1381, 1356, 1253, 1198, 856, 836, 780 cm^{-1}; ^{1}H NMR (600 MHz, C_6D_6): δ 4.75 (dd, J = 5.6, 1.6 Hz, 1H, H^2), 2.50–2.43 (m, 1H, H^3), 2.18–2.14 (m, 1H, H^1), 1.30 (ddd, J = 13.6, 6.3, 1.6 Hz, 1H, H^4), 0.96 (s, 9H, SiC(CH_3)$_3$), 0.95–0.94 (m, 1H, H^5), 0.95 (d, J = 7.0 Hz, 3H, Me^3), 0.85 (s, 3H, Me^1), 0.53 (d, J = 7.0 Hz, 3H, Me^2), 0.21 (s, 3H, Si-CH_3), 0.17 (s, 3H, Si-CH_3); ^{13}C NMR (150 MHz; C_6H_6): δ 152.7 (C), 123.9 (C), 105.8 (CH), 38.4 (CH_2), 38.0 (CH), 34.8 (C), 31.3 (CH), 25.8 (CH_3 × 3), 24.1 (CH_3), 19.6 (CH_3), 18.22 (C), 18.24 (CH_3), –4.45 (CH_3), –4.46 (CH_3); HRMS (APCI): *m/z* calcd for $C_{15}H_{28}NOSi$ ([M + H]$^+$): 280.2097, found: 280.2100. The relative stereochemistry was determined by NOESY experiment analysis.

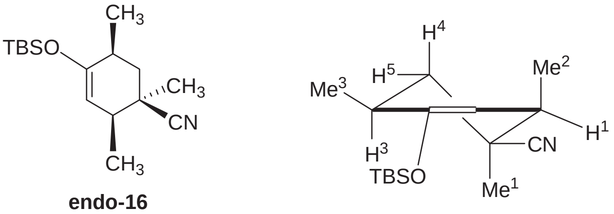

endo-16

For **endo-16**: IR (thin film): ν 2958, 2932, 2853, 2237, 1657, 1463, 1380, 1358, 1253, 1198, 856, 837, 780 cm^{-1}; ^{1}H NMR (400 MHz, C_6D_6) δ 4.49 (dd, J = 4.1, 1.5 Hz, 1H, H^2), 1.93–1.88 (m, 1H, H^3), 1.80–1.76 (m, 1H, H^1), 1.52 (dd, J = 13.7, 7.1 Hz, 1H, H^5), 1.35 (dd, J = 13.7, 6.8 Hz, 1H, H^4), 1.10 (d, J = 7.1 Hz, 3H, Me^3), 1.08 (d, J = 7.0 Hz, 3H, Me^2), 1.00 (s, 3H, Me^1), 0.93 (s, 9H, Si-C(CH_3)$_3$), 0.08 (s, 3H, Si-CH_3), 0.05 (s, 3H, Si-CH_3); ^{13}C NMR (150 MHz; C_6H_6): δ 153.8 (C), 124.9 (C), 108.0 (CH), 37.4 (CH_2), 36.8 (CH), 35.0 (C), 33.0 (CH), 25.9 (CH_3 × 3), 23.3 (CH_3), 18.3 (C), 18.0 (CH_3), 16.4 (CH_3),–4.1 (CH_3), –4.7 (CH_3); HRMS (APCI): *m/z* calcd for $C_{15}H_{28}NOSi$ ([M + H]$^+$): 280.2097, found: 280.2095. The relative stereochemistry was determined by NOESY experiment and coupling constant analysis.

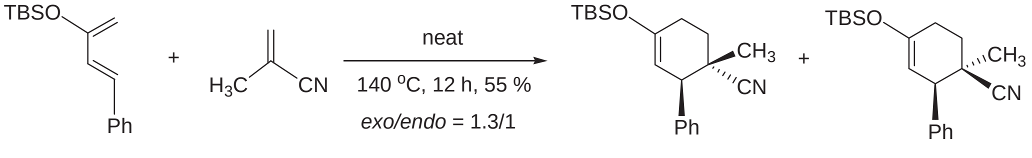

8 **15** **exo-17** **endo-17**

Rac-1-[1-methyl-2-phenyl-4-(tert-butyldimethylsilyloxy)-cyclohex-3-enyl]-carbonitriles (exo-17 and endo-17). In a 25 mL sealed tube equipped with a magnetic stir bar, methacrylonitrile (**15**, 0.46 g, 6.91 mmol, 3.0 equiv.) and silyloxydiene **8** (0.60 g, 2.30 mmol, 1.0 equiv.) were stirred for 12 h at 140 °C. Afterwards, the mixture was concentrated *in vacuo*. The residue was purified by column chromatography on silica gel (hexanes/ethyl acetate = 20/1) to give the corresponding Diels-Alder cycloadducts **exo-17** and **endo-17** (0.42 g, 55%) as a colorless oil. The diastereoselectivity of the crude products was determined by ^{1}H NMR in C_6D_6. Integration of the signal due to the allylic proton H^1 of the major isomer **exo-17** (δ_H 3.55 ppm) versus that of the minor isomer **endo-17** (δ_H 2.94 ppm) gave an *exo*/*endo* ratio of 1.3/1.

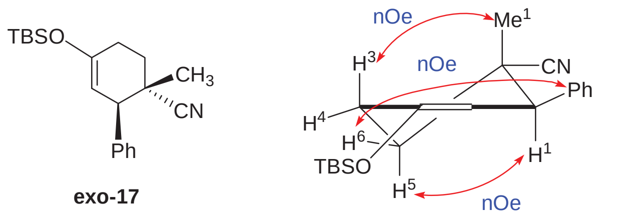

For **exo-17**: m.p. 53–54 °C; IR (thin film): ν 3029, 2951, 2928, 2231, 1664, 1450, 1367, 1182, 887, 698 cm^{-1}; ^{1}H NMR (600 MHz, C_6D_6): δ 7.09–7.01 (m, 5H, Ar-H), 4.86 (d, J = 4.4 Hz, 1H, H^2), 3.55 (d, 1H, J = 4.4 Hz, 1H, H^1), 2.23 (ddd, J = 17.2, 8.3, 6.8 Hz, 1H, H^3), 1.93 (ddd, J = 17.2, 6.1, 5.1 Hz, 1H, H^4), 1.43 (ddd, J = 14.4, 8.3, 6.1 Hz, H^6), 1.38 (ddd, J = 14.4, 6.8, 5.1 Hz, 1H, H^5), 0.96 (s, 9H, Si-C(CH_3)$_3$), 0.68 (s, 3H, Me^1), 0.15 (s, 3H, Si-CH_3), 0.12 (s, 3H, Si-CH_3); ^{13}C NMR (150 MHz, C_6D_6) δ 151.4 (C), 139.6 (C), 130.1 (CH × 2), 128.2 (CH × 2), 127.6 (CH), 124.5 (C), 105.2 (CH), 48.5 (CH), 35.1 (C), 30.0 (CH_2), 27.3 (CH_2), 25.8 (CH_3 × 3), 22.1 (CH_3), 18.1 (C), –4.2 (CH_3), –4.4 (CH_3); HRMS (ESI): *m/z* calcd for $C_{19}H_{28}NONaSi$ ([M + Na]$^+$): 350.1916, found 350.1909. The relative stereochemistry was determined by NOESY experiment analysis.

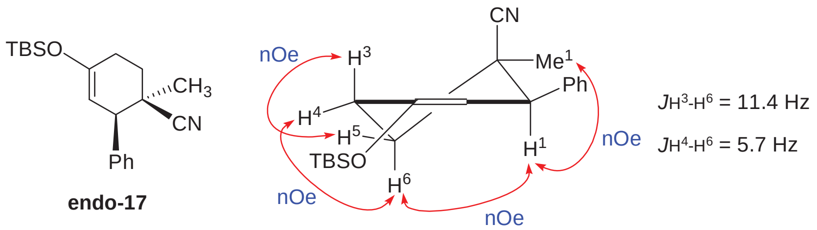

For **endo-17**: IR (thin film): ν 3028, 2952, 2928, 2231, 1665, 1451, 1367, 1183, 886, 699 cm^{-1}; ^{1}H NMR (600 MHz, C_6D_6): δ 7.31 (d, J = 7.1Hz, 2H, Ar-H), 7.18 (dd, J = 7.7, 7.1 Hz, 2H, Ar-H), 7.10–7.08 (m, 1H, Ar-H), 4.89 (s, 1H, H^2), 2.94 (br, 1H, H^2), 2.58–2.52 (m, 1H, H^3), 1.93 (dd, J = 17.7, 5.7 Hz, 1H, H^4), 1.58 (ddd, J = 13.2, 6.0, 2.1 Hz, 1H, H^5), 1.58 (ddd, J =

13.2, 11.4, 5.7 Hz, 1H, H[6]), 0.97 (s, 9H, Si-C(CH_3)$_3$), 0.86 (s, 3H, Me[1]), 0.22 (s, 3H, Si-CH_3), 0.16 (s, 3H, Si-CH_3); ^{13}C NMR (150 MHz, C_6D_6): δ 151.6 (C), 141.0 (C), 129.7 (CH × 2), 128.5 (CH × 2), 127.9 (CH), 121.9 (C), 106.6 (CH), 50.8 (CH), 38.1 (C), 34.5 (CH_2), 28.1 (CH_2), 25.8 (CH_3 × 3), 24.2 (CH_3), 18.1 (C), –4.1 (CH_3), –4.4 (CH_3); HRMS (ESI): *m*/*z* calcd for $C_{20}H_{27}NOSi$ ($[M + H]^+$): 328.2097, found: 328.2091. The relative stereochemistry was determined by NOESY experiment and coupling constant analysis.

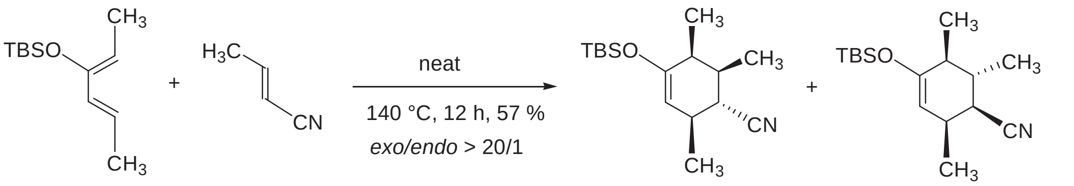

7 **18** **exo-19** **endo-19**

***Rac*-1-[2,5,6,-trimethyl-4-(*tert*-butyldimethylsilyloxy)-cyclohex-3-enyl]-carbonitriles (exo-19 and endo-19).** In a 25 mL sealed tube equipped with a magnetic stir bar, crotononitrile (**18**, 0.69 mL, 8.47 mmol, 3.0 equiv.) and silyloxydiene **7** (0.60 g, 2.40 mmol, 1.0 equiv.) were stirred for 12 h at 140 °C. Afterwards, the mixture was concentrated *in vacuo*. The residue was purified by column chromatography on silica gel (hexane/ethyl acetate = 20/1) to give the corresponding Diels-Alder cycloadducts **exo-19** and **endo-19** (combined: 0.38 g, 57%) as a colorless oil. The diastereoselectivity of the crude products was determined by ^{1}H NMR in C_6D_6. Integration of the signal due to the vinylic proton (H[3]) of the major isomer **exo-19** (δ_H 4.40 ppm) versus that of the minor isomer **endo-19** (δ_H 4.67 ppm) gave an *exo*/*endo* ratio of >20/1.

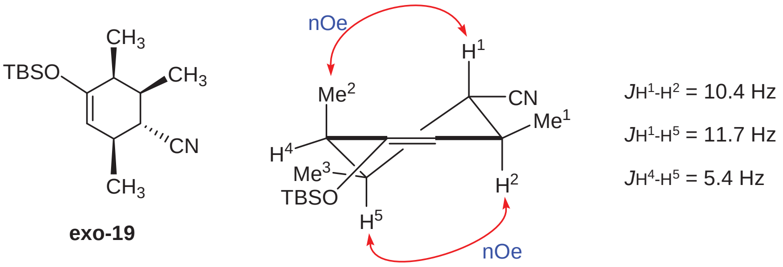

exo-19

For **exo-19**: m.p. 47–49 °C; IR (thin film): ν 3048, 2961, 2931, 2859, 2237, 1665, 1464, 1257, 1198, 837, 780 cm^{-1}; ^{1}H NMR (600 MHz, C_6D_6): δ 4.40 (d, *J* = 2.1 Hz, 1H, H[3]), 2.23 (dqdd, *J* = 10.4, 6.9, 2.1, 1.3 Hz, 1H, H[2]), 1.81 (qdd, *J* = 7.0, 5.4, 1.3 Hz, 1H, H[4]), 1.69 (dqd, *J* = 11.7, 6.9, 5.4 Hz, 1H, H[5]), 1.59 (dd, *J* = 11.7, 10.4 Hz, 1H, H[1]), 0.94 (d, *J* = 6.9 Hz, 3H, Me[1]), 0.93 (s, 9H, Si-C(CH_3)$_3$), 0.88 (d, *J* = 6.9 Hz, 3H, Me[3]), 0.67 (d, *J* = 7.0 Hz, 3H, Me[2]), 0.05 (s, 3H, Si-CH_3), 0.04 (s, 3H, Si-CH_3); ^{13}C NMR (150 MHz, C_6D_6): δ 154.6 (C), 121.2 (C), 106.1 (CH), 38.2 (CH), 36.5 (CH), 34.8 (CH), 34.6 (CH), 25.7 (CH_3 × 3), 21.4 (CH_3), 18.1 (C),

16.7 (CH_3), 12.5 (CH_3), –4.3 (CH_3), –4.7 (CH_3); HRMS (ESI): *m/z* calcd for $C_{16}H_{30}NOSi$ ($[M + H]^+$): 280.2097, found: 280.2098. The relative stereochemistry was determined by NOESY experiment and coupling constant analysis.

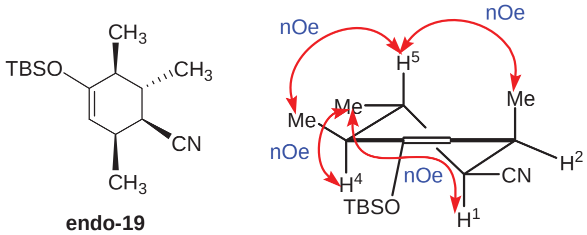

endo-19

For **endo-19**: IR (thin film): ν 3047, 2960, 2932, 2858, 2236, 1665, 1464, 1256, 1198, 838, 780 cm^{-1}; ^1H NMR (600 MHz, C_6D_6): δ 4.67 (d, *J* = 5.6 Hz, 1H, H^3), 2.28–2.25 (m, 1H, H^4), 1.86–1.82 (m, 1H, H^1), 1.81–1.78 (m, 1H, H^2), 1.65–1.62 (m, 1H, H^5), 1.17 (d, *J* = 7.0 Hz, 3H, Me^2), 0.91 (s, 9H, Si-C(CH_3)$_3$), 0.88 (d, *J* = 6.9 Hz, 3H, Me^3), 0.47 (d, *J* = 7.1 Hz, 3H, Me^1), 0.04 (s, 3H, Si-CH_3), 0.02 (s, 3H, Si-CH_3); ^{13}C NMR (150 MHz, C_6D_6): δ 150.4 (C), 121.2 (C), 109.6 (CH), 38.8 (CH), 37.3 (CH), 34.1 (CH), 32.8 (CH), 25.7 (CH_3 × 3), 18.2 (C), 17.6 (CH_3), 16.9 (C H_3), 15.5 (CH_3), –4.3 (CH_3), –4.8 (CH_3); HRMS (ESI): *m/z* calcd for $C_{16}H_{30}NOSi$ ($[M + H]^+$): 280.2097, found: 280.2094. The relative stereochemistry was determined by NOESY experiment analysis.

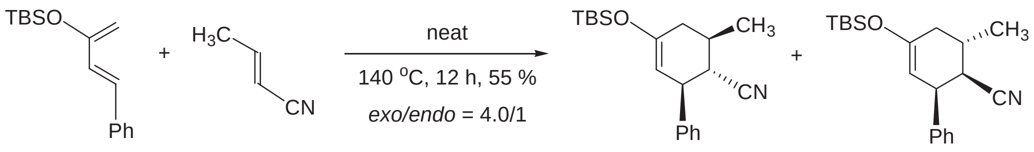

8 18 exo-20 endo-20

***Rac*-1-[6-methyl-2-phenyl-4-(*tert*-butyldimethylsilyloxy)-cyclohex-3-enyl]-carbonitriles (exo-20 and endo-20).** In a 25 mL sealed tube equipped with a magnetic stir bar, crotononitrile (**18**, 0.56 mL, 6.91 mmol, 3.0 equiv.) and silyloxydiene **8** (0.60 g, 2.30 mmol, 1.0 equiv.) were stirred for 12 h at 140 °C. Afterwards, the mixture was concentrated *in vacuo*. The residue was purified by column chromatography on silica gel (hexanes/ethyl acetate = 20/1) to give the corresponding Diels-Alder cycloadducts **exo-20** and **endo-20** (combined: 0.42 g, 55%) as a colorless oil. The diastereoselectivity of the crude products was determined by ^1H NMR in C_6D_6. Integration of the signal due to the vinylic proton (H^3) of the major isomer **exo-20** (δ_H 4.71 ppm) versus that of the minor isomer **endo-20** (δ_H 4.82 ppm) gave an *exo*/*endo* ratio of 4.0/1.

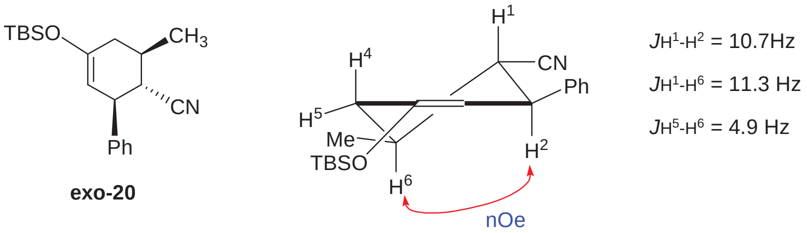

For **exo-20**: IR (thin film): ν 3048, 2961, 2859, 2237, 1665, 1463, 1257, 1198, 1180, 878, 837, 780 cm^{-1}; ^{1}H NMR (600 MHz, C_6D_6): δ 7.22 (d, *J* = 7.7 Hz, 2H, Ar-H), 7.18 (dd, *J* = 7.7, 7.4 Hz, 2H, Ar-H), 7.09 (d, *J* = 7.4 Hz, 1H, Ar-H), 4.71 (s, 1H, H^3), 3.40 (d, *J* = 10.7 Hz, 1H, H^2), 1.83 (dd, *J* = 4.9, 17.0 Hz, 1H, H^5), 1.75 (dd, *J* = 11.3, 10.7 Hz, 1H, H^1), 1.68–1.61 (m, 1H, H^6), 1.52–1.46 (m, 1H, H^4), 0.93 (s, 9H, Si-C(CH_3)$_3$), 0.85 (d, *J* = 6.6 Hz, 3H, Me), 0.04 (s, 3H, Si-CH_3), 0.01(s, 3H, Si-CH_3); ^{13}C NMR (150 MHz, C_6D_6): δ 150.7 (C), 143.2 (C), 128.9 (CH × 2), 128.2 (CH × 2), 127.3 (CH), 120.2 (C), 105.7 (CH), 46.0 (CH), 43.0 (CH), 37.2 (CH_2), 32.4 (CH), 25.7 (CH_3 × 3), 19.8 (CH_3), 18.0 (C), –4.4 (CH_3), –4.5 (CH_3); HRMS (APCI): *m/z* calcd for $C_{20}H_{30}NOSi$ ([M + H]$^+$): 328.2097, found: 328.2097. The relative stereochemistry was determined by NOESY experiment and coupling constant analysis.

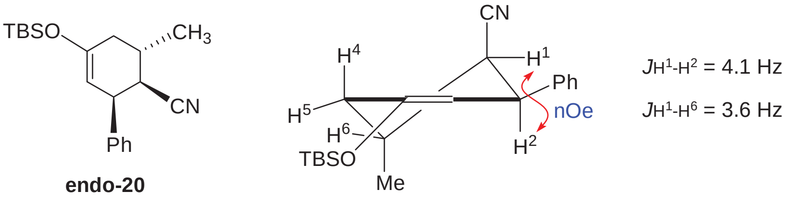

For **endo-20**: IR ($CHCl_3$): ν 3046, 2962, 2859, 2238, 1665, 1463, 1256, 1197, 1180, 876, 838, 780 cm^{-1}; ^{1}H NMR (600 MHz, C_6D_6): δ 7.09–7.07 (m, 2H, Ar-H), 7.04–7.01 (m, 1H, Ar-H), 6.98 (d, *J* = 7.2 Hz, 2H, Ar-H), 4.82 (d, *J* = 4.7 Hz, 1H, H^3), 3.61 (dd, *J* = 4.7, 4.1 Hz, 1H, H^2), 2.24 (dd, *J* = 4.1, 3.6 Hz, 1H, H^1), 1.98–1.90 (m, 2H, H^4 and H^5), 1.67-1.61 (m, 1H, H^6), 0.98 (s, 9H, Si-C(CH_3)$_3$), 0.68 (d, *J* = 6.8 Hz, 3H, Me), 0.15 (s, 3H, Si-CH_3), 0.14 (s, 3H, Si-CH_3); ^{13}C NMR (150 MHz, C_6D_6) δ 151.9 (C), 142.6 (C), 128.6 (CH × 2), 128.3 (CH × 2), 127.4 (CH), 120.1 (C), 103.0 (CH), 43.1 (CH), 39.7 (CH), 35.2 (CH_2), 26.3 (CH), 25.7 (CH_3 × 3), 18.1 (CH_3), 18.0 (C), –4.2 (CH_3), –4.6 (CH_3); HRMS (ESI): *m/z* calcd for $C_{20}H_{29}NONaSi$ ([M + Na]$^+$) : 350.1916, found 350.1913. The relative stereochemistry was determined by NOESY experiment and coupling constant analysis.

2.3 Diels–Alder reactions with α,β-unsaturated aldehydes

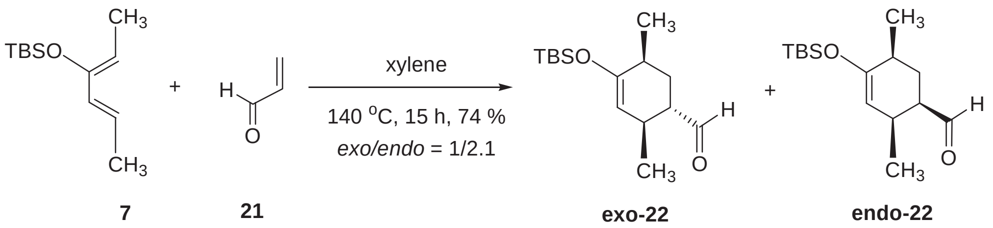

***Rac*-1-[2,5-dimethyl-4-(*tert*-butyldimethylsilyloxy)-cyclohex-3-enyl]-carbaldehydes (exo-22 and endo-22).** In a 25 mL sealed tube equipped with a magnetic stir bar, a solution of freshly distilled acrolein (**21**, 0.396 g, 7.08 mmol, 5.0 equiv.) and diene **7** (0.300 g, 1.20 mmol, 1.0 equiv.) in xylene (2.8 mL) was stirred for 15 h at 140 °C. Afterwards, the mixture was concentrated *in vacuo*. The residue was purified by column chromatography on silica gel (hexane/ethyl acetate = 20/1) to give the corresponding Diels-Alder cycloadducts **exo-22** and **endo-22** (combined: 0.239 g, 74%) as a colorless oil. The diastereoselectivity of the crude products was determined by ^{1}H NMR in $CDCl_3$. Integration of the signal due to the aldehydic proton of **exo-22** (δ_H 9.65 ppm) versus that of the **endo-22** (δ_H 9.72 ppm) gave an *exo*/*endo* ratio of 1/2.1.

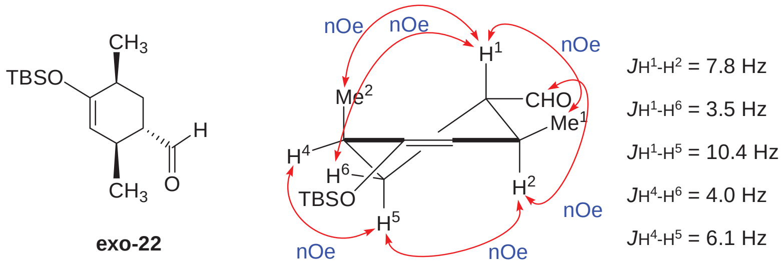

For **exo-22**: IR (thin film): ν 2959, 2931, 2859, 2711, 1727, 1658, 1463, 1362, 1254, 1198, 840, 779 cm^{-1}; ^{1}H NMR (600 MHz, $CDCl_3$): δ 9.65 (d, *J* =2.1 Hz, 1H, CHO), 4.63 (d, *J* = 3.1 Hz, 1H, H^3), 2.57 (dqd, *J* =3.1, 6.8, 7.8 Hz, 1H, H^2), 2.19 (ddq, *J* =4.0, 6.1, 7.1 Hz, 1H, H^4), 2.11 (dddd, *J* =2.1, 3.5, 7.8, 10.4 Hz, 1H, H^1), 1.85 (ddd, 1H, *J* =6.1, 10.4, 13.2 Hz, 1H, H^5), 1.63 (ddd, *J* =3.5, 4.0, 13.2 Hz, 1H, H^6), 1.08 (d, *J* = 7.1 Hz, 3H, Me^2), 1.02 (d, *J* = 6.8 Hz, 3H, Me^1), 0.90 (s, 9H, Si-$C(CH_3)_3$), 0.11 (s, 3H, Si-CH_3), 0.10 (s, 3H, Si-CH_3); ^{13}C NMR (150 MHz, $CDCl_3$): δ 204.9 (CHO), 153.9 (C), 107.7 (CH), 50.3 (CH), 32.1 (CH), 29.5 (CH), 29.4 (CH_2), 25.7 (CH_3 x 3), 21.3 (CH_3), 18.7 (CH_3), 18.1 (C), –4.5 (CH_3), –4.6 (CH_3); HRMS (ESI): *m/z* calcd for $C_{15}H_{29}O_2Si$ ($[M + H]^+$): 269.1937, found 269.1931. The relative stereochemistry was determined by NOESY experiment and coupling constant analysis.

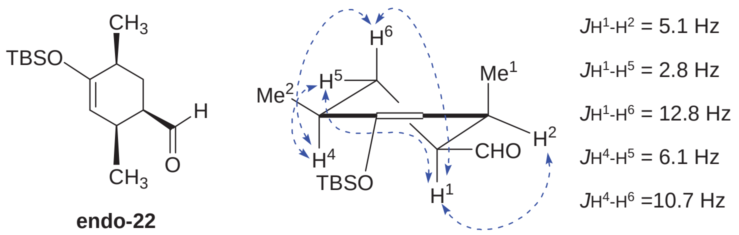

For endo-22: IR (thin film): ν 2958, 2931, 2740, 1724, 1663, 1463, 1361, 1253, 1195, 839, 779 cm^{-1}; ^1H NMR (600 MHz, $CDCl_3$): δ 9.72 (s, 1H, CHO), 4.84 (d, *J* = 5.6 Hz, 1H, H^3), 2.80 (ddq, *J* = 5.1, 5.6, 7.1 Hz, 1H, H^2), 2.56 (ddd, *J* = 2.8, 5.1, 12.8 Hz, 1H, H^1), 2.22 (dqd, *J* = 6.1, 7.0, 10.7 Hz, H^4), 1.98 (ddd, *J* =2.8, 6.1, 13.2 Hz, 1H, H^5), 1.45 (ddd, *J* =10.7, 12.8, 13.2 Hz, 1H, H^6), 1.07 (d, *J* = 7.0 Hz, 3H, Me^2), 0.91 (s, 9H, Si-$C(CH_3)_3$), 0.90 (d, *J* = 7.1 Hz, 3H, Me^1), 0.13 (s, 3H, Si-CH_3), 0.12 (s, 3H, Si-CH_3); ^{13}C NMR (150 MHz, $CDCl_3$): δ 204.6 (CHO), 154.2 (C), 108.5 (CH), 50.5 (CH), 33.9 (CH), 29.1 (CH), 26.5 (CH_2), 25.7 (CH_3 × 3), 18.6 (CH_3), 18.2 (C), 17.5 (CH_3), –4.4 (CH_3), –4.6 (CH_3); HRMS (ESI): *m/z* calcd for $C_{15}H_{29}O_2Si$ ($[M + H]^+$): 269.1937, found 269.1931. The relative stereochemistry was determined by coupling constant analysis.

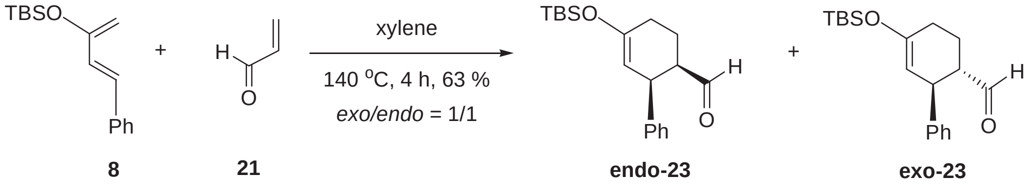

***Rac*-1-[2-phenyl-4-(*tert*-butyldimethylsilyloxy)-cyclohex-3-enyl]-carbaldehydes (exo-23 and endo-23).** In a 25 mL sealed tube equipped with a magnetic stir bar, a solution of freshly distilled acrolein (**21**, 0.388 g, 6.92 mmol, 3.0 equiv.) and diene **8** (0.600 g, 2.31 mmol, 1.0 equiv.) in xylene (4.6 mL) was stirred for 4 h at 140 °C. Afterwards, the mixture was concentrated *in vacuo*. The residue was purified by column chromatography on silica gel (hexanes/ethyl acetate = 20/1) to give the corresponding Diels-Alder cycloadducts **exo-23** and **endo-23** (0.459 g, 63%) as a colorless oil. The diastereoselectivity of the crude products was determined by ^1H NMR in $CDCl_3$. Integration of the signal due to the aldehydic proton of **exo-23** (δ_H 9.66 ppm) versus that of **endo-23** (δ_H 9.47 ppm) gave an *exo*/*endo* ratio of 1/1.

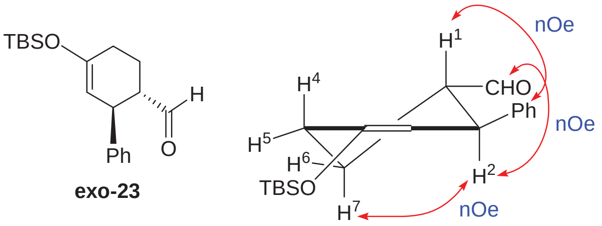

For **exo-23**: IR (thin film): ν 3028, 2956, 2930, 2858, 2711, 1726, 1667, 1602, 1463, 1258, 1203, 838, 779, 701 cm^{-1}; ^{1}H NMR (600 MHz, CDCl$_3$): δ 9.66 (d, *J* = 1.1 Hz, 1H, CHO), 7.29 (m, 2H, Ar-H), 7.21 (m, 3H, Ar-H), 4.87 (ddd, *J* = 1.4, 2.1, 3.6 Hz, 1H, H^3), 3.84 (dd, 1H, *J* = 3.6, 6.7 Hz, 1H, H^2), 2.45 (dddd, *J* = 1.1, 3.6, 6.7, 8.7 Hz, 1H, H^1), 2.18 (dddd, *J* = 2.1, 6.1, 6.1, 17.5 Hz, 1H, H^4), 2.12 (dddd, *J* = 1.4, 6.1, 6.1, 17.5 Hz, 1H, H^5), 1.92 (dddd, *J* = 3.6, 6.1, 6.1, 13.4 Hz, 1H, H^6), 1.83 (dddd, *J* = 6.1, 6.1, 8.7, 13.4 Hz, 1H, H^7), 0.91 (s, 9H, Si-C(CH$_3$)$_3$), 0.14 (s, 3H, Si-CH$_3$), 0.12 (s, 3H, Si-CH$_3$); ^{13}C NMR (150 MHz, CDCl$_3$): δ 203.8 (CHO), 151.7 (C), 144.3 (C), 128.5 (CH × 2), 128.2 (CH × 2), 126.7 (CH), 106.1 (CH), 53.7 (CH), 41.1 (CH), 27.9 (CH$_2$), 25.6 (CH$_3$ × 3), 20.9 (CH$_2$), 18.0 (C), –4.4 (CH$_3$ × 2); HRMS (ESI): *m/z* calcd for $C_{19}H_{27}O_2Si$ ([M + H]$^+$): 315.1780, found 315.1776. The relative stereochemistry was determined by NOESY experiment analysis.

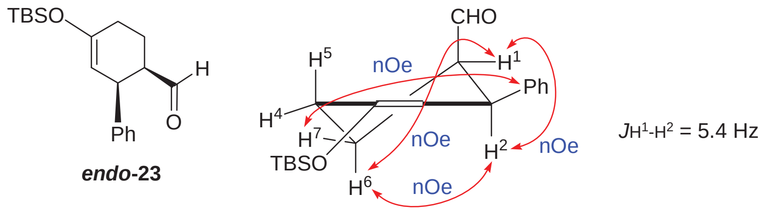

For **endo-23**: IR (thin film): ν 3030, 2955, 2930, 2857, 2710, 1721, 1668, 1602, 1462, 1254, 1197, 839, 780, 701 cm^{-1}; ^{1}H NMR (600 MHz, CDCl$_3$): δ 9.47 (d, *J* = 1.8 Hz, 1H, CHO), 7.28 (m, 2H, Ar-H), 7.21 (m, 3H, Ar-H), 5.02 (ddd, *J* = 1.5, 1.5, 4.5 Hz, 1H, H^3), 4.04 (dd, 1H, *J* = 4.5, 5.4 Hz, 1H, H^2), 2.68 (dddd, *J* = 1.8, 3.6, 5.4, 10.0 Hz, 1H, H^1), 2.23 (dddd, *J* = 1.5, 6.1, 6.4, 17.8 Hz, 1H, H^4), 2.16 (dddd, *J* = 1.5, 6.7, 8.7, 17.8 Hz, 1H, H^5),1.94 (dddd, *J* = 6.4, 8.7, 10.0, 13.7 Hz, 1H, H^6), 1.85 (dddd, *J* = 3.6, 6.1, 6.7, 13.7 Hz, 1H, H^7), 0.93 (s, 9H, Si-C(CH$_3$)$_3$), 0.16 (s, 3H, Si-CH$_3$), 0.16 (s, 3H, Si-CH$_3$); ^{13}C NMR (150 MHz, CDCl$_3$) δ 204.6 (CHO), 152.4 (C), 141.0(C), 129.0 (CH × 2), 128.4 (CH × 2), 127.0 (CH), 105.7 (CH), 50.5 (CH), 41.3 (CH), 28.1 (CH$_2$), 25.6 (CH$_3$ × 3), 19.5 (CH$_2$), 18.0 (C), –4.3 (CH$_3$ × 2); HRMS (ESI): *m/z* calcd for $C_{19}H_{28}O_2NaSi$ ([M + Na]$^+$): 339.1756, found 339.1755. The relative stereochemistry was determined by NOESY experiment and coupling constant analysis.

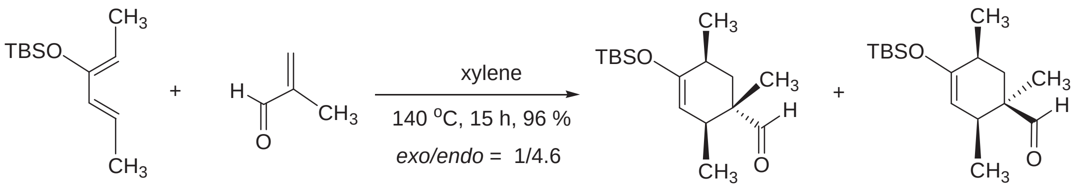

7 **24** **exo-25** **endo-25**

Rac-1-[1,2,5-trimethyl-4-(_tert_-butyldimethylsilyloxy)-cyclohex-3-enyl]-carbaldehydes (exo-25 and endo-25). In a 25 mL sealed tube equipped with a magnetic stir bar, a solution of freshly distilled acrolein (**24**, 0.297 g, 4.25 mmol, 3.0 equiv.) and diene **7** (0.300 g, 1.20 mmol, 1.0 equiv.) in xylene (2.8 mL) was stirred for 15 h at 140 °C. Afterwards, the mixture was concentrated *in vacuo*. The residue was purified by column chromatography on silica gel (hexanes/ethyl acetate = 20/1) to give the corresponding Diels-Alder cycloadducts **exo-25** and **endo-25** (combined: 0.326 g, 96%) as a colorless oil. The diastereoselectivity of the crude products was determined by ^{1}H NMR in C_6D_6. Integration of the signal due to the aldehydic proton of **exo-25** (δ_H 9.27 ppm) versus that of **endo-25** (δ_H 9.31 ppm) gave an *exo*/*endo* ratio of 1/4.6.

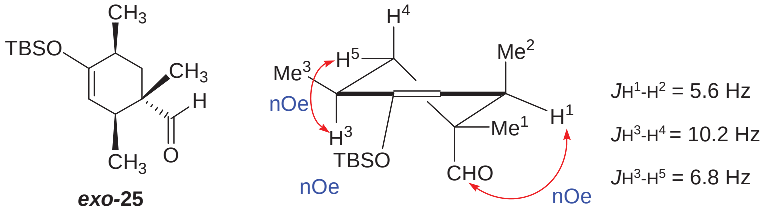

For **exo-25**: IR (thin film): ν 2959, 2932, 2859, 2694, 1729, 1659, 1463, 1361, 1254, 1201, 841, 779 cm^{-1}; ^{1}H NMR (600 MHz, C_6D_6): δ 9.27 (s, 1H, CHO), 4.85 (dd, *J* =1.4, 5.6 Hz, 1H, H^2), 2.24 (ddqd, *J* =1.4, 6.8, 6.9, 10.2 Hz, 1H, H^3), 2.18 (dq, *J* =5.6, 7.0 Hz, 1H, H^1), 1.73 (dd, *J* = 6.8, 13.5 Hz, 1H, H^5), 1.08 (dd, *J* =10.2, 13.5 Hz, 1H, H^4),1.04 (d, *J* = 6.9 Hz, 3H, Me^3), 0.95 (s, 9H, Si-C(CH_3)$_3$), 0.75 (d, *J* = 7.0 Hz, 3H, Me^2), 0.62 (s, 3H, Me^1), 0.11 (s, 3H, Si-CH_3), 0.08 (s, 3H, Si-CH_3); ^{13}C NMR (150 MHz, C_6D_6): δ 204.8 (CHO), 154.5 (C), 109.1 (CH), 48.0 (C), 35.0 (CH_2), 33.4 (CH), 32.1 (CH), 25.9 (CH_3 × 3), 19.9 (CH_3), 19.0 (CH_3), 18.2 (CH_3), 17.1 (C), -4.3(CH_3), -4.8 (CH_3); HRMS (APCI): *m/z* calcd for $C_{16}H_{31}O_2Si$ ([M + H]$^+$): 283.2093, found 283.2091. The relative stereochemistry was determined by NOESY experiment and coupling constant analysis.

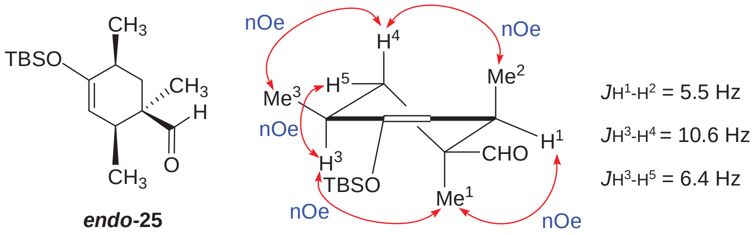

For **endo-25**: IR (thin film): ν 2959, 2932, 2859, 2691, 1729, 1660, 1464, 1361, 1254, 1201, 840, 779 cm^{-1}; ^{1}H NMR (600 MHz, C_6D_6): δ 9.31 (s, 1H, CHO),4.66 (dd, *J* =1.5, 5.5 Hz, 1H, H^2), 2.00 (ddqd, *J* =1.5, 6.4, 6.9, 10.6 Hz, 1H, H^3), 1.90 (dq, *J* =5.5, 6.9 Hz, 1H, H^1), 1.57 (dd, *J* =10.6, 13.8 Hz, 1H, H^4) , 1.46 (dd, *J* = 6.4, 13.8 Hz, 1H, H^5), 1.06 (d, *J* = 6.9 Hz, 3H, Me^3),

0.96 (s, 9H, Si-C(CH_3)$_3$), 0.88 (s, 3H, Me^1), 0.78 (d, J = 6.9 Hz, 3H, Me^2), 0.09 (s, 3H, Si-CH_3), 0.08 (s, 3H, Si-CH_3); ^{13}C NMR (150 MHz, C_6D_6): δ 204.7 (CHO), 153.2 (C), 107.0 (CH), 47.3 (C), 35.9 (CH), 32.7 (CH_2), 31.1 (CH), 25.9 (CH_3 × 3), 19.6 (CH_3), 18.9 (C), 18.7 (CH_3), 18.3 (CH_3), –4.3(CH_3), –4.6 (CH_3); HRMS (APCI): *m/z* calcd for $C_{16}H_{31}O_2Si$ ([M + H]$^+$): 283.2093, found 283.2090. The relative stereochemistry was determined by NOESY experiment and coupling constant analysis.

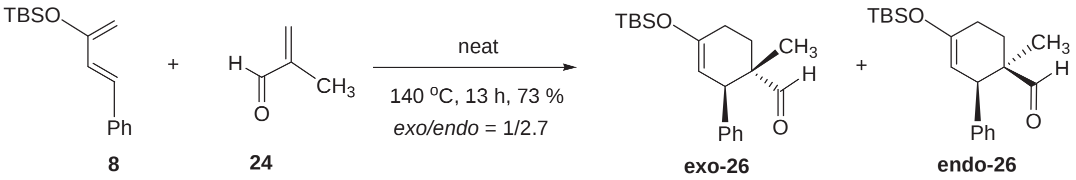

***Rac*-1-[1-methyl-2-phenyl-4-(*tert*-butyldimethylsilyloxy)-cyclohex-3-enyl]-carbaldehydes (exo-26 and endo-26).** In a 25 mL sealed tube equipped with a magnetic stir bar, freshly distilled methylacrolein (**24**, 0.485 g, 6.92 mmol, 3.0 equiv.) and diene **8** (0.600 g, 2.31 mmol, 1.0 equiv.) were stirred for 13 h at 140 °C. Afterwards, the mixture was concentrated *in vacuo*. The residue was purified by column chromatography on silica gel (hexane/ethyl acetate = 20/1) to give the corresponding Diels–Alder cycloadducts **exo-26** and **endo-26** (combined: 0.556 g, 73%) as a colorless oil. The diastereoselectivity of the crude products was determined by ^{1}H NMR in $CDCl_3$. Integration of the signal due to the aldehydic proton of **exo-26** (δ_H 9.55 ppm) versus that of **endo-26** (δ_H 9.37 ppm) gave an crude *exo*/*endo* ratio of 1/2.7.

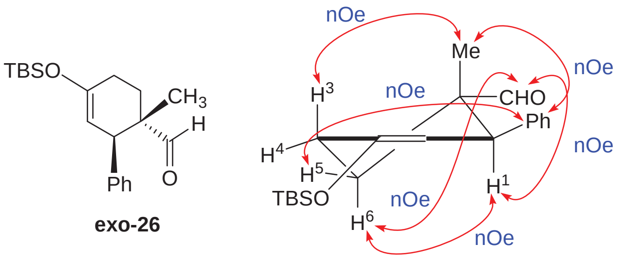

For **exo-26**: IR (thin film): ν 3024, 2952, 2930, 2858, 2710, 1724, 1667, 1600, 1460, 1367, 1257, 1206, 838, 779, 703 cm^{-1}; ^{1}H NMR (600 MHz, $CDCl_3$): δ 9.55 (s, 1H, CHO), 7.28 (dd, J= 7.2, 7.4 Hz, 2H, Ar-H), 7.22 (d, J= 7.2 Hz, 1H Ar-H), 7.15 (d, J= 7.4 Hz, 2H Ar-H), 4.94 (ddd, J = 1.3, 1.4, 4.2 Hz, 1H, H^2), 3.74 (d, J = 4.2 Hz, 1H, H^1), 2.15 (m, 2H, H^3 and H^4), 1.85 (ddd, J = 6.2, 7.5, 13.9 Hz, 1H, H^6), 1.61 (ddd, J = 7.5, 7.7, 13.4 Hz, 1H, H^5), 0.90 (s, 9H, Si-C(CH_3)$_3$), 0.69 (s, 3H, Me), 0.13 (s, 3H, Si-CH_3), 0.11 (s, 3H, Si-CH_3); ^{13}C NMR (150 MHz, $CDCl_3$): δ 205.7 (CHO), 151.8 (C), 141.0 (C), 129.7 (CH × 2), 128.0 (CH × 2), 126.8 (CH), 106.1 (CH), 48.4 (C), 45.2 (CH), 26.8 (CH_2), 26.6 (CH_2), 25.6 (CH_3 × 3), 18.8 (CH_3),

18.0 (C), –4.3 (CH_3), –4.5 (CH_3); HRMS (APCI): *m/z* calcd for $C_{20}H_{31}O_2Si$ ($[M + H]^+$): 331.2093, found 331.2090. The relative stereochemistry was determined by NOESY experiment analysis.

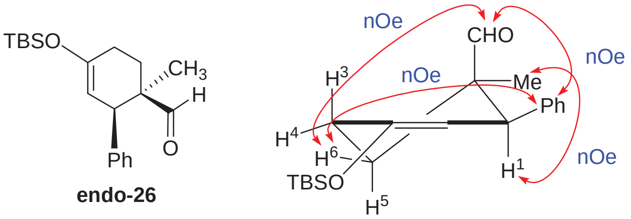

For **endo-26**: IR (thin film): ν 3024, 2952, 2930, 2858, 2710, 1724, 1667, 1602, 1458, 1367, 1257, 1207, 838, 779, 703 cm^{-1}; ^1H NMR (600 MHz, $CDCl_3$): δ 9.37 (s, 1H, CHO),7.27 (dd, *J* = 7.3, 7.3 Hz, 2H, Ar-H), 7.21 (d, *J* = 7.3 Hz, 1H, Ar-H), 7.17 (d, *J*= 7.3 Hz, 2H, Ar-H), 4.91 (d, *J* = 4.2 Hz, 1H, H^2), 3.49 (d, *J* = 4.2 Hz, 1H, H^1), 2.30 (ddd, *J* = 6.2, 6.5, 17.9 Hz, 1H, H^3), 2.15 (ddd, *J* = 7.0, 7.2, 17.9 Hz, 1H, H^4), 2.02 (ddd, *J* = 6.5, 7.2, 13.8 Hz, 1H, H^5), 1.56–1.53 (m, 1H, H^6), 1.16 (s, 3H, Me), 0.92 (s, 9H, Si-C(CH_3)$_3$), 0.16 (s, 3H, Si-CH_3), 0.15 (s, 3H, Si-CH_3); ^{13}C NMR (150 MHz, $CDCl_3$): δ 206.2 (CHO), 151.3 (C), 140.9 (C), 129.4 (CH × 2), 128.3 (CH × 2), 127.1 (CH), 105.5 (CH), 49.0 (CH), 47.3 (C), 27.1 (CH_2), 26.5 (CH_2), 25.7 (CH_3 × 3), 20.6 (CH_3), 18.0 (C), –4.3 (CH_3), –4.4 (CH_3); HRMS (ESI): *m/z* calcd for $C_{20}H_{30}O_2NaSi$ ($[M + Na]^+$): 353.1913, found 353.1906. The relative stereochemistry was determined by NOESY experiment analysis.

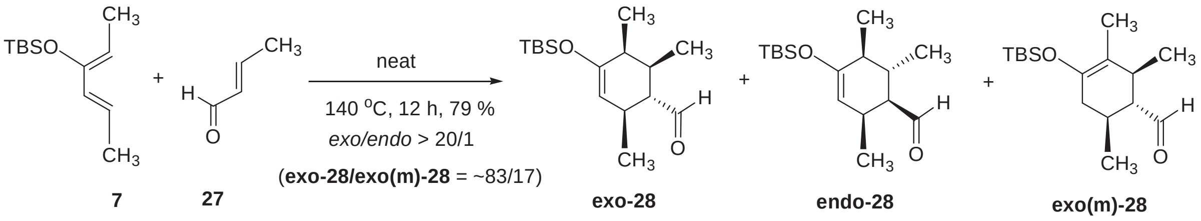

***Rac*-1-[2,5,6,-trimethyl-4-(*tert*-butyldimethylsilyloxy)-cyclohex-3-enyl]-carbaldehydes (exo-28, endo-28) and the corresponding *exo* olefin migrator exo(m)-28.** In a 25 mL sealed tube equipped with a magnetic stir bar, freshly distilled aldehyde **27** (0.297 g, 4.25 mmol, 3.0 equiv.) and diene **7** (0.300 g, 1.20 mmol, 1.0 equiv.) was stirred for 12 h at 140 °C. Afterwards, the mixture was concentrated *in vacuo*. The residue was purified by column chromatography on silica gel (hexanes/ethyl acetate = 20/1) to give the corresponding Diels–Alder cycloadducts **exo-28**, **endo-28** and **exo(m)-28** (combined: 0.268 g, 79%) as a colorless oil. The diastereoselectivity of the crude products was determined by ^1H NMR in C_6D_6. Integration of the signal due to the aldehydic proton of **exo-28** (δ_H 9.27 ppm) versus

that of the corresponding *exo* olefin migrator **exo(m)-28** (δ_H 9.23 ppm) gave a *exo*/*exo* olefin migrator ratio of ~83/17 (the signals corresponding to **endo-28** are barely visible).

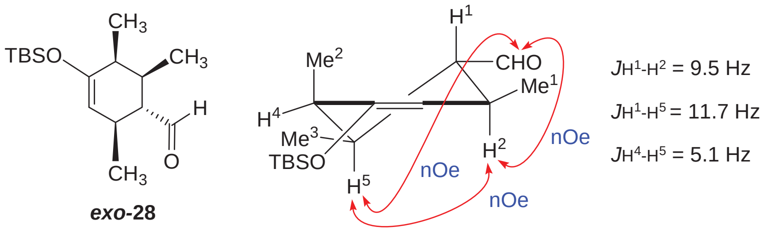

For **exo-28**: m.p. 38–40 °C; IR (thin film): ν 2960, 2930, 2859, 2701, 1728, 1668, 1361, 1254, 1198, 838, 779 cm^{-1}; ^1H NMR (600 MHz, C_6D_6): δ 9.27 (d, *J* = 4.7Hz, 1H, CHO), 4.57 (d, *J* = 2.1 Hz, 1H, H^3), 2.29 (dqd, *J* = 2.1, 7.1, 9.5 Hz, 1H, H^2), 1.92 (dq, *J* = 5.1, 7.1 Hz, 1H, H^4), 1.83 (dqd, *J* = 5.1, 6.8, 11.7 Hz, 1H, H^5), 1.76 (ddd, *J* =4.7, 9.5, 11.7 Hz, 1H, H^1), 0.97 (s, 9H, Si-C(CH_3)$_3$), 0.85 (d, *J* = 7.1 Hz, 3H, Me^2), 0.82 (d, *J* = 7.0 Hz, 3H, Me^1), 0.67 (d, *J* = 6.8 Hz, 3H, Me^3), 0.12 (s, 3H, Si-CH_3), 0.11 (s, 3H, Si-CH_3); ^{13}C NMR (150 MHz, C_6D_6): δ 203.8 (CHO), 154.7 (C), 106.6 (CH), 55.7 (CH), 38.9 (CH), 32.9 (CH), 30.0(CH), 25.8 (CH_3×3), 21.0 (CH_3), 18.2 (C), 16.3 (CH_3), 13.0 (CH_3), –4.2 (CH_3), –4.6 (CH_3); HRMS (ESI): *m/z* calcd for $C_{16}H_{31}O_2Si$ ($[M + H]^+$): 283.2093, found 283.2094. The relative stereochemistry was determined by NOESY experiment and coupling constant analysis.

For **exo(m)-28**: IR (thin film): ν 2958, 2931, 2858, 2700, 1728, 1683, 1463, 1356, 1254, 1199, 837, 779 cm^{-1}; ^1H NMR (600 MHz, C_6D_6): δ 9.23 (d, *J* = 4.5 Hz, 1H, CHO), 2.13–2.11 (m, 1H, H^5), 1.94–1.92 (m, 1H, H^3), 1.65 (m, 3H, H^1, H^2 and H^4), 1.58 (s, 3H, Me^2), 0.99 (s, 9H, Si-C(CH_3)$_3$), 0.84 (d, *J* = 6.8 Hz, 3H, Me^3), 0.70 (d, *J* = 5.6 Hz, 3H, Me^1), 0.06 (s, 3H, Si-CH_3), 0.06 (s, 3H, Si-CH_3); ^{13}C NMR (150 MHz, C_6D_6): δ 203.5 (CHO), 142.7 (C), 113.4 (C), 62.0 (CH), 38.5 (CH_2), 33.8 (CH), 29.2(CH), 26.0 (CH_3 × 3), 19.4 (CH_3), 18.8 (CH_3), 18.4 (C), 13.6 (CH_3), –3.7 (CH_3), –4.0 (CH_3); HRMS (ESI): *m/z* calcd for $C_{16}H_{31}O_2Si$ ($[M+ H]^+$): 283.2093, found 283.2088.

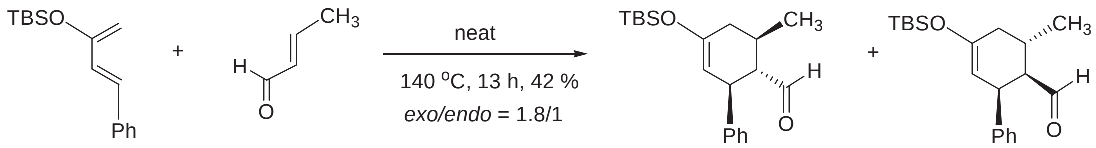

8 **27** **exo-29** **endo-29**

***Rac*-1-[6-methyl-2-phenyl-4-(*tert*-butyldimethylsilyloxy)-cyclohex-3-enyl]-carbaldehydes (exo-29 and endo-29).** In a 25 mL sealed tube equipped with a magnetic stir bar, aldehyde **27** (0.485 g, 6.923 mmol, 3.0 equiv.) and diene **8** (0.600 g, 2.308 mmol, 1.0 equiv.) were stirred for 13 h at 140 °C. Afterwards, the mixture was concentrated *in vacuo*.

The residue was purified by column chromatography on silica gel (hexanes/ethyl acetate = 20/1) to give the corresponding Diels-Alder cycloadducts **exo-29** and **endo-29** (combined: 0.320 g, 42%) as a colorless oil. The diastereoselectivity of the crude products was determined by ^{1}H NMR in C_6D_6. Integration of the signal due to the aldehydic proton of **exo-29** (δ_H 9.33 ppm) versus that of **endo-29** (δ_H 9.09 ppm) gave an *exo*/*endo* ratio of 1.8:1.

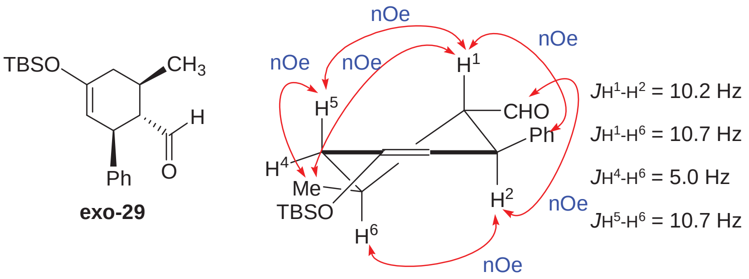

exo-29

For **exo-29**: IR (thin film): ν 3029, 2957, 2930, 2858, 2711, 1728, 1669, 1600, 1462, 1367, 1253, 1203, 838, 800, 700 cm^{-1}; ^{1}H NMR (600 MHz, C_6D_6): δ 9.33 (d, *J* =3.9 Hz, 1H,CHO), 7.11 (dd, *J* = 7.1, 7.2 Hz, 4H, Ar-H), 7.03 (d, *J* = 7.1 Hz, 1H, Ar-H), 4.86 (dd, *J* = 2.0, 2.1 Hz, 1H, H^3), 3.65 (ddd, *J* = 2.0, 3.6, 10.2 Hz, 1H, H^2), 2.08 (ddd, *J* = 3.9, 10.2, 10.7 Hz, 1H, H^1), 2.01 (ddd, *J* = 2.0, 5.0, 16.6 Hz, 1H, H^4), 1.87 (dqdd, *J* = 5.0, 6.4, 10.7, 10.7 Hz, 1H, H^6), 1.78 (dddd, *J* = 2.1, 3.5, 10.7, 16.6 Hz, 1H, H^5), 0.96 (s, 9H, Si-C(CH_3)$_3$), 0.71 (d, *J* = 6.4Hz, 1H, Me), 0.10 (s, 3H, Si-CH_3), 0.07 (s, 3H, Si-CH_3); ^{13}C NMR (150 MHz, C_6D_6): δ 203.5 (CHO), 150.5 (C), 144.5 (C), 128.9 (CH × 2), 128.3 (CH × 2), 127.1 (CH), 106.9 (CH), 61.5 (C), 43.4 (CH), 38.2 (CH_2), 30.7 (CH), 25.8 (CH_3 × 3), 19.3 (CH_3), 18.1 (C), –4.3 (CH_3), –4.4 (CH_3); HRMS (ESI): *m/z* calcd for $C_{20}H_{30}O_2NaSi$ ([M+ Na]$^+$): 353.1913, found 353.1910. The relative stereochemistry was determined by NOESY experiment and coupling constant analysis.

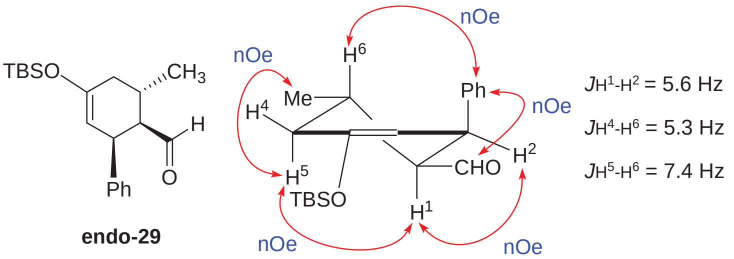

endo-29

For **endo-29**: IR (thin film): ν 3024, 2952, 2929, 2857, 2724, 1723, 1671, 1600, 1460, 1369, 157, 1205, 837, 779, 701 cm^{-1}; ^{1}H NMR (600 MHz, C_6D_6): δ 9.09 (d, *J* =3.6 Hz, 1H, CHO), 7.11–7.10 (m, 4H, Ar-H), 7.05–7.02 (m, 1H, Ar-H), 4.92 (d, *J* = 4.5 Hz, 1H, H^3), 3.69 (dd, *J* = 4.5, 5.6 Hz, 1H, H^2), 2.21 (dd, *J* = 5.3, 16.9 Hz, 1H, H^4), 2.14 (m, 2H, H^1 and H^6), 1.64 (dd, *J* = 16.9, 7.4 Hz, 1H, H^5), 0.97 (s, 9H, Si-C(CH_3)$_3$), 0.67 (d, *J* = 5.9 Hz, 1H, Me), 0.11 (s, 3H, Si-CH_3), 0.10 (s, 3H, Si-CH_3); ^{13}C NMR (150 MHz, C_6D_6) δ 204.4 (CHO), 151.5 (C), 141.0

(C), 129.7 (CH × 2), 128.6 (CH × 2), 127.1 (CH), 105.2 (CH), 56.4 (C), 41.7 (CH), 36.9 (CH_2), 25.8 (CH_3 × 3), 25.2 (CH), 19.4 (CH_3), 18.1 (C), –4.2 (CH_3), –4.4 (CH_3); HRMS (ESI): *m/z* calcd for $C_{20}H_{30}O_2NaSi$ ($[M + Na]^+$): 353.1913, found 353.1907. The relative stereochemistry was determined by NOESY experiment and coupling constant analysis.

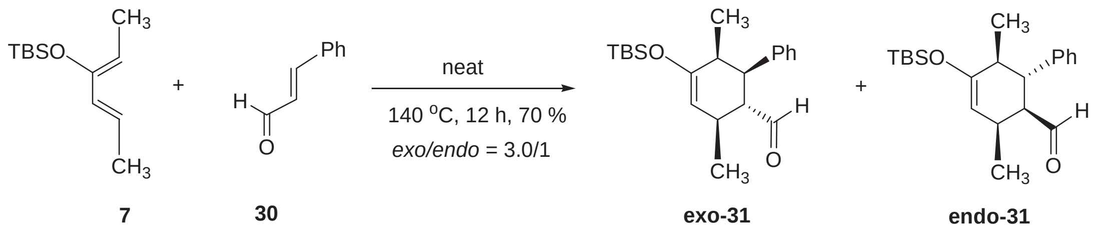

***Rac*-1-[2,5-dimethyl-6-phenyl-4-(*tert*-butyldimethylsilyloxy)-cyclohex-3-enyl]-carbaldehydes (exo-31 and endo-31)**. In a 25 mL sealed tube equipped with a magnetic stir bar, aldehyde **30** (0.373 g, 2.83 mmol, 2.0 equiv.) and diene **7** (0.300 g, 1.20 mmol, 1.0 equiv.) were stirred for 12 h at 140 °C. Afterwards, the mixture was concentrated *in vacuo*. The residue was purified by column chromatography on silica gel (hexanes/ethyl acetate = 20/1) to give the corresponding Diels-Alder cycloadducts **exo-31** and **endo-31** (combined: 0.289 g, 70%) as a colorless oil. The diastereoselectivity of the crude products was determined by ^1H NMR in $CDCl_3$. Integration of the signal due to the aldehydic proton of the major adduct **exo-31** (δ_H 9.29 ppm) versus that of the minor adduct **endo-31** (δ_H 9.40 ppm) gave an *exo*/*endo* ratio of 3.0/1.

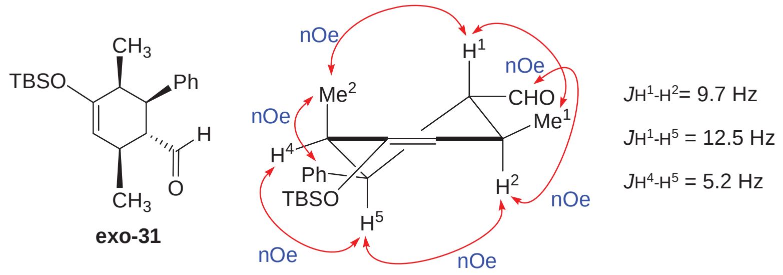

For **exo-31**: m.p. 68–70 °C; IR (thin film): ν 3029, 2958, 2931, 2858, 2705, 1726, 1667, 1602, 1359, 1254, 1181, 841, 779, 701 cm^{-1}; ^1H NMR (600 MHz, C_6D_6): δ 9.13 (d, J = 5.0 Hz, 1H, CHO), 7.08 (dd, J= 7.6, 7.4 Hz, 2H, Ar-H) , 7.00 (dd, J = 7.6, 7.4 Hz, 1H, Ar-H), 6.95 (d, J = 7.6 Hz, 1H, Ar-H), 4.65 (d, J = 2.3 Hz, 1H, H^3), 3.16 (dd J = 5.2, 12.5 Hz, 1H, H^5), 2.50 (ddd, J = 5.0, 9.7, 12.5 Hz, 1H, H^1), 2.31 (dqd, J = 2.3, 6.8, 9.7 Hz 1H, H^2), 2.23 (dq, J = 5.2, 7.1, 1H, H^4), 0.98 (d, J = 6.8Hz, 3H, Me^1), 0.96 (s, 9H, Si-C(CH_3)$_3$), 0.76 (d, J = 7.1 Hz, 3H, Me^2), 0.16 (s, 3H, Si-CH_3), 0.13 (s, 3H, Si-CH_3); ^{13}C NMR (150 MHz, C_6D_6): δ 202.3 (CHO), 154.5 (C), 140.4 (C), 129.2 (CH × 2), 128.6 (CH × 2), 126.9 (CH), 106.5 (CH), 52.3 (CH),

45.3 (CH), 40.2 (CH), 30.3 (CH), 25.8 (CH_3 × 3), 20.8 (CH_3), 18.2 (C), 14.3 (CH_3), –4.1 (CH_3), –4.7 (CH_3); HRMS (ESI): *m/z* calcd for $C_{21}H_{33}O_2Si$ ($[M+H]^+$): 345.2250, found 345.2249. The relative stereochemistry was determined by single-crystal X-ray diffraction data (racemic mixture), NOESY experiment and coupling constant analysis.

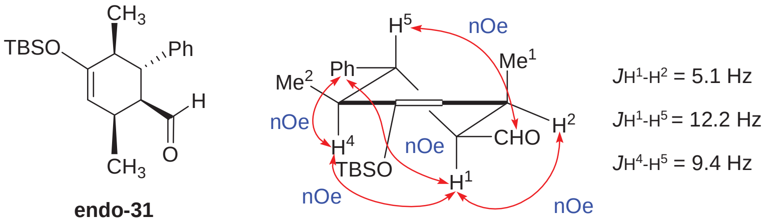

endo-31

For **endo-31**: IR (thin film): ν 3029, 2959, 2931, 2859, 2705, 1726, 1667, 1062, 1472, 1359, 1254, 1194, 838, 779, 701 cm^{-1}; ^1H NMR (600 MHz, C_6D_6): δ 9.35 (d, *J* = 3.2 Hz, 1H, CHO), 7.10–6.93 (m, 5H, Ar-H), 4.88 (dd, *J* = 1.5, 5.7Hz, 1H, H^3), 2.84 (dd, *J* =9.4, 12.2 Hz, 1H, H^5), 2.74 (ddd, *J* = 3.2, 5.1, 12.2 Hz, 1H, H^1), 2.58 (ddq, *J* = 5.1, 5.7, 7.0 Hz, 1H, H^2), 2.23 (qd, *J* = 6.8, 9.4 Hz, 1H, H^4), 1.02 (d, *J* = 6.8 Hz, 3H, Me^2), 0.96 (s, 9H, Si-$C(CH_3)_3$), 0.92 (d, *J* = 7.0Hz, 3H, Me^1), 0.14 (s, 3H, Si-CH_3) , 0.12 (s, 3H, Si-CH_3); ^{13}C NMR (150 MHz, C_6D_6) δ 203.7 (CHO), 153.4 (C), 142.8 (C), 128.9 (CH×2), 128.8 (CH × 2), 127.0 (CH), 108.4 (CH), 54.9 (CH), 45.2 (CH), 42.2 (CH), 30.8 (CH), 25.9 (CH_3 × 3), 18.3 (C), 17.5 (CH_3), 16.4 (CH_3), –4.3 (CH_3), –4.6 (CH_3); HRMS (ESI): *m/z* calcd for $C_{21}H_{33}O_2Si$ ($[M+H]^+$): 345.2250, found 345.2258. The relative stereochemistry was determined by NOESY experiment and coupling constant analysis.

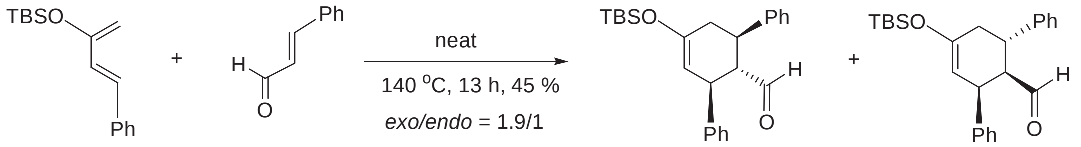

8 **30** **exo-32** **endo-32**

***Rac*-1-[2,6-diphenyl-4-(*tert*-butyldimethylsilyloxy)–cyclohex-3-enyl]-carbaldehydes (exo-32 and endo-32).** In a dry 25 mL sealed tube equipped with a magnetic stir bar, aldehyde **30** (0.609 g, 4.615 mmol, 2.0 equiv.) and diene **8** (0.600 g, 2.308 mmol, 1.0 equiv.) were stirred for 13 h at 140 °C. Afterwards, the mixture was concentrated *in vacuo*. The residue was purified by column chromatography on silica gel (hexane/ethyl acetate = 20/1) to give the corresponding Diels-Alder cycloadducts **exo-32** and **endo-32** (combined: 0.407 g, 45%) as a colorless oil. The diastereoselectivity of the crude products was determined by ^1H NMR in $CDCl_3$. Integration of the signal due to the aldehydic proton of the major adduct

exo-32 (δ_H 9.33 ppm) versus that of the minor adduct **endo-32** (δH 8.90 ppm) gave an *exo*/*endo* ratio of 1.9:1.

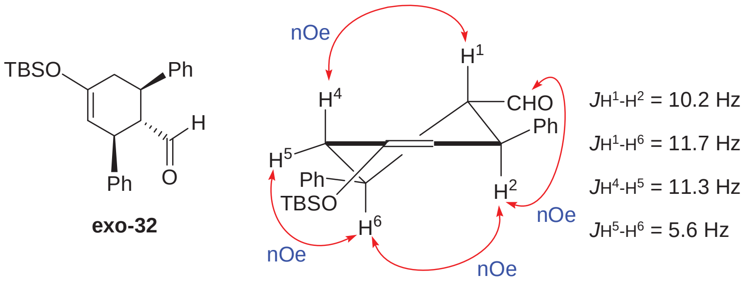

For **exo-32**: m.p. 56–58 °C; IR (thin film): ν 3028, 2955, 2929, 2857, 2734, 1724, 1700, 1601, 1470, 1253, 1205, 837, 779, 701 cm^{-1}; ^{1}H NMR (600 MHz, $CDCl_3$): δ 9.33 (d, *J* = 3.6 Hz, 1H, CHO), 7.28–7.26 (m, 4H, Ar-H), 7.21–7.19 (m, 6H, Ar-H), 4.89 (d, *J* = 2.0 Hz, 1H, H^3), 3.90 (dd, *J* = 2.0, 10.2 Hz, 1H, H^2), 3.27 (ddd, *J* = 5.6, 11.3, 11.7 Hz, 1H, H^6), 2.93 (ddd, *J* = 3.6, 10.2, 11.7 Hz, 1H, H^1), 2.47 (dd, *J* =11.3, 17.4 Hz, 1H, H^4), 2.36 (dd, *J* = 5.6, 17.4 Hz, 1H, H^5), 0.91 (s, 9H, Si-C(CH_3)$_3$), 0.17 (s, 3H, Si-CH_3), 0.15 (s, 3H, Si-CH_3); ^{13}C NMR (150 MHz, $CDCl_3$): δ 204.1 (C), 150.1 (C), 143.6 (C), 141.6 (C), 128.9 (CH × 2), 128.6 (CH × 2), 128.1 (CH × 2), 127.5 (CH × 2), 127.1 (CH), 126.9 (CH), 107.0 (CH), 59.7 (CH), 43.2 (CH × 2), 38.7 (CH_2), 25.6 (CH_3 × 3), 18.0 (C), –4.3 (CH_3), –4.5 (CH_3); HRMS (ESI): *m/z* calcd for $C_{25}H_{33}O_2Si$ ([M + H]$^+$): 393.2250, found 393.2255. The relative stereochemistry was determined by NOESY experiment and coupling constant analysis.

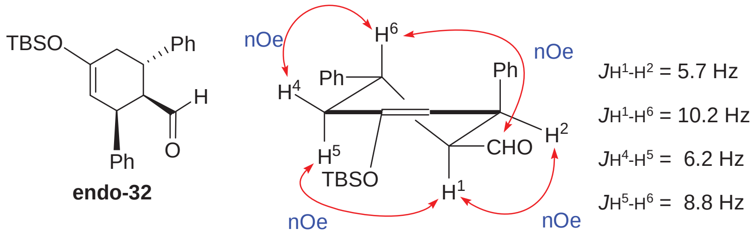

For **endo-32**: IR (thin film): ν 3029, 2955, 2929, 2857, 2730, 1724, 1668, 1602, 1460, 1369, 1253, 1205, 800, 700 cm^{-1}; ^{1}H NMR (600 MHz, $CDCl_3$): δ 8.90 (d, *J* =4.1Hz, 1H,CHO), 7.31–7.29 (m, 2H, Ar-H), 7.26–7.23 (m, 3H, Ar-H), 7.22–7.19 (m, 2H, Ar-H), 7.18–7.16 (m, 1H, Ar-H), 7.15–7.14 (m, 2H, Ar-H), 5.06 (d, *J* = 4.4 Hz, 1H, H^3), 3.91 (dd, *J* = 4.4, 5.7 Hz, 1H, H^2), 3.48 (ddd, *J* = 6.2, 8.8, 10.2 Hz, 1H, H^6), 2.88 (ddd, *J* = 4.1, 5.7, 10.2 Hz, 1H, H^1), 2.52 (dd, *J* = 6.2, 17.9 Hz, 1H, H^4), 2.30 (dd, *J* = 8.8, 17.9 Hz, 1H, H^5), 0.93 (s, 9H, Si-C(CH_3)$_3$), 0.19 (s, 3H, Si-CH_3), 0.19 (s, 3H, Si-CH_3); ^{13}C NMR (150 MHz, $CDCl_3$): δ 205.9 (C), 151.6 (C), 142.5 (C), 140.0 (C), 129.6 (CH × 2), 128.8 (CH × 2), 128.5 (CH × 2), 127.7 (CH × 2), 127.1 (CH), 127.0 (CH), 105.3 (CH), 55.2 (CH), 41.7 (CH), 36.9 (CH_2), 36.9 (CH), 25.6 (CH_3 × 3), 18.0 (C), –4.3 (CH_3), –4.4 (CH_3); HRMS (ESI): *m/z* calcd for $C_{25}H_{33}O_2Si$

([M + H]$^+$): 393.2250, found 393.2252. The relative stereochemistry was determined by NOESY experiment and coupling constant analysis.

2.4 Diels–Alder reactions with α,β-unsaturated ketones

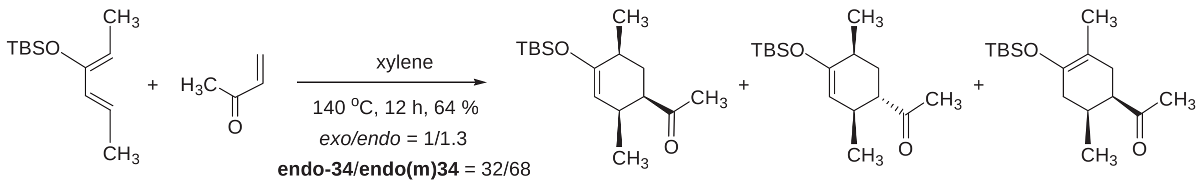

7 **33** **endo-34** **exo-34** **endo(m)-34**

***Rac*-1-[2,5-dimethyl-4-(*tert*-butyldimethylsilyloxy)-cyclohex-3-enyl]-ethan-1-ones (exo-34 and endo-34) and the corresponding *endo* olefin migrator endo(m)-34.** In a 25 mL sealed tube equipped with a magnetic stir bar, a solution of freshly distilled methylvinyl ketone (**33**, 0.590 mL, 7.075 mmol, 5.0 equiv.), diene **7** (0.300 g, 1.2 mmol, 1.0 equiv.) in xylene (2.8 mL) was stirred for 12 h at 140 °C. Afterwards, the mixture was concentrated *in vacuo*. The residue was purified by column chromatography on silica gel (hexanes/ethyl acetate = 20/1) to give the corresponding Diels-Alder cycloadducts **exo-34, endo-34** and **endo(m)-34** (combined: 0.217 g, 64%) as a colorless oil. The diastereoselectivity of the crude products was determined by ^{1}H NMR in C_6D_6. Integration of the signal due to the vinylic proton (H^3) of **exo-34** (δ_H 4.65 ppm) versus that of **endo-34** (δ_H 4.78 ppm) and H^1 of the corresponding *endo* olefin migrator **endo(m)-34** (δ_H 2.16 ppm) gave an apparent *exo*/*endo* ratio of 1/1.3 (the **endo-34**/**endo(m)-34** ratio is 32/68).

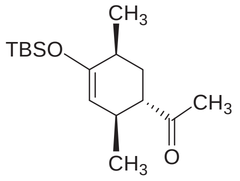

exo-34

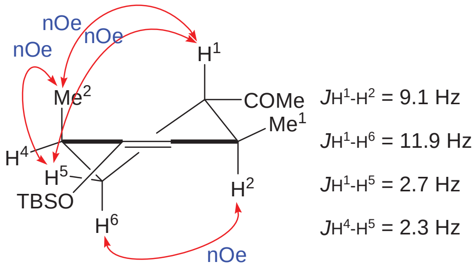

For **exo-34**: IR (thin film): ν 3406, 2957, 2931, 2860, 1716, 1657, 1465, 1371, 1257, 1173, 843, 776 cm^{-1}; ^{1}H NMR (600 MHz, C_6D_6): δ 4.65 (d, *J* = 2.4 Hz, 1H, H^3), 2.69 (dqd, *J* = 8.8, 6.8, 2.4 Hz, 1H, H^2), 2.11–2.07 (m, 1H, H^4), 2.09 (ddd, *J* = 11.7, 8.8, 2.9 Hz, 1H, H^1), 1.76 (s, 3H, $COCH_3$), 1.67 (ddd, *J* = 13.1, 11.7, 5.9 Hz, 1H, H^6), 1.33 (ddd, *J* = 13.1, 2.9, 2.4 Hz, 1H, H^5), 1.05 (d, *J* = 7.1 Hz, 3H, CH_3), 0.98 (s, 9H, Si-$C(CH_3)_3$), 0.87 (d, *J* = 6.9 Hz, 3H, CH_3), 0.13 (s, 3H, Si-CH_3), 0.11 (s, 3H, Si-CH_3); ^{13}C NMR (150 MHz, C_6D_6): δ 209.2 (C), 153.5 (C), 108.4 (CH), 51.3 (CH), 33.2 (CH), 32.9 (CH_2), 31.4 (CH), 28.3 (CH_3), 25.9 (CH_3 × 3),

21.4 (CH_3), 18.8 (CH_3), 18.2 (C), –4.3 (CH_3), –4.5 (CH_3); ^{1}H NMR (600 MHz, CDCl3): δ 4.56 (d, J = 2.2 Hz, 1H, H^3), 2.55 (dqd, J = 9.1, 7.0, 2.2 Hz, 1H, H^2), 2.25 (ddd, J = 11.9, 9.1, 2.7 Hz, 1H, H^1), 2.17-2.6 (m, 1H, H^4), 2.14 (s, 3H, $COCH_3$), 1.79 (ddd, J = 12.9, 11.9, 5.9 Hz, 1H, H^6), 1.59 (ddd, J = 12.9, 2.7, 2.3 Hz, 1H, H^5), 1.10 (d, J = 7.1 Hz, 3H, CH_3), 0.89 (s, 9H, Si-$C(CH_3)_3$), 0.88 (d, J = 7.0 Hz, 3H, CH_3), 0.11 (s, 6H, Si-$(CH_3)_2$); ^{13}C NMR (150 MHz, CDCl3) δ 212.2 (C), 153.3 (C), 108.3 (CH), 51.3 (CH), 32.9 (CH_2), 32.8 (CH), 31.3 (CH), 28.8 (CH_3), 25.6 (CH_3 × 3), 21.1 (CH_3), 18.7 (CH_3), 18.0 (C), –4.4 (CH_3), –4.6 (CH_3); HRMS (ESI): *m/z* calcd for $C_{16}H_{31}O_2Si$ ($[M + H]^+$): 283.2093, found 283.2079. The relative stereochemistry was determined by NOESY experiment and coupling constant analysis.

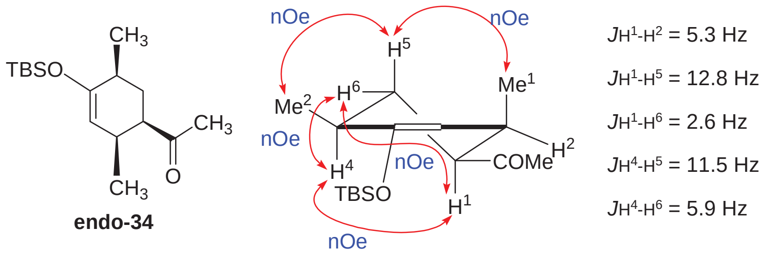

For **endo-34**: IR (thin film): ν 3407, 2958, 2931, 2859, 1716, 1658, 1463, 1370, 1257, 1173, 841, 778 cm^{-1}; ^{1}H NMR (400 MHz, C_6D_6): δ 4.78 (dd, J = 5.7, 1.8 Hz, 1H, H^3), 2.42 (m, 1H, H^2) , 2.29 (ddd, J = 12.8, 5.3, 2.6 Hz, 1H, H^1), 2.04 (m, 1H, H^4), 1.76 (dddd, J = 13.2, 5.9, 2.6, 1.1 Hz, 1H, H^6), 1.71 (s, 3H, $COCH_3$), 1.54 (ddd, J = 13.2, 12.8, 11.2 Hz, 1H, H^5), 1.08 (d, J = 6.9 Hz, 3H, CH_3), 0.98 (s, 9H, Si-$C(CH_3)_3$), 0.74 (d, J = 6.9 Hz, 3H, CH_3), 0.15 (s, 3H, Si-CH_3), 0.14 (s, 3H, Si-CH_3); ^{13}C NMR (100 MHz, C_6D_6) δ 207.9(C), 154.3 (C), 108.5 (CH), 51.1 (CH), 34.4 (CH), 30.8 (CH), 28.3 (CH_2), 27.8 (CH_3), 25.9 (CH_3 × 3), 18.8 (CH_3), 18.3 (C), 17.2 (CH_3), –4.3 (CH_3), –4.4 (CH_3); HRMS (APCI): *m/z* calcd for $C_{16}H_{31}O_2Si$ ($[M + H]^+$): 283.2093, found 283.2098. The relative stereochemistry was determined by NOESY experiment and coupling constant analysis.

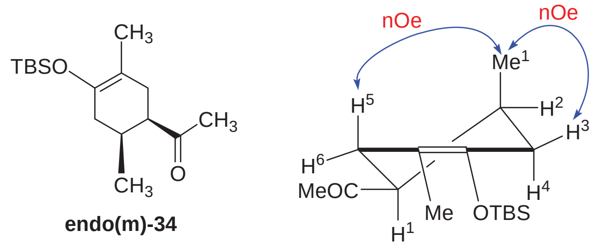

For **endo(m)-34**: IR (thin film): ν 3409, 2960, 2934, 2860, 1717, 1656, 1465, 1370, 1257, 1175, 841, 776 cm^{-1}; ^{1}H NMR (600 MHz, C_6D_6): δ 2.36–2.31 (m, 1H, H^5), 2.25–2.22 (m, 1H, H^4), 2.16 (ddd, J = 10.5, 5.4, 3.2 Hz, 1H, H^1), 2.08–2.05 (m, 1H, H^2), 1.85 (dd, J = 17.0, 5.2 Hz, 1H, H^6), 1.78 (dd, J = 16.4, 0.9 Hz, 1H, H^3), 1.70 (s, 3H, $COCH_3$), 1.64 (s, 3H, Me), 1.01

(s, 9H, Si-C(CH_3)$_3$), 0.79 (d, J = 6.9 Hz, 3H, Me^1), 0.11 (s, 3H, Si-CH_3), 0.10 (s, 3H, Si-CH_3); ^{13}C NMR (150 MHz, C_6D_6): δ 207.7 (C), 141.4 (C), 108.8 (C), 50.6 (CH), 38.1 (CH_2), 29.5 (CH), 27.9 (CH_3), 27.8 (CH_2), 26.0 (CH_3 × 3), 18.3 (C), 16.4 (CH_3), 14.6 (CH_3), –3.60 (CH_3), –3.64 (CH_3); HRMS (ESI): *m/z* calcd for $C_{16}H_{30}O_2NaSi$ ([M+Na]$^+$): 305.1913, found 305.1917. The relative stereochemistry was determined by NOESY experiment.

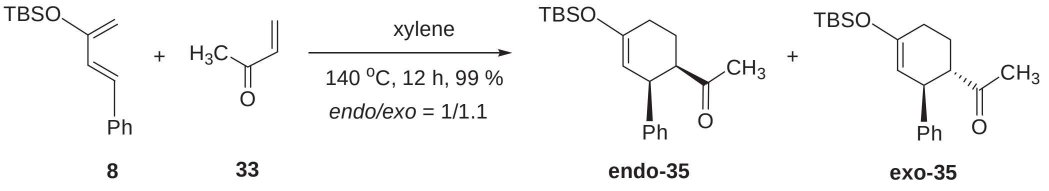

***Rac*-1-[2-phenyl-4-(*tert*-butyldimethylsilyloxy)-cyclohex-3-enyl]-ethan-1-ones (endo-35 and exo-35).** In a 25 mL sealed tube equipped with a magnetic stir bar, a solution of freshly distilled methylvinyl ketone (**33**, 0.577 mL, 6.923 mmol, 3.0 equiv.) and diene **8** (0.600 g, 2.308 mmol, 1.0 equiv.) in xylene (4.6 mL) was stirred for 12 h at 140 °C. Afterwards, the mixture was concentrated *in vacuo*. The residue was purified by column chromatography on silica gel (hexane/ethyl acetate = 20/1) to give the corresponding Diels-Alder cycloadducts **exo-35** and **endo-35** (combined: 0.754 g, 99%) as a colorless oil. The diastereoselectivity of the crude products was determined by ^{1}H NMR in $CDCl_3$. Integration of the signal due to the vinylic proton (H^3) of **exo-35** (δ_H 4.81 ppm) versus that of **endo-35** (δ_H 4.94 ppm) gave an *exo*/*endo* ratio of 1/1.1.

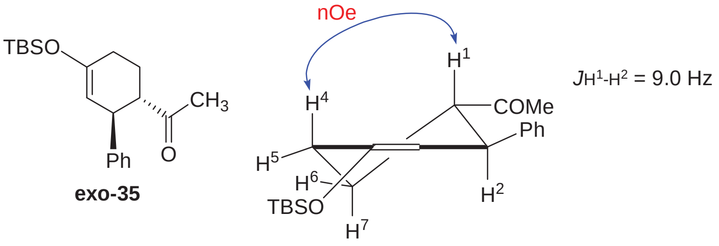

For **exo-35**: IR (thin film): ν 3027, 2930, 1713, 1666, 1472, 1258, 1175, 873 cm^{-1}; ^{1}H NMR (600 MHz, $CDCl_3$): δ 7.27–7.25 (m, 2H, Ar-H), 7.19–7.16 (m, 3H, Ar-H), 4.81 (s, 1H, H^3), 3.73 (dd, J = 9.0, 2.4 Hz, 1H, H^2), 2.60 (ddd, J = 11.3, 9.0, 3.1 Hz, 1H, H^1), 2.28–2.21 (m, 1H, H^4), 2.12–2.08 (m, 1H, H^5), 1.91–1.87 (m, 1H, H^7), 1.85 (s, 3H, COCH_3), 1.84–1.81 (m, 1H, H^6), 0.91 (s, 9H, Si-C(CH_3)$_3$), 0.14 (s, 3H, Si-CH_3), 0.12 (s, 3H, Si-CH_3); ^{13}C NMR (150 MHz, $CDCl_3$): δ 211.5 (C), 150.5 (C), 144.9 (C), 128.4 (CH × 2), 127.9 (CH × 2), 126.5 (CH), 107.5 (CH), 55.6 (CH), 43.5 (CH), 30.2 (CH_3), 29.0 (CH_2), 25.6 (CH_3 × 3), 25.2 (CH_2), 17.9 (C), –4.3 (CH_3), –4.4 (CH_3); HRMS (ESI): *m/z* calcd for $C_{20}H_{31}O2Si$ ([M + H]$^+$):

331.2093, found 331.2091. The relative stereochemistry was determined by NOESY experiment.

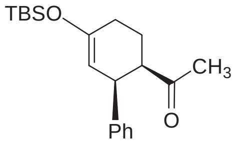

endo-35

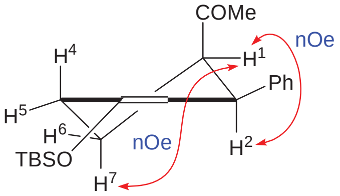

For **endo-35**: IR (thin film): ν 3028, 2930, 1712, 1667, 1472, 1252, 1178, 873 cm^{-1}; ^{1}H NMR (600 MHz, $CDCl_3$): δ 7.26–7.23 (m, 2H, Ar-H), 7.20–7.14 (m, 3H, Ar-H), 4.94 (d, *J* = 5.2 Hz, 1H, H^3), 3.91 (dd, *J* = 5.8, 5.2 Hz, 1H, H^2), 2.83 (ddd, *J* = 12.6, 5.8, 2.8 Hz, 1H, H^1), 2.21–2.15 (m, 2H, H^4 and H^5), 1.98–1.92 (m, 1H, H^6), 1.76 (s, 3H, $COCH_3$), 1.75–1.71 (m, 1H, H^7), 0.92 (s, 9H , Si-C(CH_3)$_3$), 0.15 (s, 6H, Si(CH_3)$_2$); ^{13}C NMR (150 MHz, $CDCl_3$): δ 211.1 (C), 151.8 (C), 140.9 (C), 129.1 (CH × 2), 128.1 (CH × 2), 126.9 (CH), 106.1 (CH), 52.3 (CH), 42.6 (CH), 29.09 (CH_3), 29.02 (CH_2), 25.6 (CH_3 × 3), 19.7 (CH_2), 18.0 (C), –4.2 (CH_3), –4.4 (CH_3); ^{1}H NMR (600 MHz, C_6D_6): δ 7.15–7.14 (m, 2H, Ar-H), 7.09 (dd, 7.6, 7.2 Hz, 2H, Ar-H), 7.03–7.00 (m, 1H, Ar-H), 4.95 (dd, *J* = 5.1, 1.3 Hz, 1H, H^3), 3.77 (dd, *J* = 5.8, 5.1 Hz, 1H, H^2), 2.50 (ddd, *J* = 12.4, 5.8, 2.9 Hz, 1H, H^1), 2.12–2.08 (m, 1H, H^4), 2.10–1.94 (m, 1H, H^5), 1.89–1.82 (m, 1H, H^6), 1.45 (s, 3H, $COCH_3$), 1.43–1.40 (m, 1H, H^7), 0.98 (s, 9H , Si-C(CH_3)$_3$), 0.122 (s, 3H, Si-CH_3), 0.126 (s, 3H, Si-CH_3); ^{13}C NMR (150 MHz, C_6D_6): δ 208.1 (C), 152.0 (C), 141.6 (C), 129.6 (CH × 2), 127.9 (CH × 2), 127.1 (CH), 106.3 (CH), 52.2 (CH), 42.8 (CH), 29.4 (CH_2), 28.6 (CH_2), 25.8 (CH_3 × 3), 20.2 (CH_2), 18.01 (C), –4.2 (CH_3), –4.3 (CH_3); HRMS (ESI): *m/z* calcd for $C_{20}H_{31}O2Si$ ([M + H]$^+$): 331.2093, found 331.2094. The relative stereochemistry was determined by NOESY experiments (in C_6D_6 and $CDCl_3$).

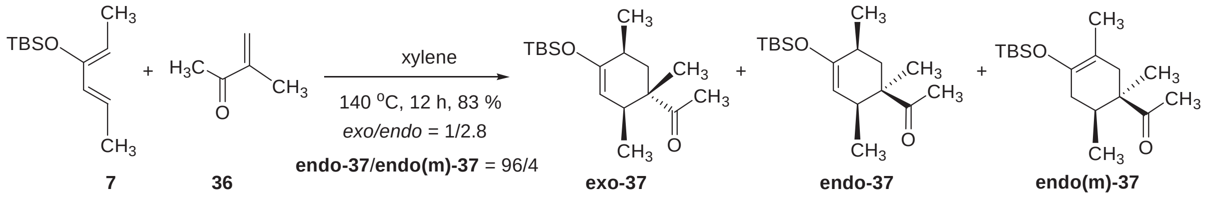

7 **36** **exo-37** **endo-37** **endo(m)-37**

***Rac*-1-[1,2,5-trimethyl-4-(*tert*-butyldimethylsilyloxy)-cyclohex-3-enyl]-ethan-1-ones (exo-37 and endo-37) and the corresponding *endo* olefin migrator *endo*(m)-37.** In a 25 mL sealed tube equipped with a magnetic stir bar, a solution of freshly distilled ketone **36** (0.425 mL, 4.25 mmol, 3.0 equiv.) and diene **7** (0.300 g, 1.20 mmol, 1.0 equiv.) in xylene (2.8 mL) was stirred for 12 h at 140 °C. Afterwards, the mixture was concentrated *in vacuo*.

The residue was purified by column chromatography on silica gel (hexanes/ethyl acetate = 20/1) to give the corresponding Diels-Alder cycloadducts **exo-37**, **endo-37** and **endo(m)-37** (combined: 0.296 g, 83%) as a colorless oil. The diastereoselectivity of the crude products was determined by ^{1}H NMR in $CDCl_3$. Integration of the signal due to the vinylic proton (H^2) of **exo-37** (δ_H 4.81 ppm) versus that of **endo-37** (δ_H 4.70 ppm) and H^4 of the corresponding *endo* olefin migrator **endo(m)-37** (δ_H 2.46 ppm) gave an apparent *exo*/*endo* ratio of 1/2.8 (the **endo-37**/**endo(m)-37** ratio is 96/4).

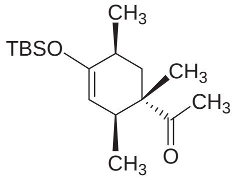

exo-37

For **exo-37**: IR (thin film): ν 2958, 2930, 2857, 1706, 1664, 1462, 1357, 1257, 1197, 866, 778 cm^{-1}; ^{1}H NMR (600 MHz, $CDCl_3$): δ 4.81 (d, *J* = 5.9 Hz, 1H, H^2), 2.64–2.60 (m, 1H, H^1) , 2.17-2.14 (m, 1H, H^3), 2.11 (s, 3H, $COCH_3$), 2.01 (ddd, *J* = 13.6, 6.7, 1.4 Hz, 1H, H^5), 1.26 (dd, *J* = 13.6, 10.1 Hz, 1H, H^4), 1.00 (d, *J* = 7.0 Hz, 3H, CH_3), 0.96 (s, 3H, CH_3), 0.93 (d, *J* = 6.9 Hz, 3H, CH_3), 0.86 (s, 9H, Si-$C(CH_3)_3$), 0.06 (s, 3H, Si-CH_3), 0.01 (s, 3H, Si-CH_3); ^{13}C NMR (150 MHz, $CDCl_3$): δ 213.2 (C), 153.9 (C), 109.4 (CH), 50.4 (CH), 35.9 (CH), 33.8 (CH_2), 32.0 (CH_3), 25.7 ($CH_3 \times 3$), 24.6 (CH_3), 22.9 (C), 18.9 (CH_3), 18.0 (CH_3), 17.8 (C), –4.3 (CH_3), –4.7 (CH_3); HRMS (APCI): *m/z* calcd for $C_{17}H_{33}O_2Si$ ($[M + H]^+$): 297.2250, found 297.2245.

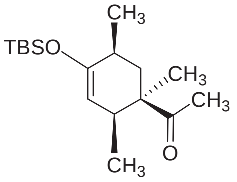

endo-37

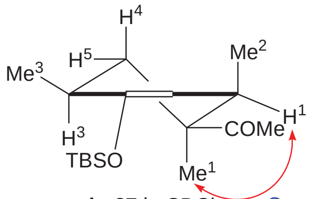

endo-37 in $CDCl_3$

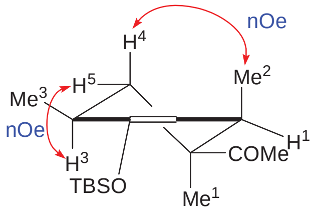

endo-37 in C_6D_6

$J_{H^3\text{-}H^4}$ = 11.1 Hz

$J_{H^3\text{-}H^5}$ = 6.3 Hz

For **endo-37**: IR (thin film): ν 2959, 2932, 2859, 1705, 1661, 1463, 1356, 1256, 1198, 865, 778 cm^{-1}; ^{1}H NMR (600 MHz, $CDCl_3$): δ 4.70 (dd, *J* = 5.8, 1.5 Hz, 1H, H^2), 2.26 (dq, *J* = 6.8, 5.8 Hz, 1H, H^1), 2.11–2.04 (m, 1H, H^3), 2.09 (s, 3H, $COCH_3$), 1.58 (m, 2H, H^4 and H^5), 1.16 (s, 3H, CH_3), 1.06 (d, *J* = 6.8 Hz, 3H, CH_3), 0.90 (s, 9H, Si-$C(CH_3)_3$), 0.79 (d, *J* = 6.8 Hz, CH_3), 0.12 (s, 3H, Si-CH_3), 0.10 (s, 3H, Si-CH_3); ^{13}C NMR (150 MHz, $CDCl_3$) δ 213.5 (C), 152.5 (C), 106.9 (CH), 49.6 (C), 37.2 (CH), 33.1 (CH_2), 31.2 (CH), 25.7 ($CH_3 \times 3$), 25.3 (CH_3), 21.6 (CH_3), 19.2 (CH_3), 18.4 (CH_3), 18.1 (C), –4.2(CH_3), –4.7 (CH_3); ^{1}H NMR (600 MHz, C_6D_6): δ 4.70 (dd, *J* = 5.9, 1.3 Hz, 1H, H^2), 2.04-2.00 (m, 1H, H^3), 2.02-1.96 (m, 1H,

H[1]), 1.77 (s, 3H, $COCH_3$), 1.74 (dd, J = 14.0, 11.1 Hz, 1H, H[4]), 1.63 (dd, J = 14.0, 6.3, 1.2 Hz, 1H, H[5]), 1.11 (d, J = 6.8 Hz, 3H, CH_3), 0.98 (s, 9H, Si-$C(CH_3)_3$), 0.96 (s, 3H, CH_3), 0.77 (d, J = 6.8 Hz, CH_3), 0.13 (s, 3H, Si-CH_3), 0.11 (s, 3H, Si-CH_3); ^{13}C NMR (150 MHz, C_6D_6): δ 210.7 (C), 152.8 (C), 107.0 (CH), 49.5 (C), 37.3 (CH), 33.7 (CH_2), 31.6 (CH), 25.9 (CH_3 × 3), 24.9 (CH_3), 21.6 (CH_3), 19.4 (CH_3), 18.8 (CH_3), 18.3 (C), –4.2 (CH_3), –4.5 (CH_3); HRMS (APCI): m/z calcd for $C_{17}H_{33}O_2Si$ ($[M + H]^+$): 297.2250, found 297.2245. The relative stereochemistry was determined by NOESY experiments (in C_6D_6 and $CDCl_3$) and coupling constant analysis.

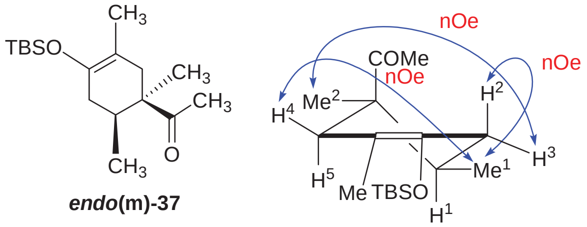

***endo*(m)-37**

For **endo(m)-37**: IR (thin film): ν 2957, 2929, 2857, 1707, 1667, 1464, 1358, 1256, 1198, 866, 776 cm^{-1}; ^{1}H NMR (600 MHz, $CDCl_3$): δ 2.46 (d, J = 17.2 Hz, 1H, H[4]), 2.37 (d, J = 17.0 Hz, 1H, H[3]), 2.08 (s, 3H, $COCH_3$), 2.05–2.02 (m, 1H, H[1]), 1.75 (d, J = 17.0 Hz, 1H, H[2]), 1.67 (d, J = 17.2 Hz, 1H, H[5]), 1.57 (s, 3H, Me[2]), 1.08 (s, 3H, Me), 0.92 (s, 9H, Si-$C(CH_3)_3$), 0.79 (d, J = 6.9 Hz, 3H, Me[1]), 0.09 (s, 3H, Si-CH_3), 0.08 (s, 3H, Si-CH_3); ^{13}C NMR (150 MHz, $CDCl_3$): δ 213.2 (C), 140.0 (C), 108.4 (C), 49.4 (C), 34.6 (CH), 34.5 (CH_2), 34.2 (CH_2), 25.8 (CH_3 × 3), 24.8 (CH_3), 22.7 (CH_3), 18.3 (C), 16.7 (CH_3), 16.4 (CH_3), –3.8 (CH_3× 2); HRMS (ESI): m/z calcd for $C_{17}H_{32}O_2NaSi$ ($[M+Na]^+$): 319.2069, found 319.2078. The relative stereochemistry was determined by NOESY experiment.

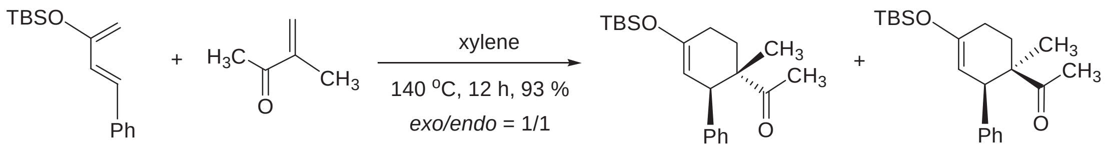

8 **36** **exo-38** **endo-38**

***Rac*-1-[1-methyl-2-phenyl-4-(*tert*-butyldimethylsilyloxy)-cyclohex-3-enyl]-ethan-1-ones (*endo*-38 and *exo*-38).** In a 25 mL sealed-tube equipped with a magnetic stir bar, a solution of freshly distilled ketone **36** (0.690 mL, 6.92 mmol, 3.0 equiv.) and diene **8** (0.600 g, 2.31 mmol, 1.0 equiv.) in xylene (4.6 mL) was stirred for 12 h at 140 °C. Afterwards, the reaction was concentrated *in vacuo*. The residue was purified by column chromatography on silica gel (hexanes/ethyl acetate = 20/1) to give the corresponding Diels-Alder cycloadducts **exo-38** and **endo-38** (combined: 0.738 g, 93%) as a colorless oil. The diastereoselectivity of

the crude products was determined by ^{1}H NMR in $CDCl_3$. Integration of the signal due to the vinylic proton (H^2) of **exo-38** (δ_H 4.89 ppm) versus that of **endo-38** (δ_H 4.84 ppm) gave an *exo/endo* ratio of 1/1.

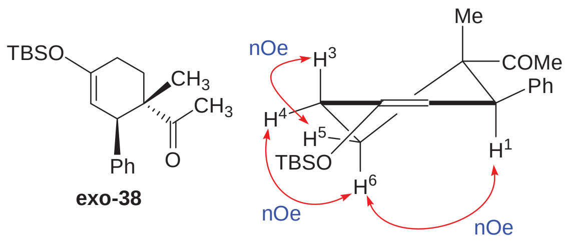

For **exo-38**: IR (thin film): ν 3027, 2930, 1702, 1666, 1461, 1258, 1175, 839 cm^{-1}; ^{1}H NMR (600 MHz, C_6D_6): δ 7.18–7.13 (m, 4H, Ar-H), 7.08–7.05 (m, 1H, Ar-H), 5.00 (d, J = 4.5 Hz, 1H, H^2), 4.06 (d, J = 4.5 Hz, 1H, H^1), 2.20-2.15 (m, 1H, H^4), 2.07–2.02 (m, 1H, H^3), 1.85–1.82 (m, 1H, H^6), 1.80 (s, 3H, $COCH_3$), 1.42–1.38 (m, 1H, H^5), 0.97 (s, 9H, Si-$C(CH_3)_3$), 0.64 (s, 3H, CH_3), 0.12 (s, 3H, Si-CH_3), 0.10 (s, 3H, Si-CH_3); ^{13}C NMR (150 MHz, C_6D_6): δ 210.6 (C), 151.5 (C), 142.4 (C), 130.2 (CH × 2), 128.2 (CH × 2), 126.8 (CH), 107.1 (CH), 50.4 (C), 46.0 (CH), 29.1 (CH_2), 27.5 (CH_2), 25.8 (CH_3 × 3), 24.9 (CH_3), 20.9 (CH_3), 18.1 (C), –4.2 (CH_3), –4.4 (CH_3); ^{1}H NMR (600 MHz, $CDCl_3$): δ 7.27–7.25 (m, 2H, Ar-H), 7.21–7.19 (m, 1H, Ar-H), 7.15–7.14 (m, 2H, Ar-H), 4.89 (ddd, J = 4.5, 1.5, 1.0 Hz, 1H, H^2), 4.01 (m, 1H, H^1), 2.14 (s, 3H, $COCH_3$), 2.13–2.12 (m, 2H, H^5 and H^6), 1.96 (ddd, J = 13.2, 6.6, 6.0 Hz, 1H, H^4), 1.61 (ddd, J = 13.2, 7.3, 6.6 Hz, 1H, H^3), 0.91 (s, 9H, Si-$C(CH_3)_3$), 0.80 (s, 3H, CH_3), 0.13 (s, 3H, Si-CH_3), 0.11 (s, 3H, Si-CH_3); ^{13}C NMR (150 MHz, $CDCl_3$): δ 213.0 (C), 151.0 (C), 141.9 (C), 129.7 (CH × 2), 127.8 (CH × 2), 126.5 (CH), 106.8 (CH), 50.4 (C), 45.8 (CH), 29.2 (CH_2), 27.0 (CH_2), 25.67 (CH_3 × 3), 26.60 (CH_3), 20.5 (CH_3), 17.9 (C), –4.3 (CH_3), –4.4 (CH_3); HRMS (ESI): m/z calcd for $C_{21}H_{32}O_2NaSi$ ($[M + Na]^+$): 367.2069, found 367.2068. The relative stereochemistry was determined by NOESY experiment.

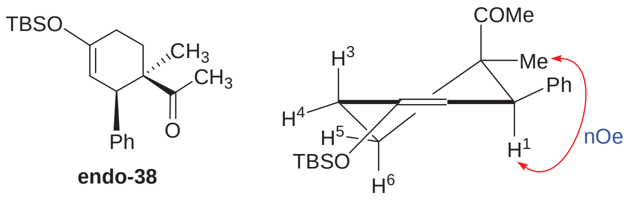

For **endo-38**: IR (thin film): ν 3028, 2932, 1704, 1670, 1462, 1251, 1173, 838 cm^{-1}; ^{1}H NMR (600 MHz, C_6D_6): δ 7.18–7.15 (m, 2H, Ar-H), 7.09–7.07 (m, 2H, Ar-H), 7.01-6.99 (m, 1H, Ar-H), 4.87 (dd, J = 5.4, 1.6 Hz, 1H, H^2), 3.33 (d, J = 5.4 Hz, 1H, H^1), 2.17–2.11 (m, 2H, H^3 and H^5), 2.06–2.00 (m, 1H, H^6), 1.37 (s, 3H, $COCH_3$), 1.26–1.22 (m, 1H, H^4), 1.20 (s, 3H, CH_3), 0.98 (s, 9H, Si-$C(CH_3)_3$), 0.11 (s, 3H, Si-CH_3), 0.10 (s, 3H, Si-CH_3); ^{13}C NMR (150

MHz, C_6D_6): δ 210.9 (C), 150.4 (C), 142.1 (C), 129.9 (CH × 2), 128.2 (CH × 2), 127.0 (CH), 105.7 (CH), 50.2 (C), 49.9 (CH), 27.1 (CH_3), 27.0 (CH_2), 25.8 (CH_3 × 3 and CH_2), 22.1 (CH_3), 18.1 (C), –4.1 (CH_3), –4.3 (CH_3); ^{1}H NMR (600 MHz, $CDCl_3$): δ 7.24–7.21 (m, 2H, Ar-H), 7.18–7.14 (m, 3H, Ar-H), 4.84 (dd, *J* = 5.4, 1.5 Hz, 1H, H^2), 3.41 (d, *J* = 5.4 Hz, 1H, H^1), 2.27–2.21 (m, 2H, H^3 and H^5), 2.18–2.13 (m, 1H, H^6), 1.61 (s, 3H, $COCH_3$), 1.51 (ddd, *J* = 6.1, 5.6, 0.9 Hz, 1H, H^4), 1.31 (s, 3H, CH_3), 0.92 (s, 9H, Si-C(CH_3)$_3$), 0.14 (s, 6H, Si(CH_3)$_2$); ^{13}C NMR (150 MHz, $CDCl_3$): δ 213.4 (C), 150.1 (C), 141.6 (C), 129.4 (CH × 2), 128.0 (CH × 2), 126.8 (CH), 105.4 (CH), 50.2 (C), 49.7 (CH), 27.2 (CH_3), 26.7 (CH_2), 25.6 (CH_3 × 3), 25.4 (CH_2), 22.0 (CH_3), 17.9 (C), –4.2 (CH_3), –4.4 (CH_3); HRMS (ESI): *m/z* calcd for $C_{21}H_{32}O_2NaSi$ ([M + Na]$^+$): 367.2069, found 367.2075. The relative stereochemistry was determined by NOESY experiment.

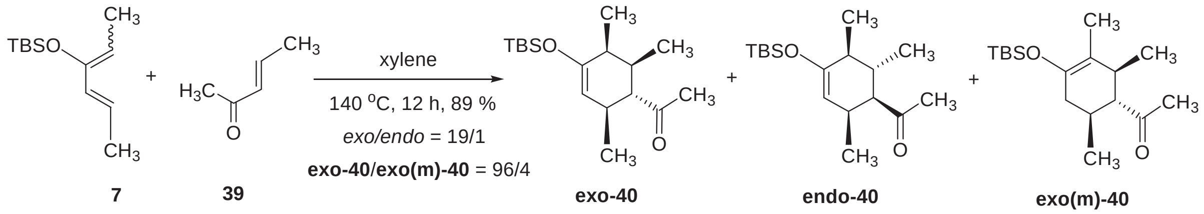

***Rac*-1-[2,5,6-trimethyl-4-(*tert*-butyldimethylsilyloxy)-cyclohex-3-enyl]-ethan-1-ones (exo-40 and endo-40) and the corresponding *exo* olefin migrator *exo*(m)-40.** In a 25 mL sealed tube equipped with a magnetic stir bar, a solution of freshly distilled ketone **39** (0.424 mL, 4.25 mmol, 3.0 equiv.), diene **7** (0.300 g, 1.20 mmol, 1.0 equiv.) in xylene (2.8 mL) was stirred for 12 h at 140 °C. Afterwards, the mixture was concentrated *in vacuo*. The residue was purified by column chromatography on silica gel (hexanes/ethyl acetate = 20/1) to give the corresponding Diels-Alder cycloadducts **exo-40**, **endo-40** and **exo(m)-40** (combined: 0.317 g, 89%) as a colorless oil. The diastereoselectivity of the crude products was determined by ^{1}H NMR in $CDCl_3$. Integration of the signal due to the vinylic proton (H^3) of **exo-40** (δ_H 4.55 ppm) versus that of the **endo-40** (δ_H 4.87 ppm) and H^1 of the corresponding *exo* olefin migrator **exo(m)-40** (δ_H 1.85 ppm) gave an apparent *exo*/*endo* ratio of 19/1 (the **exo-40**/**exo(m)-40** ratio is 96/4).

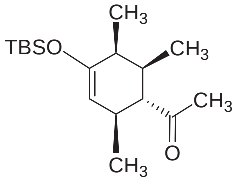

exo-40

For **exo-40**: IR (thin film): ν 2958, 2930, 2859, 1710, 1667, 1463, 1358, 1255, 1056, 837, 779 cm^{-1}; ^{1}H NMR (600 MHz, $CDCl_3$): δ 4.55 (d, J = 2.1 Hz, 1H, H^3), 2.48–2.47 (m, 1H, H^2) , 2.12 (s, 3H, $COCH_3$), 2.07–2.04 (m, 2H, H^1 and H^4), 2.00–1.97 (m, 1H, H^5), 0.93 (d, J = 7.0 Hz, 3H, Me^3), 0.90 (s, 9H, Si-$C(CH_3)_3$), 0.86 (d, J = 6.8 Hz, 3H, Me^1), 0.78 (d, J = 6.3 Hz, 3H, Me^2) , 0.11 (s, 6H, Si-$(CH_3)_2$); ^{13}C NMR (150 MHz, $CDCl_3$): δ 213.9 (C), 154.1 (C), 107.6 (CH), 57.1 (CH), 38.9 (CH), 34.9 (CH), 33.4 (CH), 30.5 (CH_3), 25.6 (CH_3 × 3), 21.0 (CH_3), 18.0 (C), 16.1 (CH_3), 12.9 (CH_3), –4.4 (CH_3), –4.6 (CH_3); HRMS (APCI): *m/z* calcd for $C_{17}H_{33}O_2Si$ ($[M+H]^+$): 297.2250, found 297.2249.

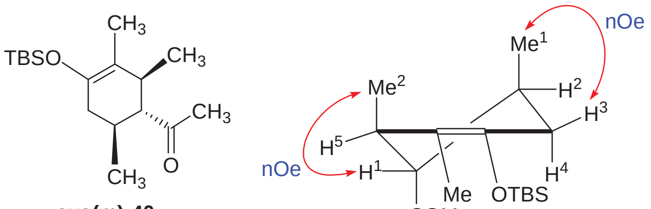

exo(m)-40

For **exo(m)-40**: IR ($CHCl_3$): ν 2957, 2932, 2857, 1706, 1665, 1462, 1358, 1256, 1196, 867, 778 cm^{-1}; ^{1}H NMR (600 MHz, C_6D_6): δ 2.43–2.37 (m, 1H, H^5), 2.43–2.37 (m, 1H, H^3), 1.85 (dd, J = 10.6, 9.6 Hz, 1H, H^1), 1.81–1.79 (m, 1H, H^2), 1.78 (s, 3H, $COCH_3$), 1.76–1.69 (m, 1H, H^4), 1.60 (s, 3H, Me), 1.01 (s, 9H, Si-$C(CH_3)_3$), 0.82 (d, J = 6.9 Hz, 3H, Me^2), 0.68 (d, J = 6.4 Hz, 3H, Me^1), 0.09 (s, 3H, Si-CH_3), 0.08 (s, 3H, Si-CH_3); ^{13}C NMR (150 MHz, C_6D_6): δ 210.9 (C), 142.6 (C), 114.2 (C), 63.8 (CH), 39.3 (CH_2), 37.3 (CH), 31.9 (CH), 29.9 (CH_3), 26.0 (CH_3 × 3), 19.4 (CH_3), 18.9 (CH_3), 18.3 (C), 13.5 (CH_3), –3.6 (CH_3), –3.9 (CH_3); HRMS (ESI): *m/z* calcd for $C_{17}H_{32}O_2NaSi$ ($[M+ Na]^+$): 319.2069, found 319.2076. The relative stereochemistry was determined by NOESY experiment.

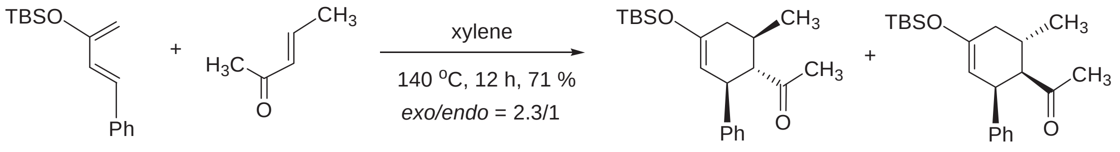

***Rac*-1-[6-methyl-2-phenyl-4-(*tert*-butyldimethylsilyloxy)-cyclohex-3-enyl]-ethan-1-ones (*exo*-41 and *endo*-41).** In a 25 mL sealed tube equipped with a magnetic stir bar, a solution of freshly distilled ketone **39** (0.690 mL, 6.92 mmol, 3.0 equiv.) and diene **8** (0.600 g, 2.308 mmol, 1.0 equiv.) in xylene (4.6 mL) was stirred for 12 h at 140 °C. Afterwards, the mixture was concentrated *in vacuo*. The residue was purified by column chromatography on silica gel (hexanes/ethyl acetate = 20/1) to give the corresponding Diels-Alder cycloadducts **exo-41** and **endo-41** (combined: 0.565 g, 71%) as a colorless oil. The diastereoselectivity of

the crude products was determined by ^{1}H NMR in $CDCl_3$. Integration of the signal due to the vinylic proton (H^3) of **exo-41** (δ_H 4.92 ppm) versus that of **endo-41** (δ_H 4.79 ppm) gave an *exo/endo* ratio of 2.3:1.

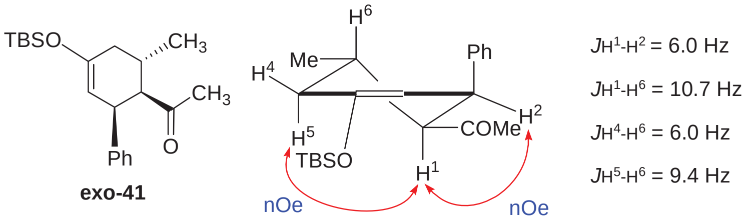

For **exo-41**: IR (thin film): ν 2961, 1711, 1666, 14446, 1255, 1157, 838 cm^{-1}; ^{1}H NMR (600 MHz, $CDCl_3$): δ 7.26–7.20 (m, 2H, Ar-H), 7.19–7.17 (m, 1H, Ar-H), 7.11–7.10 (m, 2H, Ar-H), 4.92 (dd, J = 4.8, 0.8 Hz, 1H, H^3), 3.08 (dd, J = 6.0, 4.8 Hz, 1H, H^2), 2.67 (dd, J = 10.7, 6.0 Hz, 1H, H^1), 2.30 (dd, J = 17.1, 6.0 Hz, 1H, H^4), 2.27–2.22 (m, 1H, H^6), 1.82 (ddd, J = 17.1, 9.4, 0.8 Hz, 1H, H^5), 1.73 (s, 3H, $COCH_3$), 0.92 (s, 9H, Si-C(CH_3)$_3$), 0.90 (d, J = 6.0 Hz, 3H, CH_3), 0.15 (s, 3H, Si-CH_3), 0.14 (s, 3H, Si-CH_3); ^{13}C NMR (150 MHz, $CDCl_3$): δ 210.7 (C), 150.7 (C), 141.4 (C), 129.1 (CH × 2), 128.1 (CH × 2), 126.9 (CH), 105.6 (CH), 59.3 (CH), 43.3 (CH), 37.4 (CH_2), 30.0 (CH_3), 25.67 (CH_3 × 3), 25.62 (CH), 20.1 (CH_3), 18.0 (C), –4.2 (CH_3), –4.3 (CH_3); HRMS (ESI): *m/z* calcd for $C_{21}H_{32}O_2NaSi$ ([M+ Na]$^+$): 367.2069, found 367.2061. The relative stereochemistry was determined by NOESY experiment and coupling constant analysis.

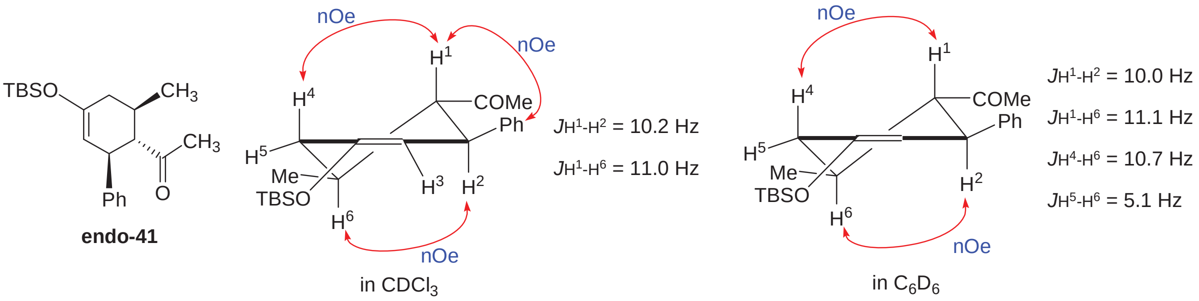

For **endo-41**: IR (thin film): ν 3028, 2929, 1713, 1668, 1463, 1253, 1178, 839 cm^{-1}; ^{1}H NMR (600 MHz, $CDCl_3$): δ 7.27–7.26 (m, 2H, Ar-H), 7.19–7.17 (m, 1H, Ar-H), 7.12–7.11 (m, 1H, Ar-H), 4.79 (dd, J = 2.0, 1.8 Hz, 1H, H^3), 3.60 (dddd, J = 10.2, 3.8, 1.8, 1.6 Hz, 1H, H^2), 2.37 (dd, J = 11.0, 10.2 Hz, 1H, H^1), 2.18–2.12 (m, 2H, H^6 and H^5), 1.96-1.91 (m, 1H, H^4), 1.68 (s, 3H, $COCH_3$), 0.91 (s, 9H, Si-C(CH_3)$_3$), 0.90 (d, J = 6.3 Hz, 3H, CH_3), 0.14 (s, 3H, Si-CH_3), 0.12 (s, 3H, Si-CH_3); ^{13}C NMR (150 MHz, $CDCl_3$): δ 213.7 (C), 150.0 (C), 144.4 (C), 128.6 (CH × 2), 127.7 (CH × 2), 126.7 (CH), 107.6 (CH), 62.8 (CH), 46.3 (CH), 38.1 (CH_2), 33.4 (CH_3), 32.4 (CH), 25.6 (CH_3 × 3), 19.4 (CH_3), 18.0 (C), –4.3 (CH_3), –4.4 (CH_3); ^{1}H NMR (600 MHz, C_6D_6) δ 7.15–7.14 (m, 2H, Ar-H), 7.12–7.09 (m, 2H, Ar-H), 7.03–7.01 (m, 1H,

Ar-H), 4.89 (dd, J = 2.1, 1.8 Hz, 1H, H^3), 3.80 (dddd, J = 10.0, 3.8, 1.8, 1.6 Hz, 1H, H^2), 2.23 (dd, J = 11.1, 10.0 Hz, 1H, H^1), 2.17 (ddqd, J = 11.1, 10.7, 6.3, 5.1 Hz, 1H, H^6), 2.06 (ddd, J = 17.0, 5.1, 1.5 Hz, 1H, H^5), 1.84 (dddd, J = 17.0, 10.7, 3.7, 2.1 Hz, 1H, H^4), 1.45 (s, 3H, $COCH_3$), 0.98 (s, 9H, Si-$C(CH_3)_3$), 0.70 (d, J = 6.3 Hz, 3H, CH_3), 0.12 (s, 3H, Si-CH_3), 0.08 (s, 3H, Si-CH_3); ^{13}C NMR (150 MHz, C_6D_6): δ 211.2 (C), 150.4 (C), 145.1 (C), 128.4 (CH × 2), 128.1 (CH × 2), 126.9 (CH), 107.6 (CH), 62.6 (CH), 46.7 (CH), 38.4 (CH_2), 33.4 (CH_3), 32.7 (CH), 25.8 (CH_3 × 3), 19.4 (CH_3), 18.1 (C), –4.30 (CH_3), –4.38 (CH_3); HRMS (ESI): *m/z* calcd for $C_{21}H_{32}O_2NaSi$ ([M+ Na]$^+$): 367.2069, found 367.2067. The relative stereochemistry was determined by NOESY experiments (in C_6D_6 and $CDCl_3$) and coupling constant analysis.

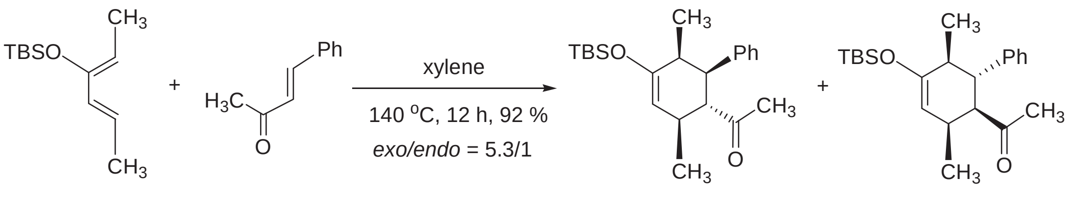

7 **42** **exo-43** **endo-43**

***Rac*-1-[2,5-dimethyl-6-phenyl-4-(*tert*-butyldimethylsilyloxy)-cyclohex-3-enyl]-ethan-1-ones (exo-43 and endo-43).** In a 25 mL sealed tube equipped with a magnetic stir bar, a solution of ketone **42** (0.413 g, 2.83 mmol, 2.0 equiv.) and diene **7** (0.300 g, 1.20 mmol, 1.0 equiv.) in xylene (2.8 mL) was stirred for 12 h at 140 °C. Afterwards, the mixture was concentrated *in vacuo*. The residue was purified by column chromatography on silica gel (hexanes/ethyl acetate = 20/1) to give the corresponding Diels-Alder cycloadducts **exo-43** and **endo-43** (combined: 0.396 g, 92%) as a colorless oil. The diastereoselectivity of the crude products was determined by ^{1}H NMR in $CDCl_3$. Integration of the signal due to the vinylic proton (H^3) of the major adduct **exo-43** (δ_H 4.64 ppm) versus that of the minor adduct **endo-43** (δ_H 4.96 ppm) gave and *exo*/*endo* ratio of 5.3:1.

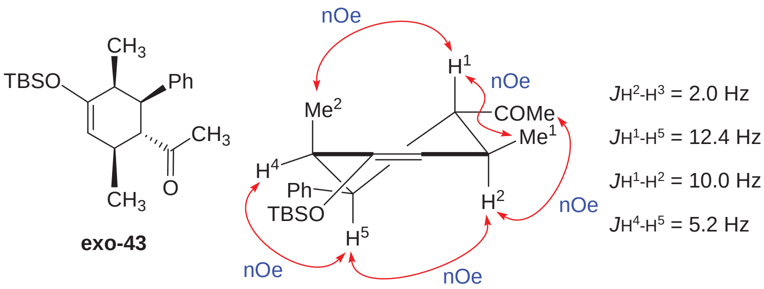

For **exo-43**: m.p. 75–76 °C; IR (thin film): ν 3095, 2958, 2930, 2858, 1705, 1668, 1472, 1354, 1257, 1177, 839, 778 cm^{-1}; ^{1}H NMR (400 MHz, $CDCl_3$): δ 7.26–7.23 (m, 2H, Ar-H), 7.18–

7.14 (m, 1H, Ar-H), 7.09 (d, J = 7.8 Hz, 2H, Ar-H), 4.64 (d, J = 2.0 Hz, 1H, H^3), 3.35 (dd, J = 12.4, 5.2 Hz, 1H, H^5), 2.70 (dd, J = 12.4, 10.0 Hz, 1H, H^1), 2.54-2.50 (m, 1H, H^2), 2.21-2.18 (m, 1H, H^4), 1.84 (s, 3H, $COCH_3$), 0.99 (d, J = 6.8 Hz, 3H, Me^1), 0.90 (s, 9H, Si-$C(CH_3)_3$), 0.78 (d, J = 7.1 Hz, 3H, Me^2), 0.15 (s, 3H, Si-CH_3) , 0.14 (s, 3H, Si-CH_3); ^{13}C NMR (75 MHz, $CDCl_3$): δ 212.0 (C), 154.1 (C), 140.8 (C), 128.6 (CH × 2), 128.2 (CH × 2), 126.5 (CH), 107.1 (CH), 55.2 (CH), 47.2 (CH), 40.0 (CH), 33.5 (CH), 27.8 (CH_3), 25.6 (CH_3 × 3), 21.0 (CH_3), 18.0 (C), 14.2 (CH_3), –4.2 (CH_3), –4.7 (CH_3); HRMS (APCI): *m/z* calcd for $C_{22}H_{35}O_2Si$ ($[M+H]^+$): 359.2406, found 359.2412. The relative stereochemistry was determined by NOESY experiment.

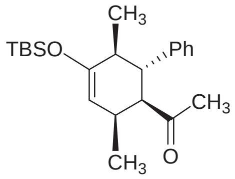

endo-43

For **endo-43**: IR (thin film): ν 3094, 2957, 2929, 2857, 1704, 1672, 1472, 1355, 1258, 1177, 839, 776 cm^{-1}; ^1H NMR (600 MHz, $CDCl_3$) δ 7.25–7.22 (m, 2H, Ar-H), 7.16–7.13 (m, 1H, Ar-H) , 7.10 (d, J = 7.8 Hz, 2H, Ar-H), 4.96 (dd, J = 5.9, 1.3 Hz, 1H, H^3), 3.31 (dd, J = 12.3, 5.2 Hz, 1H, H^1), 2.80 (dd, J = 12.3, 9.9 Hz, 1H, H^5), 2.68-2.66 (m, 1H, H^2), 2.16-2.12 (m, 1H, H^4), 1.89 (s, 3H, $COCH_3$), 0.91 (d, J = 6.8 Hz, 3H, Me^1), 0.91 (s, 9H, Si-$C(CH_3)_3$), 0.89 (d, J = 7.2 Hz, 3H, Me^2), 0.16 (s, 3H, Si-CH_3) , 0.15 (s, 3H, Si-CH_3); ^{13}C NMR (150 MHz, $CDCl_3$): δ 209.6 (C), 152.9 (C), 144.2 (C), 128.3 (CH), 128.2 (CH), 126.2 (CH), 108.1 (CH), 56.3 (CH), 45.0 (CH), 41.8 (CH), 30.7 (CH), 30.6 (CH_3), 25.7 (CH_3 × 3), 18.1 (C), 17.9 (CH_3), 16.3 (CH_3), –4.4 (CH_3), –4.6 (CH_3); HRMS (APCI): *m/z* calcd for $C_{22}H_{35}O_2Si$ ($[M+H]^+$): 359.2406, found 359.2412.

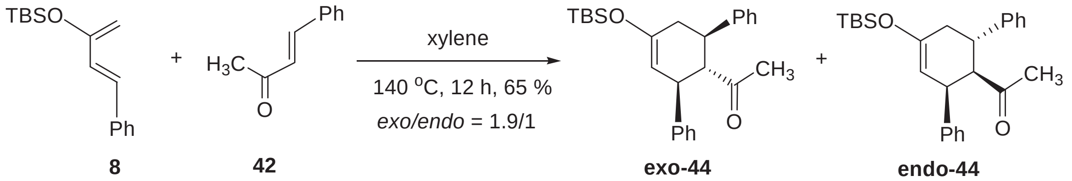

8 **42** **exo-44** **endo-44**

***Rac*-1-[2,6-diphenyl-4-(*tert*-butyldimethylsilyloxy)–cyclohex-3-enyl]-ethan-1-ones (exo-44 and endo-44).** In a 25 mL sealed tube equipped with a magnetic stir bar, a solution of freshly distilled ketone **42** (0.674 g, 4.62 mmol, 2.0 equiv.) and diene **8** (0.600 g, 2.31 mmol, 1.0 equiv.) in xylene (4.6 mL) was stirred for 12 h at 140 °C. Afterwards, the reaction was concentrated *in vacuo*. The residue was purified by column chromatography on silica

gel (eluent, Hexane/EA = 20/1) to give the corresponding Diels-Alder cycloadducts **exo-44** and **endo-44** (0.609 g, 65%) as a white solid. The diastereoselectivity of the crude products was determined by ^{1}H NMR in $CDCl_3$. Integration of the signal due to the vinylic proton (H^3) of the major adduct **exo-44** (δ_H 4.90 ppm) versus that of the minor adduct **endo-44** (δ_H 5.00 ppm) gave an *exo*/*endo* ratio of 1.9:1.

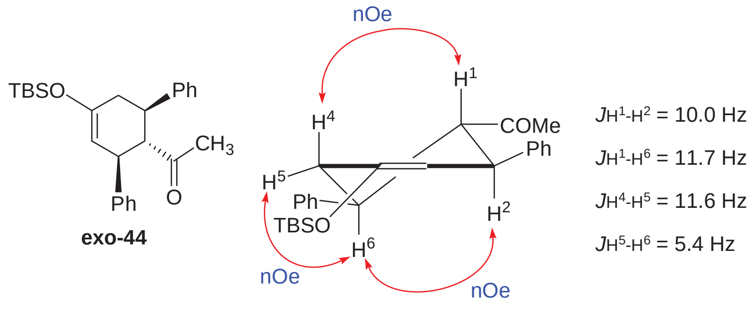

For **exo-44**: m.p. 73–74 °C; IR (thin film): ν 3028, 2929, 1713, 1668, 1454, 1252, 1176, 830 cm^{-1}; ^{1}H NMR (600 MHz, $CDCl_3$): δ 7.29–7.24 (m, 4H, Ar-H), 7.21–7.17 (m, 6H, Ar-H), 4.90 (s, 1H, H^3), 3.79 (d, J = 10.0 Hz, 1H, H^2), 3.24 (ddd, J = 11.7, 11.6, 5.4 Hz, 1H, H^6), 2.96 (dd, J = 11.7, 10.0 Hz, 1H, H^1), 2.52 (dd, J = 17.5, 11.6 Hz, 1H, H^4), 2.34 (dd, J = 17.5, 5.4 Hz, 1H, H^5), 1.24 (s, 3H, $COCH_3$), 0.92 (s, 9H, Si-$C(CH_3)_3$), 0.18 (s, 3H, Si-CH_3), 0.16 (s, 3H, Si-CH_3); ^{13}C NMR (150 MHz, $CDCl_3$): δ 212.5 (C), 150.0 (C), 144.2 (C), 142.4 (C), 128.68 (CH × 2), 128.64 (CH × 2), 127.7 (CH × 2), 127.6 (CH × 2), 126.9 (CH), 126.7 (CH), 107.5 (CH), 61.7 (CH), 46.3 (CH), 44.4 (CH), 37.8 (CH_2), 33.3 (CH_3), 25.6 (CH_3 × 3), 17.9 (C), –4.36 (CH_3), –4.45 (CH_3); HRMS (ESI): *m/z* calcd for $C_{26}H_{35}O2Si$ ($[M+ H]^+$): 407.2406, found 407.2409. The relative stereochemistry was determined by NOESY experiment and coupling constant analysis.

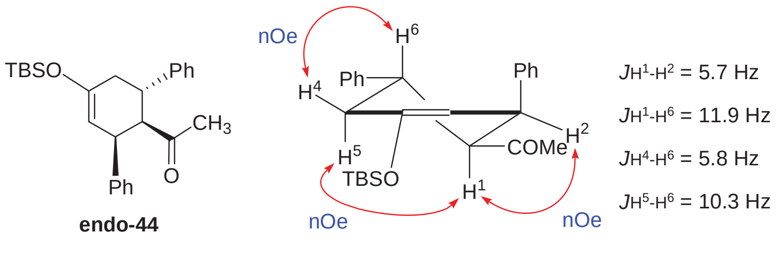

For **endo-44**: m.p. 70–71 °C; IR (thin film): ν 3028, 2929, 1698, 1672, 1453, 1253, 1199, 837 cm^{-1}; ^{1}H NMR (600 MHz, $CDCl_3$): δ 7.31–7.28 (m, 2H, Ar-H), 7.25–7.20 (m, 5H, Ar-H), 7.16–7.13 (m, 3H, Ar-H), 5.00 (dd, J = 5.1, 1.4 Hz, 1H, H^3), 3.95 (m, 1H, H^2), 3.31 (m, 2H, H^1 and H^6), 2.48 (dd, J = 18.9, 3.8 Hz, 1H, H^4), 2.23–2.15 (m, 1H, H^5), 1.16 (s, 3H, $COCH_3$), 0.91 (s, 9H, Si-$C(CH_3)_3$), 0.17 (s, 3H, Si-CH_3), 0.16 (s, 3H, Si-CH_3); ^{13}C NMR (150 MHz, $CDCl_3$): δ 211.3 (C), 150.8 (C), 144.2 (C), 140.8 (C), 129.4 (CH × 2), 128.7 (CH × 2), 128.3 (CH × 2), 127.2 (CH × 2), 127.1 (CH), 126.6 (CH), 105.5 (CH), 57.6 (CH), 43.8 (CH), 39.5

(CH_2), 37.5 (CH), 29.3 (CH_3), 25.6 (CH_3 × 3), 17.9 (C), –4.2 (CH_3), –4.4 (CH_3); ^{1}H NMR (600 MHz, C_6D_6): δ 7.23–7.21 (m, 2H, Ar-H), 7.04–7.01 (m, 3H, Ar-H), 6.98–6.93 (m, 3H, Ar-H), 5.01 (dd, *J* = 5.0, 1.5 Hz, 1H, H^3), 3.91 (dd, *J* = 5.7, 5.0 Hz, 1H, H^2), 3.32 (ddd, *J* = 11.9, 10.3, 5.8 Hz, 1H, H^6), 3.26 (dd, *J* = 11.9, 5.7 Hz, 1H, H^1), 2.52 (dd, *J* = 17.6, 5.8 Hz, 1H, H^4), 2.21 (ddd, *J* = 17.6, 10.3, 1.5 Hz, 1H, H^5), 1.15 (s, 3H, $COCH_3$), 0.97 (s, 9H, Si-$C(CH_3)_3$), 0.14 (s, 3H, Si-CH_3), 0.13 (s, 3H, Si-CH_3); ^{13}C NMR (150 MHz, C_6D_6): δ 208.3 (C), 151.2 (C), 144.8 (C), 141.6 (C), 129.7 (CH × 2), 128.9 (CH × 2), 128.5 (CH × 2), 127.6 (CH × 2), 127.2 (CH), 126.7 (CH), 105.6 (CH), 57.6 (CH), 43.9 (CH), 39.7 (CH_2), 38.0 (CH), 29.4 (CH_3), 25.7 (CH_3 × 3), 18.1 (C), –4.2 (CH_3), –4.4 (CH_3); HRMS (ESI): *m/z* calcd for $C_{26}H_{35}O_2Si$ ([M+ H]$^+$): 407.2406, found 407.2400. The relative stereochemistry was determined by NOESY experiment and coupling constant analysis.

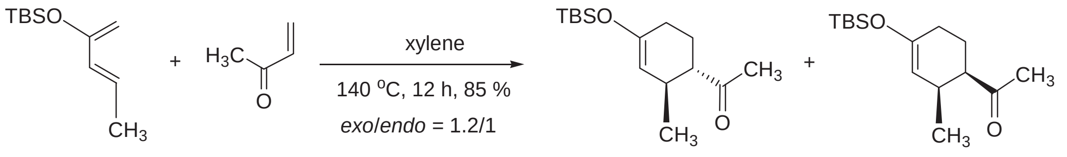

S1 **33** **exo-S2** **endo-S2**

***Rac*-1-[4-(*tert*-butyl-dimethyl-silanyloxy)-2-methyl–cyclohex-3-enyl]-ethanones (exo-S2 and endo-S2).** In a 25 mL sealed tube equipped with a magnetic stir bar, a solution of freshly distilled methylvinyl ketone (**33**, 0.590 mL, 7.08 mmol, 5.0 equiv.) and diene **S1** (0.28 g, 1.42 mmol, 1.0 equiv.) in xylene (2.8 mL) was stirred for 12 h at 140 °C. Afterwards, the reaction was concentrated *in vacuo*. The residue was purified by column chromatography on silica gel (hexanes/ethyl acetate = 20/1) to give the corresponding Diels-Alder cycloadducts **exo-S2** and **endo-S2** (0.322 g, 85%) as a colorless oil. The diastereoselectivity of the crude products was determined by ^{1}H NMR in $CDCl_3$. Integration of the signal due to the vinylic proton (H^3) of the major isomer **exo-S2** (δ_H 4.66 ppm) versus that of the minor isomer **endo-S2** (δ_H 4.87 ppm) gave an *exo*/*endo* ratio of 1.2/1.

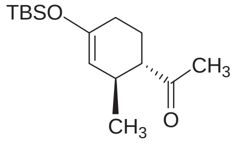

exo-S2

For **exo-S2**: IR (thin film): ν 3405, 2958, 2931, 2861, 1716, 1658, 1465, 1371, 1256, 1173, 843, 778 cm^{-1}; ^{1}H NMR (600 MHz, $CDCl_3$): δ 4.66 (s, 1H, H^3), 2.57–2.56 (m, 1H, H^2), 2.15 (s, 3H, $COCH_3$), 2.13–2.08 (m, 2H, H^6 and H^7), 2.02–2.19 (m, 1H, H^1), 1.89–1.85 (m, 1H, H^5), 1.68–1.61 (m, 1H, H^4), 0.90 (d, *J* = 6.9 Hz, 3H, CH_3), 0.89 (s, 9H, Si-$C(CH_3)_3$), 0.10 (s,

6H, Si-(CH_3)$_2$); ^{13}C NMR (150 MHz, $CDCl_3$): δ 212.1 (C), 149.3 (C), 109.7 (CH), 55.4 (CH), 31.1 (CH), 29.2 (CH_2), 29.0 (CH_3), 25.6 (CH_3 × 3 and CH_2), 21.1 (CH_3), 18.0 (C), –4.4 (CH_3), –4.5 (CH_3); HRMS (ESI): *m*/*z* calcd for $C_{15}H_{28}O_2NaSi$ ([M + Na]$^+$): 291.1756, found: 291.1751.

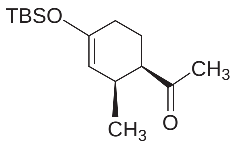

endo-S2

For **endo-S2**: IR (thin film): ν 3405, 2956, 2930, 2859, 1716, 1657, 1463, 1371, 1255, 1173, 840, 778 cm^{-1}; ^{1}H NMR (600 MHz, $CDCl_3$): δ 4.87 (dd, *J* = 5.4, 1.4 Hz, 1H, H^3), 2.73 (qdd, *J* = 6.9, 5.4, 4.5 Hz, 1H, H^2), 2.61 (ddd, *J* = 10.5, 5.1, 4.5 Hz, 1H, H^1), 2.13 (s, 3H, $COCH_3$), 2.06–1.96 (m, 2H, H^6 and H^7), 1.79–1.71 (m, 2H, H^4 and H^5), 0.89 (s, 9H, Si-C(CH_3)$_3$), 0.78 (d, *J* = 6.9 Hz, 3H, CH_3), 0.11 (s, 3H, Si-CH_3), 0.10 (s, 3H, Si-CH_3); ^{13}C NMR (150 MHz, $CDCl_3$): δ 210.8 (C), 150.3 (C), 109.4 (CH), 51.1 (CH), 30.1 (CH), 29.3 (CH_2), 28.5 (CH_3), 25.6 (CH_3 × 3), 18.5 (CH_2), 18.0 (C), 16.8 (CH_3), –4.3 (CH_3), –4.5 (CH_3); HRMS (ESI): *m*/*z* calcd for $C_{15}H_{28}O_2NaSi$ ([M + Na]$^+$): 291.1756, found: 291.1755.

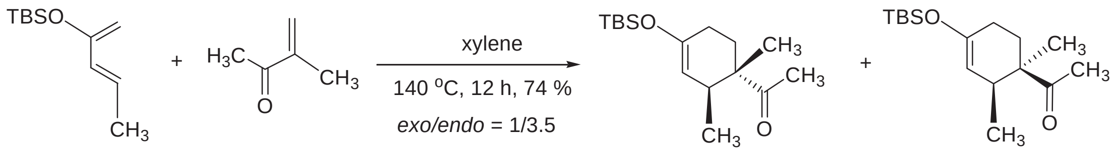

***Rac*-1-[1,2-dimethyl-4-(*tert*-butyldimethylsilyloxy)-cyclohex-3-enyl]-ethan-1-ones (exo-S5 and endo-S5).** In a 25 mL sealed-tube equipped with a magnetic stir bar, a solution of freshly distilled ketone **36** (0.425 mL, 4.25 mmol, 3.0 equiv.) and diene **S1** (0.280 g, 1.42 mmol, 1.0 equiv.) in xylene (2.8 mL) was stirred for 12 h at 140 °C. Afterwards, the reaction was concentrated *in vacuo*. The residue was purified by column chromatography on silica gel (hexanes/ethyl acetate = 20/1) to give the corresponding Diels-Alder cycloadducts **exo-S3** and **endo-S3** (0.295 g, 74%) as a colorless oil. The diastereoselectivity of the crude products was determined by ^{1}H NMR in $CDCl_3$. Integration of the signal due to the vinylic proton (H^2) of the major isomer **endo-S3** (δ_H 4.84 ppm) versus that of the minor isomer **exo-S5** (δ_H 4.65 ppm) gave an *exo*/*endo* ratio of 1/3.5.

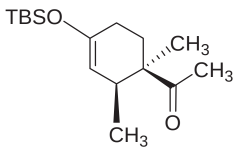

endo-S3

For **endo-S3**: IR (thin film): ν 2957, 2930, 2859, 1704, 1662, 1462, 1356, 1258, 1198, 867, 778 cm^{-1}; ^{1}H NMR (600 MHz, $CDCl_3$): δ 4.84 (d, *J* = 5.5 Hz, 1H, H^2), 2.27 (qd, *J* = 6.9, 5.5 Hz, 1H, H^1), 2.11 (s, 3H, $COCH_3$), 2.00-1.98 (m, 2H, H^5 and H^6), 1.93–1.91 (m, 1H, H^4), 1.47 (dtd, *J* = 13.4, 3.9, 1.1 Hz, 1H, H^3), 1.61 (s, 3H, CH_3), 0.90 (s, 9H , Si-$C(CH_3)_3$), 0.81 (d, *J* = 6.9 Hz, 1H, CH_3), 0.11 (s, 6H, Si $(CH_3)_2$); ^{13}C NMR (150 MHz, $CDCl_3$): δ 213.4 (C), 148.9 (C), 108.1 (CH), 48.9 (C), 36.7 (CH), 26.7 (CH_2), 25.67 (CH_3), 25.59 (CH_3 × 3), 23.8 (CH_2), 21.1 (CH_3), 18.9 (CH_3), 18.0 (C), –4.3 (CH_3), –4.5 (CH_3); HRMS (ESI): *m/z* calcd for $C_{16}H_{30}O_2NaSi$ ($[M + Na]^+$): 305.1913, found: 305.1910. The relative stereochemistry was determined by NOESY experiment.

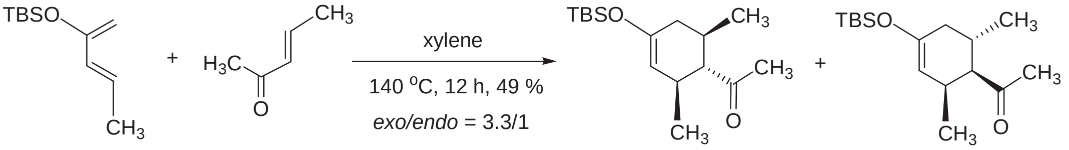

***Rac*-1-[2,6-dimethyl-4-(*tert*-butyldimethylsilyloxy)-cyclohex-3-enyl]-ethan-1-ones (exo-S4 and endo-S4).** In a 25 mL sealed tube equipped with a magnetic stir bar, a solution of freshly distilled ketone **39** (0.424 mL, 4.25 mmol, 3.0 equiv.) and diene **S1** (0.280 g, 1.42 mmol, 1.0 equiv.) in xylene (2.8 mL) was stirred for 12 h at 140 °C. Afterwards, the mixture was concentrated *in vacuo*. The residue was purified by column chromatography on silica gel (hexane/ethyl acetate = 20/1) to give the corresponding Diels–Alder cycloadducts **exo-S4** and **endo-S4** (0.195 g, 49%) as a colorless oil. The diastereoselectivity of the crude products was determined by ^{1}H NMR in $CDCl_3$. Integration of the signal due to the vinylic proton (H^3) of the major isomer **exo-S4** (δ_H 4.63 ppm) versus that of the minor isomer **endo-S4** (δ_H 4.85 ppm) gave an *exo/endo* ratio of 3.3/1.

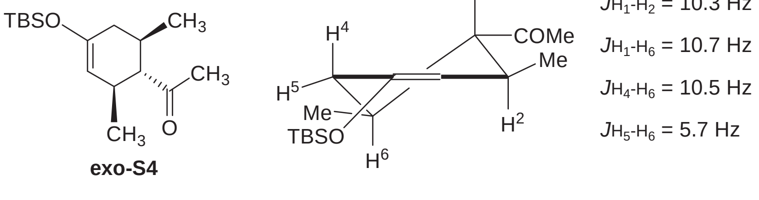

For **exo-S4**: IR (thin film): ν 2957, 2929, 2859, 1710, 1668, 1462, 1358, 1255, 1056, 836, 778 cm^{-1}; ^{1}H NMR (600 MHz, $CDCl_3$): δ 4.63 (dd, *J* = 1.8, 1.6 Hz, 1H, H^3), 2.49 (m, 1H, H^2),

2.13 (s, 3H, $COCH_3$), 2.02 (dd, J = 16.2, 5.7 Hz, 1H, H^5), 1.97 (m, 1H, H^6), 1.87 (dd, J = 10.7, 10.3 Hz, 1H, H^1), 1.76 (dddd, J = 16.2, 10.5, 3.6, 1.8 Hz, 1H, H^4), 0.89 (s, 9H, Si-$C(CH_3)_3$), 0.88 (d, J = 7.5 Hz, 3H, CH_3), 0.87 (d, J = 6.6 Hz, 3H, CH_3), 0.11 (s, 3H, Si-CH_3), 0.10 (s, 3H, Si-CH_3); ^{13}C NMR (150 MHz, $CDCl_3$): δ 213.8 (C), 149.0 (C), 109.3 (CH), 63.0 (CH), 38.2 (CH_2), 33.3 (CH), 32.1 (CH_3), 30.6 (CH_3), 25.6 (CH_3 × 3), 20.8 (CH), 19.4 (CH_3), 18.0 (C), –4.3 (CH_3), –4.5 (CH_3); HRMS (ESI): m/z calcd for $C_{16}H_{30}O_2NaSi$ ($[M + Na]^+$): 305.1913, found: 305.1918. The relative stereochemistry was determined by coupling constant analysis.

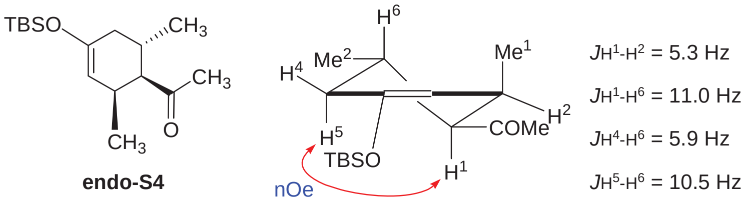

For **endo-S4**: IR (thin film): ν 2958, 2929, 2859, 1711, 1668, 1463, 1357, 1255, 1057, 837, 778 cm^{-1}; ^{1}H NMR (600 MHz, $CDCl_3$): δ 4.85 (dd, J = 5.2, 1.4 Hz, 1H, H^3), 2.61 (qdd, J = 7.0, 5.3, 5.2 Hz, 1H, H^2), 2.49 (dd, J = 11.0, 5.3 Hz, 1H, H^1), 2.14–2.11 (m, 1H, H^6), 2.12 (s, 3H, $COCH_3$), 2.05 (dd, J = 17.3, 5.9 Hz, 1H, H^4), 1.69 (dd, J = 17.3, 10.5 Hz, 1H, H^5), 0.90 (d, J = 6.9 Hz, 3H, CH_3), 0.89 (s, 9H, Si-$C(CH_3)_3$), 0.78 (d, J = 7.0 Hz, 3H, CH_3), 0.11 (s, 6H, $Si(CH_3)_2$); ^{13}C NMR (150 MHz, $CDCl_3$): δ 210.7 (C), 149.2 (C), 109.9 (CH), 57.4 (CH), 37.9 (CH_2), 30.68 (CH), 30.60 (CH_3), 25.6 (CH_3 × 3), 24.8 (CH), 19.8 (CH_3), 18.0 (C), 17.8 (CH_3), –4.39 (CH_3), –4.51 (CH_3); HRMS (ESI): m/z calcd for $C_{16}H_{30}O_2NaSi$ ($[M + Na]^+$): 305.1913, found: 305.1914. The relative stereochemistry was determined by NOESY experiment and coupling constant analysis.

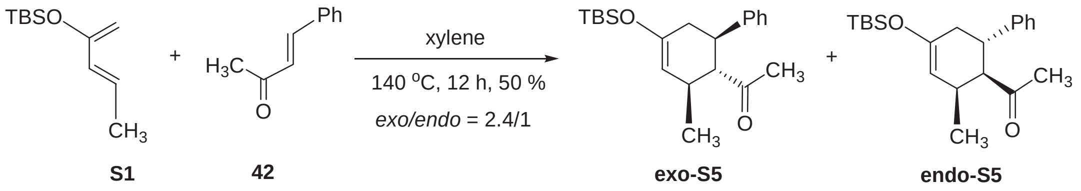

***Rac*-1-[2-methyl-6-phenyl-4-(*tert*-butyldimethylsilyloxy)-cyclohex-3-enyl]-ethan-1-ones (exo-S5 and endo-S5).** In a 25 mL sealed tube equipped with a magnetic stir bar, a solution of ketone **42** (0.413 g, 2.83 mmol, 2.0 equiv.), diene **S1** (0.280 g, 1.42 mmol, 1.0 equiv.) in xylene (2.8 mL) was stirred for 12 h at 140 °C. Afterwards, the mixture was concentrated *in vacuo*. The residue was purified by column chromatography on silica gel (hexanes/ethyl acetate = 20/1) to give the corresponding Diels–Alder cycloadducts **exo-S5**

and **endo-S5** (combined: 0.243 g, 50%) as a colorless oil. The diastereoselectivity of the crude products was determined by ^{1}H NMR in $CDCl_3$. Integration of the signal due to the vinylic proton (H^3) of the major isomer **exo-S5** (δ_H 4.73 ppm) versus that of the minor isomer **endo-S7** (δ_H 4.96 ppm) gave an *exo*/*endo* ratio of 2.1/1.

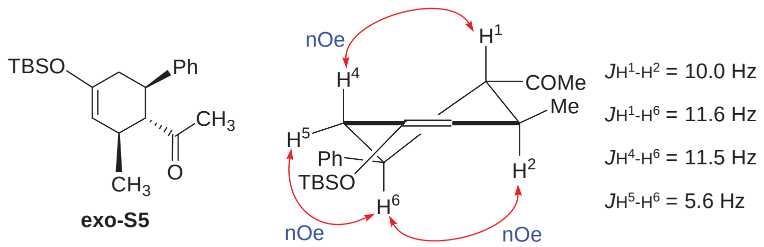

For **exo-S5**: IR (thin film): ν 3095, 2958, 2931, 2858, 1706, 1667, 1472, 1354, 1256, 1177, 839, 778 cm^{-1}; ^{1}H NMR (600 MHz, $CDCl_3$): δ 7.28–7.26 (m, 2H, Ar-H), 7.20–7.16 (m, 3H, Ar-H), 4.73 (dd, J = 2.0, 1.4 Hz, 1H, H^3), 3.05 (ddd, J = 11.6, 11.5, 5.6 Hz, 1H, H^6), 2.66–2.63 (m, 1H, H^2), 2.52 (dd, J = 11.6, 10.0 Hz, 1H, H^1), 2.33 (dddd, J = 17.2, 11.5, 3.6, 2.0 Hz, 1H, H^4), 2.22 (ddd, J = 17.2, 5.6, 1.4 Hz, 1H, H^5), 1.71 (s, 3H, $COCH_3$), 0.95 (d, J = 6.9 Hz, 3H, CH_3), 0.89 (s, 9H, Si-$C(CH_3)_3$), 0.13 (s, 6H, $Si(CH_3)_2$); ^{13}C NMR (150 MHz, $CDCl_3$): δ 212.8 (C), 148.9 (C), 142.5 (C), 128.7 (CH × 2), 127.6 (CH × 2), 126.9 (CH), 109.3 (CH), 61.5 (CH), 44.4 (CH), 38.1 (CH_2), 33.6 (CH), 31.5 (CH_3), 25.6 (CH_3 × 3), 20.9 (CH_3), 18.0 (C), –4.4 (CH_3), –4.5 (CH_3); HRMS (ESI): m/z calcd for $C_{21}H_{32}O_2NaSi$ ($[M + Na]^+$): 367.2069, found: 367.2064. The relative stereochemistry was determined by NOESY experiment and coupling constant analysis.

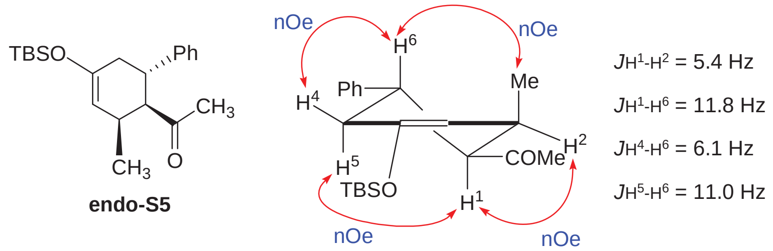

For **endo-S5**: IR (thin film): ν 3094, 2957, 2929, 2857, 1704, 1672, 1472, 1355, 1258, 1177, 839, 776 cm^{-1}; ^{1}H NMR (600 MHz, $CDCl_3$): δ 7.27–7.25 (m, 2H, Ar-H), 7.19–7.14 (m, 3H, Ar-H), 4.96 (dd, J = 5.3, 1.7 Hz, 1H, H^3), 3.28–3.23 (m, 2H, H^1 and H^6), 2.79–2.75 (m, 1H, H^2), 2.26 (dd, J = 17.7, 5.1 Hz, 1H, H^4), 2.16–2.11 (m, 1H, H^5), 1.97 (s, 3H, $COCH_3$), 0.92 (d, J = 6.9 Hz, 3H, CH_3), 0.90 (s, 9H, Si-$C(CH_3)_3$), 0.15 (s, 3H, Si-CH_3), 0.14 (s, 3H, Si-CH_3); ^{13}C NMR (150 MHz, $CDCl_3$): δ 209.6 (C), 149.1 (C), 144.8 (C), 128.4 (CH × 2), 127.1 (CH × 2), 126.1 (CH), 108.9 (CH), 55.6 (CH), 39.1 (CH_2), 37.0 (CH), 30.7 (CH), 30.4 (CH_3), 25.5 (CH_3 × 3), 17.9 (CH_3), 17.8 (C), –4.4 (CH_3), –4.5 (CH_3) ; ^{1}H NMR (600 MHz, C_6D_6): δ 7.12–

7.10 (m, 2H, Ar-H), 7.07–7.03 (m, 2H, Ar-H), 7.02–7.00 (m, 1H, Ar-H), 4.94 (d, *J* = 3.8 Hz, 1H, H^3), 3.28 (ddd, *J* = 11.8, 11.0, 6.1 Hz, 1H, H^6), 2.94 (dd, *J* = 11.8, 5.4 Hz, 1H, H^1), 2.46 (qdd, *J* = 7.0, 5.4, 3.8 Hz, 1H, H^2), 2.28 (dd, *J* = 17.5, 6.1 Hz, 1H, H^4), 2.16 (dd, *J* = 17.5, 11.0 Hz, 1H, H^5), 1.57 (s, 3H, $COCH_3$), 0.97 (s, 9H, Si-C(CH_3)$_3$), 0.89 (d, *J* = 7.0 Hz, 3H, CH_3), 0.15 (s, 3H, CH_3), 0.14 (s, 3H, Si-CH_3); ^{13}C NMR (150 MHz, C_6D_6): δ 207.2 (C), 149.5 (C), 145.4 (C), 128.6 (CH × 2), 128.2 (CH × 2), 126.3 (CH), 108.7 (CH), 55.5 (CH), 39.4 (CH_2), 37.5 (CH), 30.9 (CH), 30.1 (CH_3), 25.8 (CH_3 × 3), 18.2 (CH_3), 18.1 (C), –4.35 (CH_3), –4.38 (CH_3); HRMS (ESI): *m*/*z* calcd for $C_{21}H_{32}O_2NaSi$ ([M + Na]$^+$): 367.2069, found: 367.2065.

2.5 Diels–Alder reactions with α,β-unsaturated esters

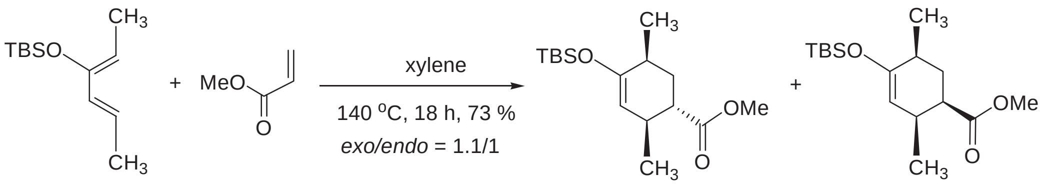

7 **45** **exo-46** **endo-46**

***Rac*-1-[2,5-dimethyl-4-(*tert*-butyldimethylsilyloxy)-cyclohex-3-enyl]-carboxylates (exo-46 and endo-46).** In a 25 mL sealed tube equipped with a magnetic stir bar, a solution of freshly distilled ester **45** (0.365 g, 4.25 mmol, 3.0 equiv.), diene **7** (0.300 g, 1.20 mmol, 1.0 equiv.) in xylene (2.8 mL) was stirred for 18 h at 140 °C. Afterwards, the mixture was concentrated *in vacuo*. The residue was purified by column chromatography on silica gel (hexanes/ethyl acetate = 20/1) to give the corresponding Diels–Alder cycloadducts **exo-46** and **endo-46** (0.262 g, 73%) as a colorless oil. The diastereoselectivity of the crude products was determined by ^{1}H NMR in C_6D_6. Integration of the signal due to the vinylic proton H^3 of **exo-46** (δ_H 4.67 ppm) versus that of **endo-46** (δ_H 4.81 ppm) gave an *exo*/*endo* ratio of 1.1/1.

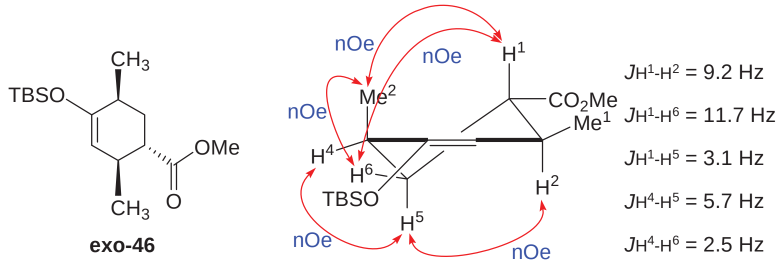

For **exo-46**: IR (thin film): ν 2958, 2932, 2859, 1740, 1661, 1463, 1361, 1253, 1194, 1175, 839, 779 cm^{-1}; ^{1}H NMR (600 MHz, C_6D_6): δ 4.67 (d, *J* =2.1 Hz, 1H, H^3), 3.37 (s, 3H, OMe), 2.78 (dqd, *J* = 2.1, 7.2, 9.2 Hz, 1H, H^2), 2.26 (ddd, *J* = 3.1, 9.2, 11.7 Hz, 1H, H^1), 2.14 (ddq, *J*

= 2.5, 5.7, 7.1 Hz, 1H, H^4), 2.02 (ddd, J =5.7, 11.7, 13.3 Hz, 1H, H^5), 1.62 (ddd, J =2.5, 3.1, 13.3 Hz, 1H, H^6), 1.05 (d, J = 7.2 Hz, 3H, Me^2), 0.99 (d, J = 7.1 Hz, 3H, Me^1), 0.96 (s, 9H, Si-C(CH_3)$_3$), 0.11 (s, 6H, Si-(CH_3) $_2$); ^{13}C NMR (150 MHz, C_6D_6): δ 175.6 (C), 153.9 (C), 108.1 (CH), 50.5 (CH_3), 44.0 (CH), 33.4 (CH_2), 33.1 (CH), 32.8 (CH), 25.9 (CH_3 × 3), 21.5 (CH_3), 18.9 (CH_3), 18.2 (C), –4.3 (CH_3), –4.5 (CH_3); HRMS (ESI): *m/z* calcd for $C_{16}H_{30}O_3NaSi$ ([M + Na]$^+$): 321.1862, found 321.1860. The relative stereochemistry was determined by NOESY experiment and coupling constant analysis.

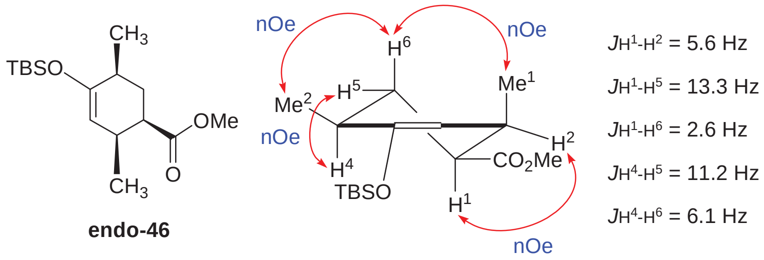

endo-46

For **endo-46**: IR (thin film): ν 2958, 2930, 2858, 1739, 1689, 1463, 1253, 1191, 1174, 837, 778 cm^{-1}; ^{1}H NMR (600 MHz, C_6D_6): δ 4.81 (dd, J =1.5, 5.6 Hz, 1H, H^3), 3.37 (s, 3H, OMe), 2.72 (ddq, J = 5.6, 5.6, 6.9 Hz, 1H, H^2) , 2.56 (ddd, J = 2.6, 5.6, 13.3 Hz, 1H, H^1), 2.05 (ddqd, J = 1.5, 6.1, 6.9, 11.2 Hz, 1H, H^4), 1.89 (ddd, J =2.6, 6.1, 13.8 Hz, 1H, H^5), 1.66 (ddd, J =11.2, 13.3, 13.8 Hz, 1H, H^6), 1.07 (d, J = 6.9 Hz, 3H, Me^2), 0.97 (s, 9H, Si-C(CH_3)$_3$), 0.94 (d, J = 6.9 Hz, 3H, Me^1), 0.10 (s, 3H, Si-CH_3), 0.09 (s, 3H, Si-CH_3); ^{13}C NMR (150 MHz, C_6D_6): δ 174.2 (C), 154.0 (C), 108.4 (CH), 50.9 (CH_3), 43.7 (CH), 34.5 (CH), 31.3 (CH), 29.2 (CH_2), 25.9 (CH_3 × 3), 18.7 (CH_3), 18.3 (C), 17.7 (CH_3), –4.3 (CH_3), –4.6 (CH_3); HRMS (ESI): *m/z* calcd for $C_{16}H_{30}O_3NaSi$ ([M + Na]$^+$): 321.1862, found 321.1855. The relative stereochemistry was determined by NOESY experiment and coupling constant analysis.

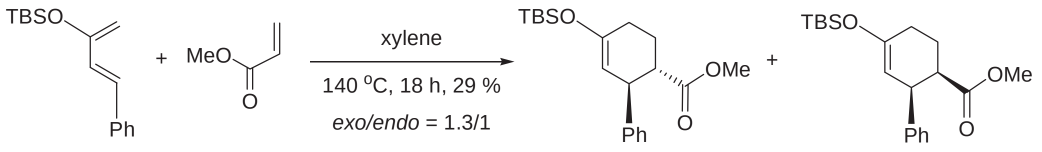

8 **45** **exo-47** **endo-47**

***Rac*-1-[2-phenyl-4-(*tert*-butyldimethylsilyloxy)-cyclohex-3-enyl]-carboxylates (*exo*-47 and *endo*-47).** In a 25 mL sealed tube equipped with a magnetic stir bar, a solution of freshly distilled ester **45** (0.595 g, 6.92 mmol, 3.0 equiv.) and diene **8** (0.600 g, 2.31 mmol, 1.0 equiv.) in xylene (4.6 mL) was stirred for 18 h at 140 °C. Afterwards, the reaction was concentrated *in vacuo*. The residue was purified by column chromatography on silica gel (hexanes/ethyl acetate = 20/1) to give the corresponding Diels–Alder cycloadducts **exo-47**

and **endo-47** (0.231 g, 29%) as a colorless oil. The diastereoselectivity of the crude products was determined by ^{1}H NMR in $CDCl_3$. Integration of the signal due to OMe of **exo-47** (δ_H 3.52 ppm) versus that of **endo-47** (δ_H 3.45 ppm) gave an *exo*/*endo* ratio of 1.3/1.

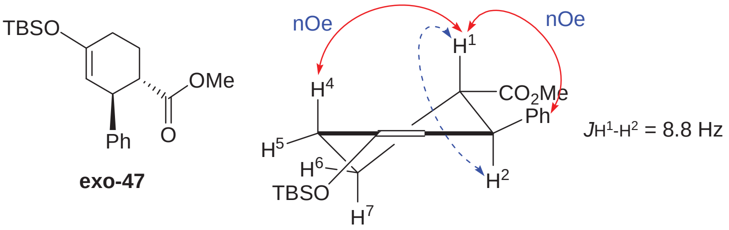

For **exo-47**: IR (thin film): ν 3029, 2955, 2929, 2857, 1738, 1668, 1602, 1463, 1254, 1174, 837, 779, 700 cm^{-1}; ^{1}H NMR (600 MHz, $CDCl_3$): δ 7.27–7.25 (m, 3H, Ar-H), 7.19–7.18 (m, 3H, Ar-H), 4.82 (ddd, *J* = 1.0, 1.0, 3.6 Hz, 1H, H^3), 3.80 (dd, 1H, *J* = 3.6, 8.8 Hz, 1H, H^2), 3.53 (s, 3H, OMe), 2.46 (ddd, *J* = 4.1, 8.8, 10.1 Hz, 1H, H^1), 2.24–2.19 (m, 2H, H^4), 2.13–2.12 (m, 2H, H^5), 1.96–1.94 (m, 1H, H^6), 1.93–1.92 (m, 1H, H^7), 0.91 (s, 9H, Si-$C(CH_3)_3$), 0.14 (s, 3H, Si-CH_3), 0.12 (s, 3H, Si-CH_3); ^{13}C NMR (150 MHz, $CDCl_3$): δ 175.4 (C), 150.8 (C), 144.7 (C), 128.4 (CH × 2), 127.9 (CH × 2), 126.6 (CH), 107.0 (CH), 51.5 (CH_3), 48.5 (CH), 43.8 (CH), 28.8 (CH_2), 25.7 (CH_3 × 3), 25.5 (CH_2), 18.0 (C), –4.4 (CH_3 × 2); HRMS (ESI): *m/z* calcd for $C_{20}H_{30}O_3NaSi$ ($[M+ Na]^+$): 369.1862, found 369.1867. The relative stereochemistry was determined by NOESY experiment and coupling constant analysis.

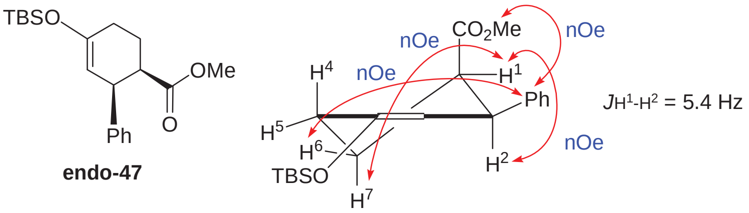

For **endo-47**: IR (thin film): ν 3029, 2955, 2929, 2857, 1738, 1668, 1602, 1463, 1254, 1205, 837, 779, 700 cm^{-1}; ^{1}H NMR (600 MHz, $CDCl_3$): δ 7.25–7.23 (m, 2H, Ar-H), 7.19–7.17 (m, 1H, Ar-H), 7.16–7.15 (m, 2H, Ar-H), 4.93 (ddd, *J* = 1.6, 1.6, 5.2 Hz, 1H, H^3), 3.91 (dd, 1H, *J* = 5.2, 5.9 Hz, 1H, H^2), 3.45 (s, 3H, OMe), 2.86 (ddd, *J* = 3.0, 5.9, 12.7 Hz, 1H, H^1), 2.20–2.16 (m, 2H, H^4 and H^5), 1.93–1.86 (m, 1H, H^6), 1.78–1.75 (m, 1H, H^7), 0.92 (s, 9H, Si-$C(CH_3)_3$), 0.16 (s, 3H, Si-CH_3), 0.15 (s, 3H, Si-CH_3); ^{13}C NMR (150 MHz, $CDCl_3$): δ 174.0 (C), 151.9 (C), 141.2 (C), 129.2 (CH × 2), 127.9 (CH × 2), 126.9 (CH), 105.8 (CH), 51.1 (CH_3), 44.9 (CH), 42.3 (CH), 29.0 (CH_2), 25.7 (CH_3 × 3), 19.5 (CH_2), 18.0 (C), –4.3 (CH_3); –4.4 (CH_3); HRMS (ESI): *m/z* calcd for $C_{20}H_{30}O_3NaSi$ ($[M+ Na]^+$): 369.1862, found 369.1866. The relative stereochemistry was determined by NOESY experiment and coupling constant analysis.

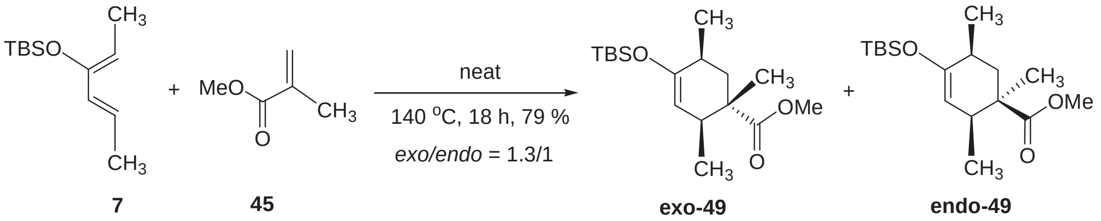

***Rac*-1-[1,2,5-trimethyl-4-(*tert*-butyldimethylsilyloxy)-cyclohex-3-enyl]-carboxylates (exo-49 and exo-49).** In a 25 mL sealed tube equipped with a magnetic stir bar, ester **45** (0.425 g, 4.25 mmol, 3.0 equiv.) and diene **7** (0.300 g, 1.20 mmol, 1.0 equiv.) were stirred for 18 h at 140 °C. Afterwards, the reaction was concentrated *in vacuo*. The residue was purified by column chromatography on silica gel (hexanes/ethyl acetate = 20/1) to give the corresponding Diels-Alder cycloadducts **exo-49** and **endo-49** (0.296 g, 79%) as a colorless oil. The diastereoselectivity of the crude products was determined by ^{1}H NMR in C_6D_6. Integration of the signal due to the vinylic proton (H^2) of **exo-49** (δ_H 4.95 ppm) versus that of **endo-49** (δ_H 4.75 ppm) gave an *exo*/*endo* ratio of 1.3/1.

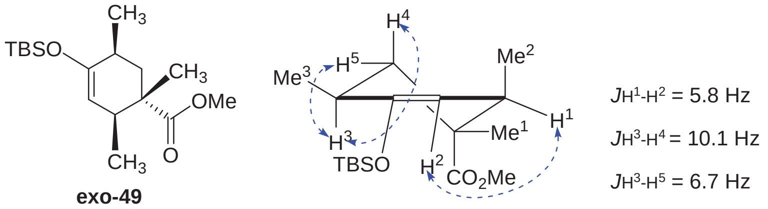

For **exo-49**: IR (thin film): ν 2958, 2933, 2859, 1737, 1662, 1464, 1361, 1257, 1199, 1184, 840, 778 cm^{-1}; ^{1}H NMR (600 MHz, C_6D_6): δ 4.95 (d, J = 5.8 Hz, 1H, H^2), 3.36 (s, 3H, OMe), 2.92 (dq, J =5.8, 7.1 Hz, 1H, H^1), 2.54 (dqd, J = 6.7, 6.9, 10.1 Hz, 1H, H^3), 2.09 (dd, J = 6.7, 13.5 Hz, 1H, H^5), 1.22 (dd, J = 10.1, 13.5 Hz, 1H, H^4) 1.12 (d, J = 6.9 Hz, 3H, Me^3), 1.02 (s, 3H, Me^1), 0.97 (s, 9H, Si-C(CH_3)$_3$), 0.85 (d, J = 7.1 Hz, 3H, Me^2), 0.13 (s, 3H, Si-CH_3), 0.11 (s, 3H, Si-CH_3); ^{13}C NMR (150 MHz, C_6D_6): δ 177.4 (C), 153.6 (C), 110.1 (CH), 51.3 (CH_3), 45.2 (C), 36.9 (CH_2), 34.7 (CH), 32.8 (CH), 25.9 (CH_3 × 3), 24.0 (CH_3), 19.3 (CH_3), 18.3 (C), 17.6 (CH_3), –4.2 (CH_3), –4.7 (CH_3); HRMS (ESI): *m/z* calcd for $C_{17}H_{32}O_3NaSi$ ($[M + Na]^+$): 335.2018, found: 335.2012. The relative stereochemistry was determined by NOESY experiment and coupling constant analysis.

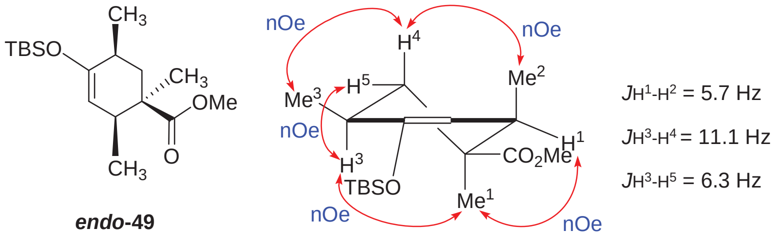

For **endo-49**: IR (thin film): ν 2957, 2932, 2859, 1734, 1662, 1464, 1361, 1259, 1197, 1183, 839, 779 cm^{-1}; ^{1}H NMR (600 MHz, C_6D_6): δ 4.75 (d, J = 5.7 Hz, 1H, H^2), 3.35 (s, 3H, OMe), 2.33 (dq, J = 5.7, 6.8 Hz, 1H, H^1), 2.07 (dqd, J = 6.3, 6.8, 11.1 Hz, 1H, H^3), 1.90 (dd, J =11.1, 13.9 Hz, 1H, H^4), 1.80 (dd, 1H, J = 6.3, 13.9 Hz, H^5), 1.31 (s, 3H, Me^1), 1.11 (d, J = 6.8 Hz, 3H, Me^3), 0.97 (s, 9H, Si-C(CH_3)$_3$), 0.97 (d, J = 6.8Hz, 3H, Me^2), 0.11 (s, 3H, Si-CH_3), 0.09 (s, 3H, Si-CH_3); ^{13}C NMR (150 MHz, C_6D_6): δ 177.1(C), 152.6 (C), 107.1 (CH), 51.0 (CH_3), 44.7 (C), 38.1 (CH), 34.7 (CH_2), 31.4 (CH), 25.9 (CH_3 × 3), 22.7 (CH_3), 19.8 (CH_3), 18.7 (CH_3), 18.3 (C), –4.2(CH_3), –4.6 (CH_3); HRMS (ESI): *m/z* calcd for $C_{17}H_{32}O_3NaSi$ ([M + Na]$^+$): 335.2018, found: 335.2018. The relative stereochemistry was determined by NOESY experiment and coupling constant analysis.

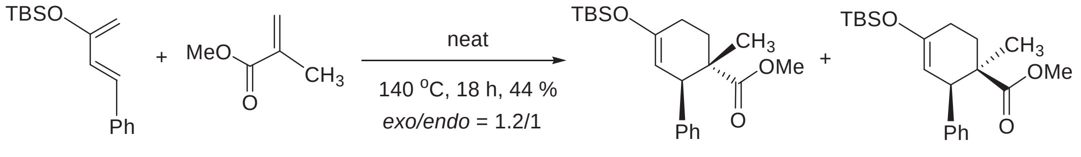

***Rac*-1-[1-methyl-2-phenyl-4-(*tert*-butyldimethylsilyloxy)-cyclohex-3-enyl]-carboxylates (*exo*-50 and *endo*-50).** In a 25 mL sealed tube equipped with a magnetic stir bar, ester **48** (0.692 g, 6.92 mmol, 3.0 equiv.) and diene **8** (0.600 g, 2.31 mmol, 1.0 equiv.) were stirred for 18 h at 140 °C. Afterwards, the mixture was concentrated *in vacuo*. The residue was purified by column chromatography on silica gel (hexanes/ethyl acetate = 20/1) to give the corresponding Diels–Alder cycloadducts **exo-50** and **endo-50** (combined: 0.366 g, 44%) as a colorless oil. The diastereoselectivity of the crude products was determined by ^{1}H NMR in $CDCl_3$. Integration of the signal due to OMe of **exo-50** (δ_H 3.35 ppm) versus that of the **endo-50** (δ_H 3.08 ppm) gave an *exo/endo* ratio of 1.2:1.

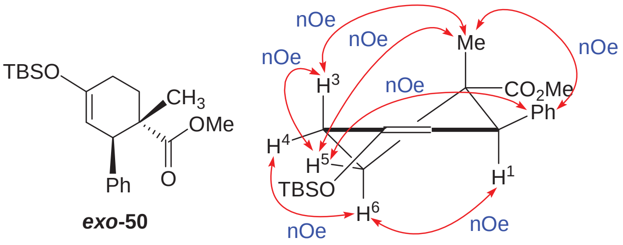

For **exo-50**: m.p. 61–62 °C; IR (thin film): ν 3024, 2952, 2931, 2858, 1730, 1668, 1600, 1463, 1367, 1253, 1197, 1174, 838, 779, 703 cm^{-1}; ^{1}H NMR (600 MHz, C_6D_6): δ 7.23 (d, J = 7.6 Hz, 2H, Ar-H), 7.13 (dd, J = 7.6, 7.4 Hz, 2H, Ar-H), 7.07 (d, J= 7.4 Hz, 1H, Ar-H), 5.07 (d, J = 4.4 Hz, 1H, H^2), 4.31 (d, J = 4.4 Hz, 1H, H^1), 3.35 (s, 3H, OMe), 2.32 (ddd, J = 6.2, 7.6, 17.3 Hz, 1H, H^4), 2.11 (ddd, J = 6.6, 6.8, 17.3 Hz, 1H, H^3), 2.05 (ddd, J = 6.2, 6.6, 13.2 Hz,

1H, H^6), 1.59 (ddd, J = 6.8, 7.6, 13.2 Hz, 1H, H^5), 0.97 (s, 9H, Si-$C(CH_3)_3$), 0.91 (s, 3H, Me), 0.12 (s, 3H, Si-CH_3), 0.11 (s, 3H, Si-CH_3); ^{13}C NMR (150 MHz, C_6D_6): δ 177.1(C), 151.3 (C), 142.2 (C), 130.2 (CH × 2), 128.3 (CH × 2), 127.0 (CH), 107.2 (CH), 51.4 (CH_3), 47.0 (CH), 45.6 (C), 29.9 (CH_2), 27.7 (CH_2), 25.9 (CH_3 × 3), 21.6 (CH_3), 18.2 (C), –4.2 (CH_3), –4.4 (CH_3); HRMS (ESI): *m/z* calcd for $C_{21}H_{32}O_3NaSi$ ([M – H]$^-$): 383.2018, found: 383.2012. The relative stereochemistry was determined by NOESY experiment analysis.

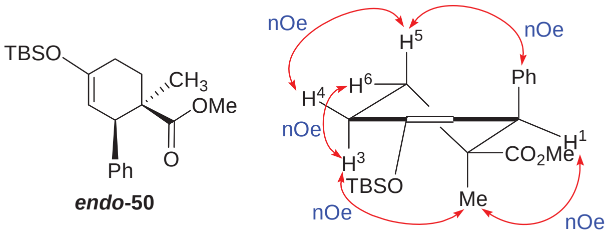

***endo*-50**

For **endo-50**: IR (thin film): ν 3024, 2952, 2931, 2858, 1731, 1668, 1602, 1460, 1368, 1258, 1205, 1176, 838, 779, 704 cm^{-1}; ^{1}H NMR (600 MHz, C_6D_6): δ 7.27 (d, J = 7.2 Hz, 2H, Ar-H), 7.13 (dd, J = 7.2, 7.7 Hz, 3H, Ar-H), 4.90 (dd, J = 1.5, 5.2 Hz, 1H, H^2), 3.50 (d, J = 5.2 Hz, 1H, H^1), 3.08 (s, 3H, OMe), 2.34 (ddd, J = 6.8, 11.5, 14.0 Hz, 1H, H^5), 2.15 (dddd, J = 1.5, 6.6, 6.8, 17.8 Hz, 1H, H^4), 2.04 (dddd, J = 1.5, 6.6, 11.5, 17.8 Hz, 1H, H^3), 1.53 (ddd, J = 6.6, 6.8, 14.0 Hz, 1H, H^6), 1.40 (s, 3H, Me), 0.98 (s, 9H, Si-$C(CH_3)_3$), 0.11 (s, 3H, Si-CH_3), 0.10 (s, 3H, Si-CH_3); ^{13}C NMR (150 MHz, C_6D_6): δ 175.7 (C), 150.7 (C), 142.5 (C), 129.9 (CH × 2), 128.3 (CH × 2), 127.0 (CH), 105.2 (CH), 50.7 (CH_3), 50.1 (CH), 45.9 (C), 27.0 (CH_2), 25.9 (CH_2), 25.8 (CH_3 × 3), 23.0 (CH_3), 18.1 (C), –4.2 (CH_3), –4.4 (CH_3); HRMS (ESI): *m/z* calcd for $C_{21}H_{32}O_3NaSi$ ([M – H]$^-$): 383.2018, found: 383.2013. The relative stereochemistry was determined by NOESY experiment analysis.

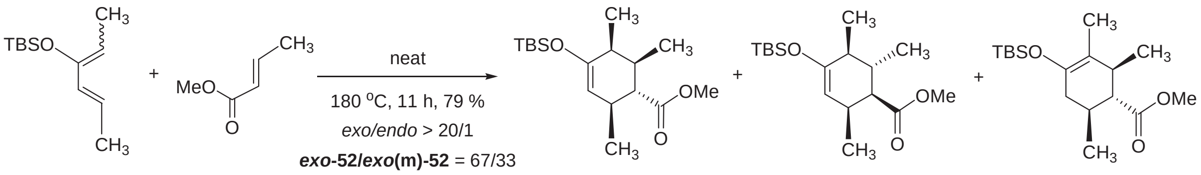

7 **51** **exo-52** **endo-52** **exo(m)-52**

***Rac*-1-[2,5,6-trimethyl-4-(*tert*-butyldimethylsilyloxy)-cyclohex-3-enyl]-carboxylates (exo-52 and endo-52) and the corresponding *exo* olefin migrator exo(m)-52.** In a 25 mL sealed tube equipped with a magnetic stir bar, freshly distilled ester **51** (0.426 g, 4.25 mmol, 3.0 equiv.) and diene **7** (0.300 g, 1.20 mmol, 1.0 equiv.) were for 11 h at 180 °C. Afterwards, the mixture was concentrated *in vacuo*. The residue was purified by column chromatography on silica gel (hexanes/ethyl acetate = 20/1) to give the corresponding Diels–Alder cycloadducts **exo-52** and **exo(m)-52** (0.296 g, 79%) as a colorless oil. The

diastereoselectivity of the crude products was determined by ^{1}H NMR in C_6D_6. Integration of the signal due to the vinylic proton (H^3) of **exo-52** (δH 4.65 ppm) versus the H^1 of the corresponding *exo* olefin migrator **exo(m)-52** (δ_H 1.95 ppm) gave the **exo-52/exo(m)-52** ratio as 67:33.

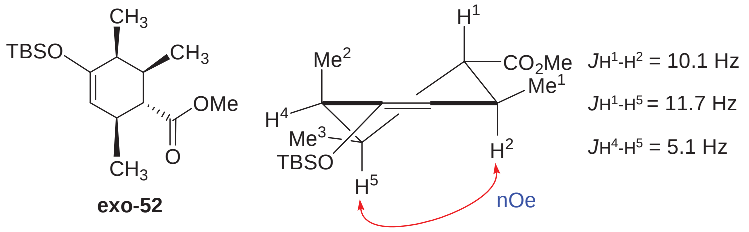

exo-52

For **exo-52**: IR (thin film): ν 2959, 2931, 2859, 1739, 1667, 1362, 1253, 1195, 1164, 838, 779 cm^{-1}; ^{1}H NMR (600 MHz, C_6D_6): δ 4.65 (d, *J* = 2.0 Hz, 1H, H^3), 3.38 (s, 3H, OMe), 2.78 (dqd, *J* = 2.0, 7.1, 10.1 Hz, 1H, H^2), 2.22 (dqd, *J* = 5.1, 6.8, 11.7 Hz, 1H, H^5), 2.09 (dd, *J* = 10.1, 11.7 Hz, 1H, H^1), 2.02 (dq, *J* = 5.1, 7.1 Hz, 1H, H^4), 0.97 (s, 9H, Si-C(CH_3)$_3$), 0.96 (d, *J* = 7.1 Hz, 3H, Me^1), 0.94 (d, *J* = 7.0 Hz, 3H, Me^2), 0.85 (d, *J* = 6.8 Hz, 3H, Me^3), 0.12 (s, 6H, Si-(CH_3)$_2$); ^{13}C NMR (150 MHz, C_6D_6): δ 175.8 (C), 154.6 (C), 107.4 (CH), 50.8 (CH_3), 50.4 (CH), 39.1 (CH), 35.4(CH), 34.2(CH), 25.9 (CH_3 × 3), 21.4 (CH_3), 18.2 (C), 16.3 (CH_3), 13.0 (CH_3), –4.2 (CH_3), –4.6 (CH_3); HRMS (ESI): *m/z* calcd for $C_{17}H_{33}O_3Si$ ([M+H]$^+$): 313.2199, found: 313.2196. The relative stereochemistry was determined by NOESY experiment and coupling constant analysis.

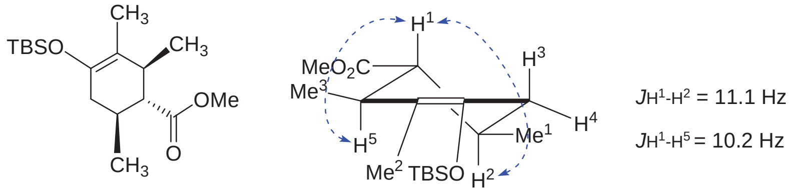

exo(m)-52

For **exo(m)-52**: IR (thin film): ν 2958, 2931, 2858, 1738, 1682, 1463, 1362, 1253, 1196, 1173, 837, 778 cm^{-1}; ^{1}H NMR (600 MHz, C_6D_6): δ 3.38 (s, 3H, OMe), 2.67 (qd, *J* = 7.0, 10.2Hz, H^5), 2.16–2.09 (m, 2H, H^2 and H^3), 2.04 (dd, *J* = 10.2, 11.1 Hz, 1H, H^1), 1.88–1.83 (m, 1H, H^4), 1.61 (s, 3H, Me^2), 1.00 (s, 9H, Si-C(CH_3)$_3$), 0.99 (d, *J* = 7.0 Hz, 3H, Me^3), 0.86 (d, 1H, *J* = 6.2 Hz, 3H, Me^1), 0.07 (s, 3H, Si-CH_3), 0.07 (s, 3H, Si-CH_3); ^{13}C NMR (150 MHz, C_6D_6): δ 175.7 (C), 142.6 (C), 114.1 (C), 57.0 (CH), 50.8 (CH_3), 39.1 (CH_2), 37.9 (CH), 32.1 (CH), 26.0 (CH_3 × 3), 19.7 (CH_3), 19.1 (CH_3), 18.4 (C), 13.5 (CH_3), –3.7 (CH_3), –4.0 (CH_3); HRMS (APCI): *m/z* calcd for $C_{17}H_{33}O_3Si$ ([M+H]$^+$): 313.2199, found: 313.2193. The relative stereochemistry was determined by coupling constant analysis.

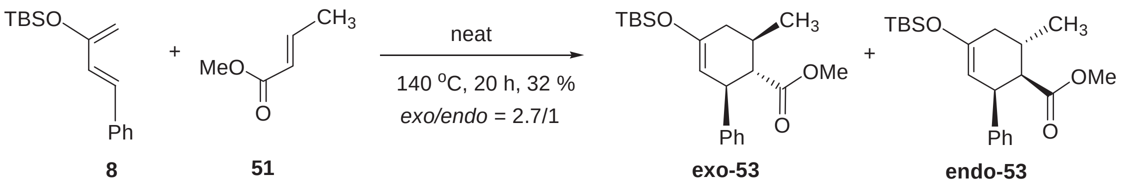

***Rac*-1-[6-methyl-2-phenyl-4-(*tert*-butyldimethylsilyloxy)-cyclohex-3-enyl]-carboxylates (exo-53 and endo-53).** In a 25 mL sealed tube equipped with a magnetic stir bar, ester **51** (0.692 g, 6.92 mmol, 3.0 equiv.) and diene **8** (0.600 g, 2.31 mmol, 1.0 equiv.) were stirred for 20 h at 140 °C. Afterwards, the mixture was concentrated *in vacuo*. The residue was purified by column chromatography on silica gel (hexanes/ethyl acetate = 20/1) to give the corresponding Diels–Alder cycloadducts **exo-53** and **endo-53** (0.266 g, 32%) as a colorless oil. The diastereoselectivity of the crude products was determined by ^{1}H NMR in C_6D_6. Integration of the signal due to H^2 of **exo-53** (δ_H 4.00 ppm) versus that of **endo-53** (δ_H 3.88 ppm) gave an *exo*/*endo* ratio of 2.7:1.

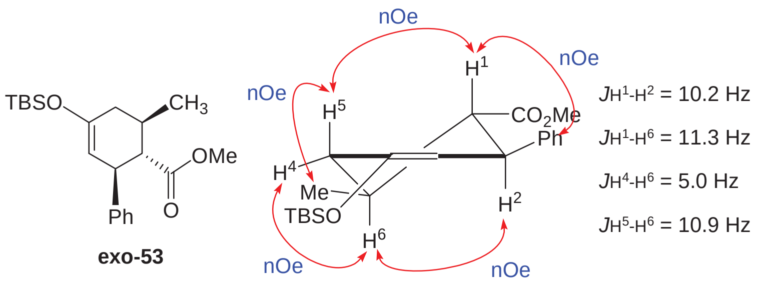

For **exo-53**: IR (thin film): ν 3028, 2954, 2930, 2858, 1735, 1671, 1602, 1460, 1368, 1258, 1203, 1173, 839, 800, 701 cm^{-1}; ^{1}H NMR (600 MHz, C_6D_6): δ 7.26 (d, *J*= 7.2 Hz, 2H, Ar-H), 7.15 (dd, *J*= 7.2, 7.4 Hz, 2H, Ar-H), 7.04 (dd, *J*= 7.4, 7.4 Hz, 1H, Ar-H), 4.94 (dd, *J* = 1.9, 2.1 Hz, 1H, H^3), 4.00 (ddd, *J* = 1.9, 3.8, 10.2 Hz, 1H, H^2), 3.17 (s, 3H, OMe), 2.29 (dd, *J* = 10.2, 11.3 Hz, 1H, H^1), 2.22 (dqdd, *J* = 5.0, 6.3, 10.9, 11.3 Hz, 1H, H^6), 2.08 (ddd, *J* = 2.1, 5.0, 17.4 Hz, 1H, H^4), 1.86 (dddd, *J* = 2.1, 3.8, 10.9, 17.4 Hz, 1H, H^5), 0.97 (s, 9H, Si-C(CH_3)$_3$), 0.86 (d, *J* = 6.3Hz, 3H, Me), 0.10 (s, 3H, Si-CH_3), 0.08 (s, 3H, Si-CH_3); ^{13}C NMR (150 MHz, C_6D_6): δ 174.9 (C), 150.6 (C), 145.1 (C), 128.8 (CH × 2), 128.1 (CH × 2), 127.0 (CH), 107.2 (CH), 57.1 (CH), 50.6 (CH_3), 46.3 (CH), 38.3 (CH_2), 33.0 (CH), 25.8 (CH_3 × 3), 19.6 (CH_3), 18.1 (C), –4.3 (CH_3), –4.4 (CH_3); HRMS (ESI): *m/z* calcd for $C_{21}H_{32}O_3NaSi$ ([M + Na]$^+$): 383.2018, found: 383.2011. The relative stereochemistry was determined by NOESY experiment and coupling constant analysis.

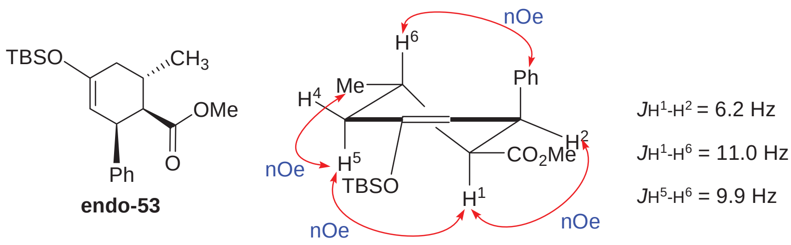

For **endo-53**: IR (thin film): ν 3029, 2955, 2930, 2858, 1737, 1669, 1602, 1462, 1368, 1258, 1204, 1166, 840, 800, 701 cm^{-1}; ^{1}H NMR (600 MHz, C_6D_6): δ 7.20 (d, *J*= 7.1 Hz, 2H, Ar-H), 7.14 (dd, *J*= 7.1, 7.2 Hz, 2H, Ar-H), 7.07 (dd, *J*= 7.2, 7.2 Hz, 1H, Ar-H), 4.95 (dd, *J* = 2.0, 5.0 Hz, 1H, H^3), 3.88 (dd, *J* = 5.0, 6.2 Hz, 1H, H^2), 3.15 (s, 3H, OMe), 2.61 (dd, *J* = 6.2, 11.0 Hz, 1H, H^1), 2.26 (m, 2H, H^6 and H^4), 1.75 (ddd, *J* = 2.0, 9.9, 17.1 Hz, 1H, H^5), 0.99 (s, 9H, Si-C(CH_3)$_3$), 0.91 (d, *J* = 6.2Hz, 3H, Me), 0.12 (s, 3H, Si-CH_3), 0.12 (s, 3H, Si-CH_3); ^{13}C NMR (150 MHz, C_6D_6): δ 172.6 (C), 151.2 (C), 142.2 (C), 129.5 (CH × 2), 128.2 (CH × 2), 127.1 (CH), 105.3 (CH), 52.5 (CH), 50.4 (CH_3), 43.9 (CH), 37.7 (CH_2), 25.9 (CH_3 × 3), 25.8 (CH), 20.2 (CH_3), 18.2 (C), –4.2 (CH_3), –4.4 (CH_3); HRMS (ESI): *m/z* calcd for $C_{21}H_{32}O_3NaSi$ ([M + Na]$^+$): 383.2018, found: 383.2012. The relative stereochemistry was determined by NOESY experiment and coupling constant analysis.

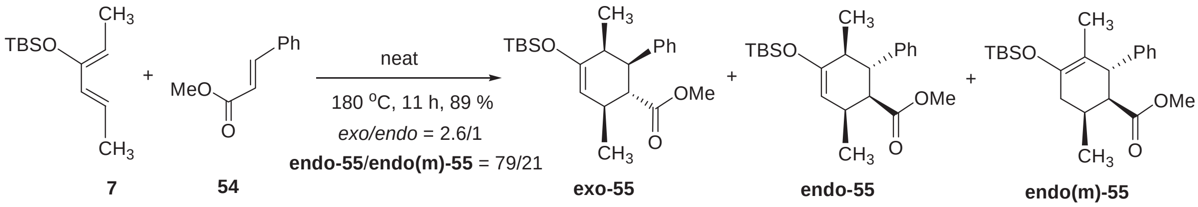

***Rac*-1-[2,5-dimethyl-6-phenyl-4-(*tert*-butyldimethylsilyloxy)-cyclohex-3-enyl]-carboxylates (*exo*-55 and *endo*-55) and the corresponding *exo* olefin migrator *endo*(m)-55.** In a 25 mL sealed tube equipped with a magnetic stir bar, ester **54** (0.458 g, 2.83 mmol, 2.0 equiv.) and diene **7** (0.300 g, 1.20 mmol, 1.0 equiv.) were stirred for 11 h at 180 °C. Afterwards, the mixture was concentrated *in vacuo*. The residue was purified by column chromatography on silica gel (hexanes/ethyl acetate = 20/1) to give the corresponding Diels–Alder cycloadducts **exo-55**, **endo-55** and **endo(m)-55** (combined: 0.400 g, 89%) as a colorless oil. The diastereoselectivity of the crude products was determined by ^{1}H NMR in C_6D_6. Integration of the signal due to the vinylic proton (H^3) of the major adduct **exo-55** (δ_H 4.73 ppm) versus that of the minor adduct **endo-55** (δ_H 4.97 ppm) and H^5 of the corresponding *endo* olefin migrator ***endo*(m)-55** (δ_H 3.89 ppm) gave an apparent *exo*/*endo* ratio of 2.6/1 (the **endo-55/endo(m)-55** ratio is 79/21).

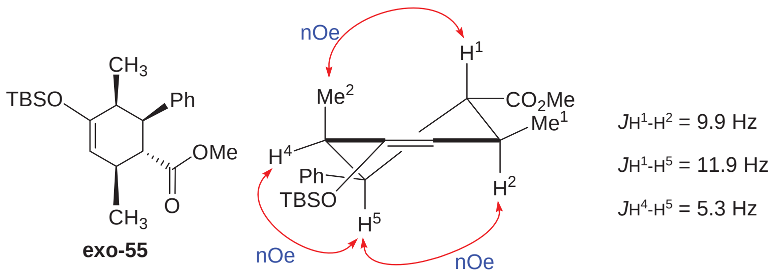

exo-55

For **exo-55**: m.p. 40–42 °C; IR (thin film): ν 3030, 2957, 2932, 1739, 1668, 1602, 1359, 1255, 1191, 1165, 840, 779, 701 cm^{-1}; ^{1}H NMR (600 MHz, C_6D_6): δ 7.13–7.12 (m, 4H, Ar-H), 7.04–7.02 (m, 1H, Ar-H), 4.73 (d, J = 2.0 Hz, 1H, H^3), 3.57 (dd, J =5.3, 11.9 Hz, 1H, H^5), 3.15 (s, 3H, OMe), 2.84 (dqd, J = 2.0, 6.7, 9.9 Hz, 1H, H^2), 2.78 (dd, J = 9.9, 11.9 Hz, 1H, H^1), 2.31 (dq, J = 5.3, 7.0 Hz, 1H, H^4), 1.09 (d, J = 6.7 Hz, 3H, Me^1), 0.96 (s, 9H, Si-$C(CH_3)_3$), 0.85 (d, J = 7.0 Hz, 3H, Me^2), 0.16 (s, 3H, Si-CH_3), 0.15 (s, 3H, Si-CH_3); ^{13}C NMR (150 MHz, C_6D_6): δ 175.1 (C), 154.6 (C), 141.8 (C), 128.8 (CH × 2), 128.2 (CH × 2), 126.6 (CH), 107.2 (CH), 50.9 (CH_3), 47.8 (CH), 47.7 (CH), 40.3 (CH), 35.1 (CH), 25.8 (CH_3 × 3), 21.5 (CH_3), 18.2 (C), 14.5 (CH_3), –4.2 (CH_3), –4.6 (CH_3); HRMS (ESI): *m/z* calcd for $C_{22}H_{35}O_3Si$ ([M+ H]$^+$): 375.2355, found: 375.2351. The relative stereochemistry was determined by NOESY experiment and coupling constant analysis.

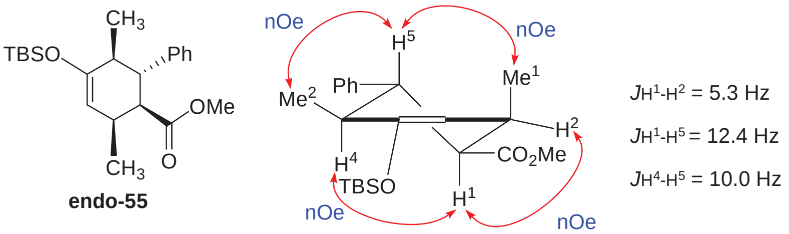

endo-55

For **endo-55**: IR (thin film): ν 3029, 2958, 2931, 2859, 1739, 1669, 1602, 1357, 1259, 1190, 1165, 840, 779, 701 cm^{-1}; ^{1}H NMR (600 MHz, C_6D_6): δ 7.15–7.12 (m, 4H, Ar-H), 7.05–7.02 (m, 1H, Ar-H), 4.97 (dd, J = 1.7, 5.7 Hz, 1H, H^3), 3.48 (dd, J = 5.3, 12.4 Hz, 1H, H^1), 3.13 (s, 3H, OMe), 3.03 (dd, J =10.0, 12.4 Hz, 1H, H^5), 2.85 (qd, J = 7.0, 10.0 Hz, 1H, H^4), 2.31 (ddq, J = 5.3, 5.7, 7.0 Hz, 1H, H^2), 1.33 (d, J = 7.0 Hz, 3H, Me^2), 0.98 (s, 9H, Si-$C(CH_3)_3$), 0.69 (d, J = 7.0Hz, 3H, Me^1), 0.16 (s, 3H, Si-CH_3), 0.15 (s, 3H, Si-CH_3); ^{13}C NMR (150 MHz, C_6D_6): δ 174.8 (C), 151.6 (C), 142.3 (C), 128.8 (CH × 2), 128.3 (CH × 2), 126.6 (CH), 109.6 (CH), 51.0 (CH_3), 48.5 (CH), 47.2 (CH), 39.3 (CH), 35.2 (CH), 25.9 (CH_3 × 3), 18.3 (C), 17.5 (CH_3 × 2), –4.3 (CH_3), –4.6 (CH_3); HRMS (ESI): *m/z* calcd for $C_{22}H_{35}O_3Si$ ([M+ H]$^+$): 375.2355, found: 375.2362. The relative stereochemistry was determined by NOESY experiment and coupling constant analysis.

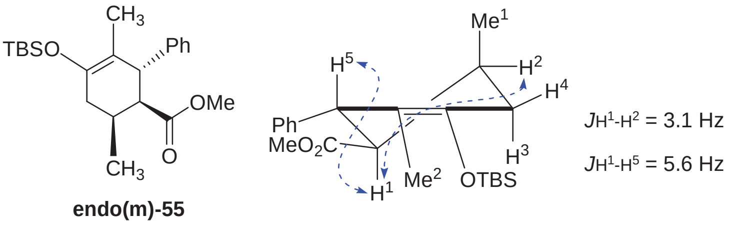

For **endo(m)-55**: IR (thin film): ν 3024, 2956, 2930, 2858, 1738, 1685, 1600, 1460, 1361, 1253, 1196, 1166, 837, 778, 702 cm^{-1}; 1H NMR (600 MHz, C_6D_6): δ 7.27 (d, *J* = 7.6 Hz, 2H, Ar-H), 7.17–7.15 (m, 2H, Ar-H), 7.04 (dd, *J* =7.6 Hz, 1H, Ar-H), 3.89 (d, *J* = 5.6 Hz, 1H, H^5), 3.25 (s, 3H, OMe), 2.73 (dd, *J* = 5.6, 3.1 Hz, 1H, H^1), 2.30 (dd, *J* = 7.4, 18.3 Hz, 1H, H^3), 2.17–2.15 (m, 2H, H^2 and H^4), 1.57 (s, 3H, Me^2), 1.02 (s, 9H, Si-$C(CH_3)_3$), 0.98 (d, *J* = 7.0 Hz, 3H, Me^1), 0.18 (s, 6H, Si-$(CH_3)_2$); ^{13}C NMR (150 MHz, C_6D_6): δ 173.4 (C), 144.7 (C), 144.5 (C), 129.1 (CH × 2), 128.7 (CH × 2), 126.7 (CH), 110.8 (CH), 52.8 (CH), 50.9 (CH_3), 47.5 (CH), 37.1 (CH_2), 28.4 (CH), 26.1 (CH_3 × 3), 18.4 (C), 17.0 (CH_3), 15.3 (CH_3), –3.5 (CH_3), –3.7 (CH_3); HRMS (APCI): *m/z* calcd for $C_{22}H_{35}O_3Si$ ($[M+H]^+$): 375.2355, found: 375.2346. The relative stereochemistry was determined by coupling constant analysis.

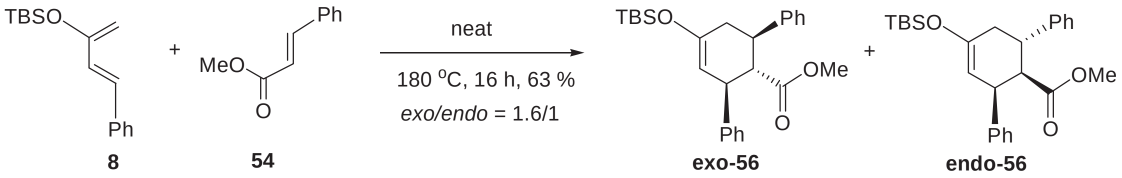

***Rac*-1-[2,6-diphenyl-4-(*tert*-butyldimethylsilyloxy)-cyclohex-3-enyl]-carboxylates (*exo*-56 and *endo*-56).** In a 25 mL sealed tube equipped with a magnetic stir bar, ester **54** (0.747 g, 4.62 mmol, 2.0 equiv.) and diene **8** (0.600 g, 2.31 mmol, 1.0 equiv.) were stirred for 16 h at 180 °C. Afterwards, the mixture was concentrated *in vacuo*. The residue was purified by column chromatography on silica gel (hexanes/ethyl acetate = 20/1) to give the corresponding Diels-Alder cycloadducts **exo-56** and **endo-56** (combined: 0.614 g, 63%) as a colorless oil. The diastereoselectivity of the crude products was determined by ^1H NMR in $CDCl_3$. Integration of the signal due to the vinylic proton (H^3) of the major adduct **exo-56** (δ_H 4.91 ppm) versus that of the minor adduct **endo-56** (δ_H 5.05 ppm) gave an *exo*/*endo* ratio of 1.6:1.

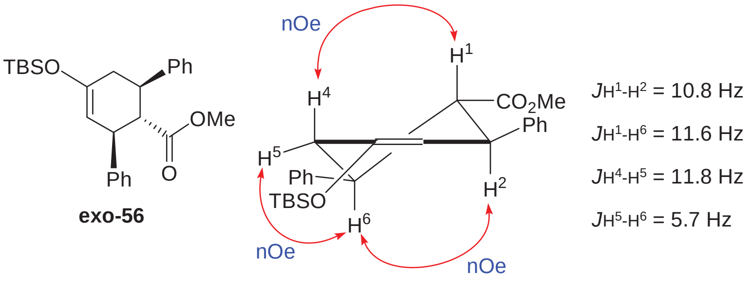

For **exo-56**: m.p. 101–102 °C; IR (thin film): ν 3028, 2953, 2930, 2858, 1741, 1672, 1602, 1454, 1254, 1201, 1159, 835, 800, 700 cm^{-1}; H NMR (600 MHz, $CDCl_3$): δ 7.28–7.25 (m, 4H, Ar-H), 7.20–7.17 (m, 6H, Ar-H), 4.91 (d, *J* = 2.0, 2.3 Hz, 1H, H^3), 3.87 (dddd, *J* = 2.0, 2.3, 3.9, 10.8 Hz, 1H, H^2), 3.28 (ddd, *J* = 5.7, 11.6, 11.8 Hz, 1H, H^6), 3.09 (s, 3H, OMe), 2.70 (dd, *J* = 10.8, 11.8 Hz, 1H, H^1), 2.46 (dddd, *J* = 2.3, 3.9, 11.8, 17.5 Hz, 1H, H^4), 2.34 (ddd, *J* = 2.0, 5.7, 17.5 Hz, 1H, H^5), 0.91 (s, 9H, Si-$C(CH_3)_3$), 0.17 (s, 3H, Si-CH_3), 0.15 (s, 3H, Si-CH_3); ^{13}C NMR (150 MHz, $CDCl_3$): δ 174.3 (C), 150.3 (C), 144.0 (C), 142.2 (C), 128.5 (CH × 2), 128.4 (CH × 2), 127.6 (CH × 2), 127.5 (CH × 2), 126.9 (CH), 126.8 (CH), 107.1 (CH), 56.0 (CH), 50.9 (CH_3), 46.0 (CH), 44.6 (CH), 38.0 (CH_2), 25.6 (CH_3 × 3), 18.0 (C), –4.3 (CH_3), –4.5 (CH_3); HRMS (ESI): *m/z* calcd for $C_{26}H_{34}O_3NaSi$ ($[M+ Na]^+$): 445.2175, found: 445.2181. The relative stereochemistry was determined by NOESY experiment and coupling constant analysis.

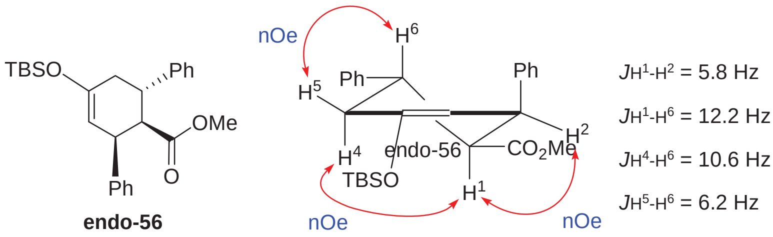

For **endo-56**: IR (thin film): ν 3029, 2954, 2930, 2857, 1736, 1668, 1603, 1463, 1362, 1255, 1176, 836, 779, 699 cm^{-1}; ^1H NMR (600 MHz, $CDCl_3$): δ 7.10–6.99 (m, 6H, Ar-H), 6.68 (d, *J*= 7.1 Hz, 2H, Ar-H), 6.61 (d, *J*= 6.7 Hz, 2H, Ar-H), 5.05 (dd, *J* = 1.5, 5.4 Hz, 1H, H^3), 3.65 (dd, *J* = 5.4, 5.8 Hz, 1H, H^2), 3.45 (dd, *J* = 5.8, 12.2 Hz, 1H, H^1), 3.35 (s, 3H, OMe), 3.22 (ddd, *J* = 6.2, 10.6, 12.2 Hz, 1H, H^6), 2.55 (ddd, *J* = 1.5, 10.6, 17.1 Hz, 1H, H^4), 2.50 (dd, *J* = 6.2, 17.1 Hz, 1H, H^5), 0.95 (s, 9H, Si-$C(CH_3)_3$), 0.20 (s, 3H, Si-CH_3), 0.19 (s, 3H, Si-CH_3); ^{13}C NMR (150 MHz, $CDCl_3$): δ 175.0 (C), 149.6 (C), 140.7 (C), 140.5 (C), 130.0 (CH × 2), 128.4 (CH × 2), 127.5 (CH × 2), 127.3 (CH × 2), 126.4 (CH), 126.2 (CH), 106.5 (CH), 51.5(CH_3), 47.4 (CH), 46.7 (CH), 40.3 (CH), 34.0 (CH_2), 25.7 (CH_3 × 3), 18.0 (C), –4.2 (CH_3), –4.4 (CH_3); HRMS (ESI): *m/z* calcd for $C_{26}H_{34}O_3NaSi$ ($[M+ Na]^+$): 445.2175, found:

445.2170. The relative stereochemistry was determined by NOESY experiment and coupling constant analysis.

2.6 Diels–Alder reactions with nitroolefins

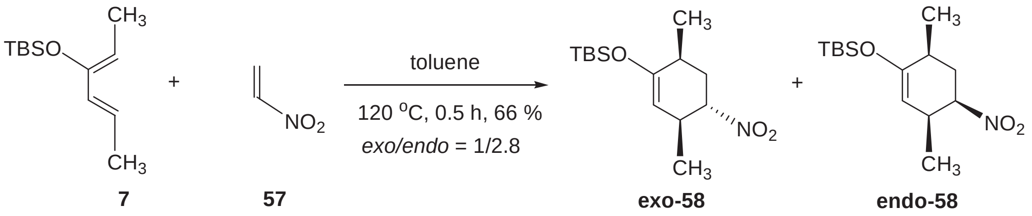

***Rac-tert*-butyl-(3,6-dimethyl-4-nitro-cyclohex-1-enyloxy)-dimethyl-silanes (exo-58 and endo-58).** In a 25 mL sealed tube equipped with a magnetic stir bar, a solution of nitroethene **57** (1.0 N in toluene, 1.88 mmol, 1.0 equiv.) and silyloxydiene **7** (0.600 g, 2.40 mmol, 1.5 equiv.) in toluene (1.9 mL) was stirred for 30 min at 120 °C. Afterwards, the mixture was concentrated *in vacuo*. The residue was purified by column chromatography on silica gel (hexanes/ethyl acetate = 20/1) to give the corresponding Diels–Alder cycloadducts **exo-58** and **endo-58** (combined: 0.36 g, 66%) as a colorless oil. The diastereoselectivity of the crude products was determined by ^{1}H NMR in $CDCl_3$. Integration of the signal due to the vinylic proton (H^3) of **exo-58** (δ_H 4.46 ppm) versus that of **endo-58** (δ_H 4.57 ppm) gave an *exo*/*endo* ratio of 1/2.8.

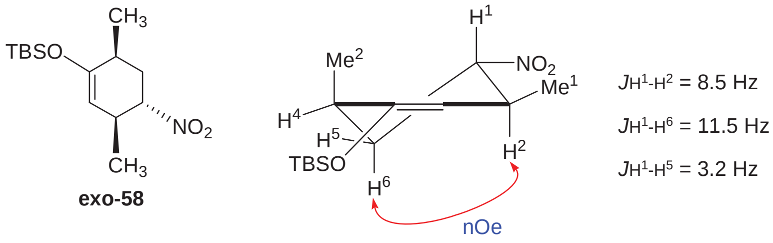

For **exo-58**: IR (thin film): ν 2966, 2934, 2855, 1718, 1547, 1458, 1377, 1347, 1254, 1176, 839, 780 cm^{-1}; ^{1}H NMR (600 MHz, $CDCl_3$): δ 4.46 (d, J = 2.4 Hz, 1H, H^3), 4.10 (ddd, J = 11.5, 8.5, 3.2 Hz, 1H, H^1), 2.88–2.86 (m, 1H, H^2), 2.16 (ddd, J = 12.7, 11.5, 6.3 Hz, 1H, H^6), 2.09–2.07 (m, 1H, H^4), 1.58 (ddd, J = 12.7, 3.2, 3.0 Hz, 1H, H^5), 0.99 (s, 9H, Si-C$(CH_3)_3$), 0.91 (d, J = 7.1 Hz, 3H, Me^2), 0.83 (d, J = 6.8 Hz, 3H, Me^1), 0.99 (s, 6H, Si-CH_3 x 2); ^{13}C NMR (150 MHz, $CDCl_3$): δ 153.5 (C), 105.1 (CH), 86.2 (CH), 34.4 (CH), 34.3 (CH_2), 32.9 (CH), 25.7 (CH_3 × 3), 19.6 (CH_3), 18.6 (CH_3), 18.1 (C), –4.5 (CH_3), –4.6 (CH_3); HRMS (ESI): *m/z* calcd for $C_{14}H_{27}NO_3N_aSi$ ($[M + Na]^+$): 308.1658, found: 308.1656. The relative stereochemistry was determined by NOESY experiment and coupling constant analysis.

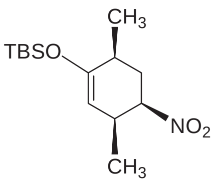

endo-58

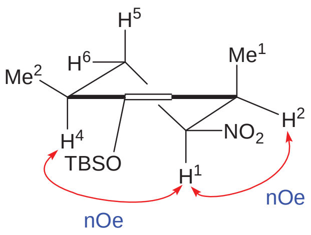

$J_{H^1\text{-}H^2}$ = 5.0 Hz

$J_{H^1\text{-}H^5}$ = 11.2 Hz

$J_{H^1\text{-}H^6}$ = 5.6 Hz

For **endo-58**: IR (thin film): ν 2960, 2932, 2859, 1657, 1548, 1464, 1389, 1220, 1169, 859, 840, 780 cm^{-1}; ^{1}H NMR (600 MHz, $CDCl_3$): δ 4.57 (dd, *J* = 5.7, 1.3 Hz, 1H, H^3), 4.15 (ddd, *J* = 11.2, 5.5, 5.0 Hz, 1H, H^1), 2.82 (qdd, *J* = 6.8, 5.7, 5.0 Hz, 1H, H^2), 1.92–1.86 (m, 1H, H^4), 1.82–1.75 (m, 2H, H^5 and H^6), 1.00 (s, 9H, Si-C(CH_3)$_3$), 0.98 (d, *J* = 6.9 Hz, 3H, Me^2), 0.81 (d, *J* = 6.9 Hz, 3H, Me^1), 0.11 (s, 6H, Si-CH_3 × 2); ^{13}C NMR (150 MHz, $CDCl_3$) δ 153.3 (C), 105.6 (CH), 83.6 (CH), 33.7 (CH), 32.8 (CH), 29.6 (CH_2), 25.8 (CH_3 × 3), 18.3 (CH_3), 18.2 (C), 16.0 (CH_3), –4.5 (CH_3), –4.7 (CH_3); HRMS (ESI): *m/z* calcd for $C_{14}H_{27}NO_3NaSi$ ([M + Na]$^+$): 308.1658, found: 308.1664. The relative stereochemistry was determined by NOESY experiment and coupling constant analysis.

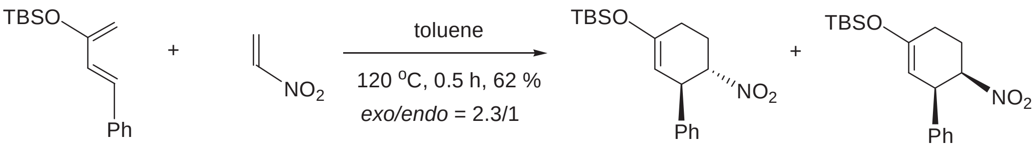

8 **57** **exo-59** **endo-59**

***Rac*-*tert*-butyl-dimethyl-(4-nitro-3-phenyl-cyclohex-1-enyloxy)-silanes (*exo*-59 and *endo*-59).** In a 25 mL sealed tube equipped with a magnetic stir bar, a solution of nitroethene **57** (1.0 N in toluene, 1.92 mmol, 1.0 equiv.) and silyloxydiene **8** (0.600 g, 2.31 mmol, 1.2 equiv.) in toluene (1.9 mL) was stirred for 30 min at 120 °C. Afterwards, the mixture was concentrated *in vacuo*. The residue was purified by column chromatography on silica gel (hexanes/toluene = 4/1) to give the corresponding Diels-Alder cycloadducts ***exo*-59** and ***endo*-59** (combined: 0.40 g, 62%) as a colorless oil. The diastereoselectivity of the crude products was determined by ^{1}H NMR in C_6D_6. Integration of the signal due to proton H^2 of the major isomer **exo-59** (δ_H 4.09 ppm) versus that of the minor isomer **endo-59** (δ_H 3.95 ppm) gave an *exo*/*endo* ratio of 2.3/1.

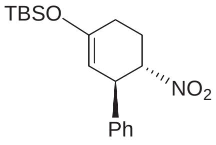

exo-59

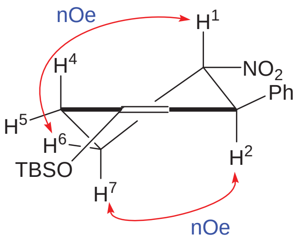

For **exo-59**: IR (thin film): ν 3028, 2956, 2932, 2857, 1666, 1543, 1255, 1206, 1177, 882, 837, 701 cm^{-1}; ^{1}H NMR (600 MHz, C_6D_6): δ 7.08–7.05 (m, 4H, Ar-H), 7.03–7.00 (m, 1H, Ar-H), 4.70 (s, 1H, H^3), 4.20 (ddd, 1H, J = 10.3, 7.9, 3.2 Hz, 1H, H^1), 4.10–4.08 (m, 1H, H^2), 1.99–1.93 (m, 1H, H^7), 1.89–1.77 (m, 2H, H^4 and H^5), 1.64–1.60 (m, 1H, H^6), 0.91 (s, 9H, Si-C(CH_3)$_3$), 0.03 (s, 3H, Si-CH_3), 0.01 (s, 3H, Si-CH_3); ^{13}C NMR (150 MHz, C_6D_6): δ 150.7 (C), 141.7 (C), 129.0 (CH × 2), 128.1 (CH × 2), 127.7 (CH), 104.4 (CH), 88.6 (CH), 45.4 (CH), 27.7 (CH_2), 26.4 (CH_2), 25.7 (CH_3 × 3), 18.0 (C), –4.4 (CH_3) , –4.5 (CH_3); HRMS (ESI): *m/z* calcd for $C_{18}H_{28}NO_3Si$ ([M + H]$^+$): 334.1838, found: 334.1839. The relative stereochemistry was determined by NOESY experiment analysis.

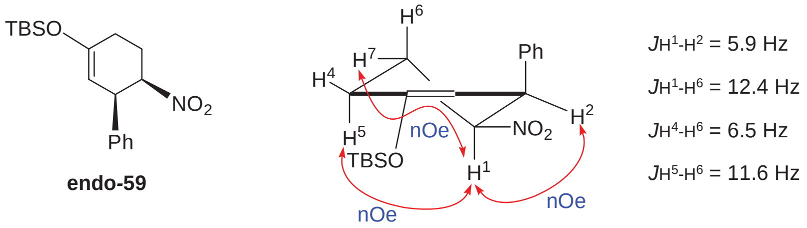

For **endo-59**: IR (thin film): ν 3027, 2955, 2932, 2857, 1667, 1543, 1256, 1206, 1176, 882, 836, 700 cm^{-1}; ^{1}H NMR (600 MHz, C_6D_6) δ 7.17–7.14 (m, 2H, Ar-H), 7.08–7.06 (m, 2H, Ar-H), 7.03–7.00 (m, 1H, Ar-H), 4.64 (dd, J = 5.4, 1.5 Hz, 1H, H^3), 4.21 (ddd, J = 12.4, 5.9, 3.2 Hz, 1H, H^1), 3.95 (dd, J = 5.9, 5.4 Hz, 1H, H^2), 2.00 (dddd, J = 17.6, 12.4, 11.6, 6.5 Hz, 1H, H^6), 1.91 (ddd, J = 18.0, 6.5, 1.0 Hz, 1H, H^4), 1.73 (dddd, J = 18.0, 11.6, 6.3, 1.5 Hz, 1H, H^5), 1.41 (dddd, J = 17.6, 6.3, 3.2, 1.0 Hz, 1H, H^7), 0.95 (s, 9H, Si-C(CH_3)$_3$), 0.06 (s, 3H, Si-CH_3), 0.05 (s, 3H, Si-CH_3); ^{13}C NMR (150 MHz; C_6D_6): δ 151.6 (C), 137.9 (C), 129.6 (CH × 2), 128.5 (CH × 2), 128.0 (CH), 103.6 (CH), 84.5 (CH), 43.8 (CH), 28.3 (CH_2), 25.7 (CH_3 × 3), 20.8 (CH_2), 18.0 (C), –4.3 (CH_3), –4.5 (CH_3); HRMS (ESI): *m/z* calcd for $C_{18}H_{28}NO_3Si$ ([M + H]$^+$): 334.1838, found: 334.1839. The relative stereochemistry was determined by NOESY experiment and coupling constant analysis.

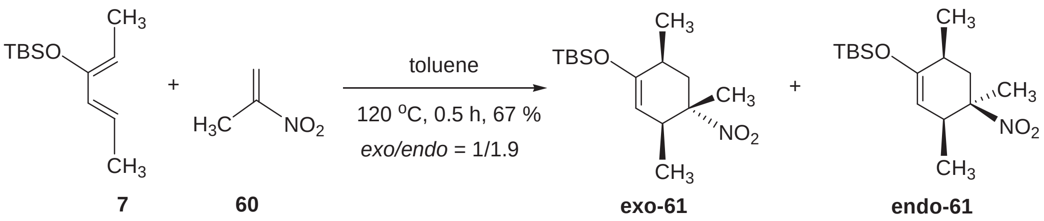

***Rac-tert*-butyl-dimethyl-(3,4,6-trimethyl-4-nitro-cyclohex-1-enyloxy)-silanes (exo-61 and endo-61).** In a 25 mL sealed tube equipped with a magnetic stir bar, a solution of 2-nitropropene (**60**, 1.0 N in toluene, 1.88 mmol, 1.0 equiv.) and silyloxydiene **7** (0.600 g, 2.40

mmol, 1.5 equiv.) in toluene (1.9 mL) was stirred for 30 min at 120 °C. Afterwards, the mixture was concentrated *in vacuo*. The residue was purified by column chromatography on silica gel (hexanes/ethyl acetate = 20/1) to give the corresponding Diels–Alder cycloadducts **exo-61** and **endo-61** (combined: 0.38 g, 67%) as a colorless oil. The diastereoselectivity of the crude products was determined by ^{1}H NMR in C_6D_6. Integration of the signal due to the vinylic proton (H^2) of **exo-61** (δ_H 4.72 ppm) versus that of ***endo*-61** (δ_H 4.50 ppm) gave an *exo*/*endo* ratio of 1/1.9.

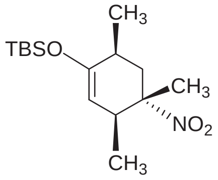

exo-61

For **exo-61**: IR (thin film): ν 2959, 2932, 2859, 1661, 1541, 1464, 1387, 1361, 1204, 866, 840, 779 cm^{-1}; ^{1}H NMR (600 MHz, C_6D_6): δ 4.72 (dd, J = 5.5, 1.3 Hz, 1H, H^2), 3.14–3.09 (m, 1H, H^1), 2.45–2.38 (m, 1H, H^3), 2.24 (ddd, J = 14.6, 6.7, 2.0 Hz, 1H, H^4), 1.12 (dd, J = 14.6, 9.8 Hz, 1H, H^5), 1.01 (s, 3H, Me^1), 0.99 (d, J = 7.0 Hz, 3H, Me^3), 0.92 (s, 9H, Si-C(CH_3)$_3$), 0.63 (d, J = 7.1 Hz, 3H, Me^2), 0.07 (s, 3H, Si-CH_3), 0.05 (s, 3H, Si-CH_3); ^{13}C NMR (150 MHz, C_6D_6): δ 153.2 (C), 107.3 (CH), 90.4 (C), 36.9 (CH_2), 36.0 (CH), 32.0 (CH), 25.8 (CH_3 × 3), 25.2 (CH_3), 18.9 (CH_3), 18.8 (CH_3), 18.1 (C), –4.3 (CH_3), –5.0 (CH_3); HRMS (ESI): *m/z* calcd for $C_{15}H_{29}NO_3NaSi$ ([M + Na]$^+$): 322.1814, found: 322.1813. The relative stereochemistry was determined by NOESY experiment analysis.

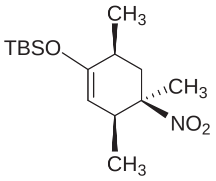

endo-61

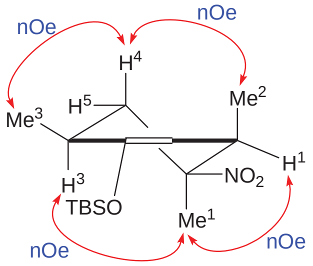

For **endo-61**: IR (thin film): ν 2959, 2931, 2859, 1690, 1663, 1540, 1254, 1192, 1179, 861, 839, 779 cm^{-1}; ^{1}H NMR (600 MHz, C_6D_6): δ 4.50 (dd, J = 5.8, 1.3 Hz, 1H, H^2), 2.40 (qd, J = 6.7, 5.8 Hz, 1H, H^1), 1.98 (dd, J = 14.6, 11.6 Hz, 1H, H^4), 1.84–1.76 (m, 2H, H^3 and H^5), 1.34 (s, 3H, Me^1), 0.96 (d, J = 6.6 Hz, 3H, Me^3), 0.92 (s, 9H, Si-C(CH_3)$_3$), 0.79 (d, J = 6.7 Hz, 3H, Me^2), 0.02 (s, 3H, Si-CH_3), 0.00 (s, 3H, Si-CH_3); ^{13}C NMR (150 MHz, C_6D_6): δ 152.1 (C), 105.7 (CH), 88.9 (C), 39.1 (CH), 34.8 (CH_2), 33.2 (CH), 25.8 (CH_3 × 3), 24.3 (CH_3), 18.5 (CH_3), 18.3 (CH_3), 18.2 (C), –4.4 (CH_3), –4.7 (CH_3); HRMS (ESI): *m/z* calcd for

$C_{15}H_{29}NO_3NaSi$ ([M + Na]$^+$): 322.1814, found: 322.1819. The relative stereochemistry was determined by NOESY experiment analysis.

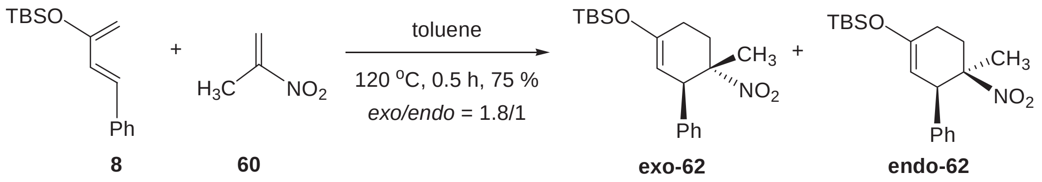

***Rac- tert*-butyl-dimethyl-(4-methyl-4-nitro-3-phenyl-cyclohex-1-enyloxy)-silanes (exo-62 and endo-62).** In a 25 mL sealed tube equipped with a magnetic stir bar, a solution of 2-nitropropene (**100**, 1.0 N in toluene, 1.92 mmol, 1.0 equiv.) and silyloxydiene **8** (0.60 g, 2.31 mmol, 1.2 equiv.) in toluene (1.9 mL) was stirred for 30 min at 120 °C. Afterwards, the mixture was concentrated *in vacuo*. The residue was purified by column chromatography on silica gel (hexane/toluene = 4/1) to give the corresponding Diels–Alder cycloadducts **exo-62** and **endo-62** (combined: 0.50 g, 75%) as a colorless oil. The diastereoselectivity of the crude products was determined by ^{1}H NMR in $CDCl_3$. Integration of the signal due to H^2 of the major isomer **exo-62** (δ_H 4.47 ppm) versus that of the minor isomer **endo-62** (δ_H 3.77 ppm) gave an *exo*/*endo* ratio of 1.8/1.

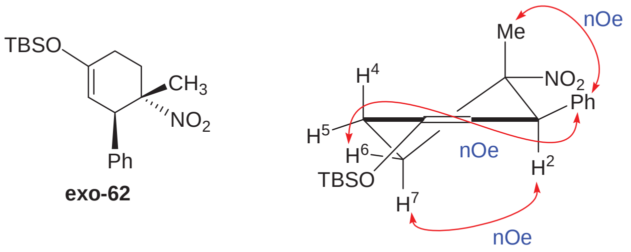

For **exo-62**: IR (thin film): ν 3028, 2953, 2931, 2857, 1667, 1542, 1256, 1206, 1176, 881, 839, 700 cm^{-1}; ^{1}H NMR (600 MHz, $CDCl_3$): δ 7.31 (dd, *J* = 7.4, 7.2 Hz, 2H, Ar-H), 7.26–7.25 (m, 1H, Ar-H), 7.19 (d, *J* = 7.2 Hz, 2H, Ar-H), 4.87 (d, *J* = 4.0 Hz, 1H, H^3), 4.47 (br, 1H, H^2), 2.53–2.49 (m, 1H, H^7), 2.32 (dt, *J* = 18.0, 6.9 Hz, 1H, H^4), 2.20 (dt, *J* = 18.0, 6.4 Hz, 1H, H^5), 1.96 (dt, *J* = 13.8, 6.9 Hz, 1H, H^6), 1.24 (s, 3H, CH_3), 0.92 (s, 9H, Si-C(CH_3)$_3$), 0.15 (s, 3H, Si-CH_3), 0.13 (s, 3H, Si-CH_3); ^{13}C NMR (150 MHz, $CDCl_3$): δ 150.5 (C), 139.5 (C), 129.4 (CH × 2), 128.3 (CH × 2), 127.5 (CH), 105.0 (CH), 90.3 (C), 47.8 (CH), 31.0 (CH_2), 27.1 (CH_2), 25.5 (CH_3 × 3), 22.6 (CH_3), 17.9 (C), –4.3 (CH_3), –4.5 (CH_3); HRMS (ESI): *m/z* calcd for $C_{19}H_{30}NO_3Si$ [M + H]$^+$: 348.1995, found: 348.1991. The relative stereochemistry was determined by NOESY experiment.

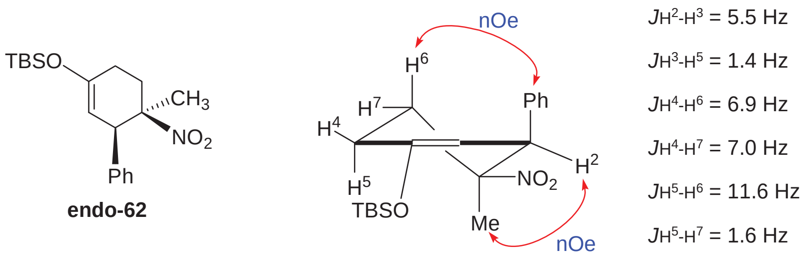

For **endo-62**: m.p. 64–65 °C; IR (thin film): ν 3029, 2952, 2930, 2855, 1668, 1541, 1258, 1204, 1178, 882, 838, 702 cm^{-1}; ^{1}H NMR (600 MHz, $CDCl_3$): δ 7.26–7.25 (m, 2H, Ar-H), 7.23–7.22 (m, 1H, Ar-H), 7.17–7.16 (m, 2H, Ar-H), 4.85 (dd, *J* = 5.5, 1.4 Hz, 1H, H^3), 3.77 (d, *J* = 5.5 Hz, 1H, H^2), 2.52 (ddd, *J* = 13.3, 11.6, 6.9 Hz, 1H, H^6), 2.40 (ddd, *J* = 18.0, 6.9, 1.4 Hz, 1H, H^4), 2.20 (dddd, *J* = 18.0, 11.6, 7.0, 1.6 Hz, 1H, H^5), 1.85 (dddd, *J* = 13.3, 7.0, 1.6 Hz, 1H, H^7), 1.82 (s, 3H, CH_3), 0.93 (s, 9H, Si-$C(CH_3)_3$), 0.16 (s, 3H, Si-CH_3), 0.15 (s, 3H, Si-CH_3); ^{13}C NMR (150 MHz, $CDCl_3$): δ 150.1 (C), 138.6 (C), 129.4 (CH × 2), 128.1 (CH × 2), 127.7 (CH), 104.0 (CH), 89.9 (C), 50.3 (CH), 28.0 (CH_2), 26.1 (CH_2), 25.6 (CH_3 × 3), 24.6 (CH_3), 17.9 (C), –4.2 (CH_3), –4.4 (CH_3); HRMS (ESI): *m/z* calcd for $C_{19}H_{29}NO_3SiNa$ ($[M + Na]^+$): 370.1814, found: 370.1806. The relative stereochemistry was determined by single-crystal X-ray diffraction data (racemic mixture), NOESY experiment and coupling constant analysis.

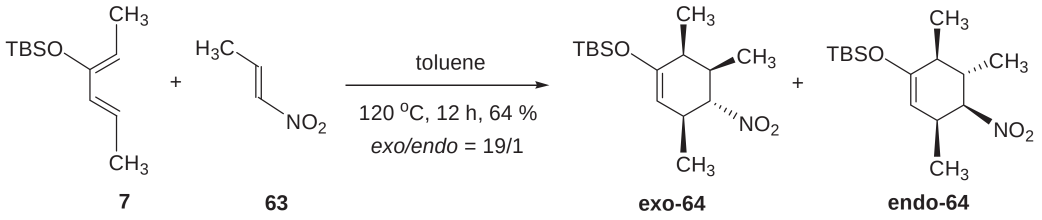

***Rac-tert*-butyl-dimethyl-(3,5,6-trimethyl-4-nitro-cyclohex-1-enyloxy)-silanes (exo-64 and endo-64).** In a 25 mL sealed-tube equipped with a magnetic stir bar, a solution of 1-nitropropene (**63**, 0.16 g, 1.88 mmol, 1.0 equiv.) and silyloxydiene **7** (0.60 g, 2.40 mmol, 1.5 equiv.) in toluene (1.9 mL) was stirred for 12 h at 120 °C. Afterwards, the mixture was concentrated *in vacuo*. The residue was purified by column chromatography on silica gel (hexanes/ethyl acetate = 20/1) to give the corresponding Diels–Alder cycloadducts **exo-64** and **endo-64** (combined: 0.36 g, 64%) as a colorless solid. The diastereoselectivity of the crude products was determined by ^{1}H NMR in C_6D_6. Integration of the signal due to the vinylic proton (H^3) of the major isomer **exo-64** (δ_H 4.36 ppm) versus that of the minor isomer **endo-64** (δ_H 4.54 ppm) gave an *exo*/*endo* ratio of 19/1.

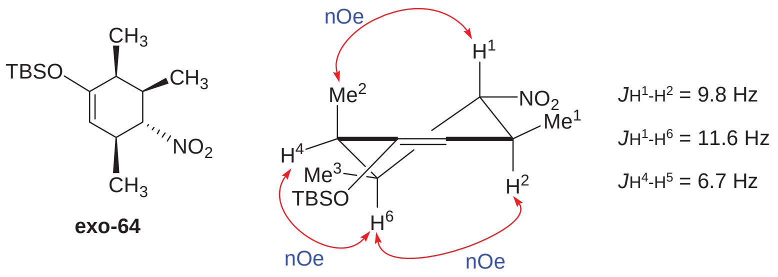

For **exo-64**: m.p. 58–60 °C; IR (thin film): ν 2961, 2931, 2859, 1664, 1551, 1463, 1374, 1338, 1254, 1203, 1181, 1059, 886, 839, 780 cm^{-1}; ^{1}H NMR (600 MHz, C_6H_6): δ 4.36 (d, *J* = 2.1 Hz, 1H, H^3), 3.96 (dd, *J* = 11.6, 9.8 Hz, 1H, H^1), 2.83 (dqdd, *J* = 9.8, 6.8, 2.1, 1.1 Hz, 1H, H^2), 2.29–2.23 (dqd, *J* = 11.6, 6.9, 6.7 Hz, 1H, H^5), 1.91 (qdd, *J* = 7.2, 6.7, 1.1 Hz, 1H, H^4), 0.93 (s, 9H, Si-C(CH_3)$_3$), 0.78 (d, *J* = 6.9 Hz, 3H, Me^1), 0.72 (d, *J* = 7.2 Hz, 3H, Me^2), 0.69 (d, *J* = 6.9 Hz, 3H, Me^3), 0.06 (s, 3H, Si-CH_3), 0.05 (s, 3H, Si-CH_3); ^{13}C NMR (150 MHz, C_6H_6): δ 154.0 (C), 104.7 (CH), 92.7 (CH), 39.0 (CH), 36.1 (CH), 35.6 (CH), 25.8 (CH_3 × 3), 19.4 (CH_3), 18.1 (C), 14.2 (CH_3), 13.0 (CH_3), –4.4 (CH_3), –4.7 (CH_3); HRMS (ESI): *m/z* calcd for $C_{15}H_{30}NO_3Si$ ([M + H]$^+$): 300.1995, found: 300.1992. The relative stereochemistry was determined by single-crystal X-ray diffraction data (racemic mixture), NOESY experiment and coupling constant analysis.

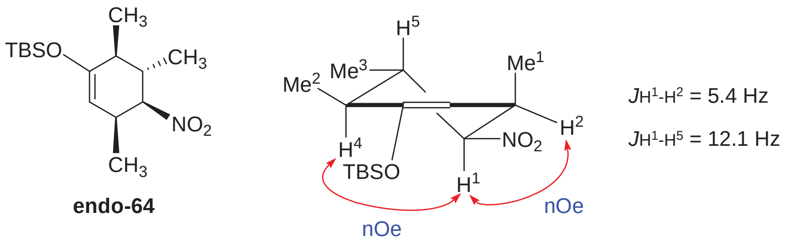

For **endo-64**: IR (thin film): ν 2962, 2930, 2859, 1667, 1551, 1464, 1375, 1338, 1256, 1206, 1182, 1059, 887, 837, 780 cm^{-1}; ^{1}H NMR (600 MHz, C_6H_6): δ 4.54 (dd, *J* = 6.2, 1.0 Hz, 1H, H^3), 4.21 (dd, *J* = 12.1, 5.4 Hz, 1H, H^1), 2.58–2.53 (m, 1H, H^2), 2.01–1.94 (m, 1H, H^5), 1.55–1.50 (m, 1H, H^4), 0.94 (d, *J* = 7.2 Hz, 3H, Me^2), 0.93 (s, 9H, Si-C(CH_3)$_3$), 0.83 (d, *J* = 7.0 Hz, 3H, Me^1), 0.74 (d, *J* = 6.9 Hz, 3H, Me^3), 0.05 (s, 3H, Si-CH_3), 0.03 (s, 3H, Si-CH_3); ^{13}C NMR (150 MHz, C_6H_6): δ 152.4 (C), 105.0 (CH), 91.4 (CH), 40.9 (CH), 33.7 (CH), 32.8 (CH), 25.7 (CH_3 × 3), 18.2 (C), 17.4 (CH_3), 16.9 (CH_3), 16.8 (CH_3), –4.5 (CH_3), –4.6 (CH_3); HRMS (ESI): *m/z* calcd for $C_{15}H_{30}NO_3Si$ ([M + H]$^+$): 300.1995, found: 300.2000. The relative stereochemistry was determined by NOESY experiment and coupling constant analysis.

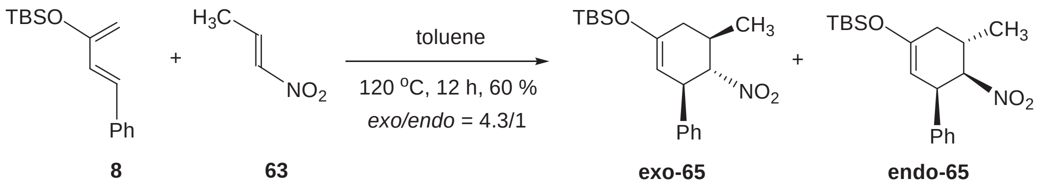

***Rac*-*tert*-butyl-dimethyl-(5-methyl-4-nitro-3-phenyl-cyclohex-1-enyloxy)-silanes (exo-65 and endo-65).** In a 25 mL sealed tube equipped with a magnetic stir bar, a solution of 1-nitropropene (**63**, 0.17 g, 1.92 mmol, 1.0 equiv.) and silyloxydiene **8** (0.60 g, 2.31 mmol, 1.2 equiv.) in toluene (1.9 mL) was stirred for 12 h at 120 °C. Afterwards, the mixture was concentrated *in vacuo*. The residue was purified by column chromatography on silica gel (hexane/ethyl acetate = 20/1) to give the corresponding Diels–Alder cycloadducts **exo-65** and **endo-65** (combined: 0.41 g, 60%) as a colorless oil. The diastereoselectivity of the crude products was determined by ^{1}H NMR in $CDCl_3$. Integration of the signal due to the vinylic proton (H^3) of the major isomer **exo-65** (δ_H 4.75 ppm) versus that of the minor isomer **endo-65** (δ_H 4.88 ppm) gave an *exo*/*endo* ratio of 4.3/1.

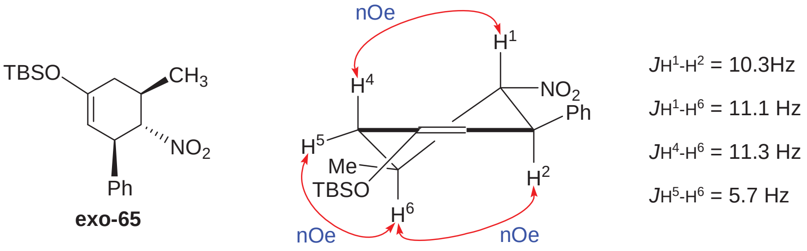

For **exo-65**: IR (thin film): ν 3026, 2952, 2931, 2858, 1666, 1541, 1258, 1203, 1178, 881, 838, 700 cm^{-1}; ^{1}H NMR (600 MHz, $CDCl_3$): δ 7.29–7.26 (m, 3H, Ar-H), 7.12 (d, J = 7.2 Hz, 2H, Ar-H), 4.75 (dd, J = 2.0, 1.7 Hz, 1H, H^3), 4.21 (dd, J = 11.1, 10.3 Hz, 1H, H^1), 4.06□4.04 (m, 1H, H^2), 2.54 (ddqd, J = 11.3, 11.1, 6.5, 5.4 Hz, 1H, H^6), 2.31 (ddd, J = 17.5, 5.7, 1.3 Hz, 1H, H^5), 2.07 (dddd, J = 17.5, 11.3, 3.6, 2.1 Hz, 1H, H^4), 1.00 (d, J = 6.5 Hz, 3H, CH_3), 0.91 (s, 9H, Si-$C(CH_3)_3$), 0.15 (s, 3H, Si-CH_3), 0.14 (s, 3H, Si-CH_3); ^{13}C NMR (150 MHz, $CDCl_3$): δ 149.8 (C), 141.0 (C), 128.9 (CH × 2), 127.7 (CH), 127.5 (CH × 2), 104.9 (CH), 96.9 (CH), 46.8 (CH), 37.1 (CH_2), 33.6 (CH), 25.5 (CH_3 × 3), 18.0 (C), 17.7 (CH_3), –4.4 (CH_3), –4.5 (CH_3); HRMS (ESI): *m/z* calcd for $C_{19}H_{30}NO_3Si$ ($[M + H]^+$): 348.1995, found: 348.1993. The relative stereochemistry was determined by NOESY experiment and coupling constant analysis.

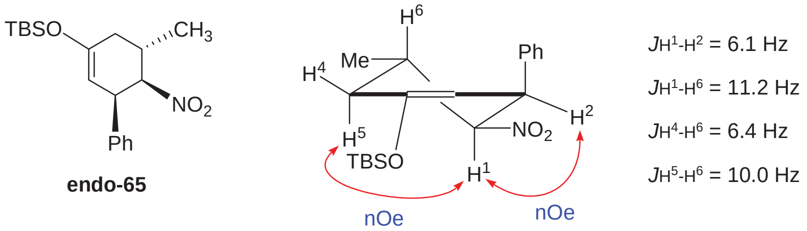

endo-65

For **endo-65**: IR (thin film): ν 3026, 2952, 2931, 2855, 1667, 1541, 1257, 1204, 1178, 882, 838, 701 cm^{-1}; ^{1}H NMR (600 MHz, $CDCl_3$): δ 7.26–7.22 (m, 3H, Ar-H), 7.12 (d, J = 7.2 Hz, 2H, Ar-H), 4.88 (dd, J = 5.4, 1.4 Hz, 1H, H^3), 4.64 (dd, J = 11.2, 6.1 Hz, 1H, H^1), 4.05 (ddd, J = 6.1, 5.4, 1.4 Hz, 1H, H^2), 2.50 (ddqd, J = 11.2, 10.0, 6.8, 6.4 Hz, 1H, H^6), 2.42 (dd, J = 17.7, 6.4 Hz, 1H, H^4), 1.96 (dddd, J = 17.7, 10.0, 1.8, 1.4 Hz, 1H, H^5), 0.98 (d, J = 6.4 Hz, 3H, CH_3), 0.93 (s, 9H, Si-C(CH_3)$_3$), 0.17 (s, 3H, Si-CH_3), 0.16 (s, 3H, Si-CH_3); ^{13}C NMR (150 MHz, $CDCl_3$): δ 150.6 (C), 138.1 (C), 128.9 (CH × 2), 128.3 (CH × 2), 127.9 (CH), 103.1 (CH), 92.0 (CH), 44.6 (CH), 36.6 (CH_2), 26.8 (CH), 25.6 (CH_3 × 3), 18.0 (C), 17.7 (CH_3), –4.3 (CH_3), –4.4 (CH_3); HRMS (ESI): *m/z* calcd for $C_{19}H_{29}NO_3SiNa$ ([M + Na]$^+$): 370.1814, found: 370.1814. The relative stereochemistry was determined by NOESY experiment and coupling constant analysis.

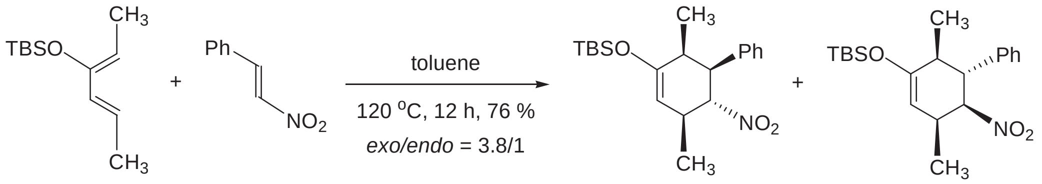

7 **66** **exo-67** **endo-67**

***Rac-tert*-butyl-(3,6-dimethyl-4-nitro-5-phenyl-cyclohex-1-enyloxy)-dimethyl-silanes (exo-67 and endo-67).** In a 25 mL sealed tube equipped with a magnetic stir bar, a solution of nitrostyrene (**66**, 0.14 g, 0.94 mmol, 1.0 equiv.) and silyloxydiene **7** (0.30 g, 1.20 mmol, 1.5 equiv.) in toluene (0.8 mL) was stirred for 12 h at 120 °C. Afterwards, the mixture was concentrated *in vacuo*. The residue was purified by column chromatography on silica gel (hexane/ethyl acetate = 20/1) to give the corresponding Diels–Alder cycloadducts **exo-67** and **endo-67** (combined: 0.26 g, 76%) as a colorless oil. The diastereoselectivity of the crude products was determined by ^{1}H NMR in C_6D_6. Integration of the signal due to proton H^5 of the major isomer **exo-67** (δ_H 3.69 ppm) versus that of the minor isomer **endo-67** (δ_H 3.10 ppm) gave an *exo/endo* ratio of 3.8/1.

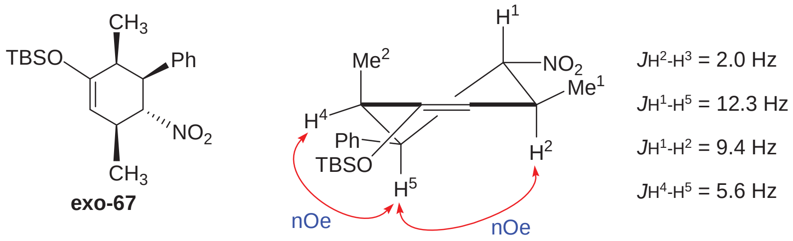

exo-67

For **exo-67**: m.p. 87–88 °C; IR (thin film): ν 3034, 2959, 2931, 2858, 1667, 1552, 1457, 1376, 1336, 1255, 1214, 1192, 1050, 859, 840, 780, 700 cm^{-1}; ^{1}H NMR (600 MHz, C_6D_6): δ 7.06 (dd, J = 7.4, 7.2 Hz, 2H, Ar-H), 7.00–6.97 (m, 3H, Ar-H), 4.67 (dd, J = 12.3, 9.5 Hz, 1H, H^1), 4.45 (d, J = 2.0 Hz, 1H, H^3), 3.69 (dd, J = 12.3, 5.6 Hz, 1H, H^5), 2.88 (dqd, J = 9.5, 6.8, 2.0 Hz, 1H, H^2), 2.24 (qd, J = 7.2, 5.6 Hz, 1H, H^4), 0.94 (d, J = 6.8 Hz, 3H, Me^1), 0.91 (s, 9H, Si-C(CH_3)$_3$), 0.68 (d, J = 7.2 Hz, 3H, Me^2), 0.10 (s, 3H, Si-CH_3), 0.07 (s, 3H, Si-CH_3); ^{13}C NMR (150 MHz, C_6D_6): δ154.0 (C), 138.1 (C), 128.6 (CH × 2), 128.2 (CH × 2), 127.4 (CH), 104.6 (CH), 89.3 (CH), 48.4 (CH), 40.6 (CH), 36.7 (CH), 25.7 (CH_3 × 3), 19.8 (CH_3), 18.0 (C), 14.6 (CH_3), –4.2 (CH_3), –4.8 (CH_3); HRMS (ESI): *m/z* calcd for $C_{20}H_{32}NO_3Si$ ([M + H]$^+$): 362.2151, found: 362.2147. The relative stereochemistry was determined by single-crystal X-ray diffraction data (racemic mixture), NOESY experiment and coupling constant analysis.

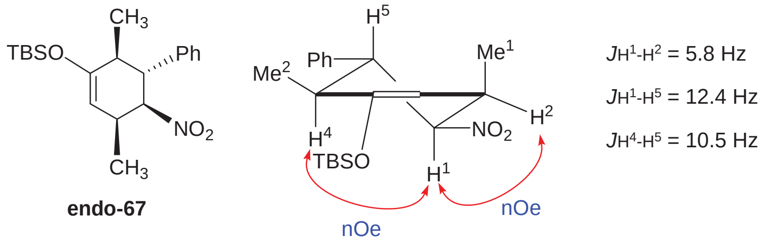

endo-67

For **endo-67**: IR (thin film): ν 3036, 2959, 2932, 2858, 1667, 1553, 1457, 1376, 1337, 1255, 1213, 1191, 1050, 858, 840, 781, 700 cm^{-1}; ^{1}H NMR (600 MHz, C_6D_6): δ 7.05 (dd, J = 7.4, 7.2 Hz, 2H, Ar-H), 6.99–6.97 (m, 3H, Ar-H), 4.95 (dd, J = 12.3, 5.8 Hz, 1H, H^1), 4.66 (dd, J = 5.7, 1.7 Hz, Hz, 1H, H^3), 3.10 (dd, J = 12.3, 10.0 Hz, 1H, H^5), 2.75 (qdd, J = 7.0, 5.8, 5.7 Hz, 1H, H^2), 2.17 (dqd, J = 10.0, 7.0, 1.7 Hz, 1H, H^4), 0.94 (d, J = 7.2 Hz, 3H, Me^1), 0.92 (d, J = 7.0 Hz, 3H, Me^2), 0.92 (s, 9H, Si-C(CH_3)$_3$), 0.007 (s, 3H, Si-CH_3), –0.013 (s, 3H, Si-CH_3); ^{13}C NMR (150 MHz; C_6D_6): δ 152.6 (C), 140.5 (C), 128.8 (CH × 2), 128.4 (CH × 2), 127.4 (CH), 105.0 (CH), 89.6 (CH), 46.6 (CH), 42.0 (CH), 33.0 (CH), 25.8 (CH_3 × 3), 18.2 (C), 17.4 (CH_3), 16.4 (CH_3), –4.5 (CH_3), –4.6 (CH_3); HRMS (ESI): *m/z* calcd for $C_{20}H_{32}NO_3Si$ ([M + H]$^+$): 362.2151, found: 362.2158. The relative stereochemistry was determined by NOESY experiment and coupling constant analysis.

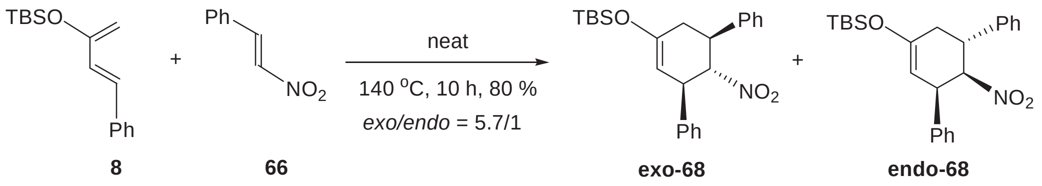

Rac- *tert*-butyl-dimethyl-(4-nitro-3,5-diphenyl-cyclohex-1-enyloxy)-silanes (*exo*-68 and *endo*-68). In a 25 mL sealed tube equipped with a magnetic stir bar, nitrostyrene (**66**, 0.29 g, 1.92 mmol, 1.0 equiv.) and silyloxydiene **8** (0.60 g, 2.31 mmol, 1.2 equiv.) were stirred for 10 h at 140 °C. Afterwards, the mixture was concentrated *in vacuo*. The residue was purified by column chromatography on silica gel (hexanes/ethyl acetate = 20/1) to give the corresponding Diels–Alder cycloadducts **exo-68** and **endo-68** (combined: 0.64 g, 80%) as a colorless oil. The diastereoselectivity of the crude products was determined by ^{1}H NMR in C_6D_6. Integration of the signal due to proton H^1 of the major isomer **exo-68** (δ_H 4.75 ppm) versus that of the minor isomer **endo-68** (δ_H 5.06 ppm) gave an *exo*/*endo* ratio of 5.7/1.

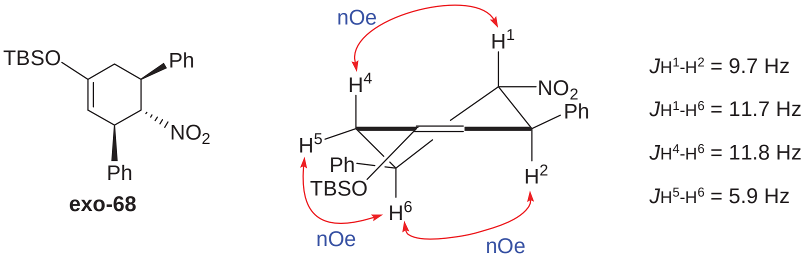

For **exo-68**: m.p. 162–163 °C; IR (thin film): ν 3030, 2952, 2930, 2857, 1665, 1552, 1374, 1182, 830, 699 cm^{-1}; ^{1}H NMR (600 MHz, C_6D_6): δ 7.20 (d, J = 7.3 Hz, 2H, Ar-H), 7.44 (dd, J = 7.5, 7.3 Hz, 2H, Ar-H), 7.05–7.01 (m, 3H, Ar-H), 6.99–6.93 (m, 3H, Ar–H), 4.83 (dd, J = 2.1, 1.7 Hz, 1H, H^3), 4.75 (dd, J = 11.7, 9.9 Hz, 1H, H^1), 4.20 (dddd, J = 9.9, 3.6, 1.7, 1.2 Hz, 1H, H^2), 3.49 (ddd, J = 11.8, 11.7, 5.9 Hz, 1H, H^6), 2.37 (dddd, J = 17.6, 11.8, 3.6, 2.1 Hz, 1H, H^4), 2.26 (ddd, J = 17.6, 5.9, 1.2 Hz, 1H, H^5), 0.92 (s, 9H, Si-C$(CH_3)_3$), 0.08 (s, 3H, Si-CH_3), 0.05 (s, 3H, Si-CH_3); ^{13}C NMR (150 MHz, C_6H_6): δ 150.3 (C), 141.3 (C), 139.0 (C), 129.2 (CH × 2), 129.0 (CH × 2), 128.1 (CH), 127.9 (CH), 127.8 (CH × 2), 127.6 (CH × 2), 104.8 (CH), 95.2 (CH), 47.7 (CH), 45.7 (CH), 37.7 (CH_2), 25.6 (CH_3 × 3), 18.0 (C), –4.4 (CH_3), –4.5 (CH_3); HRMS (ESI): *m/z* calcd for $C_{24}H_{31}NO_3SiNa$ ([M + Na]$^+$): 432.1971, found: 432.1970. The relative stereochemistry was determined by single-crystal X-ray diffraction data, NOESY experiment and coupling constant analysis.

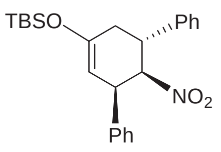

endo-68

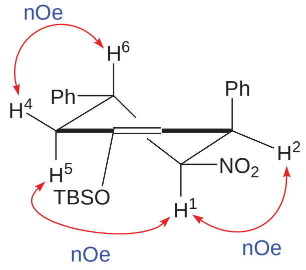

$J_{H^1\text{-}H^2}$ = 6.1 Hz

$J_{H^1\text{-}H^6}$ = 11.8 Hz

$J_{H^4\text{-}H^6}$ = 6.4 Hz

$J_{H^5\text{-}H^6}$ = 10.6 Hz

For **endo-68**: m.p. 140–142 °C; IR (thin film): ν 3029, 2953, 2931, 2856, 1665, 1553, 1374, 1182, 830, 701 cm^{-1}; ^1H NMR (600 MHz, C_6D_6): δ 7.22 (d, *J* = 7.6 Hz, 2H, Ar-H), 7.08–7.07 (m, 2H, Ar-H), 7.05–6.98 (m, 4H, Ar-H), 6.85 (d, *J* = 7.6 Hz, 2H, Ar-H), 5.06 (dd, *J* = 11.8, 6.1 Hz, 1H, H^1), 4.80 (dd, *J* =5.3, 1.3 Hz, 1H, H^3), 4.04 (dd, *J* = 6.1, 5.3 Hz, 1H, H^2), 3.53 (ddd, *J* = 11.8, 10.6, 6.4 Hz, 1H, H^6), 2.43 (dd, *J* = 17.6, 6.4 Hz, 1H, H^4), 2.19 (ddd, *J* = 17.6, 10.6, 1.3 Hz, 1H, H^5), 0.94 (s, 9H, Si-$C(CH_3)_3$), 0.098 (s, 3H, Si-CH_3), 0.092 (s, 3H, Si-CH_3); ^{13}C NMR (150 MHz, C_6H_6): δ 150.8 (C), 141.1 (C), 139.4 (C), 129.4 (CH × 2), 128.9 (CH × 2), 128.7 (CH × 2), 128.3 (CH), 127.4 (CH × 2), 127.3 (CH), 103.0 (CH), 89.7 (CH), 44.6 (CH), 38.7 (CH), 38.3 (CH_2), 25.6 (CH_3 × 3), 18.0 (C), –4.4 (CH_3), –4.5 (CH_3); HRMS (ESI): *m/z* calcd for $C_{24}H_{31}NO_3SiNa$ ($[M + Na]^+$): 432.1971, found: 432.1978. The relative stereochemistry was determined by NOESY experiment and coupling constant analysis.

Supplementary References

1. Dossetter, A. G., Jamison, T. F. & Jacobsen, E. N. Highly enantio- and diastereoselective hetero-Diels–Alder reactions catalyzed by new chiral tridentate chromium(III) catalysts. *Angew. Chem. Int. Ed.* **38**, 2398–2400 (1999).

2. Lestini, E., Robertson, K., Murphy, C. D. & Paradisi, F. Alternative mild route to the synthesis of 4-methylenecyclohex-2-enone, a key moiety of the anticancer compounds ottelione A and B. *Synth. Commun.* **42**, 1864−1876 (2012).
